# Mesoamerican Origin and Pre- and Post-Columbian Expansions of the Ranges of *Acanthoscelides obtectus* Say, a Cosmopolitan Insect Pest of the Common Bean

**DOI:** 10.1371/journal.pone.0070039

**Published:** 2013-07-31

**Authors:** Márcia Rodrigues Carvalho Oliveira, Alberto Soares Corrêa, Giselle Anselmo de Souza, Raul Narciso Carvalho Guedes, Luiz Orlando de Oliveira

**Affiliations:** 1 Laboratório de Biologia Molecular e Filogeografia, Instituto de Biotecnologia Aplicada à Agropecuária, Universidade Federal de Viçosa, Viçosa (MG), Brazil; 2 Departamento de Entomologia, Universidade Federal de Viçosa, Viçosa (MG), Brazil; Instituto de Higiene e Medicina Tropical, Portugal

## Abstract

An unprecedented global transfer of agricultural resources followed the discovery of the New World; one consequence of this process was that staple food plants of Neotropical origin, such as the common bean (*Phaseolus vulgaris*), soon expanded their ranges overseas. Yet many pests and diseases were also transported. *Acanthoscelides obtectus* is a cosmopolitan seed predator associated with *P. vulgaris*. Codispersal within the host seed seems to be an important determinant of the ability of *A. obtectus* to expand its range over long distances. We examined the phylogeographic structure of *A. obtectus* by (a) sampling three mitochondrial gene sequences (*12s rRNA*, *16s rRNA*, and the gene that encodes cytochrome c oxidase subunit I (COI)) throughout most of the species’ range and (b) exploring its late evolutionary history. Our findings indicate a Mesoamerican origin for the current genealogical lineages of *A. obtectus*. Each of the two major centers of genetic diversity of *P. vulgaris* (the Andes and Mesoamerica) contains a highly differentiated lineage of the bean beetle. Brazil has two additional, closely related lineages, both of which predate the Andean lineage and have the Mesoamerican lineage as their ancestor. The cosmopolitan distribution of *A. obtectus* has resulted from recent expansions of the two Brazilian lineages. We present additional evidence for both pre-Columbian and post-Columbian range expansions as likely events that shaped the current distribution of *A. obtectus* worldwide.

## Introduction

A significant global transfer of agriculturally valuable germplasm resources, referred to as the Columbian Exchange, occurred after the discovery of the New World [1]. One staple food plant of Neotropical origin that was included in the Columbian Exchange was the common bean (*Phaseolus vulgaris* L.), which left the Americas and reached Europe in 1506 [2].

Wild populations of *P. vulgaris* are found at altitudes ranging from 500 to 2000 m from Mexico to Northern Argentina. Two major gene pools are recognized: one in Mesoamerica and the other in the Andes [3]–[4]. A number of contrasting morphological, biochemical, and genetic traits, including partial reproductive isolation, set the Mesoamerican lineage apart from the Andean lineage of *P. vulgaris* [4]–[5] and suggest that, to a certain degree, these two major genealogical lineages evolved allopatrically under genetic isolation [6]. It has been proposed that either the Andes [7] or Mesoamerica [6] alone were the likely site of the origin of *P. vulgaris*.

Together with the transfer of useful plant and animal species, the Columbian Exchange resulted in the unintended spread of many new pests and diseases [8]. A cosmopolitan insect pest that causes severe damage to stored beans is the bean beetle *Acanthoscelides obtectus* Say 1831 (Coleoptera: Chrysomelidae: Bruchinae) [9]. This bruchid is a seed predator that feeds on wild and cultivated *P. vulgaris* [10], [11]. Several life-history traits of *A. obtectus*, especially its feeding behavior, favor its codispersal with the host as an important strategy to expand its range over long distances. Specifically, ovipositing females attack drying pods, their larvae penetrate the seed and feed on endosperm, and the larval and subsequent pupal stages together take about 23 days to complete [9]. Adult bruchids do not feed, are short-lived (with an average lifespan of 12 days) and are weak flyers [9], so it is unlikely that long-range dispersal could occur via the migration of adult bruchids. On the other hand, 23 days is enough time for human-mediated, long-distance dispersal to take place via the transport of infested seeds. Within an agricultural context, infestation begins in the field and continues as beans are moved to storage facilities, where the pest causes major losses [9]; the most plausible mechanism to account for the modern, cosmopolitan distribution of *A. obtectus* is human-mediated dispersion via infested beans. Currently, *A. obtectus* causes major economic losses, not only in the Neotropical ecozone, but also in the Palearctic, Afrotropical, and Indo-Malayan regions [2], [4], [11]–[13]. The geographic origin of *A. obtectus* remains unknown; the species may be of Mesoamerican [14] or Andean [11] origin.

It certainly appears that the finely tuned ecological interactions between *A. obtectus* and *P. vulgaris* [10]–[11], [15] would require a deep evolutionary timescale to evolve. We hypothesize that the strong dependence of *A. obtectus* on seeds of *P. vulgaris* as a source of food limits both natural and agriculturally related expansions of the range of *A. obtectus*. Although the range of P. vulgaris can be expanded in the absence of *A. obtectus*, the specialist bruchid may expand its range only to the extent that *P. vulgaris* successfully colonizes new territories. In terms of developments not influenced by human activities, codispersal events of *P. vulgaris* and *A. obtectus* may have led to phylogeographic congruence over time, which was a causal factor in the evolutionary history of the seed-predator insect (*A. obtectus*) to parallel that of its primary host (*P. vulgaris*). However, human-mediated activities in pre-Columbian times, such as the domestication of *P. vulgaris*, which was already taking place at about 8,000 years before present (yBP) [16], may have disturbed the pattern of phylogeographic congruence because it provided *A. obtectus* with an efficient mechanism for long-distance dispersal via human-mediated transport of infested beans as food or for sowing purposes. Subsequently, the Columbian Exchange provided unprecedented opportunities for both *P. vulgaris* and *A. obtectus* to reach and colonize even more distant habitats, including transoceanic dispersals.

In the study reported herein, we used a phylogeographic approach based on mitochondrial gene sequences to explore the late evolutionary history of *A. obtectus* and draw conclusions about the impact of human-mediated activities in the pre- and post-Columbian eras in shaping coevolutionary processes between *A. obtectus* and *P. vulgaris*. The following five questions were addressed: a) To what extent are the current genealogical lineages of *A. obtectus* associated with centers of genetic diversity of *P. vulgaris*? b) What is the likely geographic origin of the current genealogical lineages of *A. obtectus*? c) Is there any phylogeographic evidence for pre-Columbian range expansions of *A. obtectus*? Alternatively, are range expansions of *A. obtectus* part of the accidental introductions of new pests and diseases that followed the Columbian Exchange, or are both of these alternatives valid to some extent? d) Does the worldwide occurrence of *A. obtectus* have a single lineage source or multiple sources? e) What are the implications of this study for phytosanitary measures against invasive *A. obtectus*? By addressing these questions, molecular phylogeography can provide insights into the origin and current geographical distribution delimitation of prevailing genealogical lineages. This will, in turn, reveal plausible routes for unintended dispersal of the insect pest *A. obtectus* and enable the impact of human-mediated activities on shaping its current genetic diversity to be assessed.

## Methods

### Sampling Strategy

This study was carried out with an invasive pest insect for which no previous approval of the Committee on the Ethics of Animal Experiments of the Federal University of Viçosa was necessary. Field studies did not involve endangered or protected species. No collecting permits were required as this is an invasive, alien pest insect of cosmopolitan occurrence. When infested beans were collected in the field, we had the owner's verbal permission.

Specimens of *A. obtectus* were obtained from infested *P. vulgaris* beans from 25 sites spread throughout the main cultivation areas in Brazil. Infested beans were acquired directly from smallholder farmers with a history of using traditional cultivation systems, including on-farm seed production. Most of the samples belonged to the Mesoamerican gene pool, but we also obtained a few samples from varieties of the Andean gene pool (these latter came from Southern Brazil). We deliberately avoided sampling *A. obtectus* when the host crop was grown in large areas and under intensive agricultural practices, to avoid populations under intense gene flow. Emerging specimens were fixed in 70% ethanol and kept at –20°C until further use. To confirm the identification, a stereomicroscope was used to inspect bruchids for the presence of the characteristic morphological traits of *A. obtectus*, the most obvious of which are the presence of spiny protrusions on the hind legs [17] (Rees 1996). In addition to the samples from Brazil, specimens of *A. obtectus* were collected from Ecuador (1 site), Peru (4), Colombia (2), and South Africa (1). The specification of the location of each collection site was based on the coordinates of the nearest town.

### DNA Extraction, PCR Amplification, and Sequencing

Total genomic DNA was extracted as described [18]. Polymerase chain reaction (PCR) was performed according to standard PCR protocols [11] to amplify the three mitochondrial genes that encode (a) 12S ribosomal RNA (12s rRNA), (b) 16S ribosomal RNA (16s rRNA), and (c) the cytochrome oxidase subunit I (COI). Single PCR products for each of the three reactions were obtained for every specimen analyzed. All PCR amplicons were sequenced using the DNA sequencing services of Macrogen Inc., South Korea (www.macrogen.com). Sample sizes varied according to sites and studied loci (Table S1 in File S1). For the slowly evolving 12s rRNA gene, we sequenced 42 specimens; for the 16s rRNA gene, 66 specimens; and for the rapidly evolving COI gene, 139 specimens. All sequences were deposited in GenBank with the following accession numbers: 12s rRNA, KF157282–KF157323; 16s rRNA, KF157324–KF157389; and COI, KF157143–KF157281.

### Assembly of Datasets

All sequences were imported into Sequencher version 4.8 (Gene Codes Corp.) for editing. The sequence alignments were corrected and adjusted manually, due to the presence of a 1-bp insertion/deletion event (indel) within the 16S rRNA. This indel involved the extension or contraction of a repetitive sequence of nucleotides of the same type (i.e., A_8_ versus A_9_) and was not included in subsequent analyses.

Additional sequences were obtained from GenBank. For the 16s RNA and 12s RNA genes, we included in our analysis 23 sequences each (accessions AY826461–AY826480 for the 16s gene; AY826433–AY826457 for the 12S gene). These sequences were obtained from specimens collected in Mexico (N = 9), Peru (10), Cameroon (1), Switzerland (1), Spain (1), and France (1). For the COI gene, we included a total of 52 sequences: accessions AY676622–AY676647, AY826483–AY826505, AY881196–AY881201, FJ465153, and AY947519. These sequences are from specimens obtained in Mexico (32), Peru (10), Cameroon (3), Switzerland (3), Spain (1), France (1), China (1), and Egypt (1). After we had aligned all the sequences, we pruned their ends to eliminate fragments that could not be obtained for all specimens. The COI sequence of the Egyptian specimen (GenBank accession AY947519) was used only to assign lineage status, because it was not complete; it lacked 227 bases of the 736 bases we used in our analyses (see results for details).

Finally, we assembled the sequences into four preliminary datasets. There was one dataset for each studied gene: dataset S1 (12s rRNA, N = 65, 378 bp; dataset S1 in File S1); dataset S2 (16s rRNA, N = 89, 465 bp; dataset S2 in File S1); and dataset S3 (COI, N = 191, 736 bp; dataset S3 in File S1); we also assembled dataset S4, which contained the three sequences concatenated (N = 56, 1579 bp; dataset S4 in File S1). Distinct datasets needed to be assembled throughout the study, to accommodate the particular requirements of each analysis performed.

### Inspection for Nuclear Mitochondrial Paralogs (numts)

With the help of MEGA version 5 [19], we inspected dataset S3 for the presence of nuclear paralogs of the mitochondrial COI gene, hereafter called COI numts [20]. Specifically, we inspected each COI sequence for the following signatures of numts: 1) indels that introduce frameshifts, 2) out-of-place in-frame stop codons that would terminate protein translation prematurely, and (3) lack of codon position substitution bias towards the third position, which would lead to a higher rate of nonsynonymous mutations. We considered the presence of signatures 1 and 2 to be sufficient to regard a sequence as a COI numt; in the presence of signatures 1 and 2, we used signature 3 to confirm the numt status of a given sequence. Nevertheless, we refrained from using the presence of signature 3 alone to declare a COI sequence as a numt.

### Bayesian Phylogeny

Dataset S1, dataset S2 and dataset S3 were input independently to the software MRMODELTEST v2.3 [21]. Nucleotide substitution models were selected for each of the three datasets using the Akaike Information Criterion [22]. Using dataset S4 (containing concatenated versions of the three sequences) and the best-fit models F81 (dataset S1), HKY (dataset S2), and HKY+I (dataset S3), we ran a partition analysis using MRBAYES v3.1.2 [23] to obtain an unrooted Bayesian phylogeny. Bayesian analysis was performed using two simultaneous runs of 10 million generations each, with one cold and three heated chains in each run. Trees were sampled once every 10,000 generations; the first 250 trees were discarded as burn-in samples. A 50%-majority-rule consensus tree of the two independent runs was obtained with posterior probabilities that were equal to bipartition frequencies.

### Network Analyses

To infer genetic connections among lineages of *A. obtectus*, we reconstructed a haplotype network for each of the three mitochondrial genes. For this analysis, we excluded four variable sites of dataset S3 (40, 118, 120, and 717; having haplotype 1 as a reference sequence) because they display more than two character states. The infinite-sites model assumes that the mutation rate within a given DNA sequence is so small that only a single mutation can occur at any homologous position [24]. Those four sites violated the infinite-sites model and would introduce homoplasious relationships among haplotypes in the network. The removal of the four sites from dataset S3 generated dataset S5 (N = 191, 732 bp; dataset S5 in File S1). Gene genealogies for each of the three loci (dataset S1, dataset S2, and dataset S5) were inferred independently using the median-joining (MJ) network method [25] as implemented in NETWORK 4.5.0.2 (Fluxus Technology Ltd.) with default parameters. Finally, we mapped the geographic location of the occurrence of each haplotype using the geographic coordinates we had recorded previously; for the GenBank accessions, we used information that the collector had provided. Simple regression analyses ("least squares" method) explored whether the number of haplotypes and haplotype diversity were related to sample size (performed using Microsoft Excel).

### Inferences about Demographic History

For the studies concerned with demographic history, we refrained from using information from four sequences found in two Mesoamerican populations (XOT and YOH, respectively). The removal of these four sequences from dataset S5 generated dataset S6 (N = 187, 732 bp; dataset S6 in File S1). We used the entire dataset S6, but also split it into groups to reflect the geographic origin of the specimens and the mitogroups uncovered in the preceding analyses. The group ‘Andes’ (N = 51) comprised sequences of Peruvian, Ecuadorian, or Colombian origin; ‘Mesoamerica’ (N = 30) included sequences of Mexican origin exclusively; and ‘Old World’ (N = 18) contained sequences from Africa, Europe, and China. ‘Brazil’ (N = 88) was split into two subsets: ‘Brazil1’ (N = 78) and ‘Brazil2’ (N = 10), according to the mitogroups we uncovered in previous analyses. After forming the groups, we used DNAsp v5 [26] to estimate measures of nucleotide diversity (number of haplotypes, H; haplotype diversity, H_d_; nucleotide diversity, pi; and average number of nucleotide differences, k) for each group.

Tests of selective neutrality, Tajima’s D [27] and Fu’s Fs [28], were performed in ARLEQUIN v3.5 [29]. These two tests can distinguish between DNA sequences that harbor randomly evolving mutations (neutrality) from those that evolved via nonrandom processes, such as selection or demographic changes. Simulations have shown that Fu’s Fs is more effective in indicating population expansion than Tajima’s D [28]. Significant negative values of D or Fs indicate an excess of low-frequency polymorphisms and support population expansion or purifying selection, whereas significant positive values of D or Fs indicate fewer than expected low-frequency polymorphisms and point to bottlenecks or balancing selection. Nonsignificant values are consistent with the null hypothesis of neutrally evolving DNA. The neutrality tests were tested for significance by generating 1,000 random samples using coalescent simulations. Following the recommendation in the ARLEQUIN manual, the “Infer from distance matrix” option for “Haplotype definition” was activated; Fu’s Fs statistics were considered as significant at the 5% level if *P*<0.02.

For each distribution, ARLEQUIN also estimated tau (τ) with its 95% confidence intervals using a generalized least-squares approach and 1,000 coalescent simulations [30]. The parameter τ denotes the age of the expansion and corresponds to the mode of the mismatch distribution [31]. In equation t = τ/2 m_t_μ, t is the time since the expansion event occurred, τ is the number of generations since the expansion, μ is the mutation rate per site per generation, and m_t_ is the length of sequence. For *A. obtectus*, μ is not known and the number of generations per year cannot be estimated straightforwardly, given that specimens of distinct generations may coexist within a given population. For these reasons, we did not estimate t directly. However, assuming that neither μ nor average generation span varied significantly among the sampled populations, the term 2 m_t_μ becomes a constant, which allows values of τ to be compared among populations: a smaller value of τ suggests a newly established population, whereas a larger value of τ indicates an older one.

To explore the demographic history of *A. obtectus* further, we used ARLEQUIN to conduct mismatch distribution analysis using a spatial expansion model [31] on dataset S6. This model assumes that subdivided populations expanded their distribution and sizes over time [32]. Whereas a population that experienced a sudden expansion in its range is expected to produce a smooth, unimodal distribution, a population with a constant size yields a ragged, multimodal distribution. Parametric bootstrapping (1,000 replicates) tested the goodness-of-fit of the observed mismatch distribution to that expected under the spatial expansion model. The sum of square deviations (SSD) as a test statistic and its associated *P*-value were calculated using ARLEQUIN. A nonsignificant SSD value means that the null hypothesis of population expansion cannot be rejected. A nonsignificant raggedness index suggests a good fit of the data to the spatial expansion model.

### Divergence Dating

To estimate the time of divergence among lineages, we used the strict clock method as implemented in BEAST 1.7.2 [33] with sequence information from dataset S6 and the best-fit model (HKY+I) we had obtained previously with the Akaike Information Criterion in MRMODELTEST v2.3 as the substitution model. The 187 sequences of dataset S6 were split into ‘Taxon Sets’ following two alternative grouping possibilities: (A) based on three geographic origins (‘Mesoamerica’, ‘Andes’, and ‘Brazil+Old World’), or (B) based on the four mitogroups (I to IV) we uncovered previously (see results section). The option “Coalescent: Constant Size” was used as the tree prior, and the option “random starting tree” was chosen for the tree model.

For the analyses based on geographic origin, we calibrated BEAST with normally distributed prior probabilities for the age of the most recent common ancestor (tMRCA) following three scenarios:

1 Hypothesis 500: The tMRCA for ‘Brazil+Old World’ was placed at 500 yBP, with a standard deviation (SD) of 1, implying a post-Columbian origin for ‘Brazil+Old World’ [11];
2 Hypothesis 7000a: The tMRCA of ‘Mesoamerica’ was placed at 7,000 yBP (with SD of 1), which is the age attributed to the oldest record of *P. vulgaris* cultivation in Mesoamerica, found in the Coxcatlan Cave, Mexico [16];
3 Hypothesis 8000a: The tMRCA of ‘Andes’ was set to 8,000 yBP (with SD of 1), which is the date of the earliest archeological evidence of *P. vulgaris* cultivation in the Andean region, found in the Guitarrero cave, Peru [32].

For the analyses based on mitogroups, we calibrated BEAST with normally distributed prior probabilities following two additional scenarios:

4 Hypothesis 7000b: The tMRCA of Mitogroup I was placed at 7,000 yBP (with SD of 1), as in hypothesis 7000a;
5 Hypothesis 8000b: The tMRCA of Mitogroup II was set to 8,000 yBP (with SD of 1), as in hypothesis 8000a.

Analyses for each of these five hypotheses were run for 100 million generations, with samples taken every 1000 generations, with three replicates per run to allow chains to converge and mix adequately. With the help of TRACER 1.5 [31], final analyses were carried out by combining the three logs relative to each hypothesis. These settings ensured that both the model parameters and time estimates were sampled adequately (Effective Sample Size, ESS, was above 200 for all statistics in each independent run and above 1,000 in each combined analysis).

## Results

### Divergent COI Sequences in Mesoamerica, not Numts

Inspection of both DNA and protein sequence alignments revealed that neither indels nor in-frame stop codons were present; this ruled out two prevalent signatures associated with numts. Next, we investigated the pattern of nucleotide substitutions, taking haplotype 1 as the reference sequence. Most substitutions occurred at the third position of the codon. However, seven sequences from Mesoamerica (MAL_2, SJS_1, SJS_3, SPT_2, SPT_3, TLA_2, and YAU_1) were highly divergent. These seven sequences displayed several synonymous and several non-synonymous substitutions, ranging from five (in YAU_1) to 19 (in TLA_2). Five out of these seven sequences were obtained from bruchids that had been sampled from wild populations of *P. vulgaris* in Mesoamerica [10]–[11]. Given that none of the seven sequences contained either indels or in-frame stop codons and were obtained mostly from *A. obtectus* captured on wild *P. vulgaris*, we did not declare them as numts; rather, we considered them to be naturally occurring polymorphisms of the COI gene of *A. obtectus*.

### Unrooted Bayesian Phylogeny

**Figure 1:**
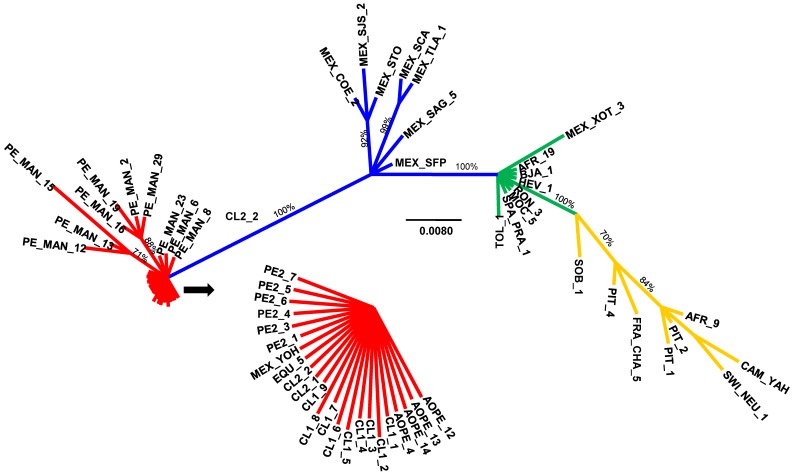
Unrooted Bayesian phylogenetic analysis of three mitochondrial genes (COI, 16s rRNA, and 12s rRNA) of *A. obtectus*. Branch lengths are drawn to scale; nodal support values are given as posterior probabilities in percent above the branches in cases with values higher than 70%. The scale bar corresponds to the expected number of substitutions per site. Codes are as given in Table S1 in File S1; for the remaining populations, see Alvarez *et al.* 2004 and 2005. Major clades have been color-coded for reference purposes.

A consensus tree displayed Mesoamerican specimens and Andean specimens in exclusive clades, with a posterior probability of 1, with YOH of Mesoamerican origin amongst Andean specimens being the only exception (Figure 1). Nested within the Mesoamerican clade and with a posterior probability of 100, there was a subclade of specimens of Brazilian, African, and European origin together with a single specimen of Mesoamerican origin (XOT_3). Within this subclade, the tree did not provide further resolution according to geographic origin; there were seven Mesoamerican specimens in basal positions and a terminal clade with specimens of distinct geographic origins.

### rRNA Genealogical Networks and Geographic Origins

**Figure 2:**
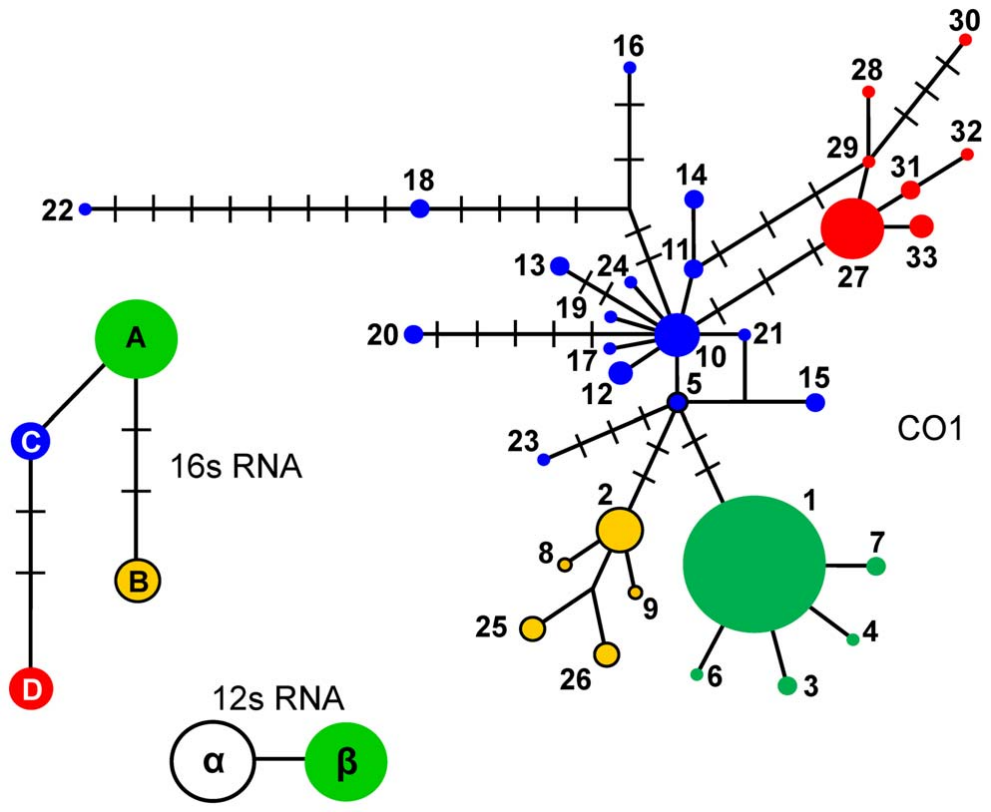
Median-joining networks depicting the relationships among genealogical lineages of *A. obtectus*. In each network, circles represent either COI haplotypes (coded with numbers), 16s rRNA haplotypes (coded with letters), or 12s RNA haplotypes (coded with Greek letters); circle size is proportional to the relative frequencies. Numbers of substitutions are indicated with bars when there is more than one. Major mitochondrial lineages have been color-coded for reference purposes.

**Figure 3:**
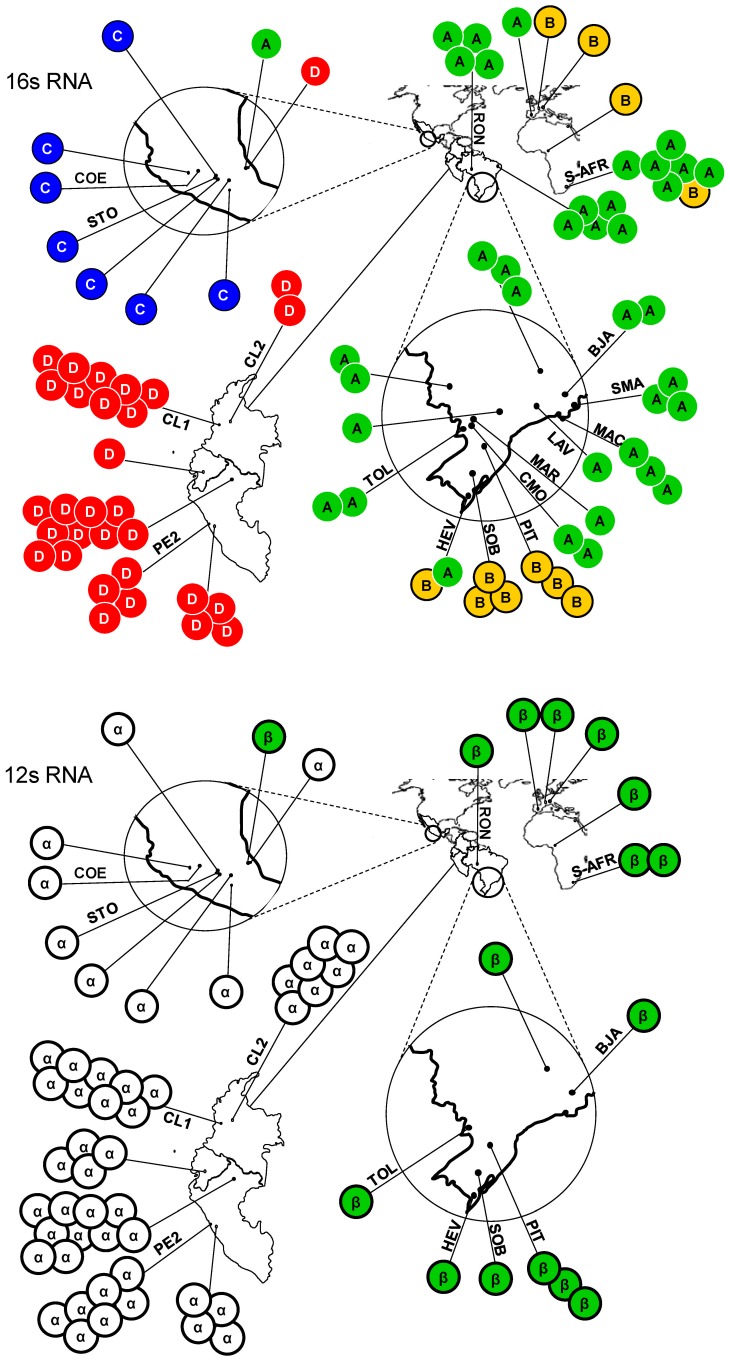
Geographic distributions of the 16s rRNA and 12s rRNA haplotypes of *A. obtectus.* Distributions of the four 16s RNA haplotypes are shown at the top, whereas the distributions of the two 12s RNA haplotypes are shown at the bottom. Each circle represents the haplotypes found in a given population; a letter within a circle denotes the rRNA haplotypes found in that population. Codes are as given in Table S1 in File S1; for the remaining populations, see Alvarez *et al.* 2004 and 2005. Major mitochondrial lineages have been color-coded for reference purposes.

Three independent networks (Figure 2) assessed genealogical relationships among lineages in the context of distinct evolutionary rates. The haplotype network that was based on the 12s rRNA gene, the slowest evolving gene in our study, contained only two closely related haplotypes (α and β) and exhibited a remarkable geographic assignment (Figure 3). The haplotype network that was based on the 16s rRNA gene, a gene that has evolved faster than the 12s rRNA gene but slower than the COI gene, recovered four haplotypes, named A through D (Figure 2). Again, the haplotypes were differentiated clearly according to their geographic origin (Figure 3).

### COI Genealogical Network and Geographic Origins

The haplotype network that was constructed using data obtained from the rapidly evolving COI gene (Figure 2) showed a much more complex structure than those we obtained for each of the two slowly evolving rRNA genes. Herein we used the term ‘mitogroup’ to refer to a subgroup of closely related haplotypes, which usually contains a high-frequency haplotype surrounded by middle- or low-frequency haplotypes. With 33 haplotypes, the COI network displayed four mitogroups.

**Figure 4:**
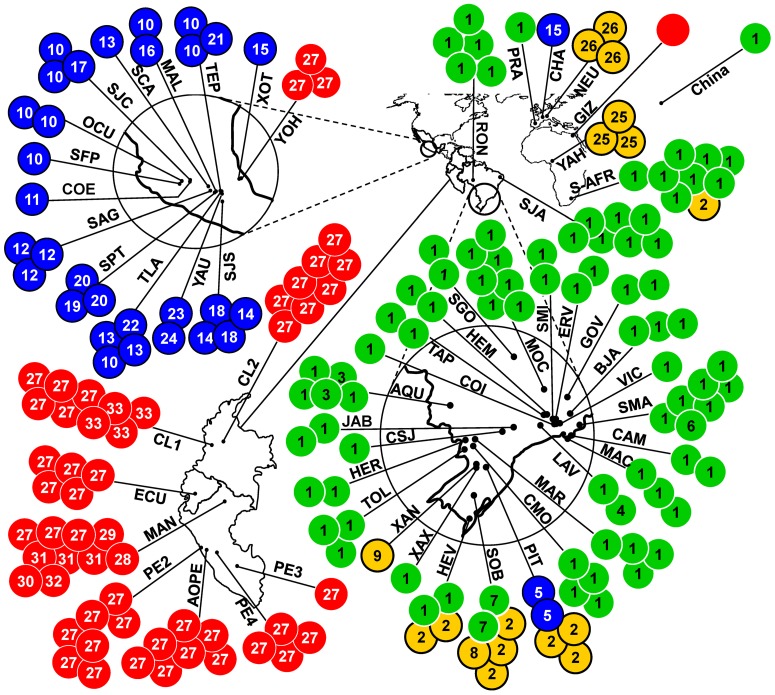
Geographic distributions of the 35 COI haplotypes of *A. obtectus*. Each circle represents the haplotypes found in a given population; a number within a circle denotes the COI haplotypes found in that population. Codes are as given in Table S1 in File S1; China, GenBank Accession FJ465153; for the remaining populations, see Alvarez et al. 2004 and 2005. Major mitochondrial lineages have been color-coded for reference purposes.

Located at the center of the COI network, Mitogroup I (Figure 2, shown in blue) contained 10 members that were organized around a high-frequency haplotype (haplotype 10). In addition, Mitogroup I included five highly divergent members (haplotypes 16, 18, 20, 22, and 23) that we had shown to contain an elevated number of substitutions and to have come from specimens that were sampled from wild populations of *P. vulgaris*. Mitogroup I was typically found in Mesoamerica (Figure 4, shown in blue), with haplotype 5 (found in the Brazilian PIT population) and haplotype 15 (found in the Swiss CHA population) being the only exceptions.

Haplotype 27 connected Mitogroup II (Figure 2, shown in red) to Mitogroup I through four intermediate missing haplotypes. Mitogroup II was found predominantly in the Andes. Nevertheless, three Mesoamerican specimens sampled at population YOH exhibited the most frequent Andean haplotype (haplotype 27). Judging from the partial sequence that we had available (GenBank accession number AY947519), a specimen collected in Giza, Egypt was of Andean origin (Figure 4).

Haplotypes found in Brazil and the Old World were organized into two closely related mitogroups; the first was clustered around the high-frequency haplotype 1, referred to as Mitogroup III (Figure 2, shown in green), and the second around haplotype 2, referred to as Mitogroup IV (Figure 2, shown in yellow). Two mutation steps distinguished haplotype 1 from haplotype 5, and connected Mitogroup III to Mitogroup I. Mitogroup IV was connected to the network via haplotype 2, which was located two mutation steps (both synonymous substitutions) away from haplotype 5. Whereas Mitogroup I and Mitogroup II each occupied a disjunct range in Mesoamerica and in the Andes, respectively, Mitogroup III and Mitogroup IV occurred mostly in sympatry throughout Brazil, Europe, and Africa (Figure 4). In Brazil, Mitogroup III seemed to have a widespread distribution, whereas Mitogroup IV appeared to be restricted to certain sites in the south of the country. The single specimen from China (GenBank accession number FJ465153, Figure 4) displayed haplotype 1, the most widespread haplotype of Mitogroup III.

Regression analyses confirmed that neither the number of haplotypes (*P = *0.84) nor nucleotide diversity (*P = *0.19) was associated significantly with sample size; this suggested that our sampling strategy did not favor a given geographic region to the detriment of the others.

### Inferences about Demographic History

Tests of selective neutrality and measures of nucleotide diversity suggested that populations of *A. obtectus* of distinct geographic origins had followed relatively distinct evolutionary paths (Table 1). The group ‘Mesoamerica’, with 30 sequences and 14 haplotypes, contained the highest haplotype diversity (0.894) and nucleotide diversity (0.0054) among all of the groups. Also remarkable was the fact that nucleotide divergence within ‘Mesoamerica’ was the highest amongst all of the groups, with differences between pairs of haplotypes reaching 3.95 nucleotides on average. In contrast, ‘Brazil1’ comprised 78 sequences but only six haplotypes; it showed the lowest haplotype diversity (0.195) and nucleotide diversity (0.0004) in our dataset. Moreover, the number of nucleotide differences among haplotypes of ‘Brazil1’ was very low (0.25 nucleotides on average) compared with those in the remaining groups. Tests of selective neutrality recovered significant, negative values for Tajima’s D (*P*<0.10) and Fu’s Fs (*P*<0.10) for all groups, with ‘Old World’ being the only exception (Table 1). Such significant negative values, particularly when considering the full dataset S6, indicated an excess of low-frequency polymorphisms and are consistent with either population expansion or purifying selection. ‘Old World’ was the only group that showed nonsignificant, positive values for both Tajima’s D and Fu’s Fs neutrality tests, which supported the null hypothesis of a neutrally evolving population.

**Table 1:** Measures of nucleotide diversities and neutrality test statistics for the cytochrome c oxidase subunit I (COI) of *Acanthoscelides obtectus.*

Groups	Sample size (numberof haplotypes - H)	Haplotype diversity (H_d_)	Nucleotide diversity (pi)	Average numberof nucleotidedifferences (k)	Number ofvariable sites (S)	Tajima’s D(*P*-value)	Fu’s Fs(*P*-value)	tau (τ) with 95% CI
Full (dataset S6)	187 (33)	0.768	0.0049	3.57	49	–1.72 (0.01)	–13.95(0.00)	4.5 (2.22–6.90)
Andes	51 (7)	0.381	0.0008	0.60	7	–1.61 (0.03)	–3.87 (0.01)	0.59 (0.0–2.10)
Mexico	30 (14)	0.894	0.0054	3.95	31	–1.79 (0.02)	–3.70 (0.06)	0.65 (0.57–11.04)
Old World	18 (5)	0.667	0.0045	3.33	9	0.97 (0.86)	2.03 (0.86)	6.2 (2.48–11.26)
Brazil	88 (9)	0.361	0.0014	1.06	10	–1.20 (0.11)	–2.88 (0.10)	4.0 (0.0–9.81)
Brazil1	78 (6)	0.195	0.0004	0.25	6	–1.85 (0.00)	–5.33 (0.00)	0.71 (0.0–2.37)
Brazil2	10 (3)	0.378	0.0006	0.40	2	–1.40 (0.10)	–1.16 (0.05)	0.48 (0.00–1.65)

**Figure 5:**
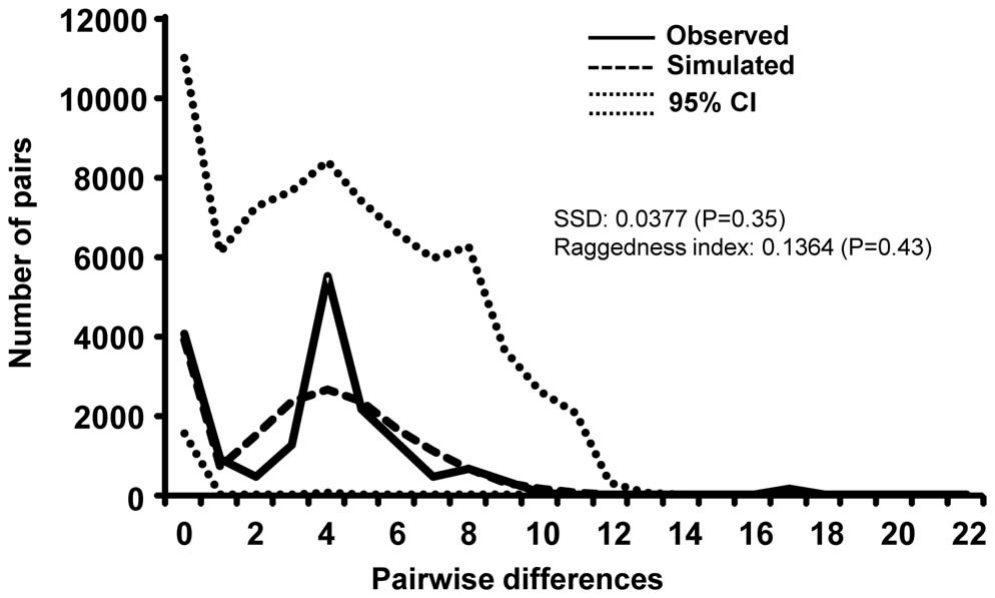
Mismatch distributions for COI haplotypes of *A. obtectus*. Observed and simulated theoretical mismatch distributions, with the 95% confidence interval, were obtained using a spatial expansion model. The raggedness index and the sum of square deviations (SSD), with their associated *P*-values, are indicated.

Estimates of τ (Table 1) suggested that the origin of ‘Mesoamerica’ (τ = 0.65) predated both ‘Andes’ (τ = 0.59) and ‘Brazil2’ (τ = 0.48). Given that the 95% confidence interval of ‘Mesoamerica’ (95% CI = 0.57 to 11.04) was much wider than that of ‘Brazil1’ (τ = 0.71; 95% CI = 0.0 to 2.37), it is plausible to think that the establishment of ‘Mesoamerica’ also preceded that of ‘Brazil1’. The high values of τ for both ‘Old World’ (τ = 6.2) and ‘Brazil’ (τ = 4.0) were difficult to interpret; at first examination, they were suggestive of those two groups being of ancient origin and having been founded well before ‘Mesoamerica’. When interpreting these high values of τ, one should take into consideration that both ‘Old World’ and ‘Brazil’ resulted from admixture of the divergent Mitogroup III and Mitogroup IV. Likewise, ‘Brazil’ and ‘Old World’ seem to display high values of other diversity estimates (Hd, pi, k, and S). Most likely, such characteristics that were suggestive of ‘Brazil’ and ‘Old World’ being of ancient origin and harboring high levels of genetic diversity were, in reality, the result of biased estimates; both groups contained differentiated mitogroups, which very likely inflated the measures of diversity when they were pooled together for those analyses. Mismatch distribution yielded a nonsignificant SSD (*P* = 0.35), which suggested that we cannot reject the null hypothesis of recent population expansion for *A. obtectus* (Figure 5). Although the mismatch yielded a distribution that seemed ragged, the nonsignificant raggedness index (*P* = 0.43) indicated a good fit of the data to the spatial expansion model. The mode of the distribution, which was centered at 4.5 pairwise differences, corresponded to τ that we had obtained for the full dataset S6 (Table 1).

### Estimated Date of Divergence

The BEAST analyses provided estimates for the time of divergence among lineages of *A. obtectus* (Table 2). Depending upon the calibration choice, age estimates for the tMRCA of ‘Mesoamerica’ ranged from very recent (1,552 yBP for hypothesis 500) to a time that predated agriculture (about 50,000 yBP for both hypotheses 8000a and 8000b). The analyses also suggested that *A. obtectus* arrived in ‘Brazil+Old World’ from 500 to about 17,000 yBP, which is earlier than it had dispersed into the ‘Andes’ (from 246 to 8,000 yBP). Analyses carried out at the mitogroup level also supported a scenario in which *A. obtectus* reached ‘Brazil’ (about 2,000 to 15,000 yBP, for Mitogroup III) before reaching the ‘Andes’ (about 1,000 to 8,000 yBP, for Mitogroup II). However, the latter analyses were more informative because they indicated that Mitogroup III diversified further and gave rise to Mitogroup IV in ‘Brazil’ (about 750 to 5,300 yBP) shortly after Mitogroup II reached the ‘Andes’ (about 1,000 to 8,000 yBP).

**Table 2:** Bayesian estimation of divergence times among lineages of *Acanthoscelides obtectus* based on cytochrome c oxidase subunit I (COI). Posterior means and the associated 95% highest probability density (in parentheses) for the age of the most recent common ancestor (tMRCA) are given in years. Calibration had the following priors: tMRCA for ‘Brazil+Old World’ (hypothesis 500) was placed at 500 years before the present (BP); tMRCAs of ‘Mesoamerica’ (hypothesis 7000a) and Mitogroup I (hypothesis 7000b) were placed at 7,000 BP; tMRCAs of ‘Andes’ (hypothesis 8000a) and Mitogroup II (hypothesis 8000b) were set to 8,000 BP (see Material and Methods for calibration details).

Tested groups	Hypotheses		
Geographicregions	500	7000a	8000a
Mesoamerica	1,552(497–2,765)	7,000(6,998–7,002)	51,398(16,865–96,013)
Andes	246 (66–471)	1,115(349–2,066)	8,000(7,998–8,002)
Brazil+OldWorld	500 (498–502)	2,263(814–4,159)	16,657(4,537–32,419)
Mitogroups		7000b	8000b
I		7,000(6,998–7,002)	49,659(15,246–91,825)
II		1,107(351–2,038)	8,000(7,998–8,002)
III		2,110(685–3,975)	15,122(3,854–30,375)
IV		741(176–1,447)	5,267(964–11,144)

## Discussion

### Congruent Lineages in *A. obtectus* and *P. vulgaris*

Our findings suggest a pattern of phylogeographic congruence, in which the evolutionary history of a seed-predator insect (*A. obtectus*) paralleled that of its primary host (*P. vulgaris*). The existence of two highly differentiated gene pools of *P. vulgaris* indicates that gene flow between the two geographic regions was prevented for a considerable period of time [4]–[6]. Our phylogenetic and phylogeographic analyses were concordant insofar as they showed that each of the two major centers of genetic diversity of *P. vulgaris–*Mesoamerica and the Andes*–*harbor a distinct genealogical lineage of *A. obtectus*. The two lineages of *A. obtectus* were highly differentiated and it is most likely that they evolved under genetic isolation. Thus, our results support a scenario in which the Mesoamerican and Andean lineages of *A. obtectus* diverged in allopatry, with selection and genetic drift in the absence of the homogenizing effects of gene flow as the likely cause of the divergence. Brazil, a secondary center of diversification of *P. vulgaris* [34], harbors neither the Mesoamerican nor the Andean lineages of *A. obtectus*; instead, Brazil harbors two additional, closely related lineages, both of which have the Mesoamerican lineage as their ancestor.

### A Mesoamerican Origin for *A. obtectus*

Both the 16s rRNA gene and the COI networks shed light on temporal relationships among the genealogical lineages. Haplotypes of the Mesoamerican lineage were located in the inner part of the network, as predicted by the coalescent theory [35] for lineages of ancestral status. The remaining lineages occupied tip positions, so they displayed characteristics of derived lineages [35]. Network configuration based on the slowest evolving 12s rRNA gene was an exception, due to the fact that the gene contained only two closely related haplotypes. Consistent with the lineage arrangements of the 16s rRNA and COI networks, the concatenation of three mitochondrial genes yielded a Bayesian phylogeny with a topology that places the Andean and Brazilian clades nested within the Mesoamerican clade.

Comparative measures of nucleotide diversity provided further evidence for the ancient origin of the Mesoamerican lineage. Moreover, we found that Mesoamerican bruchids contained numerous, naturally occurring polymorphisms that gave rise to highly divergent COI sequences, at both the DNA and protein levels. A plausible explanation for these highly divergent COI sequences is that Mesoamerica harbors wild populations that depart from both Andean and Brazilian gene pools of *A. obtectus*, the latter two being most commonly associated with domesticated *P. vulgaris*.

Our estimates of the time of divergence provide additional evidence for the early emergence of the Mesoamerican lineage of *A. obtectus*. Regardless of grouping alternatives (based on either geographic origin or mitogroups), the analyses were congruent in attributing older dates to the Mesoamerican lineage.

The origin of *P. vulgaris* may also help to clarify the origin of *A. obtectus*. Compelling evidence indicates Central Mexico, but not the Andes, as a more plausible origin of *P. vulgaris*; the independent gene pools of *P. vulgaris* currently found in South America arose from distinct dispersal events that began in Central Mexico [6]. Life-history traits of *A. obtectus* (see [9]) favor codispersal with the host as an important strategy for achieving expansion of its range over long distances. In light of those results, we concluded that *A. obtectus* from Mesoamerica could have taken advantage of at least one of those dispersal events to pursue the host and, as a consequence, have been successful in establishing a new range in the Andes.

The proposed Mesoamerican origin of *A. obtectus* is at odds with an hypothesis proposed in a previous study, such that the species left the Andes and arrived at the Mexican altiplano at 705 (±175) yBP [11]. This lack of agreement may be due in part to differences in the datasets and statistical analyses that were used in the two studies. Our dataset was larger and contained samples from a broader geographic area than the dataset that was available previously [35]. We used a series of distinct, but complementary, statistical analyses, all of which support the Mesoamerican origin of *A. obtectus* as plausible.

### Pre-Columbian Range Expansions

We did not calibrate our BEAST analyses with information based on generalized molecular clocks for COI. Such clocks have been used to unravel the phylogeography of insects when information from fossil records or geological events is missing [36]–[37]; they seem to be useful when the timescale is about 1–2 million yBP or older [38]. In light of this, the use of generalized molecular clocks to draw inferences about the late evolutionary history of *A. obtectus* would be inappropriate. In the absence of archeological or historical events directly related to *A. obtectus*, we decided to calibrate our analyses with independent data from two of the earliest archeological records of the host species (*P. vulgaris*) and interpret the results within the context of pre- and post-Columbian timeframes.

Setting the tMRCA of the Andean lineage to 8,000 yBP, as is posited by hypotheses 8000a and 8000b, pushes the tMRCA of the Mesoamerican lineage back to about 51,000 yBP (with an upper limit of about 96,000 yBP). These hypotheses place the origin of the Mesoamerican lineage well before the human occupation of the Americas [39], and suggest that naturally occurring, long-range dispersal events triggered expansions in the range of *A. obtectus*.

Hypotheses 8000a and 8000b would seem to overestimate the tMRCA of the Brazilian lineages, thus placing their veracity in doubt. Judging from the posterior means for hypothesis 8000a (16,657 yBP*–*the group ‘Brazil+Old World’) and hypothesis 8000b (15,122 yBP*–*for mitogroup III), the two most plausible dispersal mechanisms that could have brought *A. obtectus* to Eastern South America (human-mediated dispersals and codispersal with the host) were most likely not operational at about 15,000 yBP. At that time, *A. obtectus* could rely on neither human-mediated dispersal of infested seeds (because peopling of the Americas was just beginning) [38], nor on naturally occurring, codispersal events (because the known range of distribution of wild *Phaseolus* was restricted to the Andes, with wild populations scattered from Colombia to northern Argentina) [3]. However, these apparently conflicting consequences of hypotheses 8000a and 8000b can be reconciled. One should bear in mind that our analyses assume that lineages become differentiated *in situ*. Therefore, age estimates for the tMRCA of Brazilian lineages would be overestimated if the Brazilian lineages became differentiated outside Eastern South America. If they became differentiated somewhere else, they could have reached the region at a later time via human intervention, for example, activities associated with the early cultivation of *Phaseolus*.

**Figure 6:**
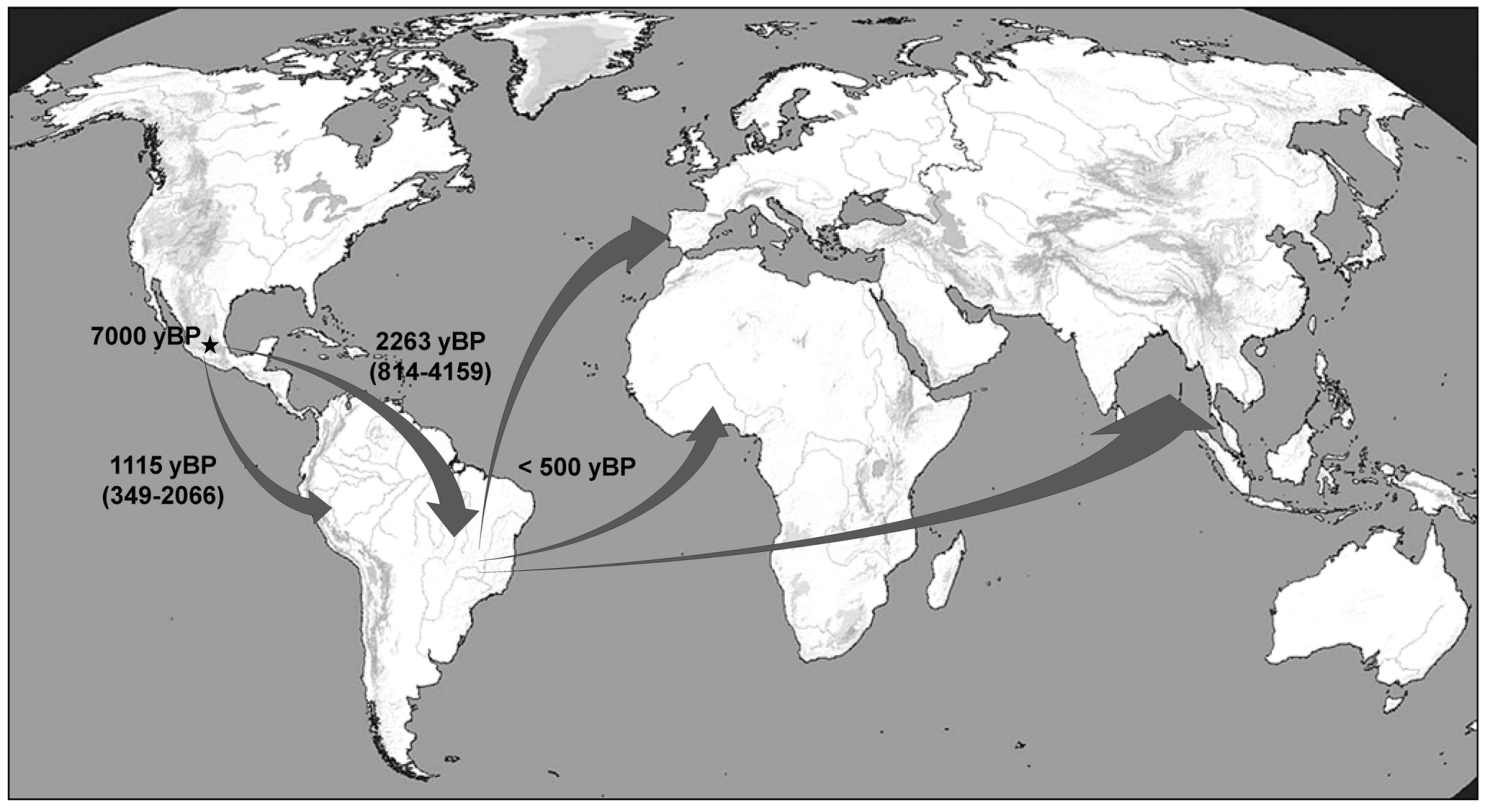
Hypothetical routes for major dispersal events and estimated times of divergence among lineages of *A. obtectus*. The star suggests the origin of migrations according to hypothesis 7000a (see text for details). Arrows indicate timing and directions of long-range dispersal events. Estimated time of divergence is given in years before the present (yBP) and the 95% highest probability density is shown in parentheses.

Setting the tMRCA of the Mesoamerican lineage to 7,000 yBP (hypotheses 7000a and 7000b) results in tMRCAs dating from about 2,100 and 740 yBP (for the oldest and youngest Brazilian lineages, respectively) to about 1,100 yBP (for the Andean lineage). Although these three age estimates seem very recent, they are within the time window compatible with human-mediated dispersal via agriculture. Moreover, they suggest that *A. obtectus* arrived in Eastern South America earlier than it did in the Andes. We depicted hypothetical dispersal routes and estimated times of divergence among genealogical lineages of *A. obtectus* according to hypothesis 7000a (Figure 6), with the caveat that the time of divergence may be earlier than the estimates that the use of this hypothesis suggested.

The absence of the Andean lineage of *A. obtectus* in Eastern South America is congruent with archeological evidence of *P. vulgaris* from that region. Lapa do Boquete, a cave located in the Eastern highlands of Central Brazil, shows traces of human occupation since 10,000 yBP [40]; the cave yielded archeological remains of *P. vulgaris* that were genetically closer to gene pools from Northern South America and Mexico than to Andean gene pools [41]. Although of recent origin (about 300 yBP), archeological remains of the Lapa do Boquete cave can be considered as part of the pre-Columbian era because contacts between native Brazilians and European settlers in the region did not begin until 200 yBP [41]. A likely route that connected Mesoamerica to Eastern South America through the Caribbean has been proposed, using biochemical data (sharing of phaseolin types) as a basis [42]. Taken together, the late evolutionary histories of *A. obtectus* and *P. vulgaris* suggest that, in pre-Columbian times, trade connections existed between the indigenous peoples of Eastern South America and those of Northern South America and Mesoamerica. Nonetheless, the trading routes for *P. vulgaris* did not include the Andes.

### Post-Columbian Range Expansions

Restricting the tMRCA of the Brazilian lineages to the post-Columbian era (hypothesis 500) results in very recent ages for both the tMRCA of the Mesoamerican lineage (about 1500 yBP, with an upper limit of about 2700 yBP) and the tMRCA of the Andean lineage (about 250 yBP). If hypothesis 500 holds true, the Mesoamerican lineage remained confined to Central Mexico and surrounding areas during the domestication of *P. vulgaris*; in addition, it escaped unintended human-mediated dispersal via seed exchanges that followed the domestication of *P. vulgaris*. Moreover, the Mesoamerican lineage had to undergo genetic differentiation and reach new territories rapidly. Such an implausible scenario led us to regard hypothesis 500 as the least parsimonious of our three alternative hypotheses to explain the origin and timing of major range expansions of *A. obtectus*.

Historical accounts mention that *P. vulgaris* was introduced into several European countries soon after the discovery of the New World and that it spread to African countries from there [2]; the most robust description of the first occurrence of *A. obtectus* in Europe dates from 1879 [14]. Given the fact that extant European germplasm of *P. vulgaris* received a larger contribution from the Andean gene pool, with the Mesoamerican gene pool contributing to a lesser extent [43], our finding that Brazilian lineages prevail in the Old World was unexpected. We conclude that there was an historical asymmetry regarding the geographic origin of the seed source and unintended, human-mediated codispersal events.

### Phytosanitary Implications

Within an agricultural context, insect pests of stored products are subject to selective pressures that are somewhat distinct from those they would experience in a natural environment. Not surprisingly, geographically distinct populations of insect pests of stored products possess a broad variety of biological traits [44]–[46]. The unintended introduction of novel genotypes may contribute to increasing the fitness of a population that is already established, because newly introduced specimens may carry traits that confer resistance to the host's defense mechanisms or to insecticides. Thus, newly established populations should be watched closely for resistance to insecticides. As an example, consider that the use of the fumigant insecticide phosphine is one of the main methods currently in use to control *A. obtectus* in Brazil. Given that our results suggest a common origin and recent dispersal routes for lineages prevailing in Brazil, Europe, Africa, and Asia, we recommend that newly established populations in those regions be watched closely for phosphine resistance.

Given that the cosmopolitan distribution of *A. obtectus* seems to be due to the recent expansion of the Brazilian lineages (with a few exceptions in France and Egypt), phytosanitary measures to prevent both the Mesoamerican lineage and the Andean lineage from becoming widespread may be worthwhile, because such prevention would eliminate the possibility of accidental admixture of distinct gene pools and the rise of new gene combinations that give rise to adaptations that can benefit populations in new territories.

Control strategies have a greater chance of success when we seek information in regions where the host, the pest, and its natural enemies coevolved for a longer period of time [47]. In light of this, the strong indication of a Mesoamerican origin for *A. obtectus* suggests that breeding programmes may benefit from searching for both resistance genes and natural enemies against this threat in Mesoamerica. Our analyses also suggest that we should not overlook Eastern South America as a potential harbor for natural enemies, given that *A. obtectus* has been present in this region for longer than had previously been thought.

## Supporting Information

File S1 **Supporting Information File containing Table S1 and Datasets S1–S6.** Table S1. Sampling sites of *Acanthoscelides obtectus*. Dataset S1– DNA alignment file for the 12s rRNA gene (in Nexus format). Dataset S2– DNA alignment file for the 16s rRNA gene (in Nexus format). Dataset S3– DNA alignment file for the COI gene (in Nexus format). Dataset S4– DNA alignment file for the concatenation of the 12s rRNA, 16s RNA, and COI genes (in Nexus format). Dataset S5– modified DNA alignment file for the COI gene (in Nexus format). Dataset S6– modified DNA alignment file for the COI gene (in Nexus format).
(PDF)
