# Supplementary material for: Mesoamerican Origin and Pre- and Post-Columbian Expansions of the Ranges of Acanthoscelides obtectus Say, a Cosmopolitan Insect Pest of the Common Bean
Source: PLoS One. 2013 Jul 31;8(7):e70039. doi: 10.1371/journal.pone.0070039 (PMC3729460; doi:10.1371/journal.pone.0070039)
Supplement: File S1 — Supporting Information File containing Table S1 and Datasets S1–S6. Table S1. Sampling sites of Acanthoscelides obtectus. Dataset S1– DNA alignment file for the 12s rRNA gene (in Nexus format). Dataset S2– DNA alignment file for the 16s rRNA gene (in Nexus format). Dataset S3– DNA alignment file for the COI gene (in Nexus format). Dataset S4– DNA alignment file for the concatenation of the 12s rRNA, 16s RNA, and COI genes (in Nexus format). Dataset S5– modified DNA alignment file for the COI gene (in Nexus format). Dataset S6– modified DNA alignment file for the COI gene (in Nexus format). (PDF) [file pone.0070039.s001.pdf]

Table S1

Sampling sites of *Acanthoscelides obtectus*. Sites where the bean bruchid was sampled for this study are listed, together with the abbreviation code, sample sizes for the COI, rRNA 16S, and rRNA 12S genes, and the geographical coordinates.

Site	Code	Sample size			Latitude (S)	Longitude (W)
		COI	16S	12S		
Aquidauana, MS	AQU	5	2		20°28’28.59’’	55°47’14.99’’
Bom Jesus do Amparo, MG	BJA	3	2	1	19º42’01.84’’	43º28’41.16’’
Campos dos Goytacazes, RJ	CAM	2			21°45’08.20’’	41°20’00.42’’
Campo Mourão, PR	CMO	4	2		24°08’20.04’’	52°36’51.60’’
Coimbra, MG	COI	1			20°50’58.84’’	42°47’27.04’’
Córrego São João, SP	CSJ	1	1		21°08’42.91’’	51°05’27.09’’
Ervália- MG	ERV	2			20°50’35.05’’	42°39’32.19’’
Governador Valadares, MG	GOV	2			18°40’17.30’’	42°20’41.42’’
Hematita, MG	HEM	2			19°31’00.00’’	43°06’17.14’’
Herculândia, PR	HER	2			23°16’11.49’’	53°24’04.54’’
Herval, RS	HEV	4	2	1	32°00’46.44’’	53°24’11.24’’
Jaboticabal, SP	JAB	2			21°15’19.40’’	48°19’20.70’’
Lavras, MG	LAV	2	1		21°14’44.65’’	44°59’59.20’’
Macaé, RJ	MAC	3	3		22°35’58.78’’	42°20’00.42’’
Maringá, PR	MAR	4	1		23°29’47.84	52°06’41.81’’
Montes Claros, MG	MOC	7	3	1	16°44’13.25’’	43°51’53.06’’
Piratuba, SC	PIT	5	3	3	27°24’29.08’’	51°45’07.43’’
Cacoal, RO	RON	5	4	1	11°24’31.37’’	61° 26’38.20”
Sto. Antônio de Goiás, GO	SGO	2			16º29’11.36’’	49°18’46.37’’
São João, PE	SJA	7	5		7°37’59.92’’	37°38’59.80’’
Santa Maria, ES	SMA	7	3		21°11’07.18’’	41°06’17.14’’
São Miguel do Anta, MG	SMI	2			20º42’51.40’’	42°45’14.52’’
Sobradinho, RS	SOB	6	3	1	29°25’21.92’’	53°01’57.12’’
Taparuba, MG	TAP	2			19°46’00.74’’	41°37’21.42’’
Toledo, PR	TOL	3	2	1	24°43’11.50’’	53°44’35.81’’
Viçosa, MG	VIC	1			20º40’20.22’’	42°59’40.72’’
Xanxerê, SC	XAN	1			26°53’09.07’’	52°24’53.16’’
Xaxim, SC	XAX	1			26°56’55.98’’	52°31’38.23’’
Pietermaritzburg, South Africa	AFR	9	7	2	29°36’12.63’’	30°22’46.62’’(E)
Argelia, Colombia	CL1	9	9	9	4°43’52’’(N)	76°7’0’’
Villa de Leyva, Colombia	CL2	8	2	7	5°35’43’’(N)	73°31’58’’
Cuenca, Ecuador	ECU	5	1	4	2°54’00’’	78°59’00’’
Marcará, Peru	AOPE	7	4	4	9°19’21’’	77°36’13’’
Chimbote, Peru	PE2	7	6	7	9°4’28’’	78°35’34’’
Pangoa, Peru	PE3	1			12°7’0’’	73°0’0’’
Huanuco, Peru	PE4	4			9°55’46’’	76°14’23’’
**Total**		**139**	**66**	**42**		

datasetS1

#NEXUS

begin data;
dimensions ntax=56 nchar=378;
format datatype=dna gap=- interleave=no;
matrix

afr19
CGGGCGATGTGTACATATTTTAGAGCTTTAATCATCTTAAAAAAATCTAAAAAATT
ACTCTCAAATCCAAATTCAAATTTTTTTAACAAAAATTATTTTAAAATAAATTTATT
GTAACCCATTTCTTCTTTATTATAAACTACACCTTGATCTGATTTATTTTTTAATAA
AAAATTTTAAATATTTATAATTCTATTAAAATATTTAAACTACGACGATATATAAAC
TAATAAATAAAGTGCATCTAATCGTGGACTATCGATTATAGAACAGGTTCCTCTG
AAAAGACTAAAATACCGCCAAATTTTTTAATTTTCAAGAACATAACTAATACTAAT
TAAGTAAAAAAATTTACATTTTTAATAATAGGGTATCTAA
afr9
CGGGCGATGTGTACATATTTTAGAGCTTTAATCATCTTAAAAAAATCTAAAAAATT
ACTCTCAAATCCAAATTCAAATTTTTTTAACAAAAATTATTTTAAAATAAATTTATT
GTAACCCATTTCTTCTTTATTATAAACTACACCTTGATCTGATTTATTTTTTAATAA
AAAATTTTAAATATTTATAATTCTATTAAAATATTTAAACTACGACGATATATAAAC
TAATAAATAAAGTGCATCTAATCGTGGACTATCGATTATAGAACAGGTTCCTCTG
AAAAGACTAAAATACCGCCAAATTTTTTAATTTTCAAGAACATAACTAATACTAAT
TAAGTAAAAAAATTTACATTTTTAATAATAGGGTATCTAA
AOPE_12
CGGGCGATGTGTACATATTTTAGAGCTTTAATCATCTTAAAAAAATCTAAAAAATT
ACTCTCAAATCCAAATTCAAATTTTTTTAACAAAAATTATTTTCAAATAAATTTATT
GTAACCCATTTCTTCTTTATTATAAACTACACCTTGATCTGATTTATTTTTTAATAA
AAAATTTTAAATATTTATAATTCTATTAAAATATTTAAACTACGACGATATATAAAC
TAATAAATAAAGTGCATCTAATCGTGGACTATCGATTATAGAACAGGTTCCTCTG
AAAAGACTAAAATACCGCCAAATTTTTTAATTTTCAAGAACATAACTAATACTAAT
TAAGTAAAAAAATTTACATTTTTAATAATAGGGTATCTAA
AOPE_13
CGGGCGATGTGTACATATTTTAGAGCTTTAATCATCTTAAAAAAATCTAAAAAATT
ACTCTCAAATCCAAATTCAAATTTTTTTAACAAAAATTATTTTCAAATAAATTTATT
GTAACCCATTTCTTCTTTATTATAAACTACACCTTGATCTGATTTATTTTTTAATAA
AAAATTTTAAATATTTATAATTCTATTAAAATATTTAAACTACGACGATATATAAAC
TAATAAATAAAGTGCATCTAATCGTGGACTATCGATTATAGAACAGGTTCCTCTG
AAAAGACTAAAATACCGCCAAATTTTTTAATTTTCAAGAACATAACTAATACTAAT
TAAGTAAAAAAATTTACATTTTTAATAATAGGGTATCTAA
AOPE_14
CGGGCGATGTGTACATATTTTAGAGCTTTAATCATCTTAAAAAAATCTAAAAAATT
ACTCTCAAATCCAAATTCAAATTTTTTTAACAAAAATTATTTTCAAATAAATTTATT
GTAACCCATTTCTTCTTTATTATAAACTACACCTTGATCTGATTTATTTTTTAATAA
AAAATTTTAAATATTTATAATTCTATTAAAATATTTAAACTACGACGATATATAAAC
TAATAAATAAAGTGCATCTAATCGTGGACTATCGATTATAGAACAGGTTCCTCTG
AAAAGACTAAAATACCGCCAAATTTTTTAATTTTCAAGAACATAACTAATACTAAT
TAAGTAAAAAAATTTACATTTTTAATAATAGGGTATCTAA

AOPE_4
CGGGCGATGTGTACATATTTTAGAGCTTTAATCATCTTAAAAAAATCTAAAAAATT
ACTCTCAAATCCAAATTCAAATTTTTTTAACAAAAATTATTTTCAAATAAATTTATT
GTAACCCATTTCTTCTTTATTATAAACTACACCTTGATCTGATTTATTTTTTAATAA
AAAATTTTAAATATTTATAATTCTATTAAAATATTTAAACTACGACGATATATAAAC
TAATAAATAAAGTGCATCTAATCGTGGACTATCGATTATAGAACAGGTTCCTCTG
AAAAGACTAAAATACCGCCAAATTTTTTAATTTTCAAGAACATAACTAATACTAAT
TAAGTAAAAAAATTTACATTTTTAATAATAGGGTATCTAA
bja1
CGGGCGATGTGTACATATTTTAGAGCTTTAATCATCTTAAAAAAATCTAAAAAATT
ACTCTCAAATCCAAATTCAAATTTTTTTAACAAAAATTATTTTAAAATAAATTTATT
GTAACCCATTTCTTCTTTATTATAAACTACACCTTGATCTGATTTATTTTTTAATAA
AAAATTTTAAATATTTATAATTCTATTAAAATATTTAAACTACGACGATATATAAAC
TAATAAATAAAGTGCATCTAATCGTGGACTATCGATTATAGAACAGGTTCCTCTG
AAAAGACTAAAATACCGCCAAATTTTTTAATTTTCAAGAACATAACTAATACTAAT
TAAGTAAAAAAATTTACATTTTTAATAATAGGGTATCTAA
CAM_YAH
CGGGCGATGTGTACATATTTTAGAGCTTTAATCATCTTAAAAAAATCTAAAAAATT
ACTCTCAAATCCAAATTCAAATTTTTTTAACAAAAATTATTTTAAAATAAATTTATT
GTAACCCATTTCTTCTTTATTATAAACTACACCTTGATCTGATTTATTTTTTAATAA
AAAATTTTAAATATTTATAATTCTATTAAAATATTTAAACTACGACGATATATAAAC
TAATAAATAAAGTGCATCTAATCGTGGACTATCGATTATAGAACAGGTTCCTCTG
AAAAGACTAAAATACCGCCAAATTTTTTAATTTTCAAGAACATAACTAATACTAAT
TAAGTAAAAAAATTTACATTTTTAATAATAGGGTATCTAA
CL1_1
CGGGCGATGTGTACATATTTTAGAGCTTTAATCATCTTAAAAAAATCTAAAAAATT
ACTCTCAAATCCAAATTCAAATTTTTTTAACAAAAATTATTTTCAAATAAATTTATT
GTAACCCATTTCTTCTTTATTATAAACTACACCTTGATCTGATTTATTTTTTAATAA
AAAATTTTAAATATTTATAATTCTATTAAAATATTTAAACTACGACGATATATAAAC
TAATAAATAAAGTGCATCTAATCGTGGACTATCGATTATAGAACAGGTTCCTCTG
AAAAGACTAAAATACCGCCAAATTTTTTAATTTTCAAGAACATAACTAATACTAAT
TAAGTAAAAAAATTTACATTTTTAATAATAGGGTATCTAA
CL1_2
CGGGCGATGTGTACATATTTTAGAGCTTTAATCATCTTAAAAAAATCTAAAAAATT
ACTCTCAAATCCAAATTCAAATTTTTTTAACAAAAATTATTTTCAAATAAATTTATT
GTAACCCATTTCTTCTTTATTATAAACTACACCTTGATCTGATTTATTTTTTAATAA
AAAATTTTAAATATTTATAATTCTATTAAAATATTTAAACTACGACGATATATAAAC
TAATAAATAAAGTGCATCTAATCGTGGACTATCGATTATAGAACAGGTTCCTCTG
AAAAGACTAAAATACCGCCAAATTTTTTAATTTTCAAGAACATAACTAATACTAAT
TAAGTAAAAAAATTTACATTTTTAATAATAGGGTATCTAA
CL1_3
CGGGCGATGTGTACATATTTTAGAGCTTTAATCATCTTAAAAAAATCTAAAAAATT
ACTCTCAAATCCAAATTCAAATTTTTTTAACAAAAATTATTTTCAAATAAATTTATT
GTAACCCATTTCTTCTTTATTATAAACTACACCTTGATCTGATTTATTTTTTAATAA
AAAATTTTAAATATTTATAATTCTATTAAAATATTTAAACTACGACGATATATAAAC
TAATAAATAAAGTGCATCTAATCGTGGACTATCGATTATAGAACAGGTTCCTCTG
AAAAGACTAAAATACCGCCAAATTTTTTAATTTTCAAGAACATAACTAATACTAAT
TAAGTAAAAAAATTTACATTTTTAATAATAGGGTATCTAA
CL1_4
CGGGCGATGTGTACATATTTTAGAGCTTTAATCATCTTAAAAAAATCTAAAAAATT

ACTCTCAAATCCAAATTCAAATTTTTTTAACAAAAATTATTTTCAAATAAATTTATT
GTAACCCATTTCTTCTTTATTATAAACTACACCTTGATCTGATTTATTTTTTAATAA
AAAATTTTAAATATTTATAATTCTATTAAAATATTTAAACTACGACGATATATAAAC
TAATAAATAAAGTGCATCTAATCGTGGACTATCGATTATAGAACAGGTTCCTCTG
AAAAGACTAAAATACCGCCAAATTTTTTAATTTTCAAGAACATAACTAATACTAAT
TAAGTAAAAAAATTTACATTTTTAATAATAGGGTATCTAA
CL1_5
CGGGCGATGTGTACATATTTTAGAGCTTTAATCATCTTAAAAAAATCTAAAAAATT
ACTCTCAAATCCAAATTCAAATTTTTTTAACAAAAATTATTTTCAAATAAATTTATT
GTAACCCATTTCTTCTTTATTATAAACTACACCTTGATCTGATTTATTTTTTAATAA
AAAATTTTAAATATTTATAATTCTATTAAAATATTTAAACTACGACGATATATAAAC
TAATAAATAAAGTGCATCTAATCGTGGACTATCGATTATAGAACAGGTTCCTCTG
AAAAGACTAAAATACCGCCAAATTTTTTAATTTTCAAGAACATAACTAATACTAAT
TAAGTAAAAAAATTTACATTTTTAATAATAGGGTATCTAA
CL1_6
CGGGCGATGTGTACATATTTTAGAGCTTTAATCATCTTAAAAAAATCTAAAAAATT
ACTCTCAAATCCAAATTCAAATTTTTTTAACAAAAATTATTTTCAAATAAATTTATT
GTAACCCATTTCTTCTTTATTATAAACTACACCTTGATCTGATTTATTTTTTAATAA
AAAATTTTAAATATTTATAATTCTATTAAAATATTTAAACTACGACGATATATAAAC
TAATAAATAAAGTGCATCTAATCGTGGACTATCGATTATAGAACAGGTTCCTCTG
AAAAGACTAAAATACCGCCAAATTTTTTAATTTTCAAGAACATAACTAATACTAAT
TAAGTAAAAAAATTTACATTTTTAATAATAGGGTATCTAA
CL1_7
CGGGCGATGTGTACATATTTTAGAGCTTTAATCATCTTAAAAAAATCTAAAAAATT
ACTCTCAAATCCAAATTCAAATTTTTTTAACAAAAATTATTTTCAAATAAATTTATT
GTAACCCATTTCTTCTTTATTATAAACTACACCTTGATCTGATTTATTTTTTAATAA
AAAATTTTAAATATTTATAATTCTATTAAAATATTTAAACTACGACGATATATAAAC
TAATAAATAAAGTGCATCTAATCGTGGACTATCGATTATAGAACAGGTTCCTCTG
AAAAGACTAAAATACCGCCAAATTTTTTAATTTTCAAGAACATAACTAATACTAAT
TAAGTAAAAAAATTTACATTTTTAATAATAGGGTATCTAA
CL1_8
CGGGCGATGTGTACATATTTTAGAGCTTTAATCATCTTAAAAAAATCTAAAAAATT
ACTCTCAAATCCAAATTCAAATTTTTTTAACAAAAATTATTTTCAAATAAATTTATT
GTAACCCATTTCTTCTTTATTATAAACTACACCTTGATCTGATTTATTTTTTAATAA
AAAATTTTAAATATTTATAATTCTATTAAAATATTTAAACTACGACGATATATAAAC
TAATAAATAAAGTGCATCTAATCGTGGACTATCGATTATAGAACAGGTTCCTCTG
AAAAGACTAAAATACCGCCAAATTTTTTAATTTTCAAGAACATAACTAATACTAAT
TAAGTAAAAAAATTTACATTTTTAATAATAGGGTATCTAA
CL1_9
CGGGCGATGTGTACATATTTTAGAGCTTTAATCATCTTAAAAAAATCTAAAAAATT
ACTCTCAAATCCAAATTCAAATTTTTTTAACAAAAATTATTTTCAAATAAATTTATT
GTAACCCATTTCTTCTTTATTATAAACTACACCTTGATCTGATTTATTTTTTAATAA
AAAATTTTAAATATTTATAATTCTATTAAAATATTTAAACTACGACGATATATAAAC
TAATAAATAAAGTGCATCTAATCGTGGACTATCGATTATAGAACAGGTTCCTCTG
AAAAGACTAAAATACCGCCAAATTTTTTAATTTTCAAGAACATAACTAATACTAAT
TAAGTAAAAAAATTTACATTTTTAATAATAGGGTATCTAA
CL2_1
CGGGCGATGTGTACATATTTTAGAGCTTTAATCATCTTAAAAAAATCTAAAAAATT
ACTCTCAAATCCAAATTCAAATTTTTTTAACAAAAATTATTTTCAAATAAATTTATT
GTAACCCATTTCTTCTTTATTATAAACTACACCTTGATCTGATTTATTTTTTAATAA

AAAATTTTAAATATTTATAATTCTATTAAAATATTTAAACTACGACGATATATAAAC
TAATAAATAAAGTGCATCTAATCGTGGACTATCGATTATAGAACAGGTTCCTCTG
AAAAGACTAAAATACCGCCAAATTTTTTAATTTTCAAGAACATAACTAATACTAAT
TAAGTAAAAAAATTTACATTTTTAATAATAGGGTATCTAA
CL2_2
CGGGCGATGTGTACATATTTTAGAGCTTTAATCATCTTAAAAAAATCTAAAAAATT
ACTCTCAAATCCAAATTCAAATTTTTTTAACAAAAATTATTTTCAAATAAATTTATT
GTAACCCATTTCTTCTTTATTATAAACTACACCTTGATCTGATTTATTTTTTAATAA
AAAATTTTAAATATTTATAATTCTATTAAAATATTTAAACTACGACGATATATAAAC
TAATAAATAAAGTGCATCTAATCGTGGACTATCGATTATAGAACAGGTTCCTCTG
AAAAGACTAAAATACCGCCAAATTTTTTAATTTTCAAGAACATAACTAATACTAAT
TAAGTAAAAAAATTTACATTTTTAATAATAGGGTATCTAA
EQU_5
CGGGCGATGTGTACATATTTTAGAGCTTTAATCATCTTAAAAAAATCTAAAAAATT
ACTCTCAAATCCAAATTCAAATTTTTTTAACAAAAATTATTTTCAAATAAATTTATT
GTAACCCATTTCTTCTTTATTATAAACTACACCTTGATCTGATTTATTTTTTAATAA
AAAATTTTAAATATTTATAATTCTATTAAAATATTTAAACTACGACGATATATAAAC
TAATAAATAAAGTGCATCTAATCGTGGACTATCGATTATAGAACAGGTTCCTCTG
AAAAGACTAAAATACCGCCAAATTTTTTAATTTTCAAGAACATAACTAATACTAAT
TAAGTAAAAAAATTTACATTTTTAATAATAGGGTATCTAA
FRA_CHA_5
CGGGCGATGTGTACATATTTTAGAGCTTTAATCATCTTAAAAAAATCTAAAAAATT
ACTCTCAAATCCAAATTCAAATTTTTTTAACAAAAATTATTTTAAAATAAATTTATT
GTAACCCATTTCTTCTTTATTATAAACTACACCTTGATCTGATTTATTTTTTAATAA
AAAATTTTAAATATTTATAATTCTATTAAAATATTTAAACTACGACGATATATAAAC
TAATAAATAAAGTGCATCTAATCGTGGACTATCGATTATAGAACAGGTTCCTCTG
AAAAGACTAAAATACCGCCAAATTTTTTAATTTTCAAGAACATAACTAATACTAAT
TAAGTAAAAAAATTTACATTTTTAATAATAGGGTATCTAA
hev1
CGGGCGATGTGTACATATTTTAGAGCTTTAATCATCTTAAAAAAATCTAAAAAATT
ACTCTCAAATCCAAATTCAAATTTTTTTAACAAAAATTATTTTAAAATAAATTTATT
GTAACCCATTTCTTCTTTATTATAAACTACACCTTGATCTGATTTATTTTTTAATAA
AAAATTTTAAATATTTATAATTCTATTAAAATATTTAAACTACGACGATATATAAAC
TAATAAATAAAGTGCATCTAATCGTGGACTATCGATTATAGAACAGGTTCCTCTG
AAAAGACTAAAATACCGCCAAATTTTTTAATTTTCAAGAACATAACTAATACTAAT
TAAGTAAAAAAATTTACATTTTTAATAATAGGGTATCTAA
MEX_COE_2
CGGGCGATGTGTACATATTTTAGAGCTTTAATCATCTTAAAAAAATCTAAAAAATT
ACTCTCAAATCCAAATTCAAATTTTTTTAACAAAAATTATTTTCAAATAAATTTATT
GTAACCCATTTCTTCTTTATTATAAACTACACCTTGATCTGATTTATTTTTTAATAA
AAAATTTTAAATATTTATAATTCTATTAAAATATTTAAACTACGACGATATATAAAC
TAATAAATAAAGTGCATCTAATCGTGGACTATCGATTATAGAACAGGTTCCTCTG
AAAAGACTAAAATACCGCCAAATTTTTTAATTTTCAAGAACATAACTAATACTAAT
TAAGTAAAAAAATTTACATTTTTAATAATAGGGTATCTAA
MEX_SAG_5
CGGGCGATGTGTACATATTTTAGAGCTTTAATCATCTTAAAAAAATCTAAAAAATT
ACTCTCAAATCCAAATTCAAATTTTTTTAACAAAAATTATTTTCAAATAAATTTATT
GTAACCCATTTCTTCTTTATTATAAACTACACCTTGATCTGATTTATTTTTTAATAA
AAAATTTTAAATATTTATAATTCTATTAAAATATTTAAACTACGACGATATATAAAC
TAATAAATAAAGTGCATCTAATCGTGGACTATCGATTATAGAACAGGTTCCTCTG

AAAAGACTAAAATACCGCCAAATTTTTTAATTTTCAAGAACATAACTAATACTAAT
TAAGTAAAAAAATTTACATTTTTAATAATAGGGTATCTAA
MEX_SCA
CGGGCGATGTGTACATATTTTAGAGCTTTAATCATCTTAAAAAAATCTAAAAAATT
ACTCTCAAATCCAAATTCAAATTTTTTTAACAAAAATTATTTTCAAATAAATTTATT
GTAACCCATTTCTTCTTTATTATAAACTACACCTTGATCTGATTTATTTTTTAATAA
AAAATTTTAAATATTTATAATTCTATTAAAATATTTAAACTACGACGATATATAAAC
TAATAAATAAAGTGCATCTAATCGTGGACTATCGATTATAGAACAGGTTCCTCTG
AAAAGACTAAAATACCGCCAAATTTTTTAATTTTCAAGAACATAACTAATACTAAT
TAAGTAAAAAAATTTACATTTTTAATAATAGGGTATCTAA
MEX_SFP
CGGGCGATGTGTACATATTTTAGAGCTTTAATCATCTTAAAAAAATCTAAAAAATT
ACTCTCAAATCCAAATTCAAATTTTTTTAACAAAAATTATTTTCAAATAAATTTATT
GTAACCCATTTCTTCTTTATTATAAACTACACCTTGATCTGATTTATTTTTTAATAA
AAAATTTTAAATATTTATAATTCTATTAAAATATTTAAACTACGACGATATATAAAC
TAATAAATAAAGTGCATCTAATCGTGGACTATCGATTATAGAACAGGTTCCTCTG
AAAAGACTAAAATACCGCCAAATTTTTTAATTTTCAAGAACATAACTAATACTAAT
TAAGTAAAAAAATTTACATTTTTAATAATAGGGTATCTAA
MEX_SJS_2
CGGGCGATGTGTACATATTTTAGAGCTTTAATCATCTTAAAAAAATCTAAAAAATT
ACTCTCAAATCCAAATTCAAATTTTTTTAACAAAAATTATTTTCAAATAAATTTATT
GTAACCCATTTCTTCTTTATTATAAACTACACCTTGATCTGATTTATTTTTTAATAA
AAAATTTTAAATATTTATAATTCTATTAAAATATTTAAACTACGACGATATATAAAC
TAATAAATAAAGTGCATCTAATCGTGGACTATCGATTATAGAACAGGTTCCTCTG
AAAAGACTAAAATACCGCCAAATTTTTTAATTTTCAAGAACATAACTAATACTAAT
TAAGTAAAAAAATTTACATTTTTAATAATAGGGTATCTAA
MEX_STO
CGGGCGATGTGTACATATTTTAGAGCTTTAATCATCTTAAAAAAATCTAAAAAATT
ACTCTCAAATCCAAATTCAAATTTTTTTAACAAAAATTATTTTCAAATAAATTTATT
GTAACCCATTTCTTCTTTATTATAAACTACACCTTGATCTGATTTATTTTTTAATAA
AAAATTTTAAATATTTATAATTCTATTAAAATATTTAAACTACGACGATATATAAAC
TAATAAATAAAGTGCATCTAATCGTGGACTATCGATTATAGAACAGGTTCCTCTG
AAAAGACTAAAATACCGCCAAATTTTTTAATTTTCAAGAACATAACTAATACTAAT
TAAGTAAAAAAATTTACATTTTTAATAATAGGGTATCTAA
MEX_TLA_1
CGGGCGATGTGTACATATTTTAGAGCTTTAATCATCTTAAAAAAATCTAAAAAATT
ACTCTCAAATCCAAATTCAAATTTTTTTAACAAAAATTATTTTCAAATAAATTTATT
GTAACCCATTTCTTCTTTATTATAAACTACACCTTGATCTGATTTATTTTTTAATAA
AAAATTTTAAATATTTATAATTCTATTAAAATATTTAAACTACGACGATATATAAAC
TAATAAATAAAGTGCATCTAATCGTGGACTATCGATTATAGAACAGGTTCCTCTG
AAAAGACTAAAATACCGCCAAATTTTTTAATTTTCAAGAACATAACTAATACTAAT
TAAGTAAAAAAATTTACATTTTTAATAATAGGGTATCTAA
MEX_XOT_3
CGGGCGATGTGTACATATTTTAGAGCTTTAATCATCTTAAAAAAATCTAAAAAATT
ACTCTCAAATCCAAATTCAAATTTTTTTAACAAAAATTATTTTAAAATAAATTTATT
GTAACCCATTTCTTCTTTATTATAAACTACACCTTGATCTGATTTATTTTTTAATAA
AAAATTTTAAATATTTATAATTCTATTAAAATATTTAAACTACGACGATATATAAAC
TAATAAATAAAGTGCATCTAATCGTGGACTATCGATTATAGAACAGGTTCCTCTG
AAAAGACTAAAATACCGCCAAATTTTTTAATTTTCAAGAACATAACTAATACTAAT
TAAGTAAAAAAATTTACATTTTTAATAATAGGGTATCTAA

MEX_YOH
CGGGCGATGTGTACATATTTTAGAGCTTTAATCATCTTAAAAAAATCTAAAAAATT
ACTCTCAAATCCAAATTCAAATTTTTTTAACAAAAATTATTTTCAAATAAATTTATT
GTAACCCATTTCTTCTTTATTATAAACTACACCTTGATCTGATTTATTTTTTAATAA
AAAATTTTAAATATTTATAATTCTATTAAAATATTTAAACTACGACGATATATAAAC
TAATAAATAAAGTGCATCTAATCGTGGACTATCGATTATAGAACAGGTTCCTCTG
AAAAGACTAAAATACCGCCAAATTTTTTAATTTTCAAGAACATAACTAATACTAAT
TAAGTAAAAAAATTTACATTTTTAATAATAGGGTATCTAA
moc5
CGGGCGATGTGTACATATTTTAGAGCTTTAATCATCTTAAAAAAATCTAAAAAATT
ACTCTCAAATCCAAATTCAAATTTTTTTAACAAAAATTATTTTAAAATAAATTTATT
GTAACCCATTTCTTCTTTATTATAAACTACACCTTGATCTGATTTATTTTTTAATAA
AAAATTTTAAATATTTATAATTCTATTAAAATATTTAAACTACGACGATATATAAAC
TAATAAATAAAGTGCATCTAATCGTGGACTATCGATTATAGAACAGGTTCCTCTG
AAAAGACTAAAATACCGCCAAATTTTTTAATTTTCAAGAACATAACTAATACTAAT
TAAGTAAAAAAATTTACATTTTTAATAATAGGGTATCTAA
PE2_1
CGGGCGATGTGTACATATTTTAGAGCTTTAATCATCTTAAAAAAATCTAAAAAATT
ACTCTCAAATCCAAATTCAAATTTTTTTAACAAAAATTATTTTCAAATAAATTTATT
GTAACCCATTTCTTCTTTATTATAAACTACACCTTGATCTGATTTATTTTTTAATAA
AAAATTTTAAATATTTATAATTCTATTAAAATATTTAAACTACGACGATATATAAAC
TAATAAATAAAGTGCATCTAATCGTGGACTATCGATTATAGAACAGGTTCCTCTG
AAAAGACTAAAATACCGCCAAATTTTTTAATTTTCAAGAACATAACTAATACTAAT
TAAGTAAAAAAATTTACATTTTTAATAATAGGGTATCTAA
PE2_3
CGGGCGATGTGTACATATTTTAGAGCTTTAATCATCTTAAAAAAATCTAAAAAATT
ACTCTCAAATCCAAATTCAAATTTTTTTAACAAAAATTATTTTCAAATAAATTTATT
GTAACCCATTTCTTCTTTATTATAAACTACACCTTGATCTGATTTATTTTTTAATAA
AAAATTTTAAATATTTATAATTCTATTAAAATATTTAAACTACGACGATATATAAAC
TAATAAATAAAGTGCATCTAATCGTGGACTATCGATTATAGAACAGGTTCCTCTG
AAAAGACTAAAATACCGCCAAATTTTTTAATTTTCAAGAACATAACTAATACTAAT
TAAGTAAAAAAATTTACATTTTTAATAATAGGGTATCTAA
PE2_4
CGGGCGATGTGTACATATTTTAGAGCTTTAATCATCTTAAAAAAATCTAAAAAATT
ACTCTCAAATCCAAATTCAAATTTTTTTAACAAAAATTATTTTCAAATAAATTTATT
GTAACCCATTTCTTCTTTATTATAAACTACACCTTGATCTGATTTATTTTTTAATAA
AAAATTTTAAATATTTATAATTCTATTAAAATATTTAAACTACGACGATATATAAAC
TAATAAATAAAGTGCATCTAATCGTGGACTATCGATTATAGAACAGGTTCCTCTG
AAAAGACTAAAATACCGCCAAATTTTTTAATTTTCAAGAACATAACTAATACTAAT
TAAGTAAAAAAATTTACATTTTTAATAATAGGGTATCTAA
PE2_5
CGGGCGATGTGTACATATTTTAGAGCTTTAATCATCTTAAAAAAATCTAAAAAATT
ACTCTCAAATCCAAATTCAAATTTTTTTAACAAAAATTATTTTCAAATAAATTTATT
GTAACCCATTTCTTCTTTATTATAAACTACACCTTGATCTGATTTATTTTTTAATAA
AAAATTTTAAATATTTATAATTCTATTAAAATATTTAAACTACGACGATATATAAAC
TAATAAATAAAGTGCATCTAATCGTGGACTATCGATTATAGAACAGGTTCCTCTG
AAAAGACTAAAATACCGCCAAATTTTTTAATTTTCAAGAACATAACTAATACTAAT
TAAGTAAAAAAATTTACATTTTTAATAATAGGGTATCTAA
PE2_6
CGGGCGATGTGTACATATTTTAGAGCTTTAATCATCTTAAAAAAATCTAAAAAATT

ACTCTCAAATCCAAATTCAAATTTTTTTAACAAAAATTATTTTCAAATAAATTTATT
GTAACCCATTTCTTCTTTATTATAAACTACACCTTGATCTGATTTATTTTTTAATAA
AAAATTTTAAATATTTATAATTCTATTAAAATATTTAAACTACGACGATATATAAAC
TAATAAATAAAGTGCATCTAATCGTGGACTATCGATTATAGAACAGGTTCCTCTG
AAAAGACTAAAATACCGCCAAATTTTTTAATTTTCAAGAACATAACTAATACTAAT
TAAGTAAAAAAATTTACATTTTTAATAATAGGGTATCTAA
PE2_7
CGGGCGATGTGTACATATTTTAGAGCTTTAATCATCTTAAAAAAATCTAAAAAATT
ACTCTCAAATCCAAATTCAAATTTTTTTAACAAAAATTATTTTCAAATAAATTTATT
GTAACCCATTTCTTCTTTATTATAAACTACACCTTGATCTGATTTATTTTTTAATAA
AAAATTTTAAATATTTATAATTCTATTAAAATATTTAAACTACGACGATATATAAAC
TAATAAATAAAGTGCATCTAATCGTGGACTATCGATTATAGAACAGGTTCCTCTG
AAAAGACTAAAATACCGCCAAATTTTTTAATTTTCAAGAACATAACTAATACTAAT
TAAGTAAAAAAATTTACATTTTTAATAATAGGGTATCTAA
PE_MAN_12
CGGGCGATGTGTACATATTTTAGAGCTTTAATCATCTTAAAAAAATCTAAAAAATT
ACTCTCAAATCCAAATTCAAATTTTTTTAACAAAAATTATTTTCAAATAAATTTATT
GTAACCCATTTCTTCTTTATTATAAACTACACCTTGATCTGATTTATTTTTTAATAA
AAAATTTTAAATATTTATAATTCTATTAAAATATTTAAACTACGACGATATATAAAC
TAATAAATAAAGTGCATCTAATCGTGGACTATCGATTATAGAACAGGTTCCTCTG
AAAAGACTAAAATACCGCCAAATTTTTTAATTTTCAAGAACATAACTAATACTAAT
TAAGTAAAAAAATTTACATTTTTAATAATAGGGTATCTAA
PE_MAN_13
CGGGCGATGTGTACATATTTTAGAGCTTTAATCATCTTAAAAAAATCTAAAAAATT
ACTCTCAAATCCAAATTCAAATTTTTTTAACAAAAATTATTTTCAAATAAATTTATT
GTAACCCATTTCTTCTTTATTATAAACTACACCTTGATCTGATTTATTTTTTAATAA
AAAATTTTAAATATTTATAATTCTATTAAAATATTTAAACTACGACGATATATAAAC
TAATAAATAAAGTGCATCTAATCGTGGACTATCGATTATAGAACAGGTTCCTCTG
AAAAGACTAAAATACCGCCAAATTTTTTAATTTTCAAGAACATAACTAATACTAAT
TAAGTAAAAAAATTTACATTTTTAATAATAGGGTATCTAA
PE_MAN_15
CGGGCGATGTGTACATATTTTAGAGCTTTAATCATCTTAAAAAAATCTAAAAAATT
ACTCTCAAATCCAAATTCAAATTTTTTTAACAAAAATTATTTTCAAATAAATTTATT
GTAACCCATTTCTTCTTTATTATAAACTACACCTTGATCTGATTTATTTTTTAATAA
AAAATTTTAAATATTTATAATTCTATTAAAATATTTAAACTACGACGATATATAAAC
TAATAAATAAAGTGCATCTAATCGTGGACTATCGATTATAGAACAGGTTCCTCTG
AAAAGACTAAAATACCGCCAAATTTTTTAATTTTCAAGAACATAACTAATACTAAT
TAAGTAAAAAAATTTACATTTTTAATAATAGGGTATCTAA
PE_MAN_16
CGGGCGATGTGTACATATTTTAGAGCTTTAATCATCTTAAAAAAATCTAAAAAATT
ACTCTCAAATCCAAATTCAAATTTTTTTAACAAAAATTATTTTCAAATAAATTTATT
GTAACCCATTTCTTCTTTATTATAAACTACACCTTGATCTGATTTATTTTTTAATAA
AAAATTTTAAATATTTATAATTCTATTAAAATATTTAAACTACGACGATATATAAAC
TAATAAATAAAGTGCATCTAATCGTGGACTATCGATTATAGAACAGGTTCCTCTG
AAAAGACTAAAATACCGCCAAATTTTTTAATTTTCAAGAACATAACTAATACTAAT
TAAGTAAAAAAATTTACATTTTTAATAATAGGGTATCTAA
PE_MAN_19
CGGGCGATGTGTACATATTTTAGAGCTTTAATCATCTTAAAAAAATCTAAAAAATT
ACTCTCAAATCCAAATTCAAATTTTTTTAACAAAAATTATTTTCAAATAAATTTATT
GTAACCCATTTCTTCTTTATTATAAACTACACCTTGATCTGATTTATTTTTTAATAA

AAAATTTTAAATATTTATAATTCTATTAAAATATTTAAACTACGACGATATATAAAC
TAATAAATAAAGTGCATCTAATCGTGGACTATCGATTATAGAACAGGTTCCTCTG
AAAAGACTAAAATACCGCCAAATTTTTTAATTTTCAAGAACATAACTAATACTAAT
TAAGTAAAAAAATTTACATTTTTAATAATAGGGTATCTAA
PE_MAN_2
CGGGCGATGTGTACATATTTTAGAGCTTTAATCATCTTAAAAAAATCTAAAAAATT
ACTCTCAAATCCAAATTCAAATTTTTTTAACAAAAATTATTTTCAAATAAATTTATT
GTAACCCATTTCTTCTTTATTATAAACTACACCTTGATCTGATTTATTTTTTAATAA
AAAATTTTAAATATTTATAATTCTATTAAAATATTTAAACTACGACGATATATAAAC
TAATAAATAAAGTGCATCTAATCGTGGACTATCGATTATAGAACAGGTTCCTCTG
AAAAGACTAAAATACCGCCAAATTTTTTAATTTTCAAGAACATAACTAATACTAAT
TAAGTAAAAAAATTTACATTTTTAATAATAGGGTATCTAA
PE_MAN_23
CGGGCGATGTGTACATATTTTAGAGCTTTAATCATCTTAAAAAAATCTAAAAAATT
ACTCTCAAATCCAAATTCAAATTTTTTTAACAAAAATTATTTTCAAATAAATTTATT
GTAACCCATTTCTTCTTTATTATAAACTACACCTTGATCTGATTTATTTTTTAATAA
AAAATTTTAAATATTTATAATTCTATTAAAATATTTAAACTACGACGATATATAAAC
TAATAAATAAAGTGCATCTAATCGTGGACTATCGATTATAGAACAGGTTCCTCTG
AAAAGACTAAAATACCGCCAAATTTTTTAATTTTCAAGAACATAACTAATACTAAT
TAAGTAAAAAAATTTACATTTTTAATAATAGGGTATCTAA
PE_MAN_29
CGGGCGATGTGTACATATTTTAGAGCTTTAATCATCTTAAAAAAATCTAAAAAATT
ACTCTCAAATCCAAATTCAAATTTTTTTAACAAAAATTATTTTCAAATAAATTTATT
GTAACCCATTTCTTCTTTATTATAAACTACACCTTGATCTGATTTATTTTTTAATAA
AAAATTTTAAATATTTATAATTCTATTAAAATATTTAAACTACGACGATATATAAAC
TAATAAATAAAGTGCATCTAATCGTGGACTATCGATTATAGAACAGGTTCCTCTG
AAAAGACTAAAATACCGCCAAATTTTTTAATTTTCAAGAACATAACTAATACTAAT
TAAGTAAAAAAATTTACATTTTTAATAATAGGGTATCTAA
PE_MAN_6
CGGGCGATGTGTACATATTTTAGAGCTTTAATCATCTTAAAAAAATCTAAAAAATT
ACTCTCAAATCCAAATTCAAATTTTTTTAACAAAAATTATTTTCAAATAAATTTATT
GTAACCCATTTCTTCTTTATTATAAACTACACCTTGATCTGATTTATTTTTTAATAA
AAAATTTTAAATATTTATAATTCTATTAAAATATTTAAACTACGACGATATATAAAC
TAATAAATAAAGTGCATCTAATCGTGGACTATCGATTATAGAACAGGTTCCTCTG
AAAAGACTAAAATACCGCCAAATTTTTTAATTTTCAAGAACATAACTAATACTAAT
TAAGTAAAAAAATTTACATTTTTAATAATAGGGTATCTAA
PE_MAN_8
CGGGCGATGTGTACATATTTTAGAGCTTTAATCATCTTAAAAAAATCTAAAAAATT
ACTCTCAAATCCAAATTCAAATTTTTTTAACAAAAATTATTTTCAAATAAATTTATT
GTAACCCATTTCTTCTTTATTATAAACTACACCTTGATCTGATTTATTTTTTAATAA
AAAATTTTAAATATTTATAATTCTATTAAAATATTTAAACTACGACGATATATAAAC
TAATAAATAAAGTGCATCTAATCGTGGACTATCGATTATAGAACAGGTTCCTCTG
AAAAGACTAAAATACCGCCAAATTTTTTAATTTTCAAGAACATAACTAATACTAAT
TAAGTAAAAAAATTTACATTTTTAATAATAGGGTATCTAA
pit1
CGGGCGATGTGTACATATTTTAGAGCTTTAATCATCTTAAAAAAATCTAAAAAATT
ACTCTCAAATCCAAATTCAAATTTTTTTAACAAAAATTATTTTAAAATAAATTTATT
GTAACCCATTTCTTCTTTATTATAAACTACACCTTGATCTGATTTATTTTTTAATAA
AAAATTTTAAATATTTATAATTCTATTAAAATATTTAAACTACGACGATATATAAAC
TAATAAATAAAGTGCATCTAATCGTGGACTATCGATTATAGAACAGGTTCCTCTG

AAAAGACTAAAATACCGCCAAATTTTTTAATTTTCAAGAACATAACTAATACTAAT
TAAGTAAAAAAATTTACATTTTTAATAATAGGGTATCTAA
pit2
CGGGCGATGTGTACATATTTTAGAGCTTTAATCATCTTAAAAAAATCTAAAAAATT
ACTCTCAAATCCAAATTCAAATTTTTTTAACAAAAATTATTTTAAAATAAATTTATT
GTAACCCATTTCTTCTTTATTATAAACTACACCTTGATCTGATTTATTTTTTAATAA
AAAATTTTAAATATTTATAATTCTATTAAAATATTTAAACTACGACGATATATAAAC
TAATAAATAAAGTGCATCTAATCGTGGACTATCGATTATAGAACAGGTTCCTCTG
AAAAGACTAAAATACCGCCAAATTTTTTAATTTTCAAGAACATAACTAATACTAAT
TAAGTAAAAAAATTTACATTTTTAATAATAGGGTATCTAA
pit4
CGGGCGATGTGTACATATTTTAGAGCTTTAATCATCTTAAAAAAATCTAAAAAATT
ACTCTCAAATCCAAATTCAAATTTTTTTAACAAAAATTATTTTAAAATAAATTTATT
GTAACCCATTTCTTCTTTATTATAAACTACACCTTGATCTGATTTATTTTTTAATAA
AAAATTTTAAATATTTATAATTCTATTAAAATATTTAAACTACGACGATATATAAAC
TAATAAATAAAGTGCATCTAATCGTGGACTATCGATTATAGAACAGGTTCCTCTG
AAAAGACTAAAATACCGCCAAATTTTTTAATTTTCAAGAACATAACTAATACTAAT
TAAGTAAAAAAATTTACATTTTTAATAATAGGGTATCTAA
ron3
CGGGCGATGTGTACATATTTTAGAGCTTTAATCATCTTAAAAAAATCTAAAAAATT
ACTCTCAAATCCAAATTCAAATTTTTTTAACAAAAATTATTTTAAAATAAATTTATT
GTAACCCATTTCTTCTTTATTATAAACTACACCTTGATCTGATTTATTTTTTAATAA
AAAATTTTAAATATTTATAATTCTATTAAAATATTTAAACTACGACGATATATAAAC
TAATAAATAAAGTGCATCTAATCGTGGACTATCGATTATAGAACAGGTTCCTCTG
AAAAGACTAAAATACCGCCAAATTTTTTAATTTTCAAGAACATAACTAATACTAAT
TAAGTAAAAAAATTTACATTTTTAATAATAGGGTATCTAA
sob1
CGGGCGATGTGTACATATTTTAGAGCTTTAATCATCTTAAAAAAATCTAAAAAATT
ACTCTCAAATCCAAATTCAAATTTTTTTAACAAAAATTATTTTAAAATAAATTTATT
GTAACCCATTTCTTCTTTATTATAAACTACACCTTGATCTGATTTATTTTTTAATAA
AAAATTTTAAATATTTATAATTCTATTAAAATATTTAAACTACGACGATATATAAAC
TAATAAATAAAGTGCATCTAATCGTGGACTATCGATTATAGAACAGGTTCCTCTG
AAAAGACTAAAATACCGCCAAATTTTTTAATTTTCAAGAACATAACTAATACTAAT
TAAGTAAAAAAATTTACATTTTTAATAATAGGGTATCTAA
SPA_PRA_1
CGGGCGATGTGTACATATTTTAGAGCTTTAATCATCTTAAAAAAATCTAAAAAATT
ACTCTCAAATCCAAATTCAAATTTTTTTAACAAAAATTATTTTAAAATAAATTTATT
GTAACCCATTTCTTCTTTATTATAAACTACACCTTGATCTGATTTATTTTTTAATAA
AAAATTTTAAATATTTATAATTCTATTAAAATATTTAAACTACGACGATATATAAAC
TAATAAATAAAGTGCATCTAATCGTGGACTATCGATTATAGAACAGGTTCCTCTG
AAAAGACTAAAATACCGCCAAATTTTTTAATTTTCAAGAACATAACTAATACTAAT
TAAGTAAAAAAATTTACATTTTTAATAATAGGGTATCTAA
SWI_NEU_1
CGGGCGATGTGTACATATTTTAGAGCTTTAATCATCTTAAAAAAATCTAAAAAATT
ACTCTCAAATCCAAATTCAAATTTTTTTAACAAAAATTATTTTAAAATAAATTTATT
GTAACCCATTTCTTCTTTATTATAAACTACACCTTGATCTGATTTATTTTTTAATAA
AAAATTTTAAATATTTATAATTCTATTAAAATATTTAAACTACGACGATATATAAAC
TAATAAATAAAGTGCATCTAATCGTGGACTATCGATTATAGAACAGGTTCCTCTG
AAAAGACTAAAATACCGCCAAATTTTTTAATTTTCAAGAACATAACTAATACTAAT
TAAGTAAAAAAATTTACATTTTTAATAATAGGGTATCTAA

tol1
CGGGCGATGTGTACATATTTTAGAGCTTTAATCATCTTAAAAAAATCTAAAAAATT
ACTCTCAAATCCAAATTCAAATTTTTTTAACAAAAATTATTTTAAAATAAATTTATT
GTAACCCATTTCTTCTTTATTATAAACTACACCTTGATCTGATTTATTTTTTAATAA
AAAATTTTAAATATTTATAATTCTATTAAAATATTTAAACTACGACGATATATAAAC
TAATAAATAAAGTGCATCTAATCGTGGACTATCGATTATAGAACAGGTTCCTCTG
AAAAGACTAAAATACCGCCAAATTTTTTAATTTTCAAGAACATAACTAATACTAAT
TAAGTAAAAAAATTTACATTTTTAATAATAGGGTATCTAA
;

endblock;

datasetS2

#NEXUS

begin data;
dimensions ntax=56 nchar=465;
format datatype=dna gap=- interleave=no;
matrix

afr19
CAGACCTAATATTTCCAGCTTCTGCACCAAAAATTAATTTTAATCCAACATCGAG
GTCGCAATCTTTATCTTCGATTAGAACTCTTTGATAATATTACGCTGTTATCCCTA
AGGTAATTAAATCTTACAATTATTACTAATAGCTCAAGTATACATAAATTAATGATT
TAAATTTAAAAAAAGTTCAATTAATTTTTCAATCACCCCAATTAAATAATTAAAAAA
ATTAAAAATTTTTATACTaAAAAAAAATTAAAATTTTTAAATATGAAACTCTATAGG
GTCTTCTCGTCTTTTAAATTAATTTTAGCTTTTTAACTAAAAAATAAATTTTTAATG
TTTAAATAGAGACAGAAATTTTTTTATCCAACCATTCATTCCAGCTTCTAATTAAG
AAACTAATGATTATGCTACCTTAGCACGGTCAAAATACCGCGGCCATTTAAATCC
TCAGAGGGCAGGCTA
afr9
CAGACCTAATATTTCCAGCTTCTGCACCAAAAATTAATTTTAATCCAACATCGAG
GTCGCAATCTTTATCTTCGATTAGAACTCTTTGATAATATTACGCTGTTATCCCTA
AGGTAATTAAATCTTACAATTATTACTAATAGCTCAAGTATACATAAATTAATGATT
TAAATTTAAAAAAAGTTCGATTAATTTTTCAATCACCCCAATTAAATAATTAAAAAA
ATTAAAAATTTTTATACTaAAAAAAAATTAAAATTTTTAAATATGAAACTCTATAGG
GTCTTCTCGTCTTTTAAATTAATCTTAGCTTTTTAACTAAAAAATAAATTTTTAATG
TTTAAATAGAGACAGAAATTTTTTTATCCAACCATTCATTCCAGCTTCTAATTAAG
AAACTAATGATTATGCTACCTTAGCACGGTCAAAATACCGCGGCCATTTAAATCC
TCAGAGGGCAGGCTA
AOPE_12
CAGACCTAATATCCCCAGCTTCTACACCAAAAATTAATTTTAATCCAACATCGAG
GTCGCAATCTTTATCTTCGATTAGAACTCTTTGATAATATTACGCTGTTATCCCTA
AGGTAATTAAATCTTACAATTATTACTAATAGCTCAAGTATACATAAATTAATGATT
TAAATTTAAAAAAAGTTCAATTAATTTTTCAATCACCCCAATTAAATAATTAAAAAA
ATTAAAAATTTTTATACTaAAAAAAAATTAAAATTTTTAAATATGAAACTCTATAGG
GTCTTCTCGTCTTTTAAATTAATTTTAGCTTTTTAACTAAAAAATAAATTTTTAATG
TTTAAATAGAGACAGAAATTTTTTTATCCAACCATTCATTCCAGCTTCTAATTAAG
AAACTAATGATTATGCTACCTTAGCACGGTCAAAATACCGCGGCCATTTAAATCC
TCAGAGGGCAGGCTA
AOPE_13
CAGACCTAATATCCCCAGCTTCTACACCAAAAATTAATTTTAATCCAACATCGAG
GTCGCAATCTTTATCTTCGATTAGAACTCTTTGATAATATTACGCTGTTATCCCTA
AGGTAATTAAATCTTACAATTATTACTAATAGCTCAAGTATACATAAATTAATGATT
TAAATTTAAAAAAAGTTCAATTAATTTTTCAATCACCCCAATTAAATAATTAAAAAA
ATTAAAAATTTTTATACTaAAAAAAAATTAAAATTTTTAAATATGAAACTCTATAGG
GTCTTCTCGTCTTTTAAATTAATTTTAGCTTTTTAACTAAAAAATAAATTTTTAATG
TTTAAATAGAGACAGAAATTTTTTTATCCAACCATTCATTCCAGCTTCTAATTAAG
AAACTAATGATTATGCTACCTTAGCACGGTCAAAATACCGCGGCCATTTAAATCC
TCAGAGGGCAGGCTA

AOPE_14
CAGACCTAATATCCCCAGCTTCTACACCAAAAATTAATTTTAATCCAACATCGAG
GTCGCAATCTTTATCTTCGATTAGAACTCTTTGATAATATTACGCTGTTATCCCTA
AGGTAATTAAATCTTACAATTATTACTAATAGCTCAAGTATACATAAATTAATGATT
TAAATTTAAAAAAAGTTCAATTAATTTTTCAATCACCCCAATTAAATAATTAAAAAA
ATTAAAAATTTTTATACTaAAAAAAAAATTAAAATTTTTAAATATGAAACTCTATAGG
GTCTTCTCGTCTTTTAAATTAATTTTAGCTTTTTAACTAAAAAATAAATTTTTAATG
TTTAAATAGAGACAGAAATTTTTTTATCCAACCATTCATTCCAGCTTCTAATTAAG
AAACTAATGATTATGCTACCTTAGCACGGTCAAAATACCGCGGCCATTTAAATCC
TCAGAGGGCAGGCTA
AOPE_4
CAGACCTAATATCCCCAGCTTCTACACCAAAAATTAATTTTAATCCAACATCGAG
GTCGCAATCTTTATCTTCGATTAGAACTCTTTGATAATATTACGCTGTTATCCCTA
AGGTAATTAAATCTTACAATTATTACTAATAGCTCAAGTATACATAAATTAATGATT
TAAATTTAAAAAAAGTTCAATTAATTTTTCAATCACCCCAATTAAATAATTAAAAAA
ATTAAAAATTTTTATACTaAAAAAAAAATTAAAATTTTTAAATATGAAACTCTATAGG
GTCTTCTCGTCTTTTAAATTAATTTTAGCTTTTTAACTAAAAAATAAATTTTTAATG
TTTAAATAGAGACAGAAATTTTTTTATCCAACCATTCATTCCAGCTTCTAATTAAG
AAACTAATGATTATGCTACCTTAGCACGGTCAAAATACCGCGGCCATTTAAATCC
TCAGAGGGCAGGCTA
bja1
CAGACCTAATATTTCCAGCTTCTGCACCAAAAATTAATTTTAATCCAACATCGAG
GTCGCAATCTTTATCTTCGATTAGAACTCTTTGATAATATTACGCTGTTATCCCTA
AGGTAATTAAATCTTACAATTATTACTAATAGCTCAAGTATACATAAATTAATGATT
TAAATTTAAAAAAAGTTCAATTAATTTTTCAATCACCCCAATTAAATAATTAAAAAA
ATTAAAAATTTTTATACTaAAAAAAAAATTAAAATTTTTAAATATGAAACTCTATAGG
GTCTTCTCGTCTTTTAAATTAATTTTAGCTTTTTAACTAAAAAATAAATTTTTAATG
TTTAAATAGAGACAGAAATTTTTTTATCCAACCATTCATTCCAGCTTCTAATTAAG
AAACTAATGATTATGCTACCTTAGCACGGTCAAAATACCGCGGCCATTTAAATCC
TCAGAGGGCAGGCTA
CAM_YAH
CAGACCTAATATTTCCAGCTTCTGCACCAAAAATTAATTTTAATCCAACATCGAG
GTCGCAATCTTTATCTTCGATTAGAACTCTTTGATAATATTACGCTGTTATCCCTA
AGGTAATTAAATCTTACAATTATTACTAATAGCTCAAGTATACATAAATTAATGATT
TAAATTTAAAAAAAGTTCGATTAATTTTTCAATCACCCCAATTAAATAATTAAAAAA
ATTAAAAATTTTTATACTaAAAAAAAAATTAAAATTTTTAAATATGAAACTCTATAGG
GTCTTCTCGTCTTTTAAATTAATCTTAGCTTTTTAACTAAAAAATAAATTTTTAATG
TTTAAATAGAGACAGAAATTTTTTTATCCAACCATTCATTCCAGCTTCTAATTAAG
AAACTAATGATTATGCTACCTTAGCACGGTCAAAATACCGCGGCCATTTAAATCC
TCAGAGGGCAGGCTA
CL1_1
CAGACCTAATATCCCCAGCTTCTACACCAAAAATTAATTTTAATCCAACATCGAG
GTCGCAATCTTTATCTTCGATTAGAACTCTTTGATAATATTACGCTGTTATCCCTA
AGGTAATTAAATCTTACAATTATTACTAATAGCTCAAGTATACATAAATTAATGATT
TAAATTTAAAAAAAGTTCAATTAATTTTTCAATCACCCCAATTAAATAATTAAAAAA
ATTAAAAATTTTTATACTAAAAAAAAAATTAAAATTTTTAAATATGAAACTCTATAGG
GTCTTCTCGTCTTTTAAATTAATTTTAGCTTTTTAACTAAAAAATAAATTTTTAATG
TTTAAATAGAGACAGAAATTTTTTTATCCAACCATTCATTCCAGCTTCTAATTAAG
AAACTAATGATTATGCTACCTTAGCACGGTCAAAATACCGCGGCCATTTAAATCC
TCAGAGGGCAGGCTA

CL1_2
CAGACCTAATATCCCCAGCTTCTACACCAAAAATTAATTTTAATCCAACATCGAG
GTCGCAATCTTTATCTTCGATTAGAACTCTTTGATAATATTACGCTGTTATCCCTA
AGGTAATTAAATCTTACAATTATTACTAATAGCTCAAGTATACATAAATTAATGATT
TAAATTTAAAAAAAGTTCAATTAATTTTTCAATCACCCCAATTAAATAATTAAAAAA
ATTAAAAATTTTTATACTAAAAAAAAAATTAAAATTTTTAAATATGAAACTCTATAGG
GTCTTCTCGTCTTTTAAATTAATTTTAGCTTTTTAACTAAAAAATAAATTTTTAATG
TTTAAATAGAGACAGAAATTTTTTTATCCAACCATTCATTCCAGCTTCTAATTAAG
AAACTAATGATTATGCTACCTTAGCACGGTCAAAATACCGCGGCCATTTAAATCC
TCAGAGGGCAGGCTA
CL1_3
CAGACCTAATATCCCCAGCTTCTACACCAAAAATTAATTTTAATCCAACATCGAG
GTCGCAATCTTTATCTTCGATTAGAACTCTTTGATAATATTACGCTGTTATCCCTA
AGGTAATTAAATCTTACAATTATTACTAATAGCTCAAGTATACATAAATTAATGATT
TAAATTTAAAAAAAGTTCAATTAATTTTTCAATCACCCCAATTAAATAATTAAAAAA
ATTAAAAATTTTTATACTAAAAAAAAAATTAAAATTTTTAAATATGAAACTCTATAGG
GTCTTCTCGTCTTTTAAATTAATTTTAGCTTTTTAACTAAAAAATAAATTTTTAATG
TTTAAATAGAGACAGAAATTTTTTTATCCAACCATTCATTCCAGCTTCTAATTAAG
AAACTAATGATTATGCTACCTTAGCACGGTCAAAATACCGCGGCCATTTAAATCC
TCAGAGGGCAGGCTA
CL1_4
CAGACCTAATATCCCCAGCTTCTACACCAAAAATTAATTTTAATCCAACATCGAG
GTCGCAATCTTTATCTTCGATTAGAACTCTTTGATAATATTACGCTGTTATCCCTA
AGGTAATTAAATCTTACAATTATTACTAATAGCTCAAGTATACATAAATTAATGATT
TAAATTTAAAAAAAGTTCAATTAATTTTTCAATCACCCCAATTAAATAATTAAAAAA
ATTAAAAATTTTTATACTAAAAAAAAAATTAAAATTTTTAAATATGAAACTCTATAGG
GTCTTCTCGTCTTTTAAATTAATTTTAGCTTTTTAACTAAAAAATAAATTTTTAATG
TTTAAATAGAGACAGAAATTTTTTTATCCAACCATTCATTCCAGCTTCTAATTAAG
AAACTAATGATTATGCTACCTTAGCACGGTCAAAATACCGCGGCCATTTAAATCC
TCAGAGGGCAGGCTA
CL1_5
CAGACCTAATATCCCCAGCTTCTACACCAAAAATTAATTTTAATCCAACATCGAG
GTCGCAATCTTTATCTTCGATTAGAACTCTTTGATAATATTACGCTGTTATCCCTA
AGGTAATTAAATCTTACAATTATTACTAATAGCTCAAGTATACATAAATTAATGATT
TAAATTTAAAAAAAGTTCAATTAATTTTTCAATCACCCCAATTAAATAATTAAAAAA
ATTAAAAATTTTTATACTAAAAAAAAAATTAAAATTTTTAAATATGAAACTCTATAGG
GTCTTCTCGTCTTTTAAATTAATTTTAGCTTTTTAACTAAAAAATAAATTTTTAATG
TTTAAATAGAGACAGAAATTTTTTTATCCAACCATTCATTCCAGCTTCTAATTAAG
AAACTAATGATTATGCTACCTTAGCACGGTCAAAATACCGCGGCCATTTAAATCC
TCAGAGGGCAGGCTA
CL1_6
CAGACCTAATATCCCCAGCTTCTACACCAAAAATTAATTTTAATCCAACATCGAG
GTCGCAATCTTTATCTTCGATTAGAACTCTTTGATAATATTACGCTGTTATCCCTA
AGGTAATTAAATCTTACAATTATTACTAATAGCTCAAGTATACATAAATTAATGATT
TAAATTTAAAAAAAGTTCAATTAATTTTTCAATCACCCCAATTAAATAATTAAAAAA
ATTAAAAATTTTTATACTAAAAAAAAAATTAAAATTTTTAAATATGAAACTCTATAGG
GTCTTCTCGTCTTTTAAATTAATTTTAGCTTTTTAACTAAAAAATAAATTTTTAATG
TTTAAATAGAGACAGAAATTTTTTTATCCAACCATTCATTCCAGCTTCTAATTAAG
AAACTAATGATTATGCTACCTTAGCACGGTCAAAATACCGCGGCCATTTAAATCC
TCAGAGGGCAGGCTA

CL1_7
CAGACCTAATATCCCCAGCTTCTACACCAAAAATTAATTTTAATCCAACATCGAG
GTCGCAATCTTTATCTTCGATTAGAACTCTTTGATAATATTACGCTGTTATCCCTA
AGGTAATTAAATCTTACAATTATTACTAATAGCTCAAGTATACATAAATTAATGATT
TAAATTTAAAAAAAGTTCAATTAATTTTTCAATCACCCCAATTAAATAATTAAAAAA
ATTAAAAATTTTTATACTAAAAAAAAAATTAAAATTTTTAAATATGAAACTCTATAGG
GTCTTCTCGTCTTTTAAATTAATTTTAGCTTTTTAACTAAAAAATAAATTTTTAATG
TTTAAATAGAGACAGAAATTTTTTTATCCAACCATTCATTCCAGCTTCTAATTAAG
AAACTAATGATTATGCTACCTTAGCACGGTCAAAATACCGCGGCCATTTAAATCC
TCAGAGGGCAGGCTA
CL1_8
CAGACCTAATATCCCCAGCTTCTACACCAAAAATTAATTTTAATCCAACATCGAG
GTCGCAATCTTTATCTTCGATTAGAACTCTTTGATAATATTACGCTGTTATCCCTA
AGGTAATTAAATCTTACAATTATTACTAATAGCTCAAGTATACATAAATTAATGATT
TAAATTTAAAAAAAGTTCAATTAATTTTTCAATCACCCCAATTAAATAATTAAAAAA
ATTAAAAATTTTTATACTAAAAAAAAAATTAAAATTTTTAAATATGAAACTCTATAGG
GTCTTCTCGTCTTTTAAATTAATTTTAGCTTTTTAACTAAAAAATAAATTTTTAATG
TTTAAATAGAGACAGAAATTTTTTTATCCAACCATTCATTCCAGCTTCTAATTAAG
AAACTAATGATTATGCTACCTTAGCACGGTCAAAATACCGCGGCCATTTAAATCC
TCAGAGGGCAGGCTA
CL1_9
CAGACCTAATATCCCCAGCTTCTACACCAAAAATTAATTTTAATCCAACATCGAG
GTCGCAATCTTTATCTTCGATTAGAACTCTTTGATAATATTACGCTGTTATCCCTA
AGGTAATTAAATCTTACAATTATTACTAATAGCTCAAGTATACATAAATTAATGATT
TAAATTTAAAAAAAGTTCAATTAATTTTTCAATCACCCCAATTAAATAATTAAAAAA
ATTAAAAATTTTTATACTAAAAAAAAAATTAAAATTTTTAAATATGAAACTCTATAGG
GTCTTCTCGTCTTTTAAATTAATTTTAGCTTTTTAACTAAAAAATAAATTTTTAATG
TTTAAATAGAGACAGAAATTTTTTTATCCAACCATTCATTCCAGCTTCTAATTAAG
AAACTAATGATTATGCTACCTTAGCACGGTCAAAATACCGCGGCCATTTAAATCC
TCAGAGGGCAGGCTA
CL2_1
CAGACCTAATATCCCCAGCTTCTACACCAAAAATTAATTTTAATCCAACATCGAG
GTCGCAATCTTTATCTTCGATTAGAACTCTTTGATAATATTACGCTGTTATCCCTA
AGGTAATTAAATCTTACAATTATTACTAATAGCTCAAGTATACATAAATTAATGATT
TAAATTTAAAAAAAGTTCAATTAATTTTTCAATCACCCCAATTAAATAATTAAAAAA
ATTAAAAATTTTTATACTAAAAAAAAAATTAAAATTTTTAAATATGAAACTCTATAGG
GTCTTCTCGTCTTTTAAATTAATTTTAGCTTTTTAACTAAAAAATAAATTTTTAATG
TTTAAATAGAGACAGAAATTTTTTTATCCAACCATTCATTCCAGCTTCTAATTAAG
AAACTAATGATTATGCTACCTTAGCACGGTCAAAATACCGCGGCCATTTAAATCC
TCAGAGGGCAGGCTA
CL2_2
CAGACCTAATATCCCCAGCTTCTACACCAAAAATTAATTTTAATCCAACATCGAG
GTCGCAATCTTTATCTTCGATTAGAACTCTTTGATAATATTACGCTGTTATCCCTA
AGGTAATTAAATCTTACAATTATTACTAATAGCTCAAGTATACATAAATTAATGATT
TAAATTTAAAAAAAGTTCAATTAATTTTTCAATCACCCCAATTAAATAATTAAAAAA
ATTAAAAATTTTTATACTAAAAAAAAAATTAAAATTTTTAAATATGAAACTCTATAGG
GTCTTCTCGTCTTTTAAATTAATTTTAGCTTTTTAACTAAAAAATAAATTTTTAATG
TTTAAATAGAGACAGAAATTTTTTTATCCAACCATTCATTCCAGCTTCTAATTAAG
AAACTAATGATTATGCTACCTTAGCACGGTCAAAATACCGCGGCCATTTAAATCC
TCAGAGGGCAGGCTA

EQU_5
CAGACCTAATATCCCCAGCTTCTACACCAAAAATTAATTTTAATCCAACATCGAG
GTCGCAATCTTTATCTTCGATTAGAACTCTTTGATAATATTACGCTGTTATCCCTA
AGGTAATTAAATCTTACAATTATTACTAATAGCTCAAGTATACATAAATTAATGATT
TAAATTTAAAAAAAGTTCAATTAATTTTTCAATCACCCCAATTAAATAATTAAAAAA
ATTAAAAATTTTTATACTAAAAAAAAATTAAAATTTTTAAATATGAAACTCTATAGG
GTCTTCTCGTCTTTTAAATTAATTTTAGCTTTTTAACTAAAAAATAAATTTTTAATG
TTTAAATAGAGACAGAAATTTTTTTATCCAACCATTCATTCCAGCTTCTAATTAAG
AAACTAATGATTATGCTACCTTAGCACGGTCAAAATACCGCGGCCATTTAAATCC
TCAGAGGGCAGGCTA
FRA_CHA_5
CAGACCTAATATTTCCAGCTTCTGCACCAAAAATTAATTTTAATCCAACATCGAG
GTCGCAATCTTTATCTTCGATTAGAACTCTTTGATAATATTACGCTGTTATCCCTA
AGGTAATTAAATCTTACAATTATTACTAATAGCTCAAGTATACATAAATTAATGATT
TAAATTTAAAAAAAGTTCGATTAATTTTTCAATCACCCCAATTAAATAATTAAAAAA
ATTAAAAATTTTTATACTaAAAAAAAATTAAAATTTTTAAATATGAAACTCTATAGG
GTCTTCTCGTCTTTTAAATTAATCTTAGCTTTTTAACTAAAAAATAAATTTTTAATG
TTTAAATAGAGACAGAAATTTTTTTATCCAACCATTCATTCCAGCTTCTAATTAAG
AAACTAATGATTATGCTACCTTAGCACGGTCAAAATACCGCGGCCATTTAAATCC
TCAGAGGGCAGGCTA
hev1
CAGACCTAATATTTCCAGCTTCTGCACCAAAAATTAATTTTAATCCAACATCGAG
GTCGCAATCTTTATCTTCGATTAGAACTCTTTGATAATATTACGCTGTTATCCCTA
AGGTAATTAAATCTTACAATTATTACTAATAGCTCAAGTATACATAAATTAATGATT
TAAATTTAAAAAAAGTTCAATTAATTTTTCAATCACCCCAATTAAATAATTAAAAAA
ATTAAAAATTTTTATACTaAAAAAAAATTAAAATTTTTAAATATGAAACTCTATAGG
GTCTTCTCGTCTTTTAAATTAATTTTAGCTTTTTAACTAAAAAATAAATTTTTAATG
TTTAAATAGAGACAGAAATTTTTTTATCCAACCATTCATTCCAGCTTCTAATTAAG
AAACTAATGATTATGCTACCTTAGCACGGTCAAAATACCGCGGCCATTTAAATCC
TCAGAGGGCAGGCTA
MEX_COE_2
CAGACCTAATATTCCCAGCTTCTGCACCAAAAATTAATTTTAATCCAACATCGAG
GTCGCAATCTTTATCTTCGATTAGAACTCTTTGATAATATTACGCTGTTATCCCTA
AGGTAATTAAATCTTACAATTATTACTAATAGCTCAAGTATACATAAATTAATGATT
TAAATTTAAAAAAAGTTCAATTAATTTTTCAATCACCCCAATTAAATAATTAAAAAA
ATTAAAAATTTTTATACTaAAAAAAAATTAAAATTTTTAAATATGAAACTCTATAGG
GTCTTCTCGTCTTTTAAATTAATTTTAGCTTTTTAACTAAAAAATAAATTTTTAATG
TTTAAATAGAGACAGAAATTTTTTTATCCAACCATTCATTCCAGCTTCTAATTAAG
AAACTAATGATTATGCTACCTTAGCACGGTCAAAATACCGCGGCCATTTAAATCC
TCAGAGGGCAGGCTA
MEX_SAG_5
CAGACCTAATATTCCCAGCTTCTGCACCAAAAATTAATTTTAATCCAACATCGAG
GTCGCAATCTTTATCTTCGATTAGAACTCTTTGATAATATTACGCTGTTATCCCTA
AGGTAATTAAATCTTACAATTATTACTAATAGCTCAAGTATACATAAATTAATGATT
TAAATTTAAAAAAAGTTCAATTAATTTTTCAATCACCCCAATTAAATAATTAAAAAA
ATTAAAAATTTTTATACTaAAAAAAAATTAAAATTTTTAAATATGAAACTCTATAGG
GTCTTCTCGTCTTTTAAATTAATTTTAGCTTTTTAACTAAAAAATAAATTTTTAATG
TTTAAATAGAGACAGAAATTTTTTTATCCAACCATTCATTCCAGCTTCTAATTAAG
AAACTAATGATTATGCTACCTTAGCACGGTCAAAATACCGCGGCCATTTAAATCC
TCAGAGGGCAGGCTA

MEX_SCA
CAGACCTAATATTCCCAGCTTCTGCACCAAAAATTAATTTTAATCCAACATCGAG
GTCGCAATCTTTATCTTCGATTAGAACTCTTTGATAATATTACGCTGTTATCCCTA
AGGTAATTAAATCTTACAATTATTACTAATAGCTCAAGTATACATAAATTAATGATT
TAAATTTAAAAAAAGTTCAATTAATTTTTCAATCACCCCAATTAAATAATTAAAAAA
ATTAAAAATTTTTATACTaAAAAAAAATTAAAATTTTTAAATATGAAACTCTATAGG
GTCTTCTCGTCTTTTAAATTAATTTTAGCTTTTTAACTAAAAAATAAATTTTTAATG
TTTAAATAGAGACAGAAATTTTTTTATCCAACCATTCATTCCAGCTTCTAATTAAG
AAACTAATGATTATGCTACCTTAGCACGGTCAAAATACCGCGGCCATTTAAATCC
TCAGAGGGCAGGCTA
MEX_SFP
CAGACCTAATATTCCCAGCTTCTGCACCAAAAATTAATTTTAATCCAACATCGAG
GTCGCAATCTTTATCTTCGATTAGAACTCTTTGATAATATTACGCTGTTATCCCTA
AGGTAATTAAATCTTACAATTATTACTAATAGCTCAAGTATACATAAATTAATGATT
TAAATTTAAAAAAAGTTCAATTAATTTTTCAATCACCCCAATTAAATAATTAAAAAA
ATTAAAAATTTTTATACTaAAAAAAAATTAAAATTTTTAAATATGAAACTCTATAGG
GTCTTCTCGTCTTTTAAATTAATTTTAGCTTTTTAACTAAAAAATAAATTTTTAATG
TTTAAATAGAGACAGAAATTTTTTTATCCAACCATTCATTCCAGCTTCTAATTAAG
AAACTAATGATTATGCTACCTTAGCACGGTCAAAATACCGCGGCCATTTAAATCC
TCAGAGGGCAGGCTA
MEX_SJS_2
CAGACCTAATATTCCCAGCTTCTGCACCAAAAATTAATTTTAATCCAACATCGAG
GTCGCAATCTTTATCTTCGATTAGAACTCTTTGATAATATTACGCTGTTATCCCTA
AGGTAATTAAATCTTACAATTATTACTAATAGCTCAAGTATACATAAATTAATGATT
TAAATTTAAAAAAAGTTCAATTAATTTTTCAATCACCCCAATTAAATAATTAAAAAA
ATTAAAAATTTTTATACTaAAAAAAAATTAAAATTTTTAAATATGAAACTCTATAGG
GTCTTCTCGTCTTTTAAATTAATTTTAGCTTTTTAACTAAAAAATAAATTTTTAATG
TTTAAATAGAGACAGAAATTTTTTTATCCAACCATTCATTCCAGCTTCTAATTAAG
AAACTAATGATTATGCTACCTTAGCACGGTCAAAATACCGCGGCCATTTAAATCC
TCAGAGGGCAGGCTA
MEX_STO
CAGACCTAATATTCCCAGCTTCTGCACCAAAAATTAATTTTAATCCAACATCGAG
GTCGCAATCTTTATCTTCGATTAGAACTCTTTGATAATATTACGCTGTTATCCCTA
AGGTAATTAAATCTTACAATTATTACTAATAGCTCAAGTATACATAAATTAATGATT
TAAATTTAAAAAAAGTTCAATTAATTTTTCAATCACCCCAATTAAATAATTAAAAAA
ATTAAAAATTTTTATACTaAAAAAAAATTAAAATTTTTAAATATGAAACTCTATAGG
GTCTTCTCGTCTTTTAAATTAATTTTAGCTTTTTAACTAAAAAATAAATTTTTAATG
TTTAAATAGAGACAGAAATTTTTTTATCCAACCATTCATTCCAGCTTCTAATTAAG
AAACTAATGATTATGCTACCTTAGCACGGTCAAAATACCGCGGCCATTTAAATCC
TCAGAGGGCAGGCTA
MEX_TLA_1
CAGACCTAATATTCCCAGCTTCTGCACCAAAAATTAATTTTAATCCAACATCGAG
GTCGCAATCTTTATCTTCGATTAGAACTCTTTGATAATATTACGCTGTTATCCCTA
AGGTAATTAAATCTTACAATTATTACTAATAGCTCAAGTATACATAAATTAATGATT
TAAATTTAAAAAAAGTTCAATTAATTTTTCAATCACCCCAATTAAATAATTAAAAAA
ATTAAAAATTTTTATACTaAAAAAAAATTAAAATTTTTAAATATGAAACTCTATAGG
GTCTTCTCGTCTTTTAAATTAATTTTAGCTTTTTAACTAAAAAATAAATTTTTAATG
TTTAAATAGAGACAGAAATTTTTTTATCCAACCATTCATTCCAGCTTCTAATTAAG
AAACTAATGATTATGCTACCTTAGCACGGTCAAAATACCGCGGCCATTTAAATCC
TCAGAGGGCAGGCTA

MEX_XOT_3
CAGACCTAATATTTCCAGCTTCTGCACCAAAAATTAATTTTAATCCAACATCGAG
GTCGCAATCTTTATCTTCGATTAGAACTCTTTGATAATATTACGCTGTTATCCCTA
AGGTAATTAAATCTTACAATTATTACTAATAGCTCAAGTATACATAAATTAATGATT
TAAATTTAAAAAAAGTTCAATTAATTTTTCAATCACCCCAATTAAATAATTAAAAAA
ATTAAAAATTTTTATACTaAAAAAAAATTAAAATTTTTAAATATGAAACTCTATAGG
GTCTTCTCGTCTTTTAAATTAATTTTAGCTTTTTAACTAAAAAATAAATTTTTAATG
TTTAAATAGAGACAGAAATTTTTTTATCCAACCATTCATTCCAGCTTCTAATTAAG
AAACTAATGATTATGCTACCTTAGCACGGTCAAAATACCGCGGCCATTTAAATCC
TCAGAGGGCAGGCTA
MEX_YOH
CAGACCTAATATCCCCAGCTTCTACACCAAAAATTAATTTTAATCCAACATCGAG
GTCGCAATCTTTATCTTCGATTAGAACTCTTTGATAATATTACGCTGTTATCCCTA
AGGTAATTAAATCTTACAATTATTACTAATAGCTCAAGTATACATAAATTAATGATT
TAAATTTAAAAAAAGTTCAATTAATTTTTCAATCACCCCAATTAAATAATTAAAAAA
ATTAAAAATTTTTATACTaAAAAAAAATTAAAATTTTTAAATATGAAACTCTATAGG
GTCTTCTCGTCTTTTAAATTAATTTTAGCTTTTTAACTAAAAAATAAATTTTTAATG
TTTAAATAGAGACAGAAATTTTTTTATCCAACCATTCATTCCAGCTTCTAATTAAG
AAACTAATGATTATGCTACCTTAGCACGGTCAAAATACCGCGGCCATTTAAATCC
TCAGAGGGCAGGCTA
moc5
CAGACCTAATATTTCCAGCTTCTGCACCAAAAATTAATTTTAATCCAACATCGAG
GTCGCAATCTTTATCTTCGATTAGAACTCTTTGATAATATTACGCTGTTATCCCTA
AGGTAATTAAATCTTACAATTATTACTAATAGCTCAAGTATACATAAATTAATGATT
TAAATTTAAAAAAAGTTCAATTAATTTTTCAATCACCCCAATTAAATAATTAAAAAA
ATTAAAAATTTTTATACTaAAAAAAAATTAAAATTTTTAAATATGAAACTCTATAGG
GTCTTCTCGTCTTTTAAATTAATTTTAGCTTTTTAACTAAAAAATAAATTTTTAATG
TTTAAATAGAGACAGAAATTTTTTTATCCAACCATTCATTCCAGCTTCTAATTAAG
AAACTAATGATTATGCTACCTTAGCACGGTCAAAATACCGCGGCCATTTAAATCC
TCAGAGGGCAGGCTA
PE2_1
CAGACCTAATATCCCCAGCTTCTACACCAAAAATTAATTTTAATCCAACATCGAG
GTCGCAATCTTTATCTTCGATTAGAACTCTTTGATAATATTACGCTGTTATCCCTA
AGGTAATTAAATCTTACAATTATTACTAATAGCTCAAGTATACATAAATTAATGATT
TAAATTTAAAAAAAGTTCAATTAATTTTTCAATCACCCCAATTAAATAATTAAAAAA
ATTAAAAATTTTTATACTAAAAAAAAATTAAAATTTTTAAATATGAAACTCTATAGG
GTCTTCTCGTCTTTTAAATTAATTTTAGCTTTTTAACTAAAAAATAAATTTTTAATG
TTTAAATAGAGACAGAAATTTTTTTATCCAACCATTCATTCCAGCTTCTAATTAAG
AAACTAATGATTATGCTACCTTAGCACGGTCAAAATACCGCGGCCATTTAAATCC
TCAGAGGGCAGGCTA
PE2_3
CAGACCTAATATCCCCAGCTTCTACACCAAAAATTAATTTTAATCCAACATCGAG
GTCGCAATCTTTATCTTCGATTAGAACTCTTTGATAATATTACGCTGTTATCCCTA
AGGTAATTAAATCTTACAATTATTACTAATAGCTCAAGTATACATAAATTAATGATT
TAAATTTAAAAAAAGTTCAATTAATTTTTCAATCACCCCAATTAAATAATTAAAAAA
ATTAAAAATTTTTATACTAAAAAAAAATTAAAATTTTTAAATATGAAACTCTATAGG
GTCTTCTCGTCTTTTAAATTAATTTTAGCTTTTTAACTAAAAAATAAATTTTTAATG
TTTAAATAGAGACAGAAATTTTTTTATCCAACCATTCATTCCAGCTTCTAATTAAG
AAACTAATGATTATGCTACCTTAGCACGGTCAAAATACCGCGGCCATTTAAATCC
TCAGAGGGCAGGCTA

PE2_4
CAGACCTAATATCCCCAGCTTCTACACCAAAAATTAATTTTAATCCAACATCGAG
GTCGCAATCTTTATCTTCGATTAGAACTCTTTGATAATATTACGCTGTTATCCCTA
AGGTAATTAAATCTTACAATTATTACTAATAGCTCAAGTATACATAAATTAATGATT
TAAATTTAAAAAAAGTTCAATTAATTTTTCAATCACCCCAATTAAATAATTAAAAAA
ATTAAAAATTTTTATACTAAAAAAAAAATTAAAATTTTTAAATATGAAACTCTATAGG
GTCTTCTCGTCTTTTAAATTAATTTTAGCTTTTTAACTAAAAAATAAATTTTTAATG
TTTAAATAGAGACAGAAATTTTTTTATCCAACCATTCATTCCAGCTTCTAATTAAG
AAACTAATGATTATGCTACCTTAGCACGGTCAAAATACCGCGGCCATTTAAATCC
TCAGAGGGCAGGCTA
PE2_5
CAGACCTAATATCCCCAGCTTCTACACCAAAAATTAATTTTAATCCAACATCGAG
GTCGCAATCTTTATCTTCGATTAGAACTCTTTGATAATATTACGCTGTTATCCCTA
AGGTAATTAAATCTTACAATTATTACTAATAGCTCAAGTATACATAAATTAATGATT
TAAATTTAAAAAAAGTTCAATTAATTTTTCAATCACCCCAATTAAATAATTAAAAAA
ATTAAAAATTTTTATACTAAAAAAAAAATTAAAATTTTTAAATATGAAACTCTATAGG
GTCTTCTCGTCTTTTAAATTAATTTTAGCTTTTTAACTAAAAAATAAATTTTTAATG
TTTAAATAGAGACAGAAATTTTTTTATCCAACCATTCATTCCAGCTTCTAATTAAG
AAACTAATGATTATGCTACCTTAGCACGGTCAAAATACCGCGGCCATTTAAATCC
TCAGAGGGCAGGCTA
PE2_6
CAGACCTAATATCCCCAGCTTCTACACCAAAAATTAATTTTAATCCAACATCGAG
GTCGCAATCTTTATCTTCGATTAGAACTCTTTGATAATATTACGCTGTTATCCCTA
AGGTAATTAAATCTTACAATTATTACTAATAGCTCAAGTATACATAAATTAATGATT
TAAATTTAAAAAAAGTTCAATTAATTTTTCAATCACCCCAATTAAATAATTAAAAAA
ATTAAAAATTTTTATACTAAAAAAAAAATTAAAATTTTTAAATATGAAACTCTATAGG
GTCTTCTCGTCTTTTAAATTAATTTTAGCTTTTTAACTAAAAAATAAATTTTTAATG
TTTAAATAGAGACAGAAATTTTTTTATCCAACCATTCATTCCAGCTTCTAATTAAG
AAACTAATGATTATGCTACCTTAGCACGGTCAAAATACCGCGGCCATTTAAATCC
TCAGAGGGCAGGCTA
PE2_7
CAGACCTAATATCCCCAGCTTCTACACCAAAAATTAATTTTAATCCAACATCGAG
GTCGCAATCTTTATCTTCGATTAGAACTCTTTGATAATATTACGCTGTTATCCCTA
AGGTAATTAAATCTTACAATTATTACTAATAGCTCAAGTATACATAAATTAATGATT
TAAATTTAAAAAAAGTTCAATTAATTTTTCAATCACCCCAATTAAATAATTAAAAAA
ATTAAAAATTTTTATACTAAAAAAAAAATTAAAATTTTTAAATATGAAACTCTATAGG
GTCTTCTCGTCTTTTAAATTAATTTTAGCTTTTTAACTAAAAAATAAATTTTTAATG
TTTAAATAGAGACAGAAATTTTTTTATCCAACCATTCATTCCAGCTTCTAATTAAG
AAACTAATGATTATGCTACCTTAGCACGGTCAAAATACCGCGGCCATTTAAATCC
TCAGAGGGCAGGCTA
PE_MAN_12
CAGACCTAATATCCCCAGCTTCTACACCAAAAATTAATTTTAATCCAACATCGAG
GTCGCAATCTTTATCTTCGATTAGAACTCTTTGATAATATTACGCTGTTATCCCTA
AGGTAATTAAATCTTACAATTATTACTAATAGCTCAAGTATACATAAATTAATGATT
TAAATTTAAAAAAAGTTCAATTAATTTTTCAATCACCCCAATTAAATAATTAAAAAA
ATTAAAAATTTTTATACTaAAAAAAAAATTAAAATTTTTAAATATGAAACTCTATAGG
GTCTTCTCGTCTTTTAAATTAATTTTAGCTTTTTAACTAAAAAATAAATTTTTAATG
TTTAAATAGAGACAGAAATTTTTTTATCCAACCATTCATTCCAGCTTCTAATTAAG
AAACTAATGATTATGCTACCTTAGCACGGTCAAAATACCGCGGCCATTTAAATCC
TCAGAGGGCAGGCTA

PE_MAN_13
CAGACCTAATATCCCCAGCTTCTACACCAAAAATTAATTTTAATCCAACATCGAG
GTCGCAATCTTTATCTTCGATTAGAACTCTTTGATAATATTACGCTGTTATCCCTA
AGGTAATTAAATCTTACAATTATTACTAATAGCTCAAGTATACATAAATTAATGATT
TAAATTTAAAAAAAGTTCAATTAATTTTTCAATCACCCCAATTAAATAATTAAAAAA
ATTAAAAATTTTTATACTaAAAAAAAATTAAAATTTTTAAATATGAAACTCTATAGG
GTCTTCTCGTCTTTTAAATTAATTTTAGCTTTTTAACTAAAAAATAAATTTTTAATG
TTTAAATAGAGACAGAAATTTTTTTATCCAACCATTCATTCCAGCTTCTAATTAAG
AAACTAATGATTATGCTACCTTAGCACGGTCAAAATACCGCGGCCATTTAAATCC
TCAGAGGGCAGGCTA
PE_MAN_15
CAGACCTAATATCCCCAGCTTCTACACCAAAAATTAATTTTAATCCAACATCGAG
GTCGCAATCTTTATCTTCGATTAGAACTCTTTGATAATATTACGCTGTTATCCCTA
AGGTAATTAAATCTTACAATTATTACTAATAGCTCAAGTATACATAAATTAATGATT
TAAATTTAAAAAAAGTTCAATTAATTTTTCAATCACCCCAATTAAATAATTAAAAAA
ATTAAAAATTTTTATACTaAAAAAAAATTAAAATTTTTAAATATGAAACTCTATAGG
GTCTTCTCGTCTTTTAAATTAATTTTAGCTTTTTAACTAAAAAATAAATTTTTAATG
TTTAAATAGAGACAGAAATTTTTTTATCCAACCATTCATTCCAGCTTCTAATTAAG
AAACTAATGATTATGCTACCTTAGCACGGTCAAAATACCGCGGCCATTTAAATCC
TCAGAGGGCAGGCTA
PE_MAN_16
CAGACCTAATATCCCCAGCTTCTACACCAAAAATTAATTTTAATCCAACATCGAG
GTCGCAATCTTTATCTTCGATTAGAACTCTTTGATAATATTACGCTGTTATCCCTA
AGGTAATTAAATCTTACAATTATTACTAATAGCTCAAGTATACATAAATTAATGATT
TAAATTTAAAAAAAGTTCAATTAATTTTTCAATCACCCCAATTAAATAATTAAAAAA
ATTAAAAATTTTTATACTaAAAAAAAATTAAAATTTTTAAATATGAAACTCTATAGG
GTCTTCTCGTCTTTTAAATTAATTTTAGCTTTTTAACTAAAAAATAAATTTTTAATG
TTTAAATAGAGACAGAAATTTTTTTATCCAACCATTCATTCCAGCTTCTAATTAAG
AAACTAATGATTATGCTACCTTAGCACGGTCAAAATACCGCGGCCATTTAAATCC
TCAGAGGGCAGGCTA
PE_MAN_19
CAGACCTAATATCCCCAGCTTCTACACCAAAAATTAATTTTAATCCAACATCGAG
GTCGCAATCTTTATCTTCGATTAGAACTCTTTGATAATATTACGCTGTTATCCCTA
AGGTAATTAAATCTTACAATTATTACTAATAGCTCAAGTATACATAAATTAATGATT
TAAATTTAAAAAAAGTTCAATTAATTTTTCAATCACCCCAATTAAATAATTAAAAAA
ATTAAAAATTTTTATACTaAAAAAAAATTAAAATTTTTAAATATGAAACTCTATAGG
GTCTTCTCGTCTTTTAAATTAATTTTAGCTTTTTAACTAAAAAATAAATTTTTAATG
TTTAAATAGAGACAGAAATTTTTTTATCCAACCATTCATTCCAGCTTCTAATTAAG
AAACTAATGATTATGCTACCTTAGCACGGTCAAAATACCGCGGCCATTTAAATCC
TCAGAGGGCAGGCTA
PE_MAN_2
CAGACCTAATATCCCCAGCTTCTACACCAAAAATTAATTTTAATCCAACATCGAG
GTCGCAATCTTTATCTTCGATTAGAACTCTTTGATAATATTACGCTGTTATCCCTA
AGGTAATTAAATCTTACAATTATTACTAATAGCTCAAGTATACATAAATTAATGATT
TAAATTTAAAAAAAGTTCAATTAATTTTTCAATCACCCCAATTAAATAATTAAAAAA
ATTAAAAATTTTTATACTaAAAAAAAATTAAAATTTTTAAATATGAAACTCTATAGG
GTCTTCTCGTCTTTTAAATTAATTTTAGCTTTTTAACTAAAAAATAAATTTTTAATG
TTTAAATAGAGACAGAAATTTTTTTATCCAACCATTCATTCCAGCTTCTAATTAAG
AAACTAATGATTATGCTACCTTAGCACGGTCAAAATACCGCGGCCATTTAAATCC
TCAGAGGGCAGGCTA

PE_MAN_23
CAGACCTAATATCCCCAGCTTCTACACCAAAAATTAATTTTAATCCAACATCGAG
GTCGCAATCTTTATCTTCGATTAGAACTCTTTGATAATATTACGCTGTTATCCCTA
AGGTAATTAAATCTTACAATTATTACTAATAGCTCAAGTATACATAAATTAATGATT
TAAATTTAAAAAAAGTTCAATTAATTTTTCAATCACCCCAATTAAATAATTAAAAAA
ATTAAAAATTTTTATACTaAAAAAAAATTAAAATTTTTAAATATGAAACTCTATAGG
GTCTTCTCGTCTTTTAAATTAATTTTAGCTTTTTAACTAAAAAATAAATTTTTAATG
TTTAAATAGAGACAGAAATTTTTTTATCCAACCATTCATTCCAGCTTCTAATTAAG
AAACTAATGATTATGCTACCTTAGCACGGTCAAAATACCGCGGCCATTTAAATCC
TCAGAGGGCAGGCTA
PE_MAN_29
CAGACCTAATATCCCCAGCTTCTACACCAAAAATTAATTTTAATCCAACATCGAG
GTCGCAATCTTTATCTTCGATTAGAACTCTTTGATAATATTACGCTGTTATCCCTA
AGGTAATTAAATCTTACAATTATTACTAATAGCTCAAGTATACATAAATTAATGATT
TAAATTTAAAAAAAGTTCAATTAATTTTTCAATCACCCCAATTAAATAATTAAAAAA
ATTAAAAATTTTTATACTaAAAAAAAATTAAAATTTTTAAATATGAAACTCTATAGG
GTCTTCTCGTCTTTTAAATTAATTTTAGCTTTTTAACTAAAAAATAAATTTTTAATG
TTTAAATAGAGACAGAAATTTTTTTATCCAACCATTCATTCCAGCTTCTAATTAAG
AAACTAATGATTATGCTACCTTAGCACGGTCAAAATACCGCGGCCATTTAAATCC
TCAGAGGGCAGGCTA
PE_MAN_6
CAGACCTAATATCCCCAGCTTCTACACCAAAAATTAATTTTAATCCAACATCGAG
GTCGCAATCTTTATCTTCGATTAGAACTCTTTGATAATATTACGCTGTTATCCCTA
AGGTAATTAAATCTTACAATTATTACTAATAGCTCAAGTATACATAAATTAATGATT
TAAATTTAAAAAAAGTTCAATTAATTTTTCAATCACCCCAATTAAATAATTAAAAAA
ATTAAAAATTTTTATACTaAAAAAAAATTAAAATTTTTAAATATGAAACTCTATAGG
GTCTTCTCGTCTTTTAAATTAATTTTAGCTTTTTAACTAAAAAATAAATTTTTAATG
TTTAAATAGAGACAGAAATTTTTTTATCCAACCATTCATTCCAGCTTCTAATTAAG
AAACTAATGATTATGCTACCTTAGCACGGTCAAAATACCGCGGCCATTTAAATCC
TCAGAGGGCAGGCTA
PE_MAN_8
CAGACCTAATATCCCCAGCTTCTACACCAAAAATTAATTTTAATCCAACATCGAG
GTCGCAATCTTTATCTTCGATTAGAACTCTTTGATAATATTACGCTGTTATCCCTA
AGGTAATTAAATCTTACAATTATTACTAATAGCTCAAGTATACATAAATTAATGATT
TAAATTTAAAAAAAGTTCAATTAATTTTTCAATCACCCCAATTAAATAATTAAAAAA
ATTAAAAATTTTTATACTaAAAAAAAATTAAAATTTTTAAATATGAAACTCTATAGG
GTCTTCTCGTCTTTTAAATTAATTTTAGCTTTTTAACTAAAAAATAAATTTTTAATG
TTTAAATAGAGACAGAAATTTTTTTATCCAACCATTCATTCCAGCTTCTAATTAAG
AAACTAATGATTATGCTACCTTAGCACGGTCAAAATACCGCGGCCATTTAAATCC
TCAGAGGGCAGGCTA
pit1
CAGACCTAATATTTCCAGCTTCTGCACCAAAAATTAATTTTAATCCAACATCGAG
GTCGCAATCTTTATCTTCGATTAGAACTCTTTGATAATATTACGCTGTTATCCCTA
AGGTAATTAAATCTTACAATTATTACTAATAGCTCAAGTATACATAAATTAATGATT
TAAATTTAAAAAAAGTTCGATTAATTTTTCAATCACCCCAATTAAATAATTAAAAAA
ATTAAAAATTTTTATACTaAAAAAAAATTAAAATTTTTAAATATGAAACTCTATAGG
GTCTTCTCGTCTTTTAAATTAATCTTAGCTTTTTAACTAAAAAATAAATTTTTAATG
TTTAAATAGAGACAGAAATTTTTTTATCCAACCATTCATTCCAGCTTCTAATTAAG
AAACTAATGATTATGCTACCTTAGCACGGTCAAAATACCGCGGCCATTTAAATCC
TCAGAGGGCAGGCTA

pit2
CAGACCTAATATTTCCAGCTTCTGCACCAAAAATTAATTTTAATCCAACATCGAG
GTCGCAATCTTTATCTTCGATTAGAACTCTTTGATAATATTACGCTGTTATCCCTA
AGGTAATTAAATCTTACAATTATTACTAATAGCTCAAGTATACATAAATTAATGATT
TAAATTTAAAAAAAGTTCGATTAATTTTTCAATCACCCCAATTAAATAATTAAAAAA
ATTAAAAATTTTTATACTaAAAAAAAATTAAAATTTTTAAATATGAAACTCTATAGG
GTCTTCTCGTCTTTTAAATTAATCTTAGCTTTTTAACTAAAAAATAAATTTTTAATG
TTTAAATAGAGACAGAAATTTTTTTATCCAACCATTCATTCCAGCTTCTAATTAAG
AAACTAATGATTATGCTACCTTAGCACGGTCAAAATACCGCGGCCATTTAAATCC
TCAGAGGGCAGGCTA
pit4
CAGACCTAATATTTCCAGCTTCTGCACCAAAAATTAATTTTAATCCAACATCGAG
GTCGCAATCTTTATCTTCGATTAGAACTCTTTGATAATATTACGCTGTTATCCCTA
AGGTAATTAAATCTTACAATTATTACTAATAGCTCAAGTATACATAAATTAATGATT
TAAATTTAAAAAAAGTTCGATTAATTTTTCAATCACCCCAATTAAATAATTAAAAAA
ATTAAAAATTTTTATACTaAAAAAAAATTAAAATTTTTAAATATGAAACTCTATAGG
GTCTTCTCGTCTTTTAAATTAATCTTAGCTTTTTAACTAAAAAATAAATTTTTAATG
TTTAAATAGAGACAGAAATTTTTTTATCCAACCATTCATTCCAGCTTCTAATTAAG
AAACTAATGATTATGCTACCTTAGCACGGTCAAAATACCGCGGCCATTTAAATCC
TCAGAGGGCAGGCTA
ron3
CAGACCTAATATTTCCAGCTTCTGCACCAAAAATTAATTTTAATCCAACATCGAG
GTCGCAATCTTTATCTTCGATTAGAACTCTTTGATAATATTACGCTGTTATCCCTA
AGGTAATTAAATCTTACAATTATTACTAATAGCTCAAGTATACATAAATTAATGATT
TAAATTTAAAAAAAGTTCAATTAATTTTTCAATCACCCCAATTAAATAATTAAAAAA
ATTAAAAATTTTTATACTaAAAAAAAATTAAAATTTTTAAATATGAAACTCTATAGG
GTCTTCTCGTCTTTTAAATTAATTTTAGCTTTTTAACTAAAAAATAAATTTTTAATG
TTTAAATAGAGACAGAAATTTTTTTATCCAACCATTCATTCCAGCTTCTAATTAAG
AAACTAATGATTATGCTACCTTAGCACGGTCAAAATACCGCGGCCATTTAAATCC
TCAGAGGGCAGGCTA
sob1
CAGACCTAATATTTCCAGCTTCTGCACCAAAAATTAATTTTAATCCAACATCGAG
GTCGCAATCTTTATCTTCGATTAGAACTCTTTGATAATATTACGCTGTTATCCCTA
AGGTAATTAAATCTTACAATTATTACTAATAGCTCAAGTATACATAAATTAATGATT
TAAATTTAAAAAAAGTTCGATTAATTTTTCAATCACCCCAATTAAATAATTAAAAAA
ATTAAAAATTTTTATACTaAAAAAAAATTAAAATTTTTAAATATGAAACTCTATAGG
GTCTTCTCGTCTTTTAAATTAATCTTAGCTTTTTAACTAAAAAATAAATTTTTAATG
TTTAAATAGAGACAGAAATTTTTTTATCCAACCATTCATTCCAGCTTCTAATTAAG
AAACTAATGATTATGCTACCTTAGCACGGTCAAAATACCGCGGCCATTTAAATCC
TCAGAGGGCAGGCTA
SPA_PRA_1
CAGACCTAATATTTCCAGCTTCTGCACCAAAAATTAATTTTAATCCAACATCGAG
GTCGCAATCTTTATCTTCGATTAGAACTCTTTGATAATATTACGCTGTTATCCCTA
AGGTAATTAAATCTTACAATTATTACTAATAGCTCAAGTATACATAAATTAATGATT
TAAATTTAAAAAAAGTTCAATTAATTTTTCAATCACCCCAATTAAATAATTAAAAAA
ATTAAAAATTTTTATACTaAAAAAAAATTAAAATTTTTAAATATGAAACTCTATAGG
GTCTTCTCGTCTTTTAAATTAATTTTAGCTTTTTAACTAAAAAATAAATTTTTAATG
TTTAAATAGAGACAGAAATTTTTTTATCCAACCATTCATTCCAGCTTCTAATTAAG
AAACTAATGATTATGCTACCTTAGCACGGTCAAAATACCGCGGCCATTTAAATCC
TCAGAGGGCAGGCTA

SWI_NEU_1
CAGACCTAATATTTCCAGCTTCTGCACCAAAAATTAATTTTAATCCAACATCGAG
GTCGCAATCTTTATCTTCGATTAGAACTCTTTGATAATATTACGCTGTTATCCCTA
AGGTAATTAAATCTTACAATTATTACTAATAGCTCAAGTATACATAAATTAATGATT
TAAATTTAAAAAAAGTTCGATTAATTTTTCAATCACCCCAATTAAATAATTAAAAAA
ATTAAAAATTTTTATACTaAAAAAAAATTAAAATTTTTAAATATGAAACTCTATAGG
GTCTTCTCGTCTTTTAAATTAATCTTAGCTTTTTAACTAAAAAATAAATTTTTAATG
TTTAAATAGAGACAGAAATTTTTTTATCCAACCATTCATTCCAGCTTCTAATTAAG
AAACTAATGATTATGCTACCTTAGCACGGTCAAAATACCGCGGCCATTTAAATCC
TCAGAGGGCAGGCTA
tol1
CAGACCTAATATTTCCAGCTTCTGCACCAAAAATTAATTTTAATCCAACATCGAG
GTCGCAATCTTTATCTTCGATTAGAACTCTTTGATAATATTACGCTGTTATCCCTA
AGGTAATTAAATCTTACAATTATTACTAATAGCTCAAGTATACATAAATTAATGATT
TAAATTTAAAAAAAGTTCAATTAATTTTTCAATCACCCCAATTAAATAATTAAAAAA
ATTAAAAATTTTTATACTaAAAAAAAATTAAAATTTTTAAATATGAAACTCTATAGG
GTCTTCTCGTCTTTTAAATTAATTTTAGCTTTTTAACTAAAAAATAAATTTTTAATG
TTTAAATAGAGACAGAAATTTTTTTATCCAACCATTCATTCCAGCTTCTAATTAAG
AAACTAATGATTATGCTACCTTAGCACGGTCAAAATACCGCGGCCATTTAAATCC
TCAGAGGGCAGGCTA
;

endblock;

datasetS3

#NEXUS

begin data;
dimensions ntax=191 nchar=736;
format datatype=dna gap=- interleave=no;
matrix

AFR10
ATTATTAGACAAGAAAGGGGGAAAAAAGAAGCTTTTGGAACCTTAGGAATAATTT
ATGCCATGATAGCTATCGGATTATTAGGATTCGTAGTTTGAGCTCACCATATATT
TACAGTAGGGATAGATGTAGATACCCGAGCTTACTTCACTTCAGCAACTATAATT
ATTGCGGTTCCAACTGGGATTAAAGTCTTTAGATGACTAGCCACCTTTCATGGAA
CTCAAATTTTAAATAGACCAGTAACCCTGTGAGCTTTAGGTTTTGTATTTTTATTT
ACAGTAGGAGGATTAACAGGGGTAATTTTAGCTAATTCTTCAATTGATATTATTCT
TCACGATACTTACTATGTAGTAGCTCATTTTCACTATGTTCTTTCTATAGGAGCTG
TATTTGCAATTATAGCCGGAATTGTCCAATGATTCCCATTATTTACCGGACTAAC
CTTAAATAATTTTTTTTTAAAAACCCAATTTATTACTATATTTTTAGGTGTAAATTTA
ACATTTTTTCCCCAACACTTTTTAGGGCTAAGAGGAATGCCTCGGCGATATTCGG
ATTATCCAGATGCTTTCACTATCTGAAATGCCATTTCATCTATTGGTTCCATTATT
TCTCTCACAAGAATTATCTTTTTTTTATTTATCTTATGAGAAAGGCTTTCTTCCCAA
CGTAAATCTTTGAGAACTTTAAATGTAACAACTTCAATTGAATGGTTACAAGCCCT
ACCCCCTAGTG
AFR11
ATTATTAGACAAGAAAGGGGGAAAAAAGAAGCTTTTGGAACCTTAGGAATAATTT
ATGCCATGATAGCTATCGGATTATTAGGATTCGTAGTTTGAGCTCACCATATATT
TACAGTAGGGATAGATGTAGATACCCGAGCTTACTTCACTTCAGCAACTATAATT
ATTGCGGTTCCAACTGGGATTAAAGTCTTTAGATGACTAGCCACCTTTCATGGAA
CTCAAATTTTAAATAGACCAGTAACCCTGTGAGCTTTAGGTTTTGTATTTTTATTT
ACAGTAGGAGGATTAACAGGGGTAATTTTAGCTAATTCTTCAATTGATATTATTCT
TCACGATACTTACTATGTAGTAGCTCATTTTCACTATGTTCTTTCTATAGGAGCTG
TATTTGCAATTATAGCCGGAATTGTCCAATGATTCCCATTATTTACCGGACTAAC
CTTAAATAATTTTTTTTTAAAAACCCAATTTATTACTATATTTTTAGGTGTAAATTTA
ACATTTTTTCCCCAACACTTTTTAGGGCTAAGAGGAATGCCTCGGCGATATTCGG
ATTATCCAGATGCTTTCACTATCTGAAATGCCATTTCATCTATTGGTTCCATTATT
TCTCTCACAAGAATTATCTTTTTTTTATTTATCTTATGAGAAAGGCTTTCTTCCCAA
CGTAAATCTTTGAGAACTTTAAATGTAACAACTTCAATTGAATGGTTACAAGCCCT
ACCCCCTAGTG
AFR12
ATTATTAGACAAGAAAGGGGGAAAAAAGAAGCTTTTGGAACCTTAGGAATAATTT
ATGCCATGATAGCTATCGGATTATTAGGATTCGTAGTTTGAGCTCACCATATATT
TACAGTAGGGATAGATGTAGATACCCGAGCTTACTTCACTTCAGCAACTATAATT
ATTGCGGTTCCAACTGGGATTAAAGTCTTTAGATGACTAGCCACCTTTCATGGAA
CTCAAATTTTAAATAGACCAGTAACCCTGTGAGCTTTAGGTTTTGTATTTTTATTT
ACAGTAGGAGGATTAACAGGGGTAATTTTAGCTAATTCTTCAATTGATATTATTCT
TCACGATACTTACTATGTAGTAGCTCATTTTCACTATGTTCTTTCTATAGGAGCTG
TATTTGCAATTATAGCCGGAATTGTCCAATGATTCCCATTATTTACCGGACTAAC
CTTAAATAATTTTTTTTTAAAAACCCAATTTATTACTATATTTTTAGGTGTAAATTTA
ACATTTTTTCCCCAACACTTTTTAGGGCTAAGAGGAATGCCTCGGCGATATTCGG
ATTATCCAGATGCTTTCACTATCTGAAATGCCATTTCATCTATTGGTTCCATTATT

TCTCTCACAAGAATTATCTTTTTTTTATTTATCTTATGAGAAAGGCTTTCTTCCCAA
CGTAAATCTTTGAGAACTTTAAATGTAACAACTTCAATTGAATGGTTACAAGCCCT
ACCCCCTAGTG
AFR18
ATTATTAGACAAGAAAGGGGGAAAAAAGAAGCTTTTGGAACCTTAGGAATAATTT
ATGCCATGATAGCTATCGGATTATTAGGATTCGTAGTTTGAGCTCACCATATATT
TACAGTAGGGATAGATGTAGATACCCGAGCTTACTTCACTTCAGCAACTATAATT
ATTGCGGTTCCAACTGGGATTAAAGTCTTTAGATGACTAGCCACCTTTCATGGAA
CTCAAATTTTAAATAGACCAGTAACCCTGTGAGCTTTAGGTTTTGTATTTTTATTT
ACAGTAGGAGGATTAACAGGGGTAATTTTAGCTAATTCTTCAATTGATATTATTCT
TCACGATACTTACTATGTAGTAGCTCATTTTCACTATGTTCTTTCTATAGGAGCTG
TATTTGCAATTATAGCCGGAATTGTCCAATGATTCCCATTATTTACCGGACTAAC
CTTAAATAATTTTTTTTTAAAAACCCAATTTATTACTATATTTTTAGGTGTAAATTTA
ACATTTTTTCCCCAACACTTTTTAGGGCTAAGAGGAATGCCTCGGCGATATTCGG
ATTATCCAGATGCTTTCACTATCTGAAATGCCATTTCATCTATTGGTTCCATTATT
TCTCTCACAAGAATTATCTTTTTTTTATTTATCTTATGAGAAAGGCTTTCTTCCCAA
CGTAAATCTTTGAGAACTTTAAATGTAACAACTTCAATTGAATGGTTACAAGCCCT
ACCCCCTAGTG
afr19
ATTATTAGACAAGAAAGGGGGAAAAAAGAAGCTTTTGGAACCTTAGGAATAATTT
ATGCCATGATAGCTATCGGATTATTAGGATTCGTAGTTTGAGCTCACCATATATT
TACAGTAGGGATAGATGTAGATACCCGAGCTTACTTCACTTCAGCAACTATAATT
ATTGCGGTTCCAACTGGGATTAAAGTCTTTAGATGACTAGCCACCTTTCATGGAA
CTCAAATTTTAAATAGACCAGTAACCCTGTGAGCTTTAGGTTTTGTATTTTTATTT
ACAGTAGGAGGATTAACAGGGGTAATTTTAGCTAATTCTTCAATTGATATTATTCT
TCACGATACTTACTATGTAGTAGCTCATTTTCACTATGTTCTTTCTATAGGAGCTG
TATTTGCAATTATAGCCGGAATTGTCCAATGATTCCCATTATTTACCGGACTAAC
CTTAAATAATTTTTTTTTAAAAACCCAATTTATTACTATATTTTTAGGTGTAAATTTA
ACATTTTTTCCCCAACACTTTTTAGGGCTAAGAGGAATGCCTCGGCGATATTCGG
ATTATCCAGATGCTTTCACTATCTGAAATGCCATTTCATCTATTGGTTCCATTATT
TCTCTCACAAGAATTATCTTTTTTTTATTTATCTTATGAGAAAGGCTTTCTTCCCAA
CGTAAATCTTTGAGAACTTTAAATGTAACAACTTCAATTGAATGGTTACAAGCCCT
ACCCCCTAGTG
AFR2
ATTATTAGACAAGAAAGGGGGAAAAAAGAAGCTTTTGGAACCTTAGGAATAATTT
ATGCCATGATAGCTATCGGATTATTAGGATTCGTAGTTTGAGCTCACCATATATT
TACAGTAGGGATAGATGTAGATACCCGAGCTTACTTCACTTCAGCAACTATAATT
ATTGCGGTTCCAACTGGGATTAAAGTCTTTAGATGACTAGCCACCTTTCATGGAA
CTCAAATTTTAAATAGACCAGTAACCCTGTGAGCTTTAGGTTTTGTATTTTTATTT
ACAGTAGGAGGATTAACAGGGGTAATTTTAGCTAATTCTTCAATTGATATTATTCT
TCACGATACTTACTATGTAGTAGCTCATTTTCACTATGTTCTTTCTATAGGAGCTG
TATTTGCAATTATAGCCGGAATTGTCCAATGATTCCCATTATTTACCGGACTAAC
CTTAAATAATTTTTTTTTAAAAACCCAATTTATTACTATATTTTTAGGTGTAAATTTA
ACATTTTTTCCCCAACACTTTTTAGGGCTAAGAGGAATGCCTCGGCGATATTCGG
ATTATCCAGATGCTTTCACTATCTGAAATGCCATTTCATCTATTGGTTCCATTATT
TCTCTCACAAGAATTATCTTTTTTTTATTTATCTTATGAGAAAGGCTTTCTTCCCAA
CGTAAATCTTTGAGAACTTTAAATGTAACAACTTCAATTGAATGGTTACAAGCCCT
ACCCCCTAGTG
AFR3
ATTATTAGACAAGAAAGGGGGAAAAAAGAAGCTTTTGGAACCTTAGGAATAATTT

ATGCCATGATAGCTATCGGATTATTAGGATTCGTAGTTTGAGCTCACCATATATT
TACAGTAGGGATAGATGTAGATACCCGAGCTTACTTCACTTCAGCAACTATAATT
ATTGCGGTTCCAACTGGGATTAAAGTCTTTAGATGACTAGCCACCTTTCATGGAA
CTCAAATTTTAAATAGACCAGTAACCCTGTGAGCTTTAGGTTTTGTATTTTTATTT
ACAGTAGGAGGATTAACAGGGGTAATTTTAGCTAATTCTTCAATTGATATTATTCT
TCACGATACTTACTATGTAGTAGCTCATTTTCACTATGTTCTTTCTATAGGAGCTG
TATTTGCAATTATAGCCGGAATTGTCCAATGATTCCCATTATTTACCGGACTAAC
CTTAAATAATTTTTTTTTAAAAACCCAATTTATTACTATATTTTTAGGTGTAAATTTA
ACATTTTTTCCCCAACACTTTTTAGGGCTAAGAGGAATGCCTCGGCGATATTCGG
ATTATCCAGATGCTTTCACTATCTGAAATGCCATTTCATCTATTGGTTCCATTATT
TCTCTCACAAGAATTATCTTTTTTTTATTTATCTTATGAGAAAGGCTTTCTTCCCAA
CGTAAATCTTTGAGAACTTTAAATGTAACAACTTCAATTGAATGGTTACAAGCCCT
ACCCCCTAGTG
AFR5
ATTATTAGACAAGAAAGGGGGAAAAAAGAAGCTTTTGGAACCTTAGGAATAATTT
ATGCCATGATAGCTATCGGATTATTAGGATTCGTAGTTTGAGCTCACCATATATT
TACAGTAGGGATAGATGTAGATACCCGAGCTTACTTCACTTCAGCAACTATAATT
ATTGCGGTTCCAACTGGGATTAAAGTCTTTAGATGACTAGCCACCTTTCATGGAA
CTCAAATTTTAAATAGACCAGTAACCCTGTGAGCTTTAGGTTTTGTATTTTTATTT
ACAGTAGGAGGATTAACAGGGGTAATTTTAGCTAATTCTTCAATTGATATTATTCT
TCACGATACTTACTATGTAGTAGCTCATTTTCACTATGTTCTTTCTATAGGAGCTG
TATTTGCAATTATAGCCGGAATTGTCCAATGATTCCCATTATTTACCGGACTAAC
CTTAAATAATTTTTTTTTAAAAACCCAATTTATTACTATATTTTTAGGTGTAAATTTA
ACATTTTTTCCCCAACACTTTTTAGGGCTAAGAGGAATGCCTCGGCGATATTCGG
ATTATCCAGATGCTTTCACTATCTGAAATGCCATTTCATCTATTGGTTCCATTATT
TCTCTCACAAGAATTATCTTTTTTTTATTTATCTTATGAGAAAGGCTTTCTTCCCAA
CGTAAATCTTTGAGAACTTTAAATGTAACAACTTCAATTGAATGGTTACAAGCCCT
ACCCCCTAGTG
afr9
ATTATTAGACAAGAAAGGGGGAAAAAAGAAGCTTTTGGAACCTTAGGAATAATTT
ATGCCATGATAGCTATCGGATTATTAGGATTCGTAGTTTGAGCTCACCATATATT
TACAGTAGGGATAGATGTAGATACCCGAGCTTACTTCACTTCAGCAACTATAATT
ATCGCGGTTCCAACTGGGATTAAAGTCTTTAGGTGACTAGCCACCTTTCATGGA
ACTCAAATTTTAAATAGACCAGTAACCCTGTGAGCTTTAGGTTTTGTATTTTTATT
TACAGTAGGAGGATTAACAGGGGTAATTTTAGCTAATTCTTCAATTGATATTATTC
TTCACGATACTTACTATGTAGTAGCTCATTTTCACTATGTTCTTTCTATAGGAGCT
GTATTTGCAATTATAGCCGGAATTGTCCAATGATTCCCATTATTTACTGGACTAA
CCTTAAATAATTTTTTTTTAAAAACCCAATTTATTACTATATTTTTAGGTGTAAATTT
AACATTTTTTCCTCAACACTTTTTAGGGCTAAGAGGAATGCCTCGGCGATATTCG
GATTATCCAGATGCTTTCACTATCTGAAATGCCATTTCATCTATTGGTTCCATTAT
TTCTCTCACAAGAATTATCTTTTTTTTATTTATCTTATGAGAAAGGCTTTCTTCCCA
ACGTAAATCTTTGAGAACTTTAAATGTAACAACTTCAATTGAATGGTTACAAGCC
CTACCCCCTAGTG
AQU10
ATTATTAGACAAGAAAGGGGGAAAAAAGAAGCTTTTGGAACCTTAGGAATAATTT
ATGCCATGATAGCTATCGGATTATTAGGATTCGTAGTTTGAGCTCACCATATATT
TACAGTAGGGATAGATGTAGATACCCGAGCTTACTTCACTTCAGCAACTATAATT
ATTGCGGTTCCAACTGGGATTAAAGTCTTTAGATGACTAGCCACCTTTCATGGAA
CTCAAATTTTAAATAGACCAGTAACCCTGTGAGCTTTAGGTTTTGTATTTTTATTT
ACAGTAGGAGGATTAACAGGGGTAATTTTAGCTAATTCTTCAATTGATATTATTCT

TCACGATACTTACTATGTAGTAGCTCATTTTCACTATGTTCTTTCTATAGGAGCTG
TATTTGCAATTATAGCCGGAATTGTCCAATGATTCCCATTATTTACCGGACTAAC
CTTAAATAATTTTTTTTTAAAAACCCAATTTATTACTATATTTTTAGGTGTAAATTTA
ACATTTTTTCCCCAACACTTTTTAGGGCTAAGAGGAATGCCTCGGCGATATTCGG
ATTATCCAGATGCTTTCACTATCTGAAATGCCATTTCATCTATTGGTTCCATTATT
TCTCTCACAAGAATTATCTTTTTTTTATTTATCTTATGAGAAAGGCTTTCTTCCCAA
CGTAAATCTTTGAGAACTTTAAATGTAACAACTTCAATTGAATGGTTACAAGCCCT
ACCCCCTAGTG
AQU12
ATTATTAGACAAGAAAGGGGGAAAAAAGAAGCTTTTGGAACCTTAGGAATAATTT
ATGCCATGATAGCTATCGGATTATTAGGATTCGTAGTCTGAGCTCACCATATATT
TACAGTAGGGATAGATGTAGATACCCGAGCTTACTTCACTTCAGCAACTATAATT
ATTGCGGTTCCAACTGGGATTAAAGTCTTTAGATGACTAGCCACCTTTCATGGAA
CTCAAATTTTAAATAGACCAGTAACCCTGTGAGCTTTAGGTTTTGTATTTTTATTT
ACAGTAGGAGGATTAACAGGGGTAATTTTAGCTAATTCTTCAATTGATATTATTCT
TCACGATACTTACTATGTAGTAGCTCATTTTCACTATGTTCTTTCTATAGGAGCTG
TATTTGCAATTATAGCCGGAATTGTCCAATGATTCCCATTATTTACCGGACTAAC
CTTAAATAATTTTTTTTTAAAAACCCAATTTATTACTATATTTTTAGGTGTAAATTTA
ACATTTTTTCCCCAACACTTTTTAGGGCTAAGAGGAATGCCTCGGCGATATTCGG
ATTATCCAGATGCTTTCACTATCTGAAATGCCATTTCATCTATTGGTTCCATTATT
TCTCTCACAAGAATTATCTTTTTTTTATTTATCTTATGAGAAAGGCTTTCTTCCCAA
CGTAAATCTTTGAGAACTTTAAATGTAACAACTTCAATTGAATGGTTACAAGCCCT
ACCCCCTAGTG
AQU13
ATTATTAGACAAGAAAGGGGGAAAAAAGAAGCTTTTGGAACCTTAGGAATAATTT
ATGCCATGATAGCTATCGGATTATTAGGATTCGTAGTTTGAGCTCACCATATATT
TACAGTAGGGATAGATGTAGATACCCGAGCTTACTTCACTTCAGCAACTATAATT
ATTGCGGTTCCAACTGGGATTAAAGTCTTTAGATGACTAGCCACCTTTCATGGAA
CTCAAATTTTAAATAGACCAGTAACCCTGTGAGCTTTAGGTTTTGTATTTTTATTT
ACAGTAGGAGGATTAACAGGGGTAATTTTAGCTAATTCTTCAATTGATATTATTCT
TCACGATACTTACTATGTAGTAGCTCATTTTCACTATGTTCTTTCTATAGGAGCTG
TATTTGCAATTATAGCCGGAATTGTCCAATGATTCCCATTATTTACCGGACTAAC
CTTAAATAATTTTTTTTTAAAAACCCAATTTATTACTATATTTTTAGGTGTAAATTTA
ACATTTTTTCCCCAACACTTTTTAGGGCTAAGAGGAATGCCTCGGCGATATTCGG
ATTATCCAGATGCTTTCACTATCTGAAATGCCATTTCATCTATTGGTTCCATTATT
TCTCTCACAAGAATTATCTTTTTTTTATTTATCTTATGAGAAAGGCTTTCTTCCCAA
CGTAAATCTTTGAGAACTTTAAATGTAACAACTTCAATTGAATGGTTACAAGCCCT
ACCCCCTAGTG
AQU14
ATTATTAGACAAGAAAGGGGGAAAAAAGAAGCTTTTGGAACCTTAGGAATAATTT
ATGCCATGATAGCTATCGGATTATTAGGATTCGTAGTTTGAGCTCACCATATATT
TACAGTAGGGATAGATGTAGATACCCGAGCTTACTTCACTTCAGCAACTATAATT
ATTGCGGTTCCAACTGGGATTAAAGTCTTTAGATGACTAGCCACCTTTCATGGAA
CTCAAATTTTAAATAGACCAGTAACCCTGTGAGCTTTAGGTTTTGTATTTTTATTT
ACAGTAGGAGGATTAACAGGGGTAATTTTAGCTAATTCTTCAATTGATATTATTCT
TCACGATACTTACTATGTAGTAGCTCATTTTCACTATGTTCTTTCTATAGGAGCTG
TATTTGCAATTATAGCCGGAATTGTCCAATGATTCCCATTATTTACCGGACTAAC
CTTAAATAATTTTTTTTTAAAAACCCAATTTATTACTATATTTTTAGGTGTAAATTTA
ACATTTTTTCCCCAACACTTTTTAGGGCTAAGAGGAATGCCTCGGCGATATTCGG
ATTATCCAGATGCTTTCACTATCTGAAATGCCATTTCATCTATTGGTTCCATTATT

TCTCTCACAAGAATTATCTTTTTTTTATTTATCTTATGAGAAAGGCTTTCTTCCCAA
CGTAAATCTTTGAGAACTTTAAATGTAACAACTTCAATTGAATGGTTACAAGCCCT
ACCCCCTAGTG
AQU4
ATTATTAGACAAGAAAGGGGGAAAAAAGAAGCTTTTGGAACCTTAGGAATAATTT
ATGCCATGATAGCTATCGGATTATTAGGATTCGTAGTCTGAGCTCACCATATATT
TACAGTAGGGATAGATGTAGATACCCGAGCTTACTTCACTTCAGCAACTATAATT
ATTGCGGTTCCAACTGGGATTAAAGTCTTTAGATGACTAGCCACCTTTCATGGAA
CTCAAATTTTAAATAGACCAGTAACCCTGTGAGCTTTAGGTTTTGTATTTTTATTT
ACAGTAGGAGGATTAACAGGGGTAATTTTAGCTAATTCTTCAATTGATATTATTCT
TCACGATACTTACTATGTAGTAGCTCATTTTCACTATGTTCTTTCTATAGGAGCTG
TATTTGCAATTATAGCCGGAATTGTCCAATGATTCCCATTATTTACCGGACTAAC
CTTAAATAATTTTTTTTTAAAAACCCAATTTATTACTATATTTTTAGGTGTAAATTTA
ACATTTTTTCCCCAACACTTTTTAGGGCTAAGAGGAATGCCTCGGCGATATTCGG
ATTATCCAGATGCTTTCACTATCTGAAATGCCATTTCATCTATTGGTTCCATTATT
TCTCTCACAAGAATTATCTTTTTTTTATTTATCTTATGAGAAAGGCTTTCTTCCCAA
CGTAAATCTTTGAGAACTTTAAATGTAACAACTTCAATTGAATGGTTACAAGCCCT
ACCCCCTAGTG
bja1
ATTATTAGACAAGAAAGGGGGAAAAAAGAAGCTTTTGGAACCTTAGGAATAATTT
ATGCCATGATAGCTATCGGATTATTAGGATTCGTAGTTTGAGCTCACCATATATT
TACAGTAGGGATAGATGTAGATACCCGAGCTTACTTCACTTCAGCAACTATAATT
ATTGCGGTTCCAACTGGGATTAAAGTCTTTAGATGACTAGCCACCTTTCATGGAA
CTCAAATTTTAAATAGACCAGTAACCCTGTGAGCTTTAGGTTTTGTATTTTTATTT
ACAGTAGGAGGATTAACAGGGGTAATTTTAGCTAATTCTTCAATTGATATTATTCT
TCACGATACTTACTATGTAGTAGCTCATTTTCACTATGTTCTTTCTATAGGAGCTG
TATTTGCAATTATAGCCGGAATTGTCCAATGATTCCCATTATTTACCGGACTAAC
CTTAAATAATTTTTTTTTAAAAACCCAATTTATTACTATATTTTTAGGTGTAAATTTA
ACATTTTTTCCCCAACACTTTTTAGGGCTAAGAGGAATGCCTCGGCGATATTCGG
ATTATCCAGATGCTTTCACTATCTGAAATGCCATTTCATCTATTGGTTCCATTATT
TCTCTCACAAGAATTATCTTTTTTTTATTTATCTTATGAGAAAGGCTTTCTTCCCAA
CGTAAATCTTTGAGAACTTTAAATGTAACAACTTCAATTGAATGGTTACAAGCCCT
ACCCCCTAGTG
BJA2
ATTATTAGACAAGAAAGGGGGAAAAAAGAAGCTTTTGGAACCTTAGGAATAATTT
ATGCCATGATAGCTATCGGATTATTAGGATTCGTAGTTTGAGCTCACCATATATT
TACAGTAGGGATAGATGTAGATACCCGAGCTTACTTCACTTCAGCAACTATAATT
ATTGCGGTTCCAACTGGGATTAAAGTCTTTAGATGACTAGCCACCTTTCATGGAA
CTCAAATTTTAAATAGACCAGTAACCCTGTGAGCTTTAGGTTTTGTATTTTTATTT
ACAGTAGGAGGATTAACAGGGGTAATTTTAGCTAATTCTTCAATTGATATTATTCT
TCACGATACTTACTATGTAGTAGCTCATTTTCACTATGTTCTTTCTATAGGAGCTG
TATTTGCAATTATAGCCGGAATTGTCCAATGATTCCCATTATTTACCGGACTAAC
CTTAAATAATTTTTTTTTAAAAACCCAATTTATTACTATATTTTTAGGTGTAAATTTA
ACATTTTTTCCCCAACACTTTTTAGGGCTAAGAGGAATGCCTCGGCGATATTCGG
ATTATCCAGATGCTTTCACTATCTGAAATGCCATTTCATCTATTGGTTCCATTATT
TCTCTCACAAGAATTATCTTTTTTTTATTTATCTTATGAGAAAGGCTTTCTTCCCAA
CGTAAATCTTTGAGAACTTTAAATGTAACAACTTCAATTGAATGGTTACAAGCCCT
ACCCCCTAGTG
BJA4
ATTATTAGACAAGAAAGGGGGAAAAAAGAAGCTTTTGGAACCTTAGGAATAATTT

ATGCCATGATAGCTATCGGATTATTAGGATTCGTAGTTTGAGCTCACCATATATT
TACAGTAGGGATAGATGTAGATACCCGAGCTTACTTCACTTCAGCAACTATAATT
ATTGCGGTTCCAACTGGGATTAAAGTCTTTAGATGACTAGCCACCTTTCATGGAA
CTCAAATTTTAAATAGACCAGTAACCCTGTGAGCTTTAGGTTTTGTATTTTTATTT
ACAGTAGGAGGATTAACAGGGGTAATTTTAGCTAATTCTTCAATTGATATTATTCT
TCACGATACTTACTATGTAGTAGCTCATTTTCACTATGTTCTTTCTATAGGAGCTG
TATTTGCAATTATAGCCGGAATTGTCCAATGATTCCCATTATTTACCGGACTAAC
CTTAAATAATTTTTTTTTAAAAACCCAATTTATTACTATATTTTTAGGTGTAAATTTA
ACATTTTTTCCCCAACACTTTTTAGGGCTAAGAGGAATGCCTCGGCGATATTCGG
ATTATCCAGATGCTTTCACTATCTGAAATGCCATTTCATCTATTGGTTCCATTATT
TCTCTCACAAGAATTATCTTTTTTTTATTTATCTTATGAGAAAGGCTTTCTTCCCAA
CGTAAATCTTTGAGAACTTTAAATGTAACAACTTCAATTGAATGGTTACAAGCCCT
ACCCCCTAGTG
CAM1
ATTATTAGACAAGAAAGGGGGAAAAAAGAAGCTTTTGGAACCTTAGGAATAATTT
ATGCCATGATAGCTATCGGATTATTAGGATTCGTAGTTTGAGCTCACCATATATT
TACAGTAGGGATAGATGTAGATACCCGAGCTTACTTCACTTCAGCAACTATAATT
ATTGCGGTTCCAACTGGGATTAAAGTCTTTAGATGACTAGCCACCTTTCATGGAA
CTCAAATTTTAAATAGACCAGTAACCCTGTGAGCTTTAGGTTTTGTATTTTTATTT
ACAGTAGGAGGATTAACAGGGGTAATTTTAGCTAATTCTTCAATTGATATTATTCT
TCACGATACTTACTATGTAGTAGCTCATTTTCACTATGTTCTTTCTATAGGAGCTG
TATTTGCAATTATAGCCGGAATTGTCCAATGATTCCCATTATTTACCGGACTAAC
CTTAAATAATTTTTTTTTAAAAACCCAATTTATTACTATATTTTTAGGTGTAAATTTA
ACATTTTTTCCCCAACACTTTTTAGGGCTAAGAGGAATGCCTCGGCGATATTCGG
ATTATCCAGATGCTTTCACTATCTGAAATGCCATTTCATCTATTGGTTCCATTATT
TCTCTCACAAGAATTATCTTTTTTTTATTTATCTTATGAGAAAGGCTTTCTTCCCAA
CGTAAATCTTTGAGAACTTTAAATGTAACAACTTCAATTGAATGGTTACAAGCCCT
ACCCCCTAGTG
CAM2
ATTATTAGACAAGAAAGGGGGAAAAAAGAAGCTTTTGGAACCTTAGGAATAATTT
ATGCCATGATAGCTATCGGATTATTAGGATTCGTAGTTTGAGCTCACCATATATT
TACAGTAGGGATAGATGTAGATACCCGAGCTTACTTCACTTCAGCAACTATAATT
ATTGCGGTTCCAACTGGGATTAAAGTCTTTAGATGACTAGCCACCTTTCATGGAA
CTCAAATTTTAAATAGACCAGTAACCCTGTGAGCTTTAGGTTTTGTATTTTTATTT
ACAGTAGGAGGATTAACAGGGGTAATTTTAGCTAATTCTTCAATTGATATTATTCT
TCACGATACTTACTATGTAGTAGCTCATTTTCACTATGTTCTTTCTATAGGAGCTG
TATTTGCAATTATAGCCGGAATTGTCCAATGATTCCCATTATTTACCGGACTAAC
CTTAAATAATTTTTTTTTAAAAACCCAATTTATTACTATATTTTTAGGTGTAAATTTA
ACATTTTTTCCCCAACACTTTTTAGGGCTAAGAGGAATGCCTCGGCGATATTCGG
ATTATCCAGATGCTTTCACTATCTGAAATGCCATTTCATCTATTGGTTCCATTATT
TCTCTCACAAGAATTATCTTTTTTTTATTTATCTTATGAGAAAGGCTTTCTTCCCAA
CGTAAATCTTTGAGAACTTTAAATGTAACAACTTCAATTGAATGGTTACAAGCCCT
ACCCCCTAGTG
CMO1_1
ATTATTAGACAAGAAAGGGGGAAAAAAGAAGCTTTTGGAACCTTAGGAATAATTT
ATGCCATGATAGCTATCGGATTATTAGGATTCGTAGTTTGAGCTCACCATATATT
TACAGTAGGGATAGATGTAGATACCCGAGCTTACTTCACTTCAGCAACTATAATT
ATTGCGGTTCCAACTGGGATTAAAGTCTTTAGATGACTAGCCACCTTTCATGGAA
CTCAAATTTTAAATAGACCAGTAACCCTGTGAGCTTTAGGTTTTGTATTTTTATTT
ACAGTAGGAGGATTAACAGGGGTAATTTTAGCTAATTCTTCAATTGATATTATTCT

TCACGATACTTACTATGTAGTAGCTCATTTTCACTATGTTCTTTCTATAGGAGCTG
TATTTGCAATTATAGCCGGAATTGTCCAATGATTCCCATTATTTACCGGACTAAC
CTTAAATAATTTTTTTTTAAAAACCCAATTTATTACTATATTTTTAGGTGTAAATTTA
ACATTTTTTCCCCAACACTTTTTAGGGCTAAGAGGAATGCCTCGGCGATATTCGG
ATTATCCAGATGCTTTCACTATCTGAAATGCCATTTCATCTATTGGTTCCATTATT
TCTCTCACAAGAATTATCTTTTTTTTATTTATCTTATGAGAAAGGCTTTCTTCCCAA
CGTAAATCTTTGAGAACTTTAAATGTAACAACTTCAATTGAATGGTTACAAGCCCT
ACCCCCTAGTG
CMO1_2
ATTATTAGACAAGAAAGGGGGAAAAAAGAAGCTTTTGGAACCTTAGGAATAATTT
ATGCCATGATAGCTATCGGATTATTAGGATTCGTAGTTTGAGCTCACCATATATT
TACAGTAGGGATAGATGTAGATACCCGAGCTTACTTCACTTCAGCAACTATAATT
ATTGCGGTTCCAACTGGGATTAAAGTCTTTAGATGACTAGCCACCTTTCATGGAA
CTCAAATTTTAAATAGACCAGTAACCCTGTGAGCTTTAGGTTTTGTATTTTTATTT
ACAGTAGGAGGATTAACAGGGGTAATTTTAGCTAATTCTTCAATTGATATTATTCT
TCACGATACTTACTATGTAGTAGCTCATTTTCACTATGTTCTTTCTATAGGAGCTG
TATTTGCAATTATAGCCGGAATTGTCCAATGATTCCCATTATTTACCGGACTAAC
CTTAAATAATTTTTTTTTAAAAACCCAATTTATTACTATATTTTTAGGTGTAAATTTA
ACATTTTTTCCCCAACACTTTTTAGGGCTAAGAGGAATGCCTCGGCGATATTCGG
ATTATCCAGATGCTTTCACTATCTGAAATGCCATTTCATCTATTGGTTCCATTATT
TCTCTCACAAGAATTATCTTTTTTTTATTTATCTTATGAGAAAGGCTTTCTTCCCAA
CGTAAATCTTTGAGAACTTTAAATGTAACAACTTCAATTGAATGGTTACAAGCCCT
ACCCCCTAGTG
CMO2_1
ATTATTAGACAAGAAAGGGGGAAAAAAGAAGCTTTTGGAACCTTAGGAATAATTT
ATGCCATGATAGCTATCGGATTATTAGGATTCGTAGTTTGAGCTCACCATATATT
TACAGTAGGGATAGATGTAGATACCCGAGCTTACTTCACTTCAGCAACTATAATT
ATTGCGGTTCCAACTGGGATTAAAGTCTTTAGATGACTAGCCACCTTTCATGGAA
CTCAAATTTTAAATAGACCAGTAACCCTGTGAGCTTTAGGTTTTGTATTTTTATTT
ACAGTAGGAGGATTAACAGGGGTAATTTTAGCTAATTCTTCAATTGATATTATTCT
TCACGATACTTACTATGTAGTAGCTCATTTTCACTATGTTCTTTCTATAGGAGCTG
TATTTGCAATTATAGCCGGAATTGTCCAATGATTCCCATTATTTACCGGACTAAC
CTTAAATAATTTTTTTTTAAAAACCCAATTTATTACTATATTTTTAGGTGTAAATTTA
ACATTTTTTCCCCAACACTTTTTAGGGCTAAGAGGAATGCCTCGGCGATATTCGG
ATTATCCAGATGCTTTCACTATCTGAAATGCCATTTCATCTATTGGTTCCATTATT
TCTCTCACAAGAATTATCTTTTTTTTATTTATCTTATGAGAAAGGCTTTCTTCCCAA
CGTAAATCTTTGAGAACTTTAAATGTAACAACTTCAATTGAATGGTTACAAGCCCT
ACCCCCTAGTG
CMO2_2
ATTATTAGACAAGAAAGGGGGAAAAAAGAAGCTTTTGGAACCTTAGGAATAATTT
ATGCCATGATAGCTATCGGATTATTAGGATTCGTAGTTTGAGCTCACCATATATT
TACAGTAGGGATAGATGTAGATACCCGAGCTTACTTCACTTCAGCAACTATAATT
ATTGCGGTTCCAACTGGGATTAAAGTCTTTAGATGACTAGCCACCTTTCATGGAA
CTCAAATTTTAAATAGACCAGTAACCCTGTGAGCTTTAGGTTTTGTATTTTTATTT
ACAGTAGGAGGATTAACAGGGGTAATTTTAGCTAATTCTTCAATTGATATTATTCT
TCACGATACTTACTATGTAGTAGCTCATTTTCACTATGTTCTTTCTATAGGAGCTG
TATTTGCAATTATAGCCGGAATTGTCCAATGATTCCCATTATTTACCGGACTAAC
CTTAAATAATTTTTTTTTAAAAACCCAATTTATTACTATATTTTTAGGTGTAAATTTA
ACATTTTTTCCCCAACACTTTTTAGGGCTAAGAGGAATGCCTCGGCGATATTCGG
ATTATCCAGATGCTTTCACTATCTGAAATGCCATTTCATCTATTGGTTCCATTATT

TCTCTCACAAGAATTATCTTTTTTTTTATTTATCTTATGAGAAAGGCTTTCTTCCCAA
CGTAAATCTTTGAGAACTTTAAATGTAACAACTTCAATTGAATGGTTACAAGCCCT
ACCCCCTAGTG
COI1
ATTATTAGACAAGAAAGGGGGAAAAAAGAAGCTTTTGGAACCTTAGGAATAATTT
ATGCCATGATAGCTATCGGATTATTAGGATTCGTAGTTTGAGCTCACCATATATT
TACAGTAGGGATAGATGTAGATACCCGAGCTTACTTCACTTCAGCAACTATAATT
ATTGCGGTTCCAACTGGGATTAAAGTCTTTAGATGACTAGCCACCTTTCATGGAA
CTCAAATTTTAAATAGACCAGTAACCCTGTGAGCTTTAGGTTTTGTATTTTTATTT
ACAGTAGGAGGATTAACAGGGGTAATTTTAGCTAATTCTTCAATTGATATTATTCT
TCACGATACTTACTATGTAGTAGCTCATTTTCACTATGTTCTTTCTATAGGAGCTG
TATTTGCAATTATAGCCGGAATTGTCCAATGATTCCCATTATTTACCGGACTAAC
CTTAAATAATTTTTTTTTAAAAACCCAATTTATTACTATATTTTTAGGTGTAAATTTA
ACATTTTTTCCCCAACACTTTTTAGGGCTAAGAGGAATGCCTCGGCGATATTCGG
ATTATCCAGATGCTTTCACTATCTGAAATGCCATTTCATCTATTGGTTCCATTATT
TCTCTCACAAGAATTATCTTTTTTTTTATTTATCTTATGAGAAAGGCTTTCTTCCCAA
CGTAAATCTTTGAGAACTTTAAATGTAACAACTTCAATTGAATGGTTACAAGCCCT
ACCCCCTAGTG
CSJ1
ATTATTAGACAAGAAAGGGGGAAAAAAGAAGCTTTTGGAACCTTAGGAATAATTT
ATGCCATGATAGCTATCGGATTATTAGGATTCGTAGTTTGAGCTCACCATATATT
TACAGTAGGGATAGATGTAGATACCCGAGCTTACTTCACTTCAGCAACTATAATT
ATTGCGGTTCCAACTGGGATTAAAGTCTTTAGATGACTAGCCACCTTTCATGGAA
CTCAAATTTTAAATAGACCAGTAACCCTGTGAGCTTTAGGTTTTGTATTTTTATTT
ACAGTAGGAGGATTAACAGGGGTAATTTTAGCTAATTCTTCAATTGATATTATTCT
TCACGATACTTACTATGTAGTAGCTCATTTTCACTATGTTCTTTCTATAGGAGCTG
TATTTGCAATTATAGCCGGAATTGTCCAATGATTCCCATTATTTACCGGACTAAC
CTTAAATAATTTTTTTTTAAAAACCCAATTTATTACTATATTTTTAGGTGTAAATTTA
ACATTTTTTCCCCAACACTTTTTAGGGCTAAGAGGAATGCCTCGGCGATATTCGG
ATTATCCAGATGCTTTCACTATCTGAAATGCCATTTCATCTATTGGTTCCATTATT
TCTCTCACAAGAATTATCTTTTTTTTTATTTATCTTATGAGAAAGGCTTTCTTCCCAA
CGTAAATCTTTGAGAACTTTAAATGTAACAACTTCAATTGAATGGTTACAAGCCCT
ACCCCCTAGTG
ERV1
ATTATTAGACAAGAAAGGGGGAAAAAAGAAGCTTTTGGAACCTTAGGAATAATTT
ATGCCATGATAGCTATCGGATTATTAGGATTCGTAGTTTGAGCTCACCATATATT
TACAGTAGGGATAGATGTAGATACCCGAGCTTACTTCACTTCAGCAACTATAATT
ATTGCGGTTCCAACTGGGATTAAAGTCTTTAGATGACTAGCCACCTTTCATGGAA
CTCAAATTTTAAATAGACCAGTAACCCTGTGAGCTTTAGGTTTTGTATTTTTATTT
ACAGTAGGAGGATTAACAGGGGTAATTTTAGCTAATTCTTCAATTGATATTATTCT
TCACGATACTTACTATGTAGTAGCTCATTTTCACTATGTTCTTTCTATAGGAGCTG
TATTTGCAATTATAGCCGGAATTGTCCAATGATTCCCATTATTTACCGGACTAAC
CTTAAATAATTTTTTTTTAAAAACCCAATTTATTACTATATTTTTAGGTGTAAATTTA
ACATTTTTTCCCCAACACTTTTTAGGGCTAAGAGGAATGCCTCGGCGATATTCGG
ATTATCCAGATGCTTTCACTATCTGAAATGCCATTTCATCTATTGGTTCCATTATT
TCTCTCACAAGAATTATCTTTTTTTTTATTTATCTTATGAGAAAGGCTTTCTTCCCAA
CGTAAATCTTTGAGAACTTTAAATGTAACAACTTCAATTGAATGGTTACAAGCCCT
ACCCCCTAGTG
ERV2
ATTATTAGACAAGAAAGGGGGAAAAAAGAAGCTTTTGGAACCTTAGGAATAATTT

ATGCCATGATAGCTATCGGATTATTAGGATTCGTAGTTTGAGCTCACCATATATT
TACAGTAGGGATAGATGTAGATACCCGAGCTTACTTCACTTCAGCAACTATAATT
ATTGCGGTTCCAACTGGGATTAAAGTCTTTAGATGACTAGCCACCTTTCATGGAA
CTCAAATTTTAAATAGACCAGTAACCCTGTGAGCTTTAGGTTTTGTATTTTTATTT
ACAGTAGGAGGATTAACAGGGGTAATTTTAGCTAATTCTTCAATTGATATTATTCT
TCACGATACTTACTATGTAGTAGCTCATTTTCACTATGTTCTTTCTATAGGAGCTG
TATTTGCAATTATAGCCGGAATTGTCCAATGATTCCCATTATTTACCGGACTAAC
CTTAAATAATTTTTTTTTAAAAACCCAATTTATTACTATATTTTTAGGTGTAAATTTA
ACATTTTTTCCCCAACACTTTTTAGGGCTAAGAGGAATGCCTCGGCGATATTCGG
ATTATCCAGATGCTTTCACTATCTGAAATGCCATTTCATCTATTGGTTCCATTATT
TCTCTCACAAGAATTATCTTTTTTTTATTTATCTTATGAGAAAGGCTTTCTTCCCAA
CGTAAATCTTTGAGAACTTTAAATGTAACAACTTCAATTGAATGGTTACAAGCCCT
ACCCCCTAGTG
GOV1
ATTATTAGACAAGAAAGGGGGAAAAAAGAAGCTTTTGGAACCTTAGGAATAATTT
ATGCCATGATAGCTATCGGATTATTAGGATTCGTAGTTTGAGCTCACCATATATT
TACAGTAGGGATAGATGTAGATACCCGAGCTTACTTCACTTCAGCAACTATAATT
ATTGCGGTTCCAACTGGGATTAAAGTCTTTAGATGACTAGCCACCTTTCATGGAA
CTCAAATTTTAAATAGACCAGTAACCCTGTGAGCTTTAGGTTTTGTATTTTTATTT
ACAGTAGGAGGATTAACAGGGGTAATTTTAGCTAATTCTTCAATTGATATTATTCT
TCACGATACTTACTATGTAGTAGCTCATTTTCACTATGTTCTTTCTATAGGAGCTG
TATTTGCAATTATAGCCGGAATTGTCCAATGATTCCCATTATTTACCGGACTAAC
CTTAAATAATTTTTTTTTAAAAACCCAATTTATTACTATATTTTTAGGTGTAAATTTA
ACATTTTTTCCCCAACACTTTTTAGGGCTAAGAGGAATGCCTCGGCGATATTCGG
ATTATCCAGATGCTTTCACTATCTGAAATGCCATTTCATCTATTGGTTCCATTATT
TCTCTCACAAGAATTATCTTTTTTTTATTTATCTTATGAGAAAGGCTTTCTTCCCAA
CGTAAATCTTTGAGAACTTTAAATGTAACAACTTCAATTGAATGGTTACAAGCCCT
ACCCCCTAGTG
GOV2
ATTATTAGACAAGAAAGGGGGAAAAAAGAAGCTTTTGGAACCTTAGGAATAATTT
ATGCCATGATAGCTATCGGATTATTAGGATTCGTAGTTTGAGCTCACCATATATT
TACAGTAGGGATAGATGTAGATACCCGAGCTTACTTCACTTCAGCAACTATAATT
ATTGCGGTTCCAACTGGGATTAAAGTCTTTAGATGACTAGCCACCTTTCATGGAA
CTCAAATTTTAAATAGACCAGTAACCCTGTGAGCTTTAGGTTTTGTATTTTTATTT
ACAGTAGGAGGATTAACAGGGGTAATTTTAGCTAATTCTTCAATTGATATTATTCT
TCACGATACTTACTATGTAGTAGCTCATTTTCACTATGTTCTTTCTATAGGAGCTG
TATTTGCAATTATAGCCGGAATTGTCCAATGATTCCCATTATTTACCGGACTAAC
CTTAAATAATTTTTTTTTAAAAACCCAATTTATTACTATATTTTTAGGTGTAAATTTA
ACATTTTTTCCCCAACACTTTTTAGGGCTAAGAGGAATGCCTCGGCGATATTCGG
ATTATCCAGATGCTTTCACTATCTGAAATGCCATTTCATCTATTGGTTCCATTATT
TCTCTCACAAGAATTATCTTTTTTTTATTTATCTTATGAGAAAGGCTTTCTTCCCAA
CGTAAATCTTTGAGAACTTTAAATGTAACAACTTCAATTGAATGGTTACAAGCCCT
ACCCCCTAGTG
HEM1
ATTATTAGACAAGAAAGGGGGAAAAAAGAAGCTTTTGGAACCTTAGGAATAATTT
ATGCCATGATAGCTATCGGATTATTAGGATTCGTAGTTTGAGCTCACCATATATT
TACAGTAGGGATAGATGTAGATACCCGAGCTTACTTCACTTCAGCAACTATAATT
ATTGCGGTTCCAACTGGGATTAAAGTCTTTAGATGACTAGCCACCTTTCATGGAA
CTCAAATTTTAAATAGACCAGTAACCCTGTGAGCTTTAGGTTTTGTATTTTTATTT
ACAGTAGGAGGATTAACAGGGGTAATTTTAGCTAATTCTTCAATTGATATTATTCT

TCACGATACTTACTATGTAGTAGCTCATTTTCACTATGTTCTTTCTATAGGAGCTG
TATTTGCAATTATAGCCGGAATTGTCCAATGATTCCCATTATTTACCGGACTAAC
CTTAAATAATTTTTTTTTAAAAACCCAATTTATTACTATATTTTTAGGTGTAAATTTA
ACATTTTTTCCCCAACACTTTTTAGGGCTAAGAGGAATGCCTCGGCGATATTCGG
ATTATCCAGATGCTTTCACTATCTGAAATGCCATTTCATCTATTGGTTCCATTATT
TCTCTCACAAGAATTATCTTTTTTTTATTTATCTTATGAGAAAGGCTTTCTTCCCAA
CGTAAATCTTTGAGAACTTTAAATGTAACAACTTCAATTGAATGGTTACAAGCCCT
ACCCCCTAGTG
HEM2
ATTATTAGACAAGAAAGGGGGAAAAAAGAAGCTTTTGGAACCTTAGGAATAATTT
ATGCCATGATAGCTATCGGATTATTAGGATTCGTAGTTTGAGCTCACCATATATT
TACAGTAGGGATAGATGTAGATACCCGAGCTTACTTCACTTCAGCAACTATAATT
ATTGCGGTTCCAACTGGGATTAAAGTCTTTAGATGACTAGCCACCTTTCATGGAA
CTCAAATTTTAAATAGACCAGTAACCCTGTGAGCTTTAGGTTTTGTATTTTTATTT
ACAGTAGGAGGATTAACAGGGGTAATTTTAGCTAATTCTTCAATTGATATTATTCT
TCACGATACTTACTATGTAGTAGCTCATTTTCACTATGTTCTTTCTATAGGAGCTG
TATTTGCAATTATAGCCGGAATTGTCCAATGATTCCCATTATTTACCGGACTAAC
CTTAAATAATTTTTTTTTAAAAACCCAATTTATTACTATATTTTTAGGTGTAAATTTA
ACATTTTTTCCCCAACACTTTTTAGGGCTAAGAGGAATGCCTCGGCGATATTCGG
ATTATCCAGATGCTTTCACTATCTGAAATGCCATTTCATCTATTGGTTCCATTATT
TCTCTCACAAGAATTATCTTTTTTTTATTTATCTTATGAGAAAGGCTTTCTTCCCAA
CGTAAATCTTTGAGAACTTTAAATGTAACAACTTCAATTGAATGGTTACAAGCCCT
ACCCCCTAGTG
HER1
ATTATTAGACAAGAAAGGGGGAAAAAAGAAGCTTTTGGAACCTTAGGAATAATTT
ATGCCATGATAGCTATCGGATTATTAGGATTCGTAGTTTGAGCTCACCATATATT
TACAGTAGGGATAGATGTAGATACCCGAGCTTACTTCACTTCAGCAACTATAATT
ATTGCGGTTCCAACTGGGATTAAAGTCTTTAGATGACTAGCCACCTTTCATGGAA
CTCAAATTTTAAATAGACCAGTAACCCTGTGAGCTTTAGGTTTTGTATTTTTATTT
ACAGTAGGAGGATTAACAGGGGTAATTTTAGCTAATTCTTCAATTGATATTATTCT
TCACGATACTTACTATGTAGTAGCTCATTTTCACTATGTTCTTTCTATAGGAGCTG
TATTTGCAATTATAGCCGGAATTGTCCAATGATTCCCATTATTTACCGGACTAAC
CTTAAATAATTTTTTTTTAAAAACCCAATTTATTACTATATTTTTAGGTGTAAATTTA
ACATTTTTTCCCCAACACTTTTTAGGGCTAAGAGGAATGCCTCGGCGATATTCGG
ATTATCCAGATGCTTTCACTATCTGAAATGCCATTTCATCTATTGGTTCCATTATT
TCTCTCACAAGAATTATCTTTTTTTTATTTATCTTATGAGAAAGGCTTTCTTCCCAA
CGTAAATCTTTGAGAACTTTAAATGTAACAACTTCAATTGAATGGTTACAAGCCCT
ACCCCCTAGTG
HER2
ATTATTAGACAAGAAAGGGGGAAAAAAGAAGCTTTTGGAACCTTAGGAATAATTT
ATGCCATGATAGCTATCGGATTATTAGGATTCGTAGTTTGAGCTCACCATATATT
TACAGTAGGGATAGATGTAGATACCCGAGCTTACTTCACTTCAGCAACTATAATT
ATTGCGGTTCCAACTGGGATTAAAGTCTTTAGATGACTAGCCACCTTTCATGGAA
CTCAAATTTTAAATAGACCAGTAACCCTGTGAGCTTTAGGTTTTGTATTTTTATTT
ACAGTAGGAGGATTAACAGGGGTAATTTTAGCTAATTCTTCAATTGATATTATTCT
TCACGATACTTACTATGTAGTAGCTCATTTTCACTATGTTCTTTCTATAGGAGCTG
TATTTGCAATTATAGCCGGAATTGTCCAATGATTCCCATTATTTACCGGACTAAC
CTTAAATAATTTTTTTTTAAAAACCCAATTTATTACTATATTTTTAGGTGTAAATTTA
ACATTTTTTCCCCAACACTTTTTAGGGCTAAGAGGAATGCCTCGGCGATATTCGG
ATTATCCAGATGCTTTCACTATCTGAAATGCCATTTCATCTATTGGTTCCATTATT

TCTCTCACAAGAATTATCTTTTTTTTATTTATCTTATGAGAAAGGCTTTCTTCCCAA
CGTAAATCTTTGAGAACTTTAAATGTAACAACTTCAATTGAATGGTTACAAGCCCT
ACCCCCTAGTG
HEV1
ATTATTAGACAAGAAAGGGGGAAAAAAGAAGCTTTTGGAACCTTAGGAATAATTT
ATGCCATGATAGCTATCGGATTATTAGGATTCGTAGTTTGAGCTCACCATATATT
TACAGTAGGGATAGATGTAGATACCCGAGCTTACTTCACTTCAGCAACTATAATT
ATTGCGGTTCCAACTGGGATTAAAGTCTTTAGATGACTAGCCACCTTTCATGGAA
CTCAAATTTTAAATAGACCAGTAACCCTGTGAGCTTTAGGTTTTGTATTTTTATTT
ACAGTAGGAGGATTAACAGGGGTAATTTTAGCTAATTCTTCAATTGATATTATTCT
TCACGATACTTACTATGTAGTAGCTCATTTTCACTATGTTCTTTCTATAGGAGCTG
TATTTGCAATTATAGCCGGAATTGTCCAATGATTCCCATTATTTACCGGACTAAC
CTTAAATAATTTTTTTTTAAAAACCCAATTTATTACTATATTTTTAGGTGTAAATTTA
ACATTTTTTCCCCAACACTTTTTAGGGCTAAGAGGAATGCCTCGGCGATATTCGG
ATTATCCAGATGCTTTCACTATCTGAAATGCCATTTCATCTATTGGTTCCATTATT
TCTCTCACAAGAATTATCTTTTTTTTATTTATCTTATGAGAAAGGCTTTCTTCCCAA
CGTAAATCTTTGAGAACTTTAAATGTAACAACTTCAATTGAATGGTTACAAGCCCT
ACCCCCTAGTG
HEV1_2
ATTATTAGACAAGAAAGGGGGAAAAAAGAAGCTTTTGGAACCTTAGGAATAATTT
ATGCCATGATAGCTATCGGATTATTAGGATTCGTAGTTTGAGCTCACCATATATT
TACAGTAGGGATAGATGTAGATACCCGAGCTTACTTCACTTCAGCAACTATAATT
ATTGCGGTTCCAACTGGGATTAAAGTCTTTAGATGACTAGCCACCTTTCATGGAA
CTCAAATTTTAAATAGACCAGTAACCCTGTGAGCTTTAGGTTTTGTATTTTTATTT
ACAGTAGGAGGATTAACAGGGGTAATTTTAGCTAATTCTTCAATTGATATTATTCT
TCACGATACTTACTATGTAGTAGCTCATTTTCACTATGTTCTTTCTATAGGAGCTG
TATTTGCAATTATAGCCGGAATTGTCCAATGATTCCCATTATTTACCGGACTAAC
CTTAAATAATTTTTTTTTAAAAACCCAATTTATTACTATATTTTTAGGTGTAAATTTA
ACATTTTTTCCCCAACACTTTTTAGGGCTAAGAGGAATGCCTCGGCGATATTCGG
ATTATCCAGATGCTTTCACTATCTGAAATGCCATTTCATCTATTGGTTCCATTATT
TCTCTCACAAGAATTATCTTTTTTTTATTTATCTTATGAGAAAGGCTTTCTTCCCAA
CGTAAATCTTTGAGAACTTTAAATGTAACAACTTCAATTGAATGGTTACAAGCCCT
ACCCCCTAGTG
HEV2
ATTATTAGACAAGAAAGGGGGAAAAAAGAAGCTTTTGGAACCTTAGGAATAATTT
ATGCCATGATAGCTATCGGATTATTAGGATTCGTAGTTTGAGCTCACCATATATT
TACAGTAGGGATAGATGTAGATACCCGAGCTTACTTCACTTCAGCAACTATAATT
ATCGCGGTTCCAACTGGGATTAAAGTCTTTAGGTGACTAGCCACCTTTCATGGA
ACTCAAATTTTAAATAGACCAGTAACCCTGTGAGCTTTAGGTTTTGTATTTTTATT
TACAGTAGGAGGATTAACAGGGGTAATTTTAGCTAATTCTTCAATTGATATTATTC
TTCACGATACTTACTATGTAGTAGCTCATTTTCACTATGTTCTTTCTATAGGAGCT
GTATTTGCAATTATAGCCGGAATTGTCCAATGATTCCCATTATTTACTGGACTAA
CCTTAAATAATTTTTTTTTAAAAACCCAATTTATTACTATATTTTTAGGTGTAAATTT
AACATTTTTTCCTCAACACTTTTTAGGGCTAAGAGGAATGCCTCGGCGATATTCG
GATTATCCAGATGCTTTCACTATCTGAAATGCCATTTCATCTATTGGTTCCATTAT
TTCTCTCACAAGAATTATCTTTTTTTTATTTATCTTATGAGAAAGGCTTTCTTCCCA
ACGTAAATCTTTGAGAACTTTAAATGTAACAACTTCAATTGAATGGTTACAAGCC
CTACCCCCTAGTG
HEV2_2
ATTATTAGACAAGAAAGGGGGAAAAAAGAAGCTTTTGGAACCTTAGGAATAATTT

ATGCCATGATAGCTATCGGATTATTAGGATTCGTAGTTTGAGCTCACCATATATT
TACAGTAGGGATAGATGTAGATACCCGAGCTTACTTCACTTCAGCAACTATAATT
ATCGCGGTTCCAACTGGGATTAAAGTCTTTAGGTGACTAGCCACCTTTCATGGA
ACTCAAATTTTAAATAGACCAGTAACCCTGTGAGCTTTAGGTTTTGTATTTTTATT
TACAGTAGGAGGATTAACAGGGGTAATTTTAGCTAATTCTTCAATTGATATTATTC
TTCACGATACTTACTATGTAGTAGCTCATTTTCACTATGTTCTTTCTATAGGAGCT
GTATTTGCAATTATAGCCGGAATTGTCCAATGATTCCCATTATTTACTGGACTAA
CCTTAAATAATTTTTTTTTAAAAACCCAATTTATTACTATATTTTTAGGTGTAAATTT
AACATTTTTTCCTCAACACTTTTTAGGGCTAAGAGGAATGCCTCGGCGATATTCG
GATTATCCAGATGCTTTCACTATCTGAAATGCCATTTCATCTATTGGTTCCATTAT
TTCTCTCACAAGAATTATCTTTTTTTTTATTTATCTTATGAGAAAGGCTTTCTTCCCA
ACGTAAATCTTTGAGAACTTTAAATGTAACAACTTCAATTGAATGGTTACAAGCC
CTACCCCCTAGTG
JAB2
ATTATTAGACAAGAAAGGGGGAAAAAAGAAGCTTTTGGAACCTTAGGAATAATTT
ATGCCATGATAGCTATCGGATTATTAGGATTCGTAGTTTGAGCTCACCATATATT
TACAGTAGGGATAGATGTAGATACCCGAGCTTACTTCACTTCAGCAACTATAATT
ATTGCGGTTCCAACTGGGATTAAAGTCTTTAGATGACTAGCCACCTTTCATGGAA
CTCAAATTTTAAATAGACCAGTAACCCTGTGAGCTTTAGGTTTTGTATTTTTATTT
ACAGTAGGAGGATTAACAGGGGTAATTTTAGCTAATTCTTCAATTGATATTATTCT
TCACGATACTTACTATGTAGTAGCTCATTTTCACTATGTTCTTTCTATAGGAGCTG
TATTTGCAATTATAGCCGGAATTGTCCAATGATTCCCATTATTTACCGGACTAAC
CTTAAATAATTTTTTTTTAAAAACCCAATTTATTACTATATTTTTAGGTGTAAATTTA
ACATTTTTTCCCCAACACTTTTTAGGGCTAAGAGGAATGCCTCGGCGATATTCGG
ATTATCCAGATGCTTTCACTATCTGAAATGCCATTTCATCTATTGGTTCCATTATT
TCTCTCACAAGAATTATCTTTTTTTTTATTTATCTTATGAGAAAGGCTTTCTTCCCAA
CGTAAATCTTTGAGAACTTTAAATGTAACAACTTCAATTGAATGGTTACAAGCCCT
ACCCCCTAGTG
JAB4
ATTATTAGACAAGAAAGGGGGAAAAAAGAAGCTTTTGGAACCTTAGGAATAATTT
ATGCCATGATAGCTATCGGATTATTAGGATTCGTAGTTTGAGCTCACCATATATT
TACAGTAGGGATAGATGTAGATACCCGAGCTTACTTCACTTCAGCAACTATAATT
ATTGCGGTTCCAACTGGGATTAAAGTCTTTAGATGACTAGCCACCTTTCATGGAA
CTCAAATTTTAAATAGACCAGTAACCCTGTGAGCTTTAGGTTTTGTATTTTTATTT
ACAGTAGGAGGATTAACAGGGGTAATTTTAGCTAATTCTTCAATTGATATTATTCT
TCACGATACTTACTATGTAGTAGCTCATTTTCACTATGTTCTTTCTATAGGAGCTG
TATTTGCAATTATAGCCGGAATTGTCCAATGATTCCCATTATTTACCGGACTAAC
CTTAAATAATTTTTTTTTAAAAACCCAATTTATTACTATATTTTTAGGTGTAAATTTA
ACATTTTTTCCCCAACACTTTTTAGGGCTAAGAGGAATGCCTCGGCGATATTCGG
ATTATCCAGATGCTTTCACTATCTGAAATGCCATTTCATCTATTGGTTCCATTATT
TCTCTCACAAGAATTATCTTTTTTTTTATTTATCTTATGAGAAAGGCTTTCTTCCCAA
CGTAAATCTTTGAGAACTTTAAATGTAACAACTTCAATTGAATGGTTACAAGCCCT
ACCCCCTAGTG
LAV1
ATTATTAGACAAGAAAGGGGGAAAAAAGAAGCTTTTGGAACCTTAGGAATAATTT
ATGCCATGATAGCTATCGGATTATTAGGATTCGTAGTTTGAGCTCACCATATATT
TACAGTAGGGATAGATGTAGATACCCGAGCTTACTTCACTTCAGCAACTATAATT
ATTGCGGTTCCAACTGGGATTAAAGTCTTTAGATGACTAGCCACCTTTCATGGAA
CTCAAATTTTAAATAGACCAGTAACCCTGTGAGCTTTAGGTTTTGTATTTTTATTT
ACAGTAGGAGGATTAACAGGGGTAATTTTAGCTAATTCTTCAATTGATATTATTCT

TCACGATACTTACTATGTAGTAGCTCATTTTCACTATGTTCTTTCTATAGGAGCTG
TATTTGCAATTATAGCCGGAATTGTCCAATGATTCCCATTATTTACCGGACTAAC
CTTAAATAATTTTTTTTTAAAAACCCAATTTATTACTATATTTTTAGGTGTAAATTTA
ACATTTTTTCCCCAACACTTTTTAGGGCTAAGAGGAATGCCTCGGCGATATTCGG
ATTATCCAGATGCTTTCACTATCTGAAATGCCATTTCATCTATTGGTTCCATTATT
TCTCTCACAAGAATTATCTTTTTTTTATTTATCTTATGAGAAAGGCTTTCTTCCCAA
CGTAAATCTTTGAGAACTTTAAATGTAACAACTTCAATTGAATGGTTACAAGCCCT
ACCCCCTAGTG
LAV2
ATTATTAGACAAGAAAGGGGGAAAAAAGAAGCTTTTGGAACCTTAGGAATAATTT
ATGCCATGATAGCTATCGGATTATTAGGATTCGTAGTTTGAGCTCACCATATATT
TACAGTAGGGATAGATGTAGATACCCGAGCTTACTTCACTTCAGCAACTATAATT
ATTGCGGTTCCAACTGGGATTAAAGTCTTTAGATGACTAGCCACCTTTCATGGAA
CTCAAATTTTAAATAGACCAGTAACCCTGTGAGCTTTAGGTTTTGTATTTTTATTT
ACAGTAGGAGGATTAACAGGGGTAATTTTAGCTAATTCTTCAATTGATATTATTCT
TCACGATACTTACTATGTAGTAGCTCATTTTCACTATGTTCTTTCTATAGGAGCTG
TATTTGCAATTATAGCCGGAATTGTTCAATGATTCCCATTATTTACCGGACTAACC
TTAAATAATTTTTTTTTAAAAACCCAATTTATTACTATATTTTTAGGTGTAAATTTAA
CATTTTTTCCCCAACACTTTTTAGGGCTAAGAGGAATGCCTCGGCGATATTCGGA
TTATCCAGATGCTTTCACTATCTGAAATGCCATTTCATCTATTGGTTCCATTATTT
CTCTCACAAGAATTATCTTTTTTTTATTTATCTTATGAGAAAGGCTTTCTTCCCAA
CGTAAATCTTTGAGAACTTTAAATGTAACAACTTCAATTGAATGGTTACAAGCCCT
ACCCCCTAGTG
MAC2
ATTATTAGACAAGAAAGGGGGAAAAAAGAAGCTTTTGGAACCTTAGGAATAATTT
ATGCCATGATAGCTATCGGATTATTAGGATTCGTAGTTTGAGCTCACCATATATT
TACAGTAGGGATAGATGTAGATACCCGAGCTTACTTCACTTCAGCAACTATAATT
ATTGCGGTTCCAACTGGGATTAAAGTCTTTAGATGACTAGCCACCTTTCATGGAA
CTCAAATTTTAAATAGACCAGTAACCCTGTGAGCTTTAGGTTTTGTATTTTTATTT
ACAGTAGGAGGATTAACAGGGGTAATTTTAGCTAATTCTTCAATTGATATTATTCT
TCACGATACTTACTATGTAGTAGCTCATTTTCACTATGTTCTTTCTATAGGAGCTG
TATTTGCAATTATAGCCGGAATTGTCCAATGATTCCCATTATTTACCGGACTAAC
CTTAAATAATTTTTTTTTAAAAACCCAATTTATTACTATATTTTTAGGTGTAAATTTA
ACATTTTTTCCCCAACACTTTTTAGGGCTAAGAGGAATGCCTCGGCGATATTCGG
ATTATCCAGATGCTTTCACTATCTGAAATGCCATTTCATCTATTGGTTCCATTATT
TCTCTCACAAGAATTATCTTTTTTTTATTTATCTTATGAGAAAGGCTTTCTTCCCAA
CGTAAATCTTTGAGAACTTTAAATGTAACAACTTCAATTGAATGGTTACAAGCCCT
ACCCCCTAGTG
MAC4
ATTATTAGACAAGAAAGGGGGAAAAAAGAAGCTTTTGGAACCTTAGGAATAATTT
ATGCCATGATAGCTATCGGATTATTAGGATTCGTAGTTTGAGCTCACCATATATT
TACAGTAGGGATAGATGTAGATACCCGAGCTTACTTCACTTCAGCAACTATAATT
ATTGCGGTTCCAACTGGGATTAAAGTCTTTAGATGACTAGCCACCTTTCATGGAA
CTCAAATTTTAAATAGACCAGTAACCCTGTGAGCTTTAGGTTTTGTATTTTTATTT
ACAGTAGGAGGATTAACAGGGGTAATTTTAGCTAATTCTTCAATTGATATTATTCT
TCACGATACTTACTATGTAGTAGCTCATTTTCACTATGTTCTTTCTATAGGAGCTG
TATTTGCAATTATAGCCGGAATTGTCCAATGATTCCCATTATTTACCGGACTAAC
CTTAAATAATTTTTTTTTAAAAACCCAATTTATTACTATATTTTTAGGTGTAAATTTA
ACATTTTTTCCCCAACACTTTTTAGGGCTAAGAGGAATGCCTCGGCGATATTCGG
ATTATCCAGATGCTTTCACTATCTGAAATGCCATTTCATCTATTGGTTCCATTATT

TCTCTCACAAGAATTATCTTTTTTTTATTTATCTTATGAGAAAGGCTTTCTTCCCAA
CGTAAATCTTTGAGAACTTTAAATGTAACAACTTCAATTGAATGGTTACAAGCCCT
ACCCCCTAGTG
MAC6
ATTATTAGACAAGAAAGGGGGAAAAAAGAAGCTTTTGGAACCTTAGGAATAATTT
ATGCCATGATAGCTATCGGATTATTAGGATTCGTAGTTTGAGCTCACCATATATT
TACAGTAGGGATAGATGTAGATACCCGAGCTTACTTCACTTCAGCAACTATAATT
ATTGCGGTTCCAACTGGGATTAAAGTCTTTAGATGACTAGCCACCTTTCATGGAA
CTCAAATTTTAAATAGACCAGTAACCCTGTGAGCTTTAGGTTTTGTATTTTTATTT
ACAGTAGGAGGATTAACAGGGGTAATTTTAGCTAATTCTTCAATTGATATTATTCT
TCACGATACTTACTATGTAGTAGCTCATTTTCACTATGTTCTTTCTATAGGAGCTG
TATTTGCAATTATAGCCGGAATTGTCCAATGATTCCCATTATTTACCGGACTAAC
CTTAAATAATTTTTTTTTAAAAACCCAATTTATTACTATATTTTTAGGTGTAAATTTA
ACATTTTTTCCCCAACACTTTTTAGGGCTAAGAGGAATGCCTCGGCGATATTCGG
ATTATCCAGATGCTTTCACTATCTGAAATGCCATTTCATCTATTGGTTCCATTATT
TCTCTCACAAGAATTATCTTTTTTTTATTTATCTTATGAGAAAGGCTTTCTTCCCAA
CGTAAATCTTTGAGAACTTTAAATGTAACAACTTCAATTGAATGGTTACAAGCCCT
ACCCCCTAGTG
MAR2_1
ATTATTAGACAAGAAAGGGGGAAAAAAGAAGCTTTTGGAACCTTAGGAATAATTT
ATGCCATGATAGCTATCGGATTATTAGGATTCGTAGTTTGAGCTCACCATATATT
TACAGTAGGGATAGATGTAGATACCCGAGCTTACTTCACTTCAGCAACTATAATT
ATTGCGGTTCCAACTGGGATTAAAGTCTTTAGATGACTAGCCACCTTTCATGGAA
CTCAAATTTTAAATAGACCAGTAACCCTGTGAGCTTTAGGTTTTGTATTTTTATTT
ACAGTAGGAGGATTAACAGGGGTAATTTTAGCTAATTCTTCAATTGATATTATTCT
TCACGATACTTACTATGTAGTAGCTCATTTTCACTATGTTCTTTCTATAGGAGCTG
TATTTGCAATTATAGCCGGAATTGTCCAATGATTCCCATTATTTACCGGACTAAC
CTTAAATAATTTTTTTTTAAAAACCCAATTTATTACTATATTTTTAGGTGTAAATTTA
ACATTTTTTCCCCAACACTTTTTAGGGCTAAGAGGAATGCCTCGGCGATATTCGG
ATTATCCAGATGCTTTCACTATCTGAAATGCCATTTCATCTATTGGTTCCATTATT
TCTCTCACAAGAATTATCTTTTTTTTATTTATCTTATGAGAAAGGCTTTCTTCCCAA
CGTAAATCTTTGAGAACTTTAAATGTAACAACTTCAATTGAATGGTTACAAGCCCT
ACCCCCTAGTG
MAR2_2
ATTATTAGACAAGAAAGGGGGAAAAAAGAAGCTTTTGGAACCTTAGGAATAATTT
ATGCCATGATAGCTATCGGATTATTAGGATTCGTAGTTTGAGCTCACCATATATT
TACAGTAGGGATAGATGTAGATACCCGAGCTTACTTCACTTCAGCAACTATAATT
ATTGCGGTTCCAACTGGGATTAAAGTCTTTAGATGACTAGCCACCTTTCATGGAA
CTCAAATTTTAAATAGACCAGTAACCCTGTGAGCTTTAGGTTTTGTATTTTTATTT
ACAGTAGGAGGATTAACAGGGGTAATTTTAGCTAATTCTTCAATTGATATTATTCT
TCACGATACTTACTATGTAGTAGCTCATTTTCACTATGTTCTTTCTATAGGAGCTG
TATTTGCAATTATAGCCGGAATTGTCCAATGATTCCCATTATTTACCGGACTAAC
CTTAAATAATTTTTTTTTAAAAACCCAATTTATTACTATATTTTTAGGTGTAAATTTA
ACATTTTTTCCCCAACACTTTTTAGGGCTAAGAGGAATGCCTCGGCGATATTCGG
ATTATCCAGATGCTTTCACTATCTGAAATGCCATTTCATCTATTGGTTCCATTATT
TCTCTCACAAGAATTATCTTTTTTTTATTTATCTTATGAGAAAGGCTTTCTTCCCAA
CGTAAATCTTTGAGAACTTTAAATGTAACAACTTCAATTGAATGGTTACAAGCCCT
ACCCCCTAGTG
MAR3_1
ATTATTAGACAAGAAAGGGGGAAAAAAGAAGCTTTTGGAACCTTAGGAATAATTT

ATGCCATGATAGCTATCGGATTATTAGGATTCGTAGTTTGAGCTCACCATATATT
TACAGTAGGGATAGATGTAGATACCCGAGCTTACTTCACTTCAGCAACTATAATT
ATTGCGGTTCCAACTGGGATTAAAGTCTTTAGATGACTAGCCACCTTTCATGGAA
CTCAAATTTTAAATAGACCAGTAACCCTGTGAGCTTTAGGTTTTGTATTTTTATTT
ACAGTAGGAGGATTAACAGGGGTAATTTTAGCTAATTCTTCAATTGATATTATTCT
TCACGATACTTACTATGTAGTAGCTCATTTTCACTATGTTCTTTCTATAGGAGCTG
TATTTGCAATTATAGCCGGAATTGTCCAATGATTCCCATTATTTACCGGACTAAC
CTTAAATAATTTTTTTTTAAAAACCCAATTTATTACTATATTTTTAGGTGTAAATTTA
ACATTTTTTCCCCAACACTTTTTAGGGCTAAGAGGAATGCCTCGGCGATATTCGG
ATTATCCAGATGCTTTCACTATCTGAAATGCCATTTCATCTATTGGTTCCATTATT
TCTCTCACAAGAATTATCTTTTTTTTATTTATCTTATGAGAAAGGCTTTCTTCCCAA
CGTAAATCTTTGAGAACTTTAAATGTAACAACTTCAATTGAATGGTTACAAGCCCT
ACCCCCTAGTG
MAR3_2
ATTATTAGACAAGAAAGGGGGAAAAAAGAAGCTTTTGGAACCTTAGGAATAATTT
ATGCCATGATAGCTATCGGATTATTAGGATTCGTAGTTTGAGCTCACCATATATT
TACAGTAGGGATAGATGTAGATACCCGAGCTTACTTCACTTCAGCAACTATAATT
ATTGCGGTTCCAACTGGGATTAAAGTCTTTAGATGACTAGCCACCTTTCATGGAA
CTCAAATTTTAAATAGACCAGTAACCCTGTGAGCTTTAGGTTTTGTATTTTTATTT
ACAGTAGGAGGATTAACAGGGGTAATTTTAGCTAATTCTTCAATTGATATTATTCT
TCACGATACTTACTATGTAGTAGCTCATTTTCACTATGTTCTTTCTATAGGAGCTG
TATTTGCAATTATAGCCGGAATTGTCCAATGATTCCCATTATTTACCGGACTAAC
CTTAAATAATTTTTTTTTAAAAACCCAATTTATTACTATATTTTTAGGTGTAAATTTA
ACATTTTTTCCCCAACACTTTTTAGGGCTAAGAGGAATGCCTCGGCGATATTCGG
ATTATCCAGATGCTTTCACTATCTGAAATGCCATTTCATCTATTGGTTCCATTATT
TCTCTCACAAGAATTATCTTTTTTTTATTTATCTTATGAGAAAGGCTTTCTTCCCAA
CGTAAATCTTTGAGAACTTTAAATGTAACAACTTCAATTGAATGGTTACAAGCCCT
ACCCCCTAGTG
MOC1
ATTATTAGACAAGAAAGGGGGAAAAAAGAAGCTTTTGGAACCTTAGGAATAATTT
ATGCCATGATAGCTATCGGATTATTAGGATTCGTAGTTTGAGCTCACCATATATT
TACAGTAGGGATAGATGTAGATACCCGAGCTTACTTCACTTCAGCAACTATAATT
ATTGCGGTTCCAACTGGGATTAAAGTCTTTAGATGACTAGCCACCTTTCATGGAA
CTCAAATTTTAAATAGACCAGTAACCCTGTGAGCTTTAGGTTTTGTATTTTTATTT
ACAGTAGGAGGATTAACAGGGGTAATTTTAGCTAATTCTTCAATTGATATTATTCT
TCACGATACTTACTATGTAGTAGCTCATTTTCACTATGTTCTTTCTATAGGAGCTG
TATTTGCAATTATAGCCGGAATTGTCCAATGATTCCCATTATTTACCGGACTAAC
CTTAAATAATTTTTTTTTAAAAACCCAATTTATTACTATATTTTTAGGTGTAAATTTA
ACATTTTTTCCCCAACACTTTTTAGGGCTAAGAGGAATGCCTCGGCGATATTCGG
ATTATCCAGATGCTTTCACTATCTGAAATGCCATTTCATCTATTGGTTCCATTATT
TCTCTCACAAGAATTATCTTTTTTTTATTTATCTTATGAGAAAGGCTTTCTTCCCAA
CGTAAATCTTTGAGAACTTTAAATGTAACAACTTCAATTGAATGGTTACAAGCCCT
ACCCCCTAGTG
MOC2
ATTATTAGACAAGAAAGGGGGAAAAAAGAAGCTTTTGGAACCTTAGGAATAATTT
ATGCCATGATAGCTATCGGATTATTAGGATTCGTAGTTTGAGCTCACCATATATT
TACAGTAGGGATAGATGTAGATACCCGAGCTTACTTCACTTCAGCAACTATAATT
ATTGCGGTTCCAACTGGGATTAAAGTCTTTAGATGACTAGCCACCTTTCATGGAA
CTCAAATTTTAAATAGACCAGTAACCCTGTGAGCTTTAGGTTTTGTATTTTTATTT
ACAGTAGGAGGATTAACAGGGGTAATTTTAGCTAATTCTTCAATTGATATTATTCT

TCACGATACTTACTATGTAGTAGCTCATTTTCACTATGTTCTTTCTATAGGAGCTG
TATTTGCAATTATAGCCGGAATTGTCCAATGATTCCCATTATTTACCGGACTAAC
CTTAAATAATTTTTTTTTAAAAACCCAATTTATTACTATATTTTTAGGTGTAAATTTA
ACATTTTTTCCCCAACACTTTTTAGGGCTAAGAGGAATGCCTCGGCGATATTCGG
ATTATCCAGATGCTTTCACTATCTGAAATGCCATTTCATCTATTGGTTCCATTATT
TCTCTCACAAGAATTATCTTTTTTTTATTTATCTTATGAGAAAGGCTTTCTTCCCAA
CGTAAATCTTTGAGAACTTTAAATGTAACAACTTCAATTGAATGGTTACAAGCCCT
ACCCCCTAGTG
MOC2_0
ATTATTAGACAAGAAAGGGGGAAAAAAGAAGCTTTTGGAACCTTAGGAATAATTT
ATGCCATGATAGCTATCGGATTATTAGGATTCGTAGTTTGAGCTCACCATATATT
TACAGTAGGGATAGATGTAGATACCCGAGCTTACTTCACTTCAGCAACTATAATT
ATTGCGGTTCCAACTGGGATTAAAGTCTTTAGATGACTAGCCACCTTTCATGGAA
CTCAAATTTTAAATAGACCAGTAACCCTGTGAGCTTTAGGTTTTGTATTTTTATTT
ACAGTAGGAGGATTAACAGGGGTAATTTTAGCTAATTCTTCAATTGATATTATTCT
TCACGATACTTACTATGTAGTAGCTCATTTTCACTATGTTCTTTCTATAGGAGCTG
TATTTGCAATTATAGCCGGAATTGTCCAATGATTCCCATTATTTACCGGACTAAC
CTTAAATAATTTTTTTTTAAAAACCCAATTTATTACTATATTTTTAGGTGTAAATTTA
ACATTTTTTCCCCAACACTTTTTAGGGCTAAGAGGAATGCCTCGGCGATATTCGG
ATTATCCAGATGCTTTCACTATCTGAAATGCCATTTCATCTATTGGTTCCATTATT
TCTCTCACAAGAATTATCTTTTTTTTATTTATCTTATGAGAAAGGCTTTCTTCCCAA
CGTAAATCTTTGAGAACTTTAAATGTAACAACTTCAATTGAATGGTTACAAGCCCT
ACCCCCTAGTG
MOC2_1
ATTATTAGACAAGAAAGGGGGAAAAAAGAAGCTTTTGGAACCTTAGGAATAATTT
ATGCCATGATAGCTATCGGATTATTAGGATTCGTAGTTTGAGCTCACCATATATT
TACAGTAGGGATAGATGTAGATACCCGAGCTTACTTCACTTCAGCAACTATAATT
ATTGCGGTTCCAACTGGGATTAAAGTCTTTAGATGACTAGCCACCTTTCATGGAA
CTCAAATTTTAAATAGACCAGTAACCCTGTGAGCTTTAGGTTTTGTATTTTTATTT
ACAGTAGGAGGATTAACAGGGGTAATTTTAGCTAATTCTTCAATTGATATTATTCT
TCACGATACTTACTATGTAGTAGCTCATTTTCACTATGTTCTTTCTATAGGAGCTG
TATTTGCAATTATAGCCGGAATTGTCCAATGATTCCCATTATTTACCGGACTAAC
CTTAAATAATTTTTTTTTAAAAACCCAATTTATTACTATATTTTTAGGTGTAAATTTA
ACATTTTTTCCCCAACACTTTTTAGGGCTAAGAGGAATGCCTCGGCGATATTCGG
ATTATCCAGATGCTTTCACTATCTGAAATGCCATTTCATCTATTGGTTCCATTATT
TCTCTCACAAGAATTATCTTTTTTTTATTTATCTTATGAGAAAGGCTTTCTTCCCAA
CGTAAATCTTTGAGAACTTTAAATGTAACAACTTCAATTGAATGGTTACAAGCCCT
ACCCCCTAGTG
MOC3
ATTATTAGACAAGAAAGGGGGAAAAAAGAAGCTTTTGGAACCTTAGGAATAATTT
ATGCCATGATAGCTATCGGATTATTAGGATTCGTAGTTTGAGCTCACCATATATT
TACAGTAGGGATAGATGTAGATACCCGAGCTTACTTCACTTCAGCAACTATAATT
ATTGCGGTTCCAACTGGGATTAAAGTCTTTAGATGACTAGCCACCTTTCATGGAA
CTCAAATTTTAAATAGACCAGTAACCCTGTGAGCTTTAGGTTTTGTATTTTTATTT
ACAGTAGGAGGATTAACAGGGGTAATTTTAGCTAATTCTTCAATTGATATTATTCT
TCACGATACTTACTATGTAGTAGCTCATTTTCACTATGTTCTTTCTATAGGAGCTG
TATTTGCAATTATAGCCGGAATTGTCCAATGATTCCCATTATTTACCGGACTAAC
CTTAAATAATTTTTTTTTAAAAACCCAATTTATTACTATATTTTTAGGTGTAAATTTA
ACATTTTTTCCCCAACACTTTTTAGGGCTAAGAGGAATGCCTCGGCGATATTCGG
ATTATCCAGATGCTTTCACTATCTGAAATGCCATTTCATCTATTGGTTCCATTATT

TCTCTCACAAGAATTATCTTTTTTTTATTTATCTTATGAGAAAGGCTTTCTTCCCAA
CGTAAATCTTTGAGAACTTTAAATGTAACAACTTCAATTGAATGGTTACAAGCCCT
ACCCCCTAGTG
MOC4
ATTATTAGACAAGAAAGGGGGAAAAAAGAAGCTTTTGGAACCTTAGGAATAATTT
ATGCCATGATAGCTATCGGATTATTAGGATTCGTAGTTTGAGCTCACCATATATT
TACAGTAGGGATAGATGTAGATACCCGAGCTTACTTCACTTCAGCAACTATAATT
ATTGCGGTTCCAACTGGGATTAAAGTCTTTAGATGACTAGCCACCTTTCATGGAA
CTCAAATTTTAAATAGACCAGTAACCCTGTGAGCTTTAGGTTTTGTATTTTTATTT
ACAGTAGGAGGATTAACAGGGGTAATTTTAGCTAATTCTTCAATTGATATTATTCT
TCACGATACTTACTATGTAGTAGCTCATTTTCACTATGTTCTTTCTATAGGAGCTG
TATTTGCAATTATAGCCGGAATTGTCCAATGATTCCCATTATTTACCGGACTAAC
CTTAAATAATTTTTTTTTAAAAACCCAATTTATTACTATATTTTTAGGTGTAAATTTA
ACATTTTTTCCCCAACACTTTTTAGGGCTAAGAGGAATGCCTCGGCGATATTCGG
ATTATCCAGATGCTTTCACTATCTGAAATGCCATTTCATCTATTGGTTCCATTATT
TCTCTCACAAGAATTATCTTTTTTTTATTTATCTTATGAGAAAGGCTTTCTTCCCAA
CGTAAATCTTTGAGAACTTTAAATGTAACAACTTCAATTGAATGGTTACAAGCCCT
ACCCCCTAGTG
moc5
ATTATTAGACAAGAAAGGGGGAAAAAAGAAGCTTTTGGAACCTTAGGAATAATTT
ATGCCATGATAGCTATCGGATTATTAGGATTCGTAGTTTGAGCTCACCATATATT
TACAGTAGGGATAGATGTAGATACCCGAGCTTACTTCACTTCAGCAACTATAATT
ATTGCGGTTCCAACTGGGATTAAAGTCTTTAGATGACTAGCCACCTTTCATGGAA
CTCAAATTTTAAATAGACCAGTAACCCTGTGAGCTTTAGGTTTTGTATTTTTATTT
ACAGTAGGAGGATTAACAGGGGTAATTTTAGCTAATTCTTCAATTGATATTATTCT
TCACGATACTTACTATGTAGTAGCTCATTTTCACTATGTTCTTTCTATAGGAGCTG
TATTTGCAATTATAGCCGGAATTGTCCAATGATTCCCATTATTTACCGGACTAAC
CTTAAATAATTTTTTTTTAAAAACCCAATTTATTACTATATTTTTAGGTGTAAATTTA
ACATTTTTTCCCCAACACTTTTTAGGGCTAAGAGGAATGCCTCGGCGATATTCGG
ATTATCCAGATGCTTTCACTATCTGAAATGCCATTTCATCTATTGGTTCCATTATT
TCTCTCACAAGAATTATCTTTTTTTTATTTATCTTATGAGAAAGGCTTTCTTCCCAA
CGTAAATCTTTGAGAACTTTAAATGTAACAACTTCAATTGAATGGTTACAAGCCCT
ACCCCCTAGTG
pit1
ATTATTAGACAAGAAAGGGGGAAAAAAGAAGCTTTTGGAACCTTAGGAATAATTT
ATGCCATGATAGCTATCGGATTATTAGGATTCGTAGTTTGAGCTCACCATATATT
TACAGTAGGGATAGATGTAGATACCCGAGCTTACTTCACTTCAGCAACTATAATT
ATCGCGGTTCCAACTGGGATTAAAGTCTTTAGGTGACTAGCCACCTTTCATGGA
ACTCAAATTTTAAATAGACCAGTAACCCTGTGAGCTTTAGGTTTTGTATTTTTATT
TACAGTAGGAGGATTAACAGGGGTAATTTTAGCTAATTCTTCAATTGATATTATTC
TTCACGATACTTACTATGTAGTAGCTCATTTTCACTATGTTCTTTCTATAGGAGCT
GTATTTGCAATTATAGCCGGAATTGTCCAATGATTCCCATTATTTACTGGACTAA
CCTTAAATAATTTTTTTTTAAAAACCCAATTTATTACTATATTTTTAGGTGTAAATTT
AACATTTTTTCCTCAACACTTTTTAGGGCTAAGAGGAATGCCTCGGCGATATTCG
GATTATCCAGATGCTTTCACTATCTGAAATGCCATTTCATCTATTGGTTCCATTAT
TTCTCTCACAAGAATTATCTTTTTTTTATTTATCTTATGAGAAAGGCTTTCTTCCCA
ACGTAAATCTTTGAGAACTTTAAATGTAACAACTTCAATTGAATGGTTACAAGCC
CTACCCCCTAGTG
PIT1_1
ATTATTAGACAAGAAAGGGGGAAAAAAGAAGCTTTTGGAACCTTAGGAATAATTT

ATGCCATGATAGCTATCGGATTATTAGGATTCGTAGTTTGAGCTCACCATATATT
TACAGTAGGGATAGATGTAGATACCCGAGCTTACTTCACTTCAGCAACTATAATT
ATCGCGGTTCCAACTGGGATTAAAGTCTTTAGGTGACTAGCCACCTTTCATGGA
ACTCAAATTTTAAATAGACCAGTAACCCTGTGAGCTTTAGGTTTTGTATTTTTATT
TACAGTAGGAGGATTAACAGGGGTAATTTTAGCTAATTCTTCAATTGATATTATTC
TTCACGATACTTACTATGTAGTAGCTCATTTTCACTATGTTCTTTCTATAGGAGCT
GTATTTGCAATTATAGCCGGAATTGTCCAATGATTCCCATTATTTACTGGACTAA
CCTTAAATAATTTTTTTTTAAAAACCCAATTTATTACTATATTTTTAGGTGTAAATTT
AACATTTTTTCCTCAACACTTTTTAGGGCTAAGAGGAATGCCTCGGCGATATTCG
GATTATCCAGATGCTTTCACTATCTGAAATGCCATTTCATCTATTGGTTCCATTAT
TTCTCTCACAAGAATTATCTTTTTTTTTATTTATCTTATGAGAAAGGCTTTCTTCCCA
ACGTAAATCTTTGAGAACTTTAAATGTAACAACTTCAATTGAATGGTTACAAGCC
CTACCCCCTAGTG
PIT1_2
ATTATTAGACAAGAAAGGGGGAAAAAAGAAGCTTTTGGAACCTTAGGAATAATTT
ATGCCATGATAGCTATCGGATTATTAGGATTCGTAGTTTGAGCTCACCATATATT
TACAGTAGGGATAGATGTAGATACCCGAGCTTACTTCACTTCAGCAACTATAATT
ATTGCGGTTCCAACTGGGATTAAAGTCTTTAGATGACTAGCCACCTTTCATGGAA
CTCAAATTTTAAATAGACCAGTAACCCTGTGAGCTTTAGGTTTTGTATTTTTATTT
ACAGTAGGAGGATTAACAGGGGTAATTTTAGCTAATTCTTCAATTGATATTATTCT
TCACGATACTTACTATGTAGTAGCTCATTTTCACTATGTTCTTTCTATAGGAGCTG
TATTTGCAATTATAGCCGGAATTGTCCAATGATTCCCATTATTTACTGGACTAACC
TTAAATAATTTTTTTTTAAAAACCCAATTTATTACTATATTTTTAGGTGTAAATTTAA
CATTTTTTCCTCAACACTTTTTAGGGCTAAGAGGAATGCCTCGGCGATATTCGGA
TTATCCAGATGCTTTCACTATCTGAAATGCCATTTCATCTATTGGTTCCATTATTT
CTCTCACAAGAATTATCTTTTTTTTTATTTATCTTATGAGAAAGGCTTTCTTCCCAA
CGTAAATCTTTGAGAACTTTAAATGTAACAACTTCAATTGAATGGTTACAAGCCCT
ACCCCCTAGTG
pit2
ATTATTAGACAAGAAAGGGGGAAAAAAGAAGCTTTTGGAACCTTAGGAATAATTT
ATGCCATGATAGCTATCGGATTATTAGGATTCGTAGTTTGAGCTCACCATATATT
TACAGTAAGGATAGATGTAGATACCCGAGCTTACTTCACTTCAGCAACTATAATT
ATCGCGGTTCCAACTGGGATTAAAGTCTTTAGGTGACTAGCCACCTTTCATGGA
ACTCAAATTTTAAATAGACCAGTAACCCTGTGAGCTTTAGGTTTTGTATTTTTATT
TACAGTAGGAGGATTAACAGGGGTAATTTTAGCTAATTCTTCAATTGATATTATTC
TTCACGATACTTACTATGTAGTAGCTCATTTTCACTATGTTCTTTCTATAGGAGCT
GTATTTGCAATTATAGCCGGAATTGTCCAATGATTCCCATTATTTACTGGACTAA
CCTTAAATAATTTTTTTTTAAAAACCCAATTTATTACTATATTTTTAGGTGTAAATTT
AACATTTTTTCCTCAACACTTTTTAGGGCTAAGAGGAATGCCTCGGCGATATTCG
GATTATCCAGATGCTTTCACTATCTGAAATGCCATTTCATCTATTGGTTCCATTAT
TTCTCTCACAAGAATTATCTTTTTTTTTATTTATCTTATGAGAAAGGCTTTCTTCCCA
ACGTAAATCTTTGAGAACTTTAAATGTAACAACTTCAATTGAATGGTTACAAGCC
CTACCCCCTAGTG
pit4
ATTATTAGACAAGAAAGGGGGAAAAAAGAAGCTTTTGGAACCTTAGGAATAATTT
ATGCCATGATAGCTATCGGATTATTAGGATTCGTAGTTTGAGCTCACCATATATT
TACAGTAGGGATAGATGTAGATACCCGAGCTTACTTCACTTCAGCAACTATAATT
ATTGCGGTTCCAACTGGGATTAAAGTCTTTAGATGACTAGCCACCTTTCATGGAA
CTCAAATTTTAAATAGACCAGTAACCCTGTGAGCTTTAGGTTTTGTATTTTTATTT
ACAGTAGGAGGATTAACAGGGGTAATTTTAGCTAATTCTTCAATTGATATTATTCT

TCACGATACTTACTATGTAGTAGCTCATTTTCACTATGTTCTTTCTATAGGAGCTG
TATTTGCAATTATAGCCGGAATTGTCCAATGATTCCCATTATTTACTGGACTAACC
TTAAATAATTTTTTTTTAAAAACCCAATTTATTACTATATTTTTAGGTGTAAATTTAA
CATTTTTTCCTCAACACTTTTTAGGGCTAAGAGGAATGCCTCGGCGATATTCGGA
TTATCCAGATGCTTTCACTATCTGAAATGCCATTTCATCTATTGGTTCCATTATTT
CTCTCACAAGAATTATCTTTTTTTTATTTATCTTATGAGAAAGGCTTTCTTCCCAA
CGTAAATCTTTGAGAACTTTAAATGTAACAACTTCAATTGAATGGTTACAAGCCCT
ACCCCCTAGTG
RON1
ATTATTAGACAAGAAAGGGGGAAAAAAGAAGCTTTTGGAACCTTAGGAATAATTT
ATGCCATGATAGCTATCGGATTATTAGGATTCGTAGTTTGAGCTCACCATATATT
TACAGTAGGGATAGATGTAGATACCCGAGCTTACTTCACTTCAGCAACTATAATT
ATTGCGGTTCCAACTGGGATTAAAGTCTTTAGATGACTAGCCACCTTTCATGGAA
CTCAAATTTTAAATAGACCAGTAACCCTGTGAGCTTTAGGTTTTGTATTTTTATTT
ACAGTAGGAGGATTAACAGGGGTAATTTTAGCTAATTCTTCAATTGATATTATTCT
TCACGATACTTACTATGTAGTAGCTCATTTTCACTATGTTCTTTCTATAGGAGCTG
TATTTGCAATTATAGCCGGAATTGTCCAATGATTCCCATTATTTACCGGACTAAC
CTTAAATAATTTTTTTTTAAAAACCCAATTTATTACTATATTTTTAGGTGTAAATTTA
ACATTTTTTCCCCAACACTTTTTAGGGCTAAGAGGAATGCCTCGGCGATATTCGG
ATTATCCAGATGCTTTCACTATCTGAAATGCCATTTCATCTATTGGTTCCATTATT
TCTCTCACAAGAATTATCTTTTTTTTATTTATCTTATGAGAAAGGCTTTCTTCCCAA
CGTAAATCTTTGAGAACTTTAAATGTAACAACTTCAATTGAATGGTTACAAGCCCT
ACCCCCTAGTG
RON2
ATTATTAGACAAGAAAGGGGGAAAAAAGAAGCTTTTGGAACCTTAGGAATAATTT
ATGCCATGATAGCTATCGGATTATTAGGATTCGTAGTTTGAGCTCACCATATATT
TACAGTAGGGATAGATGTAGATACCCGAGCTTACTTCACTTCAGCAACTATAATT
ATTGCGGTTCCAACTGGGATTAAAGTCTTTAGATGACTAGCCACCTTTCATGGAA
CTCAAATTTTAAATAGACCAGTAACCCTGTGAGCTTTAGGTTTTGTATTTTTATTT
ACAGTAGGAGGATTAACAGGGGTAATTTTAGCTAATTCTTCAATTGATATTATTCT
TCACGATACTTACTATGTAGTAGCTCATTTTCACTATGTTCTTTCTATAGGAGCTG
TATTTGCAATTATAGCCGGAATTGTCCAATGATTCCCATTATTTACCGGACTAAC
CTTAAATAATTTTTTTTTAAAAACCCAATTTATTACTATATTTTTAGGTGTAAATTTA
ACATTTTTTCCCCAACACTTTTTAGGGCTAAGAGGAATGCCTCGGCGATATTCGG
ATTATCCAGATGCTTTCACTATCTGAAATGCCATTTCATCTATTGGTTCCATTATT
TCTCTCACAAGAATTATCTTTTTTTTATTTATCTTATGAGAAAGGCTTTCTTCCCAA
CGTAAATCTTTGAGAACTTTAAATGTAACAACTTCAATTGAATGGTTACAAGCCCT
ACCCCCTAGTG
ron3
ATTATTAGACAAGAAAGGGGGAAAAAAGAAGCTTTTGGAACCTTAGGAATAATTT
ATGCCATGATAGCTATCGGATTATTAGGATTCGTAGTTTGAGCTCACCATATATT
TACAGTAGGGATAGATGTAGATACCCGAGCTTACTTCACTTCAGCAACTATAATT
ATTGCGGTTCCAACTGGGATTAAAGTCTTTAGATGACTAGCCACCTTTCATGGAA
CTCAAATTTTAAATAGACCAGTAACCCTGTGAGCTTTAGGTTTTGTATTTTTATTT
ACAGTAGGAGGATTAACAGGGGTAATTTTAGCTAATTCTTCAATTGATATTATTCT
TCACGATACTTACTATGTAGTAGCTCATTTTCACTATGTTCTTTCTATAGGAGCTG
TATTTGCAATTATAGCCGGAATTGTCCAATGATTCCCATTATTTACCGGACTAAC
CTTAAATAATTTTTTTTTAAAAACCCAATTTATTACTATATTTTTAGGTGTAAATTTA
ACATTTTTTCCCCAACACTTTTTAGGGCTAAGAGGAATGCCTCGGCGATATTCGG
ATTATCCAGATGCTTTCACTATCTGAAATGCCATTTCATCTATTGGTTCCATTATT

TCTCTCACAAGAATTATCTTTTTTTTATTTATCTTATGAGAAAGGCTTTCTTCCCAA
CGTAAATCTTTGAGAACTTTAAATGTAACAACTTCAATTGAATGGTTACAAGCCCT
ACCCCCTAGTG
RON4
ATTATTAGACAAGAAAGGGGGAAAAAAGAAGCTTTTGGAACCTTAGGAATAATTT
ATGCCATGATAGCTATCGGATTATTAGGATTCGTAGTTTGAGCTCACCATATATT
TACAGTAGGGATAGATGTAGATACCCGAGCTTACTTCACTTCAGCAACTATAATT
ATTGCGGTTCCAACTGGGATTAAAGTCTTTAGATGACTAGCCACCTTTCATGGAA
CTCAAATTTTAAATAGACCAGTAACCCTGTGAGCTTTAGGTTTTGTATTTTTATTT
ACAGTAGGAGGATTAACAGGGGTAATTTTAGCTAATTCTTCAATTGATATTATTCT
TCACGATACTTACTATGTAGTAGCTCATTTTCACTATGTTCTTTCTATAGGAGCTG
TATTTGCAATTATAGCCGGAATTGTCCAATGATTCCCATTATTTACCGGACTAAC
CTTAAATAATTTTTTTTTAAAAACCCAATTTATTACTATATTTTTAGGTGTAAATTTA
ACATTTTTTCCCCAACACTTTTTAGGGCTAAGAGGAATGCCTCGGCGATATTCGG
ATTATCCAGATGCTTTCACTATCTGAAATGCCATTTCATCTATTGGTTCCATTATT
TCTCTCACAAGAATTATCTTTTTTTTATTTATCTTATGAGAAAGGCTTTCTTCCCAA
CGTAAATCTTTGAGAACTTTAAATGTAACAACTTCAATTGAATGGTTACAAGCCCT
ACCCCCTAGTG
RON7
ATTATTAGACAAGAAAGGGGGAAAAAAGAAGCTTTTGGAACCTTAGGAATAATTT
ATGCCATGATAGCTATCGGATTATTAGGATTCGTAGTTTGAGCTCACCATATATT
TACAGTAGGGATAGATGTAGATACCCGAGCTTACTTCACTTCAGCAACTATAATT
ATTGCGGTTCCAACTGGGATTAAAGTCTTTAGATGACTAGCCACCTTTCATGGAA
CTCAAATTTTAAATAGACCAGTAACCCTGTGAGCTTTAGGTTTTGTATTTTTATTT
ACAGTAGGAGGATTAACAGGGGTAATTTTAGCTAATTCTTCAATTGATATTATTCT
TCACGATACTTACTATGTAGTAGCTCATTTTCACTATGTTCTTTCTATAGGAGCTG
TATTTGCAATTATAGCCGGAATTGTCCAATGATTCCCATTATTTACCGGACTAAC
CTTAAATAATTTTTTTTTAAAAACCCAATTTATTACTATATTTTTAGGTGTAAATTTA
ACATTTTTTCCCCAACACTTTTTAGGGCTAAGAGGAATGCCTCGGCGATATTCGG
ATTATCCAGATGCTTTCACTATCTGAAATGCCATTTCATCTATTGGTTCCATTATT
TCTCTCACAAGAATTATCTTTTTTTTATTTATCTTATGAGAAAGGCTTTCTTCCCAA
CGTAAATCTTTGAGAACTTTAAATGTAACAACTTCAATTGAATGGTTACAAGCCCT
ACCCCCTAGTG
SGO1
ATTATTAGACAAGAAAGGGGGAAAAAAGAAGCTTTTGGAACCTTAGGAATAATTT
ATGCCATGATAGCTATCGGATTATTAGGATTCGTAGTTTGAGCTCACCATATATT
TACAGTAGGGATAGATGTAGATACCCGAGCTTACTTCACTTCAGCAACTATAATT
ATTGCGGTTCCAACTGGGATTAAAGTCTTTAGATGACTAGCCACCTTTCATGGAA
CTCAAATTTTAAATAGACCAGTAACCCTGTGAGCTTTAGGTTTTGTATTTTTATTT
ACAGTAGGAGGATTAACAGGGGTAATTTTAGCTAATTCTTCAATTGATATTATTCT
TCACGATACTTACTATGTAGTAGCTCATTTTCACTATGTTCTTTCTATAGGAGCTG
TATTTGCAATTATAGCCGGAATTGTCCAATGATTCCCATTATTTACCGGACTAAC
CTTAAATAATTTTTTTTTAAAAACCCAATTTATTACTATATTTTTAGGTGTAAATTTA
ACATTTTTTCCCCAACACTTTTTAGGGCTAAGAGGAATGCCTCGGCGATATTCGG
ATTATCCAGATGCTTTCACTATCTGAAATGCCATTTCATCTATTGGTTCCATTATT
TCTCTCACAAGAATTATCTTTTTTTTATTTATCTTATGAGAAAGGCTTTCTTCCCAA
CGTAAATCTTTGAGAACTTTAAATGTAACAACTTCAATTGAATGGTTACAAGCCCT
ACCCCCTAGTG
SGO2
ATTATTAGACAAGAAAGGGGGAAAAAAGAAGCTTTTGGAACCTTAGGAATAATTT

ATGCCATGATAGCTATCGGATTATTAGGATTCGTAGTTTGAGCTCACCATATATT
TACAGTAGGGATAGATGTAGATACCCGAGCTTACTTCACTTCAGCAACTATAATT
ATTGCGGTTCCAACTGGGATTAAAGTCTTTAGATGACTAGCCACCTTTCATGGAA
CTCAAATTTTAAATAGACCAGTAACCCTGTGAGCTTTAGGTTTTGTATTTTTATTT
ACAGTAGGAGGATTAACAGGGGTAATTTTAGCTAATTCTTCAATTGATATTATTCT
TCACGATACTTACTATGTAGTAGCTCATTTTCACTATGTTCTTTCTATAGGAGCTG
TATTTGCAATTATAGCCGGAATTGTCCAATGATTCCCATTATTTACCGGACTAAC
CTTAAATAATTTTTTTTTAAAAACCCAATTTATTACTATATTTTTAGGTGTAAATTTA
ACATTTTTTCCCCAACACTTTTTAGGGCTAAGAGGAATGCCTCGGCGATATTCGG
ATTATCCAGATGCTTTCACTATCTGAAATGCCATTTCATCTATTGGTTCCATTATT
TCTCTCACAAGAATTATCTTTTTTTTTATTTATCTTATGAGAAAGGCTTTCTTCCCAA
CGTAAATCTTTGAGAACTTTAAATGTAACAACTTCAATTGAATGGTTACAAGCCCT
ACCCCCTAGTG
SJA12
ATTATTAGACAAGAAAGGGGGAAAAAAGAAGCTTTTGGAACCTTAGGAATAATTT
ATGCCATGATAGCTATCGGATTATTAGGATTCGTAGTTTGAGCTCACCATATATT
TACAGTAGGGATAGATGTAGATACCCGAGCTTACTTCACTTCAGCAACTATAATT
ATTGCGGTTCCAACTGGGATTAAAGTCTTTAGATGACTAGCCACCTTTCATGGAA
CTCAAATTTTAAATAGACCAGTAACCCTGTGAGCTTTAGGTTTTGTATTTTTATTT
ACAGTAGGAGGATTAACAGGGGTAATTTTAGCTAATTCTTCAATTGATATTATTCT
TCACGATACTTACTATGTAGTAGCTCATTTTCACTATGTTCTTTCTATAGGAGCTG
TATTTGCAATTATAGCCGGAATTGTCCAATGATTCCCATTATTTACCGGACTAAC
CTTAAATAATTTTTTTTTAAAAACCCAATTTATTACTATATTTTTAGGTGTAAATTTA
ACATTTTTTCCCCAACACTTTTTAGGGCTAAGAGGAATGCCTCGGCGATATTCGG
ATTATCCAGATGCTTTCACTATCTGAAATGCCATTTCATCTATTGGTTCCATTATT
TCTCTCACAAGAATTATCTTTTTTTTTATTTATCTTATGAGAAAGGCTTTCTTCCCAA
CGTAAATCTTTGAGAACTTTAAATGTAACAACTTCAATTGAATGGTTACAAGCCCT
ACCCCCTAGTG
SJA13
ATTATTAGACAAGAAAGGGGGAAAAAAGAAGCTTTTGGAACCTTAGGAATAATTT
ATGCCATGATAGCTATCGGATTATTAGGATTCGTAGTTTGAGCTCACCATATATT
TACAGTAGGGATAGATGTAGATACCCGAGCTTACTTCACTTCAGCAACTATAATT
ATTGCGGTTCCAACTGGGATTAAAGTCTTTAGATGACTAGCCACCTTTCATGGAA
CTCAAATTTTAAATAGACCAGTAACCCTGTGAGCTTTAGGTTTTGTATTTTTATTT
ACAGTAGGAGGATTAACAGGGGTAATTTTAGCTAATTCTTCAATTGATATTATTCT
TCACGATACTTACTATGTAGTAGCTCATTTTCACTATGTTCTTTCTATAGGAGCTG
TATTTGCAATTATAGCCGGAATTGTCCAATGATTCCCATTATTTACCGGACTAAC
CTTAAATAATTTTTTTTTAAAAACCCAATTTATTACTATATTTTTAGGTGTAAATTTA
ACATTTTTTCCCCAACACTTTTTAGGGCTAAGAGGAATGCCTCGGCGATATTCGG
ATTATCCAGATGCTTTCACTATCTGAAATGCCATTTCATCTATTGGTTCCATTATT
TCTCTCACAAGAATTATCTTTTTTTTTATTTATCTTATGAGAAAGGCTTTCTTCCCAA
CGTAAATCTTTGAGAACTTTAAATGTAACAACTTCAATTGAATGGTTACAAGCCCT
ACCCCCTAGTG
SJA14
ATTATTAGACAAGAAAGGGGGAAAAAAGAAGCTTTTGGAACCTTAGGAATAATTT
ATGCCATGATAGCTATCGGATTATTAGGATTCGTAGTTTGAGCTCACCATATATT
TACAGTAGGGATAGATGTAGATACCCGAGCTTACTTCACTTCAGCAACTATAATT
ATTGCGGTTCCAACTGGGATTAAAGTCTTTAGATGACTAGCCACCTTTCATGGAA
CTCAAATTTTAAATAGACCAGTAACCCTGTGAGCTTTAGGTTTTGTATTTTTATTT
ACAGTAGGAGGATTAACAGGGGTAATTTTAGCTAATTCTTCAATTGATATTATTCT

TCACGATACTTACTATGTAGTAGCTCATTTTCACTATGTTCTTTCTATAGGAGCTG
TATTTGCAATTATAGCCGGAATTGTCCAATGATTCCCATTATTTACCGGACTAAC
CTTAAATAATTTTTTTTTAAAAACCCAATTTATTACTATATTTTTAGGTGTAAATTTA
ACATTTTTTCCCCAACACTTTTTAGGGCTAAGAGGAATGCCTCGGCGATATTCGG
ATTATCCAGATGCTTTCACTATCTGAAATGCCATTTCATCTATTGGTTCCATTATT
TCTCTCACAAGAATTATCTTTTTTTTATTTATCTTATGAGAAAGGCTTTCTTCCCAA
CGTAAATCTTTGAGAACTTTAAATGTAACAACTTCAATTGAATGGTTACAAGCCCT
ACCCCCTAGTG
SJA17
ATTATTAGACAAGAAAGGGGGAAAAAAGAAGCTTTTGGAACCTTAGGAATAATTT
ATGCCATGATAGCTATCGGATTATTAGGATTCGTAGTTTGAGCTCACCATATATT
TACAGTAGGGATAGATGTAGATACCCGAGCTTACTTCACTTCAGCAACTATAATT
ATTGCGGTTCCAACTGGGATTAAAGTCTTTAGATGACTAGCCACCTTTCATGGAA
CTCAAATTTTAAATAGACCAGTAACCCTGTGAGCTTTAGGTTTTGTATTTTTATTT
ACAGTAGGAGGATTAACAGGGGTAATTTTAGCTAATTCTTCAATTGATATTATTCT
TCACGATACTTACTATGTAGTAGCTCATTTTCACTATGTTCTTTCTATAGGAGCTG
TATTTGCAATTATAGCCGGAATTGTCCAATGATTCCCATTATTTACCGGACTAAC
CTTAAATAATTTTTTTTTAAAAACCCAATTTATTACTATATTTTTAGGTGTAAATTTA
ACATTTTTTCCCCAACACTTTTTAGGGCTAAGAGGAATGCCTCGGCGATATTCGG
ATTATCCAGATGCTTTCACTATCTGAAATGCCATTTCATCTATTGGTTCCATTATT
TCTCTCACAAGAATTATCTTTTTTTTATTTATCTTATGAGAAAGGCTTTCTTCCCAA
CGTAAATCTTTGAGAACTTTAAATGTAACAACTTCAATTGAATGGTTACAAGCCCT
ACCCCCTAGTG
SJA3
ATTATTAGACAAGAAAGGGGGAAAAAAGAAGCTTTTGGAACCTTAGGAATAATTT
ATGCCATGATAGCTATCGGATTATTAGGATTCGTAGTTTGAGCTCACCATATATT
TACAGTAGGGATAGATGTAGATACCCGAGCTTACTTCACTTCAGCAACTATAATT
ATTGCGGTTCCAACTGGGATTAAAGTCTTTAGATGACTAGCCACCTTTCATGGAA
CTCAAATTTTAAATAGACCAGTAACCCTGTGAGCTTTAGGTTTTGTATTTTTATTT
ACAGTAGGAGGATTAACAGGGGTAATTTTAGCTAATTCTTCAATTGATATTATTCT
TCACGATACTTACTATGTAGTAGCTCATTTTCACTATGTTCTTTCTATAGGAGCTG
TATTTGCAATTATAGCCGGAATTGTCCAATGATTCCCATTATTTACCGGACTAAC
CTTAAATAATTTTTTTTTAAAAACCCAATTTATTACTATATTTTTAGGTGTAAATTTA
ACATTTTTTCCCCAACACTTTTTAGGGCTAAGAGGAATGCCTCGGCGATATTCGG
ATTATCCAGATGCTTTCACTATCTGAAATGCCATTTCATCTATTGGTTCCATTATT
TCTCTCACAAGAATTATCTTTTTTTTATTTATCTTATGAGAAAGGCTTTCTTCCCAA
CGTAAATCTTTGAGAACTTTAAATGTAACAACTTCAATTGAATGGTTACAAGCCCT
ACCCCCTAGTG
SJA4
ATTATTAGACAAGAAAGGGGGAAAAAAGAAGCTTTTGGAACCTTAGGAATAATTT
ATGCCATGATAGCTATCGGATTATTAGGATTCGTAGTTTGAGCTCACCATATATT
TACAGTAGGGATAGATGTAGATACCCGAGCTTACTTCACTTCAGCAACTATAATT
ATTGCGGTTCCAACTGGGATTAAAGTCTTTAGATGACTAGCCACCTTTCATGGAA
CTCAAATTTTAAATAGACCAGTAACCCTGTGAGCTTTAGGTTTTGTATTTTTATTT
ACAGTAGGAGGATTAACAGGGGTAATTTTAGCTAATTCTTCAATTGATATTATTCT
TCACGATACTTACTATGTAGTAGCTCATTTTCACTATGTTCTTTCTATAGGAGCTG
TATTTGCAATTATAGCCGGAATTGTCCAATGATTCCCATTATTTACCGGACTAAC
CTTAAATAATTTTTTTTTAAAAACCCAATTTATTACTATATTTTTAGGTGTAAATTTA
ACATTTTTTCCCCAACACTTTTTAGGGCTAAGAGGAATGCCTCGGCGATATTCGG
ATTATCCAGATGCTTTCACTATCTGAAATGCCATTTCATCTATTGGTTCCATTATT

TCTCTCACAAGAATTATCTTTTTTTTATTTATCTTATGAGAAAGGCTTTCTTCCCAA
CGTAAATCTTTGAGAACTTTAAATGTAACAACTTCAATTGAATGGTTACAAGCCCT
ACCCCCTAGTG
SJA5
ATTATTAGACAAGAAAGGGGGAAAAAAGAAGCTTTTGGAACCTTAGGAATAATTT
ATGCCATGATAGCTATCGGATTATTAGGATTCGTAGTTTGAGCTCACCATATATT
TACAGTAGGGATAGATGTAGATACCCGAGCTTACTTCACTTCAGCAACTATAATT
ATTGCGGTTCCAACTGGGATTAAAGTCTTTAGATGACTAGCCACCTTTCATGGAA
CTCAAATTTTAAATAGACCAGTAACCCTGTGAGCTTTAGGTTTTGTATTTTTATTT
ACAGTAGGAGGATTAACAGGGGTAATTTTAGCTAATTCTTCAATTGATATTATTCT
TCACGATACTTACTATGTAGTAGCTCATTTTCACTATGTTCTTTCTATAGGAGCTG
TATTTGCAATTATAGCCGGAATTGTCCAATGATTCCCATTATTTACCGGACTAAC
CTTAAATAATTTTTTTTTAAAAACCCAATTTATTACTATATTTTTAGGTGTAAATTTA
ACATTTTTTCCCCAACACTTTTTAGGGCTAAGAGGAATGCCTCGGCGATATTCGG
ATTATCCAGATGCTTTCACTATCTGAAATGCCATTTCATCTATTGGTTCCATTATT
TCTCTCACAAGAATTATCTTTTTTTTATTTATCTTATGAGAAAGGCTTTCTTCCCAA
CGTAAATCTTTGAGAACTTTAAATGTAACAACTTCAATTGAATGGTTACAAGCCCT
ACCCCCTAGTG
SMA1
ATTATTAGACAAGAAAGGGGGAAAAAAGAAGCTTTTGGAACCTTAGGAATAATTT
ATGCCATGATAGCTATCGGATTATTAGGATTCGTAGTTTGAGCTCACCATATATT
TACAGTAGGGATAGATGTAGATACCCGAGCTTACTTCACTTCAGCAACTATAATT
ATTGCGGTTCCAACTGGGATTAAAGTCTTTAGATGACTAGCCACCTTTCATGGAA
CTCAAATTTTAAATAGACCAGTAACCCTGTGAGCTTTAGGTTTTGTATTTTTATTT
ACAGTAGGAGGATTAACAGGGGTAATTTTAGCTAATTCTTCAATTGATATTATTCT
TCACGATACTTACTATGTAGTAGCTCATTTTCACTATGTTCTTTCTATAGGAGCTG
TATTTGCAATTATAGCCGGAATTGTCCAATGATTCCCATTATTTACCGGACTAAC
CTTAAATAATTTTTTTTTAAAAACCCAATTTATTACTATATTTTTAGGTGTAAATTTA
ACATTTTTTCCCCAACACTTTTTAGGGCTAAGAGGAATGCCTCGGCGATATTCGG
ATTATCCAGATGCTTTCACTATCTGAAATGCCATTTCATCTATTGGTTCCATTATT
TCTCTCACAAGAATTATCTTTTTTTTATTTATCTTATGAGAAAGGCTTTCTTCCCAA
CGTAAATCTTTGAGAACTTTAAATGTAACAACTTCAATTGAATGGTTACAAGCCCT
ACCCCCTAGTG
SMA10
ATTATTAGACAAGAAAGGGGGAAAAAAGAAGCTTTTGGAACCTTAGGAATAATTT
ATGCCATGATAGCTATCGGATTATTAGGATTCGTAGTTTGAGCTCACCATATATT
TACAGTAGGGATAGATGTAGATACCCGAGCTTACTTCACTTCAGCAACTATAATT
ATTGCGGTTCCAACTGGGATTAAAGTCTTTAGATGACTAGCCACCTTTCATGGAA
CTCAAATTTTAAATAGACCAGTAACCCTGTGAGCTTTAGGTTTTGTATTTTTATTT
ACAGTAGGAGGATTAACAGGGGTAATTTTAGCTAATTCTTCAATTGATATTATTCT
TCACGATACTTACTATGTAGTAGCTCATTTTCACTATGTTCTTTCTATAGGAGCTG
TATTTGCAATTATAGCCGGAATTGTCCAATGATTCCCATTATTTACCGGACTAAC
CTTAAATAATTTTTTTTTAAAAACCCAATTTATTACTATATTTTTAGGTGTAAATTTA
ACATTTTTTCCCCAACACTTTTTAGGGCTAAGAGGAATGCCTCGGCGATATTCGG
ATTATCCAGATGCTTTCACTATCTGAAATGCCATTTCATCTATTGGTTCCATTATT
TCTCTCACAAGAATTATCTTTTTTTTATTTATCTTATGAGAAAGGCTTTCTTCCCAA
CGTAAATCTTTGAGAACTTTAAATGTAACAACTTCAATTGAATGGTTACAAGCCCT
ACCCCCTAGTG
SMA11
ATTATTAGACAAGAAAGGGGGAAAAAAGAAGCTTTTGGAACCTTAGGAATAATTT

ATGCCATGATAGCTATCGGATTATTAGGATTCGTAGTTTGAGCTCACCATATATT
TACAGTAGGGATAGATGTAGATACCCGAGCTTACTTCACTTCAGCAACTATAATT
ATTGCGGTTCCAACTGGGATTAAAGTCTTTAGATGACTAGCCACCTTTCATGGAA
CTCAAATTTTAAATAGACCAGTAACCCTGTGAGCTTTAGGTTTTGTATTTTTATTT
ACAGTAGGAGGATTAACAGGGGTAATTTTAGCTAATTCTTCAATTGATATTATTCT
TCACGATACTTACTATGTAGTAGCTCATTTTCACTATGTTCTTTCTATAGGAGCTG
TATTTGCAATTATAGCCGGAATTGTCCAATGATTCCCATTATTTACCGGACTAAC
CTTAAATAATTTTTTTTTAAAAACCCAATTTATTACTATATTTTTAGGTGTAAATTTA
ACATTTTTTCCCCAACACTTTTTAGGGCTAAGAGGAATGCCTCGGCGATATTCGG
ATTATCCAGATGCTTTCACTATCTGAAATGCCATTTCATCTATTGGTTCCATTATT
TCTCTCACAAGAATTATCTTTTTTTTATTTATCTTATGAGAAAGGCTTTCTTCCCAA
CGTAAATCTTTGAGAACTTTAAATGTAACAACTTCAATTGAATGGTTACAAGCCCT
ACCCCCTAGTG
SMA12
ATTATTAGACAAGAAAGGGGGAAAAAAGAAGCTTTTGGAACCTTAGGAATAATTT
ATGCCATGATAGCTATCGGATTATTAGGATTCGTAGTTTGAGCTCACCATATATT
TACAGTAGGGATAGATGTAGATACCCGAGCTTACTTCACTTCAGCAACTATAATT
ATTGCGGTTCCAACTGGGATTAAAGTCTTTAGATGACTAGCCACCTTTCATGGAA
CTCAAATTTTAAATAGACCAGTAACCCTGTGAGCTTTAGGTTTTGTATTTTTATTT
ACAGTAGGAGGATTAACAGGGGTAATTTTAGCTAATTCTTCAATTGATATTATTCT
TCACGATACTTACTATGTAGTAGCTCATTTTCACTATGTTCTTTCTATAGGAGCTG
TATTTGCAATTATAGCCGGAATTGTCCAATGATTCCCATTATTTACCGGACTAAC
CTTAAATAATTTTTTTTTAAAAACCCAATTTATTACTATATTTTTAGGTGTAAATTTA
ACATTTTTTCCCCAACACTTTTTAGGGCTAAGAGGAATGCCTCGGCGATATTCGG
ATTATCCAGATGCTTTCACTATCTGAAATGCCATTTCATCTATTGGTTCCATTATT
TCTCTCACAAGAATTATCTTTTTTTTATTTATCTTATGAGAAAGGCTTTCTTCCCAA
CGTAAATCTTTGAGAACTTTAAATGTAACAACTTCAATTGAATGGTTACAAGCCCT
ACCCCCTAGTG
SMA13
ATTATTAGACAAGAAAGGGGGAAAAAAGAAGCTTTTGGAACCTTAGGAATAATTT
ATGCCATGATAGCTATCGGATTATTAGGATTCGTAGTTTGAGCTCACCATATATT
TACAGTAGGGATAGATGTAGATACCCGAGCTTACTTCACTTCAGCAACTATAATT
ATTGCGGTTCCAACTGGGATTAAAGTCTTTAGATGACTAGCCACCTTTCATGGAA
CTCAAATTTTAAATAGACCAGTAACCCTGTGAGCTTTAGGTTTTGTATTTTTATTT
ACAGTAGGAGGATTAACAGGGGTAATTTTAGCTAATTCTTCAATTGATATTATTCT
TCACGATACTTACTATGTAGTAGCTCATTTTCACTATGTTCTTTCTATAGGAGCTG
TATTTGCAATTATAGCCGGAATTGTCCAATGATTCCCATTATTTACCGGACTAAC
CTTAAATAATTTTTTTTTAAAAACCCAATTTATTACTATATTTTTAGGTGTAAATTTA
ACATTTTTTCCCCAACACTTTTTAGGGCTAAGAGGAATGCCTCGGCGATATTCGG
ATTATCCAGATGCTTTCACTATCTGAAATGCCATTTCATCTATTGGTTCCATTATT
TCTCTCACAAGAATTATCTTTTTTTTATTTATCTTATGAGAAAGGCTTTCTTCCCAA
CGTAAATCTTTGAGAACTTTAAATGTAACAACTTCAATTGAATGGTTACAAGCCCT
ACCCCCTAGTG
SMA14
ATTATTAGACAAGAAAGGGGGAAAAAAGAAGCTTTTGGAACCTTAGGAATAATTT
ATGCCATGATAGCTATCGGATTATTAGGATTCGTAGTTTGAGCTCACCATATATT
TACAGTAGGGATAGATGTAGATACCCGAGCTTACTTCACTTCAGCAACTATAATT
ATTGCGGTTCCAACTGGGATTAAAGTCTTTAGATGACTAGCCACCTTTCATGGAA
CTCAAATTTTAAATAGACCAGTAACCCTGTGAGCTTTAGGTTTTGTATTTTTATTT
ACAGTAGGAGGATTAACAGGGGTAATTTTAGCTAATTCTTCAATTGATATTATTCT

TCACGATACTTACTATGTAGTAGCTCATTTTCACTATGTTCCTTCTATAGGAGCTG
TATTTGCAATTATAGCCGGAATTGTCCAATGATTCCCATTATTTACCGGACTAAC
CTTAAATAATTTTTTTTTAAAAACCCAATTTATTACTATATTTTTAGGTGTAAATTTA
ACATTTTTTCCCCAACACTTTTTAGGGCTAAGAGGAATGCCTCGGCGATATTCGG
ATTATCCAGATGCTTTCACTATCTGAAATGCCATTTCATCTATTGGTTCCATTATT
TCTCTCACAAGAATTATCTTTTTTTTATTTATCTTATGAGAAAGGCTTTCTTCCCAA
CGTAAATCTTTGAGAACTTTAAATGTAACAACTTCAATTGAATGGTTACAAGCCCT
ACCCCCTAGTG
SMA6
ATTATTAGACAAGAAAGGGGGAAAAAAGAAGCTTTTGGAACCTTAGGAATAATTT
ATGCCATGATAGCTATCGGATTATTAGGATTCGTAGTTTGAGCTCACCATATATT
TACAGTAGGGATAGATGTAGATACCCGAGCTTACTTCACTTCAGCAACTATAATT
ATTGCGGTTCCAACTGGGATTAAAGTCTTTAGATGACTAGCCACCTTTCATGGAA
CTCAAATTTTAAATAGACCAGTAACCCTGTGAGCTTTAGGTTTTGTATTTTTATTT
ACAGTAGGAGGATTAACAGGGGTAATTTTAGCTAATTCTTCAATTGATATTATTCT
TCACGATACTTACTATGTAGTAGCTCATTTTCACTATGTTCTTTCTATAGGAGCTG
TATTTGCAATTATAGCCGGAATTGTCCAATGATTCCCATTATTTACCGGACTAAC
CTTAAATAATTTTTTTTTAAAAACCCAATTTATTACTATATTTTTAGGTGTAAATTTA
ACATTTTTTCCCCAACACTTTTTAGGGCTAAGAGGAATGCCTCGGCGATATTCGG
ATTATCCAGATGCTTTCACTATCTGAAATGCCATTTCATCTATTGGTTCCATTATT
TCTCTCACAAGAATTATCTTTTTTTTATTTATCTTATGAGAAAGGCTTTCTTCCCAA
CGTAAATCTTTGAGAACTTTAAATGTAACAACTTCAATTGAATGGTTACAAGCCCT
ACCCCCTAGTG
SMI1
ATTATTAGACAAGAAAGGGGGAAAAAAGAAGCTTTTGGAACCTTAGGAATAATTT
ATGCCATGATAGCTATCGGATTATTAGGATTCGTAGTTTGAGCTCACCATATATT
TACAGTAGGGATAGATGTAGATACCCGAGCTTACTTCACTTCAGCAACTATAATT
ATTGCGGTTCCAACTGGGATTAAAGTCTTTAGATGACTAGCCACCTTTCATGGAA
CTCAAATTTTAAATAGACCAGTAACCCTGTGAGCTTTAGGTTTTGTATTTTTATTT
ACAGTAGGAGGATTAACAGGGGTAATTTTAGCTAATTCTTCAATTGATATTATTCT
TCACGATACTTACTATGTAGTAGCTCATTTTCACTATGTTCTTTCTATAGGAGCTG
TATTTGCAATTATAGCCGGAATTGTCCAATGATTCCCATTATTTACCGGACTAAC
CTTAAATAATTTTTTTTTAAAAACCCAATTTATTACTATATTTTTAGGTGTAAATTTA
ACATTTTTTCCCCAACACTTTTTAGGGCTAAGAGGAATGCCTCGGCGATATTCGG
ATTATCCAGATGCTTTCACTATCTGAAATGCCATTTCATCTATTGGTTCCATTATT
TCTCTCACAAGAATTATCTTTTTTTTATTTATCTTATGAGAAAGGCTTTCTTCCCAA
CGTAAATCTTTGAGAACTTTAAATGTAACAACTTCAATTGAATGGTTACAAGCCCT
ACCCCCTAGTG
SMI2
ATTATTAGACAAGAAAGGGGGAAAAAAGAAGCTTTTGGAACCTTAGGAATAATTT
ATGCCATGATAGCTATCGGATTATTAGGATTCGTAGTTTGAGCTCACCATATATT
TACAGTAGGGATAGATGTAGATACCCGAGCTTACTTCACTTCAGCAACTATAATT
ATTGCGGTTCCAACTGGGATTAAAGTCTTTAGATGACTAGCCACCTTTCATGGAA
CTCAAATTTTAAATAGACCAGTAACCCTGTGAGCTTTAGGTTTTGTATTTTTATTT
ACAGTAGGAGGATTAACAGGGGTAATTTTAGCTAATTCTTCAATTGATATTATTCT
TCACGATACTTACTATGTAGTAGCTCATTTTCACTATGTTCTTTCTATAGGAGCTG
TATTTGCAATTATAGCCGGAATTGTCCAATGATTCCCATTATTTACCGGACTAAC
CTTAAATAATTTTTTTTTAAAAACCCAATTTATTACTATATTTTTAGGTGTAAATTTA
ACATTTTTTCCCCAACACTTTTTAGGGCTAAGAGGAATGCCTCGGCGATATTCGG
ATTATCCAGATGCTTTCACTATCTGAAATGCCATTTCATCTATTGGTTCCATTATT

TCTCTCACAAGAATTATCTTTTTTTTATTTATCTTATGAGAAAGGCTTTCTTCCCAA
CGTAAATCTTTGAGAACTTTAAATGTAACAACTTCAATTGAATGGTTACAAGCCCT
ACCCCCTAGTG
sob1
ATTATTAGACAAGAAAGGGGGAAAAAAGAAGCTTTTGGAACCTTAGGAATAATTT
ATGCCATGATAGCTATCGGATTATTAGGATTCGTAGTTTGAGCTCACCATATATT
TACAGTAGGGATAGATGTAGATACCCGAGCTTACTTCACTTCAGCAACTATAATT
ATTGCGGTTCCAACTGGGATTAAAGTCTTTAGATGACTAGCCACCTTTCATGGAA
CTCAAATTTTAAATAGACCAGTAACCCTGTGAGCTTTAGGTTTTGTATTTTTATTT
ACAGTAGGAGGATTAACGGGGGTAATTTTAGCTAATTCTTCAATTGATATTATTC
TTCACGATACTTACTATGTAGTAGCTCATTTTCACTATGTTCTTTCTATAGGAGCT
GTATTTGCAATTATAGCCGGAATTGTCCAATGATTCCCATTATTTACCGGACTAA
CCTTAAATAATTTTTTTTTAAAAACCCAATTTATTACTATATTTTTAGGTGTAAATTT
AACATTTTTTCCCCAACACTTTTTAGGGCTAAGAGGAATGCCTCGGCGATATTCG
GATTATCCAGATGCTTTCACTATCTGAAATGCCATTTCATCTATTGGTTCCATTAT
TTCTCTCACAAGAATTATCTTTTTTTTATTTATCTTATGAGAAAGGCTTTCTTCCCA
ACGTAAATCTTTGAGAACTTTAAATGTAACAACTTCAATTGAATGGTTACAAGCC
CTACCCCCTAGTG
SOB10
ATTATTAGACAAGAAAGGGGGAAAAAAGAAGCTTTTGGAACCTTAGGAATAATTT
ATGCCATGATAGCTATCGGATTATTAGGATTCGTAGTTTGAGCTCACCATATATT
TACAGTAGGGATAGATGTAGATACCCGAGCTTACTTCACTTCAGCAACTATAATT
ATCGCGGTTCCAACTGGGATTAAAGTCTTTAGGTGACTAGCCACCTTTCATGGA
ACTCAAATTTTAAATAGACCAGTAACCCTGTGAGCTTTAGGTTTTGTATTTTTATT
TACAGTAGGAGGATTAACAGGGGTAATTTTAGCTAATTCTTCAATTGATATTATTC
TTCACGATACTTACTATGTAGTAGCTCATTTTCACTATGTTCTTTCTATAGGAGCT
GTATTTGCAATTATAGCCGGAATTGTCCAATGATTCCCATTATTTACTGGACTAA
CCTTAAATAATTTTTTTTTAAAAACCCAATTTATTACTATATTTTTAGGTGTAAATTT
AACATTTTTTCCTCAACACTTTTTAGGGCTAAGAGGGATGCCTCGGCGATATTCG
GATTATCCAGATGCTTTCACTATCTGAAATGCCATTTCATCTATTGGTTCCATTAT
TTCTCTCACAAGAATTATCTTTTTTTTATTTATCTTATGAGAAAGGCTTTCTTCCCA
ACGTAAATCTTTGAGAACTTTAAATGTAACAACTTCAATTGAATGGTTACAAGCC
CTACCCCCTAGTG
SOB12
ATTATTAGACAAGAAAGGGGGAAAAAAGAAGCTTTTGGAACCTTAGGAATAATTT
ATGCCATGATAGCTATCGGATTATTAGGATTCGTAGTTTGAGCTCACCATATATT
TACAGTAGGGATAGATGTAGATACCCGAGCTTACTTCACTTCAGCAACTATAATT
ATCGCGGTTCCAACTGGGATTAAAGTCTTTAGGTGACTAGCCACCTTTCATGGA
ACTCAAATTTTAAATAGACCAGTAACCCTGTGAGCTTTAGGTTTTGTATTTTTATT
TACAGTAGGAGGATTAACAGGGGTAATTTTAGCTAATTCTTCAATTGATATTATTC
TTCACGATACTTACTATGTAGTAGCTCATTTTCACTATGTTCTTTCTATAGGAGCT
GTATTTGCAATTATAGCCGGAATTGTCCAATGATTCCCATTATTTACTGGACTAA
CCTTAAATAATTTTTTTTTAAAAACCCAATTTATTACTATATTTTTAGGTGTAAATTT
AACATTTTTTCCTCAACACTTTTTAGGGCTAAGAGGAATGCCTCGGCGATATTCG
GATTATCCAGATGCTTTCACTATCTGAAATGCCATTTCATCTATTGGTTCCATTAT
TTCTCTCACAAGAATTATCTTTTTTTTATTTATCTTATGAGAAAGGCTTTCTTCCCA
ACGTAAATCTTTGAGAACTTTAAATGTAACAACTTCAATTGAATGGTTACAAGCC
CTACCCCCTAGTG
SOB1_2
ATTATTAGACAAGAAAGGGGGAAAAAAGAAGCTTTTGGAACCTTAGGAATAATTT

ATGCCATGATAGCTATCGGATTATTAGGATTCGTAGTTTGAGCTCACCATATATT
TACAGTAGGGATAGATGTAGATACCCGAGCTTACTTCACTTCAGCAACTATAATT
ATCGCGGTTCCAACTGGGATTAAAGTCTTTAGGTGACTAGCCACCTTTCATGGA
ACTCAAATTTTAAATAGACCAGTAACCCTGTGAGCTTTAGGTTTTGTATTTTTATT
TACAGTAGGAGGATTAACAGGGGTAATTTTAGCTAATTCTTCAATTGATATTATTC
TTCACGATACTTACTATGTAGTAGCTCATTTTCACTATGTTCTTTCTATAGGAGCT
GTATTTGCAATTATAGCCGGAATTGTCCAATGATTCCCATTATTTACTGGACTAA
CCTTAAATAATTTTTTTTTAAAAACCCAATTTATTACTATATTTTTAGGTGTAAATTT
AACATTTTTTCCTCAACACTTTTTAGGGCTAAGAGGAATGCCTCGGCGATATTCG
GATTATCCAGATGCTTTCACTATCTGAAATGCCATTTCATCTATTGGTTCCATTAT
TTCTCTCACAAGAATTATCTTTTTTTTATTTATCTTATGAGAAAGGCTTTCTTCCCA
ACGTAAATCTTTGAGAACTTTAAATGTAACAACTTCAATTGAATGGTTACAAGCC
CTACCCCCTAGTG
SOB2
ATTATTAGACAAGAAAGGGGGAAAAAAGAAGCTTTTGGAACCTTAGGAATAATTT
ATGCCATGATAGCTATCGGATTATTAGGATTCGTAGTTTGAGCTCACCATATATT
TACAGTAGGGATAGATGTAGATACCCGAGCTTACTTCACTTCAGCAACTATAATT
ATTGCGGTTCCAACTGGGATTAAAGTCTTTAGATGACTAGCCACCTTTCATGGAA
CTCAAATTTTAAATAGACCAGTAACCCTGTGAGCTTTAGGTTTTGTATTTTTATTT
ACAGTAGGAGGATTAACGGGGGTAATTTTAGCTAATTCTTCAATTGATATTATTC
TTCACGATACTTACTATGTAGTAGCTCATTTTCACTATGTTCTTTCTATAGGAGCT
GTATTTGCAATTATAGCCGGAATTGTCCAATGATTCCCATTATTTACCGGACTAA
CCTTAAATAATTTTTTTTTAAAAACCCAATTTATTACTATATTTTTAGGTGTAAATTT
AACATTTTTTCCCCAACACTTTTTAGGGCTAAGAGGAATGCCTCGGCGATATTCG
GATTATCCAGATGCTTTCACTATCTGAAATGCCATTTCATCTATTGGTTCCATTAT
TTCTCTCACAAGAATTATCTTTTTTTTATTTATCTTATGAGAAAGGCTTTCTTCCCA
ACGTAAATCTTTGAGAACTTTAAATGTAACAACTTCAATTGAATGGTTACAAGCC
CTACCCCCTAGTG
SOB2_1
ATTATTAGACAAGAAAGGGGGAAAAAAGAAGCTTTTGGAACCTTAGGAATAATTT
ATGCCATGATAGCTATCGGATTATTAGGATTCGTAGTTTGAGCTCACCATATATT
TACAGTAGGGATAGATGTAGATACCCGAGCTTACTTCACTTCAGCAACTATAATT
ATCGCGGTTCCAACTGGGATTAAAGTCTTTAGGTGACTAGCCACCTTTCATGGA
ACTCAAATTTTAAATAGACCAGTAACCCTGTGAGCTTTAGGTTTTGTATTTTTATT
TACAGTAGGAGGATTAACAGGGGTAATTTTAGCTAATTCTTCAATTGATATTATTC
TTCACGATACTTACTATGTAGTAGCTCATTTTCACTATGTTCTTTCTATAGGAGCT
GTATTTGCAATTATAGCCGGAATTGTCCAATGATTCCCATTATTTACTGGACTAA
CCTTAAATAATTTTTTTTTAAAAACCCAATTTATTACTATATTTTTAGGTGTAAATTT
AACATTTTTTCCTCAACACTTTTTAGGGCTAAGAGGAATGCCTCGGCGATATTCG
GATTATCCAGATGCTTTCACTATCTGAAATGCCATTTCATCTATTGGTTCCATTAT
TTCTCTCACAAGAATTATCTTTTTTTTATTTATCTTATGAGAAAGGCTTTCTTCCCA
ACGTAAATCTTTGAGAACTTTAAATGTAACAACTTCAATTGAATGGTTACAAGCC
CTACCCCCTAGTG
TAP1
ATTATTAGACAAGAAAGGGGGAAAAAAGAAGCTTTTGGAACCTTAGGAATAATTT
ATGCCATGATAGCTATCGGATTATTAGGATTCGTAGTTTGAGCTCACCATATATT
TACAGTAGGGATAGATGTAGATACCCGAGCTTACTTCACTTCAGCAACTATAATT
ATTGCGGTTCCAACTGGGATTAAAGTCTTTAGATGACTAGCCACCTTTCATGGAA
CTCAAATTTTAAATAGACCAGTAACCCTGTGAGCTTTAGGTTTTGTATTTTTATTT
ACAGTAGGAGGATTAACAGGGGTAATTTTAGCTAATTCTTCAATTGATATTATTCT

TCACGATACTTACTATGTAGTAGCTCATTTTCACTATGTTCTTTCTATAGGAGCTG
TATTTGCAATTATAGCCGGAATTGTCCAATGATTCCCATTATTTACCGGACTAAC
CTTAAATAATTTTTTTTTAAAAACCCAATTTATTACTATATTTTTAGGTGTAAATTTA
ACATTTTTTCCCCAACACTTTTTAGGGCTAAGAGGAATGCCTCGGCGATATTCGG
ATTATCCAGATGCTTTCACTATCTGAAATGCCATTTCATCTATTGGTTCCATTATT
TCTCTCACAAGAATTATCTTTTTTTTATTTATCTTATGAGAAAGGCTTTCTTCCCAA
CGTAAATCTTTGAGAACTTTAAATGTAACAACTTCAATTGAATGGTTACAAGCCCT
ACCCCCTAGTG
TAP2
ATTATTAGACAAGAAAGGGGGAAAAAAGAAGCTTTTGGAACCTTAGGAATAATTT
ATGCCATGATAGCTATCGGATTATTAGGATTCGTAGTTTGAGCTCACCATATATT
TACAGTAGGGATAGATGTAGATACCCGAGCTTACTTCACTTCAGCAACTATAATT
ATTGCGGTTCCAACTGGGATTAAAGTCTTTAGATGACTAGCCACCTTTCATGGAA
CTCAAATTTTAAATAGACCAGTAACCCTGTGAGCTTTAGGTTTTGTATTTTTATTT
ACAGTAGGAGGATTAACAGGGGTAATTTTAGCTAATTCTTCAATTGATATTATTCT
TCACGATACTTACTATGTAGTAGCTCATTTTCACTATGTTCTTTCTATAGGAGCTG
TATTTGCAATTATAGCCGGAATTGTCCAATGATTCCCATTATTTACCGGACTAAC
CTTAAATAATTTTTTTTTAAAAACCCAATTTATTACTATATTTTTAGGTGTAAATTTA
ACATTTTTTCCCCAACACTTTTTAGGGCTAAGAGGAATGCCTCGGCGATATTCGG
ATTATCCAGATGCTTTCACTATCTGAAATGCCATTTCATCTATTGGTTCCATTATT
TCTCTCACAAGAATTATCTTTTTTTTATTTATCTTATGAGAAAGGCTTTCTTCCCAA
CGTAAATCTTTGAGAACTTTAAATGTAACAACTTCAATTGAATGGTTACAAGCCCT
ACCCCCTAGTG
tol1
ATTATTAGACAAGAAAGGGGGAAAAAAGAAGCTTTTGGAGCCTTAGGAATAATTT
ATGCCATGATAGCTATCGGATTATTAGGATTCGTAGTTTGAGCTCACCATATATT
TACAGTAGGGATAGATGTAGATACCCGAGCTTACTTCACTTCAGCAACTATAATT
ATTGCGGTTCCAACTGGGATTAAAGTCTTTAGATGACTAGCCACCTTTCATGGAA
CTCAAATTTTAAATAGACCAGTAACCCTGTGAGCTTTAGGTTTTGTATTTTTATTT
ACAGTAGGAGGATTAACAGGGGTAATTTTAGCTAATTCTTCAATTGATATTATTCT
TCACGATACTTACTATGTAGTAGCTCATTTTCACTATGTTCTTTCTATAGGAGCTG
TATTTGCAATTATAGCCGGAATTGTCCAATGATTCCCATTATTTACCGGACTAAC
CTTAAATAATTTTTTTTTAAAAACCCAATTTATTACTATATTTTTAGGTGTAAATTTA
ACATTTTTTCCCCAACACTTTTTAGGGCTAAGAGGAATGCCTCGGCGATATTCGG
ATTATCCAGATGCTTTCACTATCTGAAATGCCATTTCATCTATTGGTTCCATTATT
TCTCTCACAAGAATTATCTTTTTTTTATTTATCTTATGAGAAAGGCTTTCTTCCCAA
CGTAAATCTTTGAGAACTTTAAATGTAACAACTTCAATTGAATGGTTACAAGCCCT
ACCCCCTAGTG
TOL1_1
ATTATTAGACAAGAAAGGGGGAAAAAAGAAGCTTTTGGAACCTTAGGAATAATTT
ATGCCATGATAGCTATCGGATTATTAGGATTCGTAGTTTGAGCTCACCATATATT
TACAGTAGGGATAGATGTAGATACCCGAGCTTACTTCACTTCAGCAACTATAATT
ATTGCGGTTCCAACTGGGATTAAAGTCTTTAGATGACTAGCCACCTTTCATGGAA
CTCAAATTTTAAATAGACCAGTAACCCTGTGAGCTTTAGGTTTTGTATTTTTATTT
ACAGTAGGAGGATTAACAGGGGTAATTTTAGCTAATTCTTCAATTGATATTATTCT
TCACGATACTTACTATGTAGTAGCTCATTTTCACTATGTTCTTTCTATAGGAGCTG
TATTTGCAATTATAGCCGGAATTGTCCAATGATTCCCATTATTTACCGGACTAAC
CTTAAATAATTTTTTTTTAAAAACCCAATTTATTACTATATTTTTAGGTGTAAATTTA
ACATTTTTTCCCCAACACTTTTTAGGGCTAAGAGGAATGCCTCGGCGATATTCGG
ATTATCCAGATGCTTTCACTATCTGAAATGCCATTTCATCTATTGGTTCCATTATT

TCTCTCACAAGAATTATCTTTTTTTTATTTATCTTATGAGAAAGGCTTTCTTCCCAA
CGTAAATCTTTGAGAACTTTAAATGTAACAACTTCAATTGAATGGTTACAAGCCCT
ACCCCCTAGTG
TOL5
ATTATTAGACAAGAAAGGGGGAAAAAAGAAGCTTTTGGAACCTTAGGAATAATTT
ATGCCATGATAGCTATCGGATTATTAGGATTCGTAGTTTGAGCTCACCATATATT
TACAGTAGGGATAGATGTAGATACCCGAGCTTACTTCACTTCAGCAACTATAATT
ATTGCGGTTCCAACTGGGATTAAAGTCTTTAGATGACTAGCCACCTTTCATGGAA
CTCAAATTTTAAATAGACCAGTAACCCTGTGAGCTTTAGGTTTTGTATTTTTATTT
ACAGTAGGAGGATTAACAGGGGTAATTTTAGCTAATTCTTCAATTGATATTATTCT
TCACGATACTTACTATGTAGTAGCTCATTTTCACTATGTTCTTTCTATAGGAGCTG
TATTTGCAATTATAGCCGGAATTGTCCAATGATTCCCATTATTTACCGGACTAAC
CTTAAATAATTTTTTTTTAAAAACCCAATTTATTACTATATTTTTAGGTGTAAATTTA
ACATTTTTTCCCCAACACTTTTTAGGGCTAAGAGGAATGCCTCGGCGATATTCGG
ATTATCCAGATGCTTTCACTATCTGAAATGCCATTTCATCTATTGGTTCCATTATT
TCTCTCACAAGAATTATCTTTTTTTTATTTATCTTATGAGAAAGGCTTTCTTCCCAA
CGTAAATCTTTGAGAACTTTAAATGTAACAACTTCAATTGAATGGTTACAAGCCCT
ACCCCCTAGTG
VIC10
ATTATTAGACAAGAAAGGGGGAAAAAAGAAGCTTTTGGAACCTTAGGAATAATTT
ATGCCATGATAGCTATCGGATTATTAGGATTCGTAGTTTGAGCTCACCATATATT
TACAGTAGGGATAGATGTAGATACCCGAGCTTACTTCACTTCAGCAACTATAATT
ATTGCGGTTCCAACTGGGATTAAAGTCTTTAGATGACTAGCCACCTTTCATGGAA
CTCAAATTTTAAATAGACCAGTAACCCTGTGAGCTTTAGGTTTTGTATTTTTATTT
ACAGTAGGAGGATTAACAGGGGTAATTTTAGCTAATTCTTCAATTGATATTATTCT
TCACGATACTTACTATGTAGTAGCTCATTTTCACTATGTTCTTTCTATAGGAGCTG
TATTTGCAATTATAGCCGGAATTGTCCAATGATTCCCATTATTTACCGGACTAAC
CTTAAATAATTTTTTTTTAAAAACCCAATTTATTACTATATTTTTAGGTGTAAATTTA
ACATTTTTTCCCCAACACTTTTTAGGGCTAAGAGGAATGCCTCGGCGATATTCGG
ATTATCCAGATGCTTTCACTATCTGAAATGCCATTTCATCTATTGGTTCCATTATT
TCTCTCACAAGAATTATCTTTTTTTTATTTATCTTATGAGAAAGGCTTTCTTCCCAA
CGTAAATCTTTGAGAACTTTAAATGTAACAACTTCAATTGAATGGTTACAAGCCCT
ACCCCCTAGTG
XAN4
ATTATTAGACAAGAAAGGGGGAAAAAAGAAGCTTTTGGAACCTTAGGAATAATTT
ATGCCATGATAGCTATCGGATTATTAGGATTCGTAGTTTGAGCTCACCATATATT
TACAGTAGGGATAGATGTAGATACCCGAGCTTACTTCACTTCAGCAACTATAATT
ATCGCGGTTCCAACTGGGATTAAAGTCTTTAGGTGACTAGCCACCTTTCATGGA
ACTCAAATTTTAAATAGACCAGTAACCCTGTGAGCTTTAGGTTTTGTATTTTTATT
TACAGTAGGAGGATTAACAGGGGTAATTTTAGCTAATTCTTCAATTGATATTATTC
TTCACGATACTTACTATGTAGTAGCTCATTTTCACTATGTTCTTTCTATAGGAGCT
GTATTTGCAATTATAGCCGGAATTGTCCAATGATTCCCATTATTTACTGGACTAA
CCTTAAATAATTTTTTTTTAAAAACCCAATTTATTACTATATTTTTAGGTGTAAATTT
AACATTTTTTCCTCAACACTTTTTAGGGCTAAGAGGAATGCCTCGGCGATATTCG
GATTATCCAGATGCTTTCGCTATCTGAAATGCCATTTCATCTATTGGTTCCATTAT
TTCTCTCACAAGAATTATCTTTTTTTTATTTATCTTATGAGAAAGGCTTTCTTCCCA
ACGTAAATCTTTGAGAACTTTAAATGTAACAACTTCAATTGAATGGTTACAAGCC
CTACCCCCTAGTG
XAX2
ATTATTAGACAAGAAAGGGGGAAAAAAGAAGCTTTTGGAACCTTAGGAATAATTT

ATGCCATGATAGCTATCGGATTATTAGGATTCGTAGTTTGAGCTCACCATATATT
TACAGTAGGGATAGATGTAGATACCCGAGCTTACTTCACTTCAGCAACTATAATT
ATTGCGGTTCCAACTGGGATTAAAGTCTTTAGATGACTAGCCACCTTTCATGGAA
CTCAAATTTTAAATAGACCAGTAACCCTGTGAGCTTTAGGTTTTGTATTTTTATTT
ACAGTAGGAGGATTAACAGGGGTAATTTTAGCTAATTCTTCAATTGATATTATTCT
TCACGATACTTACTATGTAGTAGCTCATTTTCACTATGTTCTTTCTATAGGAGCTG
TATTTGCAATTATAGCCGGAATTGTCCAATGATTCCCATTATTTACCGGACTAAC
CTTAAATAATTTTTTTTTAAAAACCCAATTTATTACTATATTTTTAGGTGTAAATTTA
ACATTTTTTCCCCAACACTTTTTAGGGCTAAGAGGAATGCCTCGGCGATATTCGG
ATTATCCAGATGCTTTCACTATCTGAAATGCCATTTCATCTATTGGTTCCATTATT
TCTCTCACAAGAATTATCTTTTTTTTTATTTATCTTATGAGAAAGGCTTTCTTCCCAA
CGTAAATCTTTGAGAACTTTAAATGTAACAACTTCAATTGAATGGTTACAAGCCCT
ACCCCCTAGTG
MEX_SFP
ATTATTAGACAAGAAAGGGGGAAAAAAGAAGCTTTTGGAACCTTAGGAATAATTT
ATGCCATAATAGCTATCGGATTATTAGGATTCGTAGTTTGAGCTCACCATATATTT
ACAGTAGGGATAGATGTAGATACCCGAGCTTACTTCACTTCAGCAACTATAATTA
TTGCGGTTCCAACTGGGATTAAAGTCTTTAGATGACTAGCCACCTTTCATGGAAC
TCAAATTTTAAATAGACCAGTAACCCTGTGAGCTTTAGGTTTTGTATTTTTATTTA
CAGTAGGAGGATTAACAGGGGTAATTTTAGCTAATTCTTCAATTGATATTATTCTT
CACGATACTTACTATGTAGTAGCTCATTTTCACTATGTTCTTTCTATAGGAGCTGT
ATTTGCAATTATAGCCGGAATTGTCCAATGATTCCCATTATTTACTGGACTAACCT
TAAATAATTTTTTTTTAAAAACCCAATTTATTACTATATTTTTAGGTGTAAATTTAAC
ATTTTTTCCTCAACACTTTTTAGGGCTAAGAGGAATGCCTCGGCGATATTCGGAT
TATCCAGATGCTTTCACTATCTGAAATGCCATTTCATCTATTGGTTCCATTATTTC
TCTCACAAGAATTATCTTTTTTTTTATTTATCTTATGAGAAAGGCTTTCTTCCCAAC
GTAAATCTTTGAGAACTTTAAATGTAACAACTTCAATTGAATGGTTACAAGCCCTA
CCCCCTAGTG
MEX_COE_2
ATTATTAGACAAGAAAGGGGGAAAAAAGAACCTTTTGGAACCTTAGGAATAATTT
ATGCCATAATAGCTATCGGATTATTAGGATTCGTAGTTTGAGCTCACCATATATTT
ACAGTAGGGATAGATGTAGATACCCGAGCTTACTTCACTTCAGCAACTATAATTA
TTGCGGTTCCAACTGGGATTAAAGTCTTTAGATGACTAGCCACCTTTCATGGAAC
TCAAATTTTAAATAGACCAGTAACCCTGTGAGCTTTAGGTTTTGTATTTTTATTTA
CAGTAGGAGGATTAACAGGGGTAATTTTAGCTAATTCTTCAATTGATATTATTCTT
CACGATACTTACTATGTAGTAGCTCATTTTCACTATGTTCTTTCTATAGGAGCTGT
ATTTGCAATTATAGCCGGAATTGTCCAATGATTCCCATTATTTACTGGACTAACCT
TAAATAATTTTTTTTTAAAAACCCAATTTATTACTATATTTTTAGGTGTAAATTTAAC
ATTTTTTCCTCAACACTTTTTAGGGCTAAGAGGAATGCCTCGGCGATATTCGGAT
TATCCAGATGCTTTCACTATCTGAAATGCCATTTCATCTATTGGTTCCATTATTTC
TCTCACAAGAATTATCTTTTTTTTTATTTATCTTATGAGAAAGGCTTTCTTCCCAAC
GTAAATCTTTGAGAACTTTAAATGTAACAACTTCAATTGAATGGTTACAAGCCCTA
CCCCCTAGTG
MEX_SAG_5
ATTATTAGACAAGAAAGGGGGAAAAAAGAAGCTTTTGGAACCTTAGGAATAATTT
ATGCCATAATAGCTATCGGATTATTAGGATTCGTAGTTTGAGCTCACCATATATTT
ACAGTAGGGATAGATGTAGATACCCGAGCTTACTTCACTTCAGCAACTATAATTA
TTGCGGTTCCAACTGGGATTAAAGTCTTTAGATGACTAGCCACCTTTCATGGAAC
TCAAATTTTAAATAGACCAGTAACCCTGTGAGCTTTAGGTTTTGTATTTTTATTTA
CAGTAGGAGGATTAACAGGGGTAATTTTAGCTAATTCTTCAATTGATATTATTCTT

CACGATACTTACTATGTAGTAGCTCATTTTCACTATGTTCTTTCTATAGGAGCTGT
ATTTGCAATTATAGCCGGAATTGTCCAATGATTCCCATTATTTACTGGGCTAACC
TTAAATAATTTTTTTTTAAAAACCCAATTTATTACTATATTTTTAGGTGTAAATTTAA
CATTTTTTCCTCAACACTTTTTAGGGCTAAGAGGAATGCCTCGGCGATATTCGGA
TTATCCAGATGCTTTCACTATCTGAAATGCCATTTCATCTATTGGTTCCATTATTT
CTCTCACAAGAATTATCTTTTTTTTTATTTATCTTATGAGAAAGGCTTTCTTCCCAA
CGTAAATCTTTGAGAACTTTAAATGTAACAACTTCAATTGAATGGTTACAAGCCCT
ACCCCCTAGTG
MEX_SCA
ATTATTAGACAAGAAAGGGGGAAAAAAGAAGCTTTTGGAACCTTAGGAATAATTT
ATGCCATAATAGCTATCGGATTATTAGGATTCGTAGTTTGAGCTCACCATATATTT
ACAGTAGGGATAGATGTAGATACCCGAGCTTACTTCACTTCAGCACCTATGATTA
TTGCGGTTCCAACTGGGATTAAAGTCTTTAGATGACTAGCCACCTTTCATGGAAC
TCAAATTTTAAATAGACCAGTAACCCTGTGAGCTTTAGGTTTTGTATTTTTATTTA
CAGTAGGAGGATTAACAGGGGTAATTTTAGCTAATTCTTCAATTGATATTATTCTT
CACGATACTTACTATGTAGTAGCTCATTTTCACTATGTTCTTTCTATAGGAGCTGT
ATTTGCAATTATAGCCGGAATTGTCCAATGATTCCCATTATTTACTGGACTAACCT
TAAATAATTTTTTTTTAAAAACCCAATTTATTACTATATTTTTAGGTGTAAATTTAAC
ATTTTTTCCTCAACACTTTTTAGGGCTAAGAGGAATGCCTCGGCGATATTCGGAT
TATCCAGATGCTTTCACTATCTGAAATGCCATTTCATCTATTGGTTCCATTATTTC
TCTCACAAGAATTATCTTTTTTTTTATTTATCTTATGAGAAAGGCTTTCTTCCCAAC
GTAAATCTTTGAGAACTTTAAATGTAACAACTTCAATTGAATGGTTACAAGCCCTA
CCCCCTAGTG
MEX_SJS_2
ATTATTAGACAAGAAAGGGGGAAAAAAGAACCTTTTGGAACCTTAGGAATAATTT
ATGCCATAATAGCTATCGGATTATTAGGATTCGTAGTTTGAGCTCACCATATATTT
ACAGTAGGGATAGATGTAGATACCCGAGCTTACTTCACTTCAGCAACTATAATTA
TTGCGGTTCCAACTGGAATTAAAGTCTTTAGATGACTAGCCACCTTTCATGGAAC
TCAAATTTTAAATAGACCAGTAACCCTGTGAGCTTTAGGTTTTGTATTTTTATTTA
CAGTAGGAGGATTAACAGGGGTAATTTTAGCTAATTCTTCAATTGATATTATTCTT
CACGATACTTACTATGTAGTAGCTCATTTTCACTATGTTCTTTCTATAGGAGCTGT
ATTTGCAATTATAGCCGGAATTGTCCAATGATTCCCATTATTTACTGGACTAACCT
TAAATAATTTTTTTTTAAAAACCCAATTTATTACTATATTTTTAGGTGTAAATTTAAC
ATTTTTTCCTCAACACTTTTTAGGGCTAAGAGGAATGCCTCGGCGATATTCGGAT
TATCCAGATGCTTTCACTATCTGAAATGCCATTTCATCTATTGGTTCCATTATTTC
TCTCACAAGAATTATCTTTTTTTTTATTTATCTTATGAGAAAGGCTTTCTTCCCAAC
GTAAATCTTTGAGAACTTTAAATGTAACAACTTCAATTGAATGGTTACAAGCCCTA
CCCCCTAGTG
MEX_STO
ATTATTAGACAAGAAAGGGGGAAAAAAGAACCTTTTGGAACCTTAGGAATAATTT
ATGCCATAATAGCTATCGGATTATTAGGATTCGTAGTTTGAGCTCACCATATATTT
ACAGTAGGGATAGATGTAGATACCCGAGCTTACTTCACTTCAGCAACTATAATTA
TTGCGGTTCCAACTGGGATTAAAGTCTTTAGATGACTAGCCACCTTTCATGGAAC
TCAAATTTTAAATAGACCAGTAACCCTGTGAGCTTTAGGTTTTGTATTTTTATTTA
CAGTAGGAGGATTAACAGGGGTAATTTTAGCTAATTCTTCAATTGATATTATTCTT
CACGATACTTACTATGTAGTAGCTCATTTTCACTATGTTCTTTCTATAGGAGCTGT
ATTTGCAATTATAGCCGGAATTGTCCAATGATTCCCATTATTTACTGGACTAACCT
TAAATAATTTTTTTTTAAAAACCCAATTTATTACTATATTTTTAGGTGTAAATTTAAC
ATTTTTTCCTCAACACTTTTTAGGGCTAAGAGGAATGCCTCGGCGATATTCGGAT
TATCCAGATGCTTTCACTATCTGAAATGCCATTTCATCTATTGGTTCCATTATTTC

TCTCACAAGAATTATCTTTTTTTTATTTATCTTATGAGAAAGGCTTTCTTCCCAAC
GTAAATCTTTGAGAACTTTAAATGTAACAACTTCAATTGAATGGTTACAAGCCCTA
CCCCCTAGTG
MEX_TLA_1
ATTATTAGACAAGAAAGGGGGAAAAAAGAAGCTTTTGGAACCTTAGGAATAATTT
ATGCCATAATAGCTATCGGATTATTAGGATTCGTAGTTTGAGCTCACCATATATTT
ACAGTAGGGATAGATGTAGATACCCGAGCTTACTTCACTTCAGCACCTATGATTA
TTGCGGTTCCAACTGGGATTAAAGTCTTTAGATGACTAGCCACCTTTCATGGAAC
TCAAATTTTAAATAGACCAGTAACCCTGTGAGCTTTAGGTTTTGTATTTTTATTTA
CAGTAGGAGGATTAACAGGGGTAATTTTAGCTAATTCTTCAATTGATATTATTCTT
CACGATACTTACTATGTAGTAGCTCATTTTCACTATGTTCTTTCTATAGGAGCTGT
ATTTGCAATTATAGCCGGAATTGTCCAATGATTCCCATTATTTACTGGACTAACCT
TAAATAATTTTTTTTTAAAAACCCAATTTATTACTATATTTTTAGGTGTAAATTTAAC
ATTTTTTCCTCAACACTTTTTAGGGCTAAGAGGAATGCCTCGGCGATATTCGGAT
TATCCAGATGCTTTCACTATCTGAAATGCCATTTCATCTATTGGTTCCATTATTTC
TCTCACAAGAATTATCTTTTTTTTATTTATCTTATGAGAAAGGCTTTCTTCCCAAC
GTAAATCTTTGAGAACTTTAAATGTAACAACTTCAATTGAATGGTTACAAGCCCTA
CCCCCTAGTG
MEX_XOT_3
ATTATTAGACAAGAAAGGGGGAAAAAAGAAGCTTTTGGAACCTTAGGAATAATTT
ATGCCATGATAGCTATCGGATTATTAGGATTCGTAGTTTGAGCTCACCATATATT
TACAGTAGGGATAGATGTAGATACCCGAGCTTACTTCACTTCAGCAACTATAATT
ATTGCGGTTCCAACTGGGATTAAAGTCTTTAGATGACTAGCCACCTTTCATGGAA
CTCAAATTTTAAATAGACCAGTAACCCTGTGAGCTTTAGGCTTTGTATTTTTATTT
ACAGTAGGAGGATTAACAGGGGTAATTTTAGCTAATTCTTCAATTGATATTATTCT
TCACGATACTTACTATGTAGTAGCTCATTTTCACTATGTTCTTTCTATAGGAGCTG
TATTTGCAATTATAGCCGGAATTGTCCAATGATTTCCATTATTTACTGGACTAACC
TTAAATAATTTTTTTTTAAAAACCCAATTTATTACTATATTTTTAGGTGTAAATTTAA
CATTTTTTCCTCAACACTTTTTAGGGCTAAGAGGAATGCCTCGGCGATATTCGGA
TTATCCAGATGCTTTCACTATCTGAAATGCCATTTCATCTATTGGTTCCATTATTT
CTCTCACAAGAATTATCTTTTTTTTATTTATCTTATGAGAAAGGCTTTCTTCCCAA
CGTAAATCTTTGAGAACTTTAAATGTAACAACTTCAATTGAATGGTTACAAGCCCT
ACCCCCTAGTG
MEX_MAL_1
ATTATTAGACAAGAAAGGGGGAAAAAAGAAGCTTTTGGAACCTTAGGAATAATTT
ATGCCATAATAGCTATCGGATTATTAGGATTCGTAGTTTGAGCTCACCATATATTT
ACAGTAGGGATAGATGTAGATACCCGAGCTTACTTCACTTCAGCAACTATAATTA
TTGCGGTTCCAACTGGGATTAAAGTCTTTAGATGACTAGCCACCTTTCATGGAAC
TCAAATTTTAAATAGACCAGTAACCCTGTGAGCTTTAGGTTTTGTATTTTTATTTA
CAGTAGGAGGATTAACAGGGGTAATTTTAGCTAATTCTTCAATTGATATTATTCTT
CACGATACTTACTATGTAGTAGCTCATTTTCACTATGTTCTTTCTATAGGAGCTGT
ATTTGCAATTATAGCCGGAATTGTCCAATGATTCCCATTATTTACTGGACTAACCT
TAAATAATTTTTTTTTAAAAACCCAATTTATTACTATATTTTTAGGTGTAAATTTAAC
ATTTTTTCCTCAACACTTTTTAGGGCTAAGAGGAATGCCTCGGCGATATTCGGAT
TATCCAGATGCTTTCACTATCTGAAATGCCATTTCATCTATTGGTTCCATTATTTC
TCTCACAAGAATTATCTTTTTTTTATTTATCTTATGAGAAAGGCTTTCTTCCCAAC
GTAAATCTTTGAGAACTTTAAATGTAACAACTTCAATTGAATGGTTACAAGCCCTA
CCCCCTAGTG
MEX_MAL_2
ATTATTAGACAAGAAAGGGGGAAAAAAGAAGCTTTTGGAACCTTAGGAATAATTT

ATGCCATAATAGCTATGGGATTATTAGGATTCGTCGTTTGAGCTCACCATATATT
TACAGTAGGGATAGATGTAGATACCCGAGCTTACTTCACTTCAGCAACTATAATT
ATTGCGGTTCCAACTGGGATTAAAGTCTTTAGATGACTAGCCACCTTTCATGGAA
CTCAAATTTTAAATAGACCAGTAACCCTGTGAGCTTTAGGTTTTGTATTTTTATTT
ACAGTAGGAGGATTAACAGGGGTAATTTTAGCTAATTCTTCAATTGATATTATTCT
TCACGATACTTACTATGTAGTAGCTCATTTTCACTATGTTCTTTCTATAGGAGCTG
TATTTGCAATTATAGCCGGAATTGTCCAATGATTCCCATTATTTACTGGACTAACC
TTAAATAATTTTTTTTTAAAAACCCAATTTATTACTATATTTTTAGGTGTAAATTTAA
CATTTTTTCCTCAACACTTTTTAGGGCTAAGAGGAATGCCTCGGCGATATTCGGA
TTATCCAGATGCTTTCACTATCTGAAATGCCATTTCATCCATTGGTTCCATTATTT
CTCTCACAAGAATTATCTTTTTTTTTATTTATCCTATGAGAAAGGCTTTCTTCCCAA
CGTAAATCTTTGAGAACTTTAAATGTAACAACTTCAATTGAATGGTTACAAGCCCT
ACCCCCTAGTG
MEX_OCU_1
ATTATTAGACAAGAAAGGGGGAAAAAAGAAGCTTTTGGAACCTTAGGAATAATTT
ATGCCATAATAGCTATCGGATTATTAGGATTCGTAGTTTGAGCTCACCATATATTT
ACAGTAGGGATAGATGTAGATACCCGAGCTTACTTCACTTCAGCAACTATAATTA
TTGCGGTTCCAACTGGGATTAAAGTCTTTAGATGACTAGCCACCTTTCATGGAAC
TCAAATTTTAAATAGACCAGTAACCCTGTGAGCTTTAGGTTTTGTATTTTTATTTA
CAGTAGGAGGATTAACAGGGGTAATTTTAGCTAATTCTTCAATTGATATTATTCTT
CACGATACTTACTATGTAGTAGCTCATTTTCACTATGTTCTTTCTATAGGAGCTGT
ATTTGCAATTATAGCCGGAATTGTCCAATGATTCCCATTATTTACTGGACTAACCT
TAAATAATTTTTTTTTAAAAACCCAATTTATTACTATATTTTTAGGTGTAAATTTAAC
ATTTTTTCCTCAACACTTTTTAGGGCTAAGAGGAATGCCTCGGCGATATTCGGAT
TATCCAGATGCTTTCACTATCTGAAATGCCATTTCATCTATTGGTTCCATTATTTC
TCTCACAAGAATTATCTTTTTTTTTATTTATCTTATGAGAAAGGCTTTCTTCCCAAC
GTAAATCTTTGAGAACTTTAAATGTAACAACTTCAATTGAATGGTTACAAGCCCTA
CCCCCTAGTG
MEX_OCU_2
ATTATTAGACAAGAAAGGGGGAAAAAAGAAGCTTTTGGAACCTTAGGAATAATTT
ATGCCATAATAGCTATCGGATTATTAGGATTCGTAGTTTGAGCTCACCATATATTT
ACAGTAGGGATAGATGTAGATACCCGAGCTTACTTCACTTCAGCAACTATAATTA
TTGCGGTTCCAACTGGGATTAAAGTCTTTAGATGACTAGCCACCTTTCATGGAAC
TCAAATTTTAAATAGACCAGTAACCCTGTGAGCTTTAGGTTTTGTATTTTTATTTA
CAGTAGGAGGATTAACAGGGGTAATTTTAGCTAATTCTTCAATTGATATTATTCTT
CACGATACTTACTATGTAGTAGCTCATTTTCACTATGTTCTTTCTATAGGAGCTGT
ATTTGCAATTATAGCCGGAATTGTCCAATGATTCCCATTATTTACTGGACTAACCT
TAAATAATTTTTTTTTAAAAACCCAATTTATTACTATATTTTTAGGTGTAAATTTAAC
ATTTTTTCCTCAACACTTTTTAGGGCTAAGAGGAATGCCTCGGCGATATTCGGAT
TATCCAGATGCTTTCACTATCTGAAATGCCATTTCATCTATTGGTTCCATTATTTC
TCTCACAAGAATTATCTTTTTTTTTATTTATCTTATGAGAAAGGCTTTCTTCCCAAC
GTAAATCTTTGAGAACTTTAAATGTAACAACTTCAATTGAATGGTTACAAGCCCTA
CCCCCTAGTG
MEX_SAG_1
ATTATTAGACAAGAAAGGGGGAAAAAAGAAGCTTTTGGAACCTTAGGAATAATTT
ATGCCATAATAGCTATCGGATTATTAGGATTCGTAGTTTGAGCTCACCATATATTT
ACAGTAGGGATAGATGTAGATACCCGAGCTTACTTCACTTCAGCAACTATAATTA
TTGCGGTTCCAACTGGGATTAAAGTCTTTAGATGACTAGCCACCTTTCATGGAAC
TCAAATTTTAAATAGACCAGTAACCCTGTGAGCTTTAGGTTTTGTATTTTTATTTA
CAGTAGGAGGATTAACAGGGGTAATTTTAGCTAATTCTTCAATTGATATTATTCTT

CACGATACTTACTATGTAGTAGCTCATTTTCACTATGTTCTTTCTATAGGAGCTGT
ATTTGCAATTATAGCCGGAATTGTCCAATGATTCCCATTATTTACTGGGCTAACC
TTAAATAATTTTTTTTTAAAAACCCAATTTATTACTATATTTTTAGGTGTAAATTTAA
CATTTTTTCCTCAACACTTTTTAGGGCTAAGAGGAATGCCTCGGCGATATTCGGA
TTATCCAGATGCTTTCACTATCTGAAATGCCATTTCATCTATTGGTTCCATTATTT
CTCTCACAAGAATTATCTTTTTTTTATTTATCTTATGAGAAAGGCTTTCTTCCCAA
CGTAAATCTTTGAGAACTTTAAATGTAACAACTTCAATTGAATGGTTACAAGCCCT
ACCCCCTAGTG
MEX_SAG_2
ATTATTAGACAAGAAAGGGGGAAAAAAGAAGCTTTTGGAACCTTAGGAATAATTT
ATGCCATAATAGCTATCGGATTATTAGGATTCGTAGTTTGAGCTCACCATATATTT
ACAGTAGGGATAGATGTAGATACCCGAGCTTACTTCACTTCAGCAACTATAATTA
TTGCGGTTCCAACTGGGATTAAAGTCTTTAGATGACTAGCCACCTTTCATGGAAC
TCAAATTTTAAATAGACCAGTAACCCTGTGAGCTTTAGGTTTTGTATTTTTATTTA
CAGTAGGAGGATTAACAGGGGTAATTTTAGCTAATTCTTCAATTGATATTATTCTT
CACGATACTTACTATGTAGTAGCTCATTTTCACTATGTTCTTTCTATAGGAGCTGT
ATTTGCAATTATAGCCGGAATTGTCCAATGATTCCCATTATTTACTGGGCTAACC
TTAAATAATTTTTTTTTAAAAACCCAATTTATTACTATATTTTTAGGTGTAAATTTAA
CATTTTTTCCTCAACACTTTTTAGGGCTAAGAGGAATGCCTCGGCGATATTCGGA
TTATCCAGATGCTTTCACTATCTGAAATGCCATTTCATCTATTGGTTCCATTATTT
CTCTCACAAGAATTATCTTTTTTTTATTTATCTTATGAGAAAGGCTTTCTTCCCAA
CGTAAATCTTTGAGAACTTTAAATGTAACAACTTCAATTGAATGGTTACAAGCCCT
ACCCCCTAGTG
MEX_SJC_1
ATTATTAGACAAGAAAGGGGGAAAAAAGAAGCTTTTGGAACCTTAGGAATAATTT
ATGCCATAATAGCTATCGGATTATTAGGATTCGTAGTTTGAGCTCACCATATATTT
ACAGTAGGGATAGATGTAGATACCCGAGCTTACTTCACTTCAGCAACTATAATTA
TTGCGGTTCCAACTGGGATTAAAGTCTTTAGATGACTAGCCACCTTTCATGGAAC
TCAAATTTTAAATAGACCAGTAACCCTGTGAGCTTTAGGTTTTGTATTTTTATTTA
CAGTAGGAGGATTAACAGGGGTAATTTTAGCTAATTCTTCAATTGATATTATTCTT
CACGATACTTACTATGTAGTAGCTCATTTTCACTATGTTCTTTCTATAGGAGCTGT
ATTTGCAATTATAGCCGGAATTGTCCAATGATTCCCATTATTTACTGGACTAACCT
TAAATAATTTTTTTTTAAAAACCCAATTTATTACTATATTTTTAGGTGTAAATTTAAC
ATTTTTTCCTCAACACTTTTTAGGGCTAAGAGGAATGCCTCGGCGATATTCGGAT
TATCCAGATGCTTTCACTATCTGAAATGCCATTTCATCTATTGGTTCCATTATTTC
TCTCACAAGAATTATCTTTTTTTTATTTATCTTATGAGAAAGGCTTTCTTCCCAAC
GTAAATCTTTGAGAACTTTAAATGTAACAACTTCAATTGAATGATTACAAGCCCTA
CCCCCTAGTG
MEX_SJC_2
ATTATTAGACAAGAAAGGGGGAAAAAAGAAGCTTTTGGAACCTTAGGAATAATTT
ATGCCATAATAGCTATCGGATTATTAGGATTCGTAGTTTGAGCTCACCATATATTT
ACAGTAGGGATAGATGTAGATACCCGAGCTTACTTCACTTCAGCAACTATAATTA
TTGCGGTTCCAACTGGGATTAAAGTCTTTAGATGACTAGCCACCTTTCATGGAAC
TCAAATTTTAAATAGACCAGTAACCCTGTGAGCTTTAGGTTTTGTATTTTTATTTA
CAGTAGGAGGATTAACAGGGGTAATTTTAGCTAATTCTTCAATTGATATTATTCTT
CACGATACTTACTATGTAGTAGCTCATTTTCACTATGTTCTTTCTATAGGAGCTGT
ATTTGCAATTATAGCCGGAATTGTCCAATGATTCCCATTATTTACTGGACTAACCT
TAAATAATTTTTTTTTAAAAACCCAATTTATTACTATATTTTTAGGTGTAAATTTAAC
ATTTTTTCCTCAACACTTTTTAGGGCTAAGAGGAATGCCTCGGCGATATTCGGAT
TATCCAGATGCTTTCACTATCTGAAATGCCATTTCATCTATTGGTTCCATTATTTC

TCTCACAAGAATTATCTTTTTTTTATTTATCTTATGAGAAAGGCTTTCTTCCCAAC
GTAAATCTTTGAGAACTTTAAATGTAACAACTTCAATTGAATGGTTACAAGCCCTA
CCCCCTAGTG
MEX_SJC_3
ATTATTAGACAAGAAAGGGGGAAAAAAGAAGCTTTTGGAACCTTAGGAATAATTT
ATGCCATAATAGCTATCGGATTATTAGGATTCGTAGTTTGAGCTCACCATATATTT
ACAGTAGGGATAGATGTAGATACCCGAGCTTACTTCACTTCAGCAACTATAATTA
TTGCGGTTCCAACTGGGATTAAAGTCTTTAGATGACTAGCCACCTTTCATGGAAC
TCAAATTTTAAATAGACCAGTAACCCTGTGAGCTTTAGGTTTTGTATTTTTATTTA
CAGTAGGAGGATTAACAGGGGTAATTTTAGCTAATTCTTCAATTGATATTATTCTT
CACGATACTTACTATGTAGTAGCTCATTTTCACTATGTTCTTTCTATAGGAGCTGT
ATTTGCAATTATAGCCGGAATTGTCCAATGATTCCCATTATTTACTGGACTAACCT
TAAATAATTTTTTTTTAAAAACCCAATTTATTACTATATTTTTAGGTGTAAATTTAAC
ATTTTTTCCTCAACACTTTTTAGGGCTAAGAGGAATGCCTCGGCGATATTCGGAT
TATCCAGATGCTTTCACTATCTGAAATGCCATTTCATCTATTGGTTCCATTATTTC
TCTCACAAGAATTATCTTTTTTTTATTTATCTTATGAGAAAGGCTTTCTTCCCAAC
GTAAATCTTTGAGAACTTTAAATGTAACAACTTCAATTGAATGGTTACAAGCCCTA
CCCCCTAGTG
MEX_SJS
ATTATTAGACAAGAAAGGGGGAAAAAAGAACCTTTTGGAACCTTAGGAATAATTT
ATGCCATAATAGCTATCGGATTATTAGGATTCGTAGTTTGAGCTCACCATATATTT
ACAGTAGGGATAGATGTAGATACCCGAGCTTACTTCACTTCAGCAACTATAATTA
TTGCGGTTCCAACTGGAATTAAAGTCTTTAGATGACTAGCCACCTTTCATGGAAC
TCAAATTTTAAATAGACCAGTAACCCTGTGAGCTTTAGGTTTTGTATTTTTATTTA
CAGTAGGAGGATTAACAGGGGTAATTTTAGCTAATTCTTCAATTGATATTATTCTT
CACGATACTTACTATGTAGTAGCTCATTTTCACTATGTTCTTTCTATAGGAGCTGT
ATTTGCAATTATAGCCGGAATTGTCCAATGATTCCCATTATTTACTGGACTAACCT
TAAATAATTTTTTTTTAAAAACCCAATTTATTACTATATTTTTAGGTGTAAATTTAAC
ATTTTTTCCTCAACACTTTTTAGGGCTAAGAGGAATGCCTCGGCGATATTCGGAT
TATCCAGATGCTTTCACTATCTGAAATGCCATTTCATCTATTGGTTCCATTATTTC
TCTCACAAGAATTATCTTTTTTTTATTTATCTTATGAGAAAGGCTTTCTTCCCAAC
GTAAATCTTTGAGAACTTTAAATGTAACAACTTCAATTGAATGGTTACAAGCCCTA
CCCCCTAGTG
MEX_SJS_1
ATTATTAGACAAGAAAGGGGGAAAAAAGAAGCTTTTGGAACCTTAGGAATAATTT
ATGCCATAATACCTATCGGATTATTAGGATTCGTCGTTTGAGCTCACCATATATTT
ACAGTACGGATAGATGTAGATACCCGAGCTTACTTCACTTCAGCAACTATAATTA
TTGCGGTTCCAACTGGGATTAAAGTCTTTAGATGACTAGCCACCTTTCATGGAAC
TCAAATTTTAAATAGACCAGTAACCCTGTGAGCTTTAGGTTTTGTATTTTTATTTA
CAGTAGGAGGATTAACAGGGGTAATTTTAGCTAATTCTTCAATTGATATTATTCTT
CACGATACTTACTATGTAGTAGCTCATTTTCACTATGTTCTTTCTATAGGAGCTGT
ATTTGCAATTATAGCCGGAATTATCCAATGATTCCCATTATTTACTGGACTAACCT
TAAATAATTTTTTTTTAAAAACCCAATTTATTACTATATTTTTAGGTGTAAATTTAAC
ATTTTTTCCTCAACACTTTTTAGGGCTAAGAGGAATGCCTCGGCGATATTCGGAT
TATCCAGATGCTTTCACTATCTGAAATGCCATTTCATCCATTGGTTCCATTATTTC
TCTCACAAGAATTATCTTTTTTTTATTTATCTTATGAGAAAGGCTTTCTTCCCAAC
GTAAATCTTTGAGAACTTCAAATGTAACAACTTCAATTGAATGGTGACAAGCCCT
ACCCCCTAGTG
MEX_SJS_3
ATTATTAGACAAGAAAGGGGGAAAAAAGAAGCTTTTGGAACCTTAGGAATAATTT

ATGCCATAATACCTATCGGATTATTAGGATTCGTCGTTTGAGCTCACCATATATTT
ACAGTACGGATAGATGTAGATACCCGAGCTTACTTCACTTCAGCAACTATAATTA
TTGCGGTTCCAACTGGGATTAAAGTCTTTAGATGACTAGCCACCTTTCATGGAAC
TCAAATTTTAAATAGACCAGTAACCCTGTGAGCTTTAGGTTTTGTATTTTTATTTA
CAGTAGGAGGATTAACAGGGGTAATTTTAGCTAATTCTTCAATTGATATTATTCTT
CACGATACTTACTATGTAGTAGCTCATTTTCACTATGTTCTTTCTATAGGAGCTGT
ATTTGCAATTATAGCCGGAATTATCCAATGATTCCCATTATTTACTGGACTAACCT
TAAATAATTTTTTTTTAAAAACCCAATTTATTACTATATTTTTAGGTGTAAATTTAAC
ATTTTTTCCTCAACACTTTTTAGGGCTAAGAGGAATGCCTCGGCGATATTCGGAT
TATCCAGATGCTTTCACTATCTGAAATGCCATTTCATCCATTGGTTCCATTATTTC
TCTCACAAGAATTATCTTTTTTTTTATTTATCTTATGAGAAAGGCTTTCTTCCCAAC
GTAAATCTTTGAGAACTTCAAATGTAACAACTTCAATTGAATGGTGACAAGCCCT
ACCCCCTAGTG
MEX_SPT_1
ATTATTAGACAAGAAAGGGGGAAAAAAGAAGCTTTTGGAACCTTAGGAATAATTT
ATGCCATAATAGCTATCGGATTATTAGGATTCGTAGTTTGAGCTCACCATATATTT
ACAGTAGGGATAGATGTAGATACCCGAGCTTACTTCACTTCAGCAACTATAATTA
TTGCGGTTCCAACTGGGATTAAAGTCTTTAGATGACTAGCCACCTTTCATGGAAC
TCAAATTTTAAATAGACCAGTAACCCTGTGAGCTCTAGGTTTTGTATTTTTATTTA
CAGTAGGAGGATTAACAGGGGTAATTTTAGCTAATTCTTCAATTGATATTATTCTT
CACGATACTTACTATGTAGTAGCTCATTTTCACTATGTTCTTTCTATAGGAGCTGT
ATTTGCAATTATAGCCGGAATTGTCCAATGATTCCCATTATTTACTGGACTAACCT
TAAATAATTTTTTTTTAAAAACCCAATTTATTACTATATTTTTAGGTGTAAATTTAAC
ATTTTTTCCTCAACACTTTTTAGGGCTAAGAGGAATGCCTCGGCGATATTCGGAT
TATCCAGATGCTTTCACTATCTGAAATGCCATTTCATCTATTGGTTCCATTATTTC
TCTCACAAGAATTATCTTTTTTTTTATTTATCTTATGAGAAAGGCTTTCTTCCCAAC
GTAAATCTTTGAGAACTTTAAATGTAACAACTTCAATTGAATGGTTACAAGCCCTA
CCCCCTAGTG
MEX_SPT_2
ATTATTAGACAAGAAAGGGGGAAAAAAGAAGCTTTTGGAACCTTAGGAATAATTT
ATGCCATAATAGCTATCGGATTATTAGGATTCGTAGTTTGAGCTCACCATATATTT
ACAGTAGGGATAGATGTAGATACCCGAGCTTACTTCACTTCAGCAACTATAATTA
TTGCGGTTCCAACTGGGATTAAAGTCTTTAGATGACTAGCCACCTTTCATGGAAC
TCTAATTTTAAATAGACCAGTAACCCTATGAGCTTTAGGTTTTGTATTTTTATTTAC
AGTAGGAGGTTTAACAGGAGTAATTTTAGCTAATTCTTCAATTGATATTATTCTTC
ACGATACTTACTATGTAGTAGCTCATTTTCACTATGTTCTTTCTATAGGAGCTGTA
TTTGCAATTATAGCCGGAATTGTCCAATGATTCCCATTATTTACTGGACTAACCTT
AAATAATTTTTTTTTAAAAACCCAATTTATTACTATATTTTTAGGTGTAAATTTAACA
TTTTTTCCTCAACACTTTTTAGGGCTAAGAGGAATGCCTCGGCGATATTCGGATT
ATCCAGATGCTTTCACTATCTGAAATGCCATTTCATCTATTGGTTCTATTATTTCT
CTCACAAGAATTATCTTTTTTTTTATTTATCTTATGAGAAAGGCTTTCTTCCCAACG
TAAATCTTTGAGAACTTTAAATGTAACAACTTCAATTGAATGGTTACAAGCCCTAC
CCCCTAGTG
MEX_SPT_3
ATTATTAGACAAGAAAGGGGGAAAAAAGAAGCTTTTGGAACCTTAGGAATAATTT
ATGCCATAATAGCTATCGGATTATTAGGATTCGTAGTTTGAGCTCACCATATATTT
ACAGTAGGGATAGATGTAGATACCCGAGCTTACTTCACTTCAGCAACTATAATTA
TTGCGGTTCCAACTGGGATTAAAGTCTTTAGATGACTAGCCACCTTTCATGGAAC
TCTAATTTTAAATAGACCAGTAACCCTATGAGCTTTAGGTTTTGTATTTTTATTTAC
AGTAGGAGGTTTAACAGGAGTAATTTTAGCTAATTCTTCAATTGATATTATTCTTC

ACGATACTTACTATGTAGTAGCTCATTTTCACTATGTTCTTTCTATAGGAGCTGTA
TTTGCAATTATAGCCGGAATTGTCCAATGATTCCCATTATTTACTGGACTAACCTT
AAATAATTTTTTTTTAAAAACCCAATTTATTACTATATTTTTAGGTGTAAATTTAACA
TTTTTTCCTCAACACTTTTTAGGGCTAAGAGGAATGCCTCGGCGATATTCGGATT
ATCCAGATGCTTTCACTATCTGAAATGCCATTTCATCTATTGGTTCTATTATTTCT
CTCACAAGAATTATCTTTTTTTTATTTATCTTATGAGAAAGGCTTTCTTCCCAACG
TAAATCTTTGAGAACTTTAAATGTAACAACTTCAATTGAATGGTTACAAGCCCTAC
CCCCTAGTG
MEX_TEP
ATTATTAGACAAGAAAGGGGGAAAAAAGAAGCTTTTGGAACCTTAGGAATAATTT
ATGCCATAATAGCTATCGGATTATTAGGATTCGTAGTTTGAGCTCACCATATATTT
ACAGTAGGGATAGATGTAGATACCCGAGCTTACTTCACTTCAGCAACTATAATTA
TTGCGGTTCCAACTGGGATTAAAGTCTTTAGATGACTAGCCACCTTTCATGGAAC
TCAAATTTTAAATAGACCAGTAACCCTGTGAGCTTTAGGTTTTGTATTTTTATTTA
CAGTAGGAGGATTAACAGGGGTAATTTTAGCTAATTCTTCAATTGATATTATTCTT
CACGATACTTACTATGTAGTAGCTCATTTTCACTATGTTCTTTCTATAGGAGCTGT
ATTTGCAATTATAGCCGGAATTGTCCAATGATTCCCATTATTTACTGGACTAACCT
TAAATAATTTTTTTTTAAAAACCCAATTTATTACTATATTTTTAGGTGTAAATTTAAC
ATTTTTTCCTCAACACTTTTTAGGGCTAAGAGGAATGCCTCGGCGATATTCGGAT
TATCCAGATGCTTTCACTATCTGAAATGCCATTTCATCTATTGGTTCCATTATTTC
TCTCACAAGAATTATCTTTTTTTTATTTATCTTATGAGAAAGGCTTTCTTCCCAAC
GTAAATCTTTGAGAACTTTAAATGTAACAACTTCAATTGAATGGTTACAAGCCCTA
CCCCCTAGTG
MEX_TEP_1
ATTATTAGACAAGAAAGGGGGAAAAAAGAAGCTTTTGGAACCTTAGGAATAATTT
ATGCCATAATAGCTATCGGATTATTAGGATTCGTAGTTTGAGCTCACCATATATTT
ACAGTAGGGATAGATGTAGATACCCGAGCTTACTTCACTTCAGCAACTATAATTA
TTGCGGTTCCAACTGGGATTAAAGTCTTTAGATGACTAGCCACCTTTCATGGAAC
TCAAATTTTAAATAGACCAGTAACCCTGTGAGCTTTAGGTTTTGTATTTTTATTTA
CAGTAGGAGGATTAACAGGGGTAATTTTAGCTAATTCTTCAATTGATATTATTCTT
CACGATACTTACTATGTAGTAGCTCATTTTCACTATGTTCTTTCTATAGGAGCTGT
ATTTGCAATTATAGCCGGAATTGTCCAATGATTCCCATTATTTACTGGACTAACCT
TAAATAATTTTTTTTTAAAAACCCAATTTATTACTATATTTTTAGGTGTAAATTTAAC
ATTTTTTCCTCAACACTTTTTAGGGCTAAGAGGAATGCCTCGGCGATATTCGGAT
TATCCAGATGCTTTCACTATCTGAAATGCCATTTCATCTATTGGTTCCATTATTTC
TCTCACAAGAATTATCTTTTTTTTATTTATCTTATGAGAAAGGCTTTCTTCCCAAC
GTAAATCTTTGAGAACTTTAAATGTAACAACTTCAATTGAATGGTTACAAGCCCTA
CCCCCTAGTG
MEX_TEP_2
ATTATTAGACAAGAAAGGGGGAAAAAAGAAGCTTTTGGAACCTTAGGAATAATTT
ATGCCATAATAGCTATCGGATTATTAGGATTCGTAGTTTGAGCTCACCATATATTT
ACAGTAGGGATAGATGTAGATACCCGAGCTTACTTCACTTCAGCAACTATAATTA
TTGCGGTTCCAACTGGGATTAAAGTCTTTAGATGACTAGCCACCTTTCATGGAAC
TCAAATTTTAAATAGACCAGTAACCCTGTGAGCTTTAGGTTTTGTATTTTTATTTA
CAGTAGGAGGATTAACAGGGGTAATTTTAGCTAATTCTTCAATTGATATTATTCTT
CACGATACTTACTATGTAGTAGCTCATTTTCACTATGTTCTTTCTATAGGAGCTGT
ATTTGCAATTATAGCCGGAATTGTCCAATGATTTCCATTATTTACTGGACTAACCT
TAAATAATTTTTTTTTAAAAACCCAATTTATTACTATATTTTTAGGTGTAAATTTAAC
ATTTTTTCCTCAACACTTTTTAGGGCTAAGAGGAATGCCTCGGCGATATTCGGAT
TATCCAGATGCTTTCACTATCTGAAATGCCATTTCATCTATTGGTTCCATTATTTC

TCTCACAAGAATTATCTTTTTTTTATTTATCTTATGAGAAAGGCTTTCTTCCCAAC
GTAAATCTTTGAGAACTTTAAATGTAACAACTTCAATTGAATGGTTACAAGCCCTA
CCCCCTAGTG
MEX_TLA
ATTATTAGACAAGAAAGGGGGAAAAAAGAAGCTTTTGGAACCTTAGGAATAATTT
ATGCCATAATAGCTATCGGATTATTAGGATTCGTAGTTTGAGCTCACCATATATTT
ACAGTAGGGATAGATGTAGATACCCGAGCTTACTTCACTTCAGCACCTATGATTA
TTGCGGTTCCAACTGGGATTAAAGTCTTTAGATGACTAGCCACCTTTCATGGAAC
TCAAATTTTAAATAGACCAGTAACCCTGTGAGCTTTAGGTTTTGTATTTTTATTTA
CAGTAGGAGGATTAACAGGGGTAATTTTAGCTAATTCTTCAATTGATATTATTCTT
CACGATACTTACTATGTAGTAGCTCATTTTCACTATGTTCTTTCTATAGGAGCTGT
ATTTGCAATTATAGCCGGAATTGTCCAATGATTCCCATTATTTACTGGACTAACCT
TAAATAATTTTTTTTTAAAAACCCAATTTATTACTATATTTTTAGGTGTAAATTTAAC
ATTTTTTCCTCAACACTTTTTAGGGCTAAGAGGAATGCCTCGGCGATATTCGGAT
TATCCAGATGCTTTCACTATCTGAAATGCCATTTCATCTATTGGTTCCATTATTTC
TCTCACAAGAATTATCTTTTTTTTATTTATCTTATGAGAAAGGCTTTCTTCCCAAC
GTAAATCTTTGAGAACTTTAAATGTAACAACTTCAATTGAATGGTTACAAGCCCTA
CCCCCTAGTG
MEX_TLA_2
ATTATTAGACAAGAAAGGGGGAAAAAAGAACCTTTTGGACCCTTAGGAATAATTT
ATCCCTTATTACCTATCGGATTATTAGGATTCGTCGTTTGAGCTCACCATATCTTT
ACAGTAGGTATAGCTGTAGATACCCGAGCTTACTTCACTTCAGCAACTATAATTA
TTGCGGTTCCAACTGGGATTAAAGTCTTTAGATGACTAGCCACCTTTCATGGAAC
TCAAATTTTAAATAGACCAGTAACCCTGTGAGCTTTAGGTTTTGTATTTTTATTTA
CAGTAGGAGGATTAACAGGGGTAATTTTAGCTAATTCTTCAATTGATATTATTCTT
CACGATACTTACTATGTAGTAGCTCATTTTCACTATGTTCTTTCTATAGGAGCTGT
ATTTGCAATTATAGCCGGAATTAGCCAATGATTCCCATTATTTACTGGACTAACC
TTAAATAATTTTTTTTTAAAAACCCAATTTATTACTATATTTTTAGGTGTAAATTTAA
CATTTTTTCCTCAACACTTTTTAGGGCTAAGAGGAATGCCTCGGCGATATTCGGA
TTATCCAGATGCTTTCACTACCTGAAATGCCATTTCATCCATTGGTTCCATTATTT
CTCTCACAAGAATTATCTTTTTTTTATTTATCTTATGAGAAAGGCTTTCTTCCCAA
CGTAAATCTTTGAGAACTTCAAATGTAACAACTTCAATTGAATGGTGACAAGCCC
TACCCCCTAGTG
MEX_TLA_3
ATTATTAGACAAGAAAGGGGGAAAAAAGAAGCTTTTGGAACCTTAGGAATAATTT
ATGCCATAATAGCTATCGGATTATTAGGATTCGTAGTTTGAGCTCACCATATATTT
ACAGTAGGGATAGATGTAGATACCCGAGCTTACTTCACTTCAGCAACTATAATTA
TTGCGGTTCCAACTGGGATTAAAGTCTTTAGATGACTAGCCACCTTTCATGGAAC
TCAAATTTTAAATAGACCAGTAACCCTGTGAGCTTTAGGTTTTGTATTTTTATTTA
CAGTAGGAGGATTAACAGGGGTAATTTTAGCTAATTCTTCAATTGATATTATTCTT
CACGATACTTACTATGTAGTAGCTCATTTTCACTATGTTCTTTCTATAGGAGCTGT
ATTTGCAATTATAGCCGGAATTGTCCAATGATTCCCATTATTTACTGGACTAACCT
TAAATAATTTTTTTTTAAAAACCCAATTTATTACTATATTTTTAGGTGTAAATTTAAC
ATTTTTTCCTCAACACTTTTTAGGGCTAAGAGGAATGCCTCGGCGATATTCGGAT
TATCCAGATGCTTTCACTATCTGAAATGCCATTTCATCTATTGGTTCCATTATTTC
TCTCACAAGAATTATCTTTTTTTTATTTATCTTATGAGAAAGGCTTTCTTCCCAAC
GTAAATCTTTGAGAACTTTAAATGTAACAACTTCAATTGAATGGTTGCAAGCCCT
ACCCCCTAGTG
MEX_YAU_1
ATTATTAGACAAGAAAGGGGGAAAAAAGAAGCTTTTGGAACCTTAGGAATAATTT

ATGCCATGATAGCTATCGGATTATTAGGATTCGTAGTTTGAGCTCACCATATATT
TACAGTAGGGATAGATGTAGATACCCGAGCTTACTTCACTTCAGCAACTATAATT
ATTGCGGTTCCAACTGGGATTAAAGTCTTTAGATGGCTAGCCACCTTTCATGGAA
CTCAAATTTTAAATAGACCAGTAACCCTGTGAGCTTTAGGTTTTGTATTTTTATTT
ACAGTAGGAGGATTAACAGGGGTAATTTTAGCTAATTCTTCAATTGATATTATTCT
TCACGATACTTACTATGTAGTAGCTCATTTTCACTATGTTCTTTCTATAGGAGCTG
TATTTGCAATTATAGCCGGAATTAGCCAATGATTCCCATTATTTACTGGACTAAC
CTTAAATAATTTTTTTTTAAAAACCCAATTTATTACTATATTTTTAGGTGTAAATTTA
ACATTTTTTCCTCAACACTTTTTAGGGCTAAGAGGAATGCCTCGGCGATATTCGG
ATTATCCAGATGCTTTCACTATCTGAAATGCCATTTCATCTATTGGTTCCATTATT
TCTCTCACAAGAATTATCTTTTTTTTTATTTATCTTATGAGAAAGGCTTTCTTCCCAA
CGTAAATCTTTGAGAACTTTAAATGTAACAACTTCAATTGAATGGTTACAAGCCCT
ACCCCCTAGTG
MEX_YAU_2
ATTATTAGACAAGAAAGGGGGAAAAAAGAAGCTTTTGGAACCTTAGGAATAATTT
ATGCCATAATAGCTATCGGATTATTAGGATTCGTAGTTTGAGCTCACCATATATTT
ACAGTAGGGATAGATGTAGATACCCGAGCTTACTTCACTTCAGCAACTATAATTA
TTGCGGTTCCAACTGGGATTAAAGTCTTTAGATGACTAGCCACCTTTCATGGAAC
TCAAATTTTAAATAGACCAGTAACCCTGTGAGCTTTAGGTTTTGTATTTTTATTTA
CAGTAGGAGGATTAACAGGGGTAATTTTAGCTAATTCTTCAATTGATATTATTCTT
CACGATACTTACTATGTAGTAGCTCATTTTCACTATGTTCTTTCTATAGGAGCTGT
ATTTGCAATTATAGCCGGAATTGTCCAATGATTCCCATTATTTACTGGACTAACCT
TAAATAATTTTTTTTTAAAAACCCAATTTATTACTATATTTTTAGGTGTAAATTTAAC
ATTTTTTCCTCAACACTTTTTAGGGCTAAGAGGAATGCCTCGGCGATATTCGGAT
TATCCAGATGCTTTCACTATCTGAAATGCCATTTCATCTATTGGCTCCATTATTTC
TCTCACAAGAATTATCTTTTTTTTTATTTATCTTATGAGAAAGGCTTTCTTCCCAAC
GTAAATCTTTGAGAACTTTAAATGTAACAACTTCAATTGAATGGTTACAAGCCCTA
CCCCCTAGTG
CAM_YAH
ATTATTAGACAAGAAAGGGGGAAAAAAGAAGCTTTTGGAACCTTAGGAATAATTT
ATGCCATGATAGCTATCGGATTATTAGGATTCGTAGTTTGAGCTCTCCATATATT
TACAGTAGGGATAGATGTAGATACCCGAGCTTACTTCACTTCAGCAACTATGATT
ATCGCGGTTCCAACTGGGATTAAAGTCTTTAGGTGACTAGCCACCTTTCATGGA
ACTCAAATTTTAAATAGACCAGTAACCCTGTGAGCTTTAGGTTTTGTATTTTTATT
TACAGTAGGAGGATTAACAGGGGTAATTTTAGCTAATTCTTCAATTGATATTATTC
TTCACGATACTTACTATGTAGTAGCTCATTTTCACTATGTTCTTTCTATAGGAGCT
GTATTTGCAATTATAGCCGGAATTGTCCAATGATTCCCATTATTTACTGGACTAA
CCTTAAATAATTTTTTTTTAAAAACCCAATTTATTACTATATTTTTAGGTGTAAATTT
AACATTTTTTCCTCAACACTTTTTAGGGCTAAGAGGAATGCCTCGGCGATATTCG
GATTATCCAGATGCTTTCACTATCTGAAATGCCATTTCATCTATTGGTTCCATTAT
TTCTCTCACAAGAATTATCTTTTTTTTTATTTATCTTATGAGAAAGGCTTTCTTCCCA
ACGTAAATCTTTGAGAACTTTAAATGTAACAACTTCAATTGAATGGTTACAAGCC
CTACCCCCTAGTG
CAM_YAH01
ATTATTAGACAAGAAAGGGGGAAAAAAGAAGCTTTTGGAACCTTAGGAATAATTT
ATGCCATGATAGCTATCGGATTATTAGGATTCGTAGTTTGAGCTCTCCATATATT
TACAGTAGGGATAGATGTAGATACCCGAGCTTACTTCACTTCAGCAACTATGATT
ATCGCGGTTCCAACTGGGATTAAAGTCTTTAGGTGACTAGCCACCTTTCATGGA
ACTCAAATTTTAAATAGACCAGTAACCCTGTGAGCTTTAGGTTTTGTATTTTTATT
TACAGTAGGAGGATTAACAGGGGTAATTTTAGCTAATTCTTCAATTGATATTATTC

TTCACGATACTTACTATGTAGTAGCTCATTTTCACTATGTTCTTTCTATAGGAGCT
GTATTTGCAATTATAGCCGGAATTGTCCAATGATTCCCATTATTTACTGGACTAA
CCTTAAATAATTTTTTTTTAAAAACCCAATTTATTACTATATTTTTAGGTGTAAATTT
AACATTTTTTCCTCAACACTTTTTAGGGCTAAGAGGAATGCCTCGGCGATATTCG
GATTATCCAGATGCTTTCACTATCTGAAATGCCATTTCATCTATTGGTTCCATTAT
TTCTCTCACAAGAATTATCTTTTTTTTATTTATCTTATGAGAAAGGCTTTCTTCCCA
ACGTAAATCTTTGAGAACTTTAAATGTAACAACTTCAATTGAATGGTTACAAGCC
CTACCCCCTAGTG
CAM_YAH02
ATTATTAGACAAGAAAGGGGGAAAAAAGAAGCTTTTGGAACCTTAGGAATAATTT
ATGCCATGATAGCTATCGGATTATTAGGATTCGTAGTTTGAGCTCTCCATATATT
TACAGTAGGGATAGATGTAGATACCCGAGCTTACTTCACTTCAGCAACTATGATT
ATCGCGGTTCCAACTGGGATTAAAGTCTTTAGGTGACTAGCCACCTTTCATGGA
ACTCAAATTTTAAATAGACCAGTAACCCTGTGAGCTTTAGGTTTTGTATTTTTATT
TACAGTAGGAGGATTAACAGGGGTAATTTTAGCTAATTCTTCAATTGATATTATTC
TTCACGATACTTACTATGTAGTAGCTCATTTTCACTATGTTCTTTCTATAGGAGCT
GTATTTGCAATTATAGCCGGAATTGTCCAATGATTCCCATTATTTACTGGACTAA
CCTTAAATAATTTTTTTTTAAAAACCCAATTTATTACTATATTTTTAGGTGTAAATTT
AACATTTTTTCCTCAACACTTTTTAGGGCTAAGAGGAATGCCTCGGCGATATTCG
GATTATCCAGATGCTTTCACTATCTGAAATGCCATTTCATCTATTGGTTCCATTAT
TTCTCTCACAAGAATTATCTTTTTTTTATTTATCTTATGAGAAAGGCTTTCTTCCCA
ACGTAAATCTTTGAGAACTTTAAATGTAACAACTTCAATTGAATGGTTACAAGCC
CTACCCCCTAGTG
China
ATTATTAGACAAGAAAGGGGGAAAAAAGAAGCTTTTGGAACCTTAGGAATAATTT
ATGCCATGATAGCTATCGGATTATTAGGATTCGTAGTTTGAGCTCACCATATATT
TACAGTAGGGATAGATGTAGATACCCGAGCTTACTTCACTTCAGCAACTATAATT
ATTGCGGTTCCAACTGGGATTAAAGTCTTTAGATGACTAGCCACCTTTCATGGAA
CTCAAATTTTAAATAGACCAGTAACCCTGTGAGCTTTAGGTTTTGTATTTTTATTT
ACAGTAGGAGGATTAACAGGGGTAATTTTAGCTAATTCTTCAATTGATATTATTCT
TCACGATACTTACTATGTAGTAGCTCATTTTCACTATGTTCTTTCTATAGGAGCTG
TATTTGCAATTATAGCCGGAATTGTCCAATGATTCCCATTATTTACCGGACTAAC
CTTAAATAATTTTTTTTTAAAAACCCAATTTATTACTATATTTTTAGGTGTAAATTTA
ACATTTTTTCCCCAACACTTTTTAGGGCTAAGAGGAATGCCTCGGCGATATTCGG
ATTATCCAGATGCTTTCACTATCTGAAATGCCATTTCATCTATTGGTTCCATTATT
TCTCTCACAAGAATTATCTTTTTTTTATTTATCTTATGAGAAAGGCTTTCTTCCCAA
CGTAAATCTTTGAGAACTTTAAATGTAACAACTTCAATTGAATGGTTACAAGCCCT
ACCCCCTAGTG
FRA_CHA_5
ATTATTAGACAAGAAAGGGGGAAAAAAGAAGCTTTTGGAACCTTAGGAATAATTT
ATGCCATGATAGCTATCGGATTATTAGGATTCGTAGTTTGAGCTCACCATATATT
TACAGTAGGGATAGATGTAGATACCCGAGCTTACTTCACTTCAGCAACTATAATT
ATTGCGGTTCCAACTGGGATTAAAGTCTTTAGATGACTAGCCACCTTTCATGGAA
CTCAAATTTTAAATAGACCAGTAACCCTGTGAGCTTTAGGCTTTGTATTTTTATTT
ACAGTAGGAGGATTAACAGGGGTAATTTTAGCTAATTCTTCAATTGATATTATTCT
TCACGATACTTACTATGTAGTAGCTCATTTTCACTATGTTCTTTCTATAGGAGCTG
TATTTGCAATTATAGCCGGAATTGTCCAATGATTTCCATTATTTACTGGACTAACC
TTAAATAATTTTTTTTTAAAAACCCAATTTATTACTATATTTTTAGGTGTAAATTTAA
CATTTTTTCCTCAACACTTTTTAGGGCTAAGAGGAATGCCTCGGCGATATTCGGA
TTATCCAGATGCTTTCACTATCTGAAATGCCATTTCATCTATTGGTTCCATTATTT

CTCTCACAAGAATTATCTTTTTTTTATTTATCTTATGAGAAAGGCTTTCTTCCCAA
CGTAAATCTTTGAGAACTTTAAATGTAACAACTTCAATTGAATGGTTACAAGCCCT
ACCCCCTAGTG
SPA_PRA_1
ATTATTAGACAAGAAAGGGGGAAAAAAGAAGCTTTTGGAACCTTAGGAATAATTT
ATGCCATGATAGCTATCGGATTATTAGGATTCGTAGTTTGAGCTCACCATATATT
TACAGTAGGGATAGATGTAGATACCCGAGCTTACTTCACTTCAGCAACTATAATT
ATTGCGGTTCCAACTGGGATTAAAGTCTTTAGATGACTAGCCACCTTTCATGGAA
CTCAAATTTTAAATAGACCAGTAACCCTGTGAGCTTTAGGTTTTGTATTTTTATTT
ACAGTAGGAGGATTAACAGGGGTAATTTTAGCTAATTCTTCAATTGATATTATTCT
TCACGATACTTACTATGTAGTAGCTCATTTTCACTATGTTCTTTCTATAGGAGCTG
TATTTGCAATTATAGCCGGAATTGTCCAATGATTCCCATTATTTACCGGACTAAC
CTTAAATAATTTTTTTTTAAAAACCCAATTTATTACTATATTTTTAGGTGTAAATTTA
ACATTTTTTCCCCAACACTTTTTAGGGCTAAGAGGAATGCCTCGGCGATATTCGG
ATTATCCAGATGCTTTCACTATCTGAAATGCCATTTCATCTATTGGTTCCATTATT
TCTCTCACAAGAATTATCTTTTTTTTATTTATCTTATGAGAAAGGCTTTCTTCCCAA
CGTAAATCTTTGAGAACTTTAAATGTAACAACTTCAATTGAATGGTTACAAGCCCT
ACCCCCTAGTG
SWI_NEU_1
ATTATTAGACAAGAAAGGGGGAAAAAAGAAGCTTTTGGAACCTTAGGAATAATTT
ATGCCATGATAGCTATCGGATTATTAGGATTCGTAGTTTGAGCTCACCATATATT
TACAGTAGGGATAGATGTAGATACCCGAGCTTACTTCACTTCAGCAACTATGATT
ATCGCGGTTCCAACTGGGATTAAAGTCTTTAGGTGGCTAGCCACCTTTCATGGA
ACTCAAATTTTAAATAGACCAGTAACCCTGTGAGCTTTAGGTTTTGTATTTTTATT
TACAGTAGGAGGATTAACAGGGGTAATTTTAGCTAATTCTTCAATTGATATTATTC
TTCACGATACTTACTATGTAGTAGCTCATTTTCACTATGTTCTTTCTATAGGAGCT
GTATTTGCAATTATAGCCGGAATTGTCCAATGATTCCCATTATTTACTGGACTAA
CCTTAAATAATTTTTTTTTAAAAACCCAATTTATTACTATATTTTTAGGTGTAAATTT
AACATTTTTTCCTCAACACTTTTTAGGGCTAAGAGGAATGCCTCGGCGATATTCG
GATTATCCAGATGCTTTCACTATCTGAAATGCCATTTCATCTATTGGTTCCATTAT
TTCTCTCACAAGAATTATCTTTTTTTTATTTATCTTATGAGAAAGGCTTTCTTCCCA
ACGTAAATCTTTGAGAACTTTAAATGTAACAACTTCAATTGAATGGTTACAAGCC
CTACCCCCTAGTG
SWT_NEU
ATTATTAGACAAGAAAGGGGGAAAAAAGAAGCTTTTGGAACCTTAGGAATAATTT
ATGCCATGATAGCTATCGGATTATTAGGATTCGTAGTTTGAGCTCACCATATATT
TACAGTAGGGATAGATGTAGATACCCGAGCTTACTTCACTTCAGCAACTATGATT
ATCGCGGTTCCAACTGGGATTAAAGTCTTTAGGTGGCTAGCCACCTTTCATGGA
ACTCAAATTTTAAATAGACCAGTAACCCTGTGAGCTTTAGGTTTTGTATTTTTATT
TACAGTAGGAGGATTAACAGGGGTAATTTTAGCTAATTCTTCAATTGATATTATTC
TTCACGATACTTACTATGTAGTAGCTCATTTTCACTATGTTCTTTCTATAGGAGCT
GTATTTGCAATTATAGCCGGAATTGTCCAATGATTCCCATTATTTACTGGACTAA
CCTTAAATAATTTTTTTTTAAAAACCCAATTTATTACTATATTTTTAGGTGTAAATTT
AACATTTTTTCCTCAACACTTTTTAGGGCTAAGAGGAATGCCTCGGCGATATTCG
GATTATCCAGATGCTTTCACTATCTGAAATGCCATTTCATCTATTGGTTCCATTAT
TTCTCTCACAAGAATTATCTTTTTTTTATTTATCTTATGAGAAAGGCTTTCTTCCCA
ACGTAAATCTTTGAGAACTTTAAATGTAACAACTTCAATTGAATGGTTACAAGCC
CTACCCCCTAGTG
SWT_NEU2
ATTATTAGACAAGAAAGGGGGAAAAAAGAAGCTTTTGGAACCTTAGGAATAATTT

ATGCCATGATAGCTATCGGATTATTAGGATTCGTAGTTTGAGCTCACCATATATT
TACAGTAGGGATAGATGTAGATACCCGAGCTTACTTCACTTCAGCAACTATGATT
ATCGCGGTTCCAACTGGGATTAAAGTCTTTAGGTGGCTAGCCACCTTTCATGGA
ACTCAAATTTTAAATAGACCAGTAACCCTGTGAGCTTTAGGTTTTGTATTTTTATT
TACAGTAGGAGGATTAACAGGGGTAATTTTAGCTAATTCTTCAATTGATATTATTC
TTCACGATACTTACTATGTAGTAGCTCATTTTCACTATGTTCTTTCTATAGGAGCT
GTATTTGCAATTATAGCCGGAATTGTCCAATGATTCCCATTATTTACTGGACTAA
CCTTAAATAATTTTTTTTTAAAAACCCAATTTATTACTATATTTTTAGGTGTAAATTT
AACATTTTTTCCTCAACACTTTTTAGGGCTAAGAGGAATGCCTCGGCGATATTCG
GATTATCCAGATGCTTTCACTATCTGAAATGCCATTTCATCTATTGGTTCCATTAT
TTCTCTCACAAGAATTATCTTTTTTTTATTTATCTTATGAGAAAGGCTTTCTTCCCA
ACGTAAATCTTTGAGAACTTTAAATGTAACAACTTCAATTGAATGGTTACAAGCC
CTACCCCCTAGTG
MEX_YOH
ATTATTAGACAAGAAAGGGGGAAAAAAGAAGCTTTTGGAACCTTAGGAATAATTT
ATGCTATAATAGCTATCGGATTATTAGGATTCGTAGTTTGAGCTCACCATATATTT
ACAGTAGGAATAGATGTAGATACCCGAGCTTACTTCACTTCAGCAACTATAATTA
TTGCGGTTCCAACTGGGATTAAAGTCTTTAGATGACTAGCCACCTTTCATGGAAC
TCAAATTTTAAATAGACCAGTAACCCTGTGAGCTTTAGGTTTTGTATTTTTATTTA
CAGTAGGAGGATTAACAGGGGTAATTTTAGCTAATTCTTCAATTGATATTATTCTC
CACGATACTTACTATGTAGTAGCTCATTTTCACTATGTTCTTTCTATAGGAGCTGT
ATTTGCAATTATAGCCGGAATTGTCCAATGATTCCCATTATTTACCGGACTAACC
TTAAATAATTTTTTTTTAAAAACCCAATTTATTACTATATTTTTAGGTGTAAATTTAA
CATTTTTTCCTCAACACTTTTTAGGGCTAAGAGGAATGCCTCGGCGATATTCGGA
TTATCCAGATGCTTTCACTATCTGAAATGCCATTTCATCTATTGGTTCCATTATTT
CTCTCACAAGAATTATCTTTTTTTTATTTATCTTATGAGAAAGGCTTTCTTCCCAA
CGTAAATCTTTGAGAACTTTAAATGTAACAACTTCAATTGAATGGTTTCAAGCCCT
ACCCCCTAGTG
MEX_YOH_1
ATTATTAGACAAGAAAGGGGGAAAAAAGAAGCTTTTGGAACCTTAGGAATAATTT
ATGCTATAATAGCTATCGGATTATTAGGATTCGTAGTTTGAGCTCACCATATATTT
ACAGTAGGAATAGATGTAGATACCCGAGCTTACTTCACTTCAGCAACTATAATTA
TTGCGGTTCCAACTGGGATTAAAGTCTTTAGATGACTAGCCACCTTTCATGGAAC
TCAAATTTTAAATAGACCAGTAACCCTGTGAGCTTTAGGTTTTGTATTTTTATTTA
CAGTAGGAGGATTAACAGGGGTAATTTTAGCTAATTCTTCAATTGATATTATTCTC
CACGATACTTACTATGTAGTAGCTCATTTTCACTATGTTCTTTCTATAGGAGCTGT
ATTTGCAATTATAGCCGGAATTGTCCAATGATTCCCATTATTTACCGGACTAACC
TTAAATAATTTTTTTTTAAAAACCCAATTTATTACTATATTTTTAGGTGTAAATTTAA
CATTTTTTCCTCAACACTTTTTAGGGCTAAGAGGAATGCCTCGGCGATATTCGGA
TTATCCAGATGCTTTCACTATCTGAAATGCCATTTCATCTATTGGTTCCATTATTT
CTCTCACAAGAATTATCTTTTTTTTATTTATCTTATGAGAAAGGCTTTCTTCCCAA
CGTAAATCTTTGAGAACTTTAAATGTAACAACTTCAATTGAATGGTTTCAAGCCCT
ACCCCCTAGTG
MEX_YOH_2
ATTATTAGACAAGAAAGGGGGAAAAAAGAAGCTTTTGGAACCTTAGGAATAATTT
ATGCTATAATAGCTATCGGATTATTAGGATTCGTAGTTTGAGCTCACCATATATTT
ACAGTAGGAATAGATGTAGATACCCGAGCTTACTTCACTTCAGCAACTATAATTA
TTGCGGTTCCAACTGGGATTAAAGTCTTTAGATGACTAGCCACCTTTCATGGAAC
TCAAATTTTAAATAGACCAGTAACCCTGTGAGCTTTAGGTTTTGTATTTTTATTTA
CAGTAGGAGGATTAACAGGGGTAATTTTAGCTAATTCTTCAATTGATATTATTCTC

CACGATACTTACTATGTAGTAGCTCATTTTCACTATGTTCTTTCTATAGGAGCTGT
ATTTGCAATTATAGCCGGAATTGTCCAATGATTCCCATTATTTACCGGACTAACC
TTAAATAATTTTTTTTTAAAAACCCAATTTATTACTATATTTTTAGGTGTAAATTTAA
CATTTTTTCCTCAACACTTTTTAGGGCTAAGAGGAATGCCTCGGCGATATTCGGA
TTATCCAGATGCTTTCACTATCTGAAATGCCATTTCATCTATTGGTTCCATTATTT
CTCTCACAAGAATTATCTTTTTTTTATTTATCTTATGAGAAAGGCTTTCTTCCCAA
CGTAAATCTTTGAGAACTTTAAATGTAACAACTTCAATTGAATGGTTTCAAGCCCT
ACCCCCTAGTG
AOPE_15
ATTATTAGACAAGAAAGGGGGAAAAAAGAAGCTTTTGGAACCTTAGGAATAATTT
ATGCTATAATAGCTATCGGATTATTAGGATTCGTAGTTTGAGCTCACCATATATTT
ACAGTAGGAATAGATGTAGATACCCGAGCTTACTTCACTTCAGCAACTATAATTA
TTGCGGTTCCAACTGGGATTAAAGTCTTTAGATGACTAGCCACCTTTCATGGAAC
TCAAATTTTAAATAGACCAGTAACCCTGTGAGCTTTAGGTTTTGTATTTTTATTTA
CAGTAGGAGGATTAACAGGGGTAATTTTAGCTAATTCTTCAATTGATATTATTCTC
CACGATACTTACTATGTAGTAGCTCATTTTCACTATGTTCTTTCTATAGGAGCTGT
ATTTGCAATTATAGCCGGAATTGTCCAATGATTCCCATTATTTACCGGACTAACC
TTAAATAATTTTTTTTTAAAAACCCAATTTATTACTATATTTTTAGGTGTAAATTTAA
CATTTTTTCCTCAACACTTTTTAGGGCTAAGAGGAATGCCTCGGCGATATTCGGA
TTATCCAGATGCTTTCACTATCTGAAATGCCATTTCATCTATTGGTTCCATTATTT
CTCTCACAAGAATTATCTTTTTTTTATTTATCTTATGAGAAAGGCTTTCTTCCCAA
CGTAAATCTTTGAGAACTTTAAATGTAACAACTTCAATTGAATGGTTTCAAGCCCT
ACCCCCTAGTG
AOPE_2
ATTATTAGACAAGAAAGGGGGAAAAAAGAAGCTTTTGGAACCTTAGGAATAATTT
ATGCTATAATAGCTATCGGATTATTAGGATTCGTAGTTTGAGCTCACCATATATTT
ACAGTAGGAATAGATGTAGATACCCGAGCTTACTTCACTTCAGCAACTATAATTA
TTGCGGTTCCAACTGGGATTAAAGTCTTTAGATGACTAGCCACCTTTCATGGAAC
TCAAATTTTAAATAGACCAGTAACCCTGTGAGCTTTAGGTTTTGTATTTTTATTTA
CAGTAGGAGGATTAACAGGGGTAATTTTAGCTAATTCTTCAATTGATATTATTCTC
CACGATACTTACTATGTAGTAGCTCATTTTCACTATGTTCTTTCTATAGGAGCTGT
ATTTGCAATTATAGCCGGAATTGTCCAATGATTCCCATTATTTACCGGACTAACC
TTAAATAATTTTTTTTTAAAAACCCAATTTATTACTATATTTTTAGGTGTAAATTTAA
CATTTTTTCCTCAACACTTTTTAGGGCTAAGAGGAATGCCTCGGCGATATTCGGA
TTATCCAGATGCTTTCACTATCTGAAATGCCATTTCATCTATTGGTTCCATTATTT
CTCTCACAAGAATTATCTTTTTTTTATTTATCTTATGAGAAAGGCTTTCTTCCCAA
CGTAAATCTTTGAGAACTTTAAATGTAACAACTTCAATTGAATGGTTTCAAGCCCT
ACCCCCTAGTG
AOPE_3
ATTATTAGACAAGAAAGGGGGAAAAAAGAAGCTTTTGGAACCTTAGGAATAATTT
ATGCTATAATAGCTATCGGATTATTAGGATTCGTAGTTTGAGCTCACCATATATTT
ACAGTAGGAATAGATGTAGATACCCGAGCTTACTTCACTTCAGCAACTATAATTA
TTGCGGTTCCAACTGGGATTAAAGTCTTTAGATGACTAGCCACCTTTCATGGAAC
TCAAATTTTAAATAGACCAGTAACCCTGTGAGCTTTAGGTTTTGTATTTTTATTTA
CAGTAGGAGGATTAACAGGGGTAATTTTAGCTAATTCTTCAATTGATATTATTCTC
CACGATACTTACTATGTAGTAGCTCATTTTCACTATGTTCTTTCTATAGGAGCTGT
ATTTGCAATTATAGCCGGAATTGTCCAATGATTCCCATTATTTACCGGACTAACC
TTAAATAATTTTTTTTTAAAAACCCAATTTATTACTATATTTTTAGGTGTAAATTTAA
CATTTTTTCCTCAACACTTTTTAGGGCTAAGAGGAATGCCTCGGCGATATTCGGA
TTATCCAGATGCTTTCACTATCTGAAATGCCATTTCATCTATTGGTTCCATTATTT

CTCTCACAAGAATTATCTTTTTTTTATTTATCTTATGAGAAAGGCTTTCTTCCCAA
CGTAAATCTTTGAGAACTTTAAATGTAACAACTTCAATTGAATGGTTTCAAGCCCT
ACCCCCTAGTG
AOPE_12
ATTATTAGACAAGAAAGGGGGAAAAAAGAAGCTTTTGGAACCTTAGGAATAATTT
ATGCTATAATAGCTATCGGATTATTAGGATTCGTAGTTTGAGCTCACCATATATTT
ACAGTAGGAATAGATGTAGATACCCGAGCTTACTTCACTTCAGCAACTATAATTA
TTGCGGTTCCAACTGGGATTAAAGTCTTTAGATGACTAGCCACCTTTCATGGAAC
TCAAATTTTAAATAGACCAGTAACCCTGTGAGCTTTAGGTTTTGTATTTTTATTTA
CAGTAGGAGGATTAACAGGGGTAATTTTAGCTAATTCTTCAATTGATATTATTCTC
CACGATACTTACTATGTAGTAGCTCATTTTCACTATGTTCTTTCTATAGGAGCTGT
ATTTGCAATTATAGCCGGAATTGTCCAATGATTCCCATTATTTACCGGACTAACC
TTAAATAATTTTTTTTTAAAAACCCAATTTATTACTATATTTTTAGGTGTAAATTTAA
CATTTTTTCCTCAACACTTTTTAGGGCTAAGAGGAATGCCTCGGCGATATTCGGA
TTATCCAGATGCTTTCACTATCTGAAATGCCATTTCATCTATTGGTTCCATTATTT
CTCTCACAAGAATTATCTTTTTTTTATTTATCTTATGAGAAAGGCTTTCTTCCCAA
CGTAAATCTTTGAGAACTTTAAATGTAACAACTTCAATTGAATGGTTTCAAGCCCT
ACCCCCTAGTG
AOPE_13
ATTATTAGACAAGAAAGGGGGAAAAAAGAAGCTTTTGGAACCTTAGGAATAATTT
ATGCTATAATAGCTATCGGATTATTAGGATTCGTAGTTTGAGCTCACCATATATTT
ACAGTAGGAATAGATGTAGATACCCGAGCTTACTTCACTTCAGCAACTATAATTA
TTGCGGTTCCAACTGGGATTAAAGTCTTTAGATGACTAGCCACCTTTCATGGAAC
TCAAATTTTAAATAGACCAGTAACCCTGTGAGCTTTAGGTTTTGTATTTTTATTTA
CAGTAGGAGGATTAACAGGGGTAATTTTAGCTAATTCTTCAATTGATATTATTCTC
CACGATACTTACTATGTAGTAGCTCATTTTCACTATGTTCTTTCTATAGGAGCTGT
ATTTGCAATTATAGCCGGAATTGTCCAATGATTCCCATTATTTACCGGACTAACC
TTAAATAATTTTTTTTTAAAAACCCAATTTATTACTATATTTTTAGGTGTAAATTTAA
CATTTTTTCCTCAACACTTTTTAGGGCTAAGAGGAATGCCTCGGCGATATTCGGA
TTATCCAGATGCTTTCACTATCTGAAATGCCATTTCATCTATTGGTTCCATTATTT
CTCTCACAAGAATTATCTTTTTTTTATTTATCTTATGAGAAAGGCTTTCTTCCCAA
CGTAAATCTTTGAGAACTTTAAATGTAACAACTTCAATTGAATGGTTTCAAGCCCT
ACCCCCTAGTG
AOPE_14
ATTATTAGACAAGAAAGGGGGAAAAAAGAAGCTTTTGGAACCTTAGGAATAATTT
ATGCTATAATAGCTATCGGATTATTAGGATTCGTAGTTTGAGCTCACCATATATTT
ACAGTAGGAATAGATGTAGATACCCGAGCTTACTTCACTTCAGCAACTATAATTA
TTGCGGTTCCAACTGGGATTAAAGTCTTTAGATGACTAGCCACCTTTCATGGAAC
TCAAATTTTAAATAGACCAGTAACCCTGTGAGCTTTAGGTTTTGTATTTTTATTTA
CAGTAGGAGGATTAACAGGGGTAATTTTAGCTAATTCTTCAATTGATATTATTCTC
CACGATACTTACTATGTAGTAGCTCATTTTCACTATGTTCTTTCTATAGGAGCTGT
ATTTGCAATTATAGCCGGAATTGTCCAATGATTCCCATTATTTACCGGACTAACC
TTAAATAATTTTTTTTTAAAAACCCAATTTATTACTATATTTTTAGGTGTAAATTTAA
CATTTTTTCCTCAACACTTTTTAGGGCTAAGAGGAATGCCTCGGCGATATTCGGA
TTATCCAGATGCTTTCACTATCTGAAATGCCATTTCATCTATTGGTTCCATTATTT
CTCTCACAAGAATTATCTTTTTTTTATTTATCTTATGAGAAAGGCTTTCTTCCCAA
CGTAAATCTTTGAGAACTTTAAATGTAACAACTTCAATTGAATGGTTTCAAGCCCT
ACCCCCTAGTG
AOPE_4
ATTATTAGACAAGAAAGGGGGAAAAAAGAAGCTTTTGGAACCTTAGGAATAATTT

ATGCTATAATAGCTATCGGATTATTAGGATTCGTAGTTTGAGCTCACCATATATTT
ACAGTAGGAATAGATGTAGATACCCGAGCTTACTTCACTTCAGCAACTATAATTA
TTGCGGTTCCAACTGGGATTAAAGTCTTTAGATGACTAGCCACCTTTCATGGAAC
TCAAATTTTAAATAGACCAGTAACCCTGTGAGCTTTAGGTTTTGTATTTTTATTTA
CAGTAGGAGGATTAACAGGGGTAATTTTAGCTAATTCTTCAATTGATATTATTCTC
CACGATACTTACTATGTAGTAGCTCATTTTCACTATGTTCTTTCTATAGGAGCTGT
ATTTGCAATTATAGCCGGAATTGTCCAATGATTCCCATTATTTACCGGACTAACC
TTAAATAATTTTTTTTTAAAAACCCAATTTATTACTATATTTTTAGGTGTAAATTTAA
CATTTTTTCCTCAACACTTTTTAGGGCTAAGAGGAATGCCTCGGCGATATTCGGA
TTATCCAGATGCTTTCACTATCTGAAATGCCATTTCATCTATTGGTTCCATTATTT
CTCTCACAAGAATTATCTTTTTTTTATTTATCTTATGAGAAAGGCTTTCTTCCCAA
CGTAAATCTTTGAGAACTTTAAATGTAACAACTTCAATTGAATGGTTTCAAGCCCT
ACCCCCTAGTG
PE2_1
ATTATTAGACAAGAAAGGGGGAAAAAAGAAGCTTTTGGAACCTTAGGAATAATTT
ATGCTATAATAGCTATCGGATTATTAGGATTCGTAGTTTGAGCTCACCATATATTT
ACAGTAGGAATAGATGTAGATACCCGAGCTTACTTCACTTCAGCAACTATAATTA
TTGCGGTTCCAACTGGGATTAAAGTCTTTAGATGACTAGCCACCTTTCATGGAAC
TCAAATTTTAAATAGACCAGTAACCCTGTGAGCTTTAGGTTTTGTATTTTTATTTA
CAGTAGGAGGATTAACAGGGGTAATTTTAGCTAATTCTTCAATTGATATTATTCTC
CACGATACTTACTATGTAGTAGCTCATTTTCACTATGTTCTTTCTATAGGAGCTGT
ATTTGCAATTATAGCCGGAATTGTCCAATGATTCCCATTATTTACCGGACTAACC
TTAAATAATTTTTTTTTAAAAACCCAATTTATTACTATATTTTTAGGTGTAAATTTAA
CATTTTTTCCTCAACACTTTTTAGGGCTAAGAGGAATGCCTCGGCGATATTCGGA
TTATCCAGATGCTTTCACTATCTGAAATGCCATTTCATCTATTGGTTCCATTATTT
CTCTCACAAGAATTATCTTTTTTTTATTTATCTTATGAGAAAGGCTTTCTTCCCAA
CGTAAATCTTTGAGAACTTTAAATGTAACAACTTCAATTGAATGGTTTCAAGCCCT
ACCCCCTAGTG
PE2_2
ATTATTAGACAAGAAAGGGGGAAAAAAGAAGCTTTTGGAACCTTAGGAATAATTT
ATGCTATAATAGCTATCGGATTATTAGGATTCGTAGTTTGAGCTCACCATATATTT
ACAGTAGGAATAGATGTAGATACCCGAGCTTACTTCACTTCAGCAACTATAATTA
TTGCGGTTCCAACTGGGATTAAAGTCTTTAGATGACTAGCCACCTTTCATGGAAC
TCAAATTTTAAATAGACCAGTAACCCTGTGAGCTTTAGGTTTTGTATTTTTATTTA
CAGTAGGAGGATTAACAGGGGTAATTTTAGCTAATTCTTCAATTGATATTATTCTC
CACGATACTTACTATGTAGTAGCTCATTTTCACTATGTTCTTTCTATAGGAGCTGT
ATTTGCAATTATAGCCGGAATTGTCCAATGATTCCCATTATTTACCGGACTAACC
TTAAATAATTTTTTTTTAAAAACCCAATTTATTACTATATTTTTAGGTGTAAATTTAA
CATTTTTTCCTCAACACTTTTTAGGGCTAAGAGGAATGCCTCGGCGATATTCGGA
TTATCCAGATGCTTTCACTATCTGAAATGCCATTTCATCTATTGGTTCCATTATTT
CTCTCACAAGAATTATCTTTTTTTTATTTATCTTATGAGAAAGGCTTTCTTCCCAA
CGTAAATCTTTGAGAACTTTAAATGTAACAACTTCAATTGAATGGTTTCAAGCCCT
ACCCCCTAGTG
PE2_3
ATTATTAGACAAGAAAGGGGGAAAAAAGAAGCTTTTGGAACCTTAGGAATAATTT
ATGCTATAATAGCTATCGGATTATTAGGATTCGTAGTTTGAGCTCACCATATATTT
ACAGTAGGAATAGATGTAGATACCCGAGCTTACTTCACTTCAGCAACTATAATTA
TTGCGGTTCCAACTGGGATTAAAGTCTTTAGATGACTAGCCACCTTTCATGGAAC
TCAAATTTTAAATAGACCAGTAACCCTGTGAGCTTTAGGTTTTGTATTTTTATTTA
CAGTAGGAGGATTAACAGGGGTAATTTTAGCTAATTCTTCAATTGATATTATTCTC

CACGATACTTACTATGTAGTAGCTCATTTTCACTATGTTCTTTCTATAGGAGCTGT
ATTTGCAATTATAGCCGGAATTGTCCAATGATTCCCATTATTTACCGGACTAACC
TTAAATAATTTTTTTTTAAAAACCCAATTTATTACTATATTTTTAGGTGTAAATTTAA
CATTTTTTCCTCAACACTTTTTAGGGCTAAGAGGAATGCCTCGGCGATATTCGGA
TTATCCAGATGCTTTCACTATCTGAAATGCCATTTCATCTATTGGTTCCATTATTT
CTCTCACAAGAATTATCTTTTTTTTATTTATCTTATGAGAAAGGCTTTCTTCCCAA
CGTAAATCTTTGAGAACTTTAAATGTAACAACTTCAATTGAATGGTTTCAAGCCCT
ACCCCCTAGTG
PE2_4
ATTATTAGACAAGAAAGGGGGAAAAAAGAAGCTTTTGGAACCTTAGGAATAATTT
ATGCTATAATAGCTATCGGATTATTAGGATTCGTAGTTTGAGCTCACCATATATTT
ACAGTAGGAATAGATGTAGATACCCGAGCTTACTTCACTTCAGCAACTATAATTA
TTGCGGTTCCAACTGGGATTAAAGTCTTTAGATGACTAGCCACCTTTCATGGAAC
TCAAATTTTAAATAGACCAGTAACCCTGTGAGCTTTAGGTTTTGTATTTTTATTTA
CAGTAGGAGGATTAACAGGGGTAATTTTAGCTAATTCTTCAATTGATATTATTCTC
CACGATACTTACTATGTAGTAGCTCATTTTCACTATGTTCTTTCTATAGGAGCTGT
ATTTGCAATTATAGCCGGAATTGTCCAATGATTCCCATTATTTACCGGACTAACC
TTAAATAATTTTTTTTTAAAAACCCAATTTATTACTATATTTTTAGGTGTAAATTTAA
CATTTTTTCCTCAACACTTTTTAGGGCTAAGAGGAATGCCTCGGCGATATTCGGA
TTATCCAGATGCTTTCACTATCTGAAATGCCATTTCATCTATTGGTTCCATTATTT
CTCTCACAAGAATTATCTTTTTTTTATTTATCTTATGAGAAAGGCTTTCTTCCCAA
CGTAAATCTTTGAGAACTTTAAATGTAACAACTTCAATTGAATGGTTTCAAGCCCT
ACCCCCTAGTG
PE2_5
ATTATTAGACAAGAAAGGGGGAAAAAAGAAGCTTTTGGAACCTTAGGAATAATTT
ATGCTATAATAGCTATCGGATTATTAGGATTCGTAGTTTGAGCTCACCATATATTT
ACAGTAGGAATAGATGTAGATACCCGAGCTTACTTCACTTCAGCAACTATAATTA
TTGCGGTTCCAACTGGGATTAAAGTCTTTAGATGACTAGCCACCTTTCATGGAAC
TCAAATTTTAAATAGACCAGTAACCCTGTGAGCTTTAGGTTTTGTATTTTTATTTA
CAGTAGGAGGATTAACAGGGGTAATTTTAGCTAATTCTTCAATTGATATTATTCTC
CACGATACTTACTATGTAGTAGCTCATTTTCACTATGTTCTTTCTATAGGAGCTGT
ATTTGCAATTATAGCCGGAATTGTCCAATGATTCCCATTATTTACCGGACTAACC
TTAAATAATTTTTTTTTAAAAACCCAATTTATTACTATATTTTTAGGTGTAAATTTAA
CATTTTTTCCTCAACACTTTTTAGGGCTAAGAGGAATGCCTCGGCGATATTCGGA
TTATCCAGATGCTTTCACTATCTGAAATGCCATTTCATCTATTGGTTCCATTATTT
CTCTCACAAGAATTATCTTTTTTTTATTTATCTTATGAGAAAGGCTTTCTTCCCAA
CGTAAATCTTTGAGAACTTTAAATGTAACAACTTCAATTGAATGGTTTCAAGCCCT
ACCCCCTAGTG
PE2_6
ATTATTAGACAAGAAAGGGGGAAAAAAGAAGCTTTTGGAACCTTAGGAATAATTT
ATGCTATAATAGCTATCGGATTATTAGGATTCGTAGTTTGAGCTCACCATATATTT
ACAGTAGGAATAGATGTAGATACCCGAGCTTACTTCACTTCAGCAACTATAATTA
TTGCGGTTCCAACTGGGATTAAAGTCTTTAGATGACTAGCCACCTTTCATGGAAC
TCAAATTTTAAATAGACCAGTAACCCTGTGAGCTTTAGGTTTTGTATTTTTATTTA
CAGTAGGAGGATTAACAGGGGTAATTTTAGCTAATTCTTCAATTGATATTATTCTC
CACGATACTTACTATGTAGTAGCTCATTTTCACTATGTTCTTTCTATAGGAGCTGT
ATTTGCAATTATAGCCGGAATTGTCCAATGATTCCCATTATTTACCGGACTAACC
TTAAATAATTTTTTTTTAAAAACCCAATTTATTACTATATTTTTAGGTGTAAATTTAA
CATTTTTTCCTCAACACTTTTTAGGGCTAAGAGGAATGCCTCGGCGATATTCGGA
TTATCCAGATGCTTTCACTATCTGAAATGCCATTTCATCTATTGGTTCCATTATTT

CTCTCACAAGAATTATCTTTTTTTTATTTATCTTATGAGAAAGGCTTTCTTCCCAA
CGTAAATCTTTGAGAACTTTAAATGTAACAACTTCAATTGAATGGTTTCAAGCCCT
ACCCCCTAGTG
PE2_7
ATTATTAGACAAGAAAGGGGGAAAAAAGAAGCTTTTGGAACCTTAGGAATAATTT
ATGCTATAATAGCTATCGGATTATTAGGATTCGTAGTTTGAGCTCACCATATATTT
ACAGTAGGAATAGATGTAGATACCCGAGCTTACTTCACTTCAGCAACTATAATTA
TTGCGGTTCCAACTGGGATTAAAGTCTTTAGATGACTAGCCACCTTTCATGGAAC
TCAAATTTTAAATAGACCAGTAACCCTGTGAGCTTTAGGTTTTGTATTTTTATTTA
CAGTAGGAGGATTAACAGGGGTAATTTTAGCTAATTCTTCAATTGATATTATTCTC
CACGATACTTACTATGTAGTAGCTCATTTTCACTATGTTCTTTCTATAGGAGCTGT
ATTTGCAATTATAGCCGGAATTGTCCAATGATTCCCATTATTTACCGGACTAACC
TTAAATAATTTTTTTTTAAAAACCCAATTTATTACTATATTTTTAGGTGTAAATTTAA
CATTTTTTCCTCAACACTTTTTAGGGCTAAGAGGAATGCCTCGGCGATATTCGGA
TTATCCAGATGCTTTCACTATCTGAAATGCCATTTCATCTATTGGTTCCATTATTT
CTCTCACAAGAATTATCTTTTTTTTATTTATCTTATGAGAAAGGCTTTCTTCCCAA
CGTAAATCTTTGAGAACTTTAAATGTAACAACTTCAATTGAATGGTTTCAAGCCCT
ACCCCCTAGTG
PE3_1
ATTATTAGACAAGAAAGGGGGAAAAAAGAAGCTTTTGGAACCTTAGGAATAATTT
ATGCTATAATAGCTATCGGATTATTAGGATTCGTAGTTTGAGCTCACCATATATTT
ACAGTAGGAATAGATGTAGATACCCGAGCTTACTTCACTTCAGCAACTATAATTA
TTGCGGTTCCAACTGGGATTAAAGTCTTTAGATGACTAGCCACCTTTCATGGAAC
TCAAATTTTAAATAGACCAGTAACCCTGTGAGCTTTAGGTTTTGTATTTTTATTTA
CAGTAGGAGGATTAACAGGGGTAATTTTAGCTAATTCTTCAATTGATATTATTCTC
CACGATACTTACTATGTAGTAGCTCATTTTCACTATGTTCTTTCTATAGGAGCTGT
ATTTGCAATTATAGCCGGAATTGTCCAATGATTCCCATTATTTACCGGACTAACC
TTAAATAATTTTTTTTTAAAAACCCAATTTATTACTATATTTTTAGGTGTAAATTTAA
CATTTTTTCCTCAACACTTTTTAGGGCTAAGAGGAATGCCTCGGCGATATTCGGA
TTATCCAGATGCTTTCACTATCTGAAATGCCATTTCATCTATTGGTTCCATTATTT
CTCTCACAAGAATTATCTTTTTTTTATTTATCTTATGAGAAAGGCTTTCTTCCCAA
CGTAAATCTTTGAGAACTTTAAATGTAACAACTTCAATTGAATGGTTTCAAGCCCT
ACCCCCTAGTG
PE4_1
ATTATTAGACAAGAAAGGGGGAAAAAAGAAGCTTTTGGAACCTTAGGAATAATTT
ATGCTATAATAGCTATCGGATTATTAGGATTCGTAGTTTGAGCTCACCATATATTT
ACAGTAGGAATAGATGTAGATACCCGAGCTTACTTCACTTCAGCAACTATAATTA
TTGCGGTTCCAACTGGGATTAAAGTCTTTAGATGACTAGCCACCTTTCATGGAAC
TCAAATTTTAAATAGACCAGTAACCCTGTGAGCTTTAGGTTTTGTATTTTTATTTA
CAGTAGGAGGATTAACAGGGGTAATTTTAGCTAATTCTTCAATTGATATTATTCTC
CACGATACTTACTATGTAGTAGCTCATTTTCACTATGTTCTTTCTATAGGAGCTGT
ATTTGCAATTATAGCCGGAATTGTCCAATGATTCCCATTATTTACCGGACTAACC
TTAAATAATTTTTTTTTAAAAACCCAATTTATTACTATATTTTTAGGTGTAAATTTAA
CATTTTTTCCTCAACACTTTTTAGGGCTAAGAGGAATGCCTCGGCGATATTCGGA
TTATCCAGATGCTTTCACTATCTGAAATGCCATTTCATCTATTGGTTCCATTATTT
CTCTCACAAGAATTATCTTTTTTTTATTTATCTTATGAGAAAGGCTTTCTTCCCAA
CGTAAATCTTTGAGAACTTTAAATGTAACAACTTCAATTGAATGGTTTCAAGCCCT
ACCCCCTAGTG
PE4_2
ATTATTAGACAAGAAAGGGGGAAAAAAGAAGCTTTTGGAACCTTAGGAATAATTT

ATGCTATAATAGCTATCGGATTATTAGGATTCGTAGTTTGAGCTCACCATATATTT
ACAGTAGGAATAGATGTAGATACCCGAGCTTACTTCACTTCAGCAACTATAATTA
TTGCGGTTCCAACTGGGATTAAAGTCTTTAGATGACTAGCCACCTTTCATGGAAC
TCAAATTTTAAATAGACCAGTAACCCTGTGAGCTTTAGGTTTTGTATTTTTATTTA
CAGTAGGAGGATTAACAGGGGTAATTTTAGCTAATTCTTCAATTGATATTATTCTC
CACGATACTTACTATGTAGTAGCTCATTTTCACTATGTTCTTTCTATAGGAGCTGT
ATTTGCAATTATAGCCGGAATTGTCCAATGATTCCCATTATTTACCGGACTAACC
TTAAATAATTTTTTTTTAAAAACCCAATTTATTACTATATTTTTAGGTGTAAATTTAA
CATTTTTTCCTCAACACTTTTTAGGGCTAAGAGGAATGCCTCGGCGATATTCGGA
TTATCCAGATGCTTTCACTATCTGAAATGCCATTTCATCTATTGGTTCCATTATTT
CTCTCACAAGAATTATCTTTTTTTTATTTATCTTATGAGAAAGGCTTTCTTCCCAA
CGTAAATCTTTGAGAACTTTAAATGTAACAACTTCAATTGAATGGTTTCAAGCCCT
ACCCCCTAGTG
PE4_3
ATTATTAGACAAGAAAGGGGGAAAAAAGAAGCTTTTGGAACCTTAGGAATAATTT
ATGCTATAATAGCTATCGGATTATTAGGATTCGTAGTTTGAGCTCACCATATATTT
ACAGTAGGAATAGATGTAGATACCCGAGCTTACTTCACTTCAGCAACTATAATTA
TTGCGGTTCCAACTGGGATTAAAGTCTTTAGATGACTAGCCACCTTTCATGGAAC
TCAAATTTTAAATAGACCAGTAACCCTGTGAGCTTTAGGTTTTGTATTTTTATTTA
CAGTAGGAGGATTAACAGGGGTAATTTTAGCTAATTCTTCAATTGATATTATTCTC
CACGATACTTACTATGTAGTAGCTCATTTTCACTATGTTCTTTCTATAGGAGCTGT
ATTTGCAATTATAGCCGGAATTGTCCAATGATTCCCATTATTTACCGGACTAACC
TTAAATAATTTTTTTTTAAAAACCCAATTTATTACTATATTTTTAGGTGTAAATTTAA
CATTTTTTCCTCAACACTTTTTAGGGCTAAGAGGAATGCCTCGGCGATATTCGGA
TTATCCAGATGCTTTCACTATCTGAAATGCCATTTCATCTATTGGTTCCATTATTT
CTCTCACAAGAATTATCTTTTTTTTATTTATCTTATGAGAAAGGCTTTCTTCCCAA
CGTAAATCTTTGAGAACTTTAAATGTAACAACTTCAATTGAATGGTTTCAAGCCCT
ACCCCCTAGTG
PE4_5
ATTATTAGACAAGAAAGGGGGAAAAAAGAAGCTTTTGGAACCTTAGGAATAATTT
ATGCTATAATAGCTATCGGATTATTAGGATTCGTAGTTTGAGCTCACCATATATTT
ACAGTAGGAATAGATGTAGATACCCGAGCTTACTTCACTTCAGCAACTATAATTA
TTGCGGTTCCAACTGGGATTAAAGTCTTTAGATGACTAGCCACCTTTCATGGAAC
TCAAATTTTAAATAGACCAGTAACCCTGTGAGCTTTAGGTTTTGTATTTTTATTTA
CAGTAGGAGGATTAACAGGGGTAATTTTAGCTAATTCTTCAATTGATATTATTCTC
CACGATACTTACTATGTAGTAGCTCATTTTCACTATGTTCTTTCTATAGGAGCTGT
ATTTGCAATTATAGCCGGAATTGTCCAATGATTCCCATTATTTACCGGACTAACC
TTAAATAATTTTTTTTTAAAAACCCAATTTATTACTATATTTTTAGGTGTAAATTTAA
CATTTTTTCCTCAACACTTTTTAGGGCTAAGAGGAATGCCTCGGCGATATTCGGA
TTATCCAGATGCTTTCACTATCTGAAATGCCATTTCATCTATTGGTTCCATTATTT
CTCTCACAAGAATTATCTTTTTTTTATTTATCTTATGAGAAAGGCTTTCTTCCCAA
CGTAAATCTTTGAGAACTTTAAATGTAACAACTTCAATTGAATGGTTTCAAGCCCT
ACCCCCTAGTG
PE_12
ATTATTAGTCAAGAAAGGGGGAAAAAAGAACCTTTTGGAACCTTAGGAATAATTT
ATGCTATAATAGCTATCGGATTATTAGGATTCGTAGTTTGAGCTCACCATATATTT
ACAGTAGGAATAGATGTAGATACCCGAGCTTACTTCACTTCAGCAACTATAATTA
TTGCGGTTCCAACTGGGATTAAAGTCTTTAGATGACTAGCCACCTTTCATGGAAC
TCAAATTTTAAATAGACCAGTAACCCTGTGAGCTTTAGGTTTTGTATTTTTATTTA
CAGTAGGAGGATTAACAGGGGTAATTTTAGCTAATTCTTCAATTGATATTATTCTC

CACGATACTTACTATGTAGTAGCTCATTTTCACTATGTTCTTTCTATAGGAGCTGT
ATTTGCAATTATAGCCGGAATTGTCCAATGATTCCCATTATTTACCGGACTAACC
TTAAATAATTTTTTTTTAAAAACCCAATTTATTACTATATTTTTAGGTGTAAATTTAA
CATTTTTTCCTCAACACTTTTTAGGGCTAAGAGGAATGCCTCGGCGATATTCGGA
TTATCCAGATGCTTTCACTATCTGAAATGCCATTTCATCTATTGGTTCCATTATTT
CTCTCACAAGAATTATCTTTTTTTTATTTATCTTATGAGAAAGGCTTTCTTCCCAA
CGTAAATCTTTGAGAACTTTAAATGTAACAACTTCAATTGAATGGTTTCAAGCCCT
ACCCCCTAGTG
PE_13
ATTATTAGACAAGAAAGGGGGAAAAAAGAACCTTTTGGAACCTTAGGAATAATTT
ATGCTATAATAGCTATCGGATTATTAGGATTCGTAGTTTGAGCTCACCATATATTT
ACAGTAGGAATAGATGTAGATACCCGAGCTTACTTCACTTCAGCAACTATAATTA
TTGCGGTTCCAACTGGGATTAAAGTCTTTAGATGACTAGCCACCTTTCATGGAAC
TCAAATTTTAAATAGACCAGTAACCCTGTGAGCTTTAGGTTTTGTATTTTTATTTA
CAGTAGGAGGATTAACAGGGGTAATTTTAGCTAATTCTTCAATTGATATTATTCTC
CACGATACTTACTATGTAGTAGCTCATTTTCACTATGTTCTTTCTATAGGAGCTGT
ATTTGCAATTATAGCCGGAATTGTCCAATGATTCCCATTATTTACCGGACTAACC
TTAAATAATTTTTTTTTAAAAACCCAATTTATTACTATATTTTTAGGTGTAAATTTAA
CATTTTTTCCTCAACACTTTTTAGGGCTAAGAGGAATGCCTCGGCGATATTCGGA
TTATCCAGATGCTTTCACTATCTGAAATGCCATTTCATCTATTGGTTCCATTATTT
CTCTCACAAGAATTATCTTTTTTTTATTTATCTTATGAGAAAGGCTTTCTTCCCAA
CGTAAATCTTTGAGAACTTTAAATGTAACAACTTCAATTGAATGGTTTCAAGCCCT
ACCCCCTAGTG
PE_15
ATTATTAGACAAGAAAGGGGGAAAAAAGAACCTTTTGGAACCTTAGGAATAATTT
ATGCTATAATACCTATCGGATTATTAGGATTCGTAGTTTGAGCTCACCATATATTT
ACAGTAGGAATAGATGTAGATACCCGAGCTTACTTCACTTCAGCAACTATAATTA
TTGCGGTTCCAACTGGGATTAAAGTCTTTAGATGACTAGCCACCTTTCATGGAAC
TCAAATTTTAAATAGACCAGTAACCCTGTGAGCTTTAGGTTTTGTATTTTTATTTA
CAGTAGGAGGATTAACAGGGGTAATTTTAGCTAATTCTTCAATTGATATTATTCTC
CACGATACTTACTATGTAGTAGCTCATTTTCACTATGTTCTTTCTATAGGAGCTGT
ATTTGCAATTATAGCCGGAATTGTCCAATGATTCCCATTATTTACCGGACTAACC
TTAAATAATTTTTTTTTAAAAACCCAATTTATTACTATATTTTTAGGTGTAAATTTAA
CATTTTTTCCTCAACACTTTTTAGGGCTAAGAGGAATGCCTCGGCGATATTCGGA
TTATCCAGATGCTTTCACTATCTGAAATGCCATTTCATCCATTGGTTCCATTATTT
CTCTCACAAGAATTATCTTTTTTTTATTTATCTTATGAGAAAGGCTTTCTTCCCAA
CGTAAATCTGTGAGAACTTTAAATGTAACAACTTCAATTGAATGGTTTCAAGCCC
TACCCCCTAGTG
PE_16
ATTATTAGACAAGAAAGGGGGAAAAAAGAAGCTTTTGGAACCTTAGGAATAATTT
ATGCTATAATAGCTATCGGATTATTAGGATTCGTAGTTTGAGCTCACCATATATTT
ACAGTAGGAATAGATGTAGATACCCGAGCTTACTTCACTTCAGCAACTATAATTA
TTGCGGTTCCAACTGGGATTAAAGTCTTTAGATGACTAGCCACCTTTCATGGAAC
TCAAATTTTAAATAGACCAGTCACCCTGTGAGCTTTAGGTTTTGTATTTTTATTTA
CAGTAGGAGGATTAACAGGGGTAATTTTAGCTAATTCTTCAATTGATATTATTCTC
CACGATACTTACTATGTAGTAGCTCATTTTCACTATGTTCTTTCTATAGGAGCTGT
ATTTGCAATTATAGCCGGAATTGTCCAATGATTCCCATTATTTACCGGACTAACC
TTAAATAATTTTTTTTTAAAAACCCAATTTATTACTATATTTTTAGGTGTAAATTTAA
CATTTTTTCCTCAACACTTTTTAGGGCTAAGAGGAATGCCTCGGCGATATTCGGA
TTATCCAGATGCTTTCACTATCTGAAATGCCATTTCATCTATTGGTTCCATTATTT

CTCTCACAAGAATTATCTTTTTTTTATTTATCTTATGAGAAAGGCTTTCTTCCCAA
CGTAAATCTTTGAGAACTTTAAATGTAACAACTTCAATTGAATGGTTTCAAGCCCT
ACCCCCTAGTG
PE_19
ATTATTAGACAAGAAAGGGGGAAAAAAGAAGCTTTTGGAACCTTAGGAATAATTT
ATGCTATAATAGCTATCGGATTATTAGGATTCGTAGTTTGAGCTCACCATATATTT
ACAGTAGGAATAGATGTAGATACCCGAGCTTACTTCACTTCAGCAACTATAATTA
TTGCGGTTCCAACTGGGATTAAAGTCTTTAGATGACTAGCCACCTTTCATGGAAC
TCAAATTTTAAATAGACCAGTCACCCTGTGAGCTTTAGGTTTTGTATTTTTATTTA
CAGTAGGAGGATTAACAGGGGTAATTTTAGCTAATTCTTCAATTGATATTATTCTC
CACGATACTTACTATGTAGTAGCTCATTTTCACTATGTTCTTTCTATAGGAGCTGT
ATTTGCAATTATAGCCGGAATTGTCCAATGATTCCCATTATTTACCGGACTAACC
TTAAATAATTTTTTTTTAAAAACCCAATTTATTACTATATTTTTAGGTGTAAATTTAA
CATTTTTTCCTCAACACTTTTTAGGGCTAAGAGGAATGCCTCGGCGATATTCGGA
TTATCCAGATGCTTTCACTATCTGAAATGCCATTTCATCCATTGGTTCCATTATTT
CTCTCACAAGAATTATCTTTTTTTTATTTATCTTATGAGAAAGGCTTTCTTCCCAA
CGTAAATCTTTGAGAACTTTAAATGTAACAACTTCAATTGAATGGTTTCAAGCCCT
ACCCCCTAGTG
PE_2
ATTATTAGACAAGAAAGGGGGAAAAAAGAAGCTTTTGGAACCTTAGGAATAATTT
ATGCTATAATAGCTATCGGATTATTAGGATTCGTAGTTTGAGCTCACCATATATTT
ACAGTAGGAATAGATGTAGATACCCGAGCTTACTTCACTTCAGCAACTATAATTA
TTGCGGTTCCAACTGGGATTAAAGTCTTTAGATGACTAGCCACCTTTCATGGAAC
TCAAATTTTAAATAGACCAGTCACCCTGTGAGCTTTAGGTTTTGTATTTTTATTTA
CAGTAGGAGGATTAACAGGGGTAATTTTAGCTAATTCTTCAATTGATATTATTCTC
CACGATACTTACTATGTAGTAGCTCATTTTCACTATGTTCTTTCTATAGGAGCTGT
ATTTGCAATTATAGCCGGAATTGTCCAATGATTCCCATTATTTACCGGACTAACC
TTAAATAATTTTTTTTTAAAAACCCAATTTATTACTATATTTTTAGGTGTAAATTTAA
CATTTTTTCCTCAACACTTTTTAGGGCTAAGAGGAATGCCTCGGCGATATTCGGA
TTATCCAGATGCTTTCACTATCTGAAATGCCATTTCATCTATTGGTTCCATTATTT
CTCTCACAAGAATTATCTTTTTTTTATTTATCTTATGAGAAAGGCTTTCTTCCCAA
CGTAAATCTTTGAGAACTTTAAATGTAACAACTTCAATTGAATGGTTTCAAGCCCT
ACCCCCTAGTG
PE_23
ATTATTAGACAAGAAAGGGGGAAAAAAGAAGCTTTTGGAACCTTAGGAATAATTT
ATGCTATAATAGCTATCGGATTATTAGGATTCGTAGTTTGAGCTCACCATATATTT
ACAGTAGGAATAGATGTAGATACCCGAGCTTACTTCACTTCAGCAACTATAATTA
TTGCGGTTCCAACTGGGATTAAAGTCTTTAGATGACTAGCCACCTTTCATGGAAC
TCAAATTTTAAATAGACCAGTAACCCTGTGAGCTTTAGGTTTTGTATTTTTATTTA
CAGTAGGAGGATTAACAGGGGTAATTTTAGCTAATTCTTCAATTGATATTATTCTC
CACGATACTTACTATGTAGTAGCTCATTTTCACTATGTTCTTTCTATAGGAGCTGT
ATTTGCAATTATAGCCGGAATTGTCCAATGATTCCCATTATTTACCGGACTAACC
TTAAATAATTTTTTTTTAAAAACCCAATTTATTACTATATTTTTAGGTGTAAATTTAA
CATTTTTTCCTCAACACTTTTTAGGGCTAAGAGGAATGCCTCGGCGATATTCGGA
TTATCCAGATGCTTTCACTATCTGAAATGCCATTTCATCTATTGGTTCCATTATTT
CTCTCACAAGAATTATCTTTTTTTTATTTATCTTATGAGAAAGGCTTTCTTCCCAA
CGTAAATCTTTGAGAACTTTAAATGTAACAACTTCAATTGAATGGTTTCAAGCCCT
ACCCCCTAGTG
PE_29
ATTATTAGACAAGAAAGGGGGAAAAAAGAAGCTTTTGGAACCTTAGGAATAATTT

ATGCTATAATAGCTATCGGATTATTAGGATTCGTAGTTTGAGCTCACCATATATTT
ACAGTAGGAATAGATGTAGATACCCGAGCTTACTTCACTTCAGCAACTATAATTA
TTGCGGTTCCAACTGGGATTAAAGTCTTTAGATGACTAGCCACCTTTCATGGAAC
TCAAATTTTAAATAGACCAGTCACCCTGTGAGCTTTAGGTTTTGTATTTTTATTTA
CAGTAGGAGGATTAACAGGGGTAATTTTAGCTAATTCTTCAATTGATATTATTCTC
CACGATACTTACTATGTAGTAGCTCATTTTCACTATGTTCTTTCTATAGGAGCTGT
ATTTGCAATTATAGCCGGAATTGTCCAATGATTCCCATTATTTACCGGACTAACC
TTAAATAATTTTTTTTTAAAAACCCAATTTATTACTATATTTTTAGGTGTAAATTTAA
CATTTTTTCCTCAACACTTTTTAGGGCTAAGAGGAATGCCTCGGCGATATTCGGA
TTATCCAGATGCTTTCACTATCTGAAATGCCATTTCATCTATTGGTTCCATTATTT
CTCTCACAAGAATTATCTTTTTTTTATTTATCTTATGAGAAAGGCTTTCTTCCCAA
CGTAAATCTTTGAGAACTTTAAATGTAACAACTTCAATTGAATGGTTTCAAGCCCT
ACCCCCTAGTG
PE_6
ATTATTAGACAAGAAAGGGGGAAAAAAGAAGCTTTTGGAACCTTAGGAATAATTT
ATGCTATAATAGCTATCGGATTATTAGGATTCGTAGTTTGAGCTCACCATATATTT
ACAGTAGGAATAGATGTAGATACCCGAGCTTACTTCACTTCAGCAACTATAATTA
TTGCGGTTCCAACTGGGATTAAAGTCTTTAGATGACTAGCCACCTTTCATGGAAC
TCAAATTTTAAATAGACCAGTAACCCTGTGAGCTTTAGGTTTTGTATTTTTATTTA
CAGTAGGAGGATTAACAGGGGTAATTTTAGCTAATTCTTCAATTGATATTATTCTC
CACGATACTTACTATGTAGTAGCTCATTTTCACTATGTTCTTTCTATAGGAGCTGT
ATTTGCAATTATAGCCGGAATTGTCCAATGATTCCCATTATTTACCGGACTAACC
TTAAATAATTTTTTTTTAAAAACCCAATTTATTACTATATTTTTAGGTGTAAATTTAA
CATTTTTTCCTCAACACTTTTTAGGGCTAAGAGGAATGCCTCGGCGATATTCGGA
TTATCCAGATGCTTTCACTATCTGAAATGCCATTTCATCTATTGGTTCCATTATTT
CTCTCACAAGAATTATCTTTTTTTTATTTATCTTATGAGAAAGGCTTTCTTCCCAA
CGTAAATCTTTGAGAACTTTAAATGTAACAACTTCAATTGAATGGTTTCAAGCCCT
ACCCCCTAGTG
PE_8
ATTATTAGACAAGAAAGGGGGAAAAAAGAAGCTTTTGGAACCTTAGGAATAATTT
ATGCTATAATAGCTATCGGATTATTAGGATTCGTAGTTTGAGCTCACCATATATTT
ACAGTAGGAATAGATGTAGATACCCGAGCTTACTTCACTTCAGCAACTATAATTA
TTGCGGTTCCAACTGGGATTAAAGTCTTTAGATGACTAGCCACCTTTCATGGAAC
TCAAATTTTAAATAGACCAGTAACCCTGTGAGCTTTAGGTTTTGTATTTTTATTTA
CAGTAGGAGGATTAACAGGGGTAATTTTAGCTAATTCTTCAATTGATATTATTCTC
CACGATACTTACTATGTAGTAGCTCATTTTCACTATGTTCTTTCTATAGGAGCTGT
ATTTGCAATTATAGCCGGAATTGTCCAATGATTCCCATTATTTACCGGACTAACC
TTAAATAATTTTTTTTTAAAAACCCAATTTATTACTATATTTTTAGGTGTAAATTTAA
CATTTTTTCCTCAACACTTTTTAGGGCTAAGAGGAATGCCTCGGCGATATTCGGA
TTATCCAGATGCTTTCACTATCTGAAATGCCATTTCATCTATTGGTTCCATTATTT
CTCTCACAAGAATTATCTTTTTTTTATTTATCTTATGAGAAAGGCTTTCTTCCCAA
CGTAAATCTTTGAGAACTTTAAATGTAACAACTTCAATTGAATGGTTTCAAGCCCT
ACCCCCTAGTG
CL1_1
ATTATTAGACAAGAAAGGGGGAAAAAAGAAGCTTTTGGAACCTTAGGAATAATTT
ATGCTATAATAGCTATCGGATTATTAGGATTCGTAGTTTGAGCTCACCATATATTT
ACAGTAGGAATAGATGTAGATACCCGAGCTTACTTCACTTCAGCAACTATAATTA
TTGCGGTTCCAACTGGGATTAAAGTCTTTAGATGACTAGCCACCTTTCATGGAAC
TCAAATTTTAAATAGACCAGTAACCCTGTGAGCTTTAGGTTTTGTATTTTTATTTA
CAGTAGGAGGATTAACAGGGGTAATTTTAGCTAATTCTTCAATTGATATTATTCTC

CACGATACTTACTATGTAGTAGCTCATTTTCACTATGTTCTTTCTATAGGAGCTGT
ATTTGCAATTATAGCCGGAATTGTCCAATGATTCCCATTATTTACCGGACTAACC
TTAAATAATTTTTTTTTAAAAACCCAATTTATTACTATATTTTTAGGTGTAAATTTAA
CATTTTTTCCTCAACACTTTTTTAGGGCTAAGAGGAATGCCTCGGCGATATTCGGA
TTATCCAGATGCTTTCACTATCTGAAATGCCATTTCATCTATTGGTTCCATTATTT
CTCTCACAAGAATTATCTTTTTTTTTATTTATCTTATGAGAAAGGCTTTCTTCCCAA
CGTAAATCTTTGAGAACTTTAAATGTAACAACTTCAATTGAATGGTTTCAAGCCCT
ACCCCCTAGTG
CL1_2
ATTATTAGACAAGAAAGGGGGAAAAAAGAAGCTTTTGGAACCTTAGGAATAATTT
ATGCTATAATAGCTATCGGATTATTAGGATTCGTAGTTTGAGCTCACCATATATTT
ACAGTAGGAATAGATGTAGATACCCGAGCTTACTTCACTTCAGCAACTATAATTA
TTGCGGTTCCAACTGGGATTAAAGTCTTTAGATGACTAGCCACCTTTCATGGAAC
TCAAATTTTAAATAGACCAGTAACCCTGTGAGCTTTAGGTTTTGTATTTTTATTTA
CAGTAGGAGGATTAACAGGGGTAATTTTAGCTAATTCTTCAATTGATATTATTCTC
CACGATACTTACTATGTAGTAGCTCATTTTCACTATGTTCTTTCTATAGGAGCTGT
ATTTGCAATTATAGCCGGAATTGTCCAATGATTCCCATTATTTACCGGACTAACC
TTAAATAATTTTTTTTTAAAAACCCAATTTATTACCATATTTTTAGGTGTAAATTTAA
CATTTTTTCCTCAACACTTTTTTAGGGCTAAGAGGAATGCCTCGGCGATATTCGGA
TTATCCAGATGCTTTCACTATCTGAAATGCCATTTCATCTATTGGTTCCATTATTT
CTCTCACAAGAATTATCTTTTTTTTTATTTATCTTATGAGAAAGGCTTTCTTCCCAA
CGTAAATCTTTGAGAACTTTAAATGTAACAACTTCAATTGAATGGTTTCAAGCCCT
ACCCCCTAGTG
CL1_3
ATTATTAGACAAGAAAGGGGGAAAAAAGAAGCTTTTGGAACCTTAGGAATAATTT
ATGCTATAATAGCTATCGGATTATTAGGATTCGTAGTTTGAGCTCACCATATATTT
ACAGTAGGAATAGATGTAGATACCCGAGCTTACTTCACTTCAGCAACTATAATTA
TTGCGGTTCCAACTGGGATTAAAGTCTTTAGATGACTAGCCACCTTTCATGGAAC
TCAAATTTTAAATAGACCAGTAACCCTGTGAGCTTTAGGTTTTGTATTTTTATTTA
CAGTAGGAGGATTAACAGGGGTAATTTTAGCTAATTCTTCAATTGATATTATTCTC
CACGATACTTACTATGTAGTAGCTCATTTTCACTATGTTCTTTCTATAGGAGCTGT
ATTTGCAATTATAGCCGGAATTGTCCAATGATTCCCATTATTTACCGGACTAACC
TTAAATAATTTTTTTTTAAAAACCCAATTTATTACTATATTTTTAGGTGTAAATTTAA
CATTTTTTCCTCAACACTTTTTTAGGGCTAAGAGGAATGCCTCGGCGATATTCGGA
TTATCCAGATGCTTTCACTATCTGAAATGCCATTTCATCTATTGGTTCCATTATTT
CTCTCACAAGAATTATCTTTTTTTTTATTTATCTTATGAGAAAGGCTTTCTTCCCAA
CGTAAATCTTTGAGAACTTTAAATGTAACAACTTCAATTGAATGGTTTCAAGCCCT
ACCCCCTAGTG
CL1_4
ATTATTAGACAAGAAAGGGGGAAAAAAGAAGCTTTTGGAACCTTAGGAATAATTT
ATGCTATAATAGCTATCGGATTATTAGGATTCGTAGTTTGAGCTCACCATATATTT
ACAGTAGGAATAGATGTAGATACCCGAGCTTACTTCACTTCAGCAACTATAATTA
TTGCGGTTCCAACTGGGATTAAAGTCTTTAGATGACTAGCCACCTTTCATGGAAC
TCAAATTTTAAATAGACCAGTAACCCTGTGAGCTTTAGGTTTTGTATTTTTATTTA
CAGTAGGAGGATTAACAGGGGTAATTTTAGCTAATTCTTCAATTGATATTATTCTC
CACGATACTTACTATGTAGTAGCTCATTTTCACTATGTTCTTTCTATAGGAGCTGT
ATTTGCAATTATAGCCGGAATTGTCCAATGATTCCCATTATTTACCGGACTAACC
TTAAATAATTTTTTTTTAAAAACCCAATTTATTACTATATTTTTAGGTGTAAATTTAA
CATTTTTTCCTCAACACTTTTTTAGGGCTAAGAGGAATGCCTCGGCGATATTCGGA
TTATCCAGATGCTTTCACTATCTGAAATGCCATTTCATCTATTGGTTCCATTATTT

CTCTCACAAGAATTATCTTTTTTTTATTTATCTTATGAGAAAGGCTTTCTTCCCAA
CGTAAATCTTTGAGAACTTTAAATGTAACAACTTCAATTGAATGGTTTCAAGCCCT
ACCCCCTAGTG
CL1_5
ATTATTAGACAAGAAAGGGGGAAAAAAGAAGCTTTTGGAACCTTAGGAATAATTT
ATGCTATAATAGCTATCGGATTATTAGGATTCGTAGTTTGAGCTCACCATATATTT
ACAGTAGGAATAGATGTAGATACCCGAGCTTACTTCACTTCAGCAACTATAATTA
TTGCGGTTCCAACTGGGATTAAAGTCTTTAGATGACTAGCCACCTTTCATGGAAC
TCAAATTTTAAATAGACCAGTAACCCTGTGAGCTTTAGGTTTTGTATTTTTATTTA
CAGTAGGAGGATTAACAGGGGTAATTTTAGCTAATTCTTCAATTGATATTATTCTC
CACGATACTTACTATGTAGTAGCTCATTTTCACTATGTTCTTTCTATAGGAGCTGT
ATTTGCAATTATAGCCGGAATTGTCCAATGATTCCCATTATTTACCGGACTAACC
TTAAATAATTTTTTTTTAAAAACCCAATTTATTACCATATTTTTAGGTGTAAATTTAA
CATTTTTTCCTCAACACTTTTTAGGGCTAAGAGGAATGCCTCGGCGATATTCGGA
TTATCCAGATGCTTTCACTATCTGAAATGCCATTTCATCTATTGGTTCCATTATTT
CTCTCACAAGAATTATCTTTTTTTTATTTATCTTATGAGAAAGGCTTTCTTCCCAA
CGTAAATCTTTGAGAACTTTAAATGTAACAACTTCAATTGAATGGTTTCAAGCCCT
ACCCCCTAGTG
CL1_6
ATTATTAGACAAGAAAGGGGGAAAAAAGAAGCTTTTGGAACCTTAGGAATAATTT
ATGCTATAATAGCTATCGGATTATTAGGATTCGTAGTTTGAGCTCACCATATATTT
ACAGTAGGAATAGATGTAGATACCCGAGCTTACTTCACTTCAGCAACTATAATTA
TTGCGGTTCCAACTGGGATTAAAGTCTTTAGATGACTAGCCACCTTTCATGGAAC
TCAAATTTTAAATAGACCAGTAACCCTGTGAGCTTTAGGTTTTGTATTTTTATTTA
CAGTAGGAGGATTAACAGGGGTAATTTTAGCTAATTCTTCAATTGATATTATTCTC
CACGATACTTACTATGTAGTAGCTCATTTTCACTATGTTCTTTCTATAGGAGCTGT
ATTTGCAATTATAGCCGGAATTGTCCAATGATTCCCATTATTTACCGGACTAACC
TTAAATAATTTTTTTTTAAAAACCCAATTTATTACCATATTTTTAGGTGTAAATTTAA
CATTTTTTCCTCAACACTTTTTAGGGCTAAGAGGAATGCCTCGGCGATATTCGGA
TTATCCAGATGCTTTCACTATCTGAAATGCCATTTCATCTATTGGTTCCATTATTT
CTCTCACAAGAATTATCTTTTTTTTATTTATCTTATGAGAAAGGCTTTCTTCCCAA
CGTAAATCTTTGAGAACTTTAAATGTAACAACTTCAATTGAATGGTTTCAAGCCCT
ACCCCCTAGTG
CL1_7
ATTATTAGACAAGAAAGGGGGAAAAAAGAAGCTTTTGGAACCTTAGGAATAATTT
ATGCTATAATAGCTATCGGATTATTAGGATTCGTAGTTTGAGCTCACCATATATTT
ACAGTAGGAATAGATGTAGATACCCGAGCTTACTTCACTTCAGCAACTATAATTA
TTGCGGTTCCAACTGGGATTAAAGTCTTTAGATGACTAGCCACCTTTCATGGAAC
TCAAATTTTAAATAGACCAGTAACCCTGTGAGCTTTAGGTTTTGTATTTTTATTTA
CAGTAGGAGGATTAACAGGGGTAATTTTAGCTAATTCTTCAATTGATATTATTCTC
CACGATACTTACTATGTAGTAGCTCATTTTCACTATGTTCTTTCTATAGGAGCTGT
ATTTGCAATTATAGCCGGAATTGTCCAATGATTCCCATTATTTACCGGACTAACC
TTAAATAATTTTTTTTTAAAAACCCAATTTATTACTATATTTTTAGGTGTAAATTTAA
CATTTTTTCCTCAACACTTTTTAGGGCTAAGAGGAATGCCTCGGCGATATTCGGA
TTATCCAGATGCTTTCACTATCTGAAATGCCATTTCATCTATTGGTTCCATTATTT
CTCTCACAAGAATTATCTTTTTTTTATTTATCTTATGAGAAAGGCTTTCTTCCCAA
CGTAAATCTTTGAGAACTTTAAATGTAACAACTTCAATTGAATGGTTTCAAGCCCT
ACCCCCTAGTG
CL1_8
ATTATTAGACAAGAAAGGGGGAAAAAAGAAGCTTTTGGAACCTTAGGAATAATTT

ATGCTATAATAGCTATCGGATTATTAGGATTCGTAGTTTGAGCTCACCATATATTT
ACAGTAGGAATAGATGTAGATACCCGAGCTTACTTCACTTCAGCAACTATAATTA
TTGCGGTTCCAACTGGGATTAAAGTCTTTAGATGACTAGCCACCTTTCATGGAAC
TCAAATTTTAAATAGACCAGTAACCCTGTGAGCTTTAGGTTTTGTATTTTTATTTA
CAGTAGGAGGATTAACAGGGGTAATTTTAGCTAATTCTTCAATTGATATTATTCTC
CACGATACTTACTATGTAGTAGCTCATTTTCACTATGTTCTTTCTATAGGAGCTGT
ATTTGCAATTATAGCCGGAATTGTCCAATGATTCCCATTATTTACCGGACTAACC
TTAAATAATTTTTTTTTAAAAACCCAATTTATTACCATATTTTTAGGTGTAAATTTAA
CATTTTTTCCTCAACACTTTTTAGGGCTAAGAGGAATGCCTCGGCGATATTCGGA
TTATCCAGATGCTTTCACTATCTGAAATGCCATTTCATCTATTGGTTCCATTATTT
CTCTCACAAGAATTATCTTTTTTTTTATTTATCTTATGAGAAAGGCTTTCTTCCCAA
CGTAAATCTTTGAGAACTTTAAATGTAACAACTTCAATTGAATGGTTTCAAGCCCT
ACCCCCTAGTG
CL1_9
ATTATTAGACAAGAAAGGGGGAAAAAAGAAGCTTTTGGAACCTTAGGAATAATTT
ATGCTATAATAGCTATCGGATTATTAGGATTCGTAGTTTGAGCTCACCATATATTT
ACAGTAGGAATAGATGTAGATACCCGAGCTTACTTCACTTCAGCAACTATAATTA
TTGCGGTTCCAACTGGGATTAAAGTCTTTAGATGACTAGCCACCTTTCATGGAAC
TCAAATTTTAAATAGACCAGTAACCCTGTGAGCTTTAGGTTTTGTATTTTTATTTA
CAGTAGGAGGATTAACAGGGGTAATTTTAGCTAATTCTTCAATTGATATTATTCTC
CACGATACTTACTATGTAGTAGCTCATTTTCACTATGTTCTTTCTATAGGAGCTGT
ATTTGCAATTATAGCCGGAATTGTCCAATGATTCCCATTATTTACCGGACTAACC
TTAAATAATTTTTTTTTAAAAACCCAATTTATTACTATATTTTTAGGTGTAAATTTAA
CATTTTTTCCTCAACACTTTTTAGGGCTAAGAGGAATGCCTCGGCGATATTCGGA
TTATCCAGATGCTTTCACTATCTGAAATGCCATTTCATCTATTGGTTCCATTATTT
CTCTCACAAGAATTATCTTTTTTTTTATTTATCTTATGAGAAAGGCTTTCTTCCCAA
CGTAAATCTTTGAGAACTTTAAATGTAACAACTTCAATTGAATGGTTTCAAGCCCT
ACCCCCTAGTG
CL2_3
ATTATTAGACAAGAAAGGGGGAAAAAAGAAGCTTTTGGAACCTTAGGAATAATTT
ATGCTATAATAGCTATCGGATTATTAGGATTCGTAGTTTGAGCTCACCATATATTT
ACAGTAGGAATAGATGTAGATACCCGAGCTTACTTCACTTCAGCAACTATAATTA
TTGCGGTTCCAACTGGGATTAAAGTCTTTAGATGACTAGCCACCTTTCATGGAAC
TCAAATTTTAAATAGACCAGTAACCCTGTGAGCTTTAGGTTTTGTATTTTTATTTA
CAGTAGGAGGATTAACAGGGGTAATTTTAGCTAATTCTTCAATTGATATTATTCTC
CACGATACTTACTATGTAGTAGCTCATTTTCACTATGTTCTTTCTATAGGAGCTGT
ATTTGCAATTATAGCCGGAATTGTCCAATGATTCCCATTATTTACCGGACTAACC
TTAAATAATTTTTTTTTAAAAACCCAATTTATTACTATATTTTTAGGTGTAAATTTAA
CATTTTTTCCTCAACACTTTTTAGGGCTAAGAGGAATGCCTCGGCGATATTCGGA
TTATCCAGATGCTTTCACTATCTGAAATGCCATTTCATCTATTGGTTCCATTATTT
CTCTCACAAGAATTATCTTTTTTTTTATTTATCTTATGAGAAAGGCTTTCTTCCCAA
CGTAAATCTTTGAGAACTTTAAATGTAACAACTTCAATTGAATGGTTTCAAGCCCT
ACCCCCTAGTG
CL2_4
ATTATTAGACAAGAAAGGGGGAAAAAAGAAGCTTTTGGAACCTTAGGAATAATTT
ATGCTATAATAGCTATCGGATTATTAGGATTCGTAGTTTGAGCTCACCATATATTT
ACAGTAGGAATAGATGTAGATACCCGAGCTTACTTCACTTCAGCAACTATAATTA
TTGCGGTTCCAACTGGGATTAAAGTCTTTAGATGACTAGCCACCTTTCATGGAAC
TCAAATTTTAAATAGACCAGTAACCCTGTGAGCTTTAGGTTTTGTATTTTTATTTA
CAGTAGGAGGATTAACAGGGGTAATTTTAGCTAATTCTTCAATTGATATTATTCTC

CACGATACTTACTATGTAGTAGCTCATTTTCACTATGTTCTTTCTATAGGAGCTGT
ATTTGCAATTATAGCCGGAATTGTCCAATGATTCCCATTATTTACCGGACTAACC
TTAAATAATTTTTTTTTAAAAACCCAATTTATTACTATATTTTTAGGTGTAAATTTAA
CATTTTTTCCTCAACACTTTTTAGGGCTAAGAGGAATGCCTCGGCGATATTCGGA
TTATCCAGATGCTTTCACTATCTGAAATGCCATTTCATCTATTGGTTCCATTATTT
CTCTCACAAGAATTATCTTTTTTTTATTTATCTTATGAGAAAGGCTTTCTTCCCAA
CGTAAATCTTTGAGAACTTTAAATGTAACAACTTCAATTGAATGGTTTCAAGCCCT
ACCCCCTAGTG
CL2_5
ATTATTAGACAAGAAAGGGGGAAAAAAGAAGCTTTTGGAACCTTAGGAATAATTT
ATGCTATAATAGCTATCGGATTATTAGGATTCGTAGTTTGAGCTCACCATATATTT
ACAGTAGGAATAGATGTAGATACCCGAGCTTACTTCACTTCAGCAACTATAATTA
TTGCGGTTCCAACTGGGATTAAAGTCTTTAGATGACTAGCCACCTTTCATGGAAC
TCAAATTTTAAATAGACCAGTAACCCTGTGAGCTTTAGGTTTTGTATTTTTATTTA
CAGTAGGAGGATTAACAGGGGTAATTTTAGCTAATTCTTCAATTGATATTATTCTC
CACGATACTTACTATGTAGTAGCTCATTTTCACTATGTTCTTTCTATAGGAGCTGT
ATTTGCAATTATAGCCGGAATTGTCCAATGATTCCCATTATTTACCGGACTAACC
TTAAATAATTTTTTTTTAAAAACCCAATTTATTACTATATTTTTAGGTGTAAATTTAA
CATTTTTTCCTCAACACTTTTTAGGGCTAAGAGGAATGCCTCGGCGATATTCGGA
TTATCCAGATGCTTTCACTATCTGAAATGCCATTTCATCTATTGGTTCCATTATTT
CTCTCACAAGAATTATCTTTTTTTTATTTATCTTATGAGAAAGGCTTTCTTCCCAA
CGTAAATCTTTGAGAACTTTAAATGTAACAACTTCAATTGAATGGTTTCAAGCCCT
ACCCCCTAGTG
CL2_6
ATTATTAGACAAGAAAGGGGGAAAAAAGAAGCTTTTGGAACCTTAGGAATAATTT
ATGCTATAATAGCTATCGGATTATTAGGATTCGTAGTTTGAGCTCACCATATATTT
ACAGTAGGAATAGATGTAGATACCCGAGCTTACTTCACTTCAGCAACTATAATTA
TTGCGGTTCCAACTGGGATTAAAGTCTTTAGATGACTAGCCACCTTTCATGGAAC
TCAAATTTTAAATAGACCAGTAACCCTGTGAGCTTTAGGTTTTGTATTTTTATTTA
CAGTAGGAGGATTAACAGGGGTAATTTTAGCTAATTCTTCAATTGATATTATTCTC
CACGATACTTACTATGTAGTAGCTCATTTTCACTATGTTCTTTCTATAGGAGCTGT
ATTTGCAATTATAGCCGGAATTGTCCAATGATTCCCATTATTTACCGGACTAACC
TTAAATAATTTTTTTTTAAAAACCCAATTTATTACTATATTTTTAGGTGTAAATTTAA
CATTTTTTCCTCAACACTTTTTAGGGCTAAGAGGAATGCCTCGGCGATATTCGGA
TTATCCAGATGCTTTCACTATCTGAAATGCCATTTCATCTATTGGTTCCATTATTT
CTCTCACAAGAATTATCTTTTTTTTATTTATCTTATGAGAAAGGCTTTCTTCCCAA
CGTAAATCTTTGAGAACTTTAAATGTAACAACTTCAATTGAATGGTTTCAAGCCCT
ACCCCCTAGTG
CL2_7
ATTATTAGACAAGAAAGGGGGAAAAAAGAAGCTTTTGGAACCTTAGGAATAATTT
ATGCTATAATAGCTATCGGATTATTAGGATTCGTAGTTTGAGCTCACCATATATTT
ACAGTAGGAATAGATGTAGATACCCGAGCTTACTTCACTTCAGCAACTATAATTA
TTGCGGTTCCAACTGGGATTAAAGTCTTTAGATGACTAGCCACCTTTCATGGAAC
TCAAATTTTAAATAGACCAGTAACCCTGTGAGCTTTAGGTTTTGTATTTTTATTTA
CAGTAGGAGGATTAACAGGGGTAATTTTAGCTAATTCTTCAATTGATATTATTCTC
CACGATACTTACTATGTAGTAGCTCATTTTCACTATGTTCTTTCTATAGGAGCTGT
ATTTGCAATTATAGCCGGAATTGTCCAATGATTCCCATTATTTACCGGACTAACC
TTAAATAATTTTTTTTTAAAAACCCAATTTATTACTATATTTTTAGGTGTAAATTTAA
CATTTTTTCCTCAACACTTTTTAGGGCTAAGAGGAATGCCTCGGCGATATTCGGA
TTATCCAGATGCTTTCACTATCTGAAATGCCATTTCATCTATTGGTTCCATTATTT

CTCTCACAAGAATTATCTTTTTTTTATTTATCTTATGAGAAAGGCTTTCTTCCCAA
CGTAAATCTTTGAGAACTTTAAATGTAACAACTTCAATTGAATGGTTTCAAGCCCT
ACCCCCTAGTG
CL2_8
ATTATTAGACAAGAAAGGGGGAAAAAAGAAGCTTTTGGAACCTTAGGAATAATTT
ATGCTATAATAGCTATCGGATTATTAGGATTCGTAGTTTGAGCTCACCATATATTT
ACAGTAGGAATAGATGTAGATACCCGAGCTTACTTCACTTCAGCAACTATAATTA
TTGCGGTTCCAACTGGGATTAAAGTCTTTAGATGACTAGCCACCTTTCATGGAAC
TCAAATTTTAAATAGACCAGTAACCCTGTGAGCTTTAGGTTTTGTATTTTTATTTA
CAGTAGGAGGATTAACAGGGGTAATTTTAGCTAATTCTTCAATTGATATTATTCTC
CACGATACTTACTATGTAGTAGCTCATTTTCACTATGTTCTTTCTATAGGAGCTGT
ATTTGCAATTATAGCCGGAATTGTCCAATGATTCCCATTATTTACCGGACTAACC
TTAAATAATTTTTTTTTAAAAACCCAATTTATTACTATATTTTTAGGTGTAAATTTAA
CATTTTTTCCTCAACACTTTTTAGGGCTAAGAGGAATGCCTCGGCGATATTCGGA
TTATCCAGATGCTTTCACTATCTGAAATGCCATTTCATCTATTGGTTCCATTATTT
CTCTCACAAGAATTATCTTTTTTTTATTTATCTTATGAGAAAGGCTTTCTTCCCAA
CGTAAATCTTTGAGAACTTTAAATGTAACAACTTCAATTGAATGGTTTCAAGCCCT
ACCCCCTAGTG
CL2_1
ATTATTAGACAAGAAAGGGGGAAAAAAGAAGCTTTTGGAACCTTAGGAATAATTT
ATGCTATAATAGCTATCGGATTATTAGGATTCGTAGTTTGAGCTCACCATATATTT
ACAGTAGGAATAGATGTAGATACCCGAGCTTACTTCACTTCAGCAACTATAATTA
TTGCGGTTCCAACTGGGATTAAAGTCTTTAGATGACTAGCCACCTTTCATGGAAC
TCAAATTTTAAATAGACCAGTAACCCTGTGAGCTTTAGGTTTTGTATTTTTATTTA
CAGTAGGAGGATTAACAGGGGTAATTTTAGCTAATTCTTCAATTGATATTATTCTC
CACGATACTTACTATGTAGTAGCTCATTTTCACTATGTTCTTTCTATAGGAGCTGT
ATTTGCAATTATAGCCGGAATTGTCCAATGATTCCCATTATTTACCGGACTAACC
TTAAATAATTTTTTTTTAAAAACCCAATTTATTACTATATTTTTAGGTGTAAATTTAA
CATTTTTTCCTCAACACTTTTTAGGGCTAAGAGGAATGCCTCGGCGATATTCGGA
TTATCCAGATGCTTTCACTATCTGAAATGCCATTTCATCTATTGGTTCCATTATTT
CTCTCACAAGAATTATCTTTTTTTTATTTATCTTATGAGAAAGGCTTTCTTCCCAA
CGTAAATCTTTGAGAACTTTAAATGTAACAACTTCAATTGAATGGTTTCAAGCCCT
ACCCCCTAGTG
CL2_2
ATTATTAGACAAGAAAGGGGGAAAAAAGAAGCTTTTGGAACCTTAGGAATAATTT
ATGCTATAATAGCTATCGGATTATTAGGATTCGTAGTTTGAGCTCACCATATATTT
ACAGTAGGAATAGATGTAGATACCCGAGCTTACTTCACTTCAGCAACTATAATTA
TTGCGGTTCCAACTGGGATTAAAGTCTTTAGATGACTAGCCACCTTTCATGGAAC
TCAAATTTTAAATAGACCAGTAACCCTGTGAGCTTTAGGTTTTGTATTTTTATTTA
CAGTAGGAGGATTAACAGGGGTAATTTTAGCTAATTCTTCAATTGATATTATTCTC
CACGATACTTACTATGTAGTAGCTCATTTTCACTATGTTCTTTCTATAGGAGCTGT
ATTTGCAATTATAGCCGGAATTGTCCAATGATTCCCATTATTTACCGGACTAACC
TTAAATAATTTTTTTTTAAAAACCCAATTTATTACTATATTTTTAGGTGTAAATTTAA
CATTTTTTCCTCAACACTTTTTAGGGCTAAGAGGAATGCCTCGGCGATATTCGGA
TTATCCAGATGCTTTCACTATCTGAAATGCCATTTCATCTATTGGTTCCATTATTT
CTCTCACAAGAATTATCTTTTTTTTATTTATCTTATGAGAAAGGCTTTCTTCCCAA
CGTAAATCTTTGAGAACTTTAAATGTAACAACTTCAATTGAATGGTTTCAAGCCCT
ACCCCCTAGTG
EQ1_3
ATTATTAGACAAGAAAGGGGGAAAAAAGAAGCTTTTGGAACCTTAGGAATAATTT

ATGCTATAATAGCTATCGGATTATTAGGATTCGTAGTTTGAGCTCACCATATATTT
ACAGTAGGAATAGATGTAGATACCCGAGCTTACTTCACTTCAGCAACTATAATTA
TTGCGGTTCCAACTGGGATTAAAGTCTTTAGATGACTAGCCACCTTTCATGGAAC
TCAAATTTTAAATAGACCAGTAACCCTGTGAGCTTTAGGTTTTGTATTTTTATTTA
CAGTAGGAGGATTAACAGGGGTAATTTTAGCTAATTCTTCAATTGATATTATTCTC
CACGATACTTACTATGTAGTAGCTCATTTTCACTATGTTCTTTCTATAGGAGCTGT
ATTTGCAATTATAGCCGGAATTGTCCAATGATTCCCATTATTTACCGGACTAACC
TTAAATAATTTTTTTTTAAAAACCCAATTTATTACTATATTTTTAGGTGTAAATTTAA
CATTTTTTCCTCAACACTTTTTAGGGCTAAGAGGAATGCCTCGGCGATATTCGGA
TTATCCAGATGCTTTCACTATCTGAAATGCCATTTCATCTATTGGTTCCATTATTT
CTCTCACAAGAATTATCTTTTTTTTTATTTATCTTATGAGAAAGGCTTTCTTCCCAA
CGTAAATCTTTGAGAACTTTAAATGTAACAACTTCAATTGAATGGTTTCAAGCCCT
ACCCCCTAGTG
EQ1_6
ATTATTAGACAAGAAAGGGGGAAAAAAGAAGCTTTTGGAACCTTAGGAATAATTT
ATGCTATAATAGCTATCGGATTATTAGGATTCGTAGTTTGAGCTCACCATATATTT
ACAGTAGGAATAGATGTAGATACCCGAGCTTACTTCACTTCAGCAACTATAATTA
TTGCGGTTCCAACTGGGATTAAAGTCTTTAGATGACTAGCCACCTTTCATGGAAC
TCAAATTTTAAATAGACCAGTAACCCTGTGAGCTTTAGGTTTTGTATTTTTATTTA
CAGTAGGAGGATTAACAGGGGTAATTTTAGCTAATTCTTCAATTGATATTATTCTC
CACGATACTTACTATGTAGTAGCTCATTTTCACTATGTTCTTTCTATAGGAGCTGT
ATTTGCAATTATAGCCGGAATTGTCCAATGATTCCCATTATTTACCGGACTAACC
TTAAATAATTTTTTTTTAAAAACCCAATTTATTACTATATTTTTAGGTGTAAATTTAA
CATTTTTTCCTCAACACTTTTTAGGGCTAAGAGGAATGCCTCGGCGATATTCGGA
TTATCCAGATGCTTTCACTATCTGAAATGCCATTTCATCTATTGGTTCCATTATTT
CTCTCACAAGAATTATCTTTTTTTTTATTTATCTTATGAGAAAGGCTTTCTTCCCAA
CGTAAATCTTTGAGAACTTTAAATGTAACAACTTCAATTGAATGGTTTCAAGCCCT
ACCCCCTAGTG
EQ1_7
ATTATTAGACAAGAAAGGGGGAAAAAAGAAGCTTTTGGAACCTTAGGAATAATTT
ATGCTATAATAGCTATCGGATTATTAGGATTCGTAGTTTGAGCTCACCATATATTT
ACAGTAGGAATAGATGTAGATACCCGAGCTTACTTCACTTCAGCAACTATAATTA
TTGCGGTTCCAACTGGGATTAAAGTCTTTAGATGACTAGCCACCTTTCATGGAAC
TCAAATTTTAAATAGACCAGTAACCCTGTGAGCTTTAGGTTTTGTATTTTTATTTA
CAGTAGGAGGATTAACAGGGGTAATTTTAGCTAATTCTTCAATTGATATTATTCTC
CACGATACTTACTATGTAGTAGCTCATTTTCACTATGTTCTTTCTATAGGAGCTGT
ATTTGCAATTATAGCCGGAATTGTCCAATGATTCCCATTATTTACCGGACTAACC
TTAAATAATTTTTTTTTAAAAACCCAATTTATTACTATATTTTTAGGTGTAAATTTAA
CATTTTTTCCTCAACACTTTTTAGGGCTAAGAGGAATGCCTCGGCGATATTCGGA
TTATCCAGATGCTTTCACTATCTGAAATGCCATTTCATCTATTGGTTCCATTATTT
CTCTCACAAGAATTATCTTTTTTTTTATTTATCTTATGAGAAAGGCTTTCTTCCCAA
CGTAAATCTTTGAGAACTTTAAATGTAACAACTTCAATTGAATGGTTTCAAGCCCT
ACCCCCTAGTG
EQ1_8
ATTATTAGACAAGAAAGGGGGAAAAAAGAAGCTTTTGGAACCTTAGGAATAATTT
ATGCTATAATAGCTATCGGATTATTAGGATTCGTAGTTTGAGCTCACCATATATTT
ACAGTAGGAATAGATGTAGATACCCGAGCTTACTTCACTTCAGCAACTATAATTA
TTGCGGTTCCAACTGGGATTAAAGTCTTTAGATGACTAGCCACCTTTCATGGAAC
TCAAATTTTAAATAGACCAGTAACCCTGTGAGCTTTAGGTTTTGTATTTTTATTTA
CAGTAGGAGGATTAACAGGGGTAATTTTAGCTAATTCTTCAATTGATATTATTCTC

CACGATACTTACTATGTAGTAGCTCATTTTCACTATGTTCTTTCTATAGGAGCTGT
ATTTGCAATTATAGCCGGAATTGTCCAATGATTCCCATTATTTACCGGACTAACC
TTAAATAATTTTTTTTTAAAAACCCAATTTATTACTATATTTTTAGGTGTAAATTTAA
CATTTTTTCCTCAACACTTTTTAGGGCTAAGAGGAATGCCTCGGCGATATTCGGA
TTATCCAGATGCTTTCACTATCTGAAATGCCATTTCATCTATTGGTTCCATTATTT
CTCTCACAAGAATTATCTTTTTTTTATTTATCTTATGAGAAAGGCTTTCTTCCCAA
CGTAAATCTTTGAGAACTTTAAATGTAACAACTTCAATTGAATGGTTTCAAGCCCT
ACCCCCTAGTG
EQU_5
ATTATTAGACAAGAAAGGGGGAAAAAAGAAGCTTTTGGAACCTTAGGAATAATTT
ATGCTATAATAGCTATCGGATTATTAGGATTCGTAGTTTGAGCTCACCATATATTT
ACAGTAGGAATAGATGTAGATACCCGAGCTTACTTCACTTCAGCAACTATAATTA
TTGCGGTTCCAACTGGGATTAAAGTCTTTAGATGACTAGCCACCTTTCATGGAAC
TCAAATTTTAAATAGACCAGTAACCCTGTGAGCTTTAGGTTTTGTATTTTTATTTA
CAGTAGGAGGATTAACAGGGGTAATTTTAGCTAATTCTTCAATTGATATTATTCTC
CACGATACTTACTATGTAGTAGCTCATTTTCACTATGTTCTTTCTATAGGAGCTGT
ATTTGCAATTATAGCCGGAATTGTCCAATGATTCCCATTATTTACCGGACTAACC
TTAAATAATTTTTTTTTAAAAACCCAATTTATTACTATATTTTTAGGTGTAAATTTAA
CATTTTTTCCTCAACACTTTTTAGGGCTAAGAGGAATGCCTCGGCGATATTCGGA
TTATCCAGATGCTTTCACTATCTGAAATGCCATTTCATCTATTGGTTCCATTATTT
CTCTCACAAGAATTATCTTTTTTTTATTTATCTTATGAGAAAGGCTTTCTTCCCAA
CGTAAATCTTTGAGAACTTTAAATGTAACAACTTCAATTGAATGGTTTCAAGCCCT
ACCCCCTAGTG
;

endblock;

datasetS4

#NEXUS

begin data;
dimensions ntax=56 nchar=1579;
format datatype=dna gap=- interleave=no;
matrix

afr19
CGGGCGATGTGTACATATTTTAGAGCTTTAATCATCTTAAAAAAATCTAAAAAATT
ACTCTCAAATCCAAATTCAAATTTTTTTAACAAAAATTATTTTAAAATAAATTTATT
GTAACCCATTTCTTCTTTATTATAAACTACACCTTGATCTGATTTATTTTTTAATAA
AAAATTTTAAATATTTATAATTCTATTAAAATATTTAAACTACGACGATATATAAAC
TAATAAATAAAGTGCATCTAATCGTGGACTATCGATTATAGAACAGGTTCCTCTG
AAAAGACTAAAATACCGCCAAATTTTTTAATTTTCAAGAACATAACTAATACTAAT
TAAGTAAAAAAATTTACATTTTTAATAATAGGGTATCTAACAGACCTAATATTTCC
AGCTTCTGCACCAAAAATTAATTTTAATCCAACATCGAGGTCGCAATCTTTATCTT
CGATTAGAACTCTTTGATAATATTACGCTGTTATCCCTAAGGTAATTAAATCTTAC
AATTATTACTAATAGCTCAAGTATACATAAATTAATGATTTAAATTTAAAAAAAGTT
CAATTAATTTTTCAATCACCCCAATTAAATAATTAAAAAAATTAAAAATTTTTATAC
TaAAAAAAAATTAAAATTTTTAAATATGAAACTCTATAGGGTCTTCTCGTCTTTTAA
ATTAATTTTAGCTTTTTAACTAAAAAATAAATTTTTAATGTTTAAATAGAGACAGAA
ATTTTTTTATCCAACCATTCATTCCAGCTTCTAATTAAGAAACTAATGATTATGCTA
CCTTAGCACGGTCAAAATACCGCGGCCATTTAAATCCTCAGAGGGCAGGCTAAT
TATTAGACAAGAAAGGGGGAAAAAAGAAGCTTTTGGAACCTTAGGAATAATTTAT
GCCATGATAGCTATCGGATTATTAGGATTCGTAGTTTGAGCTCACCATATATTTA
CAGTAGGGATAGATGTAGATACCCGAGCTTACTTCACTTCAGCAACTATAATTAT
TGCGGTTCCAACTGGGATTAAAGTCTTTAGATGACTAGCCACCTTTCATGGAACT
CAAATTTTAAATAGACCAGTAACCCTGTGAGCTTTAGGTTTTGTATTTTTATTTAC
AGTAGGAGGATTAACAGGGGTAATTTTAGCTAATTCTTCAATTGATATTATTCTTC
ACGATACTTACTATGTAGTAGCTCATTTTCACTATGTTCTTTCTATAGGAGCTGTA
TTTGCAATTATAGCCGGAATTGTCCAATGATTCCCATTATTTACCGGACTAACCT
TAAATAATTTTTTTTTAAAAACCCAATTTATTACTATATTTTTAGGTGTAAATTTAAC
ATTTTTTCCCCAACACTTTTTAGGGCTAAGAGGAATGCCTCGGCGATATTCGGAT
TATCCAGATGCTTTCACTATCTGAAATGCCATTTCATCTATTGGTTCCATTATTTC
TCTCACAAGAATTATCTTTTTTTTTATTTATCTTATGAGAAAGGCTTTCTTCCCAAC
GTAAATCTTTGAGAACTTTAAATGTAACAACTTCAATTGAATGGTTACAAGCCCTA
CCCCCTAGTG
afr9
CGGGCGATGTGTACATATTTTAGAGCTTTAATCATCTTAAAAAAATCTAAAAAATT
ACTCTCAAATCCAAATTCAAATTTTTTTAACAAAAATTATTTTAAAATAAATTTATT
GTAACCCATTTCTTCTTTATTATAAACTACACCTTGATCTGATTTATTTTTTAATAA
AAAATTTTAAATATTTATAATTCTATTAAAATATTTAAACTACGACGATATATAAAC
TAATAAATAAAGTGCATCTAATCGTGGACTATCGATTATAGAACAGGTTCCTCTG
AAAAGACTAAAATACCGCCAAATTTTTTAATTTTCAAGAACATAACTAATACTAAT
TAAGTAAAAAAATTTACATTTTTAATAATAGGGTATCTAACAGACCTAATATTTCC
AGCTTCTGCACCAAAAATTAATTTTAATCCAACATCGAGGTCGCAATCTTTATCTT
CGATTAGAACTCTTTGATAATATTACGCTGTTATCCCTAAGGTAATTAAATCTTAC
AATTATTACTAATAGCTCAAGTATACATAAATTAATGATTTAAATTTAAAAAAAGTT
CGATTAATTTTTCAATCACCCCAATTAAATAATTAAAAAAATTAAAAATTTTTATAC

TaAAAAAAAATTAAAATTTTTAAATATGAAACTCTATAGGGTCTTCTCGTCTTTTAA
ATTAATCTTAGCTTTTTAACTAAAAAATAAATTTTTAATGTTTAAATAGAGACAGAA
ATTTTTTTATCCAACCATTCATTCCAGCTTCTAATTAAGAAACTAATGATTATGCTA
CCTTAGCACGGTCAAAATACCGCGGCCATTTAAATCCTCAGAGGGCAGGCTAAT
TATTAGACAAGAAAGGGGGAAAAAAGAAGCTTTTGGAACCTTAGGAATAATTTAT
GCCATGATAGCTATCGGATTATTAGGATTCGTAGTTTGAGCTCACCATATATTTA
CAGTAGGGATAGATGTAGATACCCGAGCTTACTTCACTTCAGCAACTATAATTAT
CGCGGTTCCAACTGGGATTAAAGTCTTTAGGTGACTAGCCACCTTTCATGGAAC
TCAAATTTTAAATAGACCAGTAACCCTGTGAGCTTTAGGTTTTGTATTTTTATTTA
CAGTAGGAGGATTAACAGGGGTAATTTTAGCTAATTCTTCAATTGATATTATTCTT
CACGATACTTACTATGTAGTAGCTCATTTTCACTATGTTCTTTCTATAGGAGCTGT
ATTTGCAATTATAGCCGGAATTGTCCAATGATTCCCATTATTTACTGGACTAACCT
TAAATAATTTTTTTTTAAAAACCCAATTTATTACTATATTTTTAGGTGTAAATTTAAC
ATTTTTTCCTCAACACTTTTTAGGGCTAAGAGGAATGCCTCGGCGATATTCGGAT
TATCCAGATGCTTTCACTATCTGAAATGCCATTTCATCTATTGGTTCCATTATTTC
TCTCACAAGAATTATCTTTTTTTTATTTATCTTATGAGAAAGGCTTTCTTCCCAAC
GTAAATCTTTGAGAACTTTAAATGTAACAACTTCAATTGAATGGTTACAAGCCCTA
CCCCCTAGTG
AOPE_12
CGGGCGATGTGTACATATTTTAGAGCTTTAATCATCTTAAAAAAATCTAAAAAATT
ACTCTCAAATCCAAATTCAAATTTTTTTAACAAAAATTATTTTCAAATAAATTTATT
GTAACCCATTTCTTCTTTATTATAAACTACACCTTGATCTGATTTATTTTTTAATAA
AAAATTTTAAATATTTATAATTCTATTAAAATATTTAAACTACGACGATATATAAAC
TAATAAATAAAGTGCATCTAATCGTGGACTATCGATTATAGAACAGGTTCCTCTG
AAAAGACTAAAATACCGCCAAATTTTTTAATTTTCAAGAACATAACTAATACTAAT
TAAGTAAAAAAATTTACATTTTTAATAATAGGGTATCTAACAGACCTAATATCCCC
AGCTTCTACACCAAAAATTAATTTTAATCCAACATCGAGGTCGCAATCTTTATCTT
CGATTAGAACTCTTTGATAATATTACGCTGTTATCCCTAAGGTAATTAAATCTTAC
AATTATTACTAATAGCTCAAGTATACATAAATTAATGATTTAAATTTAAAAAAAGTT
CAATTAATTTTTCAATCACCCCAATTAAATAATTAAAAAAATTAAAAATTTTTATAC
TaAAAAAAAATTAAAATTTTTAAATATGAAACTCTATAGGGTCTTCTCGTCTTTTAA
ATTAATTTTAGCTTTTTAACTAAAAAATAAATTTTTAATGTTTAAATAGAGACAGAA
ATTTTTTTATCCAACCATTCATTCCAGCTTCTAATTAAGAAACTAATGATTATGCTA
CCTTAGCACGGTCAAAATACCGCGGCCATTTAAATCCTCAGAGGGCAGGCTAAT
TATTAGACAAGAAAGGGGGAAAAAAGAAGCTTTTGGAACCTTAGGAATAATTTAT
GCTATAATAGCTATCGGATTATTAGGATTCGTAGTTTGAGCTCACCATATATTTAC
AGTAGGAATAGATGTAGATACCCGAGCTTACTTCACTTCAGCAACTATAATTATT
GCGGTTCCAACTGGGATTAAAGTCTTTAGATGACTAGCCACCTTTCATGGAACT
CAAATTTTAAATAGACCAGTAACCCTGTGAGCTTTAGGTTTTGTATTTTTATTTAC
AGTAGGAGGATTAACAGGGGTAATTTTAGCTAATTCTTCAATTGATATTATTCTCC
ACGATACTTACTATGTAGTAGCTCATTTTCACTATGTTCTTTCTATAGGAGCTGTA
TTTGCAATTATAGCCGGAATTGTCCAATGATTCCCATTATTTACCGGACTAACCT
TAAATAATTTTTTTTTAAAAACCCAATTTATTACTATATTTTTAGGTGTAAATTTAAC
ATTTTTTCCTCAACACTTTTTAGGGCTAAGAGGAATGCCTCGGCGATATTCGGAT
TATCCAGATGCTTTCACTATCTGAAATGCCATTTCATCTATTGGTTCCATTATTTC
TCTCACAAGAATTATCTTTTTTTTATTTATCTTATGAGAAAGGCTTTCTTCCCAAC
GTAAATCTTTGAGAACTTTAAATGTAACAACTTCAATTGAATGGTTTCAAGCCCTA
CCCCCTAGTG
AOPE_13
CGGGCGATGTGTACATATTTTAGAGCTTTAATCATCTTAAAAAAATCTAAAAAATT

ACTCTCAAATCCAAATTCAAATTTTTTTAACAAAAATTATTTTCAAATAAATTTATT
GTAACCCATTTCTTCTTTATTATAAACTACACCTTGATCTGATTTATTTTTTAATAA
AAAATTTTAAATATTTATAATTCTATTAAAATATTTAAACTACGACGATATATAAAC
TAATAAATAAAGTGCATCTAATCGTGGACTATCGATTATAGAACAGGTTCCTCTG
AAAAGACTAAAATACCGCCAAATTTTTTAATTTTCAAGAACATAACTAATACTAAT
TAAGTAAAAAAATTTACATTTTTAATAATAGGGTATCTAACAGACCTAATATCCCC
AGCTTCTACACCAAAAATTAATTTTAATCCAACATCGAGGTCGCAATCTTTATCTT
CGATTAGAACTCTTTGATAATATTACGCTGTTATCCCTAAGGTAATTAAATCTTAC
AATTATTACTAATAGCTCAAGTATACATAAATTAATGATTTAAATTTAAAAAAAGTT
CAATTAATTTTTCAATCACCCCAATTAAATAATTAAAAAAATTAAAAATTTTTATAC
TaAAAAAAAATTAAAATTTTTAAATATGAAACTCTATAGGGTCTTCTCGTCTTTTAA
ATTAATTTTAGCTTTTTAACTAAAAAATAAATTTTTAATGTTTAAATAGAGACAGAA
ATTTTTTTATCCAACCATTCATTCCAGCTTCTAATTAAGAAACTAATGATTATGCTA
CCTTAGCACGGTCAAAATACCGCGGCCATTTAAATCCTCAGAGGGCAGGCTAAT
TATTAGACAAGAAAGGGGGAAAAAAGAAGCTTTTGGAACCTTAGGAATAATTTAT
GCTATAATAGCTATCGGATTATTAGGATTCGTAGTTTGAGCTCACCATATATTTAC
AGTAGGAATAGATGTAGATACCCGAGCTTACTTCACTTCAGCAACTATAATTATT
GCGGTTCCAACTGGGATTAAAGTCTTTAGATGACTAGCCACCTTTCATGGAACT
CAAATTTTAAATAGACCAGTAACCCTGTGAGCTTTAGGTTTTGTATTTTTATTTAC
AGTAGGAGGATTAACAGGGGTAATTTTAGCTAATTCTTCAATTGATATTATTCTCC
ACGATACTTACTATGTAGTAGCTCATTTTCACTATGTTCTTTCTATAGGAGCTGTA
TTTGCAATTATAGCCGGAATTGTCCAATGATTCCCATTATTTACCGGACTAACCT
TAAATAATTTTTTTTTAAAAACCCAATTTATTACTATATTTTTAGGTGTAAATTTAAC
ATTTTTTCCTCAACACTTTTTAGGGCTAAGAGGAATGCCTCGGCGATATTCGGAT
TATCCAGATGCTTTCACTATCTGAAATGCCATTTCATCTATTGGTTCCATTATTTC
TCTCACAAGAATTATCTTTTTTTTTATTTATCTTATGAGAAAGGCTTTCTTCCCAAC
GTAAATCTTTGAGAACTTTAAATGTAACAACTTCAATTGAATGGTTTCAAGCCCTA
CCCCCTAGTG
AOPE_14
CGGGCGATGTGTACATATTTTAGAGCTTTAATCATCTTAAAAAAATCTAAAAAATT
ACTCTCAAATCCAAATTCAAATTTTTTTAACAAAAATTATTTTCAAATAAATTTATT
GTAACCCATTTCTTCTTTATTATAAACTACACCTTGATCTGATTTATTTTTTAATAA
AAAATTTTAAATATTTATAATTCTATTAAAATATTTAAACTACGACGATATATAAAC
TAATAAATAAAGTGCATCTAATCGTGGACTATCGATTATAGAACAGGTTCCTCTG
AAAAGACTAAAATACCGCCAAATTTTTTAATTTTCAAGAACATAACTAATACTAAT
TAAGTAAAAAAATTTACATTTTTAATAATAGGGTATCTAACAGACCTAATATCCCC
AGCTTCTACACCAAAAATTAATTTTAATCCAACATCGAGGTCGCAATCTTTATCTT
CGATTAGAACTCTTTGATAATATTACGCTGTTATCCCTAAGGTAATTAAATCTTAC
AATTATTACTAATAGCTCAAGTATACATAAATTAATGATTTAAATTTAAAAAAAGTT
CAATTAATTTTTCAATCACCCCAATTAAATAATTAAAAAAATTAAAAATTTTTATAC
TaAAAAAAAATTAAAATTTTTAAATATGAAACTCTATAGGGTCTTCTCGTCTTTTAA
ATTAATTTTAGCTTTTTAACTAAAAAATAAATTTTTAATGTTTAAATAGAGACAGAA
ATTTTTTTATCCAACCATTCATTCCAGCTTCTAATTAAGAAACTAATGATTATGCTA
CCTTAGCACGGTCAAAATACCGCGGCCATTTAAATCCTCAGAGGGCAGGCTAAT
TATTAGACAAGAAAGGGGGAAAAAAGAAGCTTTTGGAACCTTAGGAATAATTTAT
GCTATAATAGCTATCGGATTATTAGGATTCGTAGTTTGAGCTCACCATATATTTAC
AGTAGGAATAGATGTAGATACCCGAGCTTACTTCACTTCAGCAACTATAATTATT
GCGGTTCCAACTGGGATTAAAGTCTTTAGATGACTAGCCACCTTTCATGGAACT
CAAATTTTAAATAGACCAGTAACCCTGTGAGCTTTAGGTTTTGTATTTTTATTTAC
AGTAGGAGGATTAACAGGGGTAATTTTAGCTAATTCTTCAATTGATATTATTCTCC

ACGATACTTACTATGTAGTAGCTCATTTTCACTATGTTCTTTCTATAGGAGCTGTA
TTTGCAATTATAGCCGGAATTGTCCAATGATTCCCATTATTTACCGGACTAACCT
TAAATAATTTTTTTTTAAAAACCCAATTTATTACTATATTTTTAGGTGTAAATTTAAC
ATTTTTTCCTCAACACTTTTTAGGGCTAAGAGGAATGCCTCGGCGATATTCGGAT
TATCCAGATGCTTTCACTATCTGAAATGCCATTTCATCTATTGGTTCCATTATTTC
TCTCACAAGAATTATCTTTTTTTTTATTTATCTTATGAGAAAGGCTTTCTTCCCAAC
GTAAATCTTTGAGAACTTTAAATGTAACAACTTCAATTGAATGGTTTCAAGCCCTA
CCCCCTAGTG
AOPE_4
CGGGCGATGTGTACATATTTTAGAGCTTTAATCATCTTAAAAAAATCTAAAAAATT
ACTCTCAAATCCAAATTCAAATTTTTTTAACAAAAATTATTTTCAAATAAATTTATT
GTAACCCATTTCTTCTTTATTATAAACTACACCTTGATCTGATTTATTTTTTAATAA
AAAATTTTAAATATTTATAATTCTATTAAAATATTTAAACTACGACGATATATAAAC
TAATAAATAAAGTGCATCTAATCGTGGACTATCGATTATAGAACAGGTTCCTCTG
AAAAGACTAAAATACCGCCAAATTTTTTAATTTTCAAGAACATAACTAATACTAAT
TAAGTAAAAAAATTTACATTTTTAATAATAGGGTATCTAACAGACCTAATATCCCC
AGCTTCTACACCAAAAATTAATTTTAATCCAACATCGAGGTCGCAATCTTTATCTT
CGATTAGAACTCTTTGATAATATTACGCTGTTATCCCTAAGGTAATTAAATCTTAC
AATTATTACTAATAGCTCAAGTATACATAAATTAATGATTTAAATTTAAAAAAAGTT
CAATTAATTTTTCAATCACCCCAATTAAATAATTAAAAAAATTAAAAATTTTTATAC
TaAAAAAAAATTAAAATTTTTAAATATGAAACTCTATAGGGTCTTCTCGTCTTTTAA
ATTAATTTTAGCTTTTTAACTAAAAAATAAATTTTTAATGTTTAAATAGAGACAGAA
ATTTTTTTATCCAACCATTCATTCCAGCTTCTAATTAAGAAACTAATGATTATGCTA
CCTTAGCACGGTCAAAATACCGCGGCCATTTAAATCCTCAGAGGGCAGGCTAAT
TATTAGACAAGAAAGGGGGAAAAAAGAAGCTTTTGGAACCTTAGGAATAATTTAT
GCTATAATAGCTATCGGATTATTAGGATTCGTAGTTTGAGCTCACCATATATTTAC
AGTAGGAATAGATGTAGATACCCGAGCTTACTTCACTTCAGCAACTATAATTATT
GCGGTTCCAACTGGGATTAAAGTCTTTAGATGACTAGCCACCTTTCATGGAACT
CAAATTTTAAATAGACCAGTAACCCTGTGAGCTTTAGGTTTTGTATTTTTATTTAC
AGTAGGAGGATTAACAGGGGTAATTTTAGCTAATTCTTCAATTGATATTATTCTCC
ACGATACTTACTATGTAGTAGCTCATTTTCACTATGTTCTTTCTATAGGAGCTGTA
TTTGCAATTATAGCCGGAATTGTCCAATGATTCCCATTATTTACCGGACTAACCT
TAAATAATTTTTTTTTAAAAACCCAATTTATTACTATATTTTTAGGTGTAAATTTAAC
ATTTTTTCCTCAACACTTTTTAGGGCTAAGAGGAATGCCTCGGCGATATTCGGAT
TATCCAGATGCTTTCACTATCTGAAATGCCATTTCATCTATTGGTTCCATTATTTC
TCTCACAAGAATTATCTTTTTTTTTATTTATCTTATGAGAAAGGCTTTCTTCCCAAC
GTAAATCTTTGAGAACTTTAAATGTAACAACTTCAATTGAATGGTTTCAAGCCCTA
CCCCCTAGTG
bja1
CGGGCGATGTGTACATATTTTAGAGCTTTAATCATCTTAAAAAAATCTAAAAAATT
ACTCTCAAATCCAAATTCAAATTTTTTTAACAAAAATTATTTTAAAATAAATTTATT
GTAACCCATTTCTTCTTTATTATAAACTACACCTTGATCTGATTTATTTTTTAATAA
AAAATTTTAAATATTTATAATTCTATTAAAATATTTAAACTACGACGATATATAAAC
TAATAAATAAAGTGCATCTAATCGTGGACTATCGATTATAGAACAGGTTCCTCTG
AAAAGACTAAAATACCGCCAAATTTTTTAATTTTCAAGAACATAACTAATACTAAT
TAAGTAAAAAAATTTACATTTTTAATAATAGGGTATCTAACAGACCTAATATTTCC
AGCTTCTGCACCAAAAATTAATTTTAATCCAACATCGAGGTCGCAATCTTTATCTT
CGATTAGAACTCTTTGATAATATTACGCTGTTATCCCTAAGGTAATTAAATCTTAC
AATTATTACTAATAGCTCAAGTATACATAAATTAATGATTTAAATTTAAAAAAAGTT
CAATTAATTTTTCAATCACCCCAATTAAATAATTAAAAAAATTAAAAATTTTTATAC

TaAAAAAAAATTAAAATTTTTAAATATGAAACTCTATAGGGTCTTCTCGTCTTTTAA
ATTAATTTTAGCTTTTTAACTAAAAAATAAATTTTTAATGTTTAAATAGAGACAGAA
ATTTTTTTATCCAACCATTCATTCCAGCTTCTAATTAAGAAACTAATGATTATGCTA
CCTTAGCACGGTCAAAATACCGCGGCCATTTAAATCCTCAGAGGGCAGGCTAAT
TATTAGACAAGAAAGGGGGAAAAAAGAAGCTTTTGGAACCTTAGGAATAATTTAT
GCCATGATAGCTATCGGATTATTAGGATTCGTAGTTTGAGCTCACCATATATTTA
CAGTAGGGATAGATGTAGATACCCGAGCTTACTTCACTTCAGCAACTATAATTAT
TGCGGTTCCAACTGGGATTAAAGTCTTTAGATGACTAGCCACCTTTCATGGAACT
CAAATTTTAAATAGACCAGTAACCCTGTGAGCTTTAGGTTTTGTATTTTTATTTAC
AGTAGGAGGATTAACAGGGGTAATTTTAGCTAATTCTTCAATTGATATTATTCTTC
ACGATACTTACTATGTAGTAGCTCATTTTCACTATGTTCTTTCTATAGGAGCTGTA
TTTGCAATTATAGCCGGAATTGTCCAATGATTCCCATTATTTACCGGACTAACCT
TAAATAATTTTTTTTTAAAAACCCAATTTATTACTATATTTTTAGGTGTAAATTTAAC
ATTTTTTCCCCAACACTTTTTAGGGCTAAGAGGAATGCCTCGGCGATATTCGGAT
TATCCAGATGCTTTCACTATCTGAAATGCCATTTCATCTATTGGTTCCATTATTTC
TCTCACAAGAATTATCTTTTTTTTTATTTATCTTATGAGAAAGGCTTTCTTCCCAAC
GTAAATCTTTGAGAACTTTAAATGTAACAACTTCAATTGAATGGTTACAAGCCCTA
CCCCCTAGTG
CAM_YAH
CGGGCGATGTGTACATATTTTAGAGCTTTAATCATCTTAAAAAAATCTAAAAAATT
ACTCTCAAATCCAAATTCAAATTTTTTTAACAAAAATTATTTTAAAATAAATTTATT
GTAACCCATTTCTTCTTTATTATAAACTACACCTTGATCTGATTTATTTTTTAATAA
AAAATTTTAAATATTTATAATTCTATTAAAATATTTAAACTACGACGATATATAAAC
TAATAAATAAAGTGCATCTAATCGTGGACTATCGATTATAGAACAGGTTCCTCTG
AAAAGACTAAAATACCGCCAAATTTTTTAATTTTCAAGAACATAACTAATACTAAT
TAAGTAAAAAAATTTACATTTTTAATAATAGGGTATCTAACAGACCTAATATTTCC
AGCTTCTGCACCAAAAATTAATTTTAATCCAACATCGAGGTCGCAATCTTTATCTT
CGATTAGAACTCTTTGATAATATTACGCTGTTATCCCTAAGGTAATTAAATCTTAC
AATTATTACTAATAGCTCAAGTATACATAAATTAATGATTTAAATTTAAAAAAAGTT
CGATTAATTTTTCAATCACCCCAATTAAATAATTAAAAAAATTAAAAATTTTTATAC
TaAAAAAAAATTAAAATTTTTAAATATGAAACTCTATAGGGTCTTCTCGTCTTTTAA
ATTAATCTTAGCTTTTTAACTAAAAAATAAATTTTTAATGTTTAAATAGAGACAGAA
ATTTTTTTATCCAACCATTCATTCCAGCTTCTAATTAAGAAACTAATGATTATGCTA
CCTTAGCACGGTCAAAATACCGCGGCCATTTAAATCCTCAGAGGGCAGGCTAAT
TATTAGACAAGAAAGGGGGAAAAAAGAAGCTTTTGGAACCTTAGGAATAATTTAT
GCCATGATAGCTATCGGATTATTAGGATTCGTAGTTTGAGCTCTCCATATATTTA
CAGTAGGGATAGATGTAGATACCCGAGCTTACTTCACTTCAGCAACTATGATTAT
CGCGGTTCCAACTGGGATTAAAGTCTTTAGGTGACTAGCCACCTTTCATGGAAC
TCAAATTTTAAATAGACCAGTAACCCTGTGAGCTTTAGGTTTTGTATTTTTATTTA
CAGTAGGAGGATTAACAGGGGTAATTTTAGCTAATTCTTCAATTGATATTATTCTT
CACGATACTTACTATGTAGTAGCTCATTTTCACTATGTTCTTTCTATAGGAGCTGT
ATTTGCAATTATAGCCGGAATTGTCCAATGATTCCCATTATTTACTGGACTAACCT
TAAATAATTTTTTTTTAAAAACCCAATTTATTACTATATTTTTAGGTGTAAATTTAAC
ATTTTTTCCTCAACACTTTTTAGGGCTAAGAGGAATGCCTCGGCGATATTCGGAT
TATCCAGATGCTTTCACTATCTGAAATGCCATTTCATCTATTGGTTCCATTATTTC
TCTCACAAGAATTATCTTTTTTTTTATTTATCTTATGAGAAAGGCTTTCTTCCCAAC
GTAAATCTTTGAGAACTTTAAATGTAACAACTTCAATTGAATGGTTACAAGCCCTA
CCCCCTAGTG
CL1_1
CGGGCGATGTGTACATATTTTAGAGCTTTAATCATCTTAAAAAAATCTAAAAAATT

ACTCTCAAATCCAAATTCAAATTTTTTTAACAAAAATTATTTTCAAATAAATTTATT
GTAACCCATTTCTTCTTTATTATAAACTACACCTTGATCTGATTTATTTTTTAATAA
AAAATTTTAAATATTTATAATTCTATTAAAATATTTAAACTACGACGATATATAAAC
TAATAAATAAAGTGCATCTAATCGTGGACTATCGATTATAGAACAGGTTCCTCTG
AAAAGACTAAAATACCGCCAAATTTTTTAATTTTCAAGAACATAACTAATACTAAT
TAAGTAAAAAAATTTACATTTTTAATAATAGGGTATCTAACAGACCTAATATCCCC
AGCTTCTACACCAAAAATTAATTTTAATCCAACATCGAGGTCGCAATCTTTATCTT
CGATTAGAACTCTTTGATAATATTACGCTGTTATCCCTAAGGTAATTAAATCTTAC
AATTATTACTAATAGCTCAAGTATACATAAATTAATGATTTAAATTTAAAAAAAGTT
CAATTAATTTTTCAATCACCCCAATTAAATAATTAAAAAAATTAAAAATTTTTATAC
TAAAAAAAAAATTAAAATTTTTAAATATGAAACTCTATAGGGTCTTCTCGTCTTTTAA
ATTAATTTTAGCTTTTTAACTAAAAAATAAATTTTTAATGTTTAAATAGAGACAGAA
ATTTTTTTATCCAACCATTCATTCCAGCTTCTAATTAAGAAACTAATGATTATGCTA
CCTTAGCACGGTCAAAATACCGCGGCCATTTAAATCCTCAGAGGGCAGGCTAAT
TATTAGACAAGAAAGGGGGAAAAAAGAAGCTTTTGGAACCTTAGGAATAATTTAT
GCTATAATAGCTATCGGATTATTAGGATTCGTAGTTTGAGCTCACCATATATTTAC
AGTAGGAATAGATGTAGATACCCGAGCTTACTTCACTTCAGCAACTATAATTATT
GCGGTTCCAACTGGGATTAAAGTCTTTAGATGACTAGCCACCTTTCATGGAACT
CAAATTTTAAATAGACCAGTAACCCTGTGAGCTTTAGGTTTTGTATTTTTATTTAC
AGTAGGAGGATTAACAGGGGTAATTTTAGCTAATTCTTCAATTGATATTATTCTCC
ACGATACTTACTATGTAGTAGCTCATTTTCACTATGTTCTTTCTATAGGAGCTGTA
TTTGCAATTATAGCCGGAATTGTCCAATGATTCCCATTATTTACCGGACTAACCT
TAAATAATTTTTTTTTAAAAACCCAATTTATTACTATATTTTTAGGTGTAAATTTAAC
ATTTTTTCCTCAACACTTTTTAGGGCTAAGAGGAATGCCTCGGCGATATTCGGAT
TATCCAGATGCTTTCACTATCTGAAATGCCATTTCATCTATTGGTTCCATTATTTC
TCTCACAAGAATTATCTTTTTTTTTATTTATCTTATGAGAAAGGCTTTCTTCCCAAC
GTAAATCTTTGAGAACTTTAAATGTAACAACTTCAATTGAATGGTTTCAAGCCCTA
CCCCCTAGTG
CL1_2
CGGGCGATGTGTACATATTTTAGAGCTTTAATCATCTTAAAAAAATCTAAAAAATT
ACTCTCAAATCCAAATTCAAATTTTTTTAACAAAAATTATTTTCAAATAAATTTATT
GTAACCCATTTCTTCTTTATTATAAACTACACCTTGATCTGATTTATTTTTTAATAA
AAAATTTTAAATATTTATAATTCTATTAAAATATTTAAACTACGACGATATATAAAC
TAATAAATAAAGTGCATCTAATCGTGGACTATCGATTATAGAACAGGTTCCTCTG
AAAAGACTAAAATACCGCCAAATTTTTTAATTTTCAAGAACATAACTAATACTAAT
TAAGTAAAAAAATTTACATTTTTAATAATAGGGTATCTAACAGACCTAATATCCCC
AGCTTCTACACCAAAAATTAATTTTAATCCAACATCGAGGTCGCAATCTTTATCTT
CGATTAGAACTCTTTGATAATATTACGCTGTTATCCCTAAGGTAATTAAATCTTAC
AATTATTACTAATAGCTCAAGTATACATAAATTAATGATTTAAATTTAAAAAAAGTT
CAATTAATTTTTCAATCACCCCAATTAAATAATTAAAAAAATTAAAAATTTTTATAC
TAAAAAAAAAATTAAAATTTTTAAATATGAAACTCTATAGGGTCTTCTCGTCTTTTAA
ATTAATTTTAGCTTTTTAACTAAAAAATAAATTTTTAATGTTTAAATAGAGACAGAA
ATTTTTTTATCCAACCATTCATTCCAGCTTCTAATTAAGAAACTAATGATTATGCTA
CCTTAGCACGGTCAAAATACCGCGGCCATTTAAATCCTCAGAGGGCAGGCTAAT
TATTAGACAAGAAAGGGGGAAAAAAGAAGCTTTTGGAACCTTAGGAATAATTTAT
GCTATAATAGCTATCGGATTATTAGGATTCGTAGTTTGAGCTCACCATATATTTAC
AGTAGGAATAGATGTAGATACCCGAGCTTACTTCACTTCAGCAACTATAATTATT
GCGGTTCCAACTGGGATTAAAGTCTTTAGATGACTAGCCACCTTTCATGGAACT
CAAATTTTAAATAGACCAGTAACCCTGTGAGCTTTAGGTTTTGTATTTTTATTTAC
AGTAGGAGGATTAACAGGGGTAATTTTAGCTAATTCTTCAATTGATATTATTCTCC

ACGATACTTACTATGTAGTAGCTCATTTTCACTATGTTCTTTCTATAGGAGCTGTA
TTTGCAATTATAGCCGGAATTGTCCAATGATTCCCATTATTTACCGGACTAACCT
TAAATAATTTTTTTTTAAAAACCCAATTTATTACCATATTTTTAGGTGTAAATTTAA
CATTTTTTCCTCAACACTTTTTAGGGCTAAGAGGAATGCCTCGGCGATATTCGGA
TTATCCAGATGCTTTCACTATCTGAAATGCCATTTCATCTATTGGTTCCATTATTT
CTCTCACAAGAATTATCTTTTTTTTTATTTATCTTATGAGAAAGGCTTTCTTCCCAA
CGTAAATCTTTGAGAACTTTAAATGTAACAACTTCAATTGAATGGTTTCAAGCCCT
ACCCCCTAGTG
CL1_3
CGGGCGATGTGTACATATTTTAGAGCTTTAATCATCTTAAAAAAATCTAAAAAATT
ACTCTCAAATCCAAATTCAAATTTTTTTAACAAAAATTATTTTCAAATAAATTTATT
GTAACCCATTTCTTCTTTATTATAAACTACACCTTGATCTGATTTATTTTTTAATAA
AAAATTTTAAATATTTATAATTCTATTAAAATATTTAAACTACGACGATATATAAAC
TAATAAATAAAGTGCATCTAATCGTGGACTATCGATTATAGAACAGGTTCCTCTG
AAAAGACTAAAATACCGCCAAATTTTTTAATTTTCAAGAACATAACTAATACTAAT
TAAGTAAAAAAATTTACATTTTTAATAATAGGGTATCTAACAGACCTAATATCCCC
AGCTTCTACACCAAAAATTAATTTTAATCCAACATCGAGGTCGCAATCTTTATCTT
CGATTAGAACTCTTTGATAATATTACGCTGTTATCCCTAAGGTAATTAAATCTTAC
AATTATTACTAATAGCTCAAGTATACATAAATTAATGATTTAAATTTAAAAAAAGTT
CAATTAATTTTTCAATCACCCCAATTAAATAATTAAAAAAATTAAAAATTTTTATAC
TAAAAAAAAATTAAAATTTTTAAATATGAAACTCTATAGGGTCTTCTCGTCTTTTAA
ATTAATTTTAGCTTTTTAACTAAAAAATAAATTTTTAATGTTTAAATAGAGACAGAA
ATTTTTTTATCCAACCATTCATTCCAGCTTCTAATTAAGAAACTAATGATTATGCTA
CCTTAGCACGGTCAAAATACCGCGGCCATTTAAATCCTCAGAGGGCAGGCTAAT
TATTAGACAAGAAAGGGGGAAAAAAGAAGCTTTTGGAACCTTAGGAATAATTTAT
GCTATAATAGCTATCGGATTATTAGGATTCGTAGTTTGAGCTCACCATATATTTAC
AGTAGGAATAGATGTAGATACCCGAGCTTACTTCACTTCAGCAACTATAATTATT
GCGGTTCCAACTGGGATTAAAGTCTTTAGATGACTAGCCACCTTTCATGGAACT
CAAATTTTAAATAGACCAGTAACCCTGTGAGCTTTAGGTTTTGTATTTTTATTTAC
AGTAGGAGGATTAACAGGGGTAATTTTAGCTAATTCTTCAATTGATATTATTCTCC
ACGATACTTACTATGTAGTAGCTCATTTTCACTATGTTCTTTCTATAGGAGCTGTA
TTTGCAATTATAGCCGGAATTGTCCAATGATTCCCATTATTTACCGGACTAACCT
TAAATAATTTTTTTTTAAAAACCCAATTTATTACTATATTTTTAGGTGTAAATTTAAC
ATTTTTTCCTCAACACTTTTTAGGGCTAAGAGGAATGCCTCGGCGATATTCGGAT
TATCCAGATGCTTTCACTATCTGAAATGCCATTTCATCTATTGGTTCCATTATTTC
TCTCACAAGAATTATCTTTTTTTTTATTTATCTTATGAGAAAGGCTTTCTTCCCAAC
GTAAATCTTTGAGAACTTTAAATGTAACAACTTCAATTGAATGGTTTCAAGCCCTA
CCCCCTAGTG
CL1_4
CGGGCGATGTGTACATATTTTAGAGCTTTAATCATCTTAAAAAAATCTAAAAAATT
ACTCTCAAATCCAAATTCAAATTTTTTTAACAAAAATTATTTTCAAATAAATTTATT
GTAACCCATTTCTTCTTTATTATAAACTACACCTTGATCTGATTTATTTTTTAATAA
AAAATTTTAAATATTTATAATTCTATTAAAATATTTAAACTACGACGATATATAAAC
TAATAAATAAAGTGCATCTAATCGTGGACTATCGATTATAGAACAGGTTCCTCTG
AAAAGACTAAAATACCGCCAAATTTTTTAATTTTCAAGAACATAACTAATACTAAT
TAAGTAAAAAAATTTACATTTTTAATAATAGGGTATCTAACAGACCTAATATCCCC
AGCTTCTACACCAAAAATTAATTTTAATCCAACATCGAGGTCGCAATCTTTATCTT
CGATTAGAACTCTTTGATAATATTACGCTGTTATCCCTAAGGTAATTAAATCTTAC
AATTATTACTAATAGCTCAAGTATACATAAATTAATGATTTAAATTTAAAAAAAGTT
CAATTAATTTTTCAATCACCCCAATTAAATAATTAAAAAAATTAAAAATTTTTATAC

TAAAAAAAAAATTAAAATTTTTAAATATGAAACTCTATAGGGTCTTCTCGTCTTTTAA
ATTAATTTTAGCTTTTTAACTAAAAAATAAATTTTTAATGTTTAAATAGAGACAGAA
ATTTTTTTATCCAACCATTCATTCCAGCTTCTAATTAAGAAACTAATGATTATGCTA
CCTTAGCACGGTCAAAATACCGCGGCCATTTAAATCCTCAGAGGGCAGGCTAAT
TATTAGACAAGAAAGGGGGAAAAAAGAAGCTTTTGGAACCTTAGGAATAATTTAT
GCTATAATAGCTATCGGATTATTAGGATTCGTAGTTTGAGCTCACCATATATTTAC
AGTAGGAATAGATGTAGATACCCGAGCTTACTTCACTTCAGCAACTATAATTATT
GCGGTTCCAACTGGGATTAAAGTCTTTAGATGACTAGCCACCTTTCATGGAACT
CAAATTTTAAATAGACCAGTAACCCTGTGAGCTTTAGGTTTTGTATTTTTATTTAC
AGTAGGAGGATTAACAGGGGTAATTTTAGCTAATTCTTCAATTGATATTATTCTCC
ACGATACTTACTATGTAGTAGCTCATTTTCACTATGTTCTTTCTATAGGAGCTGTA
TTTGCAATTATAGCCGGAATTGTCCAATGATTCCCATTATTTACCGGACTAACCT
TAAATAATTTTTTTTTAAAAACCCAATTTATTACTATATTTTTAGGTGTAAATTTAAC
ATTTTTTCCTCAACACTTTTTAGGGCTAAGAGGAATGCCTCGGCGATATTCGGAT
TATCCAGATGCTTTCACTATCTGAAATGCCATTTCATCTATTGGTTCCATTATTTC
TCTCACAAGAATTATCTTTTTTTTATTTATCTTATGAGAAAGGCTTTCTTCCCAAC
GTAAATCTTTGAGAACTTTAAATGTAACAACTTCAATTGAATGGTTTCAAGCCCTA
CCCCCTAGTG
CL1_5
CGGGCGATGTGTACATATTTTAGAGCTTTAATCATCTTAAAAAAATCTAAAAAATT
ACTCTCAAATCCAAATTCAAATTTTTTTAACAAAAATTATTTTCAAATAAATTTATT
GTAACCCATTTCTTCTTTATTATAAACTACACCTTGATCTGATTTATTTTTTAATAA
AAAATTTTAAATATTTATAATTCTATTAAAATATTTAAACTACGACGATATATAAAC
TAATAAATAAAGTGCATCTAATCGTGGACTATCGATTATAGAACAGGTTCCTCTG
AAAAGACTAAAATACCGCCAAATTTTTTAATTTTCAAGAACATAACTAATACTAAT
TAAGTAAAAAAATTTACATTTTTAATAATAGGGTATCTAACAGACCTAATATCCCC
AGCTTCTACACCAAAAATTAATTTTAATCCAACATCGAGGTCGCAATCTTTATCTT
CGATTAGAACTCTTTGATAATATTACGCTGTTATCCCTAAGGTAATTAAATCTTAC
AATTATTACTAATAGCTCAAGTATACATAAATTAATGATTTAAATTTAAAAAAAGTT
CAATTAATTTTTCAATCACCCCAATTAAATAATTAAAAAAATTAAAAATTTTTATAC
TAAAAAAAAAATTAAAATTTTTAAATATGAAACTCTATAGGGTCTTCTCGTCTTTTAA
ATTAATTTTAGCTTTTTAACTAAAAAATAAATTTTTAATGTTTAAATAGAGACAGAA
ATTTTTTTATCCAACCATTCATTCCAGCTTCTAATTAAGAAACTAATGATTATGCTA
CCTTAGCACGGTCAAAATACCGCGGCCATTTAAATCCTCAGAGGGCAGGCTAAT
TATTAGACAAGAAAGGGGGAAAAAAGAAGCTTTTGGAACCTTAGGAATAATTTAT
GCTATAATAGCTATCGGATTATTAGGATTCGTAGTTTGAGCTCACCATATATTTAC
AGTAGGAATAGATGTAGATACCCGAGCTTACTTCACTTCAGCAACTATAATTATT
GCGGTTCCAACTGGGATTAAAGTCTTTAGATGACTAGCCACCTTTCATGGAACT
CAAATTTTAAATAGACCAGTAACCCTGTGAGCTTTAGGTTTTGTATTTTTATTTAC
AGTAGGAGGATTAACAGGGGTAATTTTAGCTAATTCTTCAATTGATATTATTCTCC
ACGATACTTACTATGTAGTAGCTCATTTTCACTATGTTCTTTCTATAGGAGCTGTA
TTTGCAATTATAGCCGGAATTGTCCAATGATTCCCATTATTTACCGGACTAACCT
TAAATAATTTTTTTTTAAAAACCCAATTTATTACCATATTTTTAGGTGTAAATTTAA
CATTTTTTCCTCAACACTTTTTAGGGCTAAGAGGAATGCCTCGGCGATATTCGGA
TTATCCAGATGCTTTCACTATCTGAAATGCCATTTCATCTATTGGTTCCATTATTT
CTCTCACAAGAATTATCTTTTTTTTATTTATCTTATGAGAAAGGCTTTCTTCCCAA
CGTAAATCTTTGAGAACTTTAAATGTAACAACTTCAATTGAATGGTTTCAAGCCCT
ACCCCCTAGTG
CL1_6
CGGGCGATGTGTACATATTTTAGAGCTTTAATCATCTTAAAAAAATCTAAAAAATT

ACTCTCAAATCCAAATTCAAATTTTTTTAACAAAAATTATTTTCAAATAAATTTATT
GTAACCCATTTCTTCTTTATTATAAACTACACCTTGATCTGATTTATTTTTTAATAA
AAAATTTTAAATATTTATAATTCTATTAAAATATTTAAACTACGACGATATATAAAC
TAATAAATAAAGTGCATCTAATCGTGGACTATCGATTATAGAACAGGTTCCTCTG
AAAAGACTAAAATACCGCCAAATTTTTTAATTTTCAAGAACATAACTAATACTAAT
TAAGTAAAAAAATTTACATTTTTAATAATAGGGTATCTAACAGACCTAATATCCCC
AGCTTCTACACCAAAAATTAATTTTAATCCAACATCGAGGTCGCAATCTTTATCTT
CGATTAGAACTCTTTGATAATATTACGCTGTTATCCCTAAGGTAATTAAATCTTAC
AATTATTACTAATAGCTCAAGTATACATAAATTAATGATTTAAATTTAAAAAAAGTT
CAATTAATTTTTCAATCACCCCAATTAAATAATTAAAAAAATTAAAAATTTTTATAC
TAAAAAAAAATTAAAATTTTTAAATATGAAACTCTATAGGGTCTTCTCGTCTTTTAA
ATTAATTTTAGCTTTTTAACTAAAAAATAAATTTTTAATGTTTAAATAGAGACAGAA
ATTTTTTTATCCAACCATTCATTCCAGCTTCTAATTAAGAAACTAATGATTATGCTA
CCTTAGCACGGTCAAAATACCGCGGCCATTTAAATCCTCAGAGGGCAGGCTAAT
TATTAGACAAGAAAGGGGGAAAAAAGAAGCTTTTGGAACCTTAGGAATAATTTAT
GCTATAATAGCTATCGGATTATTAGGATTCGTAGTTTGAGCTCACCATATATTTAC
AGTAGGAATAGATGTAGATACCCGAGCTTACTTCACTTCAGCAACTATAATTATT
GCGGTTCCAACTGGGATTAAAGTCTTTAGATGACTAGCCACCTTTCATGGAACT
CAAATTTTAAATAGACCAGTAACCCTGTGAGCTTTAGGTTTTGTATTTTTATTTAC
AGTAGGAGGATTAACAGGGGTAATTTTAGCTAATTCTTCAATTGATATTATTCTCC
ACGATACTTACTATGTAGTAGCTCATTTTCACTATGTTCTTTCTATAGGAGCTGTA
TTTGCAATTATAGCCGGAATTGTCCAATGATTCCCATTATTTACCGGACTAACCT
TAAATAATTTTTTTTTAAAAACCCAATTTATTACCATATTTTTAGGTGTAAATTTAA
CATTTTTTCCTCAACACTTTTTAGGGCTAAGAGGAATGCCTCGGCGATATTCGGA
TTATCCAGATGCTTTCACTATCTGAAATGCCATTTCATCTATTGGTTCCATTATTT
CTCTCACAAGAATTATCTTTTTTTTTATTTATCTTATGAGAAAGGCTTTCTTCCCAA
CGTAAATCTTTGAGAACTTTAAATGTAACAACTTCAATTGAATGGTTTCAAGCCCT
ACCCCCTAGTG
CL1_7
CGGGCGATGTGTACATATTTTAGAGCTTTAATCATCTTAAAAAAATCTAAAAAATT
ACTCTCAAATCCAAATTCAAATTTTTTTAACAAAAATTATTTTCAAATAAATTTATT
GTAACCCATTTCTTCTTTATTATAAACTACACCTTGATCTGATTTATTTTTTAATAA
AAAATTTTAAATATTTATAATTCTATTAAAATATTTAAACTACGACGATATATAAAC
TAATAAATAAAGTGCATCTAATCGTGGACTATCGATTATAGAACAGGTTCCTCTG
AAAAGACTAAAATACCGCCAAATTTTTTAATTTTCAAGAACATAACTAATACTAAT
TAAGTAAAAAAATTTACATTTTTAATAATAGGGTATCTAACAGACCTAATATCCCC
AGCTTCTACACCAAAAATTAATTTTAATCCAACATCGAGGTCGCAATCTTTATCTT
CGATTAGAACTCTTTGATAATATTACGCTGTTATCCCTAAGGTAATTAAATCTTAC
AATTATTACTAATAGCTCAAGTATACATAAATTAATGATTTAAATTTAAAAAAAGTT
CAATTAATTTTTCAATCACCCCAATTAAATAATTAAAAAAATTAAAAATTTTTATAC
TAAAAAAAAATTAAAATTTTTAAATATGAAACTCTATAGGGTCTTCTCGTCTTTTAA
ATTAATTTTAGCTTTTTAACTAAAAAATAAATTTTTAATGTTTAAATAGAGACAGAA
ATTTTTTTATCCAACCATTCATTCCAGCTTCTAATTAAGAAACTAATGATTATGCTA
CCTTAGCACGGTCAAAATACCGCGGCCATTTAAATCCTCAGAGGGCAGGCTAAT
TATTAGACAAGAAAGGGGGAAAAAAGAAGCTTTTGGAACCTTAGGAATAATTTAT
GCTATAATAGCTATCGGATTATTAGGATTCGTAGTTTGAGCTCACCATATATTTAC
AGTAGGAATAGATGTAGATACCCGAGCTTACTTCACTTCAGCAACTATAATTATT
GCGGTTCCAACTGGGATTAAAGTCTTTAGATGACTAGCCACCTTTCATGGAACT
CAAATTTTAAATAGACCAGTAACCCTGTGAGCTTTAGGTTTTGTATTTTTATTTAC
AGTAGGAGGATTAACAGGGGTAATTTTAGCTAATTCTTCAATTGATATTATTCTCC

ACGATACTTACTATGTAGTAGCTCATTTTCACTATGTTCTTTCTATAGGAGCTGTA
TTTGCAATTATAGCCGGAATTGTCCAATGATTCCCATTATTTACCGGACTAACCT
TAAATAATTTTTTTTTAAAAACCCAATTTATTACTATATTTTTAGGTGTAAATTTAAC
ATTTTTTCCTCAACACTTTTTAGGGCTAAGAGGAATGCCTCGGCGATATTCGGAT
TATCCAGATGCTTTCACTATCTGAAATGCCATTTCATCTATTGGTTCCATTATTTC
TCTCACAAGAATTATCTTTTTTTTATTTATCTTATGAGAAAGGCTTTCTTCCCAAC
GTAAATCTTTGAGAACTTTAAATGTAACAACTTCAATTGAATGGTTTCAAGCCCTA
CCCCCTAGTG
CL1_8
CGGGCGATGTGTACATATTTTAGAGCTTTAATCATCTTAAAAAAATCTAAAAAATT
ACTCTCAAATCCAAATTCAAATTTTTTTAACAAAAATTATTTTCAAATAAATTTATT
GTAACCCATTTCTTCTTTATTATAAACTACACCTTGATCTGATTTATTTTTTAATAA
AAAATTTTAAATATTTATAATTCTATTAAAATATTTAAACTACGACGATATATAAAC
TAATAAATAAAGTGCATCTAATCGTGGACTATCGATTATAGAACAGGTTCCTCTG
AAAAGACTAAAATACCGCCAAATTTTTTAATTTTCAAGAACATAACTAATACTAAT
TAAGTAAAAAAATTTACATTTTTAATAATAGGGTATCTAACAGACCTAATATCCCC
AGCTTCTACACCAAAAATTAATTTTAATCCAACATCGAGGTCGCAATCTTTATCTT
CGATTAGAACTCTTTGATAATATTACGCTGTTATCCCTAAGGTAATTAAATCTTAC
AATTATTACTAATAGCTCAAGTATACATAAATTAATGATTTAAATTTAAAAAAAGTT
CAATTAATTTTTCAATCACCCCAATTAAATAATTAAAAAAATTAAAAATTTTTATAC
TAAAAAAAAATTAAAATTTTTAAATATGAAACTCTATAGGGTCTTCTCGTCTTTTAA
ATTAATTTTAGCTTTTTAACTAAAAAATAAATTTTTAATGTTTAAATAGAGACAGAA
ATTTTTTTATCCAACCATTCATTCCAGCTTCTAATTAAGAAACTAATGATTATGCTA
CCTTAGCACGGTCAAAATACCGCGGCCATTTAAATCCTCAGAGGGCAGGCTAAT
TATTAGACAAGAAAGGGGGAAAAAAGAAGCTTTTGGAACCTTAGGAATAATTTAT
GCTATAATAGCTATCGGATTATTAGGATTCGTAGTTTGAGCTCACCATATATTTAC
AGTAGGAATAGATGTAGATACCCGAGCTTACTTCACTTCAGCAACTATAATTATT
GCGGTTCCAACTGGGATTAAAGTCTTTAGATGACTAGCCACCTTTCATGGAACT
CAAATTTTAAATAGACCAGTAACCCTGTGAGCTTTAGGTTTTGTATTTTTATTTAC
AGTAGGAGGATTAACAGGGGTAATTTTAGCTAATTCTTCAATTGATATTATTCTCC
ACGATACTTACTATGTAGTAGCTCATTTTCACTATGTTCTTTCTATAGGAGCTGTA
TTTGCAATTATAGCCGGAATTGTCCAATGATTCCCATTATTTACCGGACTAACCT
TAAATAATTTTTTTTTAAAAACCCAATTTATTACCATATTTTTAGGTGTAAATTTAA
CATTTTTTCCTCAACACTTTTTAGGGCTAAGAGGAATGCCTCGGCGATATTCGGA
TTATCCAGATGCTTTCACTATCTGAAATGCCATTTCATCTATTGGTTCCATTATTT
CTCTCACAAGAATTATCTTTTTTTTATTTATCTTATGAGAAAGGCTTTCTTCCCAA
CGTAAATCTTTGAGAACTTTAAATGTAACAACTTCAATTGAATGGTTTCAAGCCCT
ACCCCCTAGTG
CL1_9
CGGGCGATGTGTACATATTTTAGAGCTTTAATCATCTTAAAAAAATCTAAAAAATT
ACTCTCAAATCCAAATTCAAATTTTTTTAACAAAAATTATTTTCAAATAAATTTATT
GTAACCCATTTCTTCTTTATTATAAACTACACCTTGATCTGATTTATTTTTTAATAA
AAAATTTTAAATATTTATAATTCTATTAAAATATTTAAACTACGACGATATATAAAC
TAATAAATAAAGTGCATCTAATCGTGGACTATCGATTATAGAACAGGTTCCTCTG
AAAAGACTAAAATACCGCCAAATTTTTTAATTTTCAAGAACATAACTAATACTAAT
TAAGTAAAAAAATTTACATTTTTAATAATAGGGTATCTAACAGACCTAATATCCCC
AGCTTCTACACCAAAAATTAATTTTAATCCAACATCGAGGTCGCAATCTTTATCTT
CGATTAGAACTCTTTGATAATATTACGCTGTTATCCCTAAGGTAATTAAATCTTAC
AATTATTACTAATAGCTCAAGTATACATAAATTAATGATTTAAATTTAAAAAAAGTT
CAATTAATTTTTCAATCACCCCAATTAAATAATTAAAAAAATTAAAAATTTTTATAC

TAAAAAAAAAATTAAAATTTTTAAATATGAAACTCTATAGGGTCTTCTCGTCTTTTAA
ATTAATTTTAGCTTTTTAACTAAAAAATAAATTTTTAATGTTTAAATAGAGACAGAA
ATTTTTTTATCCAACCATTCATTCCAGCTTCTAATTAAGAAACTAATGATTATGCTA
CCTTAGCACGGTCAAAATACCGCGGCCATTTAAATCCTCAGAGGGCAGGCTAAT
TATTAGACAAGAAAGGGGGAAAAAAGAAGCTTTTGGAACCTTAGGAATAATTTAT
GCTATAATAGCTATCGGATTATTAGGATTCGTAGTTTGAGCTCACCATATATTTAC
AGTAGGAATAGATGTAGATACCCGAGCTTACTTCACTTCAGCAACTATAATTATT
GCGGTTCCAACTGGGATTAAAGTCTTTAGATGACTAGCCACCTTTCATGGAACT
CAAATTTTAAATAGACCAGTAACCCTGTGAGCTTTAGGTTTTGTATTTTTATTTAC
AGTAGGAGGATTAACAGGGGTAATTTTAGCTAATTCTTCAATTGATATTATTCTCC
ACGATACTTACTATGTAGTAGCTCATTTTCACTATGTTCTTTCTATAGGAGCTGTA
TTTGCAATTATAGCCGGAATTGTCCAATGATTCCCATTATTTACCGGACTAACCT
TAAATAATTTTTTTTTAAAAACCCAATTTATTACTATATTTTTAGGTGTAAATTTAAC
ATTTTTTCCTCAACACTTTTTAGGGCTAAGAGGAATGCCTCGGCGATATTCGGAT
TATCCAGATGCTTTCACTATCTGAAATGCCATTTCATCTATTGGTTCCATTATTTC
TCTCACAAGAATTATCTTTTTTTTATTTATCTTATGAGAAAGGCTTTCTTCCCAAC
GTAAATCTTTGAGAACTTTAAATGTAACAACTTCAATTGAATGGTTTCAAGCCCTA
CCCCCTAGTG
CL2_1
CGGGCGATGTGTACATATTTTAGAGCTTTAATCATCTTAAAAAAATCTAAAAAATT
ACTCTCAAATCCAAATTCAAATTTTTTTAACAAAAATTATTTTCAAATAAATTTATT
GTAACCCATTTCTTCTTTATTATAAACTACACCTTGATCTGATTTATTTTTTAATAA
AAAATTTTAAATATTTATAATTCTATTAAAATATTTAAACTACGACGATATATAAAC
TAATAAATAAAGTGCATCTAATCGTGGACTATCGATTATAGAACAGGTTCCTCTG
AAAAGACTAAAATACCGCCAAATTTTTTAATTTTCAAGAACATAACTAATACTAAT
TAAGTAAAAAAATTTACATTTTTAATAATAGGGTATCTAACAGACCTAATATCCCC
AGCTTCTACACCAAAAATTAATTTTAATCCAACATCGAGGTCGCAATCTTTATCTT
CGATTAGAACTCTTTGATAATATTACGCTGTTATCCCTAAGGTAATTAAATCTTAC
AATTATTACTAATAGCTCAAGTATACATAAATTAATGATTTAAATTTAAAAAAAGTT
CAATTAATTTTTCAATCACCCCAATTAAATAATTAAAAAAATTAAAAATTTTTATAC
TAAAAAAAAAATTAAAATTTTTAAATATGAAACTCTATAGGGTCTTCTCGTCTTTTAA
ATTAATTTTAGCTTTTTAACTAAAAAATAAATTTTTAATGTTTAAATAGAGACAGAA
ATTTTTTTATCCAACCATTCATTCCAGCTTCTAATTAAGAAACTAATGATTATGCTA
CCTTAGCACGGTCAAAATACCGCGGCCATTTAAATCCTCAGAGGGCAGGCTAAT
TATTAGACAAGAAAGGGGGAAAAAAGAAGCTTTTGGAACCTTAGGAATAATTTAT
GCTATAATAGCTATCGGATTATTAGGATTCGTAGTTTGAGCTCACCATATATTTAC
AGTAGGAATAGATGTAGATACCCGAGCTTACTTCACTTCAGCAACTATAATTATT
GCGGTTCCAACTGGGATTAAAGTCTTTAGATGACTAGCCACCTTTCATGGAACT
CAAATTTTAAATAGACCAGTAACCCTGTGAGCTTTAGGTTTTGTATTTTTATTTAC
AGTAGGAGGATTAACAGGGGTAATTTTAGCTAATTCTTCAATTGATATTATTCTCC
ACGATACTTACTATGTAGTAGCTCATTTTCACTATGTTCTTTCTATAGGAGCTGTA
TTTGCAATTATAGCCGGAATTGTCCAATGATTCCCATTATTTACCGGACTAACCT
TAAATAATTTTTTTTTAAAAACCCAATTTATTACTATATTTTTAGGTGTAAATTTAAC
ATTTTTTCCTCAACACTTTTTAGGGCTAAGAGGAATGCCTCGGCGATATTCGGAT
TATCCAGATGCTTTCACTATCTGAAATGCCATTTCATCTATTGGTTCCATTATTTC
TCTCACAAGAATTATCTTTTTTTTATTTATCTTATGAGAAAGGCTTTCTTCCCAAC
GTAAATCTTTGAGAACTTTAAATGTAACAACTTCAATTGAATGGTTTCAAGCCCTA
CCCCCTAGTG
CL2_2
CGGGCGATGTGTACATATTTTAGAGCTTTAATCATCTTAAAAAAATCTAAAAAATT

ACTCTCAAATCCAAATTCAAATTTTTTTAACAAAAATTATTTTCAAATAAATTTATT
GTAACCCATTTCTTCTTTATTATAAACTACACCTTGATCTGATTTATTTTTTAATAA
AAAATTTTAAATATTTATAATTCTATTAAAATATTTAAACTACGACGATATATAAAC
TAATAAATAAAGTGCATCTAATCGTGGACTATCGATTATAGAACAGGTTCCTCTG
AAAAGACTAAAATACCGCCAAATTTTTTAATTTTCAAGAACATAACTAATACTAAT
TAAGTAAAAAAATTTACATTTTTAATAATAGGGTATCTAACAGACCTAATATCCCC
AGCTTCTACACCAAAAATTAATTTTAATCCAACATCGAGGTCGCAATCTTTATCTT
CGATTAGAACTCTTTGATAATATTACGCTGTTATCCCTAAGGTAATTAAATCTTAC
AATTATTACTAATAGCTCAAGTATACATAAATTAATGATTTAAATTTAAAAAAAGTT
CAATTAATTTTTCAATCACCCCAATTAAATAATTAAAAAAATTAAAAATTTTTATAC
TAAAAAAAAATTAAAATTTTTAAATATGAAACTCTATAGGGTCTTCTCGTCTTTTAA
ATTAATTTTAGCTTTTTAACTAAAAAATAAATTTTTAATGTTTAAATAGAGACAGAA
ATTTTTTTATCCAACCATTCATTCCAGCTTCTAATTAAGAAACTAATGATTATGCTA
CCTTAGCACGGTCAAAATACCGCGGCCATTTAAATCCTCAGAGGGCAGGCTAAT
TATTAGACAAGAAAGGGGGAAAAAAGAAGCTTTTGGAACCTTAGGAATAATTTAT
GCTATAATAGCTATCGGATTATTAGGATTCGTAGTTTGAGCTCACCATATATTTAC
AGTAGGAATAGATGTAGATACCCGAGCTTACTTCACTTCAGCAACTATAATTATT
GCGGTTCCAACTGGGATTAAAGTCTTTAGATGACTAGCCACCTTTCATGGAACT
CAAATTTTAAATAGACCAGTAACCCTGTGAGCTTTAGGTTTTGTATTTTTATTTAC
AGTAGGAGGATTAACAGGGGTAATTTTAGCTAATTCTTCAATTGATATTATTCTCC
ACGATACTTACTATGTAGTAGCTCATTTTCACTATGTTCTTTCTATAGGAGCTGTA
TTTGCAATTATAGCCGGAATTGTCCAATGATTCCCATTATTTACCGGACTAACCT
TAAATAATTTTTTTTTAAAAACCCAATTTATTACTATATTTTTAGGTGTAAATTTAAC
ATTTTTTCCTCAACACTTTTTAGGGCTAAGAGGAATGCCTCGGCGATATTCGGAT
TATCCAGATGCTTTCACTATCTGAAATGCCATTTCATCTATTGGTTCCATTATTTC
TCTCACAAGAATTATCTTTTTTTTTATTTATCTTATGAGAAAGGCTTTCTTCCCAAC
GTAAATCTTTGAGAACTTTAAATGTAACAACTTCAATTGAATGGTTTCAAGCCCTA
CCCCCTAGTG
EQU_5
CGGGCGATGTGTACATATTTTAGAGCTTTAATCATCTTAAAAAAATCTAAAAAATT
ACTCTCAAATCCAAATTCAAATTTTTTTAACAAAAATTATTTTCAAATAAATTTATT
GTAACCCATTTCTTCTTTATTATAAACTACACCTTGATCTGATTTATTTTTTAATAA
AAAATTTTAAATATTTATAATTCTATTAAAATATTTAAACTACGACGATATATAAAC
TAATAAATAAAGTGCATCTAATCGTGGACTATCGATTATAGAACAGGTTCCTCTG
AAAAGACTAAAATACCGCCAAATTTTTTAATTTTCAAGAACATAACTAATACTAAT
TAAGTAAAAAAATTTACATTTTTAATAATAGGGTATCTAACAGACCTAATATCCCC
AGCTTCTACACCAAAAATTAATTTTAATCCAACATCGAGGTCGCAATCTTTATCTT
CGATTAGAACTCTTTGATAATATTACGCTGTTATCCCTAAGGTAATTAAATCTTAC
AATTATTACTAATAGCTCAAGTATACATAAATTAATGATTTAAATTTAAAAAAAGTT
CAATTAATTTTTCAATCACCCCAATTAAATAATTAAAAAAATTAAAAATTTTTATAC
TAAAAAAAAATTAAAATTTTTAAATATGAAACTCTATAGGGTCTTCTCGTCTTTTAA
ATTAATTTTAGCTTTTTAACTAAAAAATAAATTTTTAATGTTTAAATAGAGACAGAA
ATTTTTTTATCCAACCATTCATTCCAGCTTCTAATTAAGAAACTAATGATTATGCTA
CCTTAGCACGGTCAAAATACCGCGGCCATTTAAATCCTCAGAGGGCAGGCTAAT
TATTAGACAAGAAAGGGGGAAAAAAGAAGCTTTTGGAACCTTAGGAATAATTTAT
GCTATAATAGCTATCGGATTATTAGGATTCGTAGTTTGAGCTCACCATATATTTAC
AGTAGGAATAGATGTAGATACCCGAGCTTACTTCACTTCAGCAACTATAATTATT
GCGGTTCCAACTGGGATTAAAGTCTTTAGATGACTAGCCACCTTTCATGGAACT
CAAATTTTAAATAGACCAGTAACCCTGTGAGCTTTAGGTTTTGTATTTTTATTTAC
AGTAGGAGGATTAACAGGGGTAATTTTAGCTAATTCTTCAATTGATATTATTCTCC

ACGATACTTACTATGTAGTAGCTCATTTTCACTATGTTCTTTCTATAGGAGCTGTA
TTTGCAATTATAGCCGGAATTGTCCAATGATTCCCATTATTTACCGGACTAACCT
TAAATAATTTTTTTTTAAAAACCCAATTTATTACTATATTTTTAGGTGTAAATTTAAC
ATTTTTTCCTCAACACTTTTTAGGGCTAAGAGGAATGCCTCGGCGATATTCGGAT
TATCCAGATGCTTTCACTATCTGAAATGCCATTTCATCTATTGGTTCCATTATTTC
TCTCACAAGAATTATCTTTTTTTTTATTTATCTTATGAGAAAGGCTTTCTTCCCAAC
GTAAATCTTTGAGAACTTTAAATGTAACAACTTCAATTGAATGGTTTCAAGCCCTA
CCCCCTAGTG
FRA_CHA_5
CGGGCGATGTGTACATATTTTAGAGCTTTAATCATCTTAAAAAAATCTAAAAAATT
ACTCTCAAATCCAAATTCAAATTTTTTTAACAAAAATTATTTTAAAATAAATTTATT
GTAACCCATTTCTTCTTTATTATAAACTACACCTTGATCTGATTTATTTTTTAATAA
AAAATTTTAAATATTTATAATTCTATTAAAATATTTAAACTACGACGATATATAAAC
TAATAAATAAAGTGCATCTAATCGTGGACTATCGATTATAGAACAGGTTCCTCTG
AAAAGACTAAAATACCGCCAAATTTTTTAATTTTCAAGAACATAACTAATACTAAT
TAAGTAAAAAAATTTACATTTTTAATAATAGGGTATCTAACAGACCTAATATTTCC
AGCTTCTGCACCAAAAATTAATTTTAATCCAACATCGAGGTCGCAATCTTTATCTT
CGATTAGAACTCTTTGATAATATTACGCTGTTATCCCTAAGGTAATTAAATCTTAC
AATTATTACTAATAGCTCAAGTATACATAAATTAATGATTTAAATTTAAAAAAAGTT
CGATTAATTTTTCAATCACCCCAATTAAATAATTAAAAAAATTAAAAATTTTTATAC
TaAAAAAAAATTAAAATTTTTAAATATGAAACTCTATAGGGTCTTCTCGTCTTTTAA
ATTAATCTTAGCTTTTTAACTAAAAAATAAATTTTTAATGTTTAAATAGAGACAGAA
ATTTTTTTATCCAACCATTCATTCCAGCTTCTAATTAAGAAACTAATGATTATGCTA
CCTTAGCACGGTCAAAATACCGCGGCCATTTAAATCCTCAGAGGGCAGGCTAAT
TATTAGACAAGAAAGGGGGAAAAAAGAAGCTTTTGGAACCTTAGGAATAATTTAT
GCCATGATAGCTATCGGATTATTAGGATTCGTAGTTTGAGCTCACCATATATTTA
CAGTAGGGATAGATGTAGATACCCGAGCTTACTTCACTTCAGCAACTATAATTAT
TGCGGTTCCAACTGGGATTAAAGTCTTTAGATGACTAGCCACCTTTCATGGAACT
CAAATTTTAAATAGACCAGTAACCCTGTGAGCTTTAGGCTTTGTATTTTTATTTAC
AGTAGGAGGATTAACAGGGGTAATTTTAGCTAATTCTTCAATTGATATTATTCTTC
ACGATACTTACTATGTAGTAGCTCATTTTCACTATGTTCTTTCTATAGGAGCTGTA
TTTGCAATTATAGCCGGAATTGTCCAATGATTTCCATTATTTACTGGACTAACCTT
AAATAATTTTTTTTTAAAAACCCAATTTATTACTATATTTTTAGGTGTAAATTTAACA
TTTTTTCCTCAACACTTTTTAGGGCTAAGAGGAATGCCTCGGCGATATTCGGATT
ATCCAGATGCTTTCACTATCTGAAATGCCATTTCATCTATTGGTTCCATTATTTCT
CTCACAAGAATTATCTTTTTTTTTATTTATCTTATGAGAAAGGCTTTCTTCCCAACG
TAAATCTTTGAGAACTTTAAATGTAACAACTTCAATTGAATGGTTACAAGCCCTAC
CCCCTAGTG
hev1
CGGGCGATGTGTACATATTTTAGAGCTTTAATCATCTTAAAAAAATCTAAAAAATT
ACTCTCAAATCCAAATTCAAATTTTTTTAACAAAAATTATTTTAAAATAAATTTATT
GTAACCCATTTCTTCTTTATTATAAACTACACCTTGATCTGATTTATTTTTTAATAA
AAAATTTTAAATATTTATAATTCTATTAAAATATTTAAACTACGACGATATATAAAC
TAATAAATAAAGTGCATCTAATCGTGGACTATCGATTATAGAACAGGTTCCTCTG
AAAAGACTAAAATACCGCCAAATTTTTTAATTTTCAAGAACATAACTAATACTAAT
TAAGTAAAAAAATTTACATTTTTAATAATAGGGTATCTAACAGACCTAATATTTCC
AGCTTCTGCACCAAAAATTAATTTTAATCCAACATCGAGGTCGCAATCTTTATCTT
CGATTAGAACTCTTTGATAATATTACGCTGTTATCCCTAAGGTAATTAAATCTTAC
AATTATTACTAATAGCTCAAGTATACATAAATTAATGATTTAAATTTAAAAAAAGTT
CAATTAATTTTTCAATCACCCCAATTAAATAATTAAAAAAATTAAAAATTTTTATAC

TaAAAAAAAATTAAAATTTTTAAATATGAAACTCTATAGGGTCTTCTCGTCTTTTAA
ATTAATTTTAGCTTTTTAACTAAAAAATAAATTTTTAATGTTTAAATAGAGACAGAA
ATTTTTTTATCCAACCATTCATTCCAGCTTCTAATTAAGAAACTAATGATTATGCTA
CCTTAGCACGGTCAAAATACCGCGGCCATTTAAATCCTCAGAGGGCAGGCTAAT
TATTAGACAAGAAAGGGGGAAAAAAGAAGCTTTTGGAACCTTAGGAATAATTTAT
GCCATGATAGCTATCGGATTATTAGGATTCGTAGTTTGAGCTCACCATATATTTA
CAGTAGGGATAGATGTAGATACCCGAGCTTACTTCACTTCAGCAACTATAATTAT
TGCGGTTCCAACTGGGATTAAAGTCTTTAGATGACTAGCCACCTTTCATGGAACT
CAAATTTTAAATAGACCAGTAACCCTGTGAGCTTTAGGTTTTGTATTTTTATTTAC
AGTAGGAGGATTAACAGGGGTAATTTTAGCTAATTCTTCAATTGATATTATTCTTC
ACGATACTTACTATGTAGTAGCTCATTTTCACTATGTTCTTTCTATAGGAGCTGTA
TTTGCAATTATAGCCGGAATTGTCCAATGATTCCCATTATTTACCGGACTAACCT
TAAATAATTTTTTTTTAAAAACCCAATTTATTACTATATTTTTAGGTGTAAATTTAAC
ATTTTTTCCCCAACACTTTTTAGGGCTAAGAGGAATGCCTCGGCGATATTCGGAT
TATCCAGATGCTTTCACTATCTGAAATGCCATTTCATCTATTGGTTCCATTATTTC
TCTCACAAGAATTATCTTTTTTTTTATTTATCTTATGAGAAAGGCTTTCTTCCCAAC
GTAAATCTTTGAGAACTTTAAATGTAACAACTTCAATTGAATGGTTACAAGCCCTA
CCCCCTAGTG
MEX_COE_2
CGGGCGATGTGTACATATTTTAGAGCTTTAATCATCTTAAAAAAATCTAAAAAATT
ACTCTCAAATCCAAATTCAAATTTTTTTAACAAAAATTATTTTCAAATAAATTTATT
GTAACCCATTTCTTCTTTATTATAAACTACACCTTGATCTGATTTATTTTTTAATAA
AAAATTTTAAATATTTATAATTCTATTAAAATATTTAAACTACGACGATATATAAAC
TAATAAATAAAGTGCATCTAATCGTGGACTATCGATTATAGAACAGGTTCCTCTG
AAAAGACTAAAATACCGCCAAATTTTTTAATTTTCAAGAACATAACTAATACTAAT
TAAGTAAAAAAATTTACATTTTTAATAATAGGGTATCTAACAGACCTAATATTCCC
AGCTTCTGCACCAAAAATTAATTTTAATCCAACATCGAGGTCGCAATCTTTATCTT
CGATTAGAACTCTTTGATAATATTACGCTGTTATCCCTAAGGTAATTAAATCTTAC
AATTATTACTAATAGCTCAAGTATACATAAATTAATGATTTAAATTTAAAAAAAGTT
CAATTAATTTTTCAATCACCCCAATTAAATAATTAAAAAAATTAAAAATTTTTATAC
TaAAAAAAAATTAAAATTTTTAAATATGAAACTCTATAGGGTCTTCTCGTCTTTTAA
ATTAATTTTAGCTTTTTAACTAAAAAATAAATTTTTAATGTTTAAATAGAGACAGAA
ATTTTTTTATCCAACCATTCATTCCAGCTTCTAATTAAGAAACTAATGATTATGCTA
CCTTAGCACGGTCAAAATACCGCGGCCATTTAAATCCTCAGAGGGCAGGCTAAT
TATTAGACAAGAAAGGGGGAAAAAAGAACCTTTTGGAACCTTAGGAATAATTTAT
GCCATAATAGCTATCGGATTATTAGGATTCGTAGTTTGAGCTCACCATATATTTA
CAGTAGGGATAGATGTAGATACCCGAGCTTACTTCACTTCAGCAACTATAATTAT
TGCGGTTCCAACTGGGATTAAAGTCTTTAGATGACTAGCCACCTTTCATGGAACT
CAAATTTTAAATAGACCAGTAACCCTGTGAGCTTTAGGTTTTGTATTTTTATTTAC
AGTAGGAGGATTAACAGGGGTAATTTTAGCTAATTCTTCAATTGATATTATTCTTC
ACGATACTTACTATGTAGTAGCTCATTTTCACTATGTTCTTTCTATAGGAGCTGTA
TTTGCAATTATAGCCGGAATTGTCCAATGATTCCCATTATTTACTGGACTAACCTT
AAATAATTTTTTTTTAAAAACCCAATTTATTACTATATTTTTAGGTGTAAATTTAACA
TTTTTTCCTCAACACTTTTTAGGGCTAAGAGGAATGCCTCGGCGATATTCGGATT
ATCCAGATGCTTTCACTATCTGAAATGCCATTTCATCTATTGGTTCCATTATTTCT
CTCACAAGAATTATCTTTTTTTTTATTTATCTTATGAGAAAGGCTTTCTTCCCAACG
TAAATCTTTGAGAACTTTAAATGTAACAACTTCAATTGAATGGTTACAAGCCCTAC
CCCCTAGTG
MEX_SAG_5
CGGGCGATGTGTACATATTTTAGAGCTTTAATCATCTTAAAAAAATCTAAAAAATT

ACTCTCAAATCCAAATTCAAATTTTTTTAACAAAAATTATTTTCAAATAAATTTATT
GTAACCCATTTCTTCTTTATTATAAACTACACCTTGATCTGATTTATTTTTTAATAA
AAAATTTTAAATATTTATAATTCTATTAAAATATTTAAACTACGACGATATATAAAC
TAATAAATAAAGTGCATCTAATCGTGGACTATCGATTATAGAACAGGTTCCTCTG
AAAAGACTAAAATACCGCCAAATTTTTTAATTTTCAAGAACATAACTAATACTAAT
TAAGTAAAAAAATTTACATTTTTAATAATAGGGTATCTAACAGACCTAATATTCCC
AGCTTCTGCACCAAAAATTAATTTTAATCCAACATCGAGGTCGCAATCTTTATCTT
CGATTAGAACTCTTTGATAATATTACGCTGTTATCCCTAAGGTAATTAAATCTTAC
AATTATTACTAATAGCTCAAGTATACATAAATTAATGATTTAAATTTAAAAAAAGTT
CAATTAATTTTTCAATCACCCCAATTAAATAATTAAAAAAATTAAAAATTTTTATAC
TaAAAAAAAATTAAAATTTTTAAATATGAAACTCTATAGGGTCTTCTCGTCTTTTAA
ATTAATTTTAGCTTTTTAACTAAAAAATAAATTTTTAATGTTTAAATAGAGACAGAA
ATTTTTTTATCCAACCATTCATTCCAGCTTCTAATTAAGAAACTAATGATTATGCTA
CCTTAGCACGGTCAAAATACCGCGGCCATTTAAATCCTCAGAGGGCAGGCTAAT
TATTAGACAAGAAAGGGGGAAAAAAGAAGCTTTTGGAACCTTAGGAATAATTTAT
GCCATAATAGCTATCGGATTATTAGGATTCGTAGTTTGAGCTCACCATATATTTA
CAGTAGGGATAGATGTAGATACCCGAGCTTACTTCACTTCAGCAACTATAATTAT
TGCGGTTCCAACTGGGATTAAAGTCTTTAGATGACTAGCCACCTTTCATGGAACT
CAAATTTTAAATAGACCAGTAACCCTGTGAGCTTTAGGTTTTGTATTTTTATTTAC
AGTAGGAGGATTAACAGGGGTAATTTTAGCTAATTCTTCAATTGATATTATTCTTC
ACGATACTTACTATGTAGTAGCTCATTTTCACTATGTTCTTTCTATAGGAGCTGTA
TTTGCAATTATAGCCGGAATTGTCCAATGATTCCCATTATTTACTGGGCTAACCT
TAAATAATTTTTTTTTAAAAACCCAATTTATTACTATATTTTTAGGTGTAAATTTAAC
ATTTTTTCCTCAACACTTTTTAGGGCTAAGAGGAATGCCTCGGCGATATTCGGAT
TATCCAGATGCTTTCACTATCTGAAATGCCATTTCATCTATTGGTTCCATTATTTC
TCTCACAAGAATTATCTTTTTTTTTATTTATCTTATGAGAAAGGCTTTCTTCCCAAC
GTAAATCTTTGAGAACTTTAAATGTAACAACTTCAATTGAATGGTTACAAGCCCTA
CCCCCTAGTG
MEX_SCA
CGGGCGATGTGTACATATTTTAGAGCTTTAATCATCTTAAAAAAATCTAAAAAATT
ACTCTCAAATCCAAATTCAAATTTTTTTAACAAAAATTATTTTCAAATAAATTTATT
GTAACCCATTTCTTCTTTATTATAAACTACACCTTGATCTGATTTATTTTTTAATAA
AAAATTTTAAATATTTATAATTCTATTAAAATATTTAAACTACGACGATATATAAAC
TAATAAATAAAGTGCATCTAATCGTGGACTATCGATTATAGAACAGGTTCCTCTG
AAAAGACTAAAATACCGCCAAATTTTTTAATTTTCAAGAACATAACTAATACTAAT
TAAGTAAAAAAATTTACATTTTTAATAATAGGGTATCTAACAGACCTAATATTCCC
AGCTTCTGCACCAAAAATTAATTTTAATCCAACATCGAGGTCGCAATCTTTATCTT
CGATTAGAACTCTTTGATAATATTACGCTGTTATCCCTAAGGTAATTAAATCTTAC
AATTATTACTAATAGCTCAAGTATACATAAATTAATGATTTAAATTTAAAAAAAGTT
CAATTAATTTTTCAATCACCCCAATTAAATAATTAAAAAAATTAAAAATTTTTATAC
TaAAAAAAAATTAAAATTTTTAAATATGAAACTCTATAGGGTCTTCTCGTCTTTTAA
ATTAATTTTAGCTTTTTAACTAAAAAATAAATTTTTAATGTTTAAATAGAGACAGAA
ATTTTTTTATCCAACCATTCATTCCAGCTTCTAATTAAGAAACTAATGATTATGCTA
CCTTAGCACGGTCAAAATACCGCGGCCATTTAAATCCTCAGAGGGCAGGCTAAT
TATTAGACAAGAAAGGGGGAAAAAAGAAGCTTTTGGAACCTTAGGAATAATTTAT
GCCATAATAGCTATCGGATTATTAGGATTCGTAGTTTGAGCTCACCATATATTTA
CAGTAGGGATAGATGTAGATACCCGAGCTTACTTCACTTCAGCACCTATGATTAT
TGCGGTTCCAACTGGGATTAAAGTCTTTAGATGACTAGCCACCTTTCATGGAACT
CAAATTTTAAATAGACCAGTAACCCTGTGAGCTTTAGGTTTTGTATTTTTATTTAC
AGTAGGAGGATTAACAGGGGTAATTTTAGCTAATTCTTCAATTGATATTATTCTTC

ACGATACTTACTATGTAGTAGCTCATTTTCACTATGTTCTTTCTATAGGAGCTGTA
TTTGCAATTATAGCCGGAATTGTCCAATGATTCCCATTATTTACTGGACTAACCTT
AAATAATTTTTTTTTAAAAACCCAATTTATTACTATATTTTTAGGTGTAAATTTAACA
TTTTTTCCTCAACACTTTTTAGGGCTAAGAGGAATGCCTCGGCGATATTCGGATT
ATCCAGATGCTTTCACTATCTGAAATGCCATTTCATCTATTGGTTCCATTATTTCT
CTCACAAGAATTATCTTTTTTTTTATTTATCTTATGAGAAAGGCTTTCTTCCCAACG
TAAATCTTTGAGAACTTTAAATGTAACAACTTCAATTGAATGGTTACAAGCCCTAC
CCCCTAGTG
MEX_SFP
CGGGCGATGTGTACATATTTTAGAGCTTTAATCATCTTAAAAAAATCTAAAAAATT
ACTCTCAAATCCAAATTCAAATTTTTTTAACAAAAATTATTTTCAAATAAATTTATT
GTAACCCATTTCTTCTTTATTATAAACTACACCTTGATCTGATTTATTTTTTAATAA
AAAATTTTAAATATTTATAATTCTATTAAAATATTTAAACTACGACGATATATAAAC
TAATAAATAAAGTGCATCTAATCGTGGACTATCGATTATAGAACAGGTTCCTCTG
AAAAGACTAAAATACCGCCAAATTTTTTAATTTTCAAGAACATAACTAATACTAAT
TAAGTAAAAAAATTTACATTTTTAATAATAGGGTATCTAACAGACCTAATATTCCC
AGCTTCTGCACCAAAAATTAATTTTAATCCAACATCGAGGTCGCAATCTTTATCTT
CGATTAGAACTCTTTGATAATATTACGCTGTTATCCCTAAGGTAATTAAATCTTAC
AATTATTACTAATAGCTCAAGTATACATAAATTAATGATTTAAATTTAAAAAAAGTT
CAATTAATTTTTCAATCACCCCAATTAAATAATTAAAAAAATTAAAAATTTTTATAC
TaAAAAAAAATTAAAATTTTTAAATATGAAACTCTATAGGGTCTTCTCGTCTTTTAA
ATTAATTTTAGCTTTTTAACTAAAAAATAAATTTTTAATGTTTAAATAGAGACAGAA
ATTTTTTTATCCAACCATTCATTCCAGCTTCTAATTAAGAAACTAATGATTATGCTA
CCTTAGCACGGTCAAAATACCGCGGCCATTTAAATCCTCAGAGGGCAGGCTAAT
TATTAGACAAGAAAGGGGGAAAAAAGAAGCTTTTGGAACCTTAGGAATAATTTAT
GCCATAATAGCTATCGGATTATTAGGATTCGTAGTTTGAGCTCACCATATATTTA
CAGTAGGGATAGATGTAGATACCCGAGCTTACTTCACTTCAGCAACTATAATTAT
TGCGGTTCCAACTGGGATTAAAGTCTTTAGATGACTAGCCACCTTTCATGGAACT
CAAATTTTAAATAGACCAGTAACCCTGTGAGCTTTAGGTTTTGTATTTTTATTTAC
AGTAGGAGGATTAACAGGGGTAATTTTAGCTAATTCTTCAATTGATATTATTCTTC
ACGATACTTACTATGTAGTAGCTCATTTTCACTATGTTCTTTCTATAGGAGCTGTA
TTTGCAATTATAGCCGGAATTGTCCAATGATTCCCATTATTTACTGGACTAACCTT
AAATAATTTTTTTTTAAAAACCCAATTTATTACTATATTTTTAGGTGTAAATTTAACA
TTTTTTCCTCAACACTTTTTAGGGCTAAGAGGAATGCCTCGGCGATATTCGGATT
ATCCAGATGCTTTCACTATCTGAAATGCCATTTCATCTATTGGTTCCATTATTTCT
CTCACAAGAATTATCTTTTTTTTTATTTATCTTATGAGAAAGGCTTTCTTCCCAACG
TAAATCTTTGAGAACTTTAAATGTAACAACTTCAATTGAATGGTTACAAGCCCTAC
CCCCTAGTG
MEX_SJS_2
CGGGCGATGTGTACATATTTTAGAGCTTTAATCATCTTAAAAAAATCTAAAAAATT
ACTCTCAAATCCAAATTCAAATTTTTTTAACAAAAATTATTTTCAAATAAATTTATT
GTAACCCATTTCTTCTTTATTATAAACTACACCTTGATCTGATTTATTTTTTAATAA
AAAATTTTAAATATTTATAATTCTATTAAAATATTTAAACTACGACGATATATAAAC
TAATAAATAAAGTGCATCTAATCGTGGACTATCGATTATAGAACAGGTTCCTCTG
AAAAGACTAAAATACCGCCAAATTTTTTAATTTTCAAGAACATAACTAATACTAAT
TAAGTAAAAAAATTTACATTTTTAATAATAGGGTATCTAACAGACCTAATATTCCC
AGCTTCTGCACCAAAAATTAATTTTAATCCAACATCGAGGTCGCAATCTTTATCTT
CGATTAGAACTCTTTGATAATATTACGCTGTTATCCCTAAGGTAATTAAATCTTAC
AATTATTACTAATAGCTCAAGTATACATAAATTAATGATTTAAATTTAAAAAAAGTT
CAATTAATTTTTCAATCACCCCAATTAAATAATTAAAAAAATTAAAAATTTTTATAC

TaAAAAAAAATTAAAATTTTTAAATATGAAACTCTATAGGGTCTTCTCGTCTTTTAA
ATTAATTTTAGCTTTTTAACTAAAAAATAAATTTTTAATGTTTAAATAGAGACAGAA
ATTTTTTTATCCAACCATTCATTCCAGCTTCTAATTAAGAAACTAATGATTATGCTA
CCTTAGCACGGTCAAAATACCGCGGCCATTTAAATCCTCAGAGGGCAGGCTAAT
TATTAGACAAGAAAGGGGGAAAAAAGAACCTTTTGGAACCTTAGGAATAATTTAT
GCCATAATAGCTATCGGATTATTAGGATTCGTAGTTTGAGCTCACCATATATTTA
CAGTAGGGATAGATGTAGATACCCGAGCTTACTTCACTTCAGCAACTATAATTAT
TGCGGTTCCAACTGGAATTAAAGTCTTTAGATGACTAGCCACCTTTCATGGAACT
CAAATTTTAAATAGACCAGTAACCCTGTGAGCTTTAGGTTTTGTATTTTTATTTAC
AGTAGGAGGATTAACAGGGGTAATTTTAGCTAATTCTTCAATTGATATTATTCTTC
ACGATACTTACTATGTAGTAGCTCATTTTCACTATGTTCTTTCTATAGGAGCTGTA
TTTGCAATTATAGCCGGAATTGTCCAATGATTCCCATTATTTACTGGACTAACCTT
AAATAATTTTTTTTTAAAAACCCAATTTATTACTATATTTTTAGGTGTAAATTTAACA
TTTTTTCCTCAACACTTTTTAGGGCTAAGAGGAATGCCTCGGCGATATTCGGATT
ATCCAGATGCTTTCACTATCTGAAATGCCATTTCATCTATTGGTTCCATTATTTCT
CTCACAAGAATTATCTTTTTTTTATTTATCTTATGAGAAAGGCTTTCTTCCCAACG
TAAATCTTTGAGAACTTTAAATGTAACAACTTCAATTGAATGGTTACAAGCCCTAC
CCCCTAGTG
MEX_STO
CGGGCGATGTGTACATATTTTAGAGCTTTAATCATCTTAAAAAAATCTAAAAAATT
ACTCTCAAATCCAAATTCAAATTTTTTTAACAAAAATTATTTTCAAATAAATTTATT
GTAACCCATTTCTTCTTTATTATAAACTACACCTTGATCTGATTTATTTTTTAATAA
AAAATTTTAAATATTTATAATTCTATTAAAATATTTAAACTACGACGATATATAAAC
TAATAAATAAAGTGCATCTAATCGTGGACTATCGATTATAGAACAGGTTCCTCTG
AAAAGACTAAAATACCGCCAAATTTTTTAATTTTCAAGAACATAACTAATACTAAT
TAAGTAAAAAAATTTACATTTTTAATAATAGGGTATCTAACAGACCTAATATTCCC
AGCTTCTGCACCAAAAATTAATTTTAATCCAACATCGAGGTCGCAATCTTTATCTT
CGATTAGAACTCTTTGATAATATTACGCTGTTATCCCTAAGGTAATTAAATCTTAC
AATTATTACTAATAGCTCAAGTATACATAAATTAATGATTTAAATTTAAAAAAAGTT
CAATTAATTTTTCAATCACCCCAATTAAATAATTAAAAAAATTAAAAATTTTTATAC
TaAAAAAAAATTAAAATTTTTAAATATGAAACTCTATAGGGTCTTCTCGTCTTTTAA
ATTAATTTTAGCTTTTTAACTAAAAAATAAATTTTTAATGTTTAAATAGAGACAGAA
ATTTTTTTATCCAACCATTCATTCCAGCTTCTAATTAAGAAACTAATGATTATGCTA
CCTTAGCACGGTCAAAATACCGCGGCCATTTAAATCCTCAGAGGGCAGGCTAAT
TATTAGACAAGAAAGGGGGAAAAAAGAACCTTTTGGAACCTTAGGAATAATTTAT
GCCATAATAGCTATCGGATTATTAGGATTCGTAGTTTGAGCTCACCATATATTTA
CAGTAGGGATAGATGTAGATACCCGAGCTTACTTCACTTCAGCAACTATAATTAT
TGCGGTTCCAACTGGGATTAAAGTCTTTAGATGACTAGCCACCTTTCATGGAACT
CAAATTTTAAATAGACCAGTAACCCTGTGAGCTTTAGGTTTTGTATTTTTATTTAC
AGTAGGAGGATTAACAGGGGTAATTTTAGCTAATTCTTCAATTGATATTATTCTTC
ACGATACTTACTATGTAGTAGCTCATTTTCACTATGTTCTTTCTATAGGAGCTGTA
TTTGCAATTATAGCCGGAATTGTCCAATGATTCCCATTATTTACTGGACTAACCTT
AAATAATTTTTTTTTAAAAACCCAATTTATTACTATATTTTTAGGTGTAAATTTAACA
TTTTTTCCTCAACACTTTTTAGGGCTAAGAGGAATGCCTCGGCGATATTCGGATT
ATCCAGATGCTTTCACTATCTGAAATGCCATTTCATCTATTGGTTCCATTATTTCT
CTCACAAGAATTATCTTTTTTTTATTTATCTTATGAGAAAGGCTTTCTTCCCAACG
TAAATCTTTGAGAACTTTAAATGTAACAACTTCAATTGAATGGTTACAAGCCCTAC
CCCCTAGTG
MEX_TLA_1
CGGGCGATGTGTACATATTTTAGAGCTTTAATCATCTTAAAAAAATCTAAAAAATT

ACTCTCAAATCCAAATTCAAATTTTTTTAACAAAAATTATTTTCAAATAAATTTATT
GTAACCCATTTCTTCTTTATTATAAACTACACCTTGATCTGATTTATTTTTTAATAA
AAAATTTTAAATATTTATAATTCTATTAAAATATTTAAACTACGACGATATATAAAC
TAATAAATAAAGTGCATCTAATCGTGGACTATCGATTATAGAACAGGTTCCTCTG
AAAAGACTAAAATACCGCCAAATTTTTTAATTTTCAAGAACATAACTAATACTAAT
TAAGTAAAAAAATTTACATTTTTAATAATAGGGTATCTAACAGACCTAATATTCCC
AGCTTCTGCACCAAAAATTAATTTTAATCCAACATCGAGGTCGCAATCTTTATCTT
CGATTAGAACTCTTTGATAATATTACGCTGTTATCCCTAAGGTAATTAAATCTTAC
AATTATTACTAATAGCTCAAGTATACATAAATTAATGATTTAAATTTAAAAAAAGTT
CAATTAATTTTTCAATCACCCCAATTAAATAATTAAAAAAATTAAAAATTTTTATAC
TaAAAAAAAATTAAAATTTTTAAATATGAAACTCTATAGGGTCTTCTCGTCTTTTAA
ATTAATTTTAGCTTTTTAACTAAAAAATAAATTTTTAATGTTTAAATAGAGACAGAA
ATTTTTTTATCCAACCATTCATTCCAGCTTCTAATTAAGAAACTAATGATTATGCTA
CCTTAGCACGGTCAAAATACCGCGGCCATTTAAATCCTCAGAGGGCAGGCTAAT
TATTAGACAAGAAAGGGGGAAAAAAGAAGCTTTTGGAACCTTAGGAATAATTTAT
GCCATAATAGCTATCGGATTATTAGGATTCGTAGTTTGAGCTCACCATATATTTA
CAGTAGGGATAGATGTAGATACCCGAGCTTACTTCACTTCAGCACCTATGATTAT
TGCGGTTCCAACTGGGATTAAAGTCTTTAGATGACTAGCCACCTTTCATGGAACT
CAAATTTTAAATAGACCAGTAACCCTGTGAGCTTTAGGTTTTGTATTTTTATTTAC
AGTAGGAGGATTAACAGGGGTAATTTTAGCTAATTCTTCAATTGATATTATTCTTC
ACGATACTTACTATGTAGTAGCTCATTTTCACTATGTTCTTTCTATAGGAGCTGTA
TTTGCAATTATAGCCGGAATTGTCCAATGATTCCCATTATTTACTGGACTAACCTT
AAATAATTTTTTTTTAAAAACCCAATTTATTACTATATTTTTAGGTGTAAATTTAACA
TTTTTTCCTCAACACTTTTTAGGGCTAAGAGGAATGCCTCGGCGATATTCGGATT
ATCCAGATGCTTTCACTATCTGAAATGCCATTTCATCTATTGGTTCCATTATTTCT
CTCACAAGAATTATCTTTTTTTTTATTTATCTTATGAGAAAGGCTTTCTTCCCAACG
TAAATCTTTGAGAACTTTAAATGTAACAACTTCAATTGAATGGTTACAAGCCCTAC
CCCCTAGTG
MEX_XOT_3
CGGGCGATGTGTACATATTTTAGAGCTTTAATCATCTTAAAAAAATCTAAAAAATT
ACTCTCAAATCCAAATTCAAATTTTTTTAACAAAAATTATTTTAAAATAAATTTATT
GTAACCCATTTCTTCTTTATTATAAACTACACCTTGATCTGATTTATTTTTTAATAA
AAAATTTTAAATATTTATAATTCTATTAAAATATTTAAACTACGACGATATATAAAC
TAATAAATAAAGTGCATCTAATCGTGGACTATCGATTATAGAACAGGTTCCTCTG
AAAAGACTAAAATACCGCCAAATTTTTTAATTTTCAAGAACATAACTAATACTAAT
TAAGTAAAAAAATTTACATTTTTAATAATAGGGTATCTAACAGACCTAATATTTCC
AGCTTCTGCACCAAAAATTAATTTTAATCCAACATCGAGGTCGCAATCTTTATCTT
CGATTAGAACTCTTTGATAATATTACGCTGTTATCCCTAAGGTAATTAAATCTTAC
AATTATTACTAATAGCTCAAGTATACATAAATTAATGATTTAAATTTAAAAAAAGTT
CAATTAATTTTTCAATCACCCCAATTAAATAATTAAAAAAATTAAAAATTTTTATAC
TaAAAAAAAATTAAAATTTTTAAATATGAAACTCTATAGGGTCTTCTCGTCTTTTAA
ATTAATTTTAGCTTTTTAACTAAAAAATAAATTTTTAATGTTTAAATAGAGACAGAA
ATTTTTTTATCCAACCATTCATTCCAGCTTCTAATTAAGAAACTAATGATTATGCTA
CCTTAGCACGGTCAAAATACCGCGGCCATTTAAATCCTCAGAGGGCAGGCTAAT
TATTAGACAAGAAAGGGGGAAAAAAGAAGCTTTTGGAACCTTAGGAATAATTTAT
GCCATGATAGCTATCGGATTATTAGGATTCGTAGTTTGAGCTCACCATATATTTA
CAGTAGGGATAGATGTAGATACCCGAGCTTACTTCACTTCAGCAACTATAATTAT
TGCGGTTCCAACTGGGATTAAAGTCTTTAGATGACTAGCCACCTTTCATGGAACT
CAAATTTTAAATAGACCAGTAACCCTGTGAGCTTTAGGCTTTGTATTTTTATTTAC
AGTAGGAGGATTAACAGGGGTAATTTTAGCTAATTCTTCAATTGATATTATTCTTC

ACGATACTTACTATGTAGTAGCTCATTTTCACTATGTTCTTTCTATAGGAGCTGTA
TTTGCAATTATAGCCGGAATTGTCCAATGATTTCCATTATTTACTGGACTAACCTT
AAATAATTTTTTTTTAAAAACCCAATTTATTACTATATTTTTAGGTGTAAATTTAACA
TTTTTTCCTCAACACTTTTTAGGGCTAAGAGGAATGCCTCGGCGATATTCGGATT
ATCCAGATGCTTTCACTATCTGAAATGCCATTTCATCTATTGGTTCCATTATTTCT
CTCACAAGAATTATCTTTTTTTTATTTATCTTATGAGAAAGGCTTTCTTCCCAACG
TAAATCTTTGAGAACTTTAAATGTAACAACTTCAATTGAATGGTTACAAGCCCTAC
CCCCTAGTG
MEX_YOH
CGGGCGATGTGTACATATTTTAGAGCTTTAATCATCTTAAAAAAATCTAAAAAATT
ACTCTCAAATCCAAATTCAAATTTTTTTAACAAAAATTATTTTCAAATAAATTTATT
GTAACCCATTTCTTCTTTATTATAAACTACACCTTGATCTGATTTATTTTTTAATAA
AAAATTTTAAATATTTATAATTCTATTAAAATATTTAAACTACGACGATATATAAAC
TAATAAATAAAGTGCATCTAATCGTGGACTATCGATTATAGAACAGGTTCCTCTG
AAAAGACTAAAATACCGCCAAATTTTTTAATTTTCAAGAACATAACTAATACTAAT
TAAGTAAAAAAATTTACATTTTTAATAATAGGGTATCTAACAGACCTAATATCCCC
AGCTTCTACACCAAAAATTAATTTTAATCCAACATCGAGGTCGCAATCTTTATCTT
CGATTAGAACTCTTTGATAATATTACGCTGTTATCCCTAAGGTAATTAAATCTTAC
AATTATTACTAATAGCTCAAGTATACATAAATTAATGATTTAAATTTAAAAAAAGTT
CAATTAATTTTTCAATCACCCCAATTAAATAATTAAAAAAATTAAAAATTTTTATAC
TaAAAAAAAATTAAAATTTTTAAATATGAAACTCTATAGGGTCTTCTCGTCTTTTAA
ATTAATTTTAGCTTTTTAACTAAAAAATAAATTTTTAATGTTTAAATAGAGACAGAA
ATTTTTTTATCCAACCATTCATTCCAGCTTCTAATTAAGAAACTAATGATTATGCTA
CCTTAGCACGGTCAAAATACCGCGGCCATTTAAATCCTCAGAGGGCAGGCTAAT
TATTAGACAAGAAAGGGGGAAAAAAGAAGCTTTTGGAACCTTAGGAATAATTTAT
GCTATAATAGCTATCGGATTATTAGGATTCGTAGTTTGAGCTCACCATATATTTAC
AGTAGGAATAGATGTAGATACCCGAGCTTACTTCACTTCAGCAACTATAATTATT
GCGGTTCCAACTGGGATTAAAGTCTTTAGATGACTAGCCACCTTTCATGGAACT
CAAATTTTAAATAGACCAGTAACCCTGTGAGCTTTAGGTTTTGTATTTTTATTTAC
AGTAGGAGGATTAACAGGGGTAATTTTAGCTAATTCTTCAATTGATATTATTCTCC
ACGATACTTACTATGTAGTAGCTCATTTTCACTATGTTCTTTCTATAGGAGCTGTA
TTTGCAATTATAGCCGGAATTGTCCAATGATTCCCATTATTTACCGGACTAACCT
TAAATAATTTTTTTTTAAAAACCCAATTTATTACTATATTTTTAGGTGTAAATTTAAC
ATTTTTTCCTCAACACTTTTTAGGGCTAAGAGGAATGCCTCGGCGATATTCGGAT
TATCCAGATGCTTTCACTATCTGAAATGCCATTTCATCTATTGGTTCCATTATTTC
TCTCACAAGAATTATCTTTTTTTTATTTATCTTATGAGAAAGGCTTTCTTCCCAAC
GTAAATCTTTGAGAACTTTAAATGTAACAACTTCAATTGAATGGTTTCAAGCCCTA
CCCCCTAGTG
moc5
CGGGCGATGTGTACATATTTTAGAGCTTTAATCATCTTAAAAAAATCTAAAAAATT
ACTCTCAAATCCAAATTCAAATTTTTTTAACAAAAATTATTTTAAAATAAATTTATT
GTAACCCATTTCTTCTTTATTATAAACTACACCTTGATCTGATTTATTTTTTAATAA
AAAATTTTAAATATTTATAATTCTATTAAAATATTTAAACTACGACGATATATAAAC
TAATAAATAAAGTGCATCTAATCGTGGACTATCGATTATAGAACAGGTTCCTCTG
AAAAGACTAAAATACCGCCAAATTTTTTAATTTTCAAGAACATAACTAATACTAAT
TAAGTAAAAAAATTTACATTTTTAATAATAGGGTATCTAACAGACCTAATATTTCC
AGCTTCTGCACCAAAAATTAATTTTAATCCAACATCGAGGTCGCAATCTTTATCTT
CGATTAGAACTCTTTGATAATATTACGCTGTTATCCCTAAGGTAATTAAATCTTAC
AATTATTACTAATAGCTCAAGTATACATAAATTAATGATTTAAATTTAAAAAAAGTT
CAATTAATTTTTCAATCACCCCAATTAAATAATTAAAAAAATTAAAAATTTTTATAC

TaAAAAAAAATTAAAATTTTTAAATATGAAACTCTATAGGGTCTTCTCGTCTTTTAA
ATTAATTTTAGCTTTTTAACTAAAAAATAAATTTTTAATGTTTAAATAGAGACAGAA
ATTTTTTTATCCAACCATTCATTCCAGCTTCTAATTAAGAAACTAATGATTATGCTA
CCTTAGCACGGTCAAAATACCGCGGCCATTTAAATCCTCAGAGGGCAGGCTAAT
TATTAGACAAGAAAGGGGGAAAAAAGAAGCTTTTGGAACCTTAGGAATAATTTAT
GCCATGATAGCTATCGGATTATTAGGATTCGTAGTTTGAGCTCACCATATATTTA
CAGTAGGGATAGATGTAGATACCCGAGCTTACTTCACTTCAGCAACTATAATTAT
TGCGGTTCCAACTGGGATTAAAGTCTTTAGATGACTAGCCACCTTTCATGGAACT
CAAATTTTAAATAGACCAGTAACCCTGTGAGCTTTAGGTTTTGTATTTTTATTTAC
AGTAGGAGGATTAACAGGGGTAATTTTAGCTAATTCTTCAATTGATATTATTCTTC
ACGATACTTACTATGTAGTAGCTCATTTTCACTATGTTCTTTCTATAGGAGCTGTA
TTTGCAATTATAGCCGGAATTGTCCAATGATTCCCATTATTTACCGGACTAACCT
TAAATAATTTTTTTTTAAAAACCCAATTTATTACTATATTTTTAGGTGTAAATTTAAC
ATTTTTTCCCCAACACTTTTTAGGGCTAAGAGGAATGCCTCGGCGATATTCGGAT
TATCCAGATGCTTTCACTATCTGAAATGCCATTTCATCTATTGGTTCCATTATTTC
TCTCACAAGAATTATCTTTTTTTTATTTATCTTATGAGAAAGGCTTTCTTCCCAAC
GTAAATCTTTGAGAACTTTAAATGTAACAACTTCAATTGAATGGTTACAAGCCCTA
CCCCCTAGTG
PE2_1
CGGGCGATGTGTACATATTTTAGAGCTTTAATCATCTTAAAAAAATCTAAAAAATT
ACTCTCAAATCCAAATTCAAATTTTTTTAACAAAAATTATTTTCAAATAAATTTATT
GTAACCCATTTCTTCTTTATTATAAACTACACCTTGATCTGATTTATTTTTTAATAA
AAAATTTTAAATATTTATAATTCTATTAAAATATTTAAACTACGACGATATATAAAC
TAATAAATAAAGTGCATCTAATCGTGGACTATCGATTATAGAACAGGTTCCTCTG
AAAAGACTAAAATACCGCCAAATTTTTTAATTTTCAAGAACATAACTAATACTAAT
TAAGTAAAAAAATTTACATTTTTAATAATAGGGTATCTAACAGACCTAATATCCCC
AGCTTCTACACCAAAAATTAATTTTAATCCAACATCGAGGTCGCAATCTTTATCTT
CGATTAGAACTCTTTGATAATATTACGCTGTTATCCCTAAGGTAATTAAATCTTAC
AATTATTACTAATAGCTCAAGTATACATAAATTAATGATTTAAATTTAAAAAAAGTT
CAATTAATTTTTCAATCACCCCAATTAAATAATTAAAAAAATTAAAAATTTTTATAC
TAAAAAAAAAATTAAAATTTTTAAATATGAAACTCTATAGGGTCTTCTCGTCTTTTAA
ATTAATTTTAGCTTTTTAACTAAAAAATAAATTTTTAATGTTTAAATAGAGACAGAA
ATTTTTTTATCCAACCATTCATTCCAGCTTCTAATTAAGAAACTAATGATTATGCTA
CCTTAGCACGGTCAAAATACCGCGGCCATTTAAATCCTCAGAGGGCAGGCTAAT
TATTAGACAAGAAAGGGGGAAAAAAGAAGCTTTTGGAACCTTAGGAATAATTTAT
GCTATAATAGCTATCGGATTATTAGGATTCGTAGTTTGAGCTCACCATATATTTAC
AGTAGGAATAGATGTAGATACCCGAGCTTACTTCACTTCAGCAACTATAATTATT
GCGGTTCCAACTGGGATTAAAGTCTTTAGATGACTAGCCACCTTTCATGGAACT
CAAATTTTAAATAGACCAGTAACCCTGTGAGCTTTAGGTTTTGTATTTTTATTTAC
AGTAGGAGGATTAACAGGGGTAATTTTAGCTAATTCTTCAATTGATATTATTCTCC
ACGATACTTACTATGTAGTAGCTCATTTTCACTATGTTCTTTCTATAGGAGCTGTA
TTTGCAATTATAGCCGGAATTGTCCAATGATTCCCATTATTTACCGGACTAACCT
TAAATAATTTTTTTTTAAAAACCCAATTTATTACTATATTTTTAGGTGTAAATTTAAC
ATTTTTTCCTCAACACTTTTTAGGGCTAAGAGGAATGCCTCGGCGATATTCGGAT
TATCCAGATGCTTTCACTATCTGAAATGCCATTTCATCTATTGGTTCCATTATTTC
TCTCACAAGAATTATCTTTTTTTTATTTATCTTATGAGAAAGGCTTTCTTCCCAAC
GTAAATCTTTGAGAACTTTAAATGTAACAACTTCAATTGAATGGTTTCAAGCCCTA
CCCCCTAGTG
PE2_3
CGGGCGATGTGTACATATTTTAGAGCTTTAATCATCTTAAAAAAATCTAAAAAATT

ACTCTCAAATCCAAATTCAAATTTTTTTAACAAAAATTATTTTCAAATAAATTTATT
GTAACCCATTTCTTCTTTATTATAAACTACACCTTGATCTGATTTATTTTTTAATAA
AAAATTTTAAATATTTATAATTCTATTAAAATATTTAAACTACGACGATATATAAAC
TAATAAATAAAGTGCATCTAATCGTGGACTATCGATTATAGAACAGGTTCCTCTG
AAAAGACTAAAATACCGCCAAATTTTTTAATTTTCAAGAACATAACTAATACTAAT
TAAGTAAAAAAATTTACATTTTTAATAATAGGGTATCTAACAGACCTAATATCCCC
AGCTTCTACACCAAAAATTAATTTTAATCCAACATCGAGGTCGCAATCTTTATCTT
CGATTAGAACTCTTTGATAATATTACGCTGTTATCCCTAAGGTAATTAAATCTTAC
AATTATTACTAATAGCTCAAGTATACATAAATTAATGATTTAAATTTAAAAAAAGTT
CAATTAATTTTTCAATCACCCCAATTAAATAATTAAAAAAATTAAAAATTTTTATAC
TAAAAAAAAATTAAAATTTTTAAATATGAAACTCTATAGGGTCTTCTCGTCTTTTAA
ATTAATTTTAGCTTTTTAACTAAAAAATAAATTTTTAATGTTTAAATAGAGACAGAA
ATTTTTTTATCCAACCATTCATTCCAGCTTCTAATTAAGAAACTAATGATTATGCTA
CCTTAGCACGGTCAAAATACCGCGGCCATTTAAATCCTCAGAGGGCAGGCTAAT
TATTAGACAAGAAAGGGGGAAAAAAGAAGCTTTTGGAACCTTAGGAATAATTTAT
GCTATAATAGCTATCGGATTATTAGGATTCGTAGTTTGAGCTCACCATATATTTAC
AGTAGGAATAGATGTAGATACCCGAGCTTACTTCACTTCAGCAACTATAATTATT
GCGGTTCCAACTGGGATTAAAGTCTTTAGATGACTAGCCACCTTTCATGGAACT
CAAATTTTAAATAGACCAGTAACCCTGTGAGCTTTAGGTTTTGTATTTTTATTTAC
AGTAGGAGGATTAACAGGGGTAATTTTAGCTAATTCTTCAATTGATATTATTCTCC
ACGATACTTACTATGTAGTAGCTCATTTTCACTATGTTCTTTCTATAGGAGCTGTA
TTTGCAATTATAGCCGGAATTGTCCAATGATTCCCATTATTTACCGGACTAACCT
TAAATAATTTTTTTTTAAAAACCCAATTTATTACTATATTTTTAGGTGTAAATTTAAC
ATTTTTTCCTCAACACTTTTTAGGGCTAAGAGGAATGCCTCGGCGATATTCGGAT
TATCCAGATGCTTTCACTATCTGAAATGCCATTTCATCTATTGGTTCCATTATTTC
TCTCACAAGAATTATCTTTTTTTTATTTATCTTATGAGAAAGGCTTTCTTCCCAAC
GTAAATCTTTGAGAACTTTAAATGTAACAACTTCAATTGAATGGTTTCAAGCCCTA
CCCCCTAGTG
PE2_4
CGGGCGATGTGTACATATTTTAGAGCTTTAATCATCTTAAAAAAATCTAAAAAATT
ACTCTCAAATCCAAATTCAAATTTTTTTAACAAAAATTATTTTCAAATAAATTTATT
GTAACCCATTTCTTCTTTATTATAAACTACACCTTGATCTGATTTATTTTTTAATAA
AAAATTTTAAATATTTATAATTCTATTAAAATATTTAAACTACGACGATATATAAAC
TAATAAATAAAGTGCATCTAATCGTGGACTATCGATTATAGAACAGGTTCCTCTG
AAAAGACTAAAATACCGCCAAATTTTTTAATTTTCAAGAACATAACTAATACTAAT
TAAGTAAAAAAATTTACATTTTTAATAATAGGGTATCTAACAGACCTAATATCCCC
AGCTTCTACACCAAAAATTAATTTTAATCCAACATCGAGGTCGCAATCTTTATCTT
CGATTAGAACTCTTTGATAATATTACGCTGTTATCCCTAAGGTAATTAAATCTTAC
AATTATTACTAATAGCTCAAGTATACATAAATTAATGATTTAAATTTAAAAAAAGTT
CAATTAATTTTTCAATCACCCCAATTAAATAATTAAAAAAATTAAAAATTTTTATAC
TAAAAAAAAATTAAAATTTTTAAATATGAAACTCTATAGGGTCTTCTCGTCTTTTAA
ATTAATTTTAGCTTTTTAACTAAAAAATAAATTTTTAATGTTTAAATAGAGACAGAA
ATTTTTTTATCCAACCATTCATTCCAGCTTCTAATTAAGAAACTAATGATTATGCTA
CCTTAGCACGGTCAAAATACCGCGGCCATTTAAATCCTCAGAGGGCAGGCTAAT
TATTAGACAAGAAAGGGGGAAAAAAGAAGCTTTTGGAACCTTAGGAATAATTTAT
GCTATAATAGCTATCGGATTATTAGGATTCGTAGTTTGAGCTCACCATATATTTAC
AGTAGGAATAGATGTAGATACCCGAGCTTACTTCACTTCAGCAACTATAATTATT
GCGGTTCCAACTGGGATTAAAGTCTTTAGATGACTAGCCACCTTTCATGGAACT
CAAATTTTAAATAGACCAGTAACCCTGTGAGCTTTAGGTTTTGTATTTTTATTTAC
AGTAGGAGGATTAACAGGGGTAATTTTAGCTAATTCTTCAATTGATATTATTCTCC

ACGATACTTACTATGTAGTAGCTCATTTTCACTATGTTCTTTCTATAGGAGCTGTA
TTTGCAATTATAGCCGGAATTGTCCAATGATTCCCATTATTTACCGGACTAACCT
TAAATAATTTTTTTTTAAAAACCCAATTTATTACTATATTTTTAGGTGTAAATTTAAC
ATTTTTTCCTCAACACTTTTTAGGGCTAAGAGGAATGCCTCGGCGATATTCGGAT
TATCCAGATGCTTTCACTATCTGAAATGCCATTTCATCTATTGGTTCCATTATTTC
TCTCACAAGAATTATCTTTTTTTTATTTATCTTATGAGAAAGGCTTTCTTCCCAAC
GTAAATCTTTGAGAACTTTAAATGTAACAACTTCAATTGAATGGTTTCAAGCCCTA
CCCCCTAGTG
PE2_5
CGGGCGATGTGTACATATTTTAGAGCTTTAATCATCTTAAAAAAATCTAAAAAATT
ACTCTCAAATCCAAATTCAAATTTTTTTAACAAAAATTATTTTCAAATAAATTTATT
GTAACCCATTTCTTCTTTATTATAAACTACACCTTGATCTGATTTATTTTTTAATAA
AAAATTTTAAATATTTATAATTCTATTAAAATATTTAAACTACGACGATATATAAAC
TAATAAATAAAGTGCATCTAATCGTGGACTATCGATTATAGAACAGGTTCCTCTG
AAAAGACTAAAATACCGCCAAATTTTTTAATTTTCAAGAACATAACTAATACTAAT
TAAGTAAAAAAATTTACATTTTTAATAATAGGGTATCTAACAGACCTAATATCCCC
AGCTTCTACACCAAAAATTAATTTTAATCCAACATCGAGGTCGCAATCTTTATCTT
CGATTAGAACTCTTTGATAATATTACGCTGTTATCCCTAAGGTAATTAAATCTTAC
AATTATTACTAATAGCTCAAGTATACATAAATTAATGATTTAAATTTAAAAAAAGTT
CAATTAATTTTTCAATCACCCCAATTAAATAATTAAAAAAATTAAAAATTTTTATAC
TAAAAAAAAATTAAAATTTTTAAATATGAAACTCTATAGGGTCTTCTCGTCTTTTAA
ATTAATTTTAGCTTTTTAACTAAAAAATAAATTTTTAATGTTTAAATAGAGACAGAA
ATTTTTTTATCCAACCATTCATTCCAGCTTCTAATTAAGAAACTAATGATTATGCTA
CCTTAGCACGGTCAAAATACCGCGGCCATTTAAATCCTCAGAGGGCAGGCTAAT
TATTAGACAAGAAAGGGGGAAAAAAGAAGCTTTTGGAACCTTAGGAATAATTTAT
GCTATAATAGCTATCGGATTATTAGGATTCGTAGTTTGAGCTCACCATATATTTAC
AGTAGGAATAGATGTAGATACCCGAGCTTACTTCACTTCAGCAACTATAATTATT
GCGGTTCCAACTGGGATTAAAGTCTTTAGATGACTAGCCACCTTTCATGGAACT
CAAATTTTAAATAGACCAGTAACCCTGTGAGCTTTAGGTTTTGTATTTTTATTTAC
AGTAGGAGGATTAACAGGGGTAATTTTAGCTAATTCTTCAATTGATATTATTCTCC
ACGATACTTACTATGTAGTAGCTCATTTTCACTATGTTCTTTCTATAGGAGCTGTA
TTTGCAATTATAGCCGGAATTGTCCAATGATTCCCATTATTTACCGGACTAACCT
TAAATAATTTTTTTTTAAAAACCCAATTTATTACTATATTTTTAGGTGTAAATTTAAC
ATTTTTTCCTCAACACTTTTTAGGGCTAAGAGGAATGCCTCGGCGATATTCGGAT
TATCCAGATGCTTTCACTATCTGAAATGCCATTTCATCTATTGGTTCCATTATTTC
TCTCACAAGAATTATCTTTTTTTTATTTATCTTATGAGAAAGGCTTTCTTCCCAAC
GTAAATCTTTGAGAACTTTAAATGTAACAACTTCAATTGAATGGTTTCAAGCCCTA
CCCCCTAGTG
PE2_6
CGGGCGATGTGTACATATTTTAGAGCTTTAATCATCTTAAAAAAATCTAAAAAATT
ACTCTCAAATCCAAATTCAAATTTTTTTAACAAAAATTATTTTCAAATAAATTTATT
GTAACCCATTTCTTCTTTATTATAAACTACACCTTGATCTGATTTATTTTTTAATAA
AAAATTTTAAATATTTATAATTCTATTAAAATATTTAAACTACGACGATATATAAAC
TAATAAATAAAGTGCATCTAATCGTGGACTATCGATTATAGAACAGGTTCCTCTG
AAAAGACTAAAATACCGCCAAATTTTTTAATTTTCAAGAACATAACTAATACTAAT
TAAGTAAAAAAATTTACATTTTTAATAATAGGGTATCTAACAGACCTAATATCCCC
AGCTTCTACACCAAAAATTAATTTTAATCCAACATCGAGGTCGCAATCTTTATCTT
CGATTAGAACTCTTTGATAATATTACGCTGTTATCCCTAAGGTAATTAAATCTTAC
AATTATTACTAATAGCTCAAGTATACATAAATTAATGATTTAAATTTAAAAAAAGTT
CAATTAATTTTTCAATCACCCCAATTAAATAATTAAAAAAATTAAAAATTTTTATAC

TAAAAAAAAAATTAAAATTTTTAAATATGAAACTCTATAGGGTCTTCTCGTCTTTTAA
ATTAATTTTAGCTTTTTAACTAAAAAATAAATTTTTAATGTTTAAATAGAGACAGAA
ATTTTTTTATCCAACCATTCATTCCAGCTTCTAATTAAGAAACTAATGATTATGCTA
CCTTAGCACGGTCAAAATACCGCGGCCATTTAAATCCTCAGAGGGCAGGCTAAT
TATTAGACAAGAAAGGGGGAAAAAAGAAGCTTTTGGAACCTTAGGAATAATTTAT
GCTATAATAGCTATCGGATTATTAGGATTCGTAGTTTGAGCTCACCATATATTTAC
AGTAGGAATAGATGTAGATACCCGAGCTTACTTCACTTCAGCAACTATAATTATT
GCGGTTCCAACTGGGATTAAAGTCTTTAGATGACTAGCCACCTTTCATGGAACT
CAAATTTTAAATAGACCAGTAACCCTGTGAGCTTTAGGTTTTGTATTTTTATTTAC
AGTAGGAGGATTAACAGGGGTAATTTTAGCTAATTCTTCAATTGATATTATTCTCC
ACGATACTTACTATGTAGTAGCTCATTTTCACTATGTTCTTTCTATAGGAGCTGTA
TTTGCAATTATAGCCGGAATTGTCCAATGATTCCCATTATTTACCGGACTAACCT
TAAATAATTTTTTTTTAAAAACCCAATTTATTACTATATTTTTAGGTGTAAATTTAAC
ATTTTTTCCTCAACACTTTTTAGGGCTAAGAGGAATGCCTCGGCGATATTCGGAT
TATCCAGATGCTTTCACTATCTGAAATGCCATTTCATCTATTGGTTCCATTATTTC
TCTCACAAGAATTATCTTTTTTTTATTTATCTTATGAGAAAGGCTTTCTTCCCAAC
GTAAATCTTTGAGAACTTTAAATGTAACAACTTCAATTGAATGGTTTCAAGCCCTA
CCCCCTAGTG
PE2_7
CGGGCGATGTGTACATATTTTAGAGCTTTAATCATCTTAAAAAAATCTAAAAAATT
ACTCTCAAATCCAAATTCAAATTTTTTTAACAAAAATTATTTTCAAATAAATTTATT
GTAACCCATTTCTTCTTTATTATAAACTACACCTTGATCTGATTTATTTTTTAATAA
AAAATTTTAAATATTTATAATTCTATTAAAATATTTAAACTACGACGATATATAAAC
TAATAAATAAAGTGCATCTAATCGTGGACTATCGATTATAGAACAGGTTCCTCTG
AAAAGACTAAAATACCGCCAAATTTTTTAATTTTCAAGAACATAACTAATACTAAT
TAAGTAAAAAAATTTACATTTTTAATAATAGGGTATCTAACAGACCTAATATCCCC
AGCTTCTACACCAAAAATTAATTTTAATCCAACATCGAGGTCGCAATCTTTATCTT
CGATTAGAACTCTTTGATAATATTACGCTGTTATCCCTAAGGTAATTAAATCTTAC
AATTATTACTAATAGCTCAAGTATACATAAATTAATGATTTAAATTTAAAAAAAGTT
CAATTAATTTTTCAATCACCCCAATTAAATAATTAAAAAAATTAAAAATTTTTATAC
TAAAAAAAAAATTAAAATTTTTAAATATGAAACTCTATAGGGTCTTCTCGTCTTTTAA
ATTAATTTTAGCTTTTTAACTAAAAAATAAATTTTTAATGTTTAAATAGAGACAGAA
ATTTTTTTATCCAACCATTCATTCCAGCTTCTAATTAAGAAACTAATGATTATGCTA
CCTTAGCACGGTCAAAATACCGCGGCCATTTAAATCCTCAGAGGGCAGGCTAAT
TATTAGACAAGAAAGGGGGAAAAAAGAAGCTTTTGGAACCTTAGGAATAATTTAT
GCTATAATAGCTATCGGATTATTAGGATTCGTAGTTTGAGCTCACCATATATTTAC
AGTAGGAATAGATGTAGATACCCGAGCTTACTTCACTTCAGCAACTATAATTATT
GCGGTTCCAACTGGGATTAAAGTCTTTAGATGACTAGCCACCTTTCATGGAACT
CAAATTTTAAATAGACCAGTAACCCTGTGAGCTTTAGGTTTTGTATTTTTATTTAC
AGTAGGAGGATTAACAGGGGTAATTTTAGCTAATTCTTCAATTGATATTATTCTCC
ACGATACTTACTATGTAGTAGCTCATTTTCACTATGTTCTTTCTATAGGAGCTGTA
TTTGCAATTATAGCCGGAATTGTCCAATGATTCCCATTATTTACCGGACTAACCT
TAAATAATTTTTTTTTAAAAACCCAATTTATTACTATATTTTTAGGTGTAAATTTAAC
ATTTTTTCCTCAACACTTTTTAGGGCTAAGAGGAATGCCTCGGCGATATTCGGAT
TATCCAGATGCTTTCACTATCTGAAATGCCATTTCATCTATTGGTTCCATTATTTC
TCTCACAAGAATTATCTTTTTTTTATTTATCTTATGAGAAAGGCTTTCTTCCCAAC
GTAAATCTTTGAGAACTTTAAATGTAACAACTTCAATTGAATGGTTTCAAGCCCTA
CCCCCTAGTG
PE_MAN_12
CGGGCGATGTGTACATATTTTAGAGCTTTAATCATCTTAAAAAAATCTAAAAAATT

ACTCTCAAATCCAAATTCAAATTTTTTTAACAAAAATTATTTTCAAATAAATTTATT
GTAACCCATTTCTTCTTTATTATAAACTACACCTTGATCTGATTTATTTTTTAATAA
AAAATTTTAAATATTTATAATTCTATTAAAATATTTAAACTACGACGATATATAAAC
TAATAAATAAAGTGCATCTAATCGTGGACTATCGATTATAGAACAGGTTCCTCTG
AAAAGACTAAAATACCGCCAAATTTTTTAATTTTCAAGAACATAACTAATACTAAT
TAAGTAAAAAAATTTACATTTTTAATAATAGGGTATCTAACAGACCTAATATCCCC
AGCTTCTACACCAAAAATTAATTTTAATCCAACATCGAGGTCGCAATCTTTATCTT
CGATTAGAACTCTTTGATAATATTACGCTGTTATCCCTAAGGTAATTAAATCTTAC
AATTATTACTAATAGCTCAAGTATACATAAATTAATGATTTAAATTTAAAAAAAGTT
CAATTAATTTTTCAATCACCCCAATTAAATAATTAAAAAAATTAAAAATTTTTATAC
TaAAAAAAAATTAAAATTTTTAAATATGAAACTCTATAGGGTCTTCTCGTCTTTTAA
ATTAATTTTAGCTTTTTAACTAAAAAATAAATTTTTAATGTTTAAATAGAGACAGAA
ATTTTTTTATCCAACCATTCATTCCAGCTTCTAATTAAGAAACTAATGATTATGCTA
CCTTAGCACGGTCAAAATACCGCGGCCATTTAAATCCTCAGAGGGCAGGCTAAT
TATTAGTCAAGAAAGGGGGAAAAAAGAACCTTTTGGAACCTTAGGAATAATTTAT
GCTATAATAGCTATCGGATTATTAGGATTCGTAGTTTGAGCTCACCATATATTTAC
AGTAGGAATAGATGTAGATACCCGAGCTTACTTCACTTCAGCAACTATAATTATT
GCGGTTCCAACTGGGATTAAAGTCTTTAGATGACTAGCCACCTTTCATGGAACT
CAAATTTTAAATAGACCAGTAACCCTGTGAGCTTTAGGTTTTGTATTTTTATTTAC
AGTAGGAGGATTAACAGGGGTAATTTTAGCTAATTCTTCAATTGATATTATTCTCC
ACGATACTTACTATGTAGTAGCTCATTTTCACTATGTTCTTTCTATAGGAGCTGTA
TTTGCAATTATAGCCGGAATTGTCCAATGATTCCCATTATTTACCGGACTAACCT
TAAATAATTTTTTTTTAAAAACCCAATTTATTACTATATTTTTAGGTGTAAATTTAAC
ATTTTTTCCTCAACACTTTTTAGGGCTAAGAGGAATGCCTCGGCGATATTCGGAT
TATCCAGATGCTTTCACTATCTGAAATGCCATTTCATCTATTGGTTCCATTATTTC
TCTCACAAGAATTATCTTTTTTTTTATTTATCTTATGAGAAAGGCTTTCTTCCCAAC
GTAAATCTTTGAGAACTTTAAATGTAACAACTTCAATTGAATGGTTTCAAGCCCTA
CCCCCTAGTG
PE_MAN_13
CGGGCGATGTGTACATATTTTAGAGCTTTAATCATCTTAAAAAAATCTAAAAAATT
ACTCTCAAATCCAAATTCAAATTTTTTTAACAAAAATTATTTTCAAATAAATTTATT
GTAACCCATTTCTTCTTTATTATAAACTACACCTTGATCTGATTTATTTTTTAATAA
AAAATTTTAAATATTTATAATTCTATTAAAATATTTAAACTACGACGATATATAAAC
TAATAAATAAAGTGCATCTAATCGTGGACTATCGATTATAGAACAGGTTCCTCTG
AAAAGACTAAAATACCGCCAAATTTTTTAATTTTCAAGAACATAACTAATACTAAT
TAAGTAAAAAAATTTACATTTTTAATAATAGGGTATCTAACAGACCTAATATCCCC
AGCTTCTACACCAAAAATTAATTTTAATCCAACATCGAGGTCGCAATCTTTATCTT
CGATTAGAACTCTTTGATAATATTACGCTGTTATCCCTAAGGTAATTAAATCTTAC
AATTATTACTAATAGCTCAAGTATACATAAATTAATGATTTAAATTTAAAAAAAGTT
CAATTAATTTTTCAATCACCCCAATTAAATAATTAAAAAAATTAAAAATTTTTATAC
TaAAAAAAAATTAAAATTTTTAAATATGAAACTCTATAGGGTCTTCTCGTCTTTTAA
ATTAATTTTAGCTTTTTAACTAAAAAATAAATTTTTAATGTTTAAATAGAGACAGAA
ATTTTTTTATCCAACCATTCATTCCAGCTTCTAATTAAGAAACTAATGATTATGCTA
CCTTAGCACGGTCAAAATACCGCGGCCATTTAAATCCTCAGAGGGCAGGCTAAT
TATTAGACAAGAAAGGGGGAAAAAAGAACCTTTTGGAACCTTAGGAATAATTTAT
GCTATAATAGCTATCGGATTATTAGGATTCGTAGTTTGAGCTCACCATATATTTAC
AGTAGGAATAGATGTAGATACCCGAGCTTACTTCACTTCAGCAACTATAATTATT
GCGGTTCCAACTGGGATTAAAGTCTTTAGATGACTAGCCACCTTTCATGGAACT
CAAATTTTAAATAGACCAGTAACCCTGTGAGCTTTAGGTTTTGTATTTTTATTTAC
AGTAGGAGGATTAACAGGGGTAATTTTAGCTAATTCTTCAATTGATATTATTCTCC

ACGATACTTACTATGTAGTAGCTCATTTTCACTATGTTCTTTCTATAGGAGCTGTA
TTTGCAATTATAGCCGGAATTGTCCAATGATTCCCATTATTTACCGGACTAACCT
TAAATAATTTTTTTTTAAAAACCCAATTTATTACTATATTTTTAGGTGTAAATTTAAC
ATTTTTTCCTCAACACTTTTTAGGGCTAAGAGGAATGCCTCGGCGATATTCGGAT
TATCCAGATGCTTTCACTATCTGAAATGCCATTTCATCTATTGGTTCCATTATTTC
TCTCACAAGAATTATCTTTTTTTTATTTATCTTATGAGAAAGGCTTTCTTCCCAAC
GTAAATCTTTGAGAACTTTAAATGTAACAACTTCAATTGAATGGTTTCAAGCCCTA
CCCCCTAGTG
PE_MAN_15
CGGGCGATGTGTACATATTTTAGAGCTTTAATCATCTTAAAAAAATCTAAAAAATT
ACTCTCAAATCCAAATTCAAATTTTTTTAACAAAAATTATTTTCAAATAAATTTATT
GTAACCCATTTCTTCTTTATTATAAACTACACCTTGATCTGATTTATTTTTTAATAA
AAAATTTTAAATATTTATAATTCTATTAAAATATTTAAACTACGACGATATATAAAC
TAATAAATAAAGTGCATCTAATCGTGGACTATCGATTATAGAACAGGTTCCTCTG
AAAAGACTAAAATACCGCCAAATTTTTTAATTTTCAAGAACATAACTAATACTAAT
TAAGTAAAAAAATTTACATTTTTAATAATAGGGTATCTAACAGACCTAATATCCCC
AGCTTCTACACCAAAAATTAATTTTAATCCAACATCGAGGTCGCAATCTTTATCTT
CGATTAGAACTCTTTGATAATATTACGCTGTTATCCCTAAGGTAATTAAATCTTAC
AATTATTACTAATAGCTCAAGTATACATAAATTAATGATTTAAATTTAAAAAAAGTT
CAATTAATTTTTCAATCACCCCAATTAAATAATTAAAAAAATTAAAAATTTTTATAC
TaAAAAAAAATTAAAATTTTTAAATATGAAACTCTATAGGGTCTTCTCGTCTTTTAA
ATTAATTTTAGCTTTTTAACTAAAAAATAAATTTTTAATGTTTAAATAGAGACAGAA
ATTTTTTTATCCAACCATTCATTCCAGCTTCTAATTAAGAAACTAATGATTATGCTA
CCTTAGCACGGTCAAAATACCGCGGCCATTTAAATCCTCAGAGGGCAGGCTAAT
TATTAGACAAGAAAGGGGGAAAAAAGAACCTTTTGGAACCTTAGGAATAATTTAT
GCTATAATACCTATCGGATTATTAGGATTCGTAGTTTGAGCTCACCATATATTTAC
AGTAGGAATAGATGTAGATACCCGAGCTTACTTCACTTCAGCAACTATAATTATT
GCGGTTCCAACTGGGATTAAAGTCTTTAGATGACTAGCCACCTTTCATGGAACT
CAAATTTTAAATAGACCAGTAACCCTGTGAGCTTTAGGTTTTGTATTTTTATTTAC
AGTAGGAGGATTAACAGGGGTAATTTTAGCTAATTCTTCAATTGATATTATTCTCC
ACGATACTTACTATGTAGTAGCTCATTTTCACTATGTTCTTTCTATAGGAGCTGTA
TTTGCAATTATAGCCGGAATTGTCCAATGATTCCCATTATTTACCGGACTAACCT
TAAATAATTTTTTTTTAAAAACCCAATTTATTACTATATTTTTAGGTGTAAATTTAAC
ATTTTTTCCTCAACACTTTTTAGGGCTAAGAGGAATGCCTCGGCGATATTCGGAT
TATCCAGATGCTTTCACTATCTGAAATGCCATTTCATCCATTGGTTCCATTATTTC
TCTCACAAGAATTATCTTTTTTTTATTTATCTTATGAGAAAGGCTTTCTTCCCAAC
GTAAATCTGTGAGAACTTTAAATGTAACAACTTCAATTGAATGGTTTCAAGCCCT
ACCCCCTAGTG
PE_MAN_16
CGGGCGATGTGTACATATTTTAGAGCTTTAATCATCTTAAAAAAATCTAAAAAATT
ACTCTCAAATCCAAATTCAAATTTTTTTAACAAAAATTATTTTCAAATAAATTTATT
GTAACCCATTTCTTCTTTATTATAAACTACACCTTGATCTGATTTATTTTTTAATAA
AAAATTTTAAATATTTATAATTCTATTAAAATATTTAAACTACGACGATATATAAAC
TAATAAATAAAGTGCATCTAATCGTGGACTATCGATTATAGAACAGGTTCCTCTG
AAAAGACTAAAATACCGCCAAATTTTTTAATTTTCAAGAACATAACTAATACTAAT
TAAGTAAAAAAATTTACATTTTTAATAATAGGGTATCTAACAGACCTAATATCCCC
AGCTTCTACACCAAAAATTAATTTTAATCCAACATCGAGGTCGCAATCTTTATCTT
CGATTAGAACTCTTTGATAATATTACGCTGTTATCCCTAAGGTAATTAAATCTTAC
AATTATTACTAATAGCTCAAGTATACATAAATTAATGATTTAAATTTAAAAAAAGTT
CAATTAATTTTTCAATCACCCCAATTAAATAATTAAAAAAATTAAAAATTTTTATAC

TaAAAAAAAATTAAAATTTTTAAATATGAAACTCTATAGGGTCTTCTCGTCTTTTAA
ATTAATTTTAGCTTTTTAACTAAAAAATAAATTTTTAATGTTTAAATAGAGACAGAA
ATTTTTTTATCCAACCATTCATTCCAGCTTCTAATTAAGAAACTAATGATTATGCTA
CCTTAGCACGGTCAAAATACCGCGGCCATTTAAATCCTCAGAGGGCAGGCTAAT
TATTAGACAAGAAAGGGGGAAAAAAGAAGCTTTTGGAACCTTAGGAATAATTTAT
GCTATAATAGCTATCGGATTATTAGGATTCGTAGTTTGAGCTCACCATATATTTAC
AGTAGGAATAGATGTAGATACCCGAGCTTACTTCACTTCAGCAACTATAATTATT
GCGGTTCCAACTGGGATTAAAGTCTTTAGATGACTAGCCACCTTTCATGGAACT
CAAATTTTAAATAGACCAGTCACCCTGTGAGCTTTAGGTTTTGTATTTTTATTTAC
AGTAGGAGGATTAACAGGGGTAATTTTAGCTAATTCTTCAATTGATATTATTCTCC
ACGATACTTACTATGTAGTAGCTCATTTTCACTATGTTCTTTCTATAGGAGCTGTA
TTTGCAATTATAGCCGGAATTGTCCAATGATTCCCATTATTTACCGGACTAACCT
TAAATAATTTTTTTTTAAAAACCCAATTTATTACTATATTTTTAGGTGTAAATTTAAC
ATTTTTTCCTCAACACTTTTTAGGGCTAAGAGGAATGCCTCGGCGATATTCGGAT
TATCCAGATGCTTTCACTATCTGAAATGCCATTTCATCTATTGGTTCCATTATTTC
TCTCACAAGAATTATCTTTTTTTTATTTATCTTATGAGAAAGGCTTTCTTCCCAAC
GTAAATCTTTGAGAACTTTAAATGTAACAACTTCAATTGAATGGTTTCAAGCCCTA
CCCCCTAGTG
PE_MAN_19
CGGGCGATGTGTACATATTTTAGAGCTTTAATCATCTTAAAAAAATCTAAAAAATT
ACTCTCAAATCCAAATTCAAATTTTTTTAACAAAAATTATTTTCAAATAAATTTATT
GTAACCCATTTCTTCTTTATTATAAACTACACCTTGATCTGATTTATTTTTTAATAA
AAAATTTTAAATATTTATAATTCTATTAAAATATTTAAACTACGACGATATATAAAC
TAATAAATAAAGTGCATCTAATCGTGGACTATCGATTATAGAACAGGTTCCTCTG
AAAAGACTAAAATACCGCCAAATTTTTTAATTTTCAAGAACATAACTAATACTAAT
TAAGTAAAAAAATTTACATTTTTAATAATAGGGTATCTAACAGACCTAATATCCCC
AGCTTCTACACCAAAAATTAATTTTAATCCAACATCGAGGTCGCAATCTTTATCTT
CGATTAGAACTCTTTGATAATATTACGCTGTTATCCCTAAGGTAATTAAATCTTAC
AATTATTACTAATAGCTCAAGTATACATAAATTAATGATTTAAATTTAAAAAAAGTT
CAATTAATTTTTCAATCACCCCAATTAAATAATTAAAAAAATTAAAAATTTTTATAC
TaAAAAAAAATTAAAATTTTTAAATATGAAACTCTATAGGGTCTTCTCGTCTTTTAA
ATTAATTTTAGCTTTTTAACTAAAAAATAAATTTTTAATGTTTAAATAGAGACAGAA
ATTTTTTTATCCAACCATTCATTCCAGCTTCTAATTAAGAAACTAATGATTATGCTA
CCTTAGCACGGTCAAAATACCGCGGCCATTTAAATCCTCAGAGGGCAGGCTAAT
TATTAGACAAGAAAGGGGGAAAAAAGAAGCTTTTGGAACCTTAGGAATAATTTAT
GCTATAATAGCTATCGGATTATTAGGATTCGTAGTTTGAGCTCACCATATATTTAC
AGTAGGAATAGATGTAGATACCCGAGCTTACTTCACTTCAGCAACTATAATTATT
GCGGTTCCAACTGGGATTAAAGTCTTTAGATGACTAGCCACCTTTCATGGAACT
CAAATTTTAAATAGACCAGTCACCCTGTGAGCTTTAGGTTTTGTATTTTTATTTAC
AGTAGGAGGATTAACAGGGGTAATTTTAGCTAATTCTTCAATTGATATTATTCTCC
ACGATACTTACTATGTAGTAGCTCATTTTCACTATGTTCTTTCTATAGGAGCTGTA
TTTGCAATTATAGCCGGAATTGTCCAATGATTCCCATTATTTACCGGACTAACCT
TAAATAATTTTTTTTTAAAAACCCAATTTATTACTATATTTTTAGGTGTAAATTTAAC
ATTTTTTCCTCAACACTTTTTAGGGCTAAGAGGAATGCCTCGGCGATATTCGGAT
TATCCAGATGCTTTCACTATCTGAAATGCCATTTCATCCATTGGTTCCATTATTTC
TCTCACAAGAATTATCTTTTTTTTATTTATCTTATGAGAAAGGCTTTCTTCCCAAC
GTAAATCTTTGAGAACTTTAAATGTAACAACTTCAATTGAATGGTTTCAAGCCCTA
CCCCCTAGTG
PE_MAN_2
CGGGCGATGTGTACATATTTTAGAGCTTTAATCATCTTAAAAAAATCTAAAAAATT

ACTCTCAAATCCAAATTCAAATTTTTTTAACAAAAATTATTTTCAAATAAATTTATT
GTAACCCATTTCTTCTTTATTATAAACTACACCTTGATCTGATTTATTTTTTAATAA
AAAATTTTAAATATTTATAATTCTATTAAAATATTTAAACTACGACGATATATAAAC
TAATAAATAAAGTGCATCTAATCGTGGACTATCGATTATAGAACAGGTTCCTCTG
AAAAGACTAAAATACCGCCAAATTTTTTAATTTTCAAGAACATAACTAATACTAAT
TAAGTAAAAAAATTTACATTTTTAATAATAGGGTATCTAACAGACCTAATATCCCC
AGCTTCTACACCAAAAATTAATTTTAATCCAACATCGAGGTCGCAATCTTTATCTT
CGATTAGAACTCTTTGATAATATTACGCTGTTATCCCTAAGGTAATTAAATCTTAC
AATTATTACTAATAGCTCAAGTATACATAAATTAATGATTTAAATTTAAAAAAAGTT
CAATTAATTTTTCAATCACCCCAATTAAATAATTAAAAAAATTAAAAATTTTTATAC
TaAAAAAAAATTAAAATTTTTAAATATGAAACTCTATAGGGTCTTCTCGTCTTTTAA
ATTAATTTTAGCTTTTTAACTAAAAAATAAATTTTTAATGTTTAAATAGAGACAGAA
ATTTTTTTATCCAACCATTCATTCCAGCTTCTAATTAAGAAACTAATGATTATGCTA
CCTTAGCACGGTCAAAATACCGCGGCCATTTAAATCCTCAGAGGGCAGGCTAAT
TATTAGACAAGAAAGGGGGAAAAAAGAAGCTTTTGGAACCTTAGGAATAATTTAT
GCTATAATAGCTATCGGATTATTAGGATTCGTAGTTTGAGCTCACCATATATTTAC
AGTAGGAATAGATGTAGATACCCGAGCTTACTTCACTTCAGCAACTATAATTATT
GCGGTTCCAACTGGGATTAAAGTCTTTAGATGACTAGCCACCTTTCATGGAACT
CAAATTTTAAATAGACCAGTCACCCTGTGAGCTTTAGGTTTTGTATTTTTATTTAC
AGTAGGAGGATTAACAGGGGTAATTTTAGCTAATTCTTCAATTGATATTATTCTCC
ACGATACTTACTATGTAGTAGCTCATTTTCACTATGTTCTTTCTATAGGAGCTGTA
TTTGCAATTATAGCCGGAATTGTCCAATGATTCCCATTATTTACCGGACTAACCT
TAAATAATTTTTTTTTAAAAACCCAATTTATTACTATATTTTTAGGTGTAAATTTAAC
ATTTTTTCCTCAACACTTTTTAGGGCTAAGAGGAATGCCTCGGCGATATTCGGAT
TATCCAGATGCTTTCACTATCTGAAATGCCATTTCATCTATTGGTTCCATTATTTC
TCTCACAAGAATTATCTTTTTTTTTATTTATCTTATGAGAAAGGCTTTCTTCCCAAC
GTAAATCTTTGAGAACTTTAAATGTAACAACTTCAATTGAATGGTTTCAAGCCCTA
CCCCCTAGTG
PE_MAN_23
CGGGCGATGTGTACATATTTTAGAGCTTTAATCATCTTAAAAAAATCTAAAAAATT
ACTCTCAAATCCAAATTCAAATTTTTTTAACAAAAATTATTTTCAAATAAATTTATT
GTAACCCATTTCTTCTTTATTATAAACTACACCTTGATCTGATTTATTTTTTAATAA
AAAATTTTAAATATTTATAATTCTATTAAAATATTTAAACTACGACGATATATAAAC
TAATAAATAAAGTGCATCTAATCGTGGACTATCGATTATAGAACAGGTTCCTCTG
AAAAGACTAAAATACCGCCAAATTTTTTAATTTTCAAGAACATAACTAATACTAAT
TAAGTAAAAAAATTTACATTTTTAATAATAGGGTATCTAACAGACCTAATATCCCC
AGCTTCTACACCAAAAATTAATTTTAATCCAACATCGAGGTCGCAATCTTTATCTT
CGATTAGAACTCTTTGATAATATTACGCTGTTATCCCTAAGGTAATTAAATCTTAC
AATTATTACTAATAGCTCAAGTATACATAAATTAATGATTTAAATTTAAAAAAAGTT
CAATTAATTTTTCAATCACCCCAATTAAATAATTAAAAAAATTAAAAATTTTTATAC
TaAAAAAAAATTAAAATTTTTAAATATGAAACTCTATAGGGTCTTCTCGTCTTTTAA
ATTAATTTTAGCTTTTTAACTAAAAAATAAATTTTTAATGTTTAAATAGAGACAGAA
ATTTTTTTATCCAACCATTCATTCCAGCTTCTAATTAAGAAACTAATGATTATGCTA
CCTTAGCACGGTCAAAATACCGCGGCCATTTAAATCCTCAGAGGGCAGGCTAAT
TATTAGACAAGAAAGGGGGAAAAAAGAAGCTTTTGGAACCTTAGGAATAATTTAT
GCTATAATAGCTATCGGATTATTAGGATTCGTAGTTTGAGCTCACCATATATTTAC
AGTAGGAATAGATGTAGATACCCGAGCTTACTTCACTTCAGCAACTATAATTATT
GCGGTTCCAACTGGGATTAAAGTCTTTAGATGACTAGCCACCTTTCATGGAACT
CAAATTTTAAATAGACCAGTAACCCTGTGAGCTTTAGGTTTTGTATTTTTATTTAC
AGTAGGAGGATTAACAGGGGTAATTTTAGCTAATTCTTCAATTGATATTATTCTCC

ACGATACTTACTATGTAGTAGCTCATTTTCACTATGTTCTTTCTATAGGAGCTGTA
TTTGCAATTATAGCCGGAATTGTCCAATGATTCCCATTATTTACCGGACTAACCT
TAAATAATTTTTTTTTAAAAACCCAATTTATTACTATATTTTTAGGTGTAAATTTAAC
ATTTTTTCCTCAACACTTTTTAGGGCTAAGAGGAATGCCTCGGCGATATTCGGAT
TATCCAGATGCTTTCACTATCTGAAATGCCATTTCATCTATTGGTTCCATTATTTC
TCTCACAAGAATTATCTTTTTTTTATTTATCTTATGAGAAAGGCTTTCTTCCCAAC
GTAAATCTTTGAGAACTTTAAATGTAACAACTTCAATTGAATGGTTTCAAGCCCTA
CCCCCTAGTG
PE_MAN_29
CGGGCGATGTGTACATATTTTAGAGCTTTAATCATCTTAAAAAAATCTAAAAAATT
ACTCTCAAATCCAAATTCAAATTTTTTTAACAAAAATTATTTTCAAATAAATTTATT
GTAACCCATTTCTTCTTTATTATAAACTACACCTTGATCTGATTTATTTTTTAATAA
AAAATTTTAAATATTTATAATTCTATTAAAATATTTAAACTACGACGATATATAAAC
TAATAAATAAAGTGCATCTAATCGTGGACTATCGATTATAGAACAGGTTCCTCTG
AAAAGACTAAAATACCGCCAAATTTTTTAATTTTCAAGAACATAACTAATACTAAT
TAAGTAAAAAAATTTACATTTTTAATAATAGGGTATCTAACAGACCTAATATCCCC
AGCTTCTACACCAAAAATTAATTTTAATCCAACATCGAGGTCGCAATCTTTATCTT
CGATTAGAACTCTTTGATAATATTACGCTGTTATCCCTAAGGTAATTAAATCTTAC
AATTATTACTAATAGCTCAAGTATACATAAATTAATGATTTAAATTTAAAAAAAGTT
CAATTAATTTTTCAATCACCCCAATTAAATAATTAAAAAAATTAAAAATTTTTATAC
TaAAAAAAAATTAAAATTTTTAAATATGAAACTCTATAGGGTCTTCTCGTCTTTTAA
ATTAATTTTAGCTTTTTAACTAAAAAATAAATTTTTAATGTTTAAATAGAGACAGAA
ATTTTTTTATCCAACCATTCATTCCAGCTTCTAATTAAGAAACTAATGATTATGCTA
CCTTAGCACGGTCAAAATACCGCGGCCATTTAAATCCTCAGAGGGCAGGCTAAT
TATTAGACAAGAAAGGGGGAAAAAAGAAGCTTTTGGAACCTTAGGAATAATTTAT
GCTATAATAGCTATCGGATTATTAGGATTCGTAGTTTGAGCTCACCATATATTTAC
AGTAGGAATAGATGTAGATACCCGAGCTTACTTCACTTCAGCAACTATAATTATT
GCGGTTCCAACTGGGATTAAAGTCTTTAGATGACTAGCCACCTTTCATGGAACT
CAAATTTTAAATAGACCAGTCACCCTGTGAGCTTTAGGTTTTGTATTTTTATTTAC
AGTAGGAGGATTAACAGGGGTAATTTTAGCTAATTCTTCAATTGATATTATTCTCC
ACGATACTTACTATGTAGTAGCTCATTTTCACTATGTTCTTTCTATAGGAGCTGTA
TTTGCAATTATAGCCGGAATTGTCCAATGATTCCCATTATTTACCGGACTAACCT
TAAATAATTTTTTTTTAAAAACCCAATTTATTACTATATTTTTAGGTGTAAATTTAAC
ATTTTTTCCTCAACACTTTTTAGGGCTAAGAGGAATGCCTCGGCGATATTCGGAT
TATCCAGATGCTTTCACTATCTGAAATGCCATTTCATCTATTGGTTCCATTATTTC
TCTCACAAGAATTATCTTTTTTTTATTTATCTTATGAGAAAGGCTTTCTTCCCAAC
GTAAATCTTTGAGAACTTTAAATGTAACAACTTCAATTGAATGGTTTCAAGCCCTA
CCCCCTAGTG
PE_MAN_6
CGGGCGATGTGTACATATTTTAGAGCTTTAATCATCTTAAAAAAATCTAAAAAATT
ACTCTCAAATCCAAATTCAAATTTTTTTAACAAAAATTATTTTCAAATAAATTTATT
GTAACCCATTTCTTCTTTATTATAAACTACACCTTGATCTGATTTATTTTTTAATAA
AAAATTTTAAATATTTATAATTCTATTAAAATATTTAAACTACGACGATATATAAAC
TAATAAATAAAGTGCATCTAATCGTGGACTATCGATTATAGAACAGGTTCCTCTG
AAAAGACTAAAATACCGCCAAATTTTTTAATTTTCAAGAACATAACTAATACTAAT
TAAGTAAAAAAATTTACATTTTTAATAATAGGGTATCTAACAGACCTAATATCCCC
AGCTTCTACACCAAAAATTAATTTTAATCCAACATCGAGGTCGCAATCTTTATCTT
CGATTAGAACTCTTTGATAATATTACGCTGTTATCCCTAAGGTAATTAAATCTTAC
AATTATTACTAATAGCTCAAGTATACATAAATTAATGATTTAAATTTAAAAAAAGTT
CAATTAATTTTTCAATCACCCCAATTAAATAATTAAAAAAATTAAAAATTTTTATAC

TaAAAAAAAATTAAAATTTTTAAATATGAAACTCTATAGGGTCTTCTCGTCTTTTAA
ATTAATTTTAGCTTTTTAACTAAAAAATAAATTTTTAATGTTTAAATAGAGACAGAA
ATTTTTTTATCCAACCATTCATTCCAGCTTCTAATTAAGAAACTAATGATTATGCTA
CCTTAGCACGGTCAAAATACCGCGGCCATTTAAATCCTCAGAGGGCAGGCTAAT
TATTAGACAAGAAAGGGGGAAAAAAGAAGCTTTTGGAACCTTAGGAATAATTTAT
GCTATAATAGCTATCGGATTATTAGGATTCGTAGTTTGAGCTCACCATATATTTAC
AGTAGGAATAGATGTAGATACCCGAGCTTACTTCACTTCAGCAACTATAATTATT
GCGGTTCCAACTGGGATTAAAGTCTTTAGATGACTAGCCACCTTTCATGGAACT
CAAATTTTAAATAGACCAGTAACCCTGTGAGCTTTAGGTTTTGTATTTTTATTTAC
AGTAGGAGGATTAACAGGGGTAATTTTAGCTAATTCTTCAATTGATATTATTCTCC
ACGATACTTACTATGTAGTAGCTCATTTTCACTATGTTCTTTCTATAGGAGCTGTA
TTTGCAATTATAGCCGGAATTGTCCAATGATTCCCATTATTTACCGGACTAACCT
TAAATAATTTTTTTTTAAAAACCCAATTTATTACTATATTTTTAGGTGTAAATTTAAC
ATTTTTTCCTCAACACTTTTTAGGGCTAAGAGGAATGCCTCGGCGATATTCGGAT
TATCCAGATGCTTTCACTATCTGAAATGCCATTTCATCTATTGGTTCCATTATTTC
TCTCACAAGAATTATCTTTTTTTTATTTATCTTATGAGAAAGGCTTTCTTCCCAAC
GTAAATCTTTGAGAACTTTAAATGTAACAACTTCAATTGAATGGTTTCAAGCCCTA
CCCCCTAGTG
PE_MAN_8
CGGGCGATGTGTACATATTTTAGAGCTTTAATCATCTTAAAAAAATCTAAAAAATT
ACTCTCAAATCCAAATTCAAATTTTTTTAACAAAAATTATTTTCAAATAAATTTATT
GTAACCCATTTCTTCTTTATTATAAACTACACCTTGATCTGATTTATTTTTTAATAA
AAAATTTTAAATATTTATAATTCTATTAAAATATTTAAACTACGACGATATATAAAC
TAATAAATAAAGTGCATCTAATCGTGGACTATCGATTATAGAACAGGTTCCTCTG
AAAAGACTAAAATACCGCCAAATTTTTTAATTTTCAAGAACATAACTAATACTAAT
TAAGTAAAAAAATTTACATTTTTAATAATAGGGTATCTAACAGACCTAATATCCCC
AGCTTCTACACCAAAAATTAATTTTAATCCAACATCGAGGTCGCAATCTTTATCTT
CGATTAGAACTCTTTGATAATATTACGCTGTTATCCCTAAGGTAATTAAATCTTAC
AATTATTACTAATAGCTCAAGTATACATAAATTAATGATTTAAATTTAAAAAAAGTT
CAATTAATTTTTCAATCACCCCAATTAAATAATTAAAAAAATTAAAAATTTTTATAC
TaAAAAAAAATTAAAATTTTTAAATATGAAACTCTATAGGGTCTTCTCGTCTTTTAA
ATTAATTTTAGCTTTTTAACTAAAAAATAAATTTTTAATGTTTAAATAGAGACAGAA
ATTTTTTTATCCAACCATTCATTCCAGCTTCTAATTAAGAAACTAATGATTATGCTA
CCTTAGCACGGTCAAAATACCGCGGCCATTTAAATCCTCAGAGGGCAGGCTAAT
TATTAGACAAGAAAGGGGGAAAAAAGAAGCTTTTGGAACCTTAGGAATAATTTAT
GCTATAATAGCTATCGGATTATTAGGATTCGTAGTTTGAGCTCACCATATATTTAC
AGTAGGAATAGATGTAGATACCCGAGCTTACTTCACTTCAGCAACTATAATTATT
GCGGTTCCAACTGGGATTAAAGTCTTTAGATGACTAGCCACCTTTCATGGAACT
CAAATTTTAAATAGACCAGTAACCCTGTGAGCTTTAGGTTTTGTATTTTTATTTAC
AGTAGGAGGATTAACAGGGGTAATTTTAGCTAATTCTTCAATTGATATTATTCTCC
ACGATACTTACTATGTAGTAGCTCATTTTCACTATGTTCTTTCTATAGGAGCTGTA
TTTGCAATTATAGCCGGAATTGTCCAATGATTCCCATTATTTACCGGACTAACCT
TAAATAATTTTTTTTTAAAAACCCAATTTATTACTATATTTTTAGGTGTAAATTTAAC
ATTTTTTCCTCAACACTTTTTAGGGCTAAGAGGAATGCCTCGGCGATATTCGGAT
TATCCAGATGCTTTCACTATCTGAAATGCCATTTCATCTATTGGTTCCATTATTTC
TCTCACAAGAATTATCTTTTTTTTATTTATCTTATGAGAAAGGCTTTCTTCCCAAC
GTAAATCTTTGAGAACTTTAAATGTAACAACTTCAATTGAATGGTTTCAAGCCCTA
CCCCCTAGTG
pit1
CGGGCGATGTGTACATATTTTAGAGCTTTAATCATCTTAAAAAAATCTAAAAAATT

ACTCTCAAATCCAAATTCAAATTTTTTTAACAAAAATTATTTTAAAATAAATTTATT
GTAACCCATTTCTTCTTTATTATAAACTACACCTTGATCTGATTTATTTTTTAATAA
AAAATTTTAAATATTTATAATTCTATTAAAATATTTAAACTACGACGATATATAAAC
TAATAAATAAAGTGCATCTAATCGTGGACTATCGATTATAGAACAGGTTCCTCTG
AAAAGACTAAAATACCGCCAAATTTTTTAATTTTCAAGAACATAACTAATACTAAT
TAAGTAAAAAAATTTACATTTTTAATAATAGGGTATCTAACAGACCTAATATTTCC
AGCTTCTGCACCAAAAATTAATTTTAATCCAACATCGAGGTCGCAATCTTTATCTT
CGATTAGAACTCTTTGATAATATTACGCTGTTATCCCTAAGGTAATTAAATCTTAC
AATTATTACTAATAGCTCAAGTATACATAAATTAATGATTTAAATTTAAAAAAAGTT
CGATTAATTTTTCAATCACCCCAATTAAATAATTAAAAAAATTAAAAATTTTTATAC
TaAAAAAAAATTAAAATTTTTAAATATGAAACTCTATAGGGTCTTCTCGTCTTTTAA
ATTAATCTTAGCTTTTTAACTAAAAAATAAATTTTTAATGTTTAAATAGAGACAGAA
ATTTTTTTATCCAACCATTCATTCCAGCTTCTAATTAAGAAACTAATGATTATGCTA
CCTTAGCACGGTCAAAATACCGCGGCCATTTAAATCCTCAGAGGGCAGGCTAAT
TATTAGACAAGAAAGGGGGAAAAAAGAAGCTTTTGGAACCTTAGGAATAATTTAT
GCCATGATAGCTATCGGATTATTAGGATTCGTAGTTTGAGCTCACCATATATTTA
CAGTAGGGATAGATGTAGATACCCGAGCTTACTTCACTTCAGCAACTATAATTAT
CGCGGTTCCAACTGGGATTAAAGTCTTTAGGTGACTAGCCACCTTTCATGGAAC
TCAAATTTTAAATAGACCAGTAACCCTGTGAGCTTTAGGTTTTGTATTTTTATTTA
CAGTAGGAGGATTAACAGGGGTAATTTTAGCTAATTCTTCAATTGATATTATTCTT
CACGATACTTACTATGTAGTAGCTCATTTTCACTATGTTCTTTCTATAGGAGCTGT
ATTTGCAATTATAGCCGGAATTGTCCAATGATTCCCATTATTTACTGGACTAACCT
TAAATAATTTTTTTTTAAAAACCCAATTTATTACTATATTTTTAGGTGTAAATTTAAC
ATTTTTTCCTCAACACTTTTTAGGGCTAAGAGGAATGCCTCGGCGATATTCGGAT
TATCCAGATGCTTTCACTATCTGAAATGCCATTTCATCTATTGGTTCCATTATTTC
TCTCACAAGAATTATCTTTTTTTTTATTTATCTTATGAGAAAGGCTTTCTTCCCAAC
GTAAATCTTTGAGAACTTTAAATGTAACAACTTCAATTGAATGGTTACAAGCCCTA
CCCCCTAGTG
pit2
CGGGCGATGTGTACATATTTTAGAGCTTTAATCATCTTAAAAAAATCTAAAAAATT
ACTCTCAAATCCAAATTCAAATTTTTTTAACAAAAATTATTTTAAAATAAATTTATT
GTAACCCATTTCTTCTTTATTATAAACTACACCTTGATCTGATTTATTTTTTAATAA
AAAATTTTAAATATTTATAATTCTATTAAAATATTTAAACTACGACGATATATAAAC
TAATAAATAAAGTGCATCTAATCGTGGACTATCGATTATAGAACAGGTTCCTCTG
AAAAGACTAAAATACCGCCAAATTTTTTAATTTTCAAGAACATAACTAATACTAAT
TAAGTAAAAAAATTTACATTTTTAATAATAGGGTATCTAACAGACCTAATATTTCC
AGCTTCTGCACCAAAAATTAATTTTAATCCAACATCGAGGTCGCAATCTTTATCTT
CGATTAGAACTCTTTGATAATATTACGCTGTTATCCCTAAGGTAATTAAATCTTAC
AATTATTACTAATAGCTCAAGTATACATAAATTAATGATTTAAATTTAAAAAAAGTT
CGATTAATTTTTCAATCACCCCAATTAAATAATTAAAAAAATTAAAAATTTTTATAC
TaAAAAAAAATTAAAATTTTTAAATATGAAACTCTATAGGGTCTTCTCGTCTTTTAA
ATTAATCTTAGCTTTTTAACTAAAAAATAAATTTTTAATGTTTAAATAGAGACAGAA
ATTTTTTTATCCAACCATTCATTCCAGCTTCTAATTAAGAAACTAATGATTATGCTA
CCTTAGCACGGTCAAAATACCGCGGCCATTTAAATCCTCAGAGGGCAGGCTAAT
TATTAGACAAGAAAGGGGGAAAAAAGAAGCTTTTGGAACCTTAGGAATAATTTAT
GCCATGATAGCTATCGGATTATTAGGATTCGTAGTTTGAGCTCACCATATATTTA
CAGTAAGGATAGATGTAGATACCCGAGCTTACTTCACTTCAGCAACTATAATTAT
CGCGGTTCCAACTGGGATTAAAGTCTTTAGGTGACTAGCCACCTTTCATGGAAC
TCAAATTTTAAATAGACCAGTAACCCTGTGAGCTTTAGGTTTTGTATTTTTATTTA
CAGTAGGAGGATTAACAGGGGTAATTTTAGCTAATTCTTCAATTGATATTATTCTT

CACGATACTTACTATGTAGTAGCTCATTTTCACTATGTTCTTTCTATAGGAGCTGT
ATTTGCAATTATAGCCGGAATTGTCCAATGATTCCCATTATTTACTGGACTAACCT
TAAATAATTTTTTTTTAAAAACCCAATTTATTACTATATTTTTAGGTGTAAATTTAAC
ATTTTTTCCTCAACACTTTTTAGGGCTAAGAGGAATGCCTCGGCGATATTCGGAT
TATCCAGATGCTTTCACTATCTGAAATGCCATTTCATCTATTGGTTCCATTATTTC
TCTCACAAGAATTATCTTTTTTTTATTTATCTTATGAGAAAGGCTTTCTTCCCAAC
GTAAATCTTTGAGAACTTTAAATGTAACAACTTCAATTGAATGGTTACAAGCCCTA
CCCCCTAGTG
pit4
CGGGCGATGTGTACATATTTTAGAGCTTTAATCATCTTAAAAAAATCTAAAAAATT
ACTCTCAAATCCAAATTCAAATTTTTTTAACAAAAATTATTTTAAAATAAATTTATT
GTAACCCATTTCTTCTTTATTATAAACTACACCTTGATCTGATTTATTTTTTAATAA
AAAATTTTAAATATTTATAATTCTATTAAAATATTTAAACTACGACGATATATAAAC
TAATAAATAAAGTGCATCTAATCGTGGACTATCGATTATAGAACAGGTTCCTCTG
AAAAGACTAAAATACCGCCAAATTTTTTAATTTTCAAGAACATAACTAATACTAAT
TAAGTAAAAAAATTTACATTTTTAATAATAGGGTATCTAACAGACCTAATATTTCC
AGCTTCTGCACCAAAAATTAATTTTAATCCAACATCGAGGTCGCAATCTTTATCTT
CGATTAGAACTCTTTGATAATATTACGCTGTTATCCCTAAGGTAATTAAATCTTAC
AATTATTACTAATAGCTCAAGTATACATAAATTAATGATTTAAATTTAAAAAAAGTT
CGATTAATTTTTCAATCACCCCAATTAAATAATTAAAAAAATTAAAAATTTTTATAC
TaAAAAAAAATTAAAATTTTTAAATATGAAACTCTATAGGGTCTTCTCGTCTTTTAA
ATTAATCTTAGCTTTTTAACTAAAAAATAAATTTTTAATGTTTAAATAGAGACAGAA
ATTTTTTTATCCAACCATTCATTCCAGCTTCTAATTAAGAAACTAATGATTATGCTA
CCTTAGCACGGTCAAAATACCGCGGCCATTTAAATCCTCAGAGGGCAGGCTAAT
TATTAGACAAGAAAGGGGGAAAAAAGAAGCTTTTGGAACCTTAGGAATAATTTAT
GCCATGATAGCTATCGGATTATTAGGATTCGTAGTTTGAGCTCACCATATATTTA
CAGTAGGGATAGATGTAGATACCCGAGCTTACTTCACTTCAGCAACTATAATTAT
TGCGGTTCCAACTGGGATTAAAGTCTTTAGATGACTAGCCACCTTTCATGGAACT
CAAATTTTAAATAGACCAGTAACCCTGTGAGCTTTAGGTTTTGTATTTTTATTTAC
AGTAGGAGGATTAACAGGGGTAATTTTAGCTAATTCTTCAATTGATATTATTCTTC
ACGATACTTACTATGTAGTAGCTCATTTTCACTATGTTCTTTCTATAGGAGCTGTA
TTTGCAATTATAGCCGGAATTGTCCAATGATTCCCATTATTTACTGGACTAACCTT
AAATAATTTTTTTTTAAAAACCCAATTTATTACTATATTTTTAGGTGTAAATTTAACA
TTTTTTCCTCAACACTTTTTAGGGCTAAGAGGAATGCCTCGGCGATATTCGGATT
ATCCAGATGCTTTCACTATCTGAAATGCCATTTCATCTATTGGTTCCATTATTTCT
CTCACAAGAATTATCTTTTTTTTATTTATCTTATGAGAAAGGCTTTCTTCCCAACG
TAAATCTTTGAGAACTTTAAATGTAACAACTTCAATTGAATGGTTACAAGCCCTAC
CCCCTAGTG
ron3
CGGGCGATGTGTACATATTTTAGAGCTTTAATCATCTTAAAAAAATCTAAAAAATT
ACTCTCAAATCCAAATTCAAATTTTTTTAACAAAAATTATTTTAAAATAAATTTATT
GTAACCCATTTCTTCTTTATTATAAACTACACCTTGATCTGATTTATTTTTTAATAA
AAAATTTTAAATATTTATAATTCTATTAAAATATTTAAACTACGACGATATATAAAC
TAATAAATAAAGTGCATCTAATCGTGGACTATCGATTATAGAACAGGTTCCTCTG
AAAAGACTAAAATACCGCCAAATTTTTTAATTTTCAAGAACATAACTAATACTAAT
TAAGTAAAAAAATTTACATTTTTAATAATAGGGTATCTAACAGACCTAATATTTCC
AGCTTCTGCACCAAAAATTAATTTTAATCCAACATCGAGGTCGCAATCTTTATCTT
CGATTAGAACTCTTTGATAATATTACGCTGTTATCCCTAAGGTAATTAAATCTTAC
AATTATTACTAATAGCTCAAGTATACATAAATTAATGATTTAAATTTAAAAAAAGTT
CAATTAATTTTTCAATCACCCCAATTAAATAATTAAAAAAATTAAAAATTTTTATAC

TaAAAAAAAATTAAAATTTTTAAATATGAAACTCTATAGGGTCTTCTCGTCTTTTAA
ATTAATTTTAGCTTTTTAACTAAAAAATAAATTTTTAATGTTTAAATAGAGACAGAA
ATTTTTTTATCCAACCATTCATTCCAGCTTCTAATTAAGAAACTAATGATTATGCTA
CCTTAGCACGGTCAAAATACCGCGGCCATTTAAATCCTCAGAGGGCAGGCTAAT
TATTAGACAAGAAAGGGGGAAAAAAGAAGCTTTTGGAACCTTAGGAATAATTTAT
GCCATGATAGCTATCGGATTATTAGGATTCGTAGTTTGAGCTCACCATATATTTA
CAGTAGGGATAGATGTAGATACCCGAGCTTACTTCACTTCAGCAACTATAATTAT
TGCGGTTCCAACTGGGATTAAAGTCTTTAGATGACTAGCCACCTTTCATGGAACT
CAAATTTTAAATAGACCAGTAACCCTGTGAGCTTTAGGTTTTGTATTTTTATTTAC
AGTAGGAGGATTAACAGGGGTAATTTTAGCTAATTCTTCAATTGATATTATTCTTC
ACGATACTTACTATGTAGTAGCTCATTTTCACTATGTTCTTTCTATAGGAGCTGTA
TTTGCAATTATAGCCGGAATTGTCCAATGATTCCCATTATTTACCGGACTAACCT
TAAATAATTTTTTTTTAAAAACCCAATTTATTACTATATTTTTAGGTGTAAATTTAAC
ATTTTTTCCCCAACACTTTTTAGGGCTAAGAGGAATGCCTCGGCGATATTCGGAT
TATCCAGATGCTTTCACTATCTGAAATGCCATTTCATCTATTGGTTCCATTATTTC
TCTCACAAGAATTATCTTTTTTTTATTTATCTTATGAGAAAGGCTTTCTTCCCAAC
GTAAATCTTTGAGAACTTTAAATGTAACAACTTCAATTGAATGGTTACAAGCCCTA
CCCCCTAGTG
sob1
CGGGCGATGTGTACATATTTTAGAGCTTTAATCATCTTAAAAAAATCTAAAAAATT
ACTCTCAAATCCAAATTCAAATTTTTTTAACAAAAATTATTTTAAAATAAATTTATT
GTAACCCATTTCTTCTTTATTATAAACTACACCTTGATCTGATTTATTTTTTAATAA
AAAATTTTAAATATTTATAATTCTATTAAAATATTTAAACTACGACGATATATAAAC
TAATAAATAAAGTGCATCTAATCGTGGACTATCGATTATAGAACAGGTTCCTCTG
AAAAGACTAAAATACCGCCAAATTTTTTAATTTTCAAGAACATAACTAATACTAAT
TAAGTAAAAAAATTTACATTTTTAATAATAGGGTATCTAACAGACCTAATATTTCC
AGCTTCTGCACCAAAAATTAATTTTAATCCAACATCGAGGTCGCAATCTTTATCTT
CGATTAGAACTCTTTGATAATATTACGCTGTTATCCCTAAGGTAATTAAATCTTAC
AATTATTACTAATAGCTCAAGTATACATAAATTAATGATTTAAATTTAAAAAAAGTT
CGATTAATTTTTCAATCACCCCAATTAAATAATTAAAAAAATTAAAAATTTTTATAC
TaAAAAAAAATTAAAATTTTTAAATATGAAACTCTATAGGGTCTTCTCGTCTTTTAA
ATTAATCTTAGCTTTTTAACTAAAAAATAAATTTTTAATGTTTAAATAGAGACAGAA
ATTTTTTTATCCAACCATTCATTCCAGCTTCTAATTAAGAAACTAATGATTATGCTA
CCTTAGCACGGTCAAAATACCGCGGCCATTTAAATCCTCAGAGGGCAGGCTAAT
TATTAGACAAGAAAGGGGGAAAAAAGAAGCTTTTGGAACCTTAGGAATAATTTAT
GCCATGATAGCTATCGGATTATTAGGATTCGTAGTTTGAGCTCACCATATATTTA
CAGTAGGGATAGATGTAGATACCCGAGCTTACTTCACTTCAGCAACTATAATTAT
TGCGGTTCCAACTGGGATTAAAGTCTTTAGATGACTAGCCACCTTTCATGGAACT
CAAATTTTAAATAGACCAGTAACCCTGTGAGCTTTAGGTTTTGTATTTTTATTTAC
AGTAGGAGGATTAACGGGGGTAATTTTAGCTAATTCTTCAATTGATATTATTCTTC
ACGATACTTACTATGTAGTAGCTCATTTTCACTATGTTCTTTCTATAGGAGCTGTA
TTTGCAATTATAGCCGGAATTGTCCAATGATTCCCATTATTTACCGGACTAACCT
TAAATAATTTTTTTTTAAAAACCCAATTTATTACTATATTTTTAGGTGTAAATTTAAC
ATTTTTTCCCCAACACTTTTTAGGGCTAAGAGGAATGCCTCGGCGATATTCGGAT
TATCCAGATGCTTTCACTATCTGAAATGCCATTTCATCTATTGGTTCCATTATTTC
TCTCACAAGAATTATCTTTTTTTTATTTATCTTATGAGAAAGGCTTTCTTCCCAAC
GTAAATCTTTGAGAACTTTAAATGTAACAACTTCAATTGAATGGTTACAAGCCCTA
CCCCCTAGTG
SPA_PRA_1
CGGGCGATGTGTACATATTTTAGAGCTTTAATCATCTTAAAAAAATCTAAAAAATT

ACTCTCAAATCCAAATTCAAATTTTTTTAACAAAAATTATTTTAAAATAAATTTATT
GTAACCCATTTCTTCTTTATTATAAACTACACCTTGATCTGATTTATTTTTTAATAA
AAAATTTTAAATATTTATAATTCTATTAAAATATTTAAACTACGACGATATATAAAC
TAATAAATAAAGTGCATCTAATCGTGGACTATCGATTATAGAACAGGTTCCTCTG
AAAAGACTAAAATACCGCCAAATTTTTTAATTTTCAAGAACATAACTAATACTAAT
TAAGTAAAAAAATTTACATTTTTAATAATAGGGTATCTAACAGACCTAATATTTCC
AGCTTCTGCACCAAAAATTAATTTTAATCCAACATCGAGGTCGCAATCTTTATCTT
CGATTAGAACTCTTTGATAATATTACGCTGTTATCCCTAAGGTAATTAAATCTTAC
AATTATTACTAATAGCTCAAGTATACATAAATTAATGATTTAAATTTAAAAAAAGTT
CAATTAATTTTTCAATCACCCCAATTAAATAATTAAAAAAATTAAAAATTTTTATAC
TaAAAAAAAATTAAAATTTTTAAATATGAAACTCTATAGGGTCTTCTCGTCTTTTAA
ATTAATTTTAGCTTTTTAACTAAAAAATAAATTTTTAATGTTTAAATAGAGACAGAA
ATTTTTTTATCCAACCATTCATTCCAGCTTCTAATTAAGAAACTAATGATTATGCTA
CCTTAGCACGGTCAAAATACCGCGGCCATTTAAATCCTCAGAGGGCAGGCTAAT
TATTAGACAAGAAAGGGGGAAAAAAGAAGCTTTTGGAACCTTAGGAATAATTTAT
GCCATGATAGCTATCGGATTATTAGGATTCGTAGTTTGAGCTCACCATATATTTA
CAGTAGGGATAGATGTAGATACCCGAGCTTACTTCACTTCAGCAACTATAATTAT
TGCGGTTCCAACTGGGATTAAAGTCTTTAGATGACTAGCCACCTTTCATGGAACT
CAAATTTTAAATAGACCAGTAACCCTGTGAGCTTTAGGTTTTGTATTTTTATTTAC
AGTAGGAGGATTAACAGGGGTAATTTTAGCTAATTCTTCAATTGATATTATTCTTC
ACGATACTTACTATGTAGTAGCTCATTTTCACTATGTTCTTTCTATAGGAGCTGTA
TTTGCAATTATAGCCGGAATTGTCCAATGATTCCCATTATTTACCGGACTAACCT
TAAATAATTTTTTTTTAAAAACCCAATTTATTACTATATTTTTAGGTGTAAATTTAAC
ATTTTTTCCCCAACACTTTTTAGGGCTAAGAGGAATGCCTCGGCGATATTCGGAT
TATCCAGATGCTTTCACTATCTGAAATGCCATTTCATCTATTGGTTCCATTATTTC
TCTCACAAGAATTATCTTTTTTTTTATTTATCTTATGAGAAAGGCTTTCTTCCCAAC
GTAAATCTTTGAGAACTTTAAATGTAACAACTTCAATTGAATGGTTACAAGCCCTA
CCCCCTAGTG
SWI_NEU_1
CGGGCGATGTGTACATATTTTAGAGCTTTAATCATCTTAAAAAAATCTAAAAAATT
ACTCTCAAATCCAAATTCAAATTTTTTTAACAAAAATTATTTTAAAATAAATTTATT
GTAACCCATTTCTTCTTTATTATAAACTACACCTTGATCTGATTTATTTTTTAATAA
AAAATTTTAAATATTTATAATTCTATTAAAATATTTAAACTACGACGATATATAAAC
TAATAAATAAAGTGCATCTAATCGTGGACTATCGATTATAGAACAGGTTCCTCTG
AAAAGACTAAAATACCGCCAAATTTTTTAATTTTCAAGAACATAACTAATACTAAT
TAAGTAAAAAAATTTACATTTTTAATAATAGGGTATCTAACAGACCTAATATTTCC
AGCTTCTGCACCAAAAATTAATTTTAATCCAACATCGAGGTCGCAATCTTTATCTT
CGATTAGAACTCTTTGATAATATTACGCTGTTATCCCTAAGGTAATTAAATCTTAC
AATTATTACTAATAGCTCAAGTATACATAAATTAATGATTTAAATTTAAAAAAAGTT
CGATTAATTTTTCAATCACCCCAATTAAATAATTAAAAAAATTAAAAATTTTTATAC
TaAAAAAAAATTAAAATTTTTAAATATGAAACTCTATAGGGTCTTCTCGTCTTTTAA
ATTAATCTTAGCTTTTTAACTAAAAAATAAATTTTTAATGTTTAAATAGAGACAGAA
ATTTTTTTATCCAACCATTCATTCCAGCTTCTAATTAAGAAACTAATGATTATGCTA
CCTTAGCACGGTCAAAATACCGCGGCCATTTAAATCCTCAGAGGGCAGGCTAAT
TATTAGACAAGAAAGGGGGAAAAAAGAAGCTTTTGGAACCTTAGGAATAATTTAT
GCCATGATAGCTATCGGATTATTAGGATTCGTAGTTTGAGCTCACCATATATTTA
CAGTAGGGATAGATGTAGATACCCGAGCTTACTTCACTTCAGCAACTATGATTAT
CGCGGTTCCAACTGGGATTAAAGTCTTTAGGTGGCTAGCCACCTTTCATGGAAC
TCAAATTTTAAATAGACCAGTAACCCTGTGAGCTTTAGGTTTTGTATTTTTATTTA
CAGTAGGAGGATTAACAGGGGTAATTTTAGCTAATTCTTCAATTGATATTATTCTT

CACGATACTTACTATGTAGTAGCTCATTTTCACTATGTTCTTTCTATAGGAGCTGT
ATTTGCAATTATAGCCGGAATTGTCCAATGATTCCCATTATTTACTGGACTAACCT
TAAATAATTTTTTTTTAAAAACCCAATTTATTACTATATTTTTAGGTGTAAATTTAAC
ATTTTTTCCTCAACACTTTTTAGGGCTAAGAGGAATGCCTCGGCGATATTCGGAT
TATCCAGATGCTTTCACTATCTGAAATGCCATTTCATCTATTGGTTCCATTATTTC
TCTCACAAGAATTATCTTTTTTTTATTTATCTTATGAGAAAGGCTTTCTTCCCAAC
GTAAATCTTTGAGAACTTTAAATGTAACAACTTCAATTGAATGGTTACAAGCCCTA
CCCCCTAGTG
tol1
CGGGCGATGTGTACATATTTTAGAGCTTTAATCATCTTAAAAAAATCTAAAAAATT
ACTCTCAAATCCAAATTCAAATTTTTTTAACAAAAATTATTTTAAAATAAATTTATT
GTAACCCATTTCTTCTTTATTATAAACTACACCTTGATCTGATTTATTTTTTAATAA
AAAATTTTAAATATTTATAATTCTATTAAAATATTTAAACTACGACGATATATAAAC
TAATAAATAAAGTGCATCTAATCGTGGACTATCGATTATAGAACAGGTTCCTCTG
AAAAGACTAAAATACCGCCAAATTTTTTAATTTTCAAGAACATAACTAATACTAAT
TAAGTAAAAAAATTTACATTTTTAATAATAGGGTATCTAACAGACCTAATATTTCC
AGCTTCTGCACCAAAAATTAATTTTAATCCAACATCGAGGTCGCAATCTTTATCTT
CGATTAGAACTCTTTGATAATATTACGCTGTTATCCCTAAGGTAATTAAATCTTAC
AATTATTACTAATAGCTCAAGTATACATAAATTAATGATTTAAATTTAAAAAAAGTT
CAATTAATTTTTCAATCACCCCAATTAAATAATTAAAAAAATTAAAAATTTTTATAC
TaAAAAAAAATTAAAATTTTTAAATATGAAACTCTATAGGGTCTTCTCGTCTTTTAA
ATTAATTTTAGCTTTTTAACTAAAAAATAAATTTTTAATGTTTAAATAGAGACAGAA
ATTTTTTTATCCAACCATTCATTCCAGCTTCTAATTAAGAAACTAATGATTATGCTA
CCTTAGCACGGTCAAAATACCGCGGCCATTTAAATCCTCAGAGGGCAGGCTAAT
TATTAGACAAGAAAGGGGGAAAAAAGAAGCTTTTGGAGCCTTAGGAATAATTTAT
GCCATGATAGCTATCGGATTATTAGGATTCGTAGTTTGAGCTCACCATATATTTA
CAGTAGGGATAGATGTAGATACCCGAGCTTACTTCACTTCAGCAACTATAATTAT
TGCGGTTCCAACTGGGATTAAAGTCTTTAGATGACTAGCCACCTTTCATGGAACT
CAAATTTTAAATAGACCAGTAACCCTGTGAGCTTTAGGTTTTGTATTTTTATTTAC
AGTAGGAGGATTAACAGGGGTAATTTTAGCTAATTCTTCAATTGATATTATTCTTC
ACGATACTTACTATGTAGTAGCTCATTTTCACTATGTTCTTTCTATAGGAGCTGTA
TTTGCAATTATAGCCGGAATTGTCCAATGATTCCCATTATTTACCGGACTAACCT
TAAATAATTTTTTTTTAAAAACCCAATTTATTACTATATTTTTAGGTGTAAATTTAAC
ATTTTTTCCCCAACACTTTTTAGGGCTAAGAGGAATGCCTCGGCGATATTCGGAT
TATCCAGATGCTTTCACTATCTGAAATGCCATTTCATCTATTGGTTCCATTATTTC
TCTCACAAGAATTATCTTTTTTTTATTTATCTTATGAGAAAGGCTTTCTTCCCAAC
GTAAATCTTTGAGAACTTTAAATGTAACAACTTCAATTGAATGGTTACAAGCCCTA
CCCCCTAGTG
[12s
CGGGCGATGTGTACATATTTTAGAGCTTTAATCATCTTAAAAAAATCTAAAAAATT
ACTCTCAAATCCAAATTCAAATTTTTTTAACAAAAATTATTTTCAAATAAATTTATT
GTAACCCATTTCTTCTTTATTATAAACTACACCTTGATCTGATTTATTTTTTAATAA
AAAATTTTAAATATTTATAATTCTATTAAAATATTTAAACTACGACGATATATAAAC
TAATAAATAAAGTGCATCTAATCGTGGACTATCGATTATAGAACAGGTTCCTCTG
AAAAGACTAAAATACCGCCAAATTTTTTAATTTTCAAGAACATAACTAATACTAAT
TAAGTAAAAAAATTTACATTTTTAATAATAGGGTATCTAA]
[16s
CAGACCTAATATTTCCAGCTTCTGCACCAAAAATTAATTTTAATCCAACATCGAG
GTCGCAATCTTTATCTTCGATTAGAACTCTTTGATAATATTACGCTGTTATCCCTA
AGGTAATTAAATCTTACAATTATTACTAATAGCTCAAGTATACATAAATTAATGATT

```
TAAATTTAAAAAAAGTTCGATTAATTTTTCAATCACCCCAATTAAATAATTAAAAAA
ATTAAAAATTTTTATACTaAAAAAAAATTAAAATTTTTAAATATGAAACTCTATAGG
GTCTTCTCGTCTTTTAAATTAATCTTAGCTTTTTAACTAAAAAATAAATTTTTAATG
TTTAAATAGAGACAGAAATTTTTTTATCCAACCATTCATTCCAGCTTCTAATTAAG
AAACTAATGATTATGCTACCTTAGCACGGTCAAAATACCGCGGCCATTTAAATCC
TCAGAGGGCAGGCTA]
[CO1
ATTATTAGACAAGAAAGGGGGAAAAAAGAAGCTTTTGGAACCTTAGGAATAATTT
ATGCCATGATAGCTATCGGATTATTAGGATTCGTAGTTTGAGCTCACCATATATT
TACAGTAGGGATAGATGTAGATACCCGAGCTTACTTCACTTCAGCAACTATAATT
ATTGCGGTTCCAACTGGGATTAAAGTCTTTAGATGACTAGCCACCTTTCATGGAA
CTCAAATTTTAAATAGACCAGTAACCCTGTGAGCTTTAGGTTTTGTATTTTTATTT
ACAGTAGGAGGATTAACAGGGGTAATTTTAGCTAATTCTTCAATTGATATTATTCT
TCACGATACTTACTATGTAGTAGCTCATTTTCACTATGTTCTTTCTATAGGAGCTG
TATTTGCAATTATAGCCGGAATTGTCCAATGATTCCCATTATTTACCGGACTAAC
CTTAAATAATTTTTTTTTAAAAACCCAATTTATTACTATATTTTTAGGTGTAAATTTA
ACATTTTTTCCCCAACACTTTTTAGGGCTAAGAGGAATGCCTCGGCGATATTCGG
ATTATCCAGATGCTTTCACTATCTGAAATGCCATTTCATCTATTGGTTCCATTATT
TCTCTCACAAGAATTATCTTTTTTTTATTTATCTTATGAGAAAGGCTTTCTTCCCAA
CGTAAATCTTTGAGAACTTTAAATGTAACAACTTCAATTGAATGGTTACAAGCCCT
ACCCCCTAGTG]
;

endblock;


BEGIN MRBAYES;

	charset 12S = 1-378;
	charset 16S = 379-843;
	charset CO1 = 844-1579;
	partition favored = 3: 12S, 16S, CO1;
	set partition = favored;

	lset applyto=(1) nst=1 rates=equal;
	lset applyto=(2) nst=2 rates=equal;
	lset applyto=(3) nst=2 rates=propinv;
	unlink statefreq=(all) revmat=(all) pinvar=(all);
	prset applyto=(all) ratepr=variable;
	mcmc ngen=1000000 samplefreq=1000 printfreq=1000 diagnfreq=10000;


END;
```

datasetS5

#NEXUS

begin data;
dimensions ntax=191 nchar=732;
format datatype=dna gap=- interleave=no;
matrix

AFR10
ATTATTAGACAAGAAAGGGGGAAAAAAGAAGCTTTTGGACCTTAGGAATAATTTA
TGCCATGATAGCTATCGGATTATTAGGATTCGTAGTTTGAGCTCACCATATATTT
ACAGTAGATAGATGTAGATACCCGAGCTTACTTCACTTCAGCAACTATAATTATT
GCGGTTCCAACTGGGATTAAAGTCTTTAGATGACTAGCCACCTTTCATGGAACT
CAAATTTTAAATAGACCAGTAACCCTGTGAGCTTTAGGTTTTGTATTTTTATTTAC
AGTAGGAGGATTAACAGGGGTAATTTTAGCTAATTCTTCAATTGATATTATTCTTC
ACGATACTTACTATGTAGTAGCTCATTTTCACTATGTTCTTTCTATAGGAGCTGTA
TTTGCAATTATAGCCGGAATTGTCCAATGATTCCCATTATTTACCGGACTAACCT
TAAATAATTTTTTTTTAAAAACCCAATTTATTACTATATTTTTAGGTGTAAATTTAAC
ATTTTTTCCCCAACACTTTTTAGGGCTAAGAGGAATGCCTCGGCGATATTCGGAT
TATCCAGATGCTTTCACTATCTGAAATGCCATTTCATCTATTGGTTCCATTATTTC
TCTCACAAGAATTATCTTTTTTTTATTTATCTTATGAGAAAGGCTTTCTTCCCAAC
GTAAATCTTTGAGAACTTTAAATGTAACAACTTCAATTGAATGGTTCAAGCCCTAC
CCCCTAGTG
AFR11
ATTATTAGACAAGAAAGGGGGAAAAAAGAAGCTTTTGGACCTTAGGAATAATTTA
TGCCATGATAGCTATCGGATTATTAGGATTCGTAGTTTGAGCTCACCATATATTT
ACAGTAGATAGATGTAGATACCCGAGCTTACTTCACTTCAGCAACTATAATTATT
GCGGTTCCAACTGGGATTAAAGTCTTTAGATGACTAGCCACCTTTCATGGAACT
CAAATTTTAAATAGACCAGTAACCCTGTGAGCTTTAGGTTTTGTATTTTTATTTAC
AGTAGGAGGATTAACAGGGGTAATTTTAGCTAATTCTTCAATTGATATTATTCTTC
ACGATACTTACTATGTAGTAGCTCATTTTCACTATGTTCTTTCTATAGGAGCTGTA
TTTGCAATTATAGCCGGAATTGTCCAATGATTCCCATTATTTACCGGACTAACCT
TAAATAATTTTTTTTTAAAAACCCAATTTATTACTATATTTTTAGGTGTAAATTTAAC
ATTTTTTCCCCAACACTTTTTAGGGCTAAGAGGAATGCCTCGGCGATATTCGGAT
TATCCAGATGCTTTCACTATCTGAAATGCCATTTCATCTATTGGTTCCATTATTTC
TCTCACAAGAATTATCTTTTTTTTATTTATCTTATGAGAAAGGCTTTCTTCCCAAC
GTAAATCTTTGAGAACTTTAAATGTAACAACTTCAATTGAATGGTTCAAGCCCTAC
CCCCTAGTG
AFR12
ATTATTAGACAAGAAAGGGGGAAAAAAGAAGCTTTTGGACCTTAGGAATAATTTA
TGCCATGATAGCTATCGGATTATTAGGATTCGTAGTTTGAGCTCACCATATATTT
ACAGTAGATAGATGTAGATACCCGAGCTTACTTCACTTCAGCAACTATAATTATT
GCGGTTCCAACTGGGATTAAAGTCTTTAGATGACTAGCCACCTTTCATGGAACT
CAAATTTTAAATAGACCAGTAACCCTGTGAGCTTTAGGTTTTGTATTTTTATTTAC
AGTAGGAGGATTAACAGGGGTAATTTTAGCTAATTCTTCAATTGATATTATTCTTC
ACGATACTTACTATGTAGTAGCTCATTTTCACTATGTTCTTTCTATAGGAGCTGTA
TTTGCAATTATAGCCGGAATTGTCCAATGATTCCCATTATTTACCGGACTAACCT
TAAATAATTTTTTTTTAAAAACCCAATTTATTACTATATTTTTAGGTGTAAATTTAAC
ATTTTTTCCCCAACACTTTTTAGGGCTAAGAGGAATGCCTCGGCGATATTCGGAT
TATCCAGATGCTTTCACTATCTGAAATGCCATTTCATCTATTGGTTCCATTATTTC

TCTCACAAGAATTATCTTTTTTTTATTTATCTTATGAGAAAGGCTTTCTTCCCAAC
GTAAATCTTTGAGAACTTTAAATGTAACAACTTCAATTGAATGGTTCAAGCCCTAC
CCCCTAGTG
AFR18
ATTATTAGACAAGAAAGGGGGAAAAAAGAAGCTTTTGGACCTTAGGAATAATTTA
TGCCATGATAGCTATCGGATTATTAGGATTCGTAGTTTGAGCTCACCATATATTT
ACAGTAGATAGATGTAGATACCCGAGCTTACTTCACTTCAGCAACTATAATTATT
GCGGTTCCAACTGGGATTAAAGTCTTTAGATGACTAGCCACCTTTCATGGAACT
CAAATTTTAAATAGACCAGTAACCCTGTGAGCTTTAGGTTTTGTATTTTTATTTAC
AGTAGGAGGATTAACAGGGGTAATTTTAGCTAATTCTTCAATTGATATTATTCTTC
ACGATACTTACTATGTAGTAGCTCATTTTCACTATGTTCTTTCTATAGGAGCTGTA
TTTGCAATTATAGCCGGAATTGTCCAATGATTCCCATTATTTACCGGACTAACCT
TAAATAATTTTTTTTTAAAAACCCAATTTATTACTATATTTTTAGGTGTAAATTTAAC
ATTTTTTCCCCAACACTTTTTAGGGCTAAGAGGAATGCCTCGGCGATATTCGGAT
TATCCAGATGCTTTCACTATCTGAAATGCCATTTCATCTATTGGTTCCATTATTTC
TCTCACAAGAATTATCTTTTTTTTATTTATCTTATGAGAAAGGCTTTCTTCCCAAC
GTAAATCTTTGAGAACTTTAAATGTAACAACTTCAATTGAATGGTTCAAGCCCTAC
CCCCTAGTG
afr19
ATTATTAGACAAGAAAGGGGGAAAAAAGAAGCTTTTGGACCTTAGGAATAATTTA
TGCCATGATAGCTATCGGATTATTAGGATTCGTAGTTTGAGCTCACCATATATTT
ACAGTAGATAGATGTAGATACCCGAGCTTACTTCACTTCAGCAACTATAATTATT
GCGGTTCCAACTGGGATTAAAGTCTTTAGATGACTAGCCACCTTTCATGGAACT
CAAATTTTAAATAGACCAGTAACCCTGTGAGCTTTAGGTTTTGTATTTTTATTTAC
AGTAGGAGGATTAACAGGGGTAATTTTAGCTAATTCTTCAATTGATATTATTCTTC
ACGATACTTACTATGTAGTAGCTCATTTTCACTATGTTCTTTCTATAGGAGCTGTA
TTTGCAATTATAGCCGGAATTGTCCAATGATTCCCATTATTTACCGGACTAACCT
TAAATAATTTTTTTTTAAAAACCCAATTTATTACTATATTTTTAGGTGTAAATTTAAC
ATTTTTTCCCCAACACTTTTTAGGGCTAAGAGGAATGCCTCGGCGATATTCGGAT
TATCCAGATGCTTTCACTATCTGAAATGCCATTTCATCTATTGGTTCCATTATTTC
TCTCACAAGAATTATCTTTTTTTTATTTATCTTATGAGAAAGGCTTTCTTCCCAAC
GTAAATCTTTGAGAACTTTAAATGTAACAACTTCAATTGAATGGTTCAAGCCCTAC
CCCCTAGTG
AFR2
ATTATTAGACAAGAAAGGGGGAAAAAAGAAGCTTTTGGACCTTAGGAATAATTTA
TGCCATGATAGCTATCGGATTATTAGGATTCGTAGTTTGAGCTCACCATATATTT
ACAGTAGATAGATGTAGATACCCGAGCTTACTTCACTTCAGCAACTATAATTATT
GCGGTTCCAACTGGGATTAAAGTCTTTAGATGACTAGCCACCTTTCATGGAACT
CAAATTTTAAATAGACCAGTAACCCTGTGAGCTTTAGGTTTTGTATTTTTATTTAC
AGTAGGAGGATTAACAGGGGTAATTTTAGCTAATTCTTCAATTGATATTATTCTTC
ACGATACTTACTATGTAGTAGCTCATTTTCACTATGTTCTTTCTATAGGAGCTGTA
TTTGCAATTATAGCCGGAATTGTCCAATGATTCCCATTATTTACCGGACTAACCT
TAAATAATTTTTTTTTAAAAACCCAATTTATTACTATATTTTTAGGTGTAAATTTAAC
ATTTTTTCCCCAACACTTTTTAGGGCTAAGAGGAATGCCTCGGCGATATTCGGAT
TATCCAGATGCTTTCACTATCTGAAATGCCATTTCATCTATTGGTTCCATTATTTC
TCTCACAAGAATTATCTTTTTTTTATTTATCTTATGAGAAAGGCTTTCTTCCCAAC
GTAAATCTTTGAGAACTTTAAATGTAACAACTTCAATTGAATGGTTCAAGCCCTAC
CCCCTAGTG
AFR3
ATTATTAGACAAGAAAGGGGGAAAAAAGAAGCTTTTGGACCTTAGGAATAATTTA

TGCCATGATAGCTATCGGATTATTAGGATTCGTAGTTTGAGCTCACCATATATTT
ACAGTAGATAGATGTAGATACCCGAGCTTACTTCACTTCAGCAACTATAATTATT
GCGGTTCCAACTGGGATTAAAGTCTTTAGATGACTAGCCACCTTTCATGGAACT
CAAATTTTAAATAGACCAGTAACCCTGTGAGCTTTAGGTTTTGTATTTTTATTTAC
AGTAGGAGGATTAACAGGGGTAATTTTAGCTAATTCTTCAATTGATATTATTCTTC
ACGATACTTACTATGTAGTAGCTCATTTTCACTATGTTCTTTCTATAGGAGCTGTA
TTTGCAATTATAGCCGGAATTGTCCAATGATTCCCATTATTTACCGGACTAACCT
TAAATAATTTTTTTTTAAAAACCCAATTTATTACTATATTTTTAGGTGTAAATTTAAC
ATTTTTTCCCCAACACTTTTTAGGGCTAAGAGGAATGCCTCGGCGATATTCGGAT
TATCCAGATGCTTTCACTATCTGAAATGCCATTTCATCTATTGGTTCCATTATTTC
TCTCACAAGAATTATCTTTTTTTTATTTATCTTATGAGAAAGGCTTTCTTCCCAAC
GTAAATCTTTGAGAACTTTAAATGTAACAACTTCAATTGAATGGTTCAAGCCCTAC
CCCCTAGTG
AFR5
ATTATTAGACAAGAAAGGGGGAAAAAAGAAGCTTTTGGACCTTAGGAATAATTTA
TGCCATGATAGCTATCGGATTATTAGGATTCGTAGTTTGAGCTCACCATATATTT
ACAGTAGATAGATGTAGATACCCGAGCTTACTTCACTTCAGCAACTATAATTATT
GCGGTTCCAACTGGGATTAAAGTCTTTAGATGACTAGCCACCTTTCATGGAACT
CAAATTTTAAATAGACCAGTAACCCTGTGAGCTTTAGGTTTTGTATTTTTATTTAC
AGTAGGAGGATTAACAGGGGTAATTTTAGCTAATTCTTCAATTGATATTATTCTTC
ACGATACTTACTATGTAGTAGCTCATTTTCACTATGTTCTTTCTATAGGAGCTGTA
TTTGCAATTATAGCCGGAATTGTCCAATGATTCCCATTATTTACCGGACTAACCT
TAAATAATTTTTTTTTAAAAACCCAATTTATTACTATATTTTTAGGTGTAAATTTAAC
ATTTTTTCCCCAACACTTTTTAGGGCTAAGAGGAATGCCTCGGCGATATTCGGAT
TATCCAGATGCTTTCACTATCTGAAATGCCATTTCATCTATTGGTTCCATTATTTC
TCTCACAAGAATTATCTTTTTTTTATTTATCTTATGAGAAAGGCTTTCTTCCCAAC
GTAAATCTTTGAGAACTTTAAATGTAACAACTTCAATTGAATGGTTCAAGCCCTAC
CCCCTAGTG
afr9
ATTATTAGACAAGAAAGGGGGAAAAAAGAAGCTTTTGGACCTTAGGAATAATTTA
TGCCATGATAGCTATCGGATTATTAGGATTCGTAGTTTGAGCTCACCATATATTT
ACAGTAGATAGATGTAGATACCCGAGCTTACTTCACTTCAGCAACTATAATTATC
GCGGTTCCAACTGGGATTAAAGTCTTTAGGTGACTAGCCACCTTTCATGGAACT
CAAATTTTAAATAGACCAGTAACCCTGTGAGCTTTAGGTTTTGTATTTTTATTTAC
AGTAGGAGGATTAACAGGGGTAATTTTAGCTAATTCTTCAATTGATATTATTCTTC
ACGATACTTACTATGTAGTAGCTCATTTTCACTATGTTCTTTCTATAGGAGCTGTA
TTTGCAATTATAGCCGGAATTGTCCAATGATTCCCATTATTTACTGGACTAACCTT
AAATAATTTTTTTTTAAAAACCCAATTTATTACTATATTTTTAGGTGTAAATTTAACA
TTTTTTCCTCAACACTTTTTAGGGCTAAGAGGAATGCCTCGGCGATATTCGGATT
ATCCAGATGCTTTCACTATCTGAAATGCCATTTCATCTATTGGTTCCATTATTTCT
CTCACAAGAATTATCTTTTTTTTATTTATCTTATGAGAAAGGCTTTCTTCCCAACG
TAAATCTTTGAGAACTTTAAATGTAACAACTTCAATTGAATGGTTCAAGCCCTACC
CCCTAGTG
AQU10
ATTATTAGACAAGAAAGGGGGAAAAAAGAAGCTTTTGGACCTTAGGAATAATTTA
TGCCATGATAGCTATCGGATTATTAGGATTCGTAGTTTGAGCTCACCATATATTT
ACAGTAGATAGATGTAGATACCCGAGCTTACTTCACTTCAGCAACTATAATTATT
GCGGTTCCAACTGGGATTAAAGTCTTTAGATGACTAGCCACCTTTCATGGAACT
CAAATTTTAAATAGACCAGTAACCCTGTGAGCTTTAGGTTTTGTATTTTTATTTAC
AGTAGGAGGATTAACAGGGGTAATTTTAGCTAATTCTTCAATTGATATTATTCTTC

ACGATACTTACTATGTAGTAGCTCATTTTCACTATGTTCTTTCTATAGGAGCTGTA
TTTGCAATTATAGCCGGAATTGTCCAATGATTCCCATTATTTACCGGACTAACCT
TAAATAATTTTTTTTTAAAAACCCAATTTATTACTATATTTTTAGGTGTAAATTTAAC
ATTTTTTCCCCAACACTTTTTAGGGCTAAGAGGAATGCCTCGGCGATATTCGGAT
TATCCAGATGCTTTCACTATCTGAAATGCCATTTCATCTATTGGTTCCATTATTTC
TCTCACAAGAATTATCTTTTTTTTTATTTATCTTATGAGAAAGGCTTTCTTCCCAAC
GTAAATCTTTGAGAACTTTAAATGTAACAACTTCAATTGAATGGTTCAAGCCCTAC
CCCCTAGTG
AQU12
ATTATTAGACAAGAAAGGGGGAAAAAAGAAGCTTTTGGACCTTAGGAATAATTTA
TGCCATGATAGCTATCGGATTATTAGGATTCGTAGTCTGAGCTCACCATATATTT
ACAGTAGATAGATGTAGATACCCGAGCTTACTTCACTTCAGCAACTATAATTATT
GCGGTTCCAACTGGGATTAAAGTCTTTAGATGACTAGCCACCTTTCATGGAACT
CAAATTTTAAATAGACCAGTAACCCTGTGAGCTTTAGGTTTTGTATTTTTATTTAC
AGTAGGAGGATTAACAGGGGTAATTTTAGCTAATTCTTCAATTGATATTATTCTTC
ACGATACTTACTATGTAGTAGCTCATTTTCACTATGTTCTTTCTATAGGAGCTGTA
TTTGCAATTATAGCCGGAATTGTCCAATGATTCCCATTATTTACCGGACTAACCT
TAAATAATTTTTTTTTAAAAACCCAATTTATTACTATATTTTTAGGTGTAAATTTAAC
ATTTTTTCCCCAACACTTTTTAGGGCTAAGAGGAATGCCTCGGCGATATTCGGAT
TATCCAGATGCTTTCACTATCTGAAATGCCATTTCATCTATTGGTTCCATTATTTC
TCTCACAAGAATTATCTTTTTTTTTATTTATCTTATGAGAAAGGCTTTCTTCCCAAC
GTAAATCTTTGAGAACTTTAAATGTAACAACTTCAATTGAATGGTTCAAGCCCTAC
CCCCTAGTG
AQU13
ATTATTAGACAAGAAAGGGGGAAAAAAGAAGCTTTTGGACCTTAGGAATAATTTA
TGCCATGATAGCTATCGGATTATTAGGATTCGTAGTTTGAGCTCACCATATATTT
ACAGTAGATAGATGTAGATACCCGAGCTTACTTCACTTCAGCAACTATAATTATT
GCGGTTCCAACTGGGATTAAAGTCTTTAGATGACTAGCCACCTTTCATGGAACT
CAAATTTTAAATAGACCAGTAACCCTGTGAGCTTTAGGTTTTGTATTTTTATTTAC
AGTAGGAGGATTAACAGGGGTAATTTTAGCTAATTCTTCAATTGATATTATTCTTC
ACGATACTTACTATGTAGTAGCTCATTTTCACTATGTTCTTTCTATAGGAGCTGTA
TTTGCAATTATAGCCGGAATTGTCCAATGATTCCCATTATTTACCGGACTAACCT
TAAATAATTTTTTTTTAAAAACCCAATTTATTACTATATTTTTAGGTGTAAATTTAAC
ATTTTTTCCCCAACACTTTTTAGGGCTAAGAGGAATGCCTCGGCGATATTCGGAT
TATCCAGATGCTTTCACTATCTGAAATGCCATTTCATCTATTGGTTCCATTATTTC
TCTCACAAGAATTATCTTTTTTTTTATTTATCTTATGAGAAAGGCTTTCTTCCCAAC
GTAAATCTTTGAGAACTTTAAATGTAACAACTTCAATTGAATGGTTCAAGCCCTAC
CCCCTAGTG
AQU14
ATTATTAGACAAGAAAGGGGGAAAAAAGAAGCTTTTGGACCTTAGGAATAATTTA
TGCCATGATAGCTATCGGATTATTAGGATTCGTAGTTTGAGCTCACCATATATTT
ACAGTAGATAGATGTAGATACCCGAGCTTACTTCACTTCAGCAACTATAATTATT
GCGGTTCCAACTGGGATTAAAGTCTTTAGATGACTAGCCACCTTTCATGGAACT
CAAATTTTAAATAGACCAGTAACCCTGTGAGCTTTAGGTTTTGTATTTTTATTTAC
AGTAGGAGGATTAACAGGGGTAATTTTAGCTAATTCTTCAATTGATATTATTCTTC
ACGATACTTACTATGTAGTAGCTCATTTTCACTATGTTCTTTCTATAGGAGCTGTA
TTTGCAATTATAGCCGGAATTGTCCAATGATTCCCATTATTTACCGGACTAACCT
TAAATAATTTTTTTTTAAAAACCCAATTTATTACTATATTTTTAGGTGTAAATTTAAC
ATTTTTTCCCCAACACTTTTTAGGGCTAAGAGGAATGCCTCGGCGATATTCGGAT
TATCCAGATGCTTTCACTATCTGAAATGCCATTTCATCTATTGGTTCCATTATTTC

TCTCACAAGAATTATCTTTTTTTTATTTATCTTATGAGAAAGGCTTTCTTCCCAAC
GTAAATCTTTGAGAACTTTAAATGTAACAACTTCAATTGAATGGTTCAAGCCCTAC
CCCCTAGTG
AQU4
ATTATTAGACAAGAAAGGGGGAAAAAAGAAGCTTTTGGACCTTAGGAATAATTTA
TGCCATGATAGCTATCGGATTATTAGGATTCGTAGTCTGAGCTCACCATATATTT
ACAGTAGATAGATGTAGATACCCGAGCTTACTTCACTTCAGCAACTATAATTATT
GCGGTTCCAACTGGGATTAAAGTCTTTAGATGACTAGCCACCTTTCATGGAACT
CAAATTTTAAATAGACCAGTAACCCTGTGAGCTTTAGGTTTTGTATTTTTATTTAC
AGTAGGAGGATTAACAGGGGTAATTTTAGCTAATTCTTCAATTGATATTATTCTTC
ACGATACTTACTATGTAGTAGCTCATTTTCACTATGTTCTTTCTATAGGAGCTGTA
TTTGCAATTATAGCCGGAATTGTCCAATGATTCCCATTATTTACCGGACTAACCT
TAAATAATTTTTTTTTAAAAACCCAATTTATTACTATATTTTTAGGTGTAAATTTAAC
ATTTTTTCCCCAACACTTTTTAGGGCTAAGAGGAATGCCTCGGCGATATTCGGAT
TATCCAGATGCTTTCACTATCTGAAATGCCATTTCATCTATTGGTTCCATTATTTC
TCTCACAAGAATTATCTTTTTTTTATTTATCTTATGAGAAAGGCTTTCTTCCCAAC
GTAAATCTTTGAGAACTTTAAATGTAACAACTTCAATTGAATGGTTCAAGCCCTAC
CCCCTAGTG
bja1
ATTATTAGACAAGAAAGGGGGAAAAAAGAAGCTTTTGGACCTTAGGAATAATTTA
TGCCATGATAGCTATCGGATTATTAGGATTCGTAGTTTGAGCTCACCATATATTT
ACAGTAGATAGATGTAGATACCCGAGCTTACTTCACTTCAGCAACTATAATTATT
GCGGTTCCAACTGGGATTAAAGTCTTTAGATGACTAGCCACCTTTCATGGAACT
CAAATTTTAAATAGACCAGTAACCCTGTGAGCTTTAGGTTTTGTATTTTTATTTAC
AGTAGGAGGATTAACAGGGGTAATTTTAGCTAATTCTTCAATTGATATTATTCTTC
ACGATACTTACTATGTAGTAGCTCATTTTCACTATGTTCTTTCTATAGGAGCTGTA
TTTGCAATTATAGCCGGAATTGTCCAATGATTCCCATTATTTACCGGACTAACCT
TAAATAATTTTTTTTTAAAAACCCAATTTATTACTATATTTTTAGGTGTAAATTTAAC
ATTTTTTCCCCAACACTTTTTAGGGCTAAGAGGAATGCCTCGGCGATATTCGGAT
TATCCAGATGCTTTCACTATCTGAAATGCCATTTCATCTATTGGTTCCATTATTTC
TCTCACAAGAATTATCTTTTTTTTATTTATCTTATGAGAAAGGCTTTCTTCCCAAC
GTAAATCTTTGAGAACTTTAAATGTAACAACTTCAATTGAATGGTTCAAGCCCTAC
CCCCTAGTG
BJA2
ATTATTAGACAAGAAAGGGGGAAAAAAGAAGCTTTTGGACCTTAGGAATAATTTA
TGCCATGATAGCTATCGGATTATTAGGATTCGTAGTTTGAGCTCACCATATATTT
ACAGTAGATAGATGTAGATACCCGAGCTTACTTCACTTCAGCAACTATAATTATT
GCGGTTCCAACTGGGATTAAAGTCTTTAGATGACTAGCCACCTTTCATGGAACT
CAAATTTTAAATAGACCAGTAACCCTGTGAGCTTTAGGTTTTGTATTTTTATTTAC
AGTAGGAGGATTAACAGGGGTAATTTTAGCTAATTCTTCAATTGATATTATTCTTC
ACGATACTTACTATGTAGTAGCTCATTTTCACTATGTTCTTTCTATAGGAGCTGTA
TTTGCAATTATAGCCGGAATTGTCCAATGATTCCCATTATTTACCGGACTAACCT
TAAATAATTTTTTTTTAAAAACCCAATTTATTACTATATTTTTAGGTGTAAATTTAAC
ATTTTTTCCCCAACACTTTTTAGGGCTAAGAGGAATGCCTCGGCGATATTCGGAT
TATCCAGATGCTTTCACTATCTGAAATGCCATTTCATCTATTGGTTCCATTATTTC
TCTCACAAGAATTATCTTTTTTTTATTTATCTTATGAGAAAGGCTTTCTTCCCAAC
GTAAATCTTTGAGAACTTTAAATGTAACAACTTCAATTGAATGGTTCAAGCCCTAC
CCCCTAGTG
BJA4
ATTATTAGACAAGAAAGGGGGAAAAAAGAAGCTTTTGGACCTTAGGAATAATTTA

TGCCATGATAGCTATCGGATTATTAGGATTCGTAGTTTGAGCTCACCATATATTT
ACAGTAGATAGATGTAGATACCCGAGCTTACTTCACTTCAGCAACTATAATTATT
GCGGTTCCAACTGGGATTAAAGTCTTTAGATGACTAGCCACCTTTCATGGAACT
CAAATTTTAAATAGACCAGTAACCCTGTGAGCTTTAGGTTTTGTATTTTTATTTAC
AGTAGGAGGATTAACAGGGGTAATTTTAGCTAATTCTTCAATTGATATTATTCTTC
ACGATACTTACTATGTAGTAGCTCATTTTCACTATGTTCTTTCTATAGGAGCTGTA
TTTGCAATTATAGCCGGAATTGTCCAATGATTCCCATTATTTACCGGACTAACCT
TAAATAATTTTTTTTTAAAAACCCAATTTATTACTATATTTTTAGGTGTAAATTTAAC
ATTTTTTCCCCAACACTTTTTAGGGCTAAGAGGAATGCCTCGGCGATATTCGGAT
TATCCAGATGCTTTCACTATCTGAAATGCCATTTCATCTATTGGTTCCATTATTTC
TCTCACAAGAATTATCTTTTTTTTATTTATCTTATGAGAAAGGCTTTCTTCCCAAC
GTAAATCTTTGAGAACTTTAAATGTAACAACTTCAATTGAATGGTTCAAGCCCTAC
CCCCTAGTG
CAM1
ATTATTAGACAAGAAAGGGGGAAAAAAGAAGCTTTTGGACCTTAGGAATAATTTA
TGCCATGATAGCTATCGGATTATTAGGATTCGTAGTTTGAGCTCACCATATATTT
ACAGTAGATAGATGTAGATACCCGAGCTTACTTCACTTCAGCAACTATAATTATT
GCGGTTCCAACTGGGATTAAAGTCTTTAGATGACTAGCCACCTTTCATGGAACT
CAAATTTTAAATAGACCAGTAACCCTGTGAGCTTTAGGTTTTGTATTTTTATTTAC
AGTAGGAGGATTAACAGGGGTAATTTTAGCTAATTCTTCAATTGATATTATTCTTC
ACGATACTTACTATGTAGTAGCTCATTTTCACTATGTTCTTTCTATAGGAGCTGTA
TTTGCAATTATAGCCGGAATTGTCCAATGATTCCCATTATTTACCGGACTAACCT
TAAATAATTTTTTTTTAAAAACCCAATTTATTACTATATTTTTAGGTGTAAATTTAAC
ATTTTTTCCCCAACACTTTTTAGGGCTAAGAGGAATGCCTCGGCGATATTCGGAT
TATCCAGATGCTTTCACTATCTGAAATGCCATTTCATCTATTGGTTCCATTATTTC
TCTCACAAGAATTATCTTTTTTTTATTTATCTTATGAGAAAGGCTTTCTTCCCAAC
GTAAATCTTTGAGAACTTTAAATGTAACAACTTCAATTGAATGGTTCAAGCCCTAC
CCCCTAGTG
CAM2
ATTATTAGACAAGAAAGGGGGAAAAAAGAAGCTTTTGGACCTTAGGAATAATTTA
TGCCATGATAGCTATCGGATTATTAGGATTCGTAGTTTGAGCTCACCATATATTT
ACAGTAGATAGATGTAGATACCCGAGCTTACTTCACTTCAGCAACTATAATTATT
GCGGTTCCAACTGGGATTAAAGTCTTTAGATGACTAGCCACCTTTCATGGAACT
CAAATTTTAAATAGACCAGTAACCCTGTGAGCTTTAGGTTTTGTATTTTTATTTAC
AGTAGGAGGATTAACAGGGGTAATTTTAGCTAATTCTTCAATTGATATTATTCTTC
ACGATACTTACTATGTAGTAGCTCATTTTCACTATGTTCTTTCTATAGGAGCTGTA
TTTGCAATTATAGCCGGAATTGTCCAATGATTCCCATTATTTACCGGACTAACCT
TAAATAATTTTTTTTTAAAAACCCAATTTATTACTATATTTTTAGGTGTAAATTTAAC
ATTTTTTCCCCAACACTTTTTAGGGCTAAGAGGAATGCCTCGGCGATATTCGGAT
TATCCAGATGCTTTCACTATCTGAAATGCCATTTCATCTATTGGTTCCATTATTTC
TCTCACAAGAATTATCTTTTTTTTATTTATCTTATGAGAAAGGCTTTCTTCCCAAC
GTAAATCTTTGAGAACTTTAAATGTAACAACTTCAATTGAATGGTTCAAGCCCTAC
CCCCTAGTG
CMO1_1
ATTATTAGACAAGAAAGGGGGAAAAAAGAAGCTTTTGGACCTTAGGAATAATTTA
TGCCATGATAGCTATCGGATTATTAGGATTCGTAGTTTGAGCTCACCATATATTT
ACAGTAGATAGATGTAGATACCCGAGCTTACTTCACTTCAGCAACTATAATTATT
GCGGTTCCAACTGGGATTAAAGTCTTTAGATGACTAGCCACCTTTCATGGAACT
CAAATTTTAAATAGACCAGTAACCCTGTGAGCTTTAGGTTTTGTATTTTTATTTAC
AGTAGGAGGATTAACAGGGGTAATTTTAGCTAATTCTTCAATTGATATTATTCTTC

ACGATACTTACTATGTAGTAGCTCATTTTCACTATGTTCTTTCTATAGGAGCTGTA
TTTGCAATTATAGCCGGAATTGTCCAATGATTCCCATTATTTACCGGACTAACCT
TAAATAATTTTTTTTTAAAAACCCAATTTATTACTATATTTTTAGGTGTAAATTTAAC
ATTTTTTCCCCAACACTTTTTAGGGCTAAGAGGAATGCCTCGGCGATATTCGGAT
TATCCAGATGCTTTCACTATCTGAAATGCCATTTCATCTATTGGTTCCATTATTTC
TCTCACAAGAATTATCTTTTTTTTATTTATCTTATGAGAAAGGCTTTCTTCCCAAC
GTAAATCTTTGAGAACTTTAAATGTAACAACTTCAATTGAATGGTTCAAGCCCTAC
CCCCTAGTG
CMO1_2
ATTATTAGACAAGAAAGGGGGAAAAAAGAAGCTTTTGGACCTTAGGAATAATTTA
TGCCATGATAGCTATCGGATTATTAGGATTCGTAGTTTGAGCTCACCATATATTT
ACAGTAGATAGATGTAGATACCCGAGCTTACTTCACTTCAGCAACTATAATTATT
GCGGTTCCAACTGGGATTAAAGTCTTTAGATGACTAGCCACCTTTCATGGAACT
CAAATTTTAAATAGACCAGTAACCCTGTGAGCTTTAGGTTTTGTATTTTTATTTAC
AGTAGGAGGATTAACAGGGGTAATTTTAGCTAATTCTTCAATTGATATTATTCTTC
ACGATACTTACTATGTAGTAGCTCATTTTCACTATGTTCTTTCTATAGGAGCTGTA
TTTGCAATTATAGCCGGAATTGTCCAATGATTCCCATTATTTACCGGACTAACCT
TAAATAATTTTTTTTTAAAAACCCAATTTATTACTATATTTTTAGGTGTAAATTTAAC
ATTTTTTCCCCAACACTTTTTAGGGCTAAGAGGAATGCCTCGGCGATATTCGGAT
TATCCAGATGCTTTCACTATCTGAAATGCCATTTCATCTATTGGTTCCATTATTTC
TCTCACAAGAATTATCTTTTTTTTATTTATCTTATGAGAAAGGCTTTCTTCCCAAC
GTAAATCTTTGAGAACTTTAAATGTAACAACTTCAATTGAATGGTTCAAGCCCTAC
CCCCTAGTG
CMO2_1
ATTATTAGACAAGAAAGGGGGAAAAAAGAAGCTTTTGGACCTTAGGAATAATTTA
TGCCATGATAGCTATCGGATTATTAGGATTCGTAGTTTGAGCTCACCATATATTT
ACAGTAGATAGATGTAGATACCCGAGCTTACTTCACTTCAGCAACTATAATTATT
GCGGTTCCAACTGGGATTAAAGTCTTTAGATGACTAGCCACCTTTCATGGAACT
CAAATTTTAAATAGACCAGTAACCCTGTGAGCTTTAGGTTTTGTATTTTTATTTAC
AGTAGGAGGATTAACAGGGGTAATTTTAGCTAATTCTTCAATTGATATTATTCTTC
ACGATACTTACTATGTAGTAGCTCATTTTCACTATGTTCTTTCTATAGGAGCTGTA
TTTGCAATTATAGCCGGAATTGTCCAATGATTCCCATTATTTACCGGACTAACCT
TAAATAATTTTTTTTTAAAAACCCAATTTATTACTATATTTTTAGGTGTAAATTTAAC
ATTTTTTCCCCAACACTTTTTAGGGCTAAGAGGAATGCCTCGGCGATATTCGGAT
TATCCAGATGCTTTCACTATCTGAAATGCCATTTCATCTATTGGTTCCATTATTTC
TCTCACAAGAATTATCTTTTTTTTATTTATCTTATGAGAAAGGCTTTCTTCCCAAC
GTAAATCTTTGAGAACTTTAAATGTAACAACTTCAATTGAATGGTTCAAGCCCTAC
CCCCTAGTG
CMO2_2
ATTATTAGACAAGAAAGGGGGAAAAAAGAAGCTTTTGGACCTTAGGAATAATTTA
TGCCATGATAGCTATCGGATTATTAGGATTCGTAGTTTGAGCTCACCATATATTT
ACAGTAGATAGATGTAGATACCCGAGCTTACTTCACTTCAGCAACTATAATTATT
GCGGTTCCAACTGGGATTAAAGTCTTTAGATGACTAGCCACCTTTCATGGAACT
CAAATTTTAAATAGACCAGTAACCCTGTGAGCTTTAGGTTTTGTATTTTTATTTAC
AGTAGGAGGATTAACAGGGGTAATTTTAGCTAATTCTTCAATTGATATTATTCTTC
ACGATACTTACTATGTAGTAGCTCATTTTCACTATGTTCTTTCTATAGGAGCTGTA
TTTGCAATTATAGCCGGAATTGTCCAATGATTCCCATTATTTACCGGACTAACCT
TAAATAATTTTTTTTTAAAAACCCAATTTATTACTATATTTTTAGGTGTAAATTTAAC
ATTTTTTCCCCAACACTTTTTAGGGCTAAGAGGAATGCCTCGGCGATATTCGGAT
TATCCAGATGCTTTCACTATCTGAAATGCCATTTCATCTATTGGTTCCATTATTTC

TCTCACAAGAATTATCTTTTTTTTATTTATCTTATGAGAAAGGCTTTCTTCCCAAC
GTAAATCTTTGAGAACTTTAAATGTAACAACTTCAATTGAATGGTTCAAGCCCTAC
CCCCTAGTG
COI1
ATTATTAGACAAGAAAGGGGGAAAAAAGAAGCTTTTGGACCTTAGGAATAATTTA
TGCCATGATAGCTATCGGATTATTAGGATTCGTAGTTTGAGCTCACCATATATTT
ACAGTAGATAGATGTAGATACCCGAGCTTACTTCACTTCAGCAACTATAATTATT
GCGGTTCCAACTGGGATTAAAGTCTTTAGATGACTAGCCACCTTTCATGGAACT
CAAATTTTAAATAGACCAGTAACCCTGTGAGCTTTAGGTTTTGTATTTTTATTTAC
AGTAGGAGGATTAACAGGGGTAATTTTAGCTAATTCTTCAATTGATATTATTCTTC
ACGATACTTACTATGTAGTAGCTCATTTTCACTATGTTCTTTCTATAGGAGCTGTA
TTTGCAATTATAGCCGGAATTGTCCAATGATTCCCATTATTTACCGGACTAACCT
TAAATAATTTTTTTTTAAAAACCCAATTTATTACTATATTTTTAGGTGTAAATTTAAC
ATTTTTTCCCCAACACTTTTTAGGGCTAAGAGGAATGCCTCGGCGATATTCGGAT
TATCCAGATGCTTTCACTATCTGAAATGCCATTTCATCTATTGGTTCCATTATTTC
TCTCACAAGAATTATCTTTTTTTTATTTATCTTATGAGAAAGGCTTTCTTCCCAAC
GTAAATCTTTGAGAACTTTAAATGTAACAACTTCAATTGAATGGTTCAAGCCCTAC
CCCCTAGTG
CSJ1
ATTATTAGACAAGAAAGGGGGAAAAAAGAAGCTTTTGGACCTTAGGAATAATTTA
TGCCATGATAGCTATCGGATTATTAGGATTCGTAGTTTGAGCTCACCATATATTT
ACAGTAGATAGATGTAGATACCCGAGCTTACTTCACTTCAGCAACTATAATTATT
GCGGTTCCAACTGGGATTAAAGTCTTTAGATGACTAGCCACCTTTCATGGAACT
CAAATTTTAAATAGACCAGTAACCCTGTGAGCTTTAGGTTTTGTATTTTTATTTAC
AGTAGGAGGATTAACAGGGGTAATTTTAGCTAATTCTTCAATTGATATTATTCTTC
ACGATACTTACTATGTAGTAGCTCATTTTCACTATGTTCTTTCTATAGGAGCTGTA
TTTGCAATTATAGCCGGAATTGTCCAATGATTCCCATTATTTACCGGACTAACCT
TAAATAATTTTTTTTTAAAAACCCAATTTATTACTATATTTTTAGGTGTAAATTTAAC
ATTTTTTCCCCAACACTTTTTAGGGCTAAGAGGAATGCCTCGGCGATATTCGGAT
TATCCAGATGCTTTCACTATCTGAAATGCCATTTCATCTATTGGTTCCATTATTTC
TCTCACAAGAATTATCTTTTTTTTATTTATCTTATGAGAAAGGCTTTCTTCCCAAC
GTAAATCTTTGAGAACTTTAAATGTAACAACTTCAATTGAATGGTTCAAGCCCTAC
CCCCTAGTG
ERV1
ATTATTAGACAAGAAAGGGGGAAAAAAGAAGCTTTTGGACCTTAGGAATAATTTA
TGCCATGATAGCTATCGGATTATTAGGATTCGTAGTTTGAGCTCACCATATATTT
ACAGTAGATAGATGTAGATACCCGAGCTTACTTCACTTCAGCAACTATAATTATT
GCGGTTCCAACTGGGATTAAAGTCTTTAGATGACTAGCCACCTTTCATGGAACT
CAAATTTTAAATAGACCAGTAACCCTGTGAGCTTTAGGTTTTGTATTTTTATTTAC
AGTAGGAGGATTAACAGGGGTAATTTTAGCTAATTCTTCAATTGATATTATTCTTC
ACGATACTTACTATGTAGTAGCTCATTTTCACTATGTTCTTTCTATAGGAGCTGTA
TTTGCAATTATAGCCGGAATTGTCCAATGATTCCCATTATTTACCGGACTAACCT
TAAATAATTTTTTTTTAAAAACCCAATTTATTACTATATTTTTAGGTGTAAATTTAAC
ATTTTTTCCCCAACACTTTTTAGGGCTAAGAGGAATGCCTCGGCGATATTCGGAT
TATCCAGATGCTTTCACTATCTGAAATGCCATTTCATCTATTGGTTCCATTATTTC
TCTCACAAGAATTATCTTTTTTTTATTTATCTTATGAGAAAGGCTTTCTTCCCAAC
GTAAATCTTTGAGAACTTTAAATGTAACAACTTCAATTGAATGGTTCAAGCCCTAC
CCCCTAGTG
ERV2
ATTATTAGACAAGAAAGGGGGAAAAAAGAAGCTTTTGGACCTTAGGAATAATTTA

TGCCATGATAGCTATCGGATTATTAGGATTCGTAGTTTGAGCTCACCATATATTT
ACAGTAGATAGATGTAGATACCCGAGCTTACTTCACTTCAGCAACTATAATTATT
GCGGTTCCAACTGGGATTAAAGTCTTTAGATGACTAGCCACCTTTCATGGAACT
CAAATTTTAAATAGACCAGTAACCCTGTGAGCTTTAGGTTTTGTATTTTTATTTAC
AGTAGGAGGATTAACAGGGGTAATTTTAGCTAATTCTTCAATTGATATTATTCTTC
ACGATACTTACTATGTAGTAGCTCATTTTCACTATGTTCTTTCTATAGGAGCTGTA
TTTGCAATTATAGCCGGAATTGTCCAATGATTCCCATTATTTACCGGACTAACCT
TAAATAATTTTTTTTTAAAAACCCAATTTATTACTATATTTTTAGGTGTAAATTTAAC
ATTTTTTCCCCAACACTTTTTAGGGCTAAGAGGAATGCCTCGGCGATATTCGGAT
TATCCAGATGCTTTCACTATCTGAAATGCCATTTCATCTATTGGTTCCATTATTTC
TCTCACAAGAATTATCTTTTTTTTATTTATCTTATGAGAAAGGCTTTCTTCCCAAC
GTAAATCTTTGAGAACTTTAAATGTAACAACTTCAATTGAATGGTTCAAGCCCTAC
CCCCTAGTG
GOV1
ATTATTAGACAAGAAAGGGGGAAAAAAGAAGCTTTTGGACCTTAGGAATAATTTA
TGCCATGATAGCTATCGGATTATTAGGATTCGTAGTTTGAGCTCACCATATATTT
ACAGTAGATAGATGTAGATACCCGAGCTTACTTCACTTCAGCAACTATAATTATT
GCGGTTCCAACTGGGATTAAAGTCTTTAGATGACTAGCCACCTTTCATGGAACT
CAAATTTTAAATAGACCAGTAACCCTGTGAGCTTTAGGTTTTGTATTTTTATTTAC
AGTAGGAGGATTAACAGGGGTAATTTTAGCTAATTCTTCAATTGATATTATTCTTC
ACGATACTTACTATGTAGTAGCTCATTTTCACTATGTTCTTTCTATAGGAGCTGTA
TTTGCAATTATAGCCGGAATTGTCCAATGATTCCCATTATTTACCGGACTAACCT
TAAATAATTTTTTTTTAAAAACCCAATTTATTACTATATTTTTAGGTGTAAATTTAAC
ATTTTTTCCCCAACACTTTTTAGGGCTAAGAGGAATGCCTCGGCGATATTCGGAT
TATCCAGATGCTTTCACTATCTGAAATGCCATTTCATCTATTGGTTCCATTATTTC
TCTCACAAGAATTATCTTTTTTTTATTTATCTTATGAGAAAGGCTTTCTTCCCAAC
GTAAATCTTTGAGAACTTTAAATGTAACAACTTCAATTGAATGGTTCAAGCCCTAC
CCCCTAGTG
GOV2
ATTATTAGACAAGAAAGGGGGAAAAAAGAAGCTTTTGGACCTTAGGAATAATTTA
TGCCATGATAGCTATCGGATTATTAGGATTCGTAGTTTGAGCTCACCATATATTT
ACAGTAGATAGATGTAGATACCCGAGCTTACTTCACTTCAGCAACTATAATTATT
GCGGTTCCAACTGGGATTAAAGTCTTTAGATGACTAGCCACCTTTCATGGAACT
CAAATTTTAAATAGACCAGTAACCCTGTGAGCTTTAGGTTTTGTATTTTTATTTAC
AGTAGGAGGATTAACAGGGGTAATTTTAGCTAATTCTTCAATTGATATTATTCTTC
ACGATACTTACTATGTAGTAGCTCATTTTCACTATGTTCTTTCTATAGGAGCTGTA
TTTGCAATTATAGCCGGAATTGTCCAATGATTCCCATTATTTACCGGACTAACCT
TAAATAATTTTTTTTTAAAAACCCAATTTATTACTATATTTTTAGGTGTAAATTTAAC
ATTTTTTCCCCAACACTTTTTAGGGCTAAGAGGAATGCCTCGGCGATATTCGGAT
TATCCAGATGCTTTCACTATCTGAAATGCCATTTCATCTATTGGTTCCATTATTTC
TCTCACAAGAATTATCTTTTTTTTATTTATCTTATGAGAAAGGCTTTCTTCCCAAC
GTAAATCTTTGAGAACTTTAAATGTAACAACTTCAATTGAATGGTTCAAGCCCTAC
CCCCTAGTG
HEM1
ATTATTAGACAAGAAAGGGGGAAAAAAGAAGCTTTTGGACCTTAGGAATAATTTA
TGCCATGATAGCTATCGGATTATTAGGATTCGTAGTTTGAGCTCACCATATATTT
ACAGTAGATAGATGTAGATACCCGAGCTTACTTCACTTCAGCAACTATAATTATT
GCGGTTCCAACTGGGATTAAAGTCTTTAGATGACTAGCCACCTTTCATGGAACT
CAAATTTTAAATAGACCAGTAACCCTGTGAGCTTTAGGTTTTGTATTTTTATTTAC
AGTAGGAGGATTAACAGGGGTAATTTTAGCTAATTCTTCAATTGATATTATTCTTC

ACGATACTTACTATGTAGTAGCTCATTTTCACTATGTTCTTTCTATAGGAGCTGTA
TTTGCAATTATAGCCGGAATTGTCCAATGATTCCCATTATTTACCGGACTAACCT
TAAATAATTTTTTTTTAAAAACCCAATTTATTACTATATTTTTAGGTGTAAATTTAAC
ATTTTTTCCCCAACACTTTTTAGGGCTAAGAGGAATGCCTCGGCGATATTCGGAT
TATCCAGATGCTTTCACTATCTGAAATGCCATTTCATCTATTGGTTCCATTATTTC
TCTCACAAGAATTATCTTTTTTTTATTTATCTTATGAGAAAGGCTTTCTTCCCAAC
GTAAATCTTTGAGAACTTTAAATGTAACAACTTCAATTGAATGGTTCAAGCCCTAC
CCCCTAGTG
HEM2
ATTATTAGACAAGAAAGGGGGAAAAAAGAAGCTTTTGGACCTTAGGAATAATTTA
TGCCATGATAGCTATCGGATTATTAGGATTCGTAGTTTGAGCTCACCATATATTT
ACAGTAGATAGATGTAGATACCCGAGCTTACTTCACTTCAGCAACTATAATTATT
GCGGTTCCAACTGGGATTAAAGTCTTTAGATGACTAGCCACCTTTCATGGAACT
CAAATTTTAAATAGACCAGTAACCCTGTGAGCTTTAGGTTTTGTATTTTTATTTAC
AGTAGGAGGATTAACAGGGGTAATTTTAGCTAATTCTTCAATTGATATTATTCTTC
ACGATACTTACTATGTAGTAGCTCATTTTCACTATGTTCTTTCTATAGGAGCTGTA
TTTGCAATTATAGCCGGAATTGTCCAATGATTCCCATTATTTACCGGACTAACCT
TAAATAATTTTTTTTTAAAAACCCAATTTATTACTATATTTTTAGGTGTAAATTTAAC
ATTTTTTCCCCAACACTTTTTAGGGCTAAGAGGAATGCCTCGGCGATATTCGGAT
TATCCAGATGCTTTCACTATCTGAAATGCCATTTCATCTATTGGTTCCATTATTTC
TCTCACAAGAATTATCTTTTTTTTATTTATCTTATGAGAAAGGCTTTCTTCCCAAC
GTAAATCTTTGAGAACTTTAAATGTAACAACTTCAATTGAATGGTTCAAGCCCTAC
CCCCTAGTG
HER1
ATTATTAGACAAGAAAGGGGGAAAAAAGAAGCTTTTGGACCTTAGGAATAATTTA
TGCCATGATAGCTATCGGATTATTAGGATTCGTAGTTTGAGCTCACCATATATTT
ACAGTAGATAGATGTAGATACCCGAGCTTACTTCACTTCAGCAACTATAATTATT
GCGGTTCCAACTGGGATTAAAGTCTTTAGATGACTAGCCACCTTTCATGGAACT
CAAATTTTAAATAGACCAGTAACCCTGTGAGCTTTAGGTTTTGTATTTTTATTTAC
AGTAGGAGGATTAACAGGGGTAATTTTAGCTAATTCTTCAATTGATATTATTCTTC
ACGATACTTACTATGTAGTAGCTCATTTTCACTATGTTCTTTCTATAGGAGCTGTA
TTTGCAATTATAGCCGGAATTGTCCAATGATTCCCATTATTTACCGGACTAACCT
TAAATAATTTTTTTTTAAAAACCCAATTTATTACTATATTTTTAGGTGTAAATTTAAC
ATTTTTTCCCCAACACTTTTTAGGGCTAAGAGGAATGCCTCGGCGATATTCGGAT
TATCCAGATGCTTTCACTATCTGAAATGCCATTTCATCTATTGGTTCCATTATTTC
TCTCACAAGAATTATCTTTTTTTTATTTATCTTATGAGAAAGGCTTTCTTCCCAAC
GTAAATCTTTGAGAACTTTAAATGTAACAACTTCAATTGAATGGTTCAAGCCCTAC
CCCCTAGTG
HER2
ATTATTAGACAAGAAAGGGGGAAAAAAGAAGCTTTTGGACCTTAGGAATAATTTA
TGCCATGATAGCTATCGGATTATTAGGATTCGTAGTTTGAGCTCACCATATATTT
ACAGTAGATAGATGTAGATACCCGAGCTTACTTCACTTCAGCAACTATAATTATT
GCGGTTCCAACTGGGATTAAAGTCTTTAGATGACTAGCCACCTTTCATGGAACT
CAAATTTTAAATAGACCAGTAACCCTGTGAGCTTTAGGTTTTGTATTTTTATTTAC
AGTAGGAGGATTAACAGGGGTAATTTTAGCTAATTCTTCAATTGATATTATTCTTC
ACGATACTTACTATGTAGTAGCTCATTTTCACTATGTTCTTTCTATAGGAGCTGTA
TTTGCAATTATAGCCGGAATTGTCCAATGATTCCCATTATTTACCGGACTAACCT
TAAATAATTTTTTTTTAAAAACCCAATTTATTACTATATTTTTAGGTGTAAATTTAAC
ATTTTTTCCCCAACACTTTTTAGGGCTAAGAGGAATGCCTCGGCGATATTCGGAT
TATCCAGATGCTTTCACTATCTGAAATGCCATTTCATCTATTGGTTCCATTATTTC

TCTCACAAGAATTATCTTTTTTTTATTTATCTTATGAGAAAGGCTTTCTTCCCAAC
GTAAATCTTTGAGAACTTTAAATGTAACAACTTCAATTGAATGGTTCAAGCCCTAC
CCCCTAGTG
HEV1
ATTATTAGACAAGAAAGGGGGAAAAAAGAAGCTTTTGGACCTTAGGAATAATTTA
TGCCATGATAGCTATCGGATTATTAGGATTCGTAGTTTGAGCTCACCATATATTT
ACAGTAGATAGATGTAGATACCCGAGCTTACTTCACTTCAGCAACTATAATTATT
GCGGTTCCAACTGGGATTAAAGTCTTTAGATGACTAGCCACCTTTCATGGAACT
CAAATTTTAAATAGACCAGTAACCCTGTGAGCTTTAGGTTTTGTATTTTTATTTAC
AGTAGGAGGATTAACAGGGGTAATTTTAGCTAATTCTTCAATTGATATTATTCTTC
ACGATACTTACTATGTAGTAGCTCATTTTCACTATGTTCTTTCTATAGGAGCTGTA
TTTGCAATTATAGCCGGAATTGTCCAATGATTCCCATTATTTACCGGACTAACCT
TAAATAATTTTTTTTTAAAAACCCAATTTATTACTATATTTTTAGGTGTAAATTTAAC
ATTTTTTCCCCAACACTTTTTAGGGCTAAGAGGAATGCCTCGGCGATATTCGGAT
TATCCAGATGCTTTCACTATCTGAAATGCCATTTCATCTATTGGTTCCATTATTTC
TCTCACAAGAATTATCTTTTTTTTATTTATCTTATGAGAAAGGCTTTCTTCCCAAC
GTAAATCTTTGAGAACTTTAAATGTAACAACTTCAATTGAATGGTTCAAGCCCTAC
CCCCTAGTG
HEV1_2
ATTATTAGACAAGAAAGGGGGAAAAAAGAAGCTTTTGGACCTTAGGAATAATTTA
TGCCATGATAGCTATCGGATTATTAGGATTCGTAGTTTGAGCTCACCATATATTT
ACAGTAGATAGATGTAGATACCCGAGCTTACTTCACTTCAGCAACTATAATTATT
GCGGTTCCAACTGGGATTAAAGTCTTTAGATGACTAGCCACCTTTCATGGAACT
CAAATTTTAAATAGACCAGTAACCCTGTGAGCTTTAGGTTTTGTATTTTTATTTAC
AGTAGGAGGATTAACAGGGGTAATTTTAGCTAATTCTTCAATTGATATTATTCTTC
ACGATACTTACTATGTAGTAGCTCATTTTCACTATGTTCTTTCTATAGGAGCTGTA
TTTGCAATTATAGCCGGAATTGTCCAATGATTCCCATTATTTACCGGACTAACCT
TAAATAATTTTTTTTTAAAAACCCAATTTATTACTATATTTTTAGGTGTAAATTTAAC
ATTTTTTCCCCAACACTTTTTAGGGCTAAGAGGAATGCCTCGGCGATATTCGGAT
TATCCAGATGCTTTCACTATCTGAAATGCCATTTCATCTATTGGTTCCATTATTTC
TCTCACAAGAATTATCTTTTTTTTATTTATCTTATGAGAAAGGCTTTCTTCCCAAC
GTAAATCTTTGAGAACTTTAAATGTAACAACTTCAATTGAATGGTTCAAGCCCTAC
CCCCTAGTG
HEV2
ATTATTAGACAAGAAAGGGGGAAAAAAGAAGCTTTTGGACCTTAGGAATAATTTA
TGCCATGATAGCTATCGGATTATTAGGATTCGTAGTTTGAGCTCACCATATATTT
ACAGTAGATAGATGTAGATACCCGAGCTTACTTCACTTCAGCAACTATAATTATC
GCGGTTCCAACTGGGATTAAAGTCTTTAGGTGACTAGCCACCTTTCATGGAACT
CAAATTTTAAATAGACCAGTAACCCTGTGAGCTTTAGGTTTTGTATTTTTATTTAC
AGTAGGAGGATTAACAGGGGTAATTTTAGCTAATTCTTCAATTGATATTATTCTTC
ACGATACTTACTATGTAGTAGCTCATTTTCACTATGTTCTTTCTATAGGAGCTGTA
TTTGCAATTATAGCCGGAATTGTCCAATGATTCCCATTATTTACTGGACTAACCTT
AAATAATTTTTTTTTAAAAACCCAATTTATTACTATATTTTTAGGTGTAAATTTAACA
TTTTTTCCTCAACACTTTTTAGGGCTAAGAGGAATGCCTCGGCGATATTCGGATT
ATCCAGATGCTTTCACTATCTGAAATGCCATTTCATCTATTGGTTCCATTATTTCT
CTCACAAGAATTATCTTTTTTTTATTTATCTTATGAGAAAGGCTTTCTTCCCAACG
TAAATCTTTGAGAACTTTAAATGTAACAACTTCAATTGAATGGTTCAAGCCCTACC
CCCTAGTG
HEV2_2
ATTATTAGACAAGAAAGGGGGAAAAAAGAAGCTTTTGGACCTTAGGAATAATTTA

TGCCATGATAGCTATCGGATTATTAGGATTCGTAGTTTGAGCTCACCATATATTT
ACAGTAGATAGATGTAGATACCCGAGCTTACTTCACTTCAGCAACTATAATTATC
GCGGTTCCAACTGGGATTAAAGTCTTTAGGTGACTAGCCACCTTTCATGGAACT
CAAATTTTAAATAGACCAGTAACCCTGTGAGCTTTAGGTTTTGTATTTTTATTTAC
AGTAGGAGGATTAACAGGGGTAATTTTAGCTAATTCTTCAATTGATATTATTCTTC
ACGATACTTACTATGTAGTAGCTCATTTTCACTATGTTCTTTCTATAGGAGCTGTA
TTTGCAATTATAGCCGGAATTGTCCAATGATTCCCATTATTTACTGGACTAACCTT
AAATAATTTTTTTTTAAAAACCCAATTTATTACTATATTTTTAGGTGTAAATTTAACA
TTTTTTCCTCAACACTTTTTAGGGCTAAGAGGAATGCCTCGGCGATATTCGGATT
ATCCAGATGCTTTCACTATCTGAAATGCCATTTCATCTATTGGTTCCATTATTTCT
CTCACAAGAATTATCTTTTTTTTTATTTATCTTATGAGAAAGGCTTTCTTCCCAACG
TAAATCTTTGAGAACTTTAAATGTAACAACTTCAATTGAATGGTTCAAGCCCTACC
CCCTAGTG
JAB2
ATTATTAGACAAGAAAGGGGGAAAAAAGAAGCTTTTGGACCTTAGGAATAATTTA
TGCCATGATAGCTATCGGATTATTAGGATTCGTAGTTTGAGCTCACCATATATTT
ACAGTAGATAGATGTAGATACCCGAGCTTACTTCACTTCAGCAACTATAATTATT
GCGGTTCCAACTGGGATTAAAGTCTTTAGATGACTAGCCACCTTTCATGGAACT
CAAATTTTAAATAGACCAGTAACCCTGTGAGCTTTAGGTTTTGTATTTTTATTTAC
AGTAGGAGGATTAACAGGGGTAATTTTAGCTAATTCTTCAATTGATATTATTCTTC
ACGATACTTACTATGTAGTAGCTCATTTTCACTATGTTCTTTCTATAGGAGCTGTA
TTTGCAATTATAGCCGGAATTGTCCAATGATTCCCATTATTTACCGGACTAACCT
TAAATAATTTTTTTTTAAAAACCCAATTTATTACTATATTTTTAGGTGTAAATTTAAC
ATTTTTTCCCCAACACTTTTTAGGGCTAAGAGGAATGCCTCGGCGATATTCGGAT
TATCCAGATGCTTTCACTATCTGAAATGCCATTTCATCTATTGGTTCCATTATTTC
TCTCACAAGAATTATCTTTTTTTTTATTTATCTTATGAGAAAGGCTTTCTTCCCAAC
GTAAATCTTTGAGAACTTTAAATGTAACAACTTCAATTGAATGGTTCAAGCCCTAC
CCCCTAGTG
JAB4
ATTATTAGACAAGAAAGGGGGAAAAAAGAAGCTTTTGGACCTTAGGAATAATTTA
TGCCATGATAGCTATCGGATTATTAGGATTCGTAGTTTGAGCTCACCATATATTT
ACAGTAGATAGATGTAGATACCCGAGCTTACTTCACTTCAGCAACTATAATTATT
GCGGTTCCAACTGGGATTAAAGTCTTTAGATGACTAGCCACCTTTCATGGAACT
CAAATTTTAAATAGACCAGTAACCCTGTGAGCTTTAGGTTTTGTATTTTTATTTAC
AGTAGGAGGATTAACAGGGGTAATTTTAGCTAATTCTTCAATTGATATTATTCTTC
ACGATACTTACTATGTAGTAGCTCATTTTCACTATGTTCTTTCTATAGGAGCTGTA
TTTGCAATTATAGCCGGAATTGTCCAATGATTCCCATTATTTACCGGACTAACCT
TAAATAATTTTTTTTTAAAAACCCAATTTATTACTATATTTTTAGGTGTAAATTTAAC
ATTTTTTCCCCAACACTTTTTAGGGCTAAGAGGAATGCCTCGGCGATATTCGGAT
TATCCAGATGCTTTCACTATCTGAAATGCCATTTCATCTATTGGTTCCATTATTTC
TCTCACAAGAATTATCTTTTTTTTTATTTATCTTATGAGAAAGGCTTTCTTCCCAAC
GTAAATCTTTGAGAACTTTAAATGTAACAACTTCAATTGAATGGTTCAAGCCCTAC
CCCCTAGTG
LAV1
ATTATTAGACAAGAAAGGGGGAAAAAAGAAGCTTTTGGACCTTAGGAATAATTTA
TGCCATGATAGCTATCGGATTATTAGGATTCGTAGTTTGAGCTCACCATATATTT
ACAGTAGATAGATGTAGATACCCGAGCTTACTTCACTTCAGCAACTATAATTATT
GCGGTTCCAACTGGGATTAAAGTCTTTAGATGACTAGCCACCTTTCATGGAACT
CAAATTTTAAATAGACCAGTAACCCTGTGAGCTTTAGGTTTTGTATTTTTATTTAC
AGTAGGAGGATTAACAGGGGTAATTTTAGCTAATTCTTCAATTGATATTATTCTTC

ACGATACTTACTATGTAGTAGCTCATTTTCACTATGTTCTTTCTATAGGAGCTGTA
TTTGCAATTATAGCCGGAATTGTCCAATGATTCCCATTATTTACCGGACTAACCT
TAAATAATTTTTTTTTAAAAACCCAATTTATTACTATATTTTTAGGTGTAAATTTAAC
ATTTTTTCCCCAACACTTTTTAGGGCTAAGAGGAATGCCTCGGCGATATTCGGAT
TATCCAGATGCTTTCACTATCTGAAATGCCATTTCATCTATTGGTTCCATTATTTC
TCTCACAAGAATTATCTTTTTTTTTATTTATCTTATGAGAAAGGCTTTCTTCCCAAC
GTAAATCTTTGAGAACTTTAAATGTAACAACTTCAATTGAATGGTTCAAGCCCTAC
CCCCTAGTG
LAV2
ATTATTAGACAAGAAAGGGGGAAAAAAGAAGCTTTTGGACCTTAGGAATAATTTA
TGCCATGATAGCTATCGGATTATTAGGATTCGTAGTTTGAGCTCACCATATATTT
ACAGTAGATAGATGTAGATACCCGAGCTTACTTCACTTCAGCAACTATAATTATT
GCGGTTCCAACTGGGATTAAAGTCTTTAGATGACTAGCCACCTTTCATGGAACT
CAAATTTTAAATAGACCAGTAACCCTGTGAGCTTTAGGTTTTGTATTTTTATTTAC
AGTAGGAGGATTAACAGGGGTAATTTTAGCTAATTCTTCAATTGATATTATTCTTC
ACGATACTTACTATGTAGTAGCTCATTTTCACTATGTTCTTTCTATAGGAGCTGTA
TTTGCAATTATAGCCGGAATTGTTCAATGATTCCCATTATTTACCGGACTAACCTT
AAATAATTTTTTTTTAAAAACCCAATTTATTACTATATTTTTAGGTGTAAATTTAACA
TTTTTTCCCCAACACTTTTTAGGGCTAAGAGGAATGCCTCGGCGATATTCGGATT
ATCCAGATGCTTTCACTATCTGAAATGCCATTTCATCTATTGGTTCCATTATTTCT
CTCACAAGAATTATCTTTTTTTTTATTTATCTTATGAGAAAGGCTTTCTTCCCAACG
TAAATCTTTGAGAACTTTAAATGTAACAACTTCAATTGAATGGTTCAAGCCCTACC
CCCTAGTG
MAC2
ATTATTAGACAAGAAAGGGGGAAAAAAGAAGCTTTTGGACCTTAGGAATAATTTA
TGCCATGATAGCTATCGGATTATTAGGATTCGTAGTTTGAGCTCACCATATATTT
ACAGTAGATAGATGTAGATACCCGAGCTTACTTCACTTCAGCAACTATAATTATT
GCGGTTCCAACTGGGATTAAAGTCTTTAGATGACTAGCCACCTTTCATGGAACT
CAAATTTTAAATAGACCAGTAACCCTGTGAGCTTTAGGTTTTGTATTTTTATTTAC
AGTAGGAGGATTAACAGGGGTAATTTTAGCTAATTCTTCAATTGATATTATTCTTC
ACGATACTTACTATGTAGTAGCTCATTTTCACTATGTTCTTTCTATAGGAGCTGTA
TTTGCAATTATAGCCGGAATTGTCCAATGATTCCCATTATTTACCGGACTAACCT
TAAATAATTTTTTTTTAAAAACCCAATTTATTACTATATTTTTAGGTGTAAATTTAAC
ATTTTTTCCCCAACACTTTTTAGGGCTAAGAGGAATGCCTCGGCGATATTCGGAT
TATCCAGATGCTTTCACTATCTGAAATGCCATTTCATCTATTGGTTCCATTATTTC
TCTCACAAGAATTATCTTTTTTTTTATTTATCTTATGAGAAAGGCTTTCTTCCCAAC
GTAAATCTTTGAGAACTTTAAATGTAACAACTTCAATTGAATGGTTCAAGCCCTAC
CCCCTAGTG
MAC4
ATTATTAGACAAGAAAGGGGGAAAAAAGAAGCTTTTGGACCTTAGGAATAATTTA
TGCCATGATAGCTATCGGATTATTAGGATTCGTAGTTTGAGCTCACCATATATTT
ACAGTAGATAGATGTAGATACCCGAGCTTACTTCACTTCAGCAACTATAATTATT
GCGGTTCCAACTGGGATTAAAGTCTTTAGATGACTAGCCACCTTTCATGGAACT
CAAATTTTAAATAGACCAGTAACCCTGTGAGCTTTAGGTTTTGTATTTTTATTTAC
AGTAGGAGGATTAACAGGGGTAATTTTAGCTAATTCTTCAATTGATATTATTCTTC
ACGATACTTACTATGTAGTAGCTCATTTTCACTATGTTCTTTCTATAGGAGCTGTA
TTTGCAATTATAGCCGGAATTGTCCAATGATTCCCATTATTTACCGGACTAACCT
TAAATAATTTTTTTTTAAAAACCCAATTTATTACTATATTTTTAGGTGTAAATTTAAC
ATTTTTTCCCCAACACTTTTTAGGGCTAAGAGGAATGCCTCGGCGATATTCGGAT
TATCCAGATGCTTTCACTATCTGAAATGCCATTTCATCTATTGGTTCCATTATTTC

TCTCACAAGAATTATCTTTTTTTTATTTATCTTATGAGAAAGGCTTTCTTCCCAAC
GTAAATCTTTGAGAACTTTAAATGTAACAACTTCAATTGAATGGTTCAAGCCCTAC
CCCCTAGTG
MAC6
ATTATTAGACAAGAAAGGGGGAAAAAAGAAGCTTTTGGACCTTAGGAATAATTTA
TGCCATGATAGCTATCGGATTATTAGGATTCGTAGTTTGAGCTCACCATATATTT
ACAGTAGATAGATGTAGATACCCGAGCTTACTTCACTTCAGCAACTATAATTATT
GCGGTTCCAACTGGGATTAAAGTCTTTAGATGACTAGCCACCTTTCATGGAACT
CAAATTTTAAATAGACCAGTAACCCTGTGAGCTTTAGGTTTTGTATTTTTATTTAC
AGTAGGAGGATTAACAGGGGTAATTTTAGCTAATTCTTCAATTGATATTATTCTTC
ACGATACTTACTATGTAGTAGCTCATTTTCACTATGTTCTTTCTATAGGAGCTGTA
TTTGCAATTATAGCCGGAATTGTCCAATGATTCCCATTATTTACCGGACTAACCT
TAAATAATTTTTTTTTAAAAACCCAATTTATTACTATATTTTTAGGTGTAAATTTAAC
ATTTTTTCCCCAACACTTTTTAGGGCTAAGAGGAATGCCTCGGCGATATTCGGAT
TATCCAGATGCTTTCACTATCTGAAATGCCATTTCATCTATTGGTTCCATTATTTC
TCTCACAAGAATTATCTTTTTTTTATTTATCTTATGAGAAAGGCTTTCTTCCCAAC
GTAAATCTTTGAGAACTTTAAATGTAACAACTTCAATTGAATGGTTCAAGCCCTAC
CCCCTAGTG
MAR2_1
ATTATTAGACAAGAAAGGGGGAAAAAAGAAGCTTTTGGACCTTAGGAATAATTTA
TGCCATGATAGCTATCGGATTATTAGGATTCGTAGTTTGAGCTCACCATATATTT
ACAGTAGATAGATGTAGATACCCGAGCTTACTTCACTTCAGCAACTATAATTATT
GCGGTTCCAACTGGGATTAAAGTCTTTAGATGACTAGCCACCTTTCATGGAACT
CAAATTTTAAATAGACCAGTAACCCTGTGAGCTTTAGGTTTTGTATTTTTATTTAC
AGTAGGAGGATTAACAGGGGTAATTTTAGCTAATTCTTCAATTGATATTATTCTTC
ACGATACTTACTATGTAGTAGCTCATTTTCACTATGTTCTTTCTATAGGAGCTGTA
TTTGCAATTATAGCCGGAATTGTCCAATGATTCCCATTATTTACCGGACTAACCT
TAAATAATTTTTTTTTAAAAACCCAATTTATTACTATATTTTTAGGTGTAAATTTAAC
ATTTTTTCCCCAACACTTTTTAGGGCTAAGAGGAATGCCTCGGCGATATTCGGAT
TATCCAGATGCTTTCACTATCTGAAATGCCATTTCATCTATTGGTTCCATTATTTC
TCTCACAAGAATTATCTTTTTTTTATTTATCTTATGAGAAAGGCTTTCTTCCCAAC
GTAAATCTTTGAGAACTTTAAATGTAACAACTTCAATTGAATGGTTCAAGCCCTAC
CCCCTAGTG
MAR2_2
ATTATTAGACAAGAAAGGGGGAAAAAAGAAGCTTTTGGACCTTAGGAATAATTTA
TGCCATGATAGCTATCGGATTATTAGGATTCGTAGTTTGAGCTCACCATATATTT
ACAGTAGATAGATGTAGATACCCGAGCTTACTTCACTTCAGCAACTATAATTATT
GCGGTTCCAACTGGGATTAAAGTCTTTAGATGACTAGCCACCTTTCATGGAACT
CAAATTTTAAATAGACCAGTAACCCTGTGAGCTTTAGGTTTTGTATTTTTATTTAC
AGTAGGAGGATTAACAGGGGTAATTTTAGCTAATTCTTCAATTGATATTATTCTTC
ACGATACTTACTATGTAGTAGCTCATTTTCACTATGTTCTTTCTATAGGAGCTGTA
TTTGCAATTATAGCCGGAATTGTCCAATGATTCCCATTATTTACCGGACTAACCT
TAAATAATTTTTTTTTAAAAACCCAATTTATTACTATATTTTTAGGTGTAAATTTAAC
ATTTTTTCCCCAACACTTTTTAGGGCTAAGAGGAATGCCTCGGCGATATTCGGAT
TATCCAGATGCTTTCACTATCTGAAATGCCATTTCATCTATTGGTTCCATTATTTC
TCTCACAAGAATTATCTTTTTTTTATTTATCTTATGAGAAAGGCTTTCTTCCCAAC
GTAAATCTTTGAGAACTTTAAATGTAACAACTTCAATTGAATGGTTCAAGCCCTAC
CCCCTAGTG
MAR3_1
ATTATTAGACAAGAAAGGGGGAAAAAAGAAGCTTTTGGACCTTAGGAATAATTTA

TGCCATGATAGCTATCGGATTATTAGGATTCGTAGTTTGAGCTCACCATATATTT
ACAGTAGATAGATGTAGATACCCGAGCTTACTTCACTTCAGCAACTATAATTATT
GCGGTTCCAACTGGGATTAAAGTCTTTAGATGACTAGCCACCTTTCATGGAACT
CAAATTTTAAATAGACCAGTAACCCTGTGAGCTTTAGGTTTTGTATTTTTATTTAC
AGTAGGAGGATTAACAGGGGTAATTTTAGCTAATTCTTCAATTGATATTATTCTTC
ACGATACTTACTATGTAGTAGCTCATTTTCACTATGTTCTTTCTATAGGAGCTGTA
TTTGCAATTATAGCCGGAATTGTCCAATGATTCCCATTATTTACCGGACTAACCT
TAAATAATTTTTTTTTAAAAACCCAATTTATTACTATATTTTTAGGTGTAAATTTAAC
ATTTTTTCCCCAACACTTTTTAGGGCTAAGAGGAATGCCTCGGCGATATTCGGAT
TATCCAGATGCTTTCACTATCTGAAATGCCATTTCATCTATTGGTTCCATTATTTC
TCTCACAAGAATTATCTTTTTTTTATTTATCTTATGAGAAAGGCTTTCTTCCCAAC
GTAAATCTTTGAGAACTTTAAATGTAACAACTTCAATTGAATGGTTCAAGCCCTAC
CCCCTAGTG
MAR3_2
ATTATTAGACAAGAAAGGGGGAAAAAAGAAGCTTTTGGACCTTAGGAATAATTTA
TGCCATGATAGCTATCGGATTATTAGGATTCGTAGTTTGAGCTCACCATATATTT
ACAGTAGATAGATGTAGATACCCGAGCTTACTTCACTTCAGCAACTATAATTATT
GCGGTTCCAACTGGGATTAAAGTCTTTAGATGACTAGCCACCTTTCATGGAACT
CAAATTTTAAATAGACCAGTAACCCTGTGAGCTTTAGGTTTTGTATTTTTATTTAC
AGTAGGAGGATTAACAGGGGTAATTTTAGCTAATTCTTCAATTGATATTATTCTTC
ACGATACTTACTATGTAGTAGCTCATTTTCACTATGTTCTTTCTATAGGAGCTGTA
TTTGCAATTATAGCCGGAATTGTCCAATGATTCCCATTATTTACCGGACTAACCT
TAAATAATTTTTTTTTAAAAACCCAATTTATTACTATATTTTTAGGTGTAAATTTAAC
ATTTTTTCCCCAACACTTTTTAGGGCTAAGAGGAATGCCTCGGCGATATTCGGAT
TATCCAGATGCTTTCACTATCTGAAATGCCATTTCATCTATTGGTTCCATTATTTC
TCTCACAAGAATTATCTTTTTTTTATTTATCTTATGAGAAAGGCTTTCTTCCCAAC
GTAAATCTTTGAGAACTTTAAATGTAACAACTTCAATTGAATGGTTCAAGCCCTAC
CCCCTAGTG
MOC1
ATTATTAGACAAGAAAGGGGGAAAAAAGAAGCTTTTGGACCTTAGGAATAATTTA
TGCCATGATAGCTATCGGATTATTAGGATTCGTAGTTTGAGCTCACCATATATTT
ACAGTAGATAGATGTAGATACCCGAGCTTACTTCACTTCAGCAACTATAATTATT
GCGGTTCCAACTGGGATTAAAGTCTTTAGATGACTAGCCACCTTTCATGGAACT
CAAATTTTAAATAGACCAGTAACCCTGTGAGCTTTAGGTTTTGTATTTTTATTTAC
AGTAGGAGGATTAACAGGGGTAATTTTAGCTAATTCTTCAATTGATATTATTCTTC
ACGATACTTACTATGTAGTAGCTCATTTTCACTATGTTCTTTCTATAGGAGCTGTA
TTTGCAATTATAGCCGGAATTGTCCAATGATTCCCATTATTTACCGGACTAACCT
TAAATAATTTTTTTTTAAAAACCCAATTTATTACTATATTTTTAGGTGTAAATTTAAC
ATTTTTTCCCCAACACTTTTTAGGGCTAAGAGGAATGCCTCGGCGATATTCGGAT
TATCCAGATGCTTTCACTATCTGAAATGCCATTTCATCTATTGGTTCCATTATTTC
TCTCACAAGAATTATCTTTTTTTTATTTATCTTATGAGAAAGGCTTTCTTCCCAAC
GTAAATCTTTGAGAACTTTAAATGTAACAACTTCAATTGAATGGTTCAAGCCCTAC
CCCCTAGTG
MOC2
ATTATTAGACAAGAAAGGGGGAAAAAAGAAGCTTTTGGACCTTAGGAATAATTTA
TGCCATGATAGCTATCGGATTATTAGGATTCGTAGTTTGAGCTCACCATATATTT
ACAGTAGATAGATGTAGATACCCGAGCTTACTTCACTTCAGCAACTATAATTATT
GCGGTTCCAACTGGGATTAAAGTCTTTAGATGACTAGCCACCTTTCATGGAACT
CAAATTTTAAATAGACCAGTAACCCTGTGAGCTTTAGGTTTTGTATTTTTATTTAC
AGTAGGAGGATTAACAGGGGTAATTTTAGCTAATTCTTCAATTGATATTATTCTTC

ACGATACTTACTATGTAGTAGCTCATTTTCACTATGTTCTTTCTATAGGAGCTGTA
TTTGCAATTATAGCCGGAATTGTCCAATGATTCCCATTATTTACCGGACTAACCT
TAAATAATTTTTTTTTAAAAACCCAATTTATTACTATATTTTTAGGTGTAAATTTAAC
ATTTTTTCCCCAACACTTTTTAGGGCTAAGAGGAATGCCTCGGCGATATTCGGAT
TATCCAGATGCTTTCACTATCTGAAATGCCATTTCATCTATTGGTTCCATTATTTC
TCTCACAAGAATTATCTTTTTTTTTATTTATCTTATGAGAAAGGCTTTCTTCCCAAC
GTAAATCTTTGAGAACTTTAAATGTAACAACTTCAATTGAATGGTTCAAGCCCTAC
CCCCTAGTG
MOC2_0
ATTATTAGACAAGAAAGGGGGAAAAAAGAAGCTTTTGGACCTTAGGAATAATTTA
TGCCATGATAGCTATCGGATTATTAGGATTCGTAGTTTGAGCTCACCATATATTT
ACAGTAGATAGATGTAGATACCCGAGCTTACTTCACTTCAGCAACTATAATTATT
GCGGTTCCAACTGGGATTAAAGTCTTTAGATGACTAGCCACCTTTCATGGAACT
CAAATTTTAAATAGACCAGTAACCCTGTGAGCTTTAGGTTTTGTATTTTTATTTAC
AGTAGGAGGATTAACAGGGGTAATTTTAGCTAATTCTTCAATTGATATTATTCTTC
ACGATACTTACTATGTAGTAGCTCATTTTCACTATGTTCTTTCTATAGGAGCTGTA
TTTGCAATTATAGCCGGAATTGTCCAATGATTCCCATTATTTACCGGACTAACCT
TAAATAATTTTTTTTTAAAAACCCAATTTATTACTATATTTTTAGGTGTAAATTTAAC
ATTTTTTCCCCAACACTTTTTAGGGCTAAGAGGAATGCCTCGGCGATATTCGGAT
TATCCAGATGCTTTCACTATCTGAAATGCCATTTCATCTATTGGTTCCATTATTTC
TCTCACAAGAATTATCTTTTTTTTTATTTATCTTATGAGAAAGGCTTTCTTCCCAAC
GTAAATCTTTGAGAACTTTAAATGTAACAACTTCAATTGAATGGTTCAAGCCCTAC
CCCCTAGTG
MOC2_1
ATTATTAGACAAGAAAGGGGGAAAAAAGAAGCTTTTGGACCTTAGGAATAATTTA
TGCCATGATAGCTATCGGATTATTAGGATTCGTAGTTTGAGCTCACCATATATTT
ACAGTAGATAGATGTAGATACCCGAGCTTACTTCACTTCAGCAACTATAATTATT
GCGGTTCCAACTGGGATTAAAGTCTTTAGATGACTAGCCACCTTTCATGGAACT
CAAATTTTAAATAGACCAGTAACCCTGTGAGCTTTAGGTTTTGTATTTTTATTTAC
AGTAGGAGGATTAACAGGGGTAATTTTAGCTAATTCTTCAATTGATATTATTCTTC
ACGATACTTACTATGTAGTAGCTCATTTTCACTATGTTCTTTCTATAGGAGCTGTA
TTTGCAATTATAGCCGGAATTGTCCAATGATTCCCATTATTTACCGGACTAACCT
TAAATAATTTTTTTTTAAAAACCCAATTTATTACTATATTTTTAGGTGTAAATTTAAC
ATTTTTTCCCCAACACTTTTTAGGGCTAAGAGGAATGCCTCGGCGATATTCGGAT
TATCCAGATGCTTTCACTATCTGAAATGCCATTTCATCTATTGGTTCCATTATTTC
TCTCACAAGAATTATCTTTTTTTTTATTTATCTTATGAGAAAGGCTTTCTTCCCAAC
GTAAATCTTTGAGAACTTTAAATGTAACAACTTCAATTGAATGGTTCAAGCCCTAC
CCCCTAGTG
MOC3
ATTATTAGACAAGAAAGGGGGAAAAAAGAAGCTTTTGGACCTTAGGAATAATTTA
TGCCATGATAGCTATCGGATTATTAGGATTCGTAGTTTGAGCTCACCATATATTT
ACAGTAGATAGATGTAGATACCCGAGCTTACTTCACTTCAGCAACTATAATTATT
GCGGTTCCAACTGGGATTAAAGTCTTTAGATGACTAGCCACCTTTCATGGAACT
CAAATTTTAAATAGACCAGTAACCCTGTGAGCTTTAGGTTTTGTATTTTTATTTAC
AGTAGGAGGATTAACAGGGGTAATTTTAGCTAATTCTTCAATTGATATTATTCTTC
ACGATACTTACTATGTAGTAGCTCATTTTCACTATGTTCTTTCTATAGGAGCTGTA
TTTGCAATTATAGCCGGAATTGTCCAATGATTCCCATTATTTACCGGACTAACCT
TAAATAATTTTTTTTTAAAAACCCAATTTATTACTATATTTTTAGGTGTAAATTTAAC
ATTTTTTCCCCAACACTTTTTAGGGCTAAGAGGAATGCCTCGGCGATATTCGGAT
TATCCAGATGCTTTCACTATCTGAAATGCCATTTCATCTATTGGTTCCATTATTTC

TCTCACAAGAATTATCTTTTTTTTATTTATCTTATGAGAAAGGCTTTCTTCCCAAC
GTAAATCTTTGAGAACTTTAAATGTAACAACTTCAATTGAATGGTTCAAGCCCTAC
CCCCTAGTG
MOC4
ATTATTAGACAAGAAAGGGGGAAAAAAGAAGCTTTTGGACCTTAGGAATAATTTA
TGCCATGATAGCTATCGGATTATTAGGATTCGTAGTTTGAGCTCACCATATATTT
ACAGTAGATAGATGTAGATACCCGAGCTTACTTCACTTCAGCAACTATAATTATT
GCGGTTCCAACTGGGATTAAAGTCTTTAGATGACTAGCCACCTTTCATGGAACT
CAAATTTTAAATAGACCAGTAACCCTGTGAGCTTTAGGTTTTGTATTTTTATTTAC
AGTAGGAGGATTAACAGGGGTAATTTTAGCTAATTCTTCAATTGATATTATTCTTC
ACGATACTTACTATGTAGTAGCTCATTTTCACTATGTTCTTTCTATAGGAGCTGTA
TTTGCAATTATAGCCGGAATTGTCCAATGATTCCCATTATTTACCGGACTAACCT
TAAATAATTTTTTTTTAAAAACCCAATTTATTACTATATTTTTAGGTGTAAATTTAAC
ATTTTTTCCCCAACACTTTTTAGGGCTAAGAGGAATGCCTCGGCGATATTCGGAT
TATCCAGATGCTTTCACTATCTGAAATGCCATTTCATCTATTGGTTCCATTATTTC
TCTCACAAGAATTATCTTTTTTTTATTTATCTTATGAGAAAGGCTTTCTTCCCAAC
GTAAATCTTTGAGAACTTTAAATGTAACAACTTCAATTGAATGGTTCAAGCCCTAC
CCCCTAGTG
moc5
ATTATTAGACAAGAAAGGGGGAAAAAAGAAGCTTTTGGACCTTAGGAATAATTTA
TGCCATGATAGCTATCGGATTATTAGGATTCGTAGTTTGAGCTCACCATATATTT
ACAGTAGATAGATGTAGATACCCGAGCTTACTTCACTTCAGCAACTATAATTATT
GCGGTTCCAACTGGGATTAAAGTCTTTAGATGACTAGCCACCTTTCATGGAACT
CAAATTTTAAATAGACCAGTAACCCTGTGAGCTTTAGGTTTTGTATTTTTATTTAC
AGTAGGAGGATTAACAGGGGTAATTTTAGCTAATTCTTCAATTGATATTATTCTTC
ACGATACTTACTATGTAGTAGCTCATTTTCACTATGTTCTTTCTATAGGAGCTGTA
TTTGCAATTATAGCCGGAATTGTCCAATGATTCCCATTATTTACCGGACTAACCT
TAAATAATTTTTTTTTAAAAACCCAATTTATTACTATATTTTTAGGTGTAAATTTAAC
ATTTTTTCCCCAACACTTTTTAGGGCTAAGAGGAATGCCTCGGCGATATTCGGAT
TATCCAGATGCTTTCACTATCTGAAATGCCATTTCATCTATTGGTTCCATTATTTC
TCTCACAAGAATTATCTTTTTTTTATTTATCTTATGAGAAAGGCTTTCTTCCCAAC
GTAAATCTTTGAGAACTTTAAATGTAACAACTTCAATTGAATGGTTCAAGCCCTAC
CCCCTAGTG
pit1
ATTATTAGACAAGAAAGGGGGAAAAAAGAAGCTTTTGGACCTTAGGAATAATTTA
TGCCATGATAGCTATCGGATTATTAGGATTCGTAGTTTGAGCTCACCATATATTT
ACAGTAGATAGATGTAGATACCCGAGCTTACTTCACTTCAGCAACTATAATTATC
GCGGTTCCAACTGGGATTAAAGTCTTTAGGTGACTAGCCACCTTTCATGGAACT
CAAATTTTAAATAGACCAGTAACCCTGTGAGCTTTAGGTTTTGTATTTTTATTTAC
AGTAGGAGGATTAACAGGGGTAATTTTAGCTAATTCTTCAATTGATATTATTCTTC
ACGATACTTACTATGTAGTAGCTCATTTTCACTATGTTCTTTCTATAGGAGCTGTA
TTTGCAATTATAGCCGGAATTGTCCAATGATTCCCATTATTTACTGGACTAACCTT
AAATAATTTTTTTTTAAAAACCCAATTTATTACTATATTTTTAGGTGTAAATTTAACA
TTTTTTCCTCAACACTTTTTAGGGCTAAGAGGAATGCCTCGGCGATATTCGGATT
ATCCAGATGCTTTCACTATCTGAAATGCCATTTCATCTATTGGTTCCATTATTTCT
CTCACAAGAATTATCTTTTTTTTATTTATCTTATGAGAAAGGCTTTCTTCCCAACG
TAAATCTTTGAGAACTTTAAATGTAACAACTTCAATTGAATGGTTCAAGCCCTACC
CCCTAGTG
PIT1_1
ATTATTAGACAAGAAAGGGGGAAAAAAGAAGCTTTTGGACCTTAGGAATAATTTA

TGCCATGATAGCTATCGGATTATTAGGATTCGTAGTTTGAGCTCACCATATATTT
ACAGTAGATAGATGTAGATACCCGAGCTTACTTCACTTCAGCAACTATAATTATC
GCGGTTCCAACTGGGATTAAAGTCTTTAGGTGACTAGCCACCTTTCATGGAACT
CAAATTTTAAATAGACCAGTAACCCTGTGAGCTTTAGGTTTTGTATTTTTATTTAC
AGTAGGAGGATTAACAGGGGTAATTTTAGCTAATTCTTCAATTGATATTATTCTTC
ACGATACTTACTATGTAGTAGCTCATTTTCACTATGTTCTTTCTATAGGAGCTGTA
TTTGCAATTATAGCCGGAATTGTCCAATGATTCCCATTATTTACTGGACTAACCTT
AAATAATTTTTTTTTAAAAACCCAATTTATTACTATATTTTTAGGTGTAAATTTAACA
TTTTTTCCTCAACACTTTTTAGGGCTAAGAGGAATGCCTCGGCGATATTCGGATT
ATCCAGATGCTTTCACTATCTGAAATGCCATTTCATCTATTGGTTCCATTATTTCT
CTCACAAGAATTATCTTTTTTTTTATTTATCTTATGAGAAAGGCTTTCTTCCCAACG
TAAATCTTTGAGAACTTTAAATGTAACAACTTCAATTGAATGGTTCAAGCCCTACC
CCCTAGTG
PIT1_2
ATTATTAGACAAGAAAGGGGGAAAAAAGAAGCTTTTGGACCTTAGGAATAATTTA
TGCCATGATAGCTATCGGATTATTAGGATTCGTAGTTTGAGCTCACCATATATTT
ACAGTAGATAGATGTAGATACCCGAGCTTACTTCACTTCAGCAACTATAATTATT
GCGGTTCCAACTGGGATTAAAGTCTTTAGATGACTAGCCACCTTTCATGGAACT
CAAATTTTAAATAGACCAGTAACCCTGTGAGCTTTAGGTTTTGTATTTTTATTTAC
AGTAGGAGGATTAACAGGGGTAATTTTAGCTAATTCTTCAATTGATATTATTCTTC
ACGATACTTACTATGTAGTAGCTCATTTTCACTATGTTCTTTCTATAGGAGCTGTA
TTTGCAATTATAGCCGGAATTGTCCAATGATTCCCATTATTTACTGGACTAACCTT
AAATAATTTTTTTTTAAAAACCCAATTTATTACTATATTTTTAGGTGTAAATTTAACA
TTTTTTCCTCAACACTTTTTAGGGCTAAGAGGAATGCCTCGGCGATATTCGGATT
ATCCAGATGCTTTCACTATCTGAAATGCCATTTCATCTATTGGTTCCATTATTTCT
CTCACAAGAATTATCTTTTTTTTTATTTATCTTATGAGAAAGGCTTTCTTCCCAACG
TAAATCTTTGAGAACTTTAAATGTAACAACTTCAATTGAATGGTTCAAGCCCTACC
CCCTAGTG
pit2
ATTATTAGACAAGAAAGGGGGAAAAAAGAAGCTTTTGGACCTTAGGAATAATTTA
TGCCATGATAGCTATCGGATTATTAGGATTCGTAGTTTGAGCTCACCATATATTT
ACAGTAGATAGATGTAGATACCCGAGCTTACTTCACTTCAGCAACTATAATTATC
GCGGTTCCAACTGGGATTAAAGTCTTTAGGTGACTAGCCACCTTTCATGGAACT
CAAATTTTAAATAGACCAGTAACCCTGTGAGCTTTAGGTTTTGTATTTTTATTTAC
AGTAGGAGGATTAACAGGGGTAATTTTAGCTAATTCTTCAATTGATATTATTCTTC
ACGATACTTACTATGTAGTAGCTCATTTTCACTATGTTCTTTCTATAGGAGCTGTA
TTTGCAATTATAGCCGGAATTGTCCAATGATTCCCATTATTTACTGGACTAACCTT
AAATAATTTTTTTTTAAAAACCCAATTTATTACTATATTTTTAGGTGTAAATTTAACA
TTTTTTCCTCAACACTTTTTAGGGCTAAGAGGAATGCCTCGGCGATATTCGGATT
ATCCAGATGCTTTCACTATCTGAAATGCCATTTCATCTATTGGTTCCATTATTTCT
CTCACAAGAATTATCTTTTTTTTTATTTATCTTATGAGAAAGGCTTTCTTCCCAACG
TAAATCTTTGAGAACTTTAAATGTAACAACTTCAATTGAATGGTTCAAGCCCTACC
CCCTAGTG
pit4
ATTATTAGACAAGAAAGGGGGAAAAAAGAAGCTTTTGGACCTTAGGAATAATTTA
TGCCATGATAGCTATCGGATTATTAGGATTCGTAGTTTGAGCTCACCATATATTT
ACAGTAGATAGATGTAGATACCCGAGCTTACTTCACTTCAGCAACTATAATTATT
GCGGTTCCAACTGGGATTAAAGTCTTTAGATGACTAGCCACCTTTCATGGAACT
CAAATTTTAAATAGACCAGTAACCCTGTGAGCTTTAGGTTTTGTATTTTTATTTAC
AGTAGGAGGATTAACAGGGGTAATTTTAGCTAATTCTTCAATTGATATTATTCTTC

ACGATACTTACTATGTAGTAGCTCATTTTCACTATGTTCTTTCTATAGGAGCTGTA
TTTGCAATTATAGCCGGAATTGTCCAATGATTCCCATTATTTACTGGACTAACCTT
AAATAATTTTTTTTTAAAAACCCAATTTATTACTATATTTTTAGGTGTAAATTTAACA
TTTTTTCCTCAACACTTTTTAGGGCTAAGAGGAATGCCTCGGCGATATTCGGATT
ATCCAGATGCTTTCACTATCTGAAATGCCATTTCATCTATTGGTTCCATTATTTCT
CTCACAAGAATTATCTTTTTTTTATTTATCTTATGAGAAAGGCTTTCTTCCCAACG
TAAATCTTTGAGAACTTTAAATGTAACAACTTCAATTGAATGGTTCAAGCCCTACC
CCCTAGTG
RON1
ATTATTAGACAAGAAAGGGGGAAAAAAGAAGCTTTTGGACCTTAGGAATAATTTA
TGCCATGATAGCTATCGGATTATTAGGATTCGTAGTTTGAGCTCACCATATATTT
ACAGTAGATAGATGTAGATACCCGAGCTTACTTCACTTCAGCAACTATAATTATT
GCGGTTCCAACTGGGATTAAAGTCTTTAGATGACTAGCCACCTTTCATGGAACT
CAAATTTTAAATAGACCAGTAACCCTGTGAGCTTTAGGTTTTGTATTTTTATTTAC
AGTAGGAGGATTAACAGGGGTAATTTTAGCTAATTCTTCAATTGATATTATTCTTC
ACGATACTTACTATGTAGTAGCTCATTTTCACTATGTTCTTTCTATAGGAGCTGTA
TTTGCAATTATAGCCGGAATTGTCCAATGATTCCCATTATTTACCGGACTAACCT
TAAATAATTTTTTTTTAAAAACCCAATTTATTACTATATTTTTAGGTGTAAATTTAAC
ATTTTTTCCCCAACACTTTTTAGGGCTAAGAGGAATGCCTCGGCGATATTCGGAT
TATCCAGATGCTTTCACTATCTGAAATGCCATTTCATCTATTGGTTCCATTATTTC
TCTCACAAGAATTATCTTTTTTTTATTTATCTTATGAGAAAGGCTTTCTTCCCAAC
GTAAATCTTTGAGAACTTTAAATGTAACAACTTCAATTGAATGGTTCAAGCCCTAC
CCCCTAGTG
RON2
ATTATTAGACAAGAAAGGGGGAAAAAAGAAGCTTTTGGACCTTAGGAATAATTTA
TGCCATGATAGCTATCGGATTATTAGGATTCGTAGTTTGAGCTCACCATATATTT
ACAGTAGATAGATGTAGATACCCGAGCTTACTTCACTTCAGCAACTATAATTATT
GCGGTTCCAACTGGGATTAAAGTCTTTAGATGACTAGCCACCTTTCATGGAACT
CAAATTTTAAATAGACCAGTAACCCTGTGAGCTTTAGGTTTTGTATTTTTATTTAC
AGTAGGAGGATTAACAGGGGTAATTTTAGCTAATTCTTCAATTGATATTATTCTTC
ACGATACTTACTATGTAGTAGCTCATTTTCACTATGTTCTTTCTATAGGAGCTGTA
TTTGCAATTATAGCCGGAATTGTCCAATGATTCCCATTATTTACCGGACTAACCT
TAAATAATTTTTTTTTAAAAACCCAATTTATTACTATATTTTTAGGTGTAAATTTAAC
ATTTTTTCCCCAACACTTTTTAGGGCTAAGAGGAATGCCTCGGCGATATTCGGAT
TATCCAGATGCTTTCACTATCTGAAATGCCATTTCATCTATTGGTTCCATTATTTC
TCTCACAAGAATTATCTTTTTTTTATTTATCTTATGAGAAAGGCTTTCTTCCCAAC
GTAAATCTTTGAGAACTTTAAATGTAACAACTTCAATTGAATGGTTCAAGCCCTAC
CCCCTAGTG
ron3
ATTATTAGACAAGAAAGGGGGAAAAAAGAAGCTTTTGGACCTTAGGAATAATTTA
TGCCATGATAGCTATCGGATTATTAGGATTCGTAGTTTGAGCTCACCATATATTT
ACAGTAGATAGATGTAGATACCCGAGCTTACTTCACTTCAGCAACTATAATTATT
GCGGTTCCAACTGGGATTAAAGTCTTTAGATGACTAGCCACCTTTCATGGAACT
CAAATTTTAAATAGACCAGTAACCCTGTGAGCTTTAGGTTTTGTATTTTTATTTAC
AGTAGGAGGATTAACAGGGGTAATTTTAGCTAATTCTTCAATTGATATTATTCTTC
ACGATACTTACTATGTAGTAGCTCATTTTCACTATGTTCTTTCTATAGGAGCTGTA
TTTGCAATTATAGCCGGAATTGTCCAATGATTCCCATTATTTACCGGACTAACCT
TAAATAATTTTTTTTTAAAAACCCAATTTATTACTATATTTTTAGGTGTAAATTTAAC
ATTTTTTCCCCAACACTTTTTAGGGCTAAGAGGAATGCCTCGGCGATATTCGGAT
TATCCAGATGCTTTCACTATCTGAAATGCCATTTCATCTATTGGTTCCATTATTTC

TCTCACAAGAATTATCTTTTTTTTATTTATCTTATGAGAAAGGCTTTCTTCCCAAC
GTAAATCTTTGAGAACTTTAAATGTAACAACTTCAATTGAATGGTTCAAGCCCTAC
CCCCTAGTG
RON4
ATTATTAGACAAGAAAGGGGGAAAAAAGAAGCTTTTGGACCTTAGGAATAATTTA
TGCCATGATAGCTATCGGATTATTAGGATTCGTAGTTTGAGCTCACCATATATTT
ACAGTAGATAGATGTAGATACCCGAGCTTACTTCACTTCAGCAACTATAATTATT
GCGGTTCCAACTGGGATTAAAGTCTTTAGATGACTAGCCACCTTTCATGGAACT
CAAATTTTAAATAGACCAGTAACCCTGTGAGCTTTAGGTTTTGTATTTTTATTTAC
AGTAGGAGGATTAACAGGGGTAATTTTAGCTAATTCTTCAATTGATATTATTCTTC
ACGATACTTACTATGTAGTAGCTCATTTTCACTATGTTCTTTCTATAGGAGCTGTA
TTTGCAATTATAGCCGGAATTGTCCAATGATTCCCATTATTTACCGGACTAACCT
TAAATAATTTTTTTTTAAAAACCCAATTTATTACTATATTTTTAGGTGTAAATTTAAC
ATTTTTTCCCCAACACTTTTTAGGGCTAAGAGGAATGCCTCGGCGATATTCGGAT
TATCCAGATGCTTTCACTATCTGAAATGCCATTTCATCTATTGGTTCCATTATTTC
TCTCACAAGAATTATCTTTTTTTTATTTATCTTATGAGAAAGGCTTTCTTCCCAAC
GTAAATCTTTGAGAACTTTAAATGTAACAACTTCAATTGAATGGTTCAAGCCCTAC
CCCCTAGTG
RON7
ATTATTAGACAAGAAAGGGGGAAAAAAGAAGCTTTTGGACCTTAGGAATAATTTA
TGCCATGATAGCTATCGGATTATTAGGATTCGTAGTTTGAGCTCACCATATATTT
ACAGTAGATAGATGTAGATACCCGAGCTTACTTCACTTCAGCAACTATAATTATT
GCGGTTCCAACTGGGATTAAAGTCTTTAGATGACTAGCCACCTTTCATGGAACT
CAAATTTTAAATAGACCAGTAACCCTGTGAGCTTTAGGTTTTGTATTTTTATTTAC
AGTAGGAGGATTAACAGGGGTAATTTTAGCTAATTCTTCAATTGATATTATTCTTC
ACGATACTTACTATGTAGTAGCTCATTTTCACTATGTTCTTTCTATAGGAGCTGTA
TTTGCAATTATAGCCGGAATTGTCCAATGATTCCCATTATTTACCGGACTAACCT
TAAATAATTTTTTTTTAAAAACCCAATTTATTACTATATTTTTAGGTGTAAATTTAAC
ATTTTTTCCCCAACACTTTTTAGGGCTAAGAGGAATGCCTCGGCGATATTCGGAT
TATCCAGATGCTTTCACTATCTGAAATGCCATTTCATCTATTGGTTCCATTATTTC
TCTCACAAGAATTATCTTTTTTTTATTTATCTTATGAGAAAGGCTTTCTTCCCAAC
GTAAATCTTTGAGAACTTTAAATGTAACAACTTCAATTGAATGGTTCAAGCCCTAC
CCCCTAGTG
SGO1
ATTATTAGACAAGAAAGGGGGAAAAAAGAAGCTTTTGGACCTTAGGAATAATTTA
TGCCATGATAGCTATCGGATTATTAGGATTCGTAGTTTGAGCTCACCATATATTT
ACAGTAGATAGATGTAGATACCCGAGCTTACTTCACTTCAGCAACTATAATTATT
GCGGTTCCAACTGGGATTAAAGTCTTTAGATGACTAGCCACCTTTCATGGAACT
CAAATTTTAAATAGACCAGTAACCCTGTGAGCTTTAGGTTTTGTATTTTTATTTAC
AGTAGGAGGATTAACAGGGGTAATTTTAGCTAATTCTTCAATTGATATTATTCTTC
ACGATACTTACTATGTAGTAGCTCATTTTCACTATGTTCTTTCTATAGGAGCTGTA
TTTGCAATTATAGCCGGAATTGTCCAATGATTCCCATTATTTACCGGACTAACCT
TAAATAATTTTTTTTTAAAAACCCAATTTATTACTATATTTTTAGGTGTAAATTTAAC
ATTTTTTCCCCAACACTTTTTAGGGCTAAGAGGAATGCCTCGGCGATATTCGGAT
TATCCAGATGCTTTCACTATCTGAAATGCCATTTCATCTATTGGTTCCATTATTTC
TCTCACAAGAATTATCTTTTTTTTATTTATCTTATGAGAAAGGCTTTCTTCCCAAC
GTAAATCTTTGAGAACTTTAAATGTAACAACTTCAATTGAATGGTTCAAGCCCTAC
CCCCTAGTG
SGO2
ATTATTAGACAAGAAAGGGGGAAAAAAGAAGCTTTTGGACCTTAGGAATAATTTA

TGCCATGATAGCTATCGGATTATTAGGATTCGTAGTTTGAGCTCACCATATATTT
ACAGTAGATAGATGTAGATACCCGAGCTTACTTCACTTCAGCAACTATAATTATT
GCGGTTCCAACTGGGATTAAAGTCTTTAGATGACTAGCCACCTTTCATGGAACT
CAAATTTTAAATAGACCAGTAACCCTGTGAGCTTTAGGTTTTGTATTTTTATTTAC
AGTAGGAGGATTAACAGGGGTAATTTTAGCTAATTCTTCAATTGATATTATTCTTC
ACGATACTTACTATGTAGTAGCTCATTTTCACTATGTTCTTTCTATAGGAGCTGTA
TTTGCAATTATAGCCGGAATTGTCCAATGATTCCCATTATTTACCGGACTAACCT
TAAATAATTTTTTTTTAAAAACCCAATTTATTACTATATTTTTAGGTGTAAATTTAAC
ATTTTTTCCCCAACACTTTTTAGGGCTAAGAGGAATGCCTCGGCGATATTCGGAT
TATCCAGATGCTTTCACTATCTGAAATGCCATTTCATCTATTGGTTCCATTATTTC
TCTCACAAGAATTATCTTTTTTTTATTTATCTTATGAGAAAGGCTTTCTTCCCAAC
GTAAATCTTTGAGAACTTTAAATGTAACAACTTCAATTGAATGGTTCAAGCCCTAC
CCCCTAGTG
SJA12
ATTATTAGACAAGAAAGGGGGAAAAAAGAAGCTTTTGGACCTTAGGAATAATTTA
TGCCATGATAGCTATCGGATTATTAGGATTCGTAGTTTGAGCTCACCATATATTT
ACAGTAGATAGATGTAGATACCCGAGCTTACTTCACTTCAGCAACTATAATTATT
GCGGTTCCAACTGGGATTAAAGTCTTTAGATGACTAGCCACCTTTCATGGAACT
CAAATTTTAAATAGACCAGTAACCCTGTGAGCTTTAGGTTTTGTATTTTTATTTAC
AGTAGGAGGATTAACAGGGGTAATTTTAGCTAATTCTTCAATTGATATTATTCTTC
ACGATACTTACTATGTAGTAGCTCATTTTCACTATGTTCTTTCTATAGGAGCTGTA
TTTGCAATTATAGCCGGAATTGTCCAATGATTCCCATTATTTACCGGACTAACCT
TAAATAATTTTTTTTTAAAAACCCAATTTATTACTATATTTTTAGGTGTAAATTTAAC
ATTTTTTCCCCAACACTTTTTAGGGCTAAGAGGAATGCCTCGGCGATATTCGGAT
TATCCAGATGCTTTCACTATCTGAAATGCCATTTCATCTATTGGTTCCATTATTTC
TCTCACAAGAATTATCTTTTTTTTATTTATCTTATGAGAAAGGCTTTCTTCCCAAC
GTAAATCTTTGAGAACTTTAAATGTAACAACTTCAATTGAATGGTTCAAGCCCTAC
CCCCTAGTG
SJA13
ATTATTAGACAAGAAAGGGGGAAAAAAGAAGCTTTTGGACCTTAGGAATAATTTA
TGCCATGATAGCTATCGGATTATTAGGATTCGTAGTTTGAGCTCACCATATATTT
ACAGTAGATAGATGTAGATACCCGAGCTTACTTCACTTCAGCAACTATAATTATT
GCGGTTCCAACTGGGATTAAAGTCTTTAGATGACTAGCCACCTTTCATGGAACT
CAAATTTTAAATAGACCAGTAACCCTGTGAGCTTTAGGTTTTGTATTTTTATTTAC
AGTAGGAGGATTAACAGGGGTAATTTTAGCTAATTCTTCAATTGATATTATTCTTC
ACGATACTTACTATGTAGTAGCTCATTTTCACTATGTTCTTTCTATAGGAGCTGTA
TTTGCAATTATAGCCGGAATTGTCCAATGATTCCCATTATTTACCGGACTAACCT
TAAATAATTTTTTTTTAAAAACCCAATTTATTACTATATTTTTAGGTGTAAATTTAAC
ATTTTTTCCCCAACACTTTTTAGGGCTAAGAGGAATGCCTCGGCGATATTCGGAT
TATCCAGATGCTTTCACTATCTGAAATGCCATTTCATCTATTGGTTCCATTATTTC
TCTCACAAGAATTATCTTTTTTTTATTTATCTTATGAGAAAGGCTTTCTTCCCAAC
GTAAATCTTTGAGAACTTTAAATGTAACAACTTCAATTGAATGGTTCAAGCCCTAC
CCCCTAGTG
SJA14
ATTATTAGACAAGAAAGGGGGAAAAAAGAAGCTTTTGGACCTTAGGAATAATTTA
TGCCATGATAGCTATCGGATTATTAGGATTCGTAGTTTGAGCTCACCATATATTT
ACAGTAGATAGATGTAGATACCCGAGCTTACTTCACTTCAGCAACTATAATTATT
GCGGTTCCAACTGGGATTAAAGTCTTTAGATGACTAGCCACCTTTCATGGAACT
CAAATTTTAAATAGACCAGTAACCCTGTGAGCTTTAGGTTTTGTATTTTTATTTAC
AGTAGGAGGATTAACAGGGGTAATTTTAGCTAATTCTTCAATTGATATTATTCTTC

ACGATACTTACTATGTAGTAGCTCATTTTCACTATGTTCTTTCTATAGGAGCTGTA
TTTGCAATTATAGCCGGAATTGTCCAATGATTCCCATTATTTACCGGACTAACCT
TAAATAATTTTTTTTTAAAAACCCAATTTATTACTATATTTTTAGGTGTAAATTTAAC
ATTTTTTCCCCAACACTTTTTAGGGCTAAGAGGAATGCCTCGGCGATATTCGGAT
TATCCAGATGCTTTCACTATCTGAAATGCCATTTCATCTATTGGTTCCATTATTTC
TCTCACAAGAATTATCTTTTTTTTATTTATCTTATGAGAAAGGCTTTCTTCCCAAC
GTAAATCTTTGAGAACTTTAAATGTAACAACTTCAATTGAATGGTTCAAGCCCTAC
CCCCTAGTG
SJA17
ATTATTAGACAAGAAAGGGGGAAAAAAGAAGCTTTTGGACCTTAGGAATAATTTA
TGCCATGATAGCTATCGGATTATTAGGATTCGTAGTTTGAGCTCACCATATATTT
ACAGTAGATAGATGTAGATACCCGAGCTTACTTCACTTCAGCAACTATAATTATT
GCGGTTCCAACTGGGATTAAAGTCTTTAGATGACTAGCCACCTTTCATGGAACT
CAAATTTTAAATAGACCAGTAACCCTGTGAGCTTTAGGTTTTGTATTTTTATTTAC
AGTAGGAGGATTAACAGGGGTAATTTTAGCTAATTCTTCAATTGATATTATTCTTC
ACGATACTTACTATGTAGTAGCTCATTTTCACTATGTTCTTTCTATAGGAGCTGTA
TTTGCAATTATAGCCGGAATTGTCCAATGATTCCCATTATTTACCGGACTAACCT
TAAATAATTTTTTTTTAAAAACCCAATTTATTACTATATTTTTAGGTGTAAATTTAAC
ATTTTTTCCCCAACACTTTTTAGGGCTAAGAGGAATGCCTCGGCGATATTCGGAT
TATCCAGATGCTTTCACTATCTGAAATGCCATTTCATCTATTGGTTCCATTATTTC
TCTCACAAGAATTATCTTTTTTTTATTTATCTTATGAGAAAGGCTTTCTTCCCAAC
GTAAATCTTTGAGAACTTTAAATGTAACAACTTCAATTGAATGGTTCAAGCCCTAC
CCCCTAGTG
SJA3
ATTATTAGACAAGAAAGGGGGAAAAAAGAAGCTTTTGGACCTTAGGAATAATTTA
TGCCATGATAGCTATCGGATTATTAGGATTCGTAGTTTGAGCTCACCATATATTT
ACAGTAGATAGATGTAGATACCCGAGCTTACTTCACTTCAGCAACTATAATTATT
GCGGTTCCAACTGGGATTAAAGTCTTTAGATGACTAGCCACCTTTCATGGAACT
CAAATTTTAAATAGACCAGTAACCCTGTGAGCTTTAGGTTTTGTATTTTTATTTAC
AGTAGGAGGATTAACAGGGGTAATTTTAGCTAATTCTTCAATTGATATTATTCTTC
ACGATACTTACTATGTAGTAGCTCATTTTCACTATGTTCTTTCTATAGGAGCTGTA
TTTGCAATTATAGCCGGAATTGTCCAATGATTCCCATTATTTACCGGACTAACCT
TAAATAATTTTTTTTTAAAAACCCAATTTATTACTATATTTTTAGGTGTAAATTTAAC
ATTTTTTCCCCAACACTTTTTAGGGCTAAGAGGAATGCCTCGGCGATATTCGGAT
TATCCAGATGCTTTCACTATCTGAAATGCCATTTCATCTATTGGTTCCATTATTTC
TCTCACAAGAATTATCTTTTTTTTATTTATCTTATGAGAAAGGCTTTCTTCCCAAC
GTAAATCTTTGAGAACTTTAAATGTAACAACTTCAATTGAATGGTTCAAGCCCTAC
CCCCTAGTG
SJA4
ATTATTAGACAAGAAAGGGGGAAAAAAGAAGCTTTTGGACCTTAGGAATAATTTA
TGCCATGATAGCTATCGGATTATTAGGATTCGTAGTTTGAGCTCACCATATATTT
ACAGTAGATAGATGTAGATACCCGAGCTTACTTCACTTCAGCAACTATAATTATT
GCGGTTCCAACTGGGATTAAAGTCTTTAGATGACTAGCCACCTTTCATGGAACT
CAAATTTTAAATAGACCAGTAACCCTGTGAGCTTTAGGTTTTGTATTTTTATTTAC
AGTAGGAGGATTAACAGGGGTAATTTTAGCTAATTCTTCAATTGATATTATTCTTC
ACGATACTTACTATGTAGTAGCTCATTTTCACTATGTTCTTTCTATAGGAGCTGTA
TTTGCAATTATAGCCGGAATTGTCCAATGATTCCCATTATTTACCGGACTAACCT
TAAATAATTTTTTTTTAAAAACCCAATTTATTACTATATTTTTAGGTGTAAATTTAAC
ATTTTTTCCCCAACACTTTTTAGGGCTAAGAGGAATGCCTCGGCGATATTCGGAT
TATCCAGATGCTTTCACTATCTGAAATGCCATTTCATCTATTGGTTCCATTATTTC

TCTCACAAGAATTATCTTTTTTTTATTTATCTTATGAGAAAGGCTTTCTTCCCAAC
GTAAATCTTTGAGAACTTTAAATGTAACAACTTCAATTGAATGGTTCAAGCCCTAC
CCCCTAGTG
SJA5
ATTATTAGACAAGAAAGGGGGAAAAAAGAAGCTTTTGGACCTTAGGAATAATTTA
TGCCATGATAGCTATCGGATTATTAGGATTCGTAGTTTGAGCTCACCATATATTT
ACAGTAGATAGATGTAGATACCCGAGCTTACTTCACTTCAGCAACTATAATTATT
GCGGTTCCAACTGGGATTAAAGTCTTTAGATGACTAGCCACCTTTCATGGAACT
CAAATTTTAAATAGACCAGTAACCCTGTGAGCTTTAGGTTTTGTATTTTTATTTAC
AGTAGGAGGATTAACAGGGGTAATTTTAGCTAATTCTTCAATTGATATTATTCTTC
ACGATACTTACTATGTAGTAGCTCATTTTCACTATGTTCTTTCTATAGGAGCTGTA
TTTGCAATTATAGCCGGAATTGTCCAATGATTCCCATTATTTACCGGACTAACCT
TAAATAATTTTTTTTTAAAAACCCAATTTATTACTATATTTTTAGGTGTAAATTTAAC
ATTTTTTCCCCAACACTTTTTAGGGCTAAGAGGAATGCCTCGGCGATATTCGGAT
TATCCAGATGCTTTCACTATCTGAAATGCCATTTCATCTATTGGTTCCATTATTTC
TCTCACAAGAATTATCTTTTTTTTATTTATCTTATGAGAAAGGCTTTCTTCCCAAC
GTAAATCTTTGAGAACTTTAAATGTAACAACTTCAATTGAATGGTTCAAGCCCTAC
CCCCTAGTG
SMA1
ATTATTAGACAAGAAAGGGGGAAAAAAGAAGCTTTTGGACCTTAGGAATAATTTA
TGCCATGATAGCTATCGGATTATTAGGATTCGTAGTTTGAGCTCACCATATATTT
ACAGTAGATAGATGTAGATACCCGAGCTTACTTCACTTCAGCAACTATAATTATT
GCGGTTCCAACTGGGATTAAAGTCTTTAGATGACTAGCCACCTTTCATGGAACT
CAAATTTTAAATAGACCAGTAACCCTGTGAGCTTTAGGTTTTGTATTTTTATTTAC
AGTAGGAGGATTAACAGGGGTAATTTTAGCTAATTCTTCAATTGATATTATTCTTC
ACGATACTTACTATGTAGTAGCTCATTTTCACTATGTTCTTTCTATAGGAGCTGTA
TTTGCAATTATAGCCGGAATTGTCCAATGATTCCCATTATTTACCGGACTAACCT
TAAATAATTTTTTTTTAAAAACCCAATTTATTACTATATTTTTAGGTGTAAATTTAAC
ATTTTTTCCCCAACACTTTTTAGGGCTAAGAGGAATGCCTCGGCGATATTCGGAT
TATCCAGATGCTTTCACTATCTGAAATGCCATTTCATCTATTGGTTCCATTATTTC
TCTCACAAGAATTATCTTTTTTTTATTTATCTTATGAGAAAGGCTTTCTTCCCAAC
GTAAATCTTTGAGAACTTTAAATGTAACAACTTCAATTGAATGGTTCAAGCCCTAC
CCCCTAGTG
SMA10
ATTATTAGACAAGAAAGGGGGAAAAAAGAAGCTTTTGGACCTTAGGAATAATTTA
TGCCATGATAGCTATCGGATTATTAGGATTCGTAGTTTGAGCTCACCATATATTT
ACAGTAGATAGATGTAGATACCCGAGCTTACTTCACTTCAGCAACTATAATTATT
GCGGTTCCAACTGGGATTAAAGTCTTTAGATGACTAGCCACCTTTCATGGAACT
CAAATTTTAAATAGACCAGTAACCCTGTGAGCTTTAGGTTTTGTATTTTTATTTAC
AGTAGGAGGATTAACAGGGGTAATTTTAGCTAATTCTTCAATTGATATTATTCTTC
ACGATACTTACTATGTAGTAGCTCATTTTCACTATGTTCTTTCTATAGGAGCTGTA
TTTGCAATTATAGCCGGAATTGTCCAATGATTCCCATTATTTACCGGACTAACCT
TAAATAATTTTTTTTTAAAAACCCAATTTATTACTATATTTTTAGGTGTAAATTTAAC
ATTTTTTCCCCAACACTTTTTAGGGCTAAGAGGAATGCCTCGGCGATATTCGGAT
TATCCAGATGCTTTCACTATCTGAAATGCCATTTCATCTATTGGTTCCATTATTTC
TCTCACAAGAATTATCTTTTTTTTATTTATCTTATGAGAAAGGCTTTCTTCCCAAC
GTAAATCTTTGAGAACTTTAAATGTAACAACTTCAATTGAATGGTTCAAGCCCTAC
CCCCTAGTG
SMA11
ATTATTAGACAAGAAAGGGGGAAAAAAGAAGCTTTTGGACCTTAGGAATAATTTA

TGCCATGATAGCTATCGGATTATTAGGATTCGTAGTTTGAGCTCACCATATATTT
ACAGTAGATAGATGTAGATACCCGAGCTTACTTCACTTCAGCAACTATAATTATT
GCGGTTCCAACTGGGATTAAAGTCTTTAGATGACTAGCCACCTTTCATGGAACT
CAAATTTTAAATAGACCAGTAACCCTGTGAGCTTTAGGTTTTGTATTTTTATTTAC
AGTAGGAGGATTAACAGGGGTAATTTTAGCTAATTCTTCAATTGATATTATTCTTC
ACGATACTTACTATGTAGTAGCTCATTTTCACTATGTTCTTTCTATAGGAGCTGTA
TTTGCAATTATAGCCGGAATTGTCCAATGATTCCCATTATTTACCGGACTAACCT
TAAATAATTTTTTTTTAAAAACCCAATTTATTACTATATTTTTAGGTGTAAATTTAAC
ATTTTTTCCCCAACACTTTTTAGGGCTAAGAGGAATGCCTCGGCGATATTCGGAT
TATCCAGATGCTTTCACTATCTGAAATGCCATTTCATCTATTGGTTCCATTATTTC
TCTCACAAGAATTATCTTTTTTTTTATTTATCTTATGAGAAAGGCTTTCTTCCCAAC
GTAAATCTTTGAGAACTTTAAATGTAACAACTTCAATTGAATGGTTCAAGCCCTAC
CCCCTAGTG
SMA12
ATTATTAGACAAGAAAGGGGGAAAAAAGAAGCTTTTGGACCTTAGGAATAATTTA
TGCCATGATAGCTATCGGATTATTAGGATTCGTAGTTTGAGCTCACCATATATTT
ACAGTAGATAGATGTAGATACCCGAGCTTACTTCACTTCAGCAACTATAATTATT
GCGGTTCCAACTGGGATTAAAGTCTTTAGATGACTAGCCACCTTTCATGGAACT
CAAATTTTAAATAGACCAGTAACCCTGTGAGCTTTAGGTTTTGTATTTTTATTTAC
AGTAGGAGGATTAACAGGGGTAATTTTAGCTAATTCTTCAATTGATATTATTCTTC
ACGATACTTACTATGTAGTAGCTCATTTTCACTATGTTCTTTCTATAGGAGCTGTA
TTTGCAATTATAGCCGGAATTGTCCAATGATTCCCATTATTTACCGGACTAACCT
TAAATAATTTTTTTTTAAAAACCCAATTTATTACTATATTTTTAGGTGTAAATTTAAC
ATTTTTTCCCCAACACTTTTTAGGGCTAAGAGGAATGCCTCGGCGATATTCGGAT
TATCCAGATGCTTTCACTATCTGAAATGCCATTTCATCTATTGGTTCCATTATTTC
TCTCACAAGAATTATCTTTTTTTTTATTTATCTTATGAGAAAGGCTTTCTTCCCAAC
GTAAATCTTTGAGAACTTTAAATGTAACAACTTCAATTGAATGGTTCAAGCCCTAC
CCCCTAGTG
SMA13
ATTATTAGACAAGAAAGGGGGAAAAAAGAAGCTTTTGGACCTTAGGAATAATTTA
TGCCATGATAGCTATCGGATTATTAGGATTCGTAGTTTGAGCTCACCATATATTT
ACAGTAGATAGATGTAGATACCCGAGCTTACTTCACTTCAGCAACTATAATTATT
GCGGTTCCAACTGGGATTAAAGTCTTTAGATGACTAGCCACCTTTCATGGAACT
CAAATTTTAAATAGACCAGTAACCCTGTGAGCTTTAGGTTTTGTATTTTTATTTAC
AGTAGGAGGATTAACAGGGGTAATTTTAGCTAATTCTTCAATTGATATTATTCTTC
ACGATACTTACTATGTAGTAGCTCATTTTCACTATGTTCTTTCTATAGGAGCTGTA
TTTGCAATTATAGCCGGAATTGTCCAATGATTCCCATTATTTACCGGACTAACCT
TAAATAATTTTTTTTTAAAAACCCAATTTATTACTATATTTTTAGGTGTAAATTTAAC
ATTTTTTCCCCAACACTTTTTAGGGCTAAGAGGAATGCCTCGGCGATATTCGGAT
TATCCAGATGCTTTCACTATCTGAAATGCCATTTCATCTATTGGTTCCATTATTTC
TCTCACAAGAATTATCTTTTTTTTTATTTATCTTATGAGAAAGGCTTTCTTCCCAAC
GTAAATCTTTGAGAACTTTAAATGTAACAACTTCAATTGAATGGTTCAAGCCCTAC
CCCCTAGTG
SMA14
ATTATTAGACAAGAAAGGGGGAAAAAAGAAGCTTTTGGACCTTAGGAATAATTTA
TGCCATGATAGCTATCGGATTATTAGGATTCGTAGTTTGAGCTCACCATATATTT
ACAGTAGATAGATGTAGATACCCGAGCTTACTTCACTTCAGCAACTATAATTATT
GCGGTTCCAACTGGGATTAAAGTCTTTAGATGACTAGCCACCTTTCATGGAACT
CAAATTTTAAATAGACCAGTAACCCTGTGAGCTTTAGGTTTTGTATTTTTATTTAC
AGTAGGAGGATTAACAGGGGTAATTTTAGCTAATTCTTCAATTGATATTATTCTTC

ACGATACTTACTATGTAGTAGCTCATTTTCACTATGTTCCTTCTATAGGAGCTGTA
TTTGCAATTATAGCCGGAATTGTCCAATGATTCCCATTATTTACCGGACTAACCT
TAAATAATTTTTTTTTAAAAACCCAATTTATTACTATATTTTTAGGTGTAAATTTAAC
ATTTTTTCCCCAACACTTTTTAGGGCTAAGAGGAATGCCTCGGCGATATTCGGAT
TATCCAGATGCTTTCACTATCTGAAATGCCATTTCATCTATTGGTTCCATTATTTC
TCTCACAAGAATTATCTTTTTTTTATTTATCTTATGAGAAAGGCTTTCTTCCCAAC
GTAAATCTTTGAGAACTTTAAATGTAACAACTTCAATTGAATGGTTCAAGCCCTAC
CCCCTAGTG
SMA6
ATTATTAGACAAGAAAGGGGGAAAAAAGAAGCTTTTGGACCTTAGGAATAATTTA
TGCCATGATAGCTATCGGATTATTAGGATTCGTAGTTTGAGCTCACCATATATTT
ACAGTAGATAGATGTAGATACCCGAGCTTACTTCACTTCAGCAACTATAATTATT
GCGGTTCCAACTGGGATTAAAGTCTTTAGATGACTAGCCACCTTTCATGGAACT
CAAATTTTAAATAGACCAGTAACCCTGTGAGCTTTAGGTTTTGTATTTTTATTTAC
AGTAGGAGGATTAACAGGGGTAATTTTAGCTAATTCTTCAATTGATATTATTCTTC
ACGATACTTACTATGTAGTAGCTCATTTTCACTATGTTCTTTCTATAGGAGCTGTA
TTTGCAATTATAGCCGGAATTGTCCAATGATTCCCATTATTTACCGGACTAACCT
TAAATAATTTTTTTTTAAAAACCCAATTTATTACTATATTTTTAGGTGTAAATTTAAC
ATTTTTTCCCCAACACTTTTTAGGGCTAAGAGGAATGCCTCGGCGATATTCGGAT
TATCCAGATGCTTTCACTATCTGAAATGCCATTTCATCTATTGGTTCCATTATTTC
TCTCACAAGAATTATCTTTTTTTTATTTATCTTATGAGAAAGGCTTTCTTCCCAAC
GTAAATCTTTGAGAACTTTAAATGTAACAACTTCAATTGAATGGTTCAAGCCCTAC
CCCCTAGTG
SMI1
ATTATTAGACAAGAAAGGGGGAAAAAAGAAGCTTTTGGACCTTAGGAATAATTTA
TGCCATGATAGCTATCGGATTATTAGGATTCGTAGTTTGAGCTCACCATATATTT
ACAGTAGATAGATGTAGATACCCGAGCTTACTTCACTTCAGCAACTATAATTATT
GCGGTTCCAACTGGGATTAAAGTCTTTAGATGACTAGCCACCTTTCATGGAACT
CAAATTTTAAATAGACCAGTAACCCTGTGAGCTTTAGGTTTTGTATTTTTATTTAC
AGTAGGAGGATTAACAGGGGTAATTTTAGCTAATTCTTCAATTGATATTATTCTTC
ACGATACTTACTATGTAGTAGCTCATTTTCACTATGTTCTTTCTATAGGAGCTGTA
TTTGCAATTATAGCCGGAATTGTCCAATGATTCCCATTATTTACCGGACTAACCT
TAAATAATTTTTTTTTAAAAACCCAATTTATTACTATATTTTTAGGTGTAAATTTAAC
ATTTTTTCCCCAACACTTTTTAGGGCTAAGAGGAATGCCTCGGCGATATTCGGAT
TATCCAGATGCTTTCACTATCTGAAATGCCATTTCATCTATTGGTTCCATTATTTC
TCTCACAAGAATTATCTTTTTTTTATTTATCTTATGAGAAAGGCTTTCTTCCCAAC
GTAAATCTTTGAGAACTTTAAATGTAACAACTTCAATTGAATGGTTCAAGCCCTAC
CCCCTAGTG
SMI2
ATTATTAGACAAGAAAGGGGGAAAAAAGAAGCTTTTGGACCTTAGGAATAATTTA
TGCCATGATAGCTATCGGATTATTAGGATTCGTAGTTTGAGCTCACCATATATTT
ACAGTAGATAGATGTAGATACCCGAGCTTACTTCACTTCAGCAACTATAATTATT
GCGGTTCCAACTGGGATTAAAGTCTTTAGATGACTAGCCACCTTTCATGGAACT
CAAATTTTAAATAGACCAGTAACCCTGTGAGCTTTAGGTTTTGTATTTTTATTTAC
AGTAGGAGGATTAACAGGGGTAATTTTAGCTAATTCTTCAATTGATATTATTCTTC
ACGATACTTACTATGTAGTAGCTCATTTTCACTATGTTCTTTCTATAGGAGCTGTA
TTTGCAATTATAGCCGGAATTGTCCAATGATTCCCATTATTTACCGGACTAACCT
TAAATAATTTTTTTTTAAAAACCCAATTTATTACTATATTTTTAGGTGTAAATTTAAC
ATTTTTTCCCCAACACTTTTTAGGGCTAAGAGGAATGCCTCGGCGATATTCGGAT
TATCCAGATGCTTTCACTATCTGAAATGCCATTTCATCTATTGGTTCCATTATTTC

TCTCACAAGAATTATCTTTTTTTTATTTATCTTATGAGAAAGGCTTTCTTCCCAAC
GTAAATCTTTGAGAACTTTAAATGTAACAACTTCAATTGAATGGTTCAAGCCCTAC
CCCCTAGTG
sob1
ATTATTAGACAAGAAAGGGGGAAAAAAGAAGCTTTTGGACCTTAGGAATAATTTA
TGCCATGATAGCTATCGGATTATTAGGATTCGTAGTTTGAGCTCACCATATATTT
ACAGTAGATAGATGTAGATACCCGAGCTTACTTCACTTCAGCAACTATAATTATT
GCGGTTCCAACTGGGATTAAAGTCTTTAGATGACTAGCCACCTTTCATGGAACT
CAAATTTTAAATAGACCAGTAACCCTGTGAGCTTTAGGTTTTGTATTTTTATTTAC
AGTAGGAGGATTAACGGGGGTAATTTTAGCTAATTCTTCAATTGATATTATTCTTC
ACGATACTTACTATGTAGTAGCTCATTTTCACTATGTTCTTTCTATAGGAGCTGTA
TTTGCAATTATAGCCGGAATTGTCCAATGATTCCCATTATTTACCGGACTAACCT
TAAATAATTTTTTTTTAAAAACCCAATTTATTACTATATTTTTAGGTGTAAATTTAAC
ATTTTTTCCCCAACACTTTTTAGGGCTAAGAGGAATGCCTCGGCGATATTCGGAT
TATCCAGATGCTTTCACTATCTGAAATGCCATTTCATCTATTGGTTCCATTATTTC
TCTCACAAGAATTATCTTTTTTTTATTTATCTTATGAGAAAGGCTTTCTTCCCAAC
GTAAATCTTTGAGAACTTTAAATGTAACAACTTCAATTGAATGGTTCAAGCCCTAC
CCCCTAGTG
SOB10
ATTATTAGACAAGAAAGGGGGAAAAAAGAAGCTTTTGGACCTTAGGAATAATTTA
TGCCATGATAGCTATCGGATTATTAGGATTCGTAGTTTGAGCTCACCATATATTT
ACAGTAGATAGATGTAGATACCCGAGCTTACTTCACTTCAGCAACTATAATTATC
GCGGTTCCAACTGGGATTAAAGTCTTTAGGTGACTAGCCACCTTTCATGGAACT
CAAATTTTAAATAGACCAGTAACCCTGTGAGCTTTAGGTTTTGTATTTTTATTTAC
AGTAGGAGGATTAACAGGGGTAATTTTAGCTAATTCTTCAATTGATATTATTCTTC
ACGATACTTACTATGTAGTAGCTCATTTTCACTATGTTCTTTCTATAGGAGCTGTA
TTTGCAATTATAGCCGGAATTGTCCAATGATTCCCATTATTTACTGGACTAACCTT
AAATAATTTTTTTTTAAAAACCCAATTTATTACTATATTTTTAGGTGTAAATTTAACA
TTTTTTCCTCAACACTTTTTAGGGCTAAGAGGGATGCCTCGGCGATATTCGGATT
ATCCAGATGCTTTCACTATCTGAAATGCCATTTCATCTATTGGTTCCATTATTTCT
CTCACAAGAATTATCTTTTTTTTATTTATCTTATGAGAAAGGCTTTCTTCCCAACG
TAAATCTTTGAGAACTTTAAATGTAACAACTTCAATTGAATGGTTCAAGCCCTACC
CCCTAGTG
SOB12
ATTATTAGACAAGAAAGGGGGAAAAAAGAAGCTTTTGGACCTTAGGAATAATTTA
TGCCATGATAGCTATCGGATTATTAGGATTCGTAGTTTGAGCTCACCATATATTT
ACAGTAGATAGATGTAGATACCCGAGCTTACTTCACTTCAGCAACTATAATTATC
GCGGTTCCAACTGGGATTAAAGTCTTTAGGTGACTAGCCACCTTTCATGGAACT
CAAATTTTAAATAGACCAGTAACCCTGTGAGCTTTAGGTTTTGTATTTTTATTTAC
AGTAGGAGGATTAACAGGGGTAATTTTAGCTAATTCTTCAATTGATATTATTCTTC
ACGATACTTACTATGTAGTAGCTCATTTTCACTATGTTCTTTCTATAGGAGCTGTA
TTTGCAATTATAGCCGGAATTGTCCAATGATTCCCATTATTTACTGGACTAACCTT
AAATAATTTTTTTTTAAAAACCCAATTTATTACTATATTTTTAGGTGTAAATTTAACA
TTTTTTCCTCAACACTTTTTAGGGCTAAGAGGAATGCCTCGGCGATATTCGGATT
ATCCAGATGCTTTCACTATCTGAAATGCCATTTCATCTATTGGTTCCATTATTTCT
CTCACAAGAATTATCTTTTTTTTATTTATCTTATGAGAAAGGCTTTCTTCCCAACG
TAAATCTTTGAGAACTTTAAATGTAACAACTTCAATTGAATGGTTCAAGCCCTACC
CCCTAGTG
SOB1_2
ATTATTAGACAAGAAAGGGGGAAAAAAGAAGCTTTTGGACCTTAGGAATAATTTA

TGCCATGATAGCTATCGGATTATTAGGATTCGTAGTTTGAGCTCACCATATATTT
ACAGTAGATAGATGTAGATACCCGAGCTTACTTCACTTCAGCAACTATAATTATC
GCGGTTCCAACTGGGATTAAAGTCTTTAGGTGACTAGCCACCTTTCATGGAACT
CAAATTTTAAATAGACCAGTAACCCTGTGAGCTTTAGGTTTTGTATTTTTATTTAC
AGTAGGAGGATTAACAGGGGTAATTTTAGCTAATTCTTCAATTGATATTATTCTTC
ACGATACTTACTATGTAGTAGCTCATTTTCACTATGTTCTTTCTATAGGAGCTGTA
TTTGCAATTATAGCCGGAATTGTCCAATGATTCCCATTATTTACTGGACTAACCTT
AAATAATTTTTTTTTAAAAACCCAATTTATTACTATATTTTTAGGTGTAAATTTAACA
TTTTTTCCTCAACACTTTTTAGGGCTAAGAGGAATGCCTCGGCGATATTCGGATT
ATCCAGATGCTTTCACTATCTGAAATGCCATTTCATCTATTGGTTCCATTATTTCT
CTCACAAGAATTATCTTTTTTTTTATTTATCTTATGAGAAAGGCTTTCTTCCCAACG
TAAATCTTTGAGAACTTTAAATGTAACAACTTCAATTGAATGGTTCAAGCCCTACC
CCCTAGTG
SOB2
ATTATTAGACAAGAAAGGGGGAAAAAAGAAGCTTTTGGACCTTAGGAATAATTTA
TGCCATGATAGCTATCGGATTATTAGGATTCGTAGTTTGAGCTCACCATATATTT
ACAGTAGATAGATGTAGATACCCGAGCTTACTTCACTTCAGCAACTATAATTATT
GCGGTTCCAACTGGGATTAAAGTCTTTAGATGACTAGCCACCTTTCATGGAACT
CAAATTTTAAATAGACCAGTAACCCTGTGAGCTTTAGGTTTTGTATTTTTATTTAC
AGTAGGAGGATTAACGGGGGTAATTTTAGCTAATTCTTCAATTGATATTATTCTTC
ACGATACTTACTATGTAGTAGCTCATTTTCACTATGTTCTTTCTATAGGAGCTGTA
TTTGCAATTATAGCCGGAATTGTCCAATGATTCCCATTATTTACCGGACTAACCT
TAAATAATTTTTTTTTAAAAACCCAATTTATTACTATATTTTTAGGTGTAAATTTAAC
ATTTTTTCCCCAACACTTTTTAGGGCTAAGAGGAATGCCTCGGCGATATTCGGAT
TATCCAGATGCTTTCACTATCTGAAATGCCATTTCATCTATTGGTTCCATTATTTC
TCTCACAAGAATTATCTTTTTTTTTATTTATCTTATGAGAAAGGCTTTCTTCCCAAC
GTAAATCTTTGAGAACTTTAAATGTAACAACTTCAATTGAATGGTTCAAGCCCTAC
CCCCTAGTG
SOB2_1
ATTATTAGACAAGAAAGGGGGAAAAAAGAAGCTTTTGGACCTTAGGAATAATTTA
TGCCATGATAGCTATCGGATTATTAGGATTCGTAGTTTGAGCTCACCATATATTT
ACAGTAGATAGATGTAGATACCCGAGCTTACTTCACTTCAGCAACTATAATTATC
GCGGTTCCAACTGGGATTAAAGTCTTTAGGTGACTAGCCACCTTTCATGGAACT
CAAATTTTAAATAGACCAGTAACCCTGTGAGCTTTAGGTTTTGTATTTTTATTTAC
AGTAGGAGGATTAACAGGGGTAATTTTAGCTAATTCTTCAATTGATATTATTCTTC
ACGATACTTACTATGTAGTAGCTCATTTTCACTATGTTCTTTCTATAGGAGCTGTA
TTTGCAATTATAGCCGGAATTGTCCAATGATTCCCATTATTTACTGGACTAACCTT
AAATAATTTTTTTTTAAAAACCCAATTTATTACTATATTTTTAGGTGTAAATTTAACA
TTTTTTCCTCAACACTTTTTAGGGCTAAGAGGAATGCCTCGGCGATATTCGGATT
ATCCAGATGCTTTCACTATCTGAAATGCCATTTCATCTATTGGTTCCATTATTTCT
CTCACAAGAATTATCTTTTTTTTTATTTATCTTATGAGAAAGGCTTTCTTCCCAACG
TAAATCTTTGAGAACTTTAAATGTAACAACTTCAATTGAATGGTTCAAGCCCTACC
CCCTAGTG
TAP1
ATTATTAGACAAGAAAGGGGGAAAAAAGAAGCTTTTGGACCTTAGGAATAATTTA
TGCCATGATAGCTATCGGATTATTAGGATTCGTAGTTTGAGCTCACCATATATTT
ACAGTAGATAGATGTAGATACCCGAGCTTACTTCACTTCAGCAACTATAATTATT
GCGGTTCCAACTGGGATTAAAGTCTTTAGATGACTAGCCACCTTTCATGGAACT
CAAATTTTAAATAGACCAGTAACCCTGTGAGCTTTAGGTTTTGTATTTTTATTTAC
AGTAGGAGGATTAACAGGGGTAATTTTAGCTAATTCTTCAATTGATATTATTCTTC

ACGATACTTACTATGTAGTAGCTCATTTTCACTATGTTCTTTCTATAGGAGCTGTA
TTTGCAATTATAGCCGGAATTGTCCAATGATTCCCATTATTTACCGGACTAACCT
TAAATAATTTTTTTTTAAAAACCCAATTTATTACTATATTTTTAGGTGTAAATTTAAC
ATTTTTTCCCCAACACTTTTTAGGGCTAAGAGGAATGCCTCGGCGATATTCGGAT
TATCCAGATGCTTTCACTATCTGAAATGCCATTTCATCTATTGGTTCCATTATTTC
TCTCACAAGAATTATCTTTTTTTTATTTATCTTATGAGAAAGGCTTTCTTCCCAAC
GTAAATCTTTGAGAACTTTAAATGTAACAACTTCAATTGAATGGTTCAAGCCCTAC
CCCCTAGTG
TAP2
ATTATTAGACAAGAAAGGGGGAAAAAAGAAGCTTTTGGACCTTAGGAATAATTTA
TGCCATGATAGCTATCGGATTATTAGGATTCGTAGTTTGAGCTCACCATATATTT
ACAGTAGATAGATGTAGATACCCGAGCTTACTTCACTTCAGCAACTATAATTATT
GCGGTTCCAACTGGGATTAAAGTCTTTAGATGACTAGCCACCTTTCATGGAACT
CAAATTTTAAATAGACCAGTAACCCTGTGAGCTTTAGGTTTTGTATTTTTATTTAC
AGTAGGAGGATTAACAGGGGTAATTTTAGCTAATTCTTCAATTGATATTATTCTTC
ACGATACTTACTATGTAGTAGCTCATTTTCACTATGTTCTTTCTATAGGAGCTGTA
TTTGCAATTATAGCCGGAATTGTCCAATGATTCCCATTATTTACCGGACTAACCT
TAAATAATTTTTTTTTAAAAACCCAATTTATTACTATATTTTTAGGTGTAAATTTAAC
ATTTTTTCCCCAACACTTTTTAGGGCTAAGAGGAATGCCTCGGCGATATTCGGAT
TATCCAGATGCTTTCACTATCTGAAATGCCATTTCATCTATTGGTTCCATTATTTC
TCTCACAAGAATTATCTTTTTTTTATTTATCTTATGAGAAAGGCTTTCTTCCCAAC
GTAAATCTTTGAGAACTTTAAATGTAACAACTTCAATTGAATGGTTCAAGCCCTAC
CCCCTAGTG
tol1
ATTATTAGACAAGAAAGGGGGAAAAAAGAAGCTTTTGGACCTTAGGAATAATTTA
TGCCATGATAGCTATCGGATTATTAGGATTCGTAGTTTGAGCTCACCATATATTT
ACAGTAGATAGATGTAGATACCCGAGCTTACTTCACTTCAGCAACTATAATTATT
GCGGTTCCAACTGGGATTAAAGTCTTTAGATGACTAGCCACCTTTCATGGAACT
CAAATTTTAAATAGACCAGTAACCCTGTGAGCTTTAGGTTTTGTATTTTTATTTAC
AGTAGGAGGATTAACAGGGGTAATTTTAGCTAATTCTTCAATTGATATTATTCTTC
ACGATACTTACTATGTAGTAGCTCATTTTCACTATGTTCTTTCTATAGGAGCTGTA
TTTGCAATTATAGCCGGAATTGTCCAATGATTCCCATTATTTACCGGACTAACCT
TAAATAATTTTTTTTTAAAAACCCAATTTATTACTATATTTTTAGGTGTAAATTTAAC
ATTTTTTCCCCAACACTTTTTAGGGCTAAGAGGAATGCCTCGGCGATATTCGGAT
TATCCAGATGCTTTCACTATCTGAAATGCCATTTCATCTATTGGTTCCATTATTTC
TCTCACAAGAATTATCTTTTTTTTATTTATCTTATGAGAAAGGCTTTCTTCCCAAC
GTAAATCTTTGAGAACTTTAAATGTAACAACTTCAATTGAATGGTTCAAGCCCTAC
CCCCTAGTG
TOL1_1
ATTATTAGACAAGAAAGGGGGAAAAAAGAAGCTTTTGGACCTTAGGAATAATTTA
TGCCATGATAGCTATCGGATTATTAGGATTCGTAGTTTGAGCTCACCATATATTT
ACAGTAGATAGATGTAGATACCCGAGCTTACTTCACTTCAGCAACTATAATTATT
GCGGTTCCAACTGGGATTAAAGTCTTTAGATGACTAGCCACCTTTCATGGAACT
CAAATTTTAAATAGACCAGTAACCCTGTGAGCTTTAGGTTTTGTATTTTTATTTAC
AGTAGGAGGATTAACAGGGGTAATTTTAGCTAATTCTTCAATTGATATTATTCTTC
ACGATACTTACTATGTAGTAGCTCATTTTCACTATGTTCTTTCTATAGGAGCTGTA
TTTGCAATTATAGCCGGAATTGTCCAATGATTCCCATTATTTACCGGACTAACCT
TAAATAATTTTTTTTTAAAAACCCAATTTATTACTATATTTTTAGGTGTAAATTTAAC
ATTTTTTCCCCAACACTTTTTAGGGCTAAGAGGAATGCCTCGGCGATATTCGGAT
TATCCAGATGCTTTCACTATCTGAAATGCCATTTCATCTATTGGTTCCATTATTTC

TCTCACAAGAATTATCTTTTTTTTATTTATCTTATGAGAAAGGCTTTCTTCCCAAC
GTAAATCTTTGAGAACTTTAAATGTAACAACTTCAATTGAATGGTTCAAGCCCTAC
CCCCTAGTG
TOL5
ATTATTAGACAAGAAAGGGGGAAAAAAGAAGCTTTTGGACCTTAGGAATAATTTA
TGCCATGATAGCTATCGGATTATTAGGATTCGTAGTTTGAGCTCACCATATATTT
ACAGTAGATAGATGTAGATACCCGAGCTTACTTCACTTCAGCAACTATAATTATT
GCGGTTCCAACTGGGATTAAAGTCTTTAGATGACTAGCCACCTTTCATGGAACT
CAAATTTTAAATAGACCAGTAACCCTGTGAGCTTTAGGTTTTGTATTTTTATTTAC
AGTAGGAGGATTAACAGGGGTAATTTTAGCTAATTCTTCAATTGATATTATTCTTC
ACGATACTTACTATGTAGTAGCTCATTTTCACTATGTTCTTTCTATAGGAGCTGTA
TTTGCAATTATAGCCGGAATTGTCCAATGATTCCCATTATTTACCGGACTAACCT
TAAATAATTTTTTTTTAAAAACCCAATTTATTACTATATTTTTAGGTGTAAATTTAAC
ATTTTTTCCCCAACACTTTTTAGGGCTAAGAGGAATGCCTCGGCGATATTCGGAT
TATCCAGATGCTTTCACTATCTGAAATGCCATTTCATCTATTGGTTCCATTATTTC
TCTCACAAGAATTATCTTTTTTTTATTTATCTTATGAGAAAGGCTTTCTTCCCAAC
GTAAATCTTTGAGAACTTTAAATGTAACAACTTCAATTGAATGGTTCAAGCCCTAC
CCCCTAGTG
VIC10
ATTATTAGACAAGAAAGGGGGAAAAAAGAAGCTTTTGGACCTTAGGAATAATTTA
TGCCATGATAGCTATCGGATTATTAGGATTCGTAGTTTGAGCTCACCATATATTT
ACAGTAGATAGATGTAGATACCCGAGCTTACTTCACTTCAGCAACTATAATTATT
GCGGTTCCAACTGGGATTAAAGTCTTTAGATGACTAGCCACCTTTCATGGAACT
CAAATTTTAAATAGACCAGTAACCCTGTGAGCTTTAGGTTTTGTATTTTTATTTAC
AGTAGGAGGATTAACAGGGGTAATTTTAGCTAATTCTTCAATTGATATTATTCTTC
ACGATACTTACTATGTAGTAGCTCATTTTCACTATGTTCTTTCTATAGGAGCTGTA
TTTGCAATTATAGCCGGAATTGTCCAATGATTCCCATTATTTACCGGACTAACCT
TAAATAATTTTTTTTTAAAAACCCAATTTATTACTATATTTTTAGGTGTAAATTTAAC
ATTTTTTCCCCAACACTTTTTAGGGCTAAGAGGAATGCCTCGGCGATATTCGGAT
TATCCAGATGCTTTCACTATCTGAAATGCCATTTCATCTATTGGTTCCATTATTTC
TCTCACAAGAATTATCTTTTTTTTATTTATCTTATGAGAAAGGCTTTCTTCCCAAC
GTAAATCTTTGAGAACTTTAAATGTAACAACTTCAATTGAATGGTTCAAGCCCTAC
CCCCTAGTG
XAN4
ATTATTAGACAAGAAAGGGGGAAAAAAGAAGCTTTTGGACCTTAGGAATAATTTA
TGCCATGATAGCTATCGGATTATTAGGATTCGTAGTTTGAGCTCACCATATATTT
ACAGTAGATAGATGTAGATACCCGAGCTTACTTCACTTCAGCAACTATAATTATC
GCGGTTCCAACTGGGATTAAAGTCTTTAGGTGACTAGCCACCTTTCATGGAACT
CAAATTTTAAATAGACCAGTAACCCTGTGAGCTTTAGGTTTTGTATTTTTATTTAC
AGTAGGAGGATTAACAGGGGTAATTTTAGCTAATTCTTCAATTGATATTATTCTTC
ACGATACTTACTATGTAGTAGCTCATTTTCACTATGTTCTTTCTATAGGAGCTGTA
TTTGCAATTATAGCCGGAATTGTCCAATGATTCCCATTATTTACTGGACTAACCTT
AAATAATTTTTTTTTAAAAACCCAATTTATTACTATATTTTTAGGTGTAAATTTAACA
TTTTTTCCTCAACACTTTTTAGGGCTAAGAGGAATGCCTCGGCGATATTCGGATT
ATCCAGATGCTTTCGCTATCTGAAATGCCATTTCATCTATTGGTTCCATTATTTCT
CTCACAAGAATTATCTTTTTTTTATTTATCTTATGAGAAAGGCTTTCTTCCCAACG
TAAATCTTTGAGAACTTTAAATGTAACAACTTCAATTGAATGGTTCAAGCCCTACC
CCCTAGTG
XAX2
ATTATTAGACAAGAAAGGGGGAAAAAAGAAGCTTTTGGACCTTAGGAATAATTTA

TGCCATGATAGCTATCGGATTATTAGGATTCGTAGTTTGAGCTCACCATATATTT
ACAGTAGATAGATGTAGATACCCGAGCTTACTTCACTTCAGCAACTATAATTATT
GCGGTTCCAACTGGGATTAAAGTCTTTAGATGACTAGCCACCTTTCATGGAACT
CAAATTTTAAATAGACCAGTAACCCTGTGAGCTTTAGGTTTTGTATTTTTATTTAC
AGTAGGAGGATTAACAGGGGTAATTTTAGCTAATTCTTCAATTGATATTATTCTTC
ACGATACTTACTATGTAGTAGCTCATTTTCACTATGTTCTTTCTATAGGAGCTGTA
TTTGCAATTATAGCCGGAATTGTCCAATGATTCCCATTATTTACCGGACTAACCT
TAAATAATTTTTTTTTAAAAACCCAATTTATTACTATATTTTTAGGTGTAAATTTAAC
ATTTTTTCCCCAACACTTTTTAGGGCTAAGAGGAATGCCTCGGCGATATTCGGAT
TATCCAGATGCTTTCACTATCTGAAATGCCATTTCATCTATTGGTTCCATTATTTC
TCTCACAAGAATTATCTTTTTTTTATTTATCTTATGAGAAAGGCTTTCTTCCCAAC
GTAAATCTTTGAGAACTTTAAATGTAACAACTTCAATTGAATGGTTCAAGCCCTAC
CCCCTAGTG
MEX_SFP
ATTATTAGACAAGAAAGGGGGAAAAAAGAAGCTTTTGGACCTTAGGAATAATTTA
TGCCATAATAGCTATCGGATTATTAGGATTCGTAGTTTGAGCTCACCATATATTTA
CAGTAGATAGATGTAGATACCCGAGCTTACTTCACTTCAGCAACTATAATTATTG
CGGTTCCAACTGGGATTAAAGTCTTTAGATGACTAGCCACCTTTCATGGAACTCA
AATTTTAAATAGACCAGTAACCCTGTGAGCTTTAGGTTTTGTATTTTTATTTACAG
TAGGAGGATTAACAGGGGTAATTTTAGCTAATTCTTCAATTGATATTATTCTTCAC
GATACTTACTATGTAGTAGCTCATTTTCACTATGTTCTTTCTATAGGAGCTGTATT
TGCAATTATAGCCGGAATTGTCCAATGATTCCCATTATTTACTGGACTAACCTTA
AATAATTTTTTTTTAAAAACCCAATTTATTACTATATTTTTAGGTGTAAATTTAACAT
TTTTTCCTCAACACTTTTTAGGGCTAAGAGGAATGCCTCGGCGATATTCGGATTA
TCCAGATGCTTTCACTATCTGAAATGCCATTTCATCTATTGGTTCCATTATTTCTC
TCACAAGAATTATCTTTTTTTTATTTATCTTATGAGAAAGGCTTTCTTCCCAACGT
AAATCTTTGAGAACTTTAAATGTAACAACTTCAATTGAATGGTTCAAGCCCTACC
CCCTAGTG
MEX_COE_2
ATTATTAGACAAGAAAGGGGGAAAAAAGAACCTTTTGGACCTTAGGAATAATTTA
TGCCATAATAGCTATCGGATTATTAGGATTCGTAGTTTGAGCTCACCATATATTTA
CAGTAGATAGATGTAGATACCCGAGCTTACTTCACTTCAGCAACTATAATTATTG
CGGTTCCAACTGGGATTAAAGTCTTTAGATGACTAGCCACCTTTCATGGAACTCA
AATTTTAAATAGACCAGTAACCCTGTGAGCTTTAGGTTTTGTATTTTTATTTACAG
TAGGAGGATTAACAGGGGTAATTTTAGCTAATTCTTCAATTGATATTATTCTTCAC
GATACTTACTATGTAGTAGCTCATTTTCACTATGTTCTTTCTATAGGAGCTGTATT
TGCAATTATAGCCGGAATTGTCCAATGATTCCCATTATTTACTGGACTAACCTTA
AATAATTTTTTTTTAAAAACCCAATTTATTACTATATTTTTAGGTGTAAATTTAACAT
TTTTTCCTCAACACTTTTTAGGGCTAAGAGGAATGCCTCGGCGATATTCGGATTA
TCCAGATGCTTTCACTATCTGAAATGCCATTTCATCTATTGGTTCCATTATTTCTC
TCACAAGAATTATCTTTTTTTTATTTATCTTATGAGAAAGGCTTTCTTCCCAACGT
AAATCTTTGAGAACTTTAAATGTAACAACTTCAATTGAATGGTTCAAGCCCTACC
CCCTAGTG
MEX_SAG_5
ATTATTAGACAAGAAAGGGGGAAAAAAGAAGCTTTTGGACCTTAGGAATAATTTA
TGCCATAATAGCTATCGGATTATTAGGATTCGTAGTTTGAGCTCACCATATATTTA
CAGTAGATAGATGTAGATACCCGAGCTTACTTCACTTCAGCAACTATAATTATTG
CGGTTCCAACTGGGATTAAAGTCTTTAGATGACTAGCCACCTTTCATGGAACTCA
AATTTTAAATAGACCAGTAACCCTGTGAGCTTTAGGTTTTGTATTTTTATTTACAG
TAGGAGGATTAACAGGGGTAATTTTAGCTAATTCTTCAATTGATATTATTCTTCAC

GATACTTACTATGTAGTAGCTCATTTTCACTATGTTCTTTCTATAGGAGCTGTATT
TGCAATTATAGCCGGAATTGTCCAATGATTCCCATTATTTACTGGGCTAACCTTA
AATAATTTTTTTTTAAAAACCCAATTTATTACTATATTTTTAGGTGTAAATTTAACAT
TTTTTCCTCAACACTTTTTAGGGCTAAGAGGAATGCCTCGGCGATATTCGGATTA
TCCAGATGCTTTCACTATCTGAAATGCCATTTCATCTATTGGTTCCATTATTTCTC
TCACAAGAATTATCTTTTTTTTTATTTATCTTATGAGAAAGGCTTTCTTCCCAACGT
AAATCTTTGAGAACTTTAAATGTAACAACTTCAATTGAATGGTTCAAGCCCTACC
CCCTAGTG
MEX_SCA
ATTATTAGACAAGAAAGGGGGAAAAAAGAAGCTTTTGGACCTTAGGAATAATTTA
TGCCATAATAGCTATCGGATTATTAGGATTCGTAGTTTGAGCTCACCATATATTTA
CAGTAGATAGATGTAGATACCCGAGCTTACTTCACTTCAGCACCTATGATTATTG
CGGTTCCAACTGGGATTAAAGTCTTTAGATGACTAGCCACCTTTCATGGAACTCA
AATTTTAAATAGACCAGTAACCCTGTGAGCTTTAGGTTTTGTATTTTTATTTACAG
TAGGAGGATTAACAGGGGTAATTTTAGCTAATTCTTCAATTGATATTATTCTTCAC
GATACTTACTATGTAGTAGCTCATTTTCACTATGTTCTTTCTATAGGAGCTGTATT
TGCAATTATAGCCGGAATTGTCCAATGATTCCCATTATTTACTGGACTAACCTTA
AATAATTTTTTTTTAAAAACCCAATTTATTACTATATTTTTAGGTGTAAATTTAACAT
TTTTTCCTCAACACTTTTTAGGGCTAAGAGGAATGCCTCGGCGATATTCGGATTA
TCCAGATGCTTTCACTATCTGAAATGCCATTTCATCTATTGGTTCCATTATTTCTC
TCACAAGAATTATCTTTTTTTTTATTTATCTTATGAGAAAGGCTTTCTTCCCAACGT
AAATCTTTGAGAACTTTAAATGTAACAACTTCAATTGAATGGTTCAAGCCCTACC
CCCTAGTG
MEX_SJS_2
ATTATTAGACAAGAAAGGGGGAAAAAAGAACCTTTTGGACCTTAGGAATAATTTA
TGCCATAATAGCTATCGGATTATTAGGATTCGTAGTTTGAGCTCACCATATATTTA
CAGTAGATAGATGTAGATACCCGAGCTTACTTCACTTCAGCAACTATAATTATTG
CGGTTCCAACTGGAATTAAAGTCTTTAGATGACTAGCCACCTTTCATGGAACTCA
AATTTTAAATAGACCAGTAACCCTGTGAGCTTTAGGTTTTGTATTTTTATTTACAG
TAGGAGGATTAACAGGGGTAATTTTAGCTAATTCTTCAATTGATATTATTCTTCAC
GATACTTACTATGTAGTAGCTCATTTTCACTATGTTCTTTCTATAGGAGCTGTATT
TGCAATTATAGCCGGAATTGTCCAATGATTCCCATTATTTACTGGACTAACCTTA
AATAATTTTTTTTTAAAAACCCAATTTATTACTATATTTTTAGGTGTAAATTTAACAT
TTTTTCCTCAACACTTTTTAGGGCTAAGAGGAATGCCTCGGCGATATTCGGATTA
TCCAGATGCTTTCACTATCTGAAATGCCATTTCATCTATTGGTTCCATTATTTCTC
TCACAAGAATTATCTTTTTTTTTATTTATCTTATGAGAAAGGCTTTCTTCCCAACGT
AAATCTTTGAGAACTTTAAATGTAACAACTTCAATTGAATGGTTCAAGCCCTACC
CCCTAGTG
MEX_STO
ATTATTAGACAAGAAAGGGGGAAAAAAGAACCTTTTGGACCTTAGGAATAATTTA
TGCCATAATAGCTATCGGATTATTAGGATTCGTAGTTTGAGCTCACCATATATTTA
CAGTAGATAGATGTAGATACCCGAGCTTACTTCACTTCAGCAACTATAATTATTG
CGGTTCCAACTGGGATTAAAGTCTTTAGATGACTAGCCACCTTTCATGGAACTCA
AATTTTAAATAGACCAGTAACCCTGTGAGCTTTAGGTTTTGTATTTTTATTTACAG
TAGGAGGATTAACAGGGGTAATTTTAGCTAATTCTTCAATTGATATTATTCTTCAC
GATACTTACTATGTAGTAGCTCATTTTCACTATGTTCTTTCTATAGGAGCTGTATT
TGCAATTATAGCCGGAATTGTCCAATGATTCCCATTATTTACTGGACTAACCTTA
AATAATTTTTTTTTAAAAACCCAATTTATTACTATATTTTTAGGTGTAAATTTAACAT
TTTTTCCTCAACACTTTTTAGGGCTAAGAGGAATGCCTCGGCGATATTCGGATTA
TCCAGATGCTTTCACTATCTGAAATGCCATTTCATCTATTGGTTCCATTATTTCTC

TCACAAGAATTATCTTTTTTTTATTTATCTTATGAGAAAGGCTTTCTTCCCAACGT
AAATCTTTGAGAACTTTAAATGTAACAACTTCAATTGAATGGTTCAAGCCCTACC
CCCTAGTG
MEX_TLA_1
ATTATTAGACAAGAAAGGGGGAAAAAAGAAGCTTTTGGACCTTAGGAATAATTTA
TGCCATAATAGCTATCGGATTATTAGGATTCGTAGTTTGAGCTCACCATATATTTA
CAGTAGATAGATGTAGATACCCGAGCTTACTTCACTTCAGCACCTATGATTATTG
CGGTTCCAACTGGGATTAAAGTCTTTAGATGACTAGCCACCTTTCATGGAACTCA
AATTTTAAATAGACCAGTAACCCTGTGAGCTTTAGGTTTTGTATTTTTATTTACAG
TAGGAGGATTAACAGGGGTAATTTTAGCTAATTCTTCAATTGATATTATTCTTCAC
GATACTTACTATGTAGTAGCTCATTTTCACTATGTTCTTTCTATAGGAGCTGTATT
TGCAATTATAGCCGGAATTGTCCAATGATTCCCATTATTTACTGGACTAACCTTA
AATAATTTTTTTTTAAAAACCCAATTTATTACTATATTTTTAGGTGTAAATTTAACAT
TTTTTCCTCAACACTTTTTAGGGCTAAGAGGAATGCCTCGGCGATATTCGGATTA
TCCAGATGCTTTCACTATCTGAAATGCCATTTCATCTATTGGTTCCATTATTTCTC
TCACAAGAATTATCTTTTTTTTATTTATCTTATGAGAAAGGCTTTCTTCCCAACGT
AAATCTTTGAGAACTTTAAATGTAACAACTTCAATTGAATGGTTCAAGCCCTACC
CCCTAGTG
MEX_XOT_3
ATTATTAGACAAGAAAGGGGGAAAAAAGAAGCTTTTGGACCTTAGGAATAATTTA
TGCCATGATAGCTATCGGATTATTAGGATTCGTAGTTTGAGCTCACCATATATTT
ACAGTAGATAGATGTAGATACCCGAGCTTACTTCACTTCAGCAACTATAATTATT
GCGGTTCCAACTGGGATTAAAGTCTTTAGATGACTAGCCACCTTTCATGGAACT
CAAATTTTAAATAGACCAGTAACCCTGTGAGCTTTAGGCTTTGTATTTTTATTTAC
AGTAGGAGGATTAACAGGGGTAATTTTAGCTAATTCTTCAATTGATATTATTCTTC
ACGATACTTACTATGTAGTAGCTCATTTTCACTATGTTCTTTCTATAGGAGCTGTA
TTTGCAATTATAGCCGGAATTGTCCAATGATTTCCATTATTTACTGGACTAACCTT
AAATAATTTTTTTTTAAAAACCCAATTTATTACTATATTTTTAGGTGTAAATTTAACA
TTTTTTCCTCAACACTTTTTAGGGCTAAGAGGAATGCCTCGGCGATATTCGGATT
ATCCAGATGCTTTCACTATCTGAAATGCCATTTCATCTATTGGTTCCATTATTTCT
CTCACAAGAATTATCTTTTTTTTATTTATCTTATGAGAAAGGCTTTCTTCCCAACG
TAAATCTTTGAGAACTTTAAATGTAACAACTTCAATTGAATGGTTCAAGCCCTACC
CCCTAGTG
MEX_MAL_1
ATTATTAGACAAGAAAGGGGGAAAAAAGAAGCTTTTGGACCTTAGGAATAATTTA
TGCCATAATAGCTATCGGATTATTAGGATTCGTAGTTTGAGCTCACCATATATTTA
CAGTAGATAGATGTAGATACCCGAGCTTACTTCACTTCAGCAACTATAATTATTG
CGGTTCCAACTGGGATTAAAGTCTTTAGATGACTAGCCACCTTTCATGGAACTCA
AATTTTAAATAGACCAGTAACCCTGTGAGCTTTAGGTTTTGTATTTTTATTTACAG
TAGGAGGATTAACAGGGGTAATTTTAGCTAATTCTTCAATTGATATTATTCTTCAC
GATACTTACTATGTAGTAGCTCATTTTCACTATGTTCTTTCTATAGGAGCTGTATT
TGCAATTATAGCCGGAATTGTCCAATGATTCCCATTATTTACTGGACTAACCTTA
AATAATTTTTTTTTAAAAACCCAATTTATTACTATATTTTTAGGTGTAAATTTAACAT
TTTTTCCTCAACACTTTTTAGGGCTAAGAGGAATGCCTCGGCGATATTCGGATTA
TCCAGATGCTTTCACTATCTGAAATGCCATTTCATCTATTGGTTCCATTATTTCTC
TCACAAGAATTATCTTTTTTTTATTTATCTTATGAGAAAGGCTTTCTTCCCAACGT
AAATCTTTGAGAACTTTAAATGTAACAACTTCAATTGAATGGTTCAAGCCCTACC
CCCTAGTG
MEX_MAL_2
ATTATTAGACAAGAAAGGGGGAAAAAAGAAGCTTTTGGACCTTAGGAATAATTTA

TGCCATAATAGCTATGGGATTATTAGGATTCGTCGTTTGAGCTCACCATATATTT
ACAGTAGATAGATGTAGATACCCGAGCTTACTTCACTTCAGCAACTATAATTATT
GCGGTTCCAACTGGGATTAAAGTCTTTAGATGACTAGCCACCTTTCATGGAACT
CAAATTTTAAATAGACCAGTAACCCTGTGAGCTTTAGGTTTTGTATTTTTATTTAC
AGTAGGAGGATTAACAGGGGTAATTTTAGCTAATTCTTCAATTGATATTATTCTTC
ACGATACTTACTATGTAGTAGCTCATTTTCACTATGTTCTTTCTATAGGAGCTGTA
TTTGCAATTATAGCCGGAATTGTCCAATGATTCCCATTATTTACTGGACTAACCTT
AAATAATTTTTTTTTAAAAACCCAATTTATTACTATATTTTTAGGTGTAAATTTAACA
TTTTTTCCTCAACACTTTTTAGGGCTAAGAGGAATGCCTCGGCGATATTCGGATT
ATCCAGATGCTTTCACTATCTGAAATGCCATTTCATCCATTGGTTCCATTATTTCT
CTCACAAGAATTATCTTTTTTTTTATTTATCCTATGAGAAAGGCTTTCTTCCCAACG
TAAATCTTTGAGAACTTTAAATGTAACAACTTCAATTGAATGGTTCAAGCCCTACC
CCCTAGTG
MEX_OCU_1
ATTATTAGACAAGAAAGGGGGAAAAAAGAAGCTTTTGGACCTTAGGAATAATTTA
TGCCATAATAGCTATCGGATTATTAGGATTCGTAGTTTGAGCTCACCATATATTTA
CAGTAGATAGATGTAGATACCCGAGCTTACTTCACTTCAGCAACTATAATTATTG
CGGTTCCAACTGGGATTAAAGTCTTTAGATGACTAGCCACCTTTCATGGAACTCA
AATTTTAAATAGACCAGTAACCCTGTGAGCTTTAGGTTTTGTATTTTTATTTACAG
TAGGAGGATTAACAGGGGTAATTTTAGCTAATTCTTCAATTGATATTATTCTTCAC
GATACTTACTATGTAGTAGCTCATTTTCACTATGTTCTTTCTATAGGAGCTGTATT
TGCAATTATAGCCGGAATTGTCCAATGATTCCCATTATTTACTGGACTAACCTTA
AATAATTTTTTTTTAAAAACCCAATTTATTACTATATTTTTAGGTGTAAATTTAACAT
TTTTTCCTCAACACTTTTTAGGGCTAAGAGGAATGCCTCGGCGATATTCGGATTA
TCCAGATGCTTTCACTATCTGAAATGCCATTTCATCTATTGGTTCCATTATTTCTC
TCACAAGAATTATCTTTTTTTTTATTTATCTTATGAGAAAGGCTTTCTTCCCAACGT
AAATCTTTGAGAACTTTAAATGTAACAACTTCAATTGAATGGTTCAAGCCCTACC
CCCTAGTG
MEX_OCU_2
ATTATTAGACAAGAAAGGGGGAAAAAAGAAGCTTTTGGACCTTAGGAATAATTTA
TGCCATAATAGCTATCGGATTATTAGGATTCGTAGTTTGAGCTCACCATATATTTA
CAGTAGATAGATGTAGATACCCGAGCTTACTTCACTTCAGCAACTATAATTATTG
CGGTTCCAACTGGGATTAAAGTCTTTAGATGACTAGCCACCTTTCATGGAACTCA
AATTTTAAATAGACCAGTAACCCTGTGAGCTTTAGGTTTTGTATTTTTATTTACAG
TAGGAGGATTAACAGGGGTAATTTTAGCTAATTCTTCAATTGATATTATTCTTCAC
GATACTTACTATGTAGTAGCTCATTTTCACTATGTTCTTTCTATAGGAGCTGTATT
TGCAATTATAGCCGGAATTGTCCAATGATTCCCATTATTTACTGGACTAACCTTA
AATAATTTTTTTTTAAAAACCCAATTTATTACTATATTTTTAGGTGTAAATTTAACAT
TTTTTCCTCAACACTTTTTAGGGCTAAGAGGAATGCCTCGGCGATATTCGGATTA
TCCAGATGCTTTCACTATCTGAAATGCCATTTCATCTATTGGTTCCATTATTTCTC
TCACAAGAATTATCTTTTTTTTTATTTATCTTATGAGAAAGGCTTTCTTCCCAACGT
AAATCTTTGAGAACTTTAAATGTAACAACTTCAATTGAATGGTTCAAGCCCTACC
CCCTAGTG
MEX_SAG_1
ATTATTAGACAAGAAAGGGGGAAAAAAGAAGCTTTTGGACCTTAGGAATAATTTA
TGCCATAATAGCTATCGGATTATTAGGATTCGTAGTTTGAGCTCACCATATATTTA
CAGTAGATAGATGTAGATACCCGAGCTTACTTCACTTCAGCAACTATAATTATTG
CGGTTCCAACTGGGATTAAAGTCTTTAGATGACTAGCCACCTTTCATGGAACTCA
AATTTTAAATAGACCAGTAACCCTGTGAGCTTTAGGTTTTGTATTTTTATTTACAG
TAGGAGGATTAACAGGGGTAATTTTAGCTAATTCTTCAATTGATATTATTCTTCAC

GATACTTACTATGTAGTAGCTCATTTTCACTATGTTCTTTCTATAGGAGCTGTATT
TGCAATTATAGCCGGAATTGTCCAATGATTCCCATTATTTACTGGGCTAACCTTA
AATAATTTTTTTTTAAAAACCCAATTTATTACTATATTTTTAGGTGTAAATTTAACAT
TTTTTCCTCAACACTTTTTAGGGCTAAGAGGAATGCCTCGGCGATATTCGGATTA
TCCAGATGCTTTCACTATCTGAAATGCCATTTCATCTATTGGTTCCATTATTTCTC
TCACAAGAATTATCTTTTTTTTATTTATCTTATGAGAAAGGCTTTCTTCCCAACGT
AAATCTTTGAGAACTTTAAATGTAACAACTTCAATTGAATGGTTCAAGCCCTACC
CCCTAGTG
MEX_SAG_2
ATTATTAGACAAGAAAGGGGGAAAAAAGAAGCTTTTGGACCTTAGGAATAATTTA
TGCCATAATAGCTATCGGATTATTAGGATTCGTAGTTTGAGCTCACCATATATTTA
CAGTAGATAGATGTAGATACCCGAGCTTACTTCACTTCAGCAACTATAATTATTG
CGGTTCCAACTGGGATTAAAGTCTTTAGATGACTAGCCACCTTTCATGGAACTCA
AATTTTAAATAGACCAGTAACCCTGTGAGCTTTAGGTTTTGTATTTTTATTTACAG
TAGGAGGATTAACAGGGGTAATTTTAGCTAATTCTTCAATTGATATTATTCTTCAC
GATACTTACTATGTAGTAGCTCATTTTCACTATGTTCTTTCTATAGGAGCTGTATT
TGCAATTATAGCCGGAATTGTCCAATGATTCCCATTATTTACTGGGCTAACCTTA
AATAATTTTTTTTTAAAAACCCAATTTATTACTATATTTTTAGGTGTAAATTTAACAT
TTTTTCCTCAACACTTTTTAGGGCTAAGAGGAATGCCTCGGCGATATTCGGATTA
TCCAGATGCTTTCACTATCTGAAATGCCATTTCATCTATTGGTTCCATTATTTCTC
TCACAAGAATTATCTTTTTTTTATTTATCTTATGAGAAAGGCTTTCTTCCCAACGT
AAATCTTTGAGAACTTTAAATGTAACAACTTCAATTGAATGGTTCAAGCCCTACC
CCCTAGTG
MEX_SJC_1
ATTATTAGACAAGAAAGGGGGAAAAAAGAAGCTTTTGGACCTTAGGAATAATTTA
TGCCATAATAGCTATCGGATTATTAGGATTCGTAGTTTGAGCTCACCATATATTTA
CAGTAGATAGATGTAGATACCCGAGCTTACTTCACTTCAGCAACTATAATTATTG
CGGTTCCAACTGGGATTAAAGTCTTTAGATGACTAGCCACCTTTCATGGAACTCA
AATTTTAAATAGACCAGTAACCCTGTGAGCTTTAGGTTTTGTATTTTTATTTACAG
TAGGAGGATTAACAGGGGTAATTTTAGCTAATTCTTCAATTGATATTATTCTTCAC
GATACTTACTATGTAGTAGCTCATTTTCACTATGTTCTTTCTATAGGAGCTGTATT
TGCAATTATAGCCGGAATTGTCCAATGATTCCCATTATTTACTGGACTAACCTTA
AATAATTTTTTTTTAAAAACCCAATTTATTACTATATTTTTAGGTGTAAATTTAACAT
TTTTTCCTCAACACTTTTTAGGGCTAAGAGGAATGCCTCGGCGATATTCGGATTA
TCCAGATGCTTTCACTATCTGAAATGCCATTTCATCTATTGGTTCCATTATTTCTC
TCACAAGAATTATCTTTTTTTTATTTATCTTATGAGAAAGGCTTTCTTCCCAACGT
AAATCTTTGAGAACTTTAAATGTAACAACTTCAATTGAATGATTCAAGCCCTACCC
CCTAGTG
MEX_SJC_2
ATTATTAGACAAGAAAGGGGGAAAAAAGAAGCTTTTGGACCTTAGGAATAATTTA
TGCCATAATAGCTATCGGATTATTAGGATTCGTAGTTTGAGCTCACCATATATTTA
CAGTAGATAGATGTAGATACCCGAGCTTACTTCACTTCAGCAACTATAATTATTG
CGGTTCCAACTGGGATTAAAGTCTTTAGATGACTAGCCACCTTTCATGGAACTCA
AATTTTAAATAGACCAGTAACCCTGTGAGCTTTAGGTTTTGTATTTTTATTTACAG
TAGGAGGATTAACAGGGGTAATTTTAGCTAATTCTTCAATTGATATTATTCTTCAC
GATACTTACTATGTAGTAGCTCATTTTCACTATGTTCTTTCTATAGGAGCTGTATT
TGCAATTATAGCCGGAATTGTCCAATGATTCCCATTATTTACTGGACTAACCTTA
AATAATTTTTTTTTAAAAACCCAATTTATTACTATATTTTTAGGTGTAAATTTAACAT
TTTTTCCTCAACACTTTTTAGGGCTAAGAGGAATGCCTCGGCGATATTCGGATTA
TCCAGATGCTTTCACTATCTGAAATGCCATTTCATCTATTGGTTCCATTATTTCTC

TCACAAGAATTATCTTTTTTTTATTTATCTTATGAGAAAGGCTTTCTTCCCAACGT
AAATCTTTGAGAACTTTAAATGTAACAACTTCAATTGAATGGTTCAAGCCCTACC
CCCTAGTG
MEX_SJC_3
ATTATTAGACAAGAAAGGGGGAAAAAAGAAGCTTTTGGACCTTAGGAATAATTTA
TGCCATAATAGCTATCGGATTATTAGGATTCGTAGTTTGAGCTCACCATATATTTA
CAGTAGATAGATGTAGATACCCGAGCTTACTTCACTTCAGCAACTATAATTATTG
CGGTTCCAACTGGGATTAAAGTCTTTAGATGACTAGCCACCTTTCATGGAACTCA
AATTTTAAATAGACCAGTAACCCTGTGAGCTTTAGGTTTTGTATTTTTATTTACAG
TAGGAGGATTAACAGGGGTAATTTTAGCTAATTCTTCAATTGATATTATTCTTCAC
GATACTTACTATGTAGTAGCTCATTTTCACTATGTTCTTTCTATAGGAGCTGTATT
TGCAATTATAGCCGGAATTGTCCAATGATTCCCATTATTTACTGGACTAACCTTA
AATAATTTTTTTTTAAAAACCCAATTTATTACTATATTTTTAGGTGTAAATTTAACAT
TTTTTCCTCAACACTTTTTAGGGCTAAGAGGAATGCCTCGGCGATATTCGGATTA
TCCAGATGCTTTCACTATCTGAAATGCCATTTCATCTATTGGTTCCATTATTTCTC
TCACAAGAATTATCTTTTTTTTATTTATCTTATGAGAAAGGCTTTCTTCCCAACGT
AAATCTTTGAGAACTTTAAATGTAACAACTTCAATTGAATGGTTCAAGCCCTACC
CCCTAGTG
MEX_SJS
ATTATTAGACAAGAAAGGGGGAAAAAAGAACCTTTTGGACCTTAGGAATAATTTA
TGCCATAATAGCTATCGGATTATTAGGATTCGTAGTTTGAGCTCACCATATATTTA
CAGTAGATAGATGTAGATACCCGAGCTTACTTCACTTCAGCAACTATAATTATTG
CGGTTCCAACTGGAATTAAAGTCTTTAGATGACTAGCCACCTTTCATGGAACTCA
AATTTTAAATAGACCAGTAACCCTGTGAGCTTTAGGTTTTGTATTTTTATTTACAG
TAGGAGGATTAACAGGGGTAATTTTAGCTAATTCTTCAATTGATATTATTCTTCAC
GATACTTACTATGTAGTAGCTCATTTTCACTATGTTCTTTCTATAGGAGCTGTATT
TGCAATTATAGCCGGAATTGTCCAATGATTCCCATTATTTACTGGACTAACCTTA
AATAATTTTTTTTTAAAAACCCAATTTATTACTATATTTTTAGGTGTAAATTTAACAT
TTTTTCCTCAACACTTTTTAGGGCTAAGAGGAATGCCTCGGCGATATTCGGATTA
TCCAGATGCTTTCACTATCTGAAATGCCATTTCATCTATTGGTTCCATTATTTCTC
TCACAAGAATTATCTTTTTTTTATTTATCTTATGAGAAAGGCTTTCTTCCCAACGT
AAATCTTTGAGAACTTTAAATGTAACAACTTCAATTGAATGGTTCAAGCCCTACC
CCCTAGTG
MEX_SJS_1
ATTATTAGACAAGAAAGGGGGAAAAAAGAAGCTTTTGGACCTTAGGAATAATTTA
TGCCATAATACCTATCGGATTATTAGGATTCGTCGTTTGAGCTCACCATATATTTA
CAGTAGATAGATGTAGATACCCGAGCTTACTTCACTTCAGCAACTATAATTATTG
CGGTTCCAACTGGGATTAAAGTCTTTAGATGACTAGCCACCTTTCATGGAACTCA
AATTTTAAATAGACCAGTAACCCTGTGAGCTTTAGGTTTTGTATTTTTATTTACAG
TAGGAGGATTAACAGGGGTAATTTTAGCTAATTCTTCAATTGATATTATTCTTCAC
GATACTTACTATGTAGTAGCTCATTTTCACTATGTTCTTTCTATAGGAGCTGTATT
TGCAATTATAGCCGGAATTATCCAATGATTCCCATTATTTACTGGACTAACCTTAA
ATAATTTTTTTTTAAAAACCCAATTTATTACTATATTTTTAGGTGTAAATTTAACATT
TTTTCCTCAACACTTTTTAGGGCTAAGAGGAATGCCTCGGCGATATTCGGATTAT
CCAGATGCTTTCACTATCTGAAATGCCATTTCATCCATTGGTTCCATTATTTCTCT
CACAAGAATTATCTTTTTTTTATTTATCTTATGAGAAAGGCTTTCTTCCCAACGTA
AATCTTTGAGAACTTCAAATGTAACAACTTCAATTGAATGGTGCAAGCCCTACCC
CCTAGTG
MEX_SJS_3
ATTATTAGACAAGAAAGGGGGAAAAAAGAAGCTTTTGGACCTTAGGAATAATTTA

TGCCATAATACCTATCGGATTATTAGGATTCGTCGTTTGAGCTCACCATATATTTA
CAGTAGATAGATGTAGATACCCGAGCTTACTTCACTTCAGCAACTATAATTATTG
CGGTTCCAACTGGGATTAAAGTCTTTAGATGACTAGCCACCTTTCATGGAACTCA
AATTTTAAATAGACCAGTAACCCTGTGAGCTTTAGGTTTTGTATTTTTATTTACAG
TAGGAGGATTAACAGGGGTAATTTTAGCTAATTCTTCAATTGATATTATTCTTCAC
GATACTTACTATGTAGTAGCTCATTTTCACTATGTTCTTTCTATAGGAGCTGTATT
TGCAATTATAGCCGGAATTATCCAATGATTCCCATTATTTACTGGACTAACCTTAA
ATAATTTTTTTTTAAAAACCCAATTTATTACTATATTTTTAGGTGTAAATTTAACATT
TTTTCCTCAACACTTTTTAGGGCTAAGAGGAATGCCTCGGCGATATTCGGATTAT
CCAGATGCTTTCACTATCTGAAATGCCATTTCATCCATTGGTTCCATTATTTCTCT
CACAAGAATTATCTTTTTTTTATTTATCTTATGAGAAAGGCTTTCTTCCCAACGTA
AATCTTTGAGAACTTCAAATGTAACAACTTCAATTGAATGGTGCAAGCCCTACCC
CCTAGTG
MEX_SPT_1
ATTATTAGACAAGAAAGGGGGAAAAAAGAAGCTTTTGGACCTTAGGAATAATTTA
TGCCATAATAGCTATCGGATTATTAGGATTCGTAGTTTGAGCTCACCATATATTTA
CAGTAGATAGATGTAGATACCCGAGCTTACTTCACTTCAGCAACTATAATTATTG
CGGTTCCAACTGGGATTAAAGTCTTTAGATGACTAGCCACCTTTCATGGAACTCA
AATTTTAAATAGACCAGTAACCCTGTGAGCTCTAGGTTTTGTATTTTTATTTACAG
TAGGAGGATTAACAGGGGTAATTTTAGCTAATTCTTCAATTGATATTATTCTTCAC
GATACTTACTATGTAGTAGCTCATTTTCACTATGTTCTTTCTATAGGAGCTGTATT
TGCAATTATAGCCGGAATTGTCCAATGATTCCCATTATTTACTGGACTAACCTTA
AATAATTTTTTTTTAAAAACCCAATTTATTACTATATTTTTAGGTGTAAATTTAACAT
TTTTTCCTCAACACTTTTTAGGGCTAAGAGGAATGCCTCGGCGATATTCGGATTA
TCCAGATGCTTTCACTATCTGAAATGCCATTTCATCTATTGGTTCCATTATTTCTC
TCACAAGAATTATCTTTTTTTTATTTATCTTATGAGAAAGGCTTTCTTCCCAACGT
AAATCTTTGAGAACTTTAAATGTAACAACTTCAATTGAATGGTTCAAGCCCTACC
CCCTAGTG
MEX_SPT_2
ATTATTAGACAAGAAAGGGGGAAAAAAGAAGCTTTTGGACCTTAGGAATAATTTA
TGCCATAATAGCTATCGGATTATTAGGATTCGTAGTTTGAGCTCACCATATATTTA
CAGTAGATAGATGTAGATACCCGAGCTTACTTCACTTCAGCAACTATAATTATTG
CGGTTCCAACTGGGATTAAAGTCTTTAGATGACTAGCCACCTTTCATGGAACTCT
AATTTTAAATAGACCAGTAACCCTATGAGCTTTAGGTTTTGTATTTTTATTTACAG
TAGGAGGTTTAACAGGAGTAATTTTAGCTAATTCTTCAATTGATATTATTCTTCAC
GATACTTACTATGTAGTAGCTCATTTTCACTATGTTCTTTCTATAGGAGCTGTATT
TGCAATTATAGCCGGAATTGTCCAATGATTCCCATTATTTACTGGACTAACCTTA
AATAATTTTTTTTTAAAAACCCAATTTATTACTATATTTTTAGGTGTAAATTTAACAT
TTTTTCCTCAACACTTTTTAGGGCTAAGAGGAATGCCTCGGCGATATTCGGATTA
TCCAGATGCTTTCACTATCTGAAATGCCATTTCATCTATTGGTTCTATTATTTCTC
TCACAAGAATTATCTTTTTTTTATTTATCTTATGAGAAAGGCTTTCTTCCCAACGT
AAATCTTTGAGAACTTTAAATGTAACAACTTCAATTGAATGGTTCAAGCCCTACC
CCCTAGTG
MEX_SPT_3
ATTATTAGACAAGAAAGGGGGAAAAAAGAAGCTTTTGGACCTTAGGAATAATTTA
TGCCATAATAGCTATCGGATTATTAGGATTCGTAGTTTGAGCTCACCATATATTTA
CAGTAGATAGATGTAGATACCCGAGCTTACTTCACTTCAGCAACTATAATTATTG
CGGTTCCAACTGGGATTAAAGTCTTTAGATGACTAGCCACCTTTCATGGAACTCT
AATTTTAAATAGACCAGTAACCCTATGAGCTTTAGGTTTTGTATTTTTATTTACAG
TAGGAGGTTTAACAGGAGTAATTTTAGCTAATTCTTCAATTGATATTATTCTTCAC

GATACTTACTATGTAGTAGCTCATTTTCACTATGTTCTTTCTATAGGAGCTGTATT
TGCAATTATAGCCGGAATTGTCCAATGATTCCCATTATTTACTGGACTAACCTTA
AATAATTTTTTTTTAAAAACCCAATTTATTACTATATTTTTAGGTGTAAATTTAACAT
TTTTTCCTCAACACTTTTTAGGGCTAAGAGGAATGCCTCGGCGATATTCGGATTA
TCCAGATGCTTTCACTATCTGAAATGCCATTTCATCTATTGGTTCTATTATTTCTC
TCACAAGAATTATCTTTTTTTTTATTTATCTTATGAGAAAGGCTTTCTTCCCAACGT
AAATCTTTGAGAACTTTAAATGTAACAACTTCAATTGAATGGTTCAAGCCCTACC
CCCTAGTG
MEX_TEP
ATTATTAGACAAGAAAGGGGGAAAAAAGAAGCTTTTGGACCTTAGGAATAATTTA
TGCCATAATAGCTATCGGATTATTAGGATTCGTAGTTTGAGCTCACCATATATTTA
CAGTAGATAGATGTAGATACCCGAGCTTACTTCACTTCAGCAACTATAATTATTG
CGGTTCCAACTGGGATTAAAGTCTTTAGATGACTAGCCACCTTTCATGGAACTCA
AATTTTAAATAGACCAGTAACCCTGTGAGCTTTAGGTTTTGTATTTTTATTTACAG
TAGGAGGATTAACAGGGGTAATTTTAGCTAATTCTTCAATTGATATTATTCTTCAC
GATACTTACTATGTAGTAGCTCATTTTCACTATGTTCTTTCTATAGGAGCTGTATT
TGCAATTATAGCCGGAATTGTCCAATGATTCCCATTATTTACTGGACTAACCTTA
AATAATTTTTTTTTAAAAACCCAATTTATTACTATATTTTTAGGTGTAAATTTAACAT
TTTTTCCTCAACACTTTTTAGGGCTAAGAGGAATGCCTCGGCGATATTCGGATTA
TCCAGATGCTTTCACTATCTGAAATGCCATTTCATCTATTGGTTCCATTATTTCTC
TCACAAGAATTATCTTTTTTTTTATTTATCTTATGAGAAAGGCTTTCTTCCCAACGT
AAATCTTTGAGAACTTTAAATGTAACAACTTCAATTGAATGGTTCAAGCCCTACC
CCCTAGTG
MEX_TEP_1
ATTATTAGACAAGAAAGGGGGAAAAAAGAAGCTTTTGGACCTTAGGAATAATTTA
TGCCATAATAGCTATCGGATTATTAGGATTCGTAGTTTGAGCTCACCATATATTTA
CAGTAGATAGATGTAGATACCCGAGCTTACTTCACTTCAGCAACTATAATTATTG
CGGTTCCAACTGGGATTAAAGTCTTTAGATGACTAGCCACCTTTCATGGAACTCA
AATTTTAAATAGACCAGTAACCCTGTGAGCTTTAGGTTTTGTATTTTTATTTACAG
TAGGAGGATTAACAGGGGTAATTTTAGCTAATTCTTCAATTGATATTATTCTTCAC
GATACTTACTATGTAGTAGCTCATTTTCACTATGTTCTTTCTATAGGAGCTGTATT
TGCAATTATAGCCGGAATTGTCCAATGATTCCCATTATTTACTGGACTAACCTTA
AATAATTTTTTTTTAAAAACCCAATTTATTACTATATTTTTAGGTGTAAATTTAACAT
TTTTTCCTCAACACTTTTTAGGGCTAAGAGGAATGCCTCGGCGATATTCGGATTA
TCCAGATGCTTTCACTATCTGAAATGCCATTTCATCTATTGGTTCCATTATTTCTC
TCACAAGAATTATCTTTTTTTTTATTTATCTTATGAGAAAGGCTTTCTTCCCAACGT
AAATCTTTGAGAACTTTAAATGTAACAACTTCAATTGAATGGTTCAAGCCCTACC
CCCTAGTG
MEX_TEP_2
ATTATTAGACAAGAAAGGGGGAAAAAAGAAGCTTTTGGACCTTAGGAATAATTTA
TGCCATAATAGCTATCGGATTATTAGGATTCGTAGTTTGAGCTCACCATATATTTA
CAGTAGATAGATGTAGATACCCGAGCTTACTTCACTTCAGCAACTATAATTATTG
CGGTTCCAACTGGGATTAAAGTCTTTAGATGACTAGCCACCTTTCATGGAACTCA
AATTTTAAATAGACCAGTAACCCTGTGAGCTTTAGGTTTTGTATTTTTATTTACAG
TAGGAGGATTAACAGGGGTAATTTTAGCTAATTCTTCAATTGATATTATTCTTCAC
GATACTTACTATGTAGTAGCTCATTTTCACTATGTTCTTTCTATAGGAGCTGTATT
TGCAATTATAGCCGGAATTGTCCAATGATTTCCATTATTTACTGGACTAACCTTAA
ATAATTTTTTTTTAAAAACCCAATTTATTACTATATTTTTAGGTGTAAATTTAACATT
TTTTCCTCAACACTTTTTAGGGCTAAGAGGAATGCCTCGGCGATATTCGGATTAT
CCAGATGCTTTCACTATCTGAAATGCCATTTCATCTATTGGTTCCATTATTTCTCT

CACAAGAATTATCTTTTTTTTATTTATCTTATGAGAAAGGCTTTCTTCCCAACGTA
AATCTTTGAGAACTTTAAATGTAACAACTTCAATTGAATGGTTCAAGCCCTACCC
CCTAGTG
MEX_TLA
ATTATTAGACAAGAAAGGGGGAAAAAAGAAGCTTTTGGACCTTAGGAATAATTTA
TGCCATAATAGCTATCGGATTATTAGGATTCGTAGTTTGAGCTCACCATATATTTA
CAGTAGATAGATGTAGATACCCGAGCTTACTTCACTTCAGCACCTATGATTATTG
CGGTTCCAACTGGGATTAAAGTCTTTAGATGACTAGCCACCTTTCATGGAACTCA
AATTTTAAATAGACCAGTAACCCTGTGAGCTTTAGGTTTTGTATTTTTATTTACAG
TAGGAGGATTAACAGGGGTAATTTTAGCTAATTCTTCAATTGATATTATTCTTCAC
GATACTTACTATGTAGTAGCTCATTTTCACTATGTTCTTTCTATAGGAGCTGTATT
TGCAATTATAGCCGGAATTGTCCAATGATTCCCATTATTTACTGGACTAACCTTA
AATAATTTTTTTTTAAAAACCCAATTTATTACTATATTTTTAGGTGTAAATTTAACAT
TTTTTCCTCAACACTTTTTAGGGCTAAGAGGAATGCCTCGGCGATATTCGGATTA
TCCAGATGCTTTCACTATCTGAAATGCCATTTCATCTATTGGTTCCATTATTTCTC
TCACAAGAATTATCTTTTTTTTATTTATCTTATGAGAAAGGCTTTCTTCCCAACGT
AAATCTTTGAGAACTTTAAATGTAACAACTTCAATTGAATGGTTCAAGCCCTACC
CCCTAGTG
MEX_TLA_2
ATTATTAGACAAGAAAGGGGGAAAAAAGAACCTTTTGGACCTTAGGAATAATTTA
TCCCTTATTACCTATCGGATTATTAGGATTCGTCGTTTGAGCTCACCATATCTTTA
CAGTAGATAGCTGTAGATACCCGAGCTTACTTCACTTCAGCAACTATAATTATTG
CGGTTCCAACTGGGATTAAAGTCTTTAGATGACTAGCCACCTTTCATGGAACTCA
AATTTTAAATAGACCAGTAACCCTGTGAGCTTTAGGTTTTGTATTTTTATTTACAG
TAGGAGGATTAACAGGGGTAATTTTAGCTAATTCTTCAATTGATATTATTCTTCAC
GATACTTACTATGTAGTAGCTCATTTTCACTATGTTCTTTCTATAGGAGCTGTATT
TGCAATTATAGCCGGAATTAGCCAATGATTCCCATTATTTACTGGACTAACCTTA
AATAATTTTTTTTTAAAAACCCAATTTATTACTATATTTTTAGGTGTAAATTTAACAT
TTTTTCCTCAACACTTTTTAGGGCTAAGAGGAATGCCTCGGCGATATTCGGATTA
TCCAGATGCTTTCACTACCTGAAATGCCATTTCATCCATTGGTTCCATTATTTCTC
TCACAAGAATTATCTTTTTTTTATTTATCTTATGAGAAAGGCTTTCTTCCCAACGT
AAATCTTTGAGAACTTCAAATGTAACAACTTCAATTGAATGGTGCAAGCCCTACC
CCCTAGTG
MEX_TLA_3
ATTATTAGACAAGAAAGGGGGAAAAAAGAAGCTTTTGGACCTTAGGAATAATTTA
TGCCATAATAGCTATCGGATTATTAGGATTCGTAGTTTGAGCTCACCATATATTTA
CAGTAGATAGATGTAGATACCCGAGCTTACTTCACTTCAGCAACTATAATTATTG
CGGTTCCAACTGGGATTAAAGTCTTTAGATGACTAGCCACCTTTCATGGAACTCA
AATTTTAAATAGACCAGTAACCCTGTGAGCTTTAGGTTTTGTATTTTTATTTACAG
TAGGAGGATTAACAGGGGTAATTTTAGCTAATTCTTCAATTGATATTATTCTTCAC
GATACTTACTATGTAGTAGCTCATTTTCACTATGTTCTTTCTATAGGAGCTGTATT
TGCAATTATAGCCGGAATTGTCCAATGATTCCCATTATTTACTGGACTAACCTTA
AATAATTTTTTTTTAAAAACCCAATTTATTACTATATTTTTAGGTGTAAATTTAACAT
TTTTTCCTCAACACTTTTTAGGGCTAAGAGGAATGCCTCGGCGATATTCGGATTA
TCCAGATGCTTTCACTATCTGAAATGCCATTTCATCTATTGGTTCCATTATTTCTC
TCACAAGAATTATCTTTTTTTTATTTATCTTATGAGAAAGGCTTTCTTCCCAACGT
AAATCTTTGAGAACTTTAAATGTAACAACTTCAATTGAATGGTTCAAGCCCTACC
CCCTAGTG
MEX_YAU_1
ATTATTAGACAAGAAAGGGGGAAAAAAGAAGCTTTTGGACCTTAGGAATAATTTA

TGCCATGATAGCTATCGGATTATTAGGATTCGTAGTTTGAGCTCACCATATATTT
ACAGTAGATAGATGTAGATACCCGAGCTTACTTCACTTCAGCAACTATAATTATT
GCGGTTCCAACTGGGATTAAAGTCTTTAGATGGCTAGCCACCTTTCATGGAACT
CAAATTTTAAATAGACCAGTAACCCTGTGAGCTTTAGGTTTTGTATTTTTATTTAC
AGTAGGAGGATTAACAGGGGTAATTTTAGCTAATTCTTCAATTGATATTATTCTTC
ACGATACTTACTATGTAGTAGCTCATTTTCACTATGTTCTTTCTATAGGAGCTGTA
TTTGCAATTATAGCCGGAATTAGCCAATGATTCCCATTATTTACTGGACTAACCTT
AAATAATTTTTTTTTAAAAACCCAATTTATTACTATATTTTTAGGTGTAAATTTAACA
TTTTTTCCTCAACACTTTTTAGGGCTAAGAGGAATGCCTCGGCGATATTCGGATT
ATCCAGATGCTTTCACTATCTGAAATGCCATTTCATCTATTGGTTCCATTATTTCT
CTCACAAGAATTATCTTTTTTTTATTTATCTTATGAGAAAGGCTTTCTTCCCAACG
TAAATCTTTGAGAACTTTAAATGTAACAACTTCAATTGAATGGTTCAAGCCCTACC
CCCTAGTG
MEX_YAU_2
ATTATTAGACAAGAAAGGGGGAAAAAAGAAGCTTTTGGACCTTAGGAATAATTTA
TGCCATAATAGCTATCGGATTATTAGGATTCGTAGTTTGAGCTCACCATATATTTA
CAGTAGATAGATGTAGATACCCGAGCTTACTTCACTTCAGCAACTATAATTATTG
CGGTTCCAACTGGGATTAAAGTCTTTAGATGACTAGCCACCTTTCATGGAACTCA
AATTTTAAATAGACCAGTAACCCTGTGAGCTTTAGGTTTTGTATTTTTATTTACAG
TAGGAGGATTAACAGGGGTAATTTTAGCTAATTCTTCAATTGATATTATTCTTCAC
GATACTTACTATGTAGTAGCTCATTTTCACTATGTTCTTTCTATAGGAGCTGTATT
TGCAATTATAGCCGGAATTGTCCAATGATTCCCATTATTTACTGGACTAACCTTA
AATAATTTTTTTTTAAAAACCCAATTTATTACTATATTTTTAGGTGTAAATTTAACAT
TTTTTCCTCAACACTTTTTAGGGCTAAGAGGAATGCCTCGGCGATATTCGGATTA
TCCAGATGCTTTCACTATCTGAAATGCCATTTCATCTATTGGCTCCATTATTTCTC
TCACAAGAATTATCTTTTTTTTATTTATCTTATGAGAAAGGCTTTCTTCCCAACGT
AAATCTTTGAGAACTTTAAATGTAACAACTTCAATTGAATGGTTCAAGCCCTACC
CCCTAGTG
CAM_YAH
ATTATTAGACAAGAAAGGGGGAAAAAAGAAGCTTTTGGACCTTAGGAATAATTTA
TGCCATGATAGCTATCGGATTATTAGGATTCGTAGTTTGAGCTCTCCATATATTT
ACAGTAGATAGATGTAGATACCCGAGCTTACTTCACTTCAGCAACTATGATTATC
GCGGTTCCAACTGGGATTAAAGTCTTTAGGTGACTAGCCACCTTTCATGGAACT
CAAATTTTAAATAGACCAGTAACCCTGTGAGCTTTAGGTTTTGTATTTTTATTTAC
AGTAGGAGGATTAACAGGGGTAATTTTAGCTAATTCTTCAATTGATATTATTCTTC
ACGATACTTACTATGTAGTAGCTCATTTTCACTATGTTCTTTCTATAGGAGCTGTA
TTTGCAATTATAGCCGGAATTGTCCAATGATTCCCATTATTTACTGGACTAACCTT
AAATAATTTTTTTTTAAAAACCCAATTTATTACTATATTTTTAGGTGTAAATTTAACA
TTTTTTCCTCAACACTTTTTAGGGCTAAGAGGAATGCCTCGGCGATATTCGGATT
ATCCAGATGCTTTCACTATCTGAAATGCCATTTCATCTATTGGTTCCATTATTTCT
CTCACAAGAATTATCTTTTTTTTATTTATCTTATGAGAAAGGCTTTCTTCCCAACG
TAAATCTTTGAGAACTTTAAATGTAACAACTTCAATTGAATGGTTCAAGCCCTACC
CCCTAGTG
CAM_YAH01
ATTATTAGACAAGAAAGGGGGAAAAAAGAAGCTTTTGGACCTTAGGAATAATTTA
TGCCATGATAGCTATCGGATTATTAGGATTCGTAGTTTGAGCTCTCCATATATTT
ACAGTAGATAGATGTAGATACCCGAGCTTACTTCACTTCAGCAACTATGATTATC
GCGGTTCCAACTGGGATTAAAGTCTTTAGGTGACTAGCCACCTTTCATGGAACT
CAAATTTTAAATAGACCAGTAACCCTGTGAGCTTTAGGTTTTGTATTTTTATTTAC
AGTAGGAGGATTAACAGGGGTAATTTTAGCTAATTCTTCAATTGATATTATTCTTC

ACGATACTTACTATGTAGTAGCTCATTTTCACTATGTTCTTTCTATAGGAGCTGTA
TTTGCAATTATAGCCGGAATTGTCCAATGATTCCCATTATTTACTGGACTAACCTT
AAATAATTTTTTTTTAAAAACCCAATTTATTACTATATTTTTAGGTGTAAATTTAACA
TTTTTTCCTCAACACTTTTTAGGGCTAAGAGGAATGCCTCGGCGATATTCGGATT
ATCCAGATGCTTTCACTATCTGAAATGCCATTTCATCTATTGGTTCCATTATTTCT
CTCACAAGAATTATCTTTTTTTTTATTTATCTTATGAGAAAGGCTTTCTTCCCAACG
TAAATCTTTGAGAACTTTAAATGTAACAACTTCAATTGAATGGTTCAAGCCCTACC
CCCTAGTG
CAM_YAH02
ATTATTAGACAAGAAAGGGGGAAAAAAGAAGCTTTTGGACCTTAGGAATAATTTA
TGCCATGATAGCTATCGGATTATTAGGATTCGTAGTTTGAGCTCTCCATATATTT
ACAGTAGATAGATGTAGATACCCGAGCTTACTTCACTTCAGCAACTATGATTATC
GCGGTTCCAACTGGGATTAAAGTCTTTAGGTGACTAGCCACCTTTCATGGAACT
CAAATTTTAAATAGACCAGTAACCCTGTGAGCTTTAGGTTTTGTATTTTTATTTAC
AGTAGGAGGATTAACAGGGGTAATTTTAGCTAATTCTTCAATTGATATTATTCTTC
ACGATACTTACTATGTAGTAGCTCATTTTCACTATGTTCTTTCTATAGGAGCTGTA
TTTGCAATTATAGCCGGAATTGTCCAATGATTCCCATTATTTACTGGACTAACCTT
AAATAATTTTTTTTTAAAAACCCAATTTATTACTATATTTTTAGGTGTAAATTTAACA
TTTTTTCCTCAACACTTTTTAGGGCTAAGAGGAATGCCTCGGCGATATTCGGATT
ATCCAGATGCTTTCACTATCTGAAATGCCATTTCATCTATTGGTTCCATTATTTCT
CTCACAAGAATTATCTTTTTTTTTATTTATCTTATGAGAAAGGCTTTCTTCCCAACG
TAAATCTTTGAGAACTTTAAATGTAACAACTTCAATTGAATGGTTCAAGCCCTACC
CCCTAGTG
China
ATTATTAGACAAGAAAGGGGGAAAAAAGAAGCTTTTGGACCTTAGGAATAATTTA
TGCCATGATAGCTATCGGATTATTAGGATTCGTAGTTTGAGCTCACCATATATTT
ACAGTAGATAGATGTAGATACCCGAGCTTACTTCACTTCAGCAACTATAATTATT
GCGGTTCCAACTGGGATTAAAGTCTTTAGATGACTAGCCACCTTTCATGGAACT
CAAATTTTAAATAGACCAGTAACCCTGTGAGCTTTAGGTTTTGTATTTTTATTTAC
AGTAGGAGGATTAACAGGGGTAATTTTAGCTAATTCTTCAATTGATATTATTCTTC
ACGATACTTACTATGTAGTAGCTCATTTTCACTATGTTCTTTCTATAGGAGCTGTA
TTTGCAATTATAGCCGGAATTGTCCAATGATTCCCATTATTTACCGGACTAACCT
TAAATAATTTTTTTTTAAAAACCCAATTTATTACTATATTTTTAGGTGTAAATTTAAC
ATTTTTTCCCCAACACTTTTTAGGGCTAAGAGGAATGCCTCGGCGATATTCGGAT
TATCCAGATGCTTTCACTATCTGAAATGCCATTTCATCTATTGGTTCCATTATTTC
TCTCACAAGAATTATCTTTTTTTTTATTTATCTTATGAGAAAGGCTTTCTTCCCAAC
GTAAATCTTTGAGAACTTTAAATGTAACAACTTCAATTGAATGGTTCAAGCCCTAC
CCCCTAGTG
FRA_CHA_5
ATTATTAGACAAGAAAGGGGGAAAAAAGAAGCTTTTGGACCTTAGGAATAATTTA
TGCCATGATAGCTATCGGATTATTAGGATTCGTAGTTTGAGCTCACCATATATTT
ACAGTAGATAGATGTAGATACCCGAGCTTACTTCACTTCAGCAACTATAATTATT
GCGGTTCCAACTGGGATTAAAGTCTTTAGATGACTAGCCACCTTTCATGGAACT
CAAATTTTAAATAGACCAGTAACCCTGTGAGCTTTAGGCTTTGTATTTTTATTTAC
AGTAGGAGGATTAACAGGGGTAATTTTAGCTAATTCTTCAATTGATATTATTCTTC
ACGATACTTACTATGTAGTAGCTCATTTTCACTATGTTCTTTCTATAGGAGCTGTA
TTTGCAATTATAGCCGGAATTGTCCAATGATTTCCATTATTTACTGGACTAACCTT
AAATAATTTTTTTTTAAAAACCCAATTTATTACTATATTTTTAGGTGTAAATTTAACA
TTTTTTCCTCAACACTTTTTAGGGCTAAGAGGAATGCCTCGGCGATATTCGGATT
ATCCAGATGCTTTCACTATCTGAAATGCCATTTCATCTATTGGTTCCATTATTTCT

CTCACAAGAATTATCTTTTTTTTATTTATCTTATGAGAAAGGCTTTCTTCCCAACG
TAAATCTTTGAGAACTTTAAATGTAACAACTTCAATTGAATGGTTCAAGCCCTACC
CCCTAGTG
SPA_PRA_1
ATTATTAGACAAGAAAGGGGGAAAAAAGAAGCTTTTGGACCTTAGGAATAATTTA
TGCCATGATAGCTATCGGATTATTAGGATTCGTAGTTTGAGCTCACCATATATTT
ACAGTAGATAGATGTAGATACCCGAGCTTACTTCACTTCAGCAACTATAATTATT
GCGGTTCCAACTGGGATTAAAGTCTTTAGATGACTAGCCACCTTTCATGGAACT
CAAATTTTAAATAGACCAGTAACCCTGTGAGCTTTAGGTTTTGTATTTTTATTTAC
AGTAGGAGGATTAACAGGGGTAATTTTAGCTAATTCTTCAATTGATATTATTCTTC
ACGATACTTACTATGTAGTAGCTCATTTTCACTATGTTCTTTCTATAGGAGCTGTA
TTTGCAATTATAGCCGGAATTGTCCAATGATTCCCATTATTTACCGGACTAACCT
TAAATAATTTTTTTTTAAAAACCCAATTTATTACTATATTTTTAGGTGTAAATTTAAC
ATTTTTTCCCCAACACTTTTTAGGGCTAAGAGGAATGCCTCGGCGATATTCGGAT
TATCCAGATGCTTTCACTATCTGAAATGCCATTTCATCTATTGGTTCCATTATTTC
TCTCACAAGAATTATCTTTTTTTTATTTATCTTATGAGAAAGGCTTTCTTCCCAAC
GTAAATCTTTGAGAACTTTAAATGTAACAACTTCAATTGAATGGTTCAAGCCCTAC
CCCCTAGTG
SWI_NEU_1
ATTATTAGACAAGAAAGGGGGAAAAAAGAAGCTTTTGGACCTTAGGAATAATTTA
TGCCATGATAGCTATCGGATTATTAGGATTCGTAGTTTGAGCTCACCATATATTT
ACAGTAGATAGATGTAGATACCCGAGCTTACTTCACTTCAGCAACTATGATTATC
GCGGTTCCAACTGGGATTAAAGTCTTTAGGTGGCTAGCCACCTTTCATGGAACT
CAAATTTTAAATAGACCAGTAACCCTGTGAGCTTTAGGTTTTGTATTTTTATTTAC
AGTAGGAGGATTAACAGGGGTAATTTTAGCTAATTCTTCAATTGATATTATTCTTC
ACGATACTTACTATGTAGTAGCTCATTTTCACTATGTTCTTTCTATAGGAGCTGTA
TTTGCAATTATAGCCGGAATTGTCCAATGATTCCCATTATTTACTGGACTAACCTT
AAATAATTTTTTTTTAAAAACCCAATTTATTACTATATTTTTAGGTGTAAATTTAACA
TTTTTTCCTCAACACTTTTTAGGGCTAAGAGGAATGCCTCGGCGATATTCGGATT
ATCCAGATGCTTTCACTATCTGAAATGCCATTTCATCTATTGGTTCCATTATTTCT
CTCACAAGAATTATCTTTTTTTTATTTATCTTATGAGAAAGGCTTTCTTCCCAACG
TAAATCTTTGAGAACTTTAAATGTAACAACTTCAATTGAATGGTTCAAGCCCTACC
CCCTAGTG
SWT_NEU
ATTATTAGACAAGAAAGGGGGAAAAAAGAAGCTTTTGGACCTTAGGAATAATTTA
TGCCATGATAGCTATCGGATTATTAGGATTCGTAGTTTGAGCTCACCATATATTT
ACAGTAGATAGATGTAGATACCCGAGCTTACTTCACTTCAGCAACTATGATTATC
GCGGTTCCAACTGGGATTAAAGTCTTTAGGTGGCTAGCCACCTTTCATGGAACT
CAAATTTTAAATAGACCAGTAACCCTGTGAGCTTTAGGTTTTGTATTTTTATTTAC
AGTAGGAGGATTAACAGGGGTAATTTTAGCTAATTCTTCAATTGATATTATTCTTC
ACGATACTTACTATGTAGTAGCTCATTTTCACTATGTTCTTTCTATAGGAGCTGTA
TTTGCAATTATAGCCGGAATTGTCCAATGATTCCCATTATTTACTGGACTAACCTT
AAATAATTTTTTTTTAAAAACCCAATTTATTACTATATTTTTAGGTGTAAATTTAACA
TTTTTTCCTCAACACTTTTTAGGGCTAAGAGGAATGCCTCGGCGATATTCGGATT
ATCCAGATGCTTTCACTATCTGAAATGCCATTTCATCTATTGGTTCCATTATTTCT
CTCACAAGAATTATCTTTTTTTTATTTATCTTATGAGAAAGGCTTTCTTCCCAACG
TAAATCTTTGAGAACTTTAAATGTAACAACTTCAATTGAATGGTTCAAGCCCTACC
CCCTAGTG
SWT_NEU2
ATTATTAGACAAGAAAGGGGGAAAAAAGAAGCTTTTGGACCTTAGGAATAATTTA

TGCCATGATAGCTATCGGATTATTAGGATTCGTAGTTTGAGCTCACCATATATTT
ACAGTAGATAGATGTAGATACCCGAGCTTACTTCACTTCAGCAACTATGATTATC
GCGGTTCCAACTGGGATTAAAGTCTTTAGGTGGCTAGCCACCTTTCATGGAACT
CAAATTTTAAATAGACCAGTAACCCTGTGAGCTTTAGGTTTTGTATTTTTATTTAC
AGTAGGAGGATTAACAGGGGTAATTTTAGCTAATTCTTCAATTGATATTATTCTTC
ACGATACTTACTATGTAGTAGCTCATTTTCACTATGTTCTTTCTATAGGAGCTGTA
TTTGCAATTATAGCCGGAATTGTCCAATGATTCCCATTATTTACTGGACTAACCTT
AAATAATTTTTTTTTAAAAACCCAATTTATTACTATATTTTTAGGTGTAAATTTAACA
TTTTTTCCTCAACACTTTTTAGGGCTAAGAGGAATGCCTCGGCGATATTCGGATT
ATCCAGATGCTTTCACTATCTGAAATGCCATTTCATCTATTGGTTCCATTATTTCT
CTCACAAGAATTATCTTTTTTTTTATTTATCTTATGAGAAAGGCTTTCTTCCCAACG
TAAATCTTTGAGAACTTTAAATGTAACAACTTCAATTGAATGGTTCAAGCCCTACC
CCCTAGTG
MEX_YOH
ATTATTAGACAAGAAAGGGGGAAAAAAGAAGCTTTTGGACCTTAGGAATAATTTA
TGCTATAATAGCTATCGGATTATTAGGATTCGTAGTTTGAGCTCACCATATATTTA
CAGTAGATAGATGTAGATACCCGAGCTTACTTCACTTCAGCAACTATAATTATTG
CGGTTCCAACTGGGATTAAAGTCTTTAGATGACTAGCCACCTTTCATGGAACTCA
AATTTTAAATAGACCAGTAACCCTGTGAGCTTTAGGTTTTGTATTTTTATTTACAG
TAGGAGGATTAACAGGGGTAATTTTAGCTAATTCTTCAATTGATATTATTCTCCAC
GATACTTACTATGTAGTAGCTCATTTTCACTATGTTCTTTCTATAGGAGCTGTATT
TGCAATTATAGCCGGAATTGTCCAATGATTCCCATTATTTACCGGACTAACCTTA
AATAATTTTTTTTTAAAAACCCAATTTATTACTATATTTTTAGGTGTAAATTTAACAT
TTTTTCCTCAACACTTTTTAGGGCTAAGAGGAATGCCTCGGCGATATTCGGATTA
TCCAGATGCTTTCACTATCTGAAATGCCATTTCATCTATTGGTTCCATTATTTCTC
TCACAAGAATTATCTTTTTTTTTATTTATCTTATGAGAAAGGCTTTCTTCCCAACGT
AAATCTTTGAGAACTTTAAATGTAACAACTTCAATTGAATGGTTCAAGCCCTACC
CCCTAGTG
MEX_YOH_1
ATTATTAGACAAGAAAGGGGGAAAAAAGAAGCTTTTGGACCTTAGGAATAATTTA
TGCTATAATAGCTATCGGATTATTAGGATTCGTAGTTTGAGCTCACCATATATTTA
CAGTAGATAGATGTAGATACCCGAGCTTACTTCACTTCAGCAACTATAATTATTG
CGGTTCCAACTGGGATTAAAGTCTTTAGATGACTAGCCACCTTTCATGGAACTCA
AATTTTAAATAGACCAGTAACCCTGTGAGCTTTAGGTTTTGTATTTTTATTTACAG
TAGGAGGATTAACAGGGGTAATTTTAGCTAATTCTTCAATTGATATTATTCTCCAC
GATACTTACTATGTAGTAGCTCATTTTCACTATGTTCTTTCTATAGGAGCTGTATT
TGCAATTATAGCCGGAATTGTCCAATGATTCCCATTATTTACCGGACTAACCTTA
AATAATTTTTTTTTAAAAACCCAATTTATTACTATATTTTTAGGTGTAAATTTAACAT
TTTTTCCTCAACACTTTTTAGGGCTAAGAGGAATGCCTCGGCGATATTCGGATTA
TCCAGATGCTTTCACTATCTGAAATGCCATTTCATCTATTGGTTCCATTATTTCTC
TCACAAGAATTATCTTTTTTTTTATTTATCTTATGAGAAAGGCTTTCTTCCCAACGT
AAATCTTTGAGAACTTTAAATGTAACAACTTCAATTGAATGGTTCAAGCCCTACC
CCCTAGTG
MEX_YOH_2
ATTATTAGACAAGAAAGGGGGAAAAAAGAAGCTTTTGGACCTTAGGAATAATTTA
TGCTATAATAGCTATCGGATTATTAGGATTCGTAGTTTGAGCTCACCATATATTTA
CAGTAGATAGATGTAGATACCCGAGCTTACTTCACTTCAGCAACTATAATTATTG
CGGTTCCAACTGGGATTAAAGTCTTTAGATGACTAGCCACCTTTCATGGAACTCA
AATTTTAAATAGACCAGTAACCCTGTGAGCTTTAGGTTTTGTATTTTTATTTACAG
TAGGAGGATTAACAGGGGTAATTTTAGCTAATTCTTCAATTGATATTATTCTCCAC

GATACTTACTATGTAGTAGCTCATTTTCACTATGTTCTTTCTATAGGAGCTGTATT
TGCAATTATAGCCGGAATTGTCCAATGATTCCCATTATTTACCGGACTAACCTTA
AATAATTTTTTTTTAAAAACCCAATTTATTACTATATTTTTAGGTGTAAATTTAACAT
TTTTTCCTCAACACTTTTTAGGGCTAAGAGGAATGCCTCGGCGATATTCGGATTA
TCCAGATGCTTTCACTATCTGAAATGCCATTTCATCTATTGGTTCCATTATTTCTC
TCACAAGAATTATCTTTTTTTTTATTTATCTTATGAGAAAGGCTTTCTTCCCAACGT
AAATCTTTGAGAACTTTAAATGTAACAACTTCAATTGAATGGTTCAAGCCCTACC
CCCTAGTG
AOPE_15
ATTATTAGACAAGAAAGGGGGAAAAAAGAAGCTTTTGGACCTTAGGAATAATTTA
TGCTATAATAGCTATCGGATTATTAGGATTCGTAGTTTGAGCTCACCATATATTTA
CAGTAGATAGATGTAGATACCCGAGCTTACTTCACTTCAGCAACTATAATTATTG
CGGTTCCAACTGGGATTAAAGTCTTTAGATGACTAGCCACCTTTCATGGAACTCA
AATTTTAAATAGACCAGTAACCCTGTGAGCTTTAGGTTTTGTATTTTTATTTACAG
TAGGAGGATTAACAGGGGTAATTTTAGCTAATTCTTCAATTGATATTATTCTCCAC
GATACTTACTATGTAGTAGCTCATTTTCACTATGTTCTTTCTATAGGAGCTGTATT
TGCAATTATAGCCGGAATTGTCCAATGATTCCCATTATTTACCGGACTAACCTTA
AATAATTTTTTTTTAAAAACCCAATTTATTACTATATTTTTAGGTGTAAATTTAACAT
TTTTTCCTCAACACTTTTTAGGGCTAAGAGGAATGCCTCGGCGATATTCGGATTA
TCCAGATGCTTTCACTATCTGAAATGCCATTTCATCTATTGGTTCCATTATTTCTC
TCACAAGAATTATCTTTTTTTTTATTTATCTTATGAGAAAGGCTTTCTTCCCAACGT
AAATCTTTGAGAACTTTAAATGTAACAACTTCAATTGAATGGTTCAAGCCCTACC
CCCTAGTG
AOPE_2
ATTATTAGACAAGAAAGGGGGAAAAAAGAAGCTTTTGGACCTTAGGAATAATTTA
TGCTATAATAGCTATCGGATTATTAGGATTCGTAGTTTGAGCTCACCATATATTTA
CAGTAGATAGATGTAGATACCCGAGCTTACTTCACTTCAGCAACTATAATTATTG
CGGTTCCAACTGGGATTAAAGTCTTTAGATGACTAGCCACCTTTCATGGAACTCA
AATTTTAAATAGACCAGTAACCCTGTGAGCTTTAGGTTTTGTATTTTTATTTACAG
TAGGAGGATTAACAGGGGTAATTTTAGCTAATTCTTCAATTGATATTATTCTCCAC
GATACTTACTATGTAGTAGCTCATTTTCACTATGTTCTTTCTATAGGAGCTGTATT
TGCAATTATAGCCGGAATTGTCCAATGATTCCCATTATTTACCGGACTAACCTTA
AATAATTTTTTTTTAAAAACCCAATTTATTACTATATTTTTAGGTGTAAATTTAACAT
TTTTTCCTCAACACTTTTTAGGGCTAAGAGGAATGCCTCGGCGATATTCGGATTA
TCCAGATGCTTTCACTATCTGAAATGCCATTTCATCTATTGGTTCCATTATTTCTC
TCACAAGAATTATCTTTTTTTTTATTTATCTTATGAGAAAGGCTTTCTTCCCAACGT
AAATCTTTGAGAACTTTAAATGTAACAACTTCAATTGAATGGTTCAAGCCCTACC
CCCTAGTG
AOPE_3
ATTATTAGACAAGAAAGGGGGAAAAAAGAAGCTTTTGGACCTTAGGAATAATTTA
TGCTATAATAGCTATCGGATTATTAGGATTCGTAGTTTGAGCTCACCATATATTTA
CAGTAGATAGATGTAGATACCCGAGCTTACTTCACTTCAGCAACTATAATTATTG
CGGTTCCAACTGGGATTAAAGTCTTTAGATGACTAGCCACCTTTCATGGAACTCA
AATTTTAAATAGACCAGTAACCCTGTGAGCTTTAGGTTTTGTATTTTTATTTACAG
TAGGAGGATTAACAGGGGTAATTTTAGCTAATTCTTCAATTGATATTATTCTCCAC
GATACTTACTATGTAGTAGCTCATTTTCACTATGTTCTTTCTATAGGAGCTGTATT
TGCAATTATAGCCGGAATTGTCCAATGATTCCCATTATTTACCGGACTAACCTTA
AATAATTTTTTTTTAAAAACCCAATTTATTACTATATTTTTAGGTGTAAATTTAACAT
TTTTTCCTCAACACTTTTTAGGGCTAAGAGGAATGCCTCGGCGATATTCGGATTA
TCCAGATGCTTTCACTATCTGAAATGCCATTTCATCTATTGGTTCCATTATTTCTC

TCACAAGAATTATCTTTTTTTTATTTATCTTATGAGAAAGGCTTTCTTCCCAACGT
AAATCTTTGAGAACTTTAAATGTAACAACTTCAATTGAATGGTTCAAGCCCTACC
CCCTAGTG
AOPE_12
ATTATTAGACAAGAAAGGGGGAAAAAAGAAGCTTTTGGACCTTAGGAATAATTTA
TGCTATAATAGCTATCGGATTATTAGGATTCGTAGTTTGAGCTCACCATATATTTA
CAGTAGATAGATGTAGATACCCGAGCTTACTTCACTTCAGCAACTATAATTATTG
CGGTTCCAACTGGGATTAAAGTCTTTAGATGACTAGCCACCTTTCATGGAACTCA
AATTTTAAATAGACCAGTAACCCTGTGAGCTTTAGGTTTTGTATTTTTATTTACAG
TAGGAGGATTAACAGGGGTAATTTTAGCTAATTCTTCAATTGATATTATTCTCCAC
GATACTTACTATGTAGTAGCTCATTTTCACTATGTTCTTTCTATAGGAGCTGTATT
TGCAATTATAGCCGGAATTGTCCAATGATTCCCATTATTTACCGGACTAACCTTA
AATAATTTTTTTTTAAAAACCCAATTTATTACTATATTTTTAGGTGTAAATTTAACAT
TTTTTCCTCAACACTTTTTAGGGCTAAGAGGAATGCCTCGGCGATATTCGGATTA
TCCAGATGCTTTCACTATCTGAAATGCCATTTCATCTATTGGTTCCATTATTTCTC
TCACAAGAATTATCTTTTTTTTATTTATCTTATGAGAAAGGCTTTCTTCCCAACGT
AAATCTTTGAGAACTTTAAATGTAACAACTTCAATTGAATGGTTCAAGCCCTACC
CCCTAGTG
AOPE_13
ATTATTAGACAAGAAAGGGGGAAAAAAGAAGCTTTTGGACCTTAGGAATAATTTA
TGCTATAATAGCTATCGGATTATTAGGATTCGTAGTTTGAGCTCACCATATATTTA
CAGTAGATAGATGTAGATACCCGAGCTTACTTCACTTCAGCAACTATAATTATTG
CGGTTCCAACTGGGATTAAAGTCTTTAGATGACTAGCCACCTTTCATGGAACTCA
AATTTTAAATAGACCAGTAACCCTGTGAGCTTTAGGTTTTGTATTTTTATTTACAG
TAGGAGGATTAACAGGGGTAATTTTAGCTAATTCTTCAATTGATATTATTCTCCAC
GATACTTACTATGTAGTAGCTCATTTTCACTATGTTCTTTCTATAGGAGCTGTATT
TGCAATTATAGCCGGAATTGTCCAATGATTCCCATTATTTACCGGACTAACCTTA
AATAATTTTTTTTTAAAAACCCAATTTATTACTATATTTTTAGGTGTAAATTTAACAT
TTTTTCCTCAACACTTTTTAGGGCTAAGAGGAATGCCTCGGCGATATTCGGATTA
TCCAGATGCTTTCACTATCTGAAATGCCATTTCATCTATTGGTTCCATTATTTCTC
TCACAAGAATTATCTTTTTTTTATTTATCTTATGAGAAAGGCTTTCTTCCCAACGT
AAATCTTTGAGAACTTTAAATGTAACAACTTCAATTGAATGGTTCAAGCCCTACC
CCCTAGTG
AOPE_14
ATTATTAGACAAGAAAGGGGGAAAAAAGAAGCTTTTGGACCTTAGGAATAATTTA
TGCTATAATAGCTATCGGATTATTAGGATTCGTAGTTTGAGCTCACCATATATTTA
CAGTAGATAGATGTAGATACCCGAGCTTACTTCACTTCAGCAACTATAATTATTG
CGGTTCCAACTGGGATTAAAGTCTTTAGATGACTAGCCACCTTTCATGGAACTCA
AATTTTAAATAGACCAGTAACCCTGTGAGCTTTAGGTTTTGTATTTTTATTTACAG
TAGGAGGATTAACAGGGGTAATTTTAGCTAATTCTTCAATTGATATTATTCTCCAC
GATACTTACTATGTAGTAGCTCATTTTCACTATGTTCTTTCTATAGGAGCTGTATT
TGCAATTATAGCCGGAATTGTCCAATGATTCCCATTATTTACCGGACTAACCTTA
AATAATTTTTTTTTAAAAACCCAATTTATTACTATATTTTTAGGTGTAAATTTAACAT
TTTTTCCTCAACACTTTTTAGGGCTAAGAGGAATGCCTCGGCGATATTCGGATTA
TCCAGATGCTTTCACTATCTGAAATGCCATTTCATCTATTGGTTCCATTATTTCTC
TCACAAGAATTATCTTTTTTTTATTTATCTTATGAGAAAGGCTTTCTTCCCAACGT
AAATCTTTGAGAACTTTAAATGTAACAACTTCAATTGAATGGTTCAAGCCCTACC
CCCTAGTG
AOPE_4
ATTATTAGACAAGAAAGGGGGAAAAAAGAAGCTTTTGGACCTTAGGAATAATTTA

TGCTATAATAGCTATCGGATTATTAGGATTCGTAGTTTGAGCTCACCATATATTTA
CAGTAGATAGATGTAGATACCCGAGCTTACTTCACTTCAGCAACTATAATTATTG
CGGTTCCAACTGGGATTAAAGTCTTTAGATGACTAGCCACCTTTCATGGAACTCA
AATTTTAAATAGACCAGTAACCCTGTGAGCTTTAGGTTTTGTATTTTTATTTACAG
TAGGAGGATTAACAGGGGTAATTTTAGCTAATTCTTCAATTGATATTATTCTCCAC
GATACTTACTATGTAGTAGCTCATTTTCACTATGTTCTTTCTATAGGAGCTGTATT
TGCAATTATAGCCGGAATTGTCCAATGATTCCCATTATTTACCGGACTAACCTTA
AATAATTTTTTTTTAAAAACCCAATTTATTACTATATTTTTAGGTGTAAATTTAACAT
TTTTTCCTCAACACTTTTTAGGGCTAAGAGGAATGCCTCGGCGATATTCGGATTA
TCCAGATGCTTTCACTATCTGAAATGCCATTTCATCTATTGGTTCCATTATTTCTC
TCACAAGAATTATCTTTTTTTTTATTTATCTTATGAGAAAGGCTTTCTTCCCAACGT
AAATCTTTGAGAACTTTAAATGTAACAACTTCAATTGAATGGTTCAAGCCCTACC
CCCTAGTG
PE2_1
ATTATTAGACAAGAAAGGGGGAAAAAAGAAGCTTTTGGACCTTAGGAATAATTTA
TGCTATAATAGCTATCGGATTATTAGGATTCGTAGTTTGAGCTCACCATATATTTA
CAGTAGATAGATGTAGATACCCGAGCTTACTTCACTTCAGCAACTATAATTATTG
CGGTTCCAACTGGGATTAAAGTCTTTAGATGACTAGCCACCTTTCATGGAACTCA
AATTTTAAATAGACCAGTAACCCTGTGAGCTTTAGGTTTTGTATTTTTATTTACAG
TAGGAGGATTAACAGGGGTAATTTTAGCTAATTCTTCAATTGATATTATTCTCCAC
GATACTTACTATGTAGTAGCTCATTTTCACTATGTTCTTTCTATAGGAGCTGTATT
TGCAATTATAGCCGGAATTGTCCAATGATTCCCATTATTTACCGGACTAACCTTA
AATAATTTTTTTTTAAAAACCCAATTTATTACTATATTTTTAGGTGTAAATTTAACAT
TTTTTCCTCAACACTTTTTAGGGCTAAGAGGAATGCCTCGGCGATATTCGGATTA
TCCAGATGCTTTCACTATCTGAAATGCCATTTCATCTATTGGTTCCATTATTTCTC
TCACAAGAATTATCTTTTTTTTTATTTATCTTATGAGAAAGGCTTTCTTCCCAACGT
AAATCTTTGAGAACTTTAAATGTAACAACTTCAATTGAATGGTTCAAGCCCTACC
CCCTAGTG
PE2_2
ATTATTAGACAAGAAAGGGGGAAAAAAGAAGCTTTTGGACCTTAGGAATAATTTA
TGCTATAATAGCTATCGGATTATTAGGATTCGTAGTTTGAGCTCACCATATATTTA
CAGTAGATAGATGTAGATACCCGAGCTTACTTCACTTCAGCAACTATAATTATTG
CGGTTCCAACTGGGATTAAAGTCTTTAGATGACTAGCCACCTTTCATGGAACTCA
AATTTTAAATAGACCAGTAACCCTGTGAGCTTTAGGTTTTGTATTTTTATTTACAG
TAGGAGGATTAACAGGGGTAATTTTAGCTAATTCTTCAATTGATATTATTCTCCAC
GATACTTACTATGTAGTAGCTCATTTTCACTATGTTCTTTCTATAGGAGCTGTATT
TGCAATTATAGCCGGAATTGTCCAATGATTCCCATTATTTACCGGACTAACCTTA
AATAATTTTTTTTTAAAAACCCAATTTATTACTATATTTTTAGGTGTAAATTTAACAT
TTTTTCCTCAACACTTTTTAGGGCTAAGAGGAATGCCTCGGCGATATTCGGATTA
TCCAGATGCTTTCACTATCTGAAATGCCATTTCATCTATTGGTTCCATTATTTCTC
TCACAAGAATTATCTTTTTTTTTATTTATCTTATGAGAAAGGCTTTCTTCCCAACGT
AAATCTTTGAGAACTTTAAATGTAACAACTTCAATTGAATGGTTCAAGCCCTACC
CCCTAGTG
PE2_3
ATTATTAGACAAGAAAGGGGGAAAAAAGAAGCTTTTGGACCTTAGGAATAATTTA
TGCTATAATAGCTATCGGATTATTAGGATTCGTAGTTTGAGCTCACCATATATTTA
CAGTAGATAGATGTAGATACCCGAGCTTACTTCACTTCAGCAACTATAATTATTG
CGGTTCCAACTGGGATTAAAGTCTTTAGATGACTAGCCACCTTTCATGGAACTCA
AATTTTAAATAGACCAGTAACCCTGTGAGCTTTAGGTTTTGTATTTTTATTTACAG
TAGGAGGATTAACAGGGGTAATTTTAGCTAATTCTTCAATTGATATTATTCTCCAC

GATACTTACTATGTAGTAGCTCATTTTCACTATGTTCTTTCTATAGGAGCTGTATT
TGCAATTATAGCCGGAATTGTCCAATGATTCCCATTATTTACCGGACTAACCTTA
AATAATTTTTTTTTAAAAACCCAATTTATTACTATATTTTTAGGTGTAAATTTAACAT
TTTTTCCTCAACACTTTTTAGGGCTAAGAGGAATGCCTCGGCGATATTCGGATTA
TCCAGATGCTTTCACTATCTGAAATGCCATTTCATCTATTGGTTCCATTATTTCTC
TCACAAGAATTATCTTTTTTTTATTTATCTTATGAGAAAGGCTTTCTTCCCAACGT
AAATCTTTGAGAACTTTAAATGTAACAACTTCAATTGAATGGTTCAAGCCCTACC
CCCTAGTG
PE2_4
ATTATTAGACAAGAAAGGGGGAAAAAAGAAGCTTTTGGACCTTAGGAATAATTTA
TGCTATAATAGCTATCGGATTATTAGGATTCGTAGTTTGAGCTCACCATATATTTA
CAGTAGATAGATGTAGATACCCGAGCTTACTTCACTTCAGCAACTATAATTATTG
CGGTTCCAACTGGGATTAAAGTCTTTAGATGACTAGCCACCTTTCATGGAACTCA
AATTTTAAATAGACCAGTAACCCTGTGAGCTTTAGGTTTTGTATTTTTATTTACAG
TAGGAGGATTAACAGGGGTAATTTTAGCTAATTCTTCAATTGATATTATTCTCCAC
GATACTTACTATGTAGTAGCTCATTTTCACTATGTTCTTTCTATAGGAGCTGTATT
TGCAATTATAGCCGGAATTGTCCAATGATTCCCATTATTTACCGGACTAACCTTA
AATAATTTTTTTTTAAAAACCCAATTTATTACTATATTTTTAGGTGTAAATTTAACAT
TTTTTCCTCAACACTTTTTAGGGCTAAGAGGAATGCCTCGGCGATATTCGGATTA
TCCAGATGCTTTCACTATCTGAAATGCCATTTCATCTATTGGTTCCATTATTTCTC
TCACAAGAATTATCTTTTTTTTATTTATCTTATGAGAAAGGCTTTCTTCCCAACGT
AAATCTTTGAGAACTTTAAATGTAACAACTTCAATTGAATGGTTCAAGCCCTACC
CCCTAGTG
PE2_5
ATTATTAGACAAGAAAGGGGGAAAAAAGAAGCTTTTGGACCTTAGGAATAATTTA
TGCTATAATAGCTATCGGATTATTAGGATTCGTAGTTTGAGCTCACCATATATTTA
CAGTAGATAGATGTAGATACCCGAGCTTACTTCACTTCAGCAACTATAATTATTG
CGGTTCCAACTGGGATTAAAGTCTTTAGATGACTAGCCACCTTTCATGGAACTCA
AATTTTAAATAGACCAGTAACCCTGTGAGCTTTAGGTTTTGTATTTTTATTTACAG
TAGGAGGATTAACAGGGGTAATTTTAGCTAATTCTTCAATTGATATTATTCTCCAC
GATACTTACTATGTAGTAGCTCATTTTCACTATGTTCTTTCTATAGGAGCTGTATT
TGCAATTATAGCCGGAATTGTCCAATGATTCCCATTATTTACCGGACTAACCTTA
AATAATTTTTTTTTAAAAACCCAATTTATTACTATATTTTTAGGTGTAAATTTAACAT
TTTTTCCTCAACACTTTTTAGGGCTAAGAGGAATGCCTCGGCGATATTCGGATTA
TCCAGATGCTTTCACTATCTGAAATGCCATTTCATCTATTGGTTCCATTATTTCTC
TCACAAGAATTATCTTTTTTTTATTTATCTTATGAGAAAGGCTTTCTTCCCAACGT
AAATCTTTGAGAACTTTAAATGTAACAACTTCAATTGAATGGTTCAAGCCCTACC
CCCTAGTG
PE2_6
ATTATTAGACAAGAAAGGGGGAAAAAAGAAGCTTTTGGACCTTAGGAATAATTTA
TGCTATAATAGCTATCGGATTATTAGGATTCGTAGTTTGAGCTCACCATATATTTA
CAGTAGATAGATGTAGATACCCGAGCTTACTTCACTTCAGCAACTATAATTATTG
CGGTTCCAACTGGGATTAAAGTCTTTAGATGACTAGCCACCTTTCATGGAACTCA
AATTTTAAATAGACCAGTAACCCTGTGAGCTTTAGGTTTTGTATTTTTATTTACAG
TAGGAGGATTAACAGGGGTAATTTTAGCTAATTCTTCAATTGATATTATTCTCCAC
GATACTTACTATGTAGTAGCTCATTTTCACTATGTTCTTTCTATAGGAGCTGTATT
TGCAATTATAGCCGGAATTGTCCAATGATTCCCATTATTTACCGGACTAACCTTA
AATAATTTTTTTTTAAAAACCCAATTTATTACTATATTTTTAGGTGTAAATTTAACAT
TTTTTCCTCAACACTTTTTAGGGCTAAGAGGAATGCCTCGGCGATATTCGGATTA
TCCAGATGCTTTCACTATCTGAAATGCCATTTCATCTATTGGTTCCATTATTTCTC

TCACAAGAATTATCTTTTTTTTATTTATCTTATGAGAAAGGCTTTCTTCCCAACGT
AAATCTTTGAGAACTTTAAATGTAACAACTTCAATTGAATGGTTCAAGCCCTACC
CCCTAGTG
PE2_7
ATTATTAGACAAGAAAGGGGGAAAAAAGAAGCTTTTGGACCTTAGGAATAATTTA
TGCTATAATAGCTATCGGATTATTAGGATTCGTAGTTTGAGCTCACCATATATTTA
CAGTAGATAGATGTAGATACCCGAGCTTACTTCACTTCAGCAACTATAATTATTG
CGGTTCCAACTGGGATTAAAGTCTTTAGATGACTAGCCACCTTTCATGGAACTCA
AATTTTAAATAGACCAGTAACCCTGTGAGCTTTAGGTTTTGTATTTTTATTTACAG
TAGGAGGATTAACAGGGGTAATTTTAGCTAATTCTTCAATTGATATTATTCTCCAC
GATACTTACTATGTAGTAGCTCATTTTCACTATGTTCTTTCTATAGGAGCTGTATT
TGCAATTATAGCCGGAATTGTCCAATGATTCCCATTATTTACCGGACTAACCTTA
AATAATTTTTTTTTAAAAACCCAATTTATTACTATATTTTTAGGTGTAAATTTAACAT
TTTTTCCTCAACACTTTTTAGGGCTAAGAGGAATGCCTCGGCGATATTCGGATTA
TCCAGATGCTTTCACTATCTGAAATGCCATTTCATCTATTGGTTCCATTATTTCTC
TCACAAGAATTATCTTTTTTTTATTTATCTTATGAGAAAGGCTTTCTTCCCAACGT
AAATCTTTGAGAACTTTAAATGTAACAACTTCAATTGAATGGTTCAAGCCCTACC
CCCTAGTG
PE3_1
ATTATTAGACAAGAAAGGGGGAAAAAAGAAGCTTTTGGACCTTAGGAATAATTTA
TGCTATAATAGCTATCGGATTATTAGGATTCGTAGTTTGAGCTCACCATATATTTA
CAGTAGATAGATGTAGATACCCGAGCTTACTTCACTTCAGCAACTATAATTATTG
CGGTTCCAACTGGGATTAAAGTCTTTAGATGACTAGCCACCTTTCATGGAACTCA
AATTTTAAATAGACCAGTAACCCTGTGAGCTTTAGGTTTTGTATTTTTATTTACAG
TAGGAGGATTAACAGGGGTAATTTTAGCTAATTCTTCAATTGATATTATTCTCCAC
GATACTTACTATGTAGTAGCTCATTTTCACTATGTTCTTTCTATAGGAGCTGTATT
TGCAATTATAGCCGGAATTGTCCAATGATTCCCATTATTTACCGGACTAACCTTA
AATAATTTTTTTTTAAAAACCCAATTTATTACTATATTTTTAGGTGTAAATTTAACAT
TTTTTCCTCAACACTTTTTAGGGCTAAGAGGAATGCCTCGGCGATATTCGGATTA
TCCAGATGCTTTCACTATCTGAAATGCCATTTCATCTATTGGTTCCATTATTTCTC
TCACAAGAATTATCTTTTTTTTATTTATCTTATGAGAAAGGCTTTCTTCCCAACGT
AAATCTTTGAGAACTTTAAATGTAACAACTTCAATTGAATGGTTCAAGCCCTACC
CCCTAGTG
PE4_1
ATTATTAGACAAGAAAGGGGGAAAAAAGAAGCTTTTGGACCTTAGGAATAATTTA
TGCTATAATAGCTATCGGATTATTAGGATTCGTAGTTTGAGCTCACCATATATTTA
CAGTAGATAGATGTAGATACCCGAGCTTACTTCACTTCAGCAACTATAATTATTG
CGGTTCCAACTGGGATTAAAGTCTTTAGATGACTAGCCACCTTTCATGGAACTCA
AATTTTAAATAGACCAGTAACCCTGTGAGCTTTAGGTTTTGTATTTTTATTTACAG
TAGGAGGATTAACAGGGGTAATTTTAGCTAATTCTTCAATTGATATTATTCTCCAC
GATACTTACTATGTAGTAGCTCATTTTCACTATGTTCTTTCTATAGGAGCTGTATT
TGCAATTATAGCCGGAATTGTCCAATGATTCCCATTATTTACCGGACTAACCTTA
AATAATTTTTTTTTAAAAACCCAATTTATTACTATATTTTTAGGTGTAAATTTAACAT
TTTTTCCTCAACACTTTTTAGGGCTAAGAGGAATGCCTCGGCGATATTCGGATTA
TCCAGATGCTTTCACTATCTGAAATGCCATTTCATCTATTGGTTCCATTATTTCTC
TCACAAGAATTATCTTTTTTTTATTTATCTTATGAGAAAGGCTTTCTTCCCAACGT
AAATCTTTGAGAACTTTAAATGTAACAACTTCAATTGAATGGTTCAAGCCCTACC
CCCTAGTG
PE4_2
ATTATTAGACAAGAAAGGGGGAAAAAAGAAGCTTTTGGACCTTAGGAATAATTTA

TGCTATAATAGCTATCGGATTATTAGGATTCGTAGTTTGAGCTCACCATATATTTA
CAGTAGATAGATGTAGATACCCGAGCTTACTTCACTTCAGCAACTATAATTATTG
CGGTTCCAACTGGGATTAAAGTCTTTAGATGACTAGCCACCTTTCATGGAACTCA
AATTTTAAATAGACCAGTAACCCTGTGAGCTTTAGGTTTTGTATTTTTATTTACAG
TAGGAGGATTAACAGGGGTAATTTTAGCTAATTCTTCAATTGATATTATTCTCCAC
GATACTTACTATGTAGTAGCTCATTTTCACTATGTTCTTTCTATAGGAGCTGTATT
TGCAATTATAGCCGGAATTGTCCAATGATTCCCATTATTTACCGGACTAACCTTA
AATAATTTTTTTTTAAAAACCCAATTTATTACTATATTTTTAGGTGTAAATTTAACAT
TTTTTCCTCAACACTTTTTAGGGCTAAGAGGAATGCCTCGGCGATATTCGGATTA
TCCAGATGCTTTCACTATCTGAAATGCCATTTCATCTATTGGTTCCATTATTTCTC
TCACAAGAATTATCTTTTTTTTTATTTATCTTATGAGAAAGGCTTTCTTCCCAACGT
AAATCTTTGAGAACTTTAAATGTAACAACTTCAATTGAATGGTTCAAGCCCTACC
CCCTAGTG
PE4_3
ATTATTAGACAAGAAAGGGGGAAAAAAGAAGCTTTTGGACCTTAGGAATAATTTA
TGCTATAATAGCTATCGGATTATTAGGATTCGTAGTTTGAGCTCACCATATATTTA
CAGTAGATAGATGTAGATACCCGAGCTTACTTCACTTCAGCAACTATAATTATTG
CGGTTCCAACTGGGATTAAAGTCTTTAGATGACTAGCCACCTTTCATGGAACTCA
AATTTTAAATAGACCAGTAACCCTGTGAGCTTTAGGTTTTGTATTTTTATTTACAG
TAGGAGGATTAACAGGGGTAATTTTAGCTAATTCTTCAATTGATATTATTCTCCAC
GATACTTACTATGTAGTAGCTCATTTTCACTATGTTCTTTCTATAGGAGCTGTATT
TGCAATTATAGCCGGAATTGTCCAATGATTCCCATTATTTACCGGACTAACCTTA
AATAATTTTTTTTTAAAAACCCAATTTATTACTATATTTTTAGGTGTAAATTTAACAT
TTTTTCCTCAACACTTTTTAGGGCTAAGAGGAATGCCTCGGCGATATTCGGATTA
TCCAGATGCTTTCACTATCTGAAATGCCATTTCATCTATTGGTTCCATTATTTCTC
TCACAAGAATTATCTTTTTTTTTATTTATCTTATGAGAAAGGCTTTCTTCCCAACGT
AAATCTTTGAGAACTTTAAATGTAACAACTTCAATTGAATGGTTCAAGCCCTACC
CCCTAGTG
PE4_5
ATTATTAGACAAGAAAGGGGGAAAAAAGAAGCTTTTGGACCTTAGGAATAATTTA
TGCTATAATAGCTATCGGATTATTAGGATTCGTAGTTTGAGCTCACCATATATTTA
CAGTAGATAGATGTAGATACCCGAGCTTACTTCACTTCAGCAACTATAATTATTG
CGGTTCCAACTGGGATTAAAGTCTTTAGATGACTAGCCACCTTTCATGGAACTCA
AATTTTAAATAGACCAGTAACCCTGTGAGCTTTAGGTTTTGTATTTTTATTTACAG
TAGGAGGATTAACAGGGGTAATTTTAGCTAATTCTTCAATTGATATTATTCTCCAC
GATACTTACTATGTAGTAGCTCATTTTCACTATGTTCTTTCTATAGGAGCTGTATT
TGCAATTATAGCCGGAATTGTCCAATGATTCCCATTATTTACCGGACTAACCTTA
AATAATTTTTTTTTAAAAACCCAATTTATTACTATATTTTTAGGTGTAAATTTAACAT
TTTTTCCTCAACACTTTTTAGGGCTAAGAGGAATGCCTCGGCGATATTCGGATTA
TCCAGATGCTTTCACTATCTGAAATGCCATTTCATCTATTGGTTCCATTATTTCTC
TCACAAGAATTATCTTTTTTTTTATTTATCTTATGAGAAAGGCTTTCTTCCCAACGT
AAATCTTTGAGAACTTTAAATGTAACAACTTCAATTGAATGGTTCAAGCCCTACC
CCCTAGTG
PE_12
ATTATTAGTCAAGAAAGGGGGAAAAAAGAACCTTTTGGACCTTAGGAATAATTTA
TGCTATAATAGCTATCGGATTATTAGGATTCGTAGTTTGAGCTCACCATATATTTA
CAGTAGATAGATGTAGATACCCGAGCTTACTTCACTTCAGCAACTATAATTATTG
CGGTTCCAACTGGGATTAAAGTCTTTAGATGACTAGCCACCTTTCATGGAACTCA
AATTTTAAATAGACCAGTAACCCTGTGAGCTTTAGGTTTTGTATTTTTATTTACAG
TAGGAGGATTAACAGGGGTAATTTTAGCTAATTCTTCAATTGATATTATTCTCCAC

GATACTTACTATGTAGTAGCTCATTTTCACTATGTTCTTTCTATAGGAGCTGTATT
TGCAATTATAGCCGGAATTGTCCAATGATTCCCATTATTTACCGGACTAACCTTA
AATAATTTTTTTTTAAAAACCCAATTTATTACTATATTTTTAGGTGTAAATTTAACAT
TTTTTCCTCAACACTTTTTAGGGCTAAGAGGAATGCCTCGGCGATATTCGGATTA
TCCAGATGCTTTCACTATCTGAAATGCCATTTCATCTATTGGTTCCATTATTTCTC
TCACAAGAATTATCTTTTTTTTTATTTATCTTATGAGAAAGGCTTTCTTCCCAACGT
AAATCTTTGAGAACTTTAAATGTAACAACTTCAATTGAATGGTTCAAGCCCTACC
CCCTAGTG
PE_13
ATTATTAGACAAGAAAGGGGGAAAAAAGAACCTTTTGGACCTTAGGAATAATTTA
TGCTATAATAGCTATCGGATTATTAGGATTCGTAGTTTGAGCTCACCATATATTTA
CAGTAGATAGATGTAGATACCCGAGCTTACTTCACTTCAGCAACTATAATTATTG
CGGTTCCAACTGGGATTAAAGTCTTTAGATGACTAGCCACCTTTCATGGAACTCA
AATTTTAAATAGACCAGTAACCCTGTGAGCTTTAGGTTTTGTATTTTTATTTACAG
TAGGAGGATTAACAGGGGTAATTTTAGCTAATTCTTCAATTGATATTATTCTCCAC
GATACTTACTATGTAGTAGCTCATTTTCACTATGTTCTTTCTATAGGAGCTGTATT
TGCAATTATAGCCGGAATTGTCCAATGATTCCCATTATTTACCGGACTAACCTTA
AATAATTTTTTTTTAAAAACCCAATTTATTACTATATTTTTAGGTGTAAATTTAACAT
TTTTTCCTCAACACTTTTTAGGGCTAAGAGGAATGCCTCGGCGATATTCGGATTA
TCCAGATGCTTTCACTATCTGAAATGCCATTTCATCTATTGGTTCCATTATTTCTC
TCACAAGAATTATCTTTTTTTTTATTTATCTTATGAGAAAGGCTTTCTTCCCAACGT
AAATCTTTGAGAACTTTAAATGTAACAACTTCAATTGAATGGTTCAAGCCCTACC
CCCTAGTG
PE_15
ATTATTAGACAAGAAAGGGGGAAAAAAGAACCTTTTGGACCTTAGGAATAATTTA
TGCTATAATACCTATCGGATTATTAGGATTCGTAGTTTGAGCTCACCATATATTTA
CAGTAGATAGATGTAGATACCCGAGCTTACTTCACTTCAGCAACTATAATTATTG
CGGTTCCAACTGGGATTAAAGTCTTTAGATGACTAGCCACCTTTCATGGAACTCA
AATTTTAAATAGACCAGTAACCCTGTGAGCTTTAGGTTTTGTATTTTTATTTACAG
TAGGAGGATTAACAGGGGTAATTTTAGCTAATTCTTCAATTGATATTATTCTCCAC
GATACTTACTATGTAGTAGCTCATTTTCACTATGTTCTTTCTATAGGAGCTGTATT
TGCAATTATAGCCGGAATTGTCCAATGATTCCCATTATTTACCGGACTAACCTTA
AATAATTTTTTTTTAAAAACCCAATTTATTACTATATTTTTAGGTGTAAATTTAACAT
TTTTTCCTCAACACTTTTTAGGGCTAAGAGGAATGCCTCGGCGATATTCGGATTA
TCCAGATGCTTTCACTATCTGAAATGCCATTTCATCCATTGGTTCCATTATTTCTC
TCACAAGAATTATCTTTTTTTTTATTTATCTTATGAGAAAGGCTTTCTTCCCAACGT
AAATCTGTGAGAACTTTAAATGTAACAACTTCAATTGAATGGTTCAAGCCCTACC
CCCTAGTG
PE_16
ATTATTAGACAAGAAAGGGGGAAAAAAGAAGCTTTTGGACCTTAGGAATAATTTA
TGCTATAATAGCTATCGGATTATTAGGATTCGTAGTTTGAGCTCACCATATATTTA
CAGTAGATAGATGTAGATACCCGAGCTTACTTCACTTCAGCAACTATAATTATTG
CGGTTCCAACTGGGATTAAAGTCTTTAGATGACTAGCCACCTTTCATGGAACTCA
AATTTTAAATAGACCAGTCACCCTGTGAGCTTTAGGTTTTGTATTTTTATTTACAG
TAGGAGGATTAACAGGGGTAATTTTAGCTAATTCTTCAATTGATATTATTCTCCAC
GATACTTACTATGTAGTAGCTCATTTTCACTATGTTCTTTCTATAGGAGCTGTATT
TGCAATTATAGCCGGAATTGTCCAATGATTCCCATTATTTACCGGACTAACCTTA
AATAATTTTTTTTTAAAAACCCAATTTATTACTATATTTTTAGGTGTAAATTTAACAT
TTTTTCCTCAACACTTTTTAGGGCTAAGAGGAATGCCTCGGCGATATTCGGATTA
TCCAGATGCTTTCACTATCTGAAATGCCATTTCATCTATTGGTTCCATTATTTCTC

TCACAAGAATTATCTTTTTTTTATTTATCTTATGAGAAAGGCTTTCTTCCCAACGT
AAATCTTTGAGAACTTTAAATGTAACAACTTCAATTGAATGGTTCAAGCCCTACC
CCCTAGTG
PE_19
ATTATTAGACAAGAAAGGGGGAAAAAAGAAGCTTTTGGACCTTAGGAATAATTTA
TGCTATAATAGCTATCGGATTATTAGGATTCGTAGTTTGAGCTCACCATATATTTA
CAGTAGATAGATGTAGATACCCGAGCTTACTTCACTTCAGCAACTATAATTATTG
CGGTTCCAACTGGGATTAAAGTCTTTAGATGACTAGCCACCTTTCATGGAACTCA
AATTTTAAATAGACCAGTCACCCTGTGAGCTTTAGGTTTTGTATTTTTATTTACAG
TAGGAGGATTAACAGGGGTAATTTTAGCTAATTCTTCAATTGATATTATTCTCCAC
GATACTTACTATGTAGTAGCTCATTTTCACTATGTTCTTTCTATAGGAGCTGTATT
TGCAATTATAGCCGGAATTGTCCAATGATTCCCATTATTTACCGGACTAACCTTA
AATAATTTTTTTTTAAAAACCCAATTTATTACTATATTTTTAGGTGTAAATTTAACAT
TTTTTCCTCAACACTTTTTAGGGCTAAGAGGAATGCCTCGGCGATATTCGGATTA
TCCAGATGCTTTCACTATCTGAAATGCCATTTCATCCATTGGTTCCATTATTTCTC
TCACAAGAATTATCTTTTTTTTATTTATCTTATGAGAAAGGCTTTCTTCCCAACGT
AAATCTTTGAGAACTTTAAATGTAACAACTTCAATTGAATGGTTCAAGCCCTACC
CCCTAGTG
PE_2
ATTATTAGACAAGAAAGGGGGAAAAAAGAAGCTTTTGGACCTTAGGAATAATTTA
TGCTATAATAGCTATCGGATTATTAGGATTCGTAGTTTGAGCTCACCATATATTTA
CAGTAGATAGATGTAGATACCCGAGCTTACTTCACTTCAGCAACTATAATTATTG
CGGTTCCAACTGGGATTAAAGTCTTTAGATGACTAGCCACCTTTCATGGAACTCA
AATTTTAAATAGACCAGTCACCCTGTGAGCTTTAGGTTTTGTATTTTTATTTACAG
TAGGAGGATTAACAGGGGTAATTTTAGCTAATTCTTCAATTGATATTATTCTCCAC
GATACTTACTATGTAGTAGCTCATTTTCACTATGTTCTTTCTATAGGAGCTGTATT
TGCAATTATAGCCGGAATTGTCCAATGATTCCCATTATTTACCGGACTAACCTTA
AATAATTTTTTTTTAAAAACCCAATTTATTACTATATTTTTAGGTGTAAATTTAACAT
TTTTTCCTCAACACTTTTTAGGGCTAAGAGGAATGCCTCGGCGATATTCGGATTA
TCCAGATGCTTTCACTATCTGAAATGCCATTTCATCTATTGGTTCCATTATTTCTC
TCACAAGAATTATCTTTTTTTTATTTATCTTATGAGAAAGGCTTTCTTCCCAACGT
AAATCTTTGAGAACTTTAAATGTAACAACTTCAATTGAATGGTTCAAGCCCTACC
CCCTAGTG
PE_23
ATTATTAGACAAGAAAGGGGGAAAAAAGAAGCTTTTGGACCTTAGGAATAATTTA
TGCTATAATAGCTATCGGATTATTAGGATTCGTAGTTTGAGCTCACCATATATTTA
CAGTAGATAGATGTAGATACCCGAGCTTACTTCACTTCAGCAACTATAATTATTG
CGGTTCCAACTGGGATTAAAGTCTTTAGATGACTAGCCACCTTTCATGGAACTCA
AATTTTAAATAGACCAGTAACCCTGTGAGCTTTAGGTTTTGTATTTTTATTTACAG
TAGGAGGATTAACAGGGGTAATTTTAGCTAATTCTTCAATTGATATTATTCTCCAC
GATACTTACTATGTAGTAGCTCATTTTCACTATGTTCTTTCTATAGGAGCTGTATT
TGCAATTATAGCCGGAATTGTCCAATGATTCCCATTATTTACCGGACTAACCTTA
AATAATTTTTTTTTAAAAACCCAATTTATTACTATATTTTTAGGTGTAAATTTAACAT
TTTTTCCTCAACACTTTTTAGGGCTAAGAGGAATGCCTCGGCGATATTCGGATTA
TCCAGATGCTTTCACTATCTGAAATGCCATTTCATCTATTGGTTCCATTATTTCTC
TCACAAGAATTATCTTTTTTTTATTTATCTTATGAGAAAGGCTTTCTTCCCAACGT
AAATCTTTGAGAACTTTAAATGTAACAACTTCAATTGAATGGTTCAAGCCCTACC
CCCTAGTG
PE_29
ATTATTAGACAAGAAAGGGGGAAAAAAGAAGCTTTTGGACCTTAGGAATAATTTA

TGCTATAATAGCTATCGGATTATTAGGATTCGTAGTTTGAGCTCACCATATATTTA
CAGTAGATAGATGTAGATACCCGAGCTTACTTCACTTCAGCAACTATAATTATTG
CGGTTCCAACTGGGATTAAAGTCTTTAGATGACTAGCCACCTTTCATGGAACTCA
AATTTTAAATAGACCAGTCACCCTGTGAGCTTTAGGTTTTGTATTTTTATTTACAG
TAGGAGGATTAACAGGGGTAATTTTAGCTAATTCTTCAATTGATATTATTCTCCAC
GATACTTACTATGTAGTAGCTCATTTTCACTATGTTCTTTCTATAGGAGCTGTATT
TGCAATTATAGCCGGAATTGTCCAATGATTCCCATTATTTACCGGACTAACCTTA
AATAATTTTTTTTTAAAAACCCAATTTATTACTATATTTTTAGGTGTAAATTTAACAT
TTTTTCCTCAACACTTTTTAGGGCTAAGAGGAATGCCTCGGCGATATTCGGATTA
TCCAGATGCTTTCACTATCTGAAATGCCATTTCATCTATTGGTTCCATTATTTCTC
TCACAAGAATTATCTTTTTTTTTATTTATCTTATGAGAAAGGCTTTCTTCCCAACGT
AAATCTTTGAGAACTTTAAATGTAACAACTTCAATTGAATGGTTCAAGCCCTACC
CCCTAGTG
PE_6
ATTATTAGACAAGAAAGGGGGAAAAAAGAAGCTTTTGGACCTTAGGAATAATTTA
TGCTATAATAGCTATCGGATTATTAGGATTCGTAGTTTGAGCTCACCATATATTTA
CAGTAGATAGATGTAGATACCCGAGCTTACTTCACTTCAGCAACTATAATTATTG
CGGTTCCAACTGGGATTAAAGTCTTTAGATGACTAGCCACCTTTCATGGAACTCA
AATTTTAAATAGACCAGTAACCCTGTGAGCTTTAGGTTTTGTATTTTTATTTACAG
TAGGAGGATTAACAGGGGTAATTTTAGCTAATTCTTCAATTGATATTATTCTCCAC
GATACTTACTATGTAGTAGCTCATTTTCACTATGTTCTTTCTATAGGAGCTGTATT
TGCAATTATAGCCGGAATTGTCCAATGATTCCCATTATTTACCGGACTAACCTTA
AATAATTTTTTTTTAAAAACCCAATTTATTACTATATTTTTAGGTGTAAATTTAACAT
TTTTTCCTCAACACTTTTTAGGGCTAAGAGGAATGCCTCGGCGATATTCGGATTA
TCCAGATGCTTTCACTATCTGAAATGCCATTTCATCTATTGGTTCCATTATTTCTC
TCACAAGAATTATCTTTTTTTTTATTTATCTTATGAGAAAGGCTTTCTTCCCAACGT
AAATCTTTGAGAACTTTAAATGTAACAACTTCAATTGAATGGTTCAAGCCCTACC
CCCTAGTG
PE_8
ATTATTAGACAAGAAAGGGGGAAAAAAGAAGCTTTTGGACCTTAGGAATAATTTA
TGCTATAATAGCTATCGGATTATTAGGATTCGTAGTTTGAGCTCACCATATATTTA
CAGTAGATAGATGTAGATACCCGAGCTTACTTCACTTCAGCAACTATAATTATTG
CGGTTCCAACTGGGATTAAAGTCTTTAGATGACTAGCCACCTTTCATGGAACTCA
AATTTTAAATAGACCAGTAACCCTGTGAGCTTTAGGTTTTGTATTTTTATTTACAG
TAGGAGGATTAACAGGGGTAATTTTAGCTAATTCTTCAATTGATATTATTCTCCAC
GATACTTACTATGTAGTAGCTCATTTTCACTATGTTCTTTCTATAGGAGCTGTATT
TGCAATTATAGCCGGAATTGTCCAATGATTCCCATTATTTACCGGACTAACCTTA
AATAATTTTTTTTTAAAAACCCAATTTATTACTATATTTTTAGGTGTAAATTTAACAT
TTTTTCCTCAACACTTTTTAGGGCTAAGAGGAATGCCTCGGCGATATTCGGATTA
TCCAGATGCTTTCACTATCTGAAATGCCATTTCATCTATTGGTTCCATTATTTCTC
TCACAAGAATTATCTTTTTTTTTATTTATCTTATGAGAAAGGCTTTCTTCCCAACGT
AAATCTTTGAGAACTTTAAATGTAACAACTTCAATTGAATGGTTCAAGCCCTACC
CCCTAGTG
CL1_1
ATTATTAGACAAGAAAGGGGGAAAAAAGAAGCTTTTGGACCTTAGGAATAATTTA
TGCTATAATAGCTATCGGATTATTAGGATTCGTAGTTTGAGCTCACCATATATTTA
CAGTAGATAGATGTAGATACCCGAGCTTACTTCACTTCAGCAACTATAATTATTG
CGGTTCCAACTGGGATTAAAGTCTTTAGATGACTAGCCACCTTTCATGGAACTCA
AATTTTAAATAGACCAGTAACCCTGTGAGCTTTAGGTTTTGTATTTTTATTTACAG
TAGGAGGATTAACAGGGGTAATTTTAGCTAATTCTTCAATTGATATTATTCTCCAC

GATACTTACTATGTAGTAGCTCATTTTCACTATGTTCTTTCTATAGGAGCTGTATT
TGCAATTATAGCCGGAATTGTCCAATGATTCCCATTATTTACCGGACTAACCTTA
AATAATTTTTTTTTAAAAACCCAATTTATTACTATATTTTTAGGTGTAAATTTAACAT
TTTTTCCTCAACACTTTTTAGGGCTAAGAGGAATGCCTCGGCGATATTCGGATTA
TCCAGATGCTTTCACTATCTGAAATGCCATTTCATCTATTGGTTCCATTATTTCTC
TCACAAGAATTATCTTTTTTTTTATTTATCTTATGAGAAAGGCTTTCTTCCCAACGT
AAATCTTTGAGAACTTTAAATGTAACAACTTCAATTGAATGGTTCAAGCCCTACC
CCCTAGTG
CL1_2
ATTATTAGACAAGAAAGGGGGAAAAAAGAAGCTTTTGGACCTTAGGAATAATTTA
TGCTATAATAGCTATCGGATTATTAGGATTCGTAGTTTGAGCTCACCATATATTTA
CAGTAGATAGATGTAGATACCCGAGCTTACTTCACTTCAGCAACTATAATTATTG
CGGTTCCAACTGGGATTAAAGTCTTTAGATGACTAGCCACCTTTCATGGAACTCA
AATTTTAAATAGACCAGTAACCCTGTGAGCTTTAGGTTTTGTATTTTTATTTACAG
TAGGAGGATTAACAGGGGTAATTTTAGCTAATTCTTCAATTGATATTATTCTCCAC
GATACTTACTATGTAGTAGCTCATTTTCACTATGTTCTTTCTATAGGAGCTGTATT
TGCAATTATAGCCGGAATTGTCCAATGATTCCCATTATTTACCGGACTAACCTTA
AATAATTTTTTTTTAAAAACCCAATTTATTACCATATTTTTAGGTGTAAATTTAACA
TTTTTTCCTCAACACTTTTTAGGGCTAAGAGGAATGCCTCGGCGATATTCGGATT
ATCCAGATGCTTTCACTATCTGAAATGCCATTTCATCTATTGGTTCCATTATTTCT
CTCACAAGAATTATCTTTTTTTTTATTTATCTTATGAGAAAGGCTTTCTTCCCAACG
TAAATCTTTGAGAACTTTAAATGTAACAACTTCAATTGAATGGTTCAAGCCCTACC
CCCTAGTG
CL1_3
ATTATTAGACAAGAAAGGGGGAAAAAAGAAGCTTTTGGACCTTAGGAATAATTTA
TGCTATAATAGCTATCGGATTATTAGGATTCGTAGTTTGAGCTCACCATATATTTA
CAGTAGATAGATGTAGATACCCGAGCTTACTTCACTTCAGCAACTATAATTATTG
CGGTTCCAACTGGGATTAAAGTCTTTAGATGACTAGCCACCTTTCATGGAACTCA
AATTTTAAATAGACCAGTAACCCTGTGAGCTTTAGGTTTTGTATTTTTATTTACAG
TAGGAGGATTAACAGGGGTAATTTTAGCTAATTCTTCAATTGATATTATTCTCCAC
GATACTTACTATGTAGTAGCTCATTTTCACTATGTTCTTTCTATAGGAGCTGTATT
TGCAATTATAGCCGGAATTGTCCAATGATTCCCATTATTTACCGGACTAACCTTA
AATAATTTTTTTTTAAAAACCCAATTTATTACTATATTTTTAGGTGTAAATTTAACAT
TTTTTCCTCAACACTTTTTAGGGCTAAGAGGAATGCCTCGGCGATATTCGGATTA
TCCAGATGCTTTCACTATCTGAAATGCCATTTCATCTATTGGTTCCATTATTTCTC
TCACAAGAATTATCTTTTTTTTTATTTATCTTATGAGAAAGGCTTTCTTCCCAACGT
AAATCTTTGAGAACTTTAAATGTAACAACTTCAATTGAATGGTTCAAGCCCTACC
CCCTAGTG
CL1_4
ATTATTAGACAAGAAAGGGGGAAAAAAGAAGCTTTTGGACCTTAGGAATAATTTA
TGCTATAATAGCTATCGGATTATTAGGATTCGTAGTTTGAGCTCACCATATATTTA
CAGTAGATAGATGTAGATACCCGAGCTTACTTCACTTCAGCAACTATAATTATTG
CGGTTCCAACTGGGATTAAAGTCTTTAGATGACTAGCCACCTTTCATGGAACTCA
AATTTTAAATAGACCAGTAACCCTGTGAGCTTTAGGTTTTGTATTTTTATTTACAG
TAGGAGGATTAACAGGGGTAATTTTAGCTAATTCTTCAATTGATATTATTCTCCAC
GATACTTACTATGTAGTAGCTCATTTTCACTATGTTCTTTCTATAGGAGCTGTATT
TGCAATTATAGCCGGAATTGTCCAATGATTCCCATTATTTACCGGACTAACCTTA
AATAATTTTTTTTTAAAAACCCAATTTATTACTATATTTTTAGGTGTAAATTTAACAT
TTTTTCCTCAACACTTTTTAGGGCTAAGAGGAATGCCTCGGCGATATTCGGATTA
TCCAGATGCTTTCACTATCTGAAATGCCATTTCATCTATTGGTTCCATTATTTCTC

TCACAAGAATTATCTTTTTTTTATTTATCTTATGAGAAAGGCTTTCTTCCCAACGT
AAATCTTTGAGAACTTTAAATGTAACAACTTCAATTGAATGGTTCAAGCCCTACC
CCCTAGTG
CL1_5
ATTATTAGACAAGAAAGGGGGAAAAAAGAAGCTTTTGGACCTTAGGAATAATTTA
TGCTATAATAGCTATCGGATTATTAGGATTCGTAGTTTGAGCTCACCATATATTTA
CAGTAGATAGATGTAGATACCCGAGCTTACTTCACTTCAGCAACTATAATTATTG
CGGTTCCAACTGGGATTAAAGTCTTTAGATGACTAGCCACCTTTCATGGAACTCA
AATTTTAAATAGACCAGTAACCCTGTGAGCTTTAGGTTTTGTATTTTTATTTACAG
TAGGAGGATTAACAGGGGTAATTTTAGCTAATTCTTCAATTGATATTATTCTCCAC
GATACTTACTATGTAGTAGCTCATTTTCACTATGTTCTTTCTATAGGAGCTGTATT
TGCAATTATAGCCGGAATTGTCCAATGATTCCCATTATTTACCGGACTAACCTTA
AATAATTTTTTTTTAAAAACCCAATTTATTACCATATTTTTAGGTGTAAATTTAACA
TTTTTTCCTCAACACTTTTTAGGGCTAAGAGGAATGCCTCGGCGATATTCGGATT
ATCCAGATGCTTTCACTATCTGAAATGCCATTTCATCTATTGGTTCCATTATTTCT
CTCACAAGAATTATCTTTTTTTTATTTATCTTATGAGAAAGGCTTTCTTCCCAACG
TAAATCTTTGAGAACTTTAAATGTAACAACTTCAATTGAATGGTTCAAGCCCTACC
CCCTAGTG
CL1_6
ATTATTAGACAAGAAAGGGGGAAAAAAGAAGCTTTTGGACCTTAGGAATAATTTA
TGCTATAATAGCTATCGGATTATTAGGATTCGTAGTTTGAGCTCACCATATATTTA
CAGTAGATAGATGTAGATACCCGAGCTTACTTCACTTCAGCAACTATAATTATTG
CGGTTCCAACTGGGATTAAAGTCTTTAGATGACTAGCCACCTTTCATGGAACTCA
AATTTTAAATAGACCAGTAACCCTGTGAGCTTTAGGTTTTGTATTTTTATTTACAG
TAGGAGGATTAACAGGGGTAATTTTAGCTAATTCTTCAATTGATATTATTCTCCAC
GATACTTACTATGTAGTAGCTCATTTTCACTATGTTCTTTCTATAGGAGCTGTATT
TGCAATTATAGCCGGAATTGTCCAATGATTCCCATTATTTACCGGACTAACCTTA
AATAATTTTTTTTTAAAAACCCAATTTATTACCATATTTTTAGGTGTAAATTTAACA
TTTTTTCCTCAACACTTTTTAGGGCTAAGAGGAATGCCTCGGCGATATTCGGATT
ATCCAGATGCTTTCACTATCTGAAATGCCATTTCATCTATTGGTTCCATTATTTCT
CTCACAAGAATTATCTTTTTTTTATTTATCTTATGAGAAAGGCTTTCTTCCCAACG
TAAATCTTTGAGAACTTTAAATGTAACAACTTCAATTGAATGGTTCAAGCCCTACC
CCCTAGTG
CL1_7
ATTATTAGACAAGAAAGGGGGAAAAAAGAAGCTTTTGGACCTTAGGAATAATTTA
TGCTATAATAGCTATCGGATTATTAGGATTCGTAGTTTGAGCTCACCATATATTTA
CAGTAGATAGATGTAGATACCCGAGCTTACTTCACTTCAGCAACTATAATTATTG
CGGTTCCAACTGGGATTAAAGTCTTTAGATGACTAGCCACCTTTCATGGAACTCA
AATTTTAAATAGACCAGTAACCCTGTGAGCTTTAGGTTTTGTATTTTTATTTACAG
TAGGAGGATTAACAGGGGTAATTTTAGCTAATTCTTCAATTGATATTATTCTCCAC
GATACTTACTATGTAGTAGCTCATTTTCACTATGTTCTTTCTATAGGAGCTGTATT
TGCAATTATAGCCGGAATTGTCCAATGATTCCCATTATTTACCGGACTAACCTTA
AATAATTTTTTTTTAAAAACCCAATTTATTACTATATTTTTAGGTGTAAATTTAACAT
TTTTTCCTCAACACTTTTTAGGGCTAAGAGGAATGCCTCGGCGATATTCGGATTA
TCCAGATGCTTTCACTATCTGAAATGCCATTTCATCTATTGGTTCCATTATTTCTC
TCACAAGAATTATCTTTTTTTTATTTATCTTATGAGAAAGGCTTTCTTCCCAACGT
AAATCTTTGAGAACTTTAAATGTAACAACTTCAATTGAATGGTTCAAGCCCTACC
CCCTAGTG
CL1_8
ATTATTAGACAAGAAAGGGGGAAAAAAGAAGCTTTTGGACCTTAGGAATAATTTA

TGCTATAATAGCTATCGGATTATTAGGATTCGTAGTTTGAGCTCACCATATATTTA
CAGTAGATAGATGTAGATACCCGAGCTTACTTCACTTCAGCAACTATAATTATTG
CGGTTCCAACTGGGATTAAAGTCTTTAGATGACTAGCCACCTTTCATGGAACTCA
AATTTTAAATAGACCAGTAACCCTGTGAGCTTTAGGTTTTGTATTTTTATTTACAG
TAGGAGGATTAACAGGGGTAATTTTAGCTAATTCTTCAATTGATATTATTCTCCAC
GATACTTACTATGTAGTAGCTCATTTTCACTATGTTCTTTCTATAGGAGCTGTATT
TGCAATTATAGCCGGAATTGTCCAATGATTCCCATTATTTACCGGACTAACCTTA
AATAATTTTTTTTTAAAAACCCAATTTATTACCATATTTTTAGGTGTAAATTTAACA
TTTTTTCCTCAACACTTTTTAGGGCTAAGAGGAATGCCTCGGCGATATTCGGATT
ATCCAGATGCTTTCACTATCTGAAATGCCATTTCATCTATTGGTTCCATTATTTCT
CTCACAAGAATTATCTTTTTTTTTATTTATCTTATGAGAAAGGCTTTCTTCCCAACG
TAAATCTTTGAGAACTTTAAATGTAACAACTTCAATTGAATGGTTCAAGCCCTACC
CCCTAGTG
CL1_9
ATTATTAGACAAGAAAGGGGGAAAAAAGAAGCTTTTGGACCTTAGGAATAATTTA
TGCTATAATAGCTATCGGATTATTAGGATTCGTAGTTTGAGCTCACCATATATTTA
CAGTAGATAGATGTAGATACCCGAGCTTACTTCACTTCAGCAACTATAATTATTG
CGGTTCCAACTGGGATTAAAGTCTTTAGATGACTAGCCACCTTTCATGGAACTCA
AATTTTAAATAGACCAGTAACCCTGTGAGCTTTAGGTTTTGTATTTTTATTTACAG
TAGGAGGATTAACAGGGGTAATTTTAGCTAATTCTTCAATTGATATTATTCTCCAC
GATACTTACTATGTAGTAGCTCATTTTCACTATGTTCTTTCTATAGGAGCTGTATT
TGCAATTATAGCCGGAATTGTCCAATGATTCCCATTATTTACCGGACTAACCTTA
AATAATTTTTTTTTAAAAACCCAATTTATTACTATATTTTTAGGTGTAAATTTAACAT
TTTTTCCTCAACACTTTTTAGGGCTAAGAGGAATGCCTCGGCGATATTCGGATTA
TCCAGATGCTTTCACTATCTGAAATGCCATTTCATCTATTGGTTCCATTATTTCTC
TCACAAGAATTATCTTTTTTTTTATTTATCTTATGAGAAAGGCTTTCTTCCCAACGT
AAATCTTTGAGAACTTTAAATGTAACAACTTCAATTGAATGGTTCAAGCCCTACC
CCCTAGTG
CL2_3
ATTATTAGACAAGAAAGGGGGAAAAAAGAAGCTTTTGGACCTTAGGAATAATTTA
TGCTATAATAGCTATCGGATTATTAGGATTCGTAGTTTGAGCTCACCATATATTTA
CAGTAGATAGATGTAGATACCCGAGCTTACTTCACTTCAGCAACTATAATTATTG
CGGTTCCAACTGGGATTAAAGTCTTTAGATGACTAGCCACCTTTCATGGAACTCA
AATTTTAAATAGACCAGTAACCCTGTGAGCTTTAGGTTTTGTATTTTTATTTACAG
TAGGAGGATTAACAGGGGTAATTTTAGCTAATTCTTCAATTGATATTATTCTCCAC
GATACTTACTATGTAGTAGCTCATTTTCACTATGTTCTTTCTATAGGAGCTGTATT
TGCAATTATAGCCGGAATTGTCCAATGATTCCCATTATTTACCGGACTAACCTTA
AATAATTTTTTTTTAAAAACCCAATTTATTACTATATTTTTAGGTGTAAATTTAACAT
TTTTTCCTCAACACTTTTTAGGGCTAAGAGGAATGCCTCGGCGATATTCGGATTA
TCCAGATGCTTTCACTATCTGAAATGCCATTTCATCTATTGGTTCCATTATTTCTC
TCACAAGAATTATCTTTTTTTTTATTTATCTTATGAGAAAGGCTTTCTTCCCAACGT
AAATCTTTGAGAACTTTAAATGTAACAACTTCAATTGAATGGTTCAAGCCCTACC
CCCTAGTG
CL2_4
ATTATTAGACAAGAAAGGGGGAAAAAAGAAGCTTTTGGACCTTAGGAATAATTTA
TGCTATAATAGCTATCGGATTATTAGGATTCGTAGTTTGAGCTCACCATATATTTA
CAGTAGATAGATGTAGATACCCGAGCTTACTTCACTTCAGCAACTATAATTATTG
CGGTTCCAACTGGGATTAAAGTCTTTAGATGACTAGCCACCTTTCATGGAACTCA
AATTTTAAATAGACCAGTAACCCTGTGAGCTTTAGGTTTTGTATTTTTATTTACAG
TAGGAGGATTAACAGGGGTAATTTTAGCTAATTCTTCAATTGATATTATTCTCCAC

GATACTTACTATGTAGTAGCTCATTTTCACTATGTTCTTTCTATAGGAGCTGTATT
TGCAATTATAGCCGGAATTGTCCAATGATTCCCATTATTTACCGGACTAACCTTA
AATAATTTTTTTTTAAAAACCCAATTTATTACTATATTTTTAGGTGTAAATTTAACAT
TTTTTCCTCAACACTTTTTAGGGCTAAGAGGAATGCCTCGGCGATATTCGGATTA
TCCAGATGCTTTCACTATCTGAAATGCCATTTCATCTATTGGTTCCATTATTTCTC
TCACAAGAATTATCTTTTTTTTTATTTATCTTATGAGAAAGGCTTTCTTCCCAACGT
AAATCTTTGAGAACTTTAAATGTAACAACTTCAATTGAATGGTTCAAGCCCTACC
CCCTAGTG
CL2_5
ATTATTAGACAAGAAAGGGGGAAAAAAGAAGCTTTTGGACCTTAGGAATAATTTA
TGCTATAATAGCTATCGGATTATTAGGATTCGTAGTTTGAGCTCACCATATATTTA
CAGTAGATAGATGTAGATACCCGAGCTTACTTCACTTCAGCAACTATAATTATTG
CGGTTCCAACTGGGATTAAAGTCTTTAGATGACTAGCCACCTTTCATGGAACTCA
AATTTTAAATAGACCAGTAACCCTGTGAGCTTTAGGTTTTGTATTTTTATTTACAG
TAGGAGGATTAACAGGGGTAATTTTAGCTAATTCTTCAATTGATATTATTCTCCAC
GATACTTACTATGTAGTAGCTCATTTTCACTATGTTCTTTCTATAGGAGCTGTATT
TGCAATTATAGCCGGAATTGTCCAATGATTCCCATTATTTACCGGACTAACCTTA
AATAATTTTTTTTTAAAAACCCAATTTATTACTATATTTTTAGGTGTAAATTTAACAT
TTTTTCCTCAACACTTTTTAGGGCTAAGAGGAATGCCTCGGCGATATTCGGATTA
TCCAGATGCTTTCACTATCTGAAATGCCATTTCATCTATTGGTTCCATTATTTCTC
TCACAAGAATTATCTTTTTTTTTATTTATCTTATGAGAAAGGCTTTCTTCCCAACGT
AAATCTTTGAGAACTTTAAATGTAACAACTTCAATTGAATGGTTCAAGCCCTACC
CCCTAGTG
CL2_6
ATTATTAGACAAGAAAGGGGGAAAAAAGAAGCTTTTGGACCTTAGGAATAATTTA
TGCTATAATAGCTATCGGATTATTAGGATTCGTAGTTTGAGCTCACCATATATTTA
CAGTAGATAGATGTAGATACCCGAGCTTACTTCACTTCAGCAACTATAATTATTG
CGGTTCCAACTGGGATTAAAGTCTTTAGATGACTAGCCACCTTTCATGGAACTCA
AATTTTAAATAGACCAGTAACCCTGTGAGCTTTAGGTTTTGTATTTTTATTTACAG
TAGGAGGATTAACAGGGGTAATTTTAGCTAATTCTTCAATTGATATTATTCTCCAC
GATACTTACTATGTAGTAGCTCATTTTCACTATGTTCTTTCTATAGGAGCTGTATT
TGCAATTATAGCCGGAATTGTCCAATGATTCCCATTATTTACCGGACTAACCTTA
AATAATTTTTTTTTAAAAACCCAATTTATTACTATATTTTTAGGTGTAAATTTAACAT
TTTTTCCTCAACACTTTTTAGGGCTAAGAGGAATGCCTCGGCGATATTCGGATTA
TCCAGATGCTTTCACTATCTGAAATGCCATTTCATCTATTGGTTCCATTATTTCTC
TCACAAGAATTATCTTTTTTTTTATTTATCTTATGAGAAAGGCTTTCTTCCCAACGT
AAATCTTTGAGAACTTTAAATGTAACAACTTCAATTGAATGGTTCAAGCCCTACC
CCCTAGTG
CL2_7
ATTATTAGACAAGAAAGGGGGAAAAAAGAAGCTTTTGGACCTTAGGAATAATTTA
TGCTATAATAGCTATCGGATTATTAGGATTCGTAGTTTGAGCTCACCATATATTTA
CAGTAGATAGATGTAGATACCCGAGCTTACTTCACTTCAGCAACTATAATTATTG
CGGTTCCAACTGGGATTAAAGTCTTTAGATGACTAGCCACCTTTCATGGAACTCA
AATTTTAAATAGACCAGTAACCCTGTGAGCTTTAGGTTTTGTATTTTTATTTACAG
TAGGAGGATTAACAGGGGTAATTTTAGCTAATTCTTCAATTGATATTATTCTCCAC
GATACTTACTATGTAGTAGCTCATTTTCACTATGTTCTTTCTATAGGAGCTGTATT
TGCAATTATAGCCGGAATTGTCCAATGATTCCCATTATTTACCGGACTAACCTTA
AATAATTTTTTTTTAAAAACCCAATTTATTACTATATTTTTAGGTGTAAATTTAACAT
TTTTTCCTCAACACTTTTTAGGGCTAAGAGGAATGCCTCGGCGATATTCGGATTA
TCCAGATGCTTTCACTATCTGAAATGCCATTTCATCTATTGGTTCCATTATTTCTC

TCACAAGAATTATCTTTTTTTTATTTATCTTATGAGAAAGGCTTTCTTCCCAACGT
AAATCTTTGAGAACTTTAAATGTAACAACTTCAATTGAATGGTTCAAGCCCTACC
CCCTAGTG
CL2_8
ATTATTAGACAAGAAAGGGGGAAAAAAGAAGCTTTTGGACCTTAGGAATAATTTA
TGCTATAATAGCTATCGGATTATTAGGATTCGTAGTTTGAGCTCACCATATATTTA
CAGTAGATAGATGTAGATACCCGAGCTTACTTCACTTCAGCAACTATAATTATTG
CGGTTCCAACTGGGATTAAAGTCTTTAGATGACTAGCCACCTTTCATGGAACTCA
AATTTTAAATAGACCAGTAACCCTGTGAGCTTTAGGTTTTGTATTTTTATTTACAG
TAGGAGGATTAACAGGGGTAATTTTAGCTAATTCTTCAATTGATATTATTCTCCAC
GATACTTACTATGTAGTAGCTCATTTTCACTATGTTCTTTCTATAGGAGCTGTATT
TGCAATTATAGCCGGAATTGTCCAATGATTCCCATTATTTACCGGACTAACCTTA
AATAATTTTTTTTTAAAAACCCAATTTATTACTATATTTTTAGGTGTAAATTTAACAT
TTTTTCCTCAACACTTTTTAGGGCTAAGAGGAATGCCTCGGCGATATTCGGATTA
TCCAGATGCTTTCACTATCTGAAATGCCATTTCATCTATTGGTTCCATTATTTCTC
TCACAAGAATTATCTTTTTTTTATTTATCTTATGAGAAAGGCTTTCTTCCCAACGT
AAATCTTTGAGAACTTTAAATGTAACAACTTCAATTGAATGGTTCAAGCCCTACC
CCCTAGTG
CL2_1
ATTATTAGACAAGAAAGGGGGAAAAAAGAAGCTTTTGGACCTTAGGAATAATTTA
TGCTATAATAGCTATCGGATTATTAGGATTCGTAGTTTGAGCTCACCATATATTTA
CAGTAGATAGATGTAGATACCCGAGCTTACTTCACTTCAGCAACTATAATTATTG
CGGTTCCAACTGGGATTAAAGTCTTTAGATGACTAGCCACCTTTCATGGAACTCA
AATTTTAAATAGACCAGTAACCCTGTGAGCTTTAGGTTTTGTATTTTTATTTACAG
TAGGAGGATTAACAGGGGTAATTTTAGCTAATTCTTCAATTGATATTATTCTCCAC
GATACTTACTATGTAGTAGCTCATTTTCACTATGTTCTTTCTATAGGAGCTGTATT
TGCAATTATAGCCGGAATTGTCCAATGATTCCCATTATTTACCGGACTAACCTTA
AATAATTTTTTTTTAAAAACCCAATTTATTACTATATTTTTAGGTGTAAATTTAACAT
TTTTTCCTCAACACTTTTTAGGGCTAAGAGGAATGCCTCGGCGATATTCGGATTA
TCCAGATGCTTTCACTATCTGAAATGCCATTTCATCTATTGGTTCCATTATTTCTC
TCACAAGAATTATCTTTTTTTTATTTATCTTATGAGAAAGGCTTTCTTCCCAACGT
AAATCTTTGAGAACTTTAAATGTAACAACTTCAATTGAATGGTTCAAGCCCTACC
CCCTAGTG
CL2_2
ATTATTAGACAAGAAAGGGGGAAAAAAGAAGCTTTTGGACCTTAGGAATAATTTA
TGCTATAATAGCTATCGGATTATTAGGATTCGTAGTTTGAGCTCACCATATATTTA
CAGTAGATAGATGTAGATACCCGAGCTTACTTCACTTCAGCAACTATAATTATTG
CGGTTCCAACTGGGATTAAAGTCTTTAGATGACTAGCCACCTTTCATGGAACTCA
AATTTTAAATAGACCAGTAACCCTGTGAGCTTTAGGTTTTGTATTTTTATTTACAG
TAGGAGGATTAACAGGGGTAATTTTAGCTAATTCTTCAATTGATATTATTCTCCAC
GATACTTACTATGTAGTAGCTCATTTTCACTATGTTCTTTCTATAGGAGCTGTATT
TGCAATTATAGCCGGAATTGTCCAATGATTCCCATTATTTACCGGACTAACCTTA
AATAATTTTTTTTTAAAAACCCAATTTATTACTATATTTTTAGGTGTAAATTTAACAT
TTTTTCCTCAACACTTTTTAGGGCTAAGAGGAATGCCTCGGCGATATTCGGATTA
TCCAGATGCTTTCACTATCTGAAATGCCATTTCATCTATTGGTTCCATTATTTCTC
TCACAAGAATTATCTTTTTTTTATTTATCTTATGAGAAAGGCTTTCTTCCCAACGT
AAATCTTTGAGAACTTTAAATGTAACAACTTCAATTGAATGGTTCAAGCCCTACC
CCCTAGTG
EQ1_3
ATTATTAGACAAGAAAGGGGGAAAAAAGAAGCTTTTGGACCTTAGGAATAATTTA

TGCTATAATAGCTATCGGATTATTAGGATTCGTAGTTTGAGCTCACCATATATTTA
CAGTAGATAGATGTAGATACCCGAGCTTACTTCACTTCAGCAACTATAATTATTG
CGGTTCCAACTGGGATTAAAGTCTTTAGATGACTAGCCACCTTTCATGGAACTCA
AATTTTAAATAGACCAGTAACCCTGTGAGCTTTAGGTTTTGTATTTTTATTTACAG
TAGGAGGATTAACAGGGGTAATTTTAGCTAATTCTTCAATTGATATTATTCTCCAC
GATACTTACTATGTAGTAGCTCATTTTCACTATGTTCTTTCTATAGGAGCTGTATT
TGCAATTATAGCCGGAATTGTCCAATGATTCCCATTATTTACCGGACTAACCTTA
AATAATTTTTTTTTAAAAACCCAATTTATTACTATATTTTTAGGTGTAAATTTAACAT
TTTTTCCTCAACACTTTTTAGGGCTAAGAGGAATGCCTCGGCGATATTCGGATTA
TCCAGATGCTTTCACTATCTGAAATGCCATTTCATCTATTGGTTCCATTATTTCTC
TCACAAGAATTATCTTTTTTTTATTTATCTTATGAGAAAGGCTTTCTTCCCAACGT
AAATCTTTGAGAACTTTAAATGTAACAACTTCAATTGAATGGTTCAAGCCCTACC
CCCTAGTG
EQ1_6
ATTATTAGACAAGAAAGGGGGAAAAAAGAAGCTTTTGGACCTTAGGAATAATTTA
TGCTATAATAGCTATCGGATTATTAGGATTCGTAGTTTGAGCTCACCATATATTTA
CAGTAGATAGATGTAGATACCCGAGCTTACTTCACTTCAGCAACTATAATTATTG
CGGTTCCAACTGGGATTAAAGTCTTTAGATGACTAGCCACCTTTCATGGAACTCA
AATTTTAAATAGACCAGTAACCCTGTGAGCTTTAGGTTTTGTATTTTTATTTACAG
TAGGAGGATTAACAGGGGTAATTTTAGCTAATTCTTCAATTGATATTATTCTCCAC
GATACTTACTATGTAGTAGCTCATTTTCACTATGTTCTTTCTATAGGAGCTGTATT
TGCAATTATAGCCGGAATTGTCCAATGATTCCCATTATTTACCGGACTAACCTTA
AATAATTTTTTTTTAAAAACCCAATTTATTACTATATTTTTAGGTGTAAATTTAACAT
TTTTTCCTCAACACTTTTTAGGGCTAAGAGGAATGCCTCGGCGATATTCGGATTA
TCCAGATGCTTTCACTATCTGAAATGCCATTTCATCTATTGGTTCCATTATTTCTC
TCACAAGAATTATCTTTTTTTTATTTATCTTATGAGAAAGGCTTTCTTCCCAACGT
AAATCTTTGAGAACTTTAAATGTAACAACTTCAATTGAATGGTTCAAGCCCTACC
CCCTAGTG
EQ1_7
ATTATTAGACAAGAAAGGGGGAAAAAAGAAGCTTTTGGACCTTAGGAATAATTTA
TGCTATAATAGCTATCGGATTATTAGGATTCGTAGTTTGAGCTCACCATATATTTA
CAGTAGATAGATGTAGATACCCGAGCTTACTTCACTTCAGCAACTATAATTATTG
CGGTTCCAACTGGGATTAAAGTCTTTAGATGACTAGCCACCTTTCATGGAACTCA
AATTTTAAATAGACCAGTAACCCTGTGAGCTTTAGGTTTTGTATTTTTATTTACAG
TAGGAGGATTAACAGGGGTAATTTTAGCTAATTCTTCAATTGATATTATTCTCCAC
GATACTTACTATGTAGTAGCTCATTTTCACTATGTTCTTTCTATAGGAGCTGTATT
TGCAATTATAGCCGGAATTGTCCAATGATTCCCATTATTTACCGGACTAACCTTA
AATAATTTTTTTTTAAAAACCCAATTTATTACTATATTTTTAGGTGTAAATTTAACAT
TTTTTCCTCAACACTTTTTAGGGCTAAGAGGAATGCCTCGGCGATATTCGGATTA
TCCAGATGCTTTCACTATCTGAAATGCCATTTCATCTATTGGTTCCATTATTTCTC
TCACAAGAATTATCTTTTTTTTATTTATCTTATGAGAAAGGCTTTCTTCCCAACGT
AAATCTTTGAGAACTTTAAATGTAACAACTTCAATTGAATGGTTCAAGCCCTACC
CCCTAGTG
EQ1_8
ATTATTAGACAAGAAAGGGGGAAAAAAGAAGCTTTTGGACCTTAGGAATAATTTA
TGCTATAATAGCTATCGGATTATTAGGATTCGTAGTTTGAGCTCACCATATATTTA
CAGTAGATAGATGTAGATACCCGAGCTTACTTCACTTCAGCAACTATAATTATTG
CGGTTCCAACTGGGATTAAAGTCTTTAGATGACTAGCCACCTTTCATGGAACTCA
AATTTTAAATAGACCAGTAACCCTGTGAGCTTTAGGTTTTGTATTTTTATTTACAG
TAGGAGGATTAACAGGGGTAATTTTAGCTAATTCTTCAATTGATATTATTCTCCAC

GATACTTACTATGTAGTAGCTCATTTTCACTATGTTCTTTCTATAGGAGCTGTATT
TGCAATTATAGCCGGAATTGTCCAATGATTCCCATTATTTACCGGACTAACCTTA
AATAATTTTTTTTTAAAAACCCAATTTATTACTATATTTTTAGGTGTAAATTTAACAT
TTTTTCCTCAACACTTTTTAGGGCTAAGAGGAATGCCTCGGCGATATTCGGATTA
TCCAGATGCTTTCACTATCTGAAATGCCATTTCATCTATTGGTTCCATTATTTCTC
TCACAAGAATTATCTTTTTTTTATTTATCTTATGAGAAAGGCTTTCTTCCCAACGT
AAATCTTTGAGAACTTTAAATGTAACAACTTCAATTGAATGGTTCAAGCCCTACC
CCCTAGTG
EQU_5
ATTATTAGACAAGAAAGGGGGAAAAAAGAAGCTTTTGGACCTTAGGAATAATTTA
TGCTATAATAGCTATCGGATTATTAGGATTCGTAGTTTGAGCTCACCATATATTTA
CAGTAGATAGATGTAGATACCCGAGCTTACTTCACTTCAGCAACTATAATTATTG
CGGTTCCAACTGGGATTAAAGTCTTTAGATGACTAGCCACCTTTCATGGAACTCA
AATTTTAAATAGACCAGTAACCCTGTGAGCTTTAGGTTTTGTATTTTTATTTACAG
TAGGAGGATTAACAGGGGTAATTTTAGCTAATTCTTCAATTGATATTATTCTCCAC
GATACTTACTATGTAGTAGCTCATTTTCACTATGTTCTTTCTATAGGAGCTGTATT
TGCAATTATAGCCGGAATTGTCCAATGATTCCCATTATTTACCGGACTAACCTTA
AATAATTTTTTTTTAAAAACCCAATTTATTACTATATTTTTAGGTGTAAATTTAACAT
TTTTTCCTCAACACTTTTTAGGGCTAAGAGGAATGCCTCGGCGATATTCGGATTA
TCCAGATGCTTTCACTATCTGAAATGCCATTTCATCTATTGGTTCCATTATTTCTC
TCACAAGAATTATCTTTTTTTTATTTATCTTATGAGAAAGGCTTTCTTCCCAACGT
AAATCTTTGAGAACTTTAAATGTAACAACTTCAATTGAATGGTTCAAGCCCTACC
CCCTAGTG
;

endblock;

datasetS6

#NEXUS

begin data;
dimensions ntax=187 nchar=732;
format datatype=dna gap=- interleave=no;
matrix

AFR10
ATTATTAGACAAGAAAGGGGGAAAAAAGAAGCTTTTGGACCTTAGGAATAATTTA
TGCCATGATAGCTATCGGATTATTAGGATTCGTAGTTTGAGCTCACCATATATTT
ACAGTAGATAGATGTAGATACCCGAGCTTACTTCACTTCAGCAACTATAATTATT
GCGGTTCCAACTGGGATTAAAGTCTTTAGATGACTAGCCACCTTTCATGGAACT
CAAATTTTAAATAGACCAGTAACCCTGTGAGCTTTAGGTTTTGTATTTTTATTTAC
AGTAGGAGGATTAACAGGGGTAATTTTAGCTAATTCTTCAATTGATATTATTCTTC
ACGATACTTACTATGTAGTAGCTCATTTTCACTATGTTCTTTCTATAGGAGCTGTA
TTTGCAATTATAGCCGGAATTGTCCAATGATTCCCATTATTTACCGGACTAACCT
TAAATAATTTTTTTTTAAAAACCCAATTTATTACTATATTTTTAGGTGTAAATTTAAC
ATTTTTTCCCCAACACTTTTTAGGGCTAAGAGGAATGCCTCGGCGATATTCGGAT
TATCCAGATGCTTTCACTATCTGAAATGCCATTTCATCTATTGGTTCCATTATTTC
TCTCACAAGAATTATCTTTTTTTTATTTATCTTATGAGAAAGGCTTTCTTCCCAAC
GTAAATCTTTGAGAACTTTAAATGTAACAACTTCAATTGAATGGTTCAAGCCCTAC
CCCCTAGTG
AFR11
ATTATTAGACAAGAAAGGGGGAAAAAAGAAGCTTTTGGACCTTAGGAATAATTTA
TGCCATGATAGCTATCGGATTATTAGGATTCGTAGTTTGAGCTCACCATATATTT
ACAGTAGATAGATGTAGATACCCGAGCTTACTTCACTTCAGCAACTATAATTATT
GCGGTTCCAACTGGGATTAAAGTCTTTAGATGACTAGCCACCTTTCATGGAACT
CAAATTTTAAATAGACCAGTAACCCTGTGAGCTTTAGGTTTTGTATTTTTATTTAC
AGTAGGAGGATTAACAGGGGTAATTTTAGCTAATTCTTCAATTGATATTATTCTTC
ACGATACTTACTATGTAGTAGCTCATTTTCACTATGTTCTTTCTATAGGAGCTGTA
TTTGCAATTATAGCCGGAATTGTCCAATGATTCCCATTATTTACCGGACTAACCT
TAAATAATTTTTTTTTAAAAACCCAATTTATTACTATATTTTTAGGTGTAAATTTAAC
ATTTTTTCCCCAACACTTTTTAGGGCTAAGAGGAATGCCTCGGCGATATTCGGAT
TATCCAGATGCTTTCACTATCTGAAATGCCATTTCATCTATTGGTTCCATTATTTC
TCTCACAAGAATTATCTTTTTTTTATTTATCTTATGAGAAAGGCTTTCTTCCCAAC
GTAAATCTTTGAGAACTTTAAATGTAACAACTTCAATTGAATGGTTCAAGCCCTAC
CCCCTAGTG
AFR12
ATTATTAGACAAGAAAGGGGGAAAAAAGAAGCTTTTGGACCTTAGGAATAATTTA
TGCCATGATAGCTATCGGATTATTAGGATTCGTAGTTTGAGCTCACCATATATTT
ACAGTAGATAGATGTAGATACCCGAGCTTACTTCACTTCAGCAACTATAATTATT
GCGGTTCCAACTGGGATTAAAGTCTTTAGATGACTAGCCACCTTTCATGGAACT
CAAATTTTAAATAGACCAGTAACCCTGTGAGCTTTAGGTTTTGTATTTTTATTTAC
AGTAGGAGGATTAACAGGGGTAATTTTAGCTAATTCTTCAATTGATATTATTCTTC
ACGATACTTACTATGTAGTAGCTCATTTTCACTATGTTCTTTCTATAGGAGCTGTA
TTTGCAATTATAGCCGGAATTGTCCAATGATTCCCATTATTTACCGGACTAACCT
TAAATAATTTTTTTTTAAAAACCCAATTTATTACTATATTTTTAGGTGTAAATTTAAC
ATTTTTTCCCCAACACTTTTTAGGGCTAAGAGGAATGCCTCGGCGATATTCGGAT
TATCCAGATGCTTTCACTATCTGAAATGCCATTTCATCTATTGGTTCCATTATTTC

TCTCACAAGAATTATCTTTTTTTTATTTATCTTATGAGAAAGGCTTTCTTCCCAAC
GTAAATCTTTGAGAACTTTAAATGTAACAACTTCAATTGAATGGTTCAAGCCCTAC
CCCCTAGTG
AFR18
ATTATTAGACAAGAAAGGGGGAAAAAAGAAGCTTTTGGACCTTAGGAATAATTTA
TGCCATGATAGCTATCGGATTATTAGGATTCGTAGTTTGAGCTCACCATATATTT
ACAGTAGATAGATGTAGATACCCGAGCTTACTTCACTTCAGCAACTATAATTATT
GCGGTTCCAACTGGGATTAAAGTCTTTAGATGACTAGCCACCTTTCATGGAACT
CAAATTTTAAATAGACCAGTAACCCTGTGAGCTTTAGGTTTTGTATTTTTATTTAC
AGTAGGAGGATTAACAGGGGTAATTTTAGCTAATTCTTCAATTGATATTATTCTTC
ACGATACTTACTATGTAGTAGCTCATTTTCACTATGTTCTTTCTATAGGAGCTGTA
TTTGCAATTATAGCCGGAATTGTCCAATGATTCCCATTATTTACCGGACTAACCT
TAAATAATTTTTTTTTAAAAACCCAATTTATTACTATATTTTTAGGTGTAAATTTAAC
ATTTTTTCCCCAACACTTTTTAGGGCTAAGAGGAATGCCTCGGCGATATTCGGAT
TATCCAGATGCTTTCACTATCTGAAATGCCATTTCATCTATTGGTTCCATTATTTC
TCTCACAAGAATTATCTTTTTTTTATTTATCTTATGAGAAAGGCTTTCTTCCCAAC
GTAAATCTTTGAGAACTTTAAATGTAACAACTTCAATTGAATGGTTCAAGCCCTAC
CCCCTAGTG
afr19
ATTATTAGACAAGAAAGGGGGAAAAAAGAAGCTTTTGGACCTTAGGAATAATTTA
TGCCATGATAGCTATCGGATTATTAGGATTCGTAGTTTGAGCTCACCATATATTT
ACAGTAGATAGATGTAGATACCCGAGCTTACTTCACTTCAGCAACTATAATTATT
GCGGTTCCAACTGGGATTAAAGTCTTTAGATGACTAGCCACCTTTCATGGAACT
CAAATTTTAAATAGACCAGTAACCCTGTGAGCTTTAGGTTTTGTATTTTTATTTAC
AGTAGGAGGATTAACAGGGGTAATTTTAGCTAATTCTTCAATTGATATTATTCTTC
ACGATACTTACTATGTAGTAGCTCATTTTCACTATGTTCTTTCTATAGGAGCTGTA
TTTGCAATTATAGCCGGAATTGTCCAATGATTCCCATTATTTACCGGACTAACCT
TAAATAATTTTTTTTTAAAAACCCAATTTATTACTATATTTTTAGGTGTAAATTTAAC
ATTTTTTCCCCAACACTTTTTAGGGCTAAGAGGAATGCCTCGGCGATATTCGGAT
TATCCAGATGCTTTCACTATCTGAAATGCCATTTCATCTATTGGTTCCATTATTTC
TCTCACAAGAATTATCTTTTTTTTATTTATCTTATGAGAAAGGCTTTCTTCCCAAC
GTAAATCTTTGAGAACTTTAAATGTAACAACTTCAATTGAATGGTTCAAGCCCTAC
CCCCTAGTG
AFR2
ATTATTAGACAAGAAAGGGGGAAAAAAGAAGCTTTTGGACCTTAGGAATAATTTA
TGCCATGATAGCTATCGGATTATTAGGATTCGTAGTTTGAGCTCACCATATATTT
ACAGTAGATAGATGTAGATACCCGAGCTTACTTCACTTCAGCAACTATAATTATT
GCGGTTCCAACTGGGATTAAAGTCTTTAGATGACTAGCCACCTTTCATGGAACT
CAAATTTTAAATAGACCAGTAACCCTGTGAGCTTTAGGTTTTGTATTTTTATTTAC
AGTAGGAGGATTAACAGGGGTAATTTTAGCTAATTCTTCAATTGATATTATTCTTC
ACGATACTTACTATGTAGTAGCTCATTTTCACTATGTTCTTTCTATAGGAGCTGTA
TTTGCAATTATAGCCGGAATTGTCCAATGATTCCCATTATTTACCGGACTAACCT
TAAATAATTTTTTTTTAAAAACCCAATTTATTACTATATTTTTAGGTGTAAATTTAAC
ATTTTTTCCCCAACACTTTTTAGGGCTAAGAGGAATGCCTCGGCGATATTCGGAT
TATCCAGATGCTTTCACTATCTGAAATGCCATTTCATCTATTGGTTCCATTATTTC
TCTCACAAGAATTATCTTTTTTTTATTTATCTTATGAGAAAGGCTTTCTTCCCAAC
GTAAATCTTTGAGAACTTTAAATGTAACAACTTCAATTGAATGGTTCAAGCCCTAC
CCCCTAGTG
AFR3
ATTATTAGACAAGAAAGGGGGAAAAAAGAAGCTTTTGGACCTTAGGAATAATTTA

TGCCATGATAGCTATCGGATTATTAGGATTCGTAGTTTGAGCTCACCATATATTT
ACAGTAGATAGATGTAGATACCCGAGCTTACTTCACTTCAGCAACTATAATTATT
GCGGTTCCAACTGGGATTAAAGTCTTTAGATGACTAGCCACCTTTCATGGAACT
CAAATTTTAAATAGACCAGTAACCCTGTGAGCTTTAGGTTTTGTATTTTTATTTAC
AGTAGGAGGATTAACAGGGGTAATTTTAGCTAATTCTTCAATTGATATTATTCTTC
ACGATACTTACTATGTAGTAGCTCATTTTCACTATGTTCTTTCTATAGGAGCTGTA
TTTGCAATTATAGCCGGAATTGTCCAATGATTCCCATTATTTACCGGACTAACCT
TAAATAATTTTTTTTTAAAAACCCAATTTATTACTATATTTTTAGGTGTAAATTTAAC
ATTTTTTCCCCAACACTTTTTAGGGCTAAGAGGAATGCCTCGGCGATATTCGGAT
TATCCAGATGCTTTCACTATCTGAAATGCCATTTCATCTATTGGTTCCATTATTTC
TCTCACAAGAATTATCTTTTTTTTATTTATCTTATGAGAAAGGCTTTCTTCCCAAC
GTAAATCTTTGAGAACTTTAAATGTAACAACTTCAATTGAATGGTTCAAGCCCTAC
CCCCTAGTG
AFR5
ATTATTAGACAAGAAAGGGGGAAAAAAGAAGCTTTTGGACCTTAGGAATAATTTA
TGCCATGATAGCTATCGGATTATTAGGATTCGTAGTTTGAGCTCACCATATATTT
ACAGTAGATAGATGTAGATACCCGAGCTTACTTCACTTCAGCAACTATAATTATT
GCGGTTCCAACTGGGATTAAAGTCTTTAGATGACTAGCCACCTTTCATGGAACT
CAAATTTTAAATAGACCAGTAACCCTGTGAGCTTTAGGTTTTGTATTTTTATTTAC
AGTAGGAGGATTAACAGGGGTAATTTTAGCTAATTCTTCAATTGATATTATTCTTC
ACGATACTTACTATGTAGTAGCTCATTTTCACTATGTTCTTTCTATAGGAGCTGTA
TTTGCAATTATAGCCGGAATTGTCCAATGATTCCCATTATTTACCGGACTAACCT
TAAATAATTTTTTTTTAAAAACCCAATTTATTACTATATTTTTAGGTGTAAATTTAAC
ATTTTTTCCCCAACACTTTTTAGGGCTAAGAGGAATGCCTCGGCGATATTCGGAT
TATCCAGATGCTTTCACTATCTGAAATGCCATTTCATCTATTGGTTCCATTATTTC
TCTCACAAGAATTATCTTTTTTTTATTTATCTTATGAGAAAGGCTTTCTTCCCAAC
GTAAATCTTTGAGAACTTTAAATGTAACAACTTCAATTGAATGGTTCAAGCCCTAC
CCCCTAGTG
afr9
ATTATTAGACAAGAAAGGGGGAAAAAAGAAGCTTTTGGACCTTAGGAATAATTTA
TGCCATGATAGCTATCGGATTATTAGGATTCGTAGTTTGAGCTCACCATATATTT
ACAGTAGATAGATGTAGATACCCGAGCTTACTTCACTTCAGCAACTATAATTATC
GCGGTTCCAACTGGGATTAAAGTCTTTAGGTGACTAGCCACCTTTCATGGAACT
CAAATTTTAAATAGACCAGTAACCCTGTGAGCTTTAGGTTTTGTATTTTTATTTAC
AGTAGGAGGATTAACAGGGGTAATTTTAGCTAATTCTTCAATTGATATTATTCTTC
ACGATACTTACTATGTAGTAGCTCATTTTCACTATGTTCTTTCTATAGGAGCTGTA
TTTGCAATTATAGCCGGAATTGTCCAATGATTCCCATTATTTACTGGACTAACCTT
AAATAATTTTTTTTTAAAAACCCAATTTATTACTATATTTTTAGGTGTAAATTTAACA
TTTTTTCCTCAACACTTTTTAGGGCTAAGAGGAATGCCTCGGCGATATTCGGATT
ATCCAGATGCTTTCACTATCTGAAATGCCATTTCATCTATTGGTTCCATTATTTCT
CTCACAAGAATTATCTTTTTTTTATTTATCTTATGAGAAAGGCTTTCTTCCCAACG
TAAATCTTTGAGAACTTTAAATGTAACAACTTCAATTGAATGGTTCAAGCCCTACC
CCCTAGTG
AQU10
ATTATTAGACAAGAAAGGGGGAAAAAAGAAGCTTTTGGACCTTAGGAATAATTTA
TGCCATGATAGCTATCGGATTATTAGGATTCGTAGTTTGAGCTCACCATATATTT
ACAGTAGATAGATGTAGATACCCGAGCTTACTTCACTTCAGCAACTATAATTATT
GCGGTTCCAACTGGGATTAAAGTCTTTAGATGACTAGCCACCTTTCATGGAACT
CAAATTTTAAATAGACCAGTAACCCTGTGAGCTTTAGGTTTTGTATTTTTATTTAC
AGTAGGAGGATTAACAGGGGTAATTTTAGCTAATTCTTCAATTGATATTATTCTTC

ACGATACTTACTATGTAGTAGCTCATTTTCACTATGTTCTTTCTATAGGAGCTGTA
TTTGCAATTATAGCCGGAATTGTCCAATGATTCCCATTATTTACCGGACTAACCT
TAAATAATTTTTTTTTAAAAACCCAATTTATTACTATATTTTTAGGTGTAAATTTAAC
ATTTTTTCCCCAACACTTTTTAGGGCTAAGAGGAATGCCTCGGCGATATTCGGAT
TATCCAGATGCTTTCACTATCTGAAATGCCATTTCATCTATTGGTTCCATTATTTC
TCTCACAAGAATTATCTTTTTTTTATTTATCTTATGAGAAAGGCTTTCTTCCCAAC
GTAAATCTTTGAGAACTTTAAATGTAACAACTTCAATTGAATGGTTCAAGCCCTAC
CCCCTAGTG
AQU12
ATTATTAGACAAGAAAGGGGGAAAAAAGAAGCTTTTGGACCTTAGGAATAATTTA
TGCCATGATAGCTATCGGATTATTAGGATTCGTAGTCTGAGCTCACCATATATTT
ACAGTAGATAGATGTAGATACCCGAGCTTACTTCACTTCAGCAACTATAATTATT
GCGGTTCCAACTGGGATTAAAGTCTTTAGATGACTAGCCACCTTTCATGGAACT
CAAATTTTAAATAGACCAGTAACCCTGTGAGCTTTAGGTTTTGTATTTTTATTTAC
AGTAGGAGGATTAACAGGGGTAATTTTAGCTAATTCTTCAATTGATATTATTCTTC
ACGATACTTACTATGTAGTAGCTCATTTTCACTATGTTCTTTCTATAGGAGCTGTA
TTTGCAATTATAGCCGGAATTGTCCAATGATTCCCATTATTTACCGGACTAACCT
TAAATAATTTTTTTTTAAAAACCCAATTTATTACTATATTTTTAGGTGTAAATTTAAC
ATTTTTTCCCCAACACTTTTTAGGGCTAAGAGGAATGCCTCGGCGATATTCGGAT
TATCCAGATGCTTTCACTATCTGAAATGCCATTTCATCTATTGGTTCCATTATTTC
TCTCACAAGAATTATCTTTTTTTTATTTATCTTATGAGAAAGGCTTTCTTCCCAAC
GTAAATCTTTGAGAACTTTAAATGTAACAACTTCAATTGAATGGTTCAAGCCCTAC
CCCCTAGTG
AQU13
ATTATTAGACAAGAAAGGGGGAAAAAAGAAGCTTTTGGACCTTAGGAATAATTTA
TGCCATGATAGCTATCGGATTATTAGGATTCGTAGTTTGAGCTCACCATATATTT
ACAGTAGATAGATGTAGATACCCGAGCTTACTTCACTTCAGCAACTATAATTATT
GCGGTTCCAACTGGGATTAAAGTCTTTAGATGACTAGCCACCTTTCATGGAACT
CAAATTTTAAATAGACCAGTAACCCTGTGAGCTTTAGGTTTTGTATTTTTATTTAC
AGTAGGAGGATTAACAGGGGTAATTTTAGCTAATTCTTCAATTGATATTATTCTTC
ACGATACTTACTATGTAGTAGCTCATTTTCACTATGTTCTTTCTATAGGAGCTGTA
TTTGCAATTATAGCCGGAATTGTCCAATGATTCCCATTATTTACCGGACTAACCT
TAAATAATTTTTTTTTAAAAACCCAATTTATTACTATATTTTTAGGTGTAAATTTAAC
ATTTTTTCCCCAACACTTTTTAGGGCTAAGAGGAATGCCTCGGCGATATTCGGAT
TATCCAGATGCTTTCACTATCTGAAATGCCATTTCATCTATTGGTTCCATTATTTC
TCTCACAAGAATTATCTTTTTTTTATTTATCTTATGAGAAAGGCTTTCTTCCCAAC
GTAAATCTTTGAGAACTTTAAATGTAACAACTTCAATTGAATGGTTCAAGCCCTAC
CCCCTAGTG
AQU14
ATTATTAGACAAGAAAGGGGGAAAAAAGAAGCTTTTGGACCTTAGGAATAATTTA
TGCCATGATAGCTATCGGATTATTAGGATTCGTAGTTTGAGCTCACCATATATTT
ACAGTAGATAGATGTAGATACCCGAGCTTACTTCACTTCAGCAACTATAATTATT
GCGGTTCCAACTGGGATTAAAGTCTTTAGATGACTAGCCACCTTTCATGGAACT
CAAATTTTAAATAGACCAGTAACCCTGTGAGCTTTAGGTTTTGTATTTTTATTTAC
AGTAGGAGGATTAACAGGGGTAATTTTAGCTAATTCTTCAATTGATATTATTCTTC
ACGATACTTACTATGTAGTAGCTCATTTTCACTATGTTCTTTCTATAGGAGCTGTA
TTTGCAATTATAGCCGGAATTGTCCAATGATTCCCATTATTTACCGGACTAACCT
TAAATAATTTTTTTTTAAAAACCCAATTTATTACTATATTTTTAGGTGTAAATTTAAC
ATTTTTTCCCCAACACTTTTTAGGGCTAAGAGGAATGCCTCGGCGATATTCGGAT
TATCCAGATGCTTTCACTATCTGAAATGCCATTTCATCTATTGGTTCCATTATTTC

TCTCACAAGAATTATCTTTTTTTTATTTATCTTATGAGAAAGGCTTTCTTCCCAAC
GTAAATCTTTGAGAACTTTAAATGTAACAACTTCAATTGAATGGTTCAAGCCCTAC
CCCCTAGTG
AQU4
ATTATTAGACAAGAAAGGGGGAAAAAAGAAGCTTTTGGACCTTAGGAATAATTTA
TGCCATGATAGCTATCGGATTATTAGGATTCGTAGTCTGAGCTCACCATATATTT
ACAGTAGATAGATGTAGATACCCGAGCTTACTTCACTTCAGCAACTATAATTATT
GCGGTTCCAACTGGGATTAAAGTCTTTAGATGACTAGCCACCTTTCATGGAACT
CAAATTTTAAATAGACCAGTAACCCTGTGAGCTTTAGGTTTTGTATTTTTATTTAC
AGTAGGAGGATTAACAGGGGTAATTTTAGCTAATTCTTCAATTGATATTATTCTTC
ACGATACTTACTATGTAGTAGCTCATTTTCACTATGTTCTTTCTATAGGAGCTGTA
TTTGCAATTATAGCCGGAATTGTCCAATGATTCCCATTATTTACCGGACTAACCT
TAAATAATTTTTTTTTAAAAACCCAATTTATTACTATATTTTTAGGTGTAAATTTAAC
ATTTTTTCCCCAACACTTTTTAGGGCTAAGAGGAATGCCTCGGCGATATTCGGAT
TATCCAGATGCTTTCACTATCTGAAATGCCATTTCATCTATTGGTTCCATTATTTC
TCTCACAAGAATTATCTTTTTTTTATTTATCTTATGAGAAAGGCTTTCTTCCCAAC
GTAAATCTTTGAGAACTTTAAATGTAACAACTTCAATTGAATGGTTCAAGCCCTAC
CCCCTAGTG
bja1
ATTATTAGACAAGAAAGGGGGAAAAAAGAAGCTTTTGGACCTTAGGAATAATTTA
TGCCATGATAGCTATCGGATTATTAGGATTCGTAGTTTGAGCTCACCATATATTT
ACAGTAGATAGATGTAGATACCCGAGCTTACTTCACTTCAGCAACTATAATTATT
GCGGTTCCAACTGGGATTAAAGTCTTTAGATGACTAGCCACCTTTCATGGAACT
CAAATTTTAAATAGACCAGTAACCCTGTGAGCTTTAGGTTTTGTATTTTTATTTAC
AGTAGGAGGATTAACAGGGGTAATTTTAGCTAATTCTTCAATTGATATTATTCTTC
ACGATACTTACTATGTAGTAGCTCATTTTCACTATGTTCTTTCTATAGGAGCTGTA
TTTGCAATTATAGCCGGAATTGTCCAATGATTCCCATTATTTACCGGACTAACCT
TAAATAATTTTTTTTTAAAAACCCAATTTATTACTATATTTTTAGGTGTAAATTTAAC
ATTTTTTCCCCAACACTTTTTAGGGCTAAGAGGAATGCCTCGGCGATATTCGGAT
TATCCAGATGCTTTCACTATCTGAAATGCCATTTCATCTATTGGTTCCATTATTTC
TCTCACAAGAATTATCTTTTTTTTATTTATCTTATGAGAAAGGCTTTCTTCCCAAC
GTAAATCTTTGAGAACTTTAAATGTAACAACTTCAATTGAATGGTTCAAGCCCTAC
CCCCTAGTG
BJA2
ATTATTAGACAAGAAAGGGGGAAAAAAGAAGCTTTTGGACCTTAGGAATAATTTA
TGCCATGATAGCTATCGGATTATTAGGATTCGTAGTTTGAGCTCACCATATATTT
ACAGTAGATAGATGTAGATACCCGAGCTTACTTCACTTCAGCAACTATAATTATT
GCGGTTCCAACTGGGATTAAAGTCTTTAGATGACTAGCCACCTTTCATGGAACT
CAAATTTTAAATAGACCAGTAACCCTGTGAGCTTTAGGTTTTGTATTTTTATTTAC
AGTAGGAGGATTAACAGGGGTAATTTTAGCTAATTCTTCAATTGATATTATTCTTC
ACGATACTTACTATGTAGTAGCTCATTTTCACTATGTTCTTTCTATAGGAGCTGTA
TTTGCAATTATAGCCGGAATTGTCCAATGATTCCCATTATTTACCGGACTAACCT
TAAATAATTTTTTTTTAAAAACCCAATTTATTACTATATTTTTAGGTGTAAATTTAAC
ATTTTTTCCCCAACACTTTTTAGGGCTAAGAGGAATGCCTCGGCGATATTCGGAT
TATCCAGATGCTTTCACTATCTGAAATGCCATTTCATCTATTGGTTCCATTATTTC
TCTCACAAGAATTATCTTTTTTTTATTTATCTTATGAGAAAGGCTTTCTTCCCAAC
GTAAATCTTTGAGAACTTTAAATGTAACAACTTCAATTGAATGGTTCAAGCCCTAC
CCCCTAGTG
BJA4
ATTATTAGACAAGAAAGGGGGAAAAAAGAAGCTTTTGGACCTTAGGAATAATTTA

TGCCATGATAGCTATCGGATTATTAGGATTCGTAGTTTGAGCTCACCATATATTT
ACAGTAGATAGATGTAGATACCCGAGCTTACTTCACTTCAGCAACTATAATTATT
GCGGTTCCAACTGGGATTAAAGTCTTTAGATGACTAGCCACCTTTCATGGAACT
CAAATTTTAAATAGACCAGTAACCCTGTGAGCTTTAGGTTTTGTATTTTTATTTAC
AGTAGGAGGATTAACAGGGGTAATTTTAGCTAATTCTTCAATTGATATTATTCTTC
ACGATACTTACTATGTAGTAGCTCATTTTCACTATGTTCTTTCTATAGGAGCTGTA
TTTGCAATTATAGCCGGAATTGTCCAATGATTCCCATTATTTACCGGACTAACCT
TAAATAATTTTTTTTTAAAAACCCAATTTATTACTATATTTTTAGGTGTAAATTTAAC
ATTTTTTCCCCAACACTTTTTAGGGCTAAGAGGAATGCCTCGGCGATATTCGGAT
TATCCAGATGCTTTCACTATCTGAAATGCCATTTCATCTATTGGTTCCATTATTTC
TCTCACAAGAATTATCTTTTTTTTATTTATCTTATGAGAAAGGCTTTCTTCCCAAC
GTAAATCTTTGAGAACTTTAAATGTAACAACTTCAATTGAATGGTTCAAGCCCTAC
CCCCTAGTG
CAM1
ATTATTAGACAAGAAAGGGGGAAAAAAGAAGCTTTTGGACCTTAGGAATAATTTA
TGCCATGATAGCTATCGGATTATTAGGATTCGTAGTTTGAGCTCACCATATATTT
ACAGTAGATAGATGTAGATACCCGAGCTTACTTCACTTCAGCAACTATAATTATT
GCGGTTCCAACTGGGATTAAAGTCTTTAGATGACTAGCCACCTTTCATGGAACT
CAAATTTTAAATAGACCAGTAACCCTGTGAGCTTTAGGTTTTGTATTTTTATTTAC
AGTAGGAGGATTAACAGGGGTAATTTTAGCTAATTCTTCAATTGATATTATTCTTC
ACGATACTTACTATGTAGTAGCTCATTTTCACTATGTTCTTTCTATAGGAGCTGTA
TTTGCAATTATAGCCGGAATTGTCCAATGATTCCCATTATTTACCGGACTAACCT
TAAATAATTTTTTTTTAAAAACCCAATTTATTACTATATTTTTAGGTGTAAATTTAAC
ATTTTTTCCCCAACACTTTTTAGGGCTAAGAGGAATGCCTCGGCGATATTCGGAT
TATCCAGATGCTTTCACTATCTGAAATGCCATTTCATCTATTGGTTCCATTATTTC
TCTCACAAGAATTATCTTTTTTTTATTTATCTTATGAGAAAGGCTTTCTTCCCAAC
GTAAATCTTTGAGAACTTTAAATGTAACAACTTCAATTGAATGGTTCAAGCCCTAC
CCCCTAGTG
CAM2
ATTATTAGACAAGAAAGGGGGAAAAAAGAAGCTTTTGGACCTTAGGAATAATTTA
TGCCATGATAGCTATCGGATTATTAGGATTCGTAGTTTGAGCTCACCATATATTT
ACAGTAGATAGATGTAGATACCCGAGCTTACTTCACTTCAGCAACTATAATTATT
GCGGTTCCAACTGGGATTAAAGTCTTTAGATGACTAGCCACCTTTCATGGAACT
CAAATTTTAAATAGACCAGTAACCCTGTGAGCTTTAGGTTTTGTATTTTTATTTAC
AGTAGGAGGATTAACAGGGGTAATTTTAGCTAATTCTTCAATTGATATTATTCTTC
ACGATACTTACTATGTAGTAGCTCATTTTCACTATGTTCTTTCTATAGGAGCTGTA
TTTGCAATTATAGCCGGAATTGTCCAATGATTCCCATTATTTACCGGACTAACCT
TAAATAATTTTTTTTTAAAAACCCAATTTATTACTATATTTTTAGGTGTAAATTTAAC
ATTTTTTCCCCAACACTTTTTAGGGCTAAGAGGAATGCCTCGGCGATATTCGGAT
TATCCAGATGCTTTCACTATCTGAAATGCCATTTCATCTATTGGTTCCATTATTTC
TCTCACAAGAATTATCTTTTTTTTATTTATCTTATGAGAAAGGCTTTCTTCCCAAC
GTAAATCTTTGAGAACTTTAAATGTAACAACTTCAATTGAATGGTTCAAGCCCTAC
CCCCTAGTG
CMO1_1
ATTATTAGACAAGAAAGGGGGAAAAAAGAAGCTTTTGGACCTTAGGAATAATTTA
TGCCATGATAGCTATCGGATTATTAGGATTCGTAGTTTGAGCTCACCATATATTT
ACAGTAGATAGATGTAGATACCCGAGCTTACTTCACTTCAGCAACTATAATTATT
GCGGTTCCAACTGGGATTAAAGTCTTTAGATGACTAGCCACCTTTCATGGAACT
CAAATTTTAAATAGACCAGTAACCCTGTGAGCTTTAGGTTTTGTATTTTTATTTAC
AGTAGGAGGATTAACAGGGGTAATTTTAGCTAATTCTTCAATTGATATTATTCTTC

ACGATACTTACTATGTAGTAGCTCATTTTCACTATGTTCTTTCTATAGGAGCTGTA
TTTGCAATTATAGCCGGAATTGTCCAATGATTCCCATTATTTACCGGACTAACCT
TAAATAATTTTTTTTTAAAAACCCAATTTATTACTATATTTTTAGGTGTAAATTTAAC
ATTTTTTCCCCAACACTTTTTAGGGCTAAGAGGAATGCCTCGGCGATATTCGGAT
TATCCAGATGCTTTCACTATCTGAAATGCCATTTCATCTATTGGTTCCATTATTTC
TCTCACAAGAATTATCTTTTTTTTATTTATCTTATGAGAAAGGCTTTCTTCCCAAC
GTAAATCTTTGAGAACTTTAAATGTAACAACTTCAATTGAATGGTTCAAGCCCTAC
CCCCTAGTG
CMO1_2
ATTATTAGACAAGAAAGGGGGAAAAAAGAAGCTTTTGGACCTTAGGAATAATTTA
TGCCATGATAGCTATCGGATTATTAGGATTCGTAGTTTGAGCTCACCATATATTT
ACAGTAGATAGATGTAGATACCCGAGCTTACTTCACTTCAGCAACTATAATTATT
GCGGTTCCAACTGGGATTAAAGTCTTTAGATGACTAGCCACCTTTCATGGAACT
CAAATTTTAAATAGACCAGTAACCCTGTGAGCTTTAGGTTTTGTATTTTTATTTAC
AGTAGGAGGATTAACAGGGGTAATTTTAGCTAATTCTTCAATTGATATTATTCTTC
ACGATACTTACTATGTAGTAGCTCATTTTCACTATGTTCTTTCTATAGGAGCTGTA
TTTGCAATTATAGCCGGAATTGTCCAATGATTCCCATTATTTACCGGACTAACCT
TAAATAATTTTTTTTTAAAAACCCAATTTATTACTATATTTTTAGGTGTAAATTTAAC
ATTTTTTCCCCAACACTTTTTAGGGCTAAGAGGAATGCCTCGGCGATATTCGGAT
TATCCAGATGCTTTCACTATCTGAAATGCCATTTCATCTATTGGTTCCATTATTTC
TCTCACAAGAATTATCTTTTTTTTATTTATCTTATGAGAAAGGCTTTCTTCCCAAC
GTAAATCTTTGAGAACTTTAAATGTAACAACTTCAATTGAATGGTTCAAGCCCTAC
CCCCTAGTG
CMO2_1
ATTATTAGACAAGAAAGGGGGAAAAAAGAAGCTTTTGGACCTTAGGAATAATTTA
TGCCATGATAGCTATCGGATTATTAGGATTCGTAGTTTGAGCTCACCATATATTT
ACAGTAGATAGATGTAGATACCCGAGCTTACTTCACTTCAGCAACTATAATTATT
GCGGTTCCAACTGGGATTAAAGTCTTTAGATGACTAGCCACCTTTCATGGAACT
CAAATTTTAAATAGACCAGTAACCCTGTGAGCTTTAGGTTTTGTATTTTTATTTAC
AGTAGGAGGATTAACAGGGGTAATTTTAGCTAATTCTTCAATTGATATTATTCTTC
ACGATACTTACTATGTAGTAGCTCATTTTCACTATGTTCTTTCTATAGGAGCTGTA
TTTGCAATTATAGCCGGAATTGTCCAATGATTCCCATTATTTACCGGACTAACCT
TAAATAATTTTTTTTTAAAAACCCAATTTATTACTATATTTTTAGGTGTAAATTTAAC
ATTTTTTCCCCAACACTTTTTAGGGCTAAGAGGAATGCCTCGGCGATATTCGGAT
TATCCAGATGCTTTCACTATCTGAAATGCCATTTCATCTATTGGTTCCATTATTTC
TCTCACAAGAATTATCTTTTTTTTATTTATCTTATGAGAAAGGCTTTCTTCCCAAC
GTAAATCTTTGAGAACTTTAAATGTAACAACTTCAATTGAATGGTTCAAGCCCTAC
CCCCTAGTG
CMO2_2
ATTATTAGACAAGAAAGGGGGAAAAAAGAAGCTTTTGGACCTTAGGAATAATTTA
TGCCATGATAGCTATCGGATTATTAGGATTCGTAGTTTGAGCTCACCATATATTT
ACAGTAGATAGATGTAGATACCCGAGCTTACTTCACTTCAGCAACTATAATTATT
GCGGTTCCAACTGGGATTAAAGTCTTTAGATGACTAGCCACCTTTCATGGAACT
CAAATTTTAAATAGACCAGTAACCCTGTGAGCTTTAGGTTTTGTATTTTTATTTAC
AGTAGGAGGATTAACAGGGGTAATTTTAGCTAATTCTTCAATTGATATTATTCTTC
ACGATACTTACTATGTAGTAGCTCATTTTCACTATGTTCTTTCTATAGGAGCTGTA
TTTGCAATTATAGCCGGAATTGTCCAATGATTCCCATTATTTACCGGACTAACCT
TAAATAATTTTTTTTTAAAAACCCAATTTATTACTATATTTTTAGGTGTAAATTTAAC
ATTTTTTCCCCAACACTTTTTAGGGCTAAGAGGAATGCCTCGGCGATATTCGGAT
TATCCAGATGCTTTCACTATCTGAAATGCCATTTCATCTATTGGTTCCATTATTTC

TCTCACAAGAATTATCTTTTTTTTATTTATCTTATGAGAAAGGCTTTCTTCCCAAC
GTAAATCTTTGAGAACTTTAAATGTAACAACTTCAATTGAATGGTTCAAGCCCTAC
CCCCTAGTG
COI1
ATTATTAGACAAGAAAGGGGGAAAAAAGAAGCTTTTGGACCTTAGGAATAATTTA
TGCCATGATAGCTATCGGATTATTAGGATTCGTAGTTTGAGCTCACCATATATTT
ACAGTAGATAGATGTAGATACCCGAGCTTACTTCACTTCAGCAACTATAATTATT
GCGGTTCCAACTGGGATTAAAGTCTTTAGATGACTAGCCACCTTTCATGGAACT
CAAATTTTAAATAGACCAGTAACCCTGTGAGCTTTAGGTTTTGTATTTTTATTTAC
AGTAGGAGGATTAACAGGGGTAATTTTAGCTAATTCTTCAATTGATATTATTCTTC
ACGATACTTACTATGTAGTAGCTCATTTTCACTATGTTCTTTCTATAGGAGCTGTA
TTTGCAATTATAGCCGGAATTGTCCAATGATTCCCATTATTTACCGGACTAACCT
TAAATAATTTTTTTTTAAAAACCCAATTTATTACTATATTTTTAGGTGTAAATTTAAC
ATTTTTTCCCCAACACTTTTTAGGGCTAAGAGGAATGCCTCGGCGATATTCGGAT
TATCCAGATGCTTTCACTATCTGAAATGCCATTTCATCTATTGGTTCCATTATTTC
TCTCACAAGAATTATCTTTTTTTTATTTATCTTATGAGAAAGGCTTTCTTCCCAAC
GTAAATCTTTGAGAACTTTAAATGTAACAACTTCAATTGAATGGTTCAAGCCCTAC
CCCCTAGTG
CSJ1
ATTATTAGACAAGAAAGGGGGAAAAAAGAAGCTTTTGGACCTTAGGAATAATTTA
TGCCATGATAGCTATCGGATTATTAGGATTCGTAGTTTGAGCTCACCATATATTT
ACAGTAGATAGATGTAGATACCCGAGCTTACTTCACTTCAGCAACTATAATTATT
GCGGTTCCAACTGGGATTAAAGTCTTTAGATGACTAGCCACCTTTCATGGAACT
CAAATTTTAAATAGACCAGTAACCCTGTGAGCTTTAGGTTTTGTATTTTTATTTAC
AGTAGGAGGATTAACAGGGGTAATTTTAGCTAATTCTTCAATTGATATTATTCTTC
ACGATACTTACTATGTAGTAGCTCATTTTCACTATGTTCTTTCTATAGGAGCTGTA
TTTGCAATTATAGCCGGAATTGTCCAATGATTCCCATTATTTACCGGACTAACCT
TAAATAATTTTTTTTTAAAAACCCAATTTATTACTATATTTTTAGGTGTAAATTTAAC
ATTTTTTCCCCAACACTTTTTAGGGCTAAGAGGAATGCCTCGGCGATATTCGGAT
TATCCAGATGCTTTCACTATCTGAAATGCCATTTCATCTATTGGTTCCATTATTTC
TCTCACAAGAATTATCTTTTTTTTATTTATCTTATGAGAAAGGCTTTCTTCCCAAC
GTAAATCTTTGAGAACTTTAAATGTAACAACTTCAATTGAATGGTTCAAGCCCTAC
CCCCTAGTG
ERV1
ATTATTAGACAAGAAAGGGGGAAAAAAGAAGCTTTTGGACCTTAGGAATAATTTA
TGCCATGATAGCTATCGGATTATTAGGATTCGTAGTTTGAGCTCACCATATATTT
ACAGTAGATAGATGTAGATACCCGAGCTTACTTCACTTCAGCAACTATAATTATT
GCGGTTCCAACTGGGATTAAAGTCTTTAGATGACTAGCCACCTTTCATGGAACT
CAAATTTTAAATAGACCAGTAACCCTGTGAGCTTTAGGTTTTGTATTTTTATTTAC
AGTAGGAGGATTAACAGGGGTAATTTTAGCTAATTCTTCAATTGATATTATTCTTC
ACGATACTTACTATGTAGTAGCTCATTTTCACTATGTTCTTTCTATAGGAGCTGTA
TTTGCAATTATAGCCGGAATTGTCCAATGATTCCCATTATTTACCGGACTAACCT
TAAATAATTTTTTTTTAAAAACCCAATTTATTACTATATTTTTAGGTGTAAATTTAAC
ATTTTTTCCCCAACACTTTTTAGGGCTAAGAGGAATGCCTCGGCGATATTCGGAT
TATCCAGATGCTTTCACTATCTGAAATGCCATTTCATCTATTGGTTCCATTATTTC
TCTCACAAGAATTATCTTTTTTTTATTTATCTTATGAGAAAGGCTTTCTTCCCAAC
GTAAATCTTTGAGAACTTTAAATGTAACAACTTCAATTGAATGGTTCAAGCCCTAC
CCCCTAGTG
ERV2
ATTATTAGACAAGAAAGGGGGAAAAAAGAAGCTTTTGGACCTTAGGAATAATTTA

TGCCATGATAGCTATCGGATTATTAGGATTCGTAGTTTGAGCTCACCATATATTT
ACAGTAGATAGATGTAGATACCCGAGCTTACTTCACTTCAGCAACTATAATTATT
GCGGTTCCAACTGGGATTAAAGTCTTTAGATGACTAGCCACCTTTCATGGAACT
CAAATTTTAAATAGACCAGTAACCCTGTGAGCTTTAGGTTTTGTATTTTTATTTAC
AGTAGGAGGATTAACAGGGGTAATTTTAGCTAATTCTTCAATTGATATTATTCTTC
ACGATACTTACTATGTAGTAGCTCATTTTCACTATGTTCTTTCTATAGGAGCTGTA
TTTGCAATTATAGCCGGAATTGTCCAATGATTCCCATTATTTACCGGACTAACCT
TAAATAATTTTTTTTTAAAAACCCAATTTATTACTATATTTTTAGGTGTAAATTTAAC
ATTTTTTCCCCAACACTTTTTAGGGCTAAGAGGAATGCCTCGGCGATATTCGGAT
TATCCAGATGCTTTCACTATCTGAAATGCCATTTCATCTATTGGTTCCATTATTTC
TCTCACAAGAATTATCTTTTTTTTATTTATCTTATGAGAAAGGCTTTCTTCCCAAC
GTAAATCTTTGAGAACTTTAAATGTAACAACTTCAATTGAATGGTTCAAGCCCTAC
CCCCTAGTG
GOV1
ATTATTAGACAAGAAAGGGGGAAAAAAGAAGCTTTTGGACCTTAGGAATAATTTA
TGCCATGATAGCTATCGGATTATTAGGATTCGTAGTTTGAGCTCACCATATATTT
ACAGTAGATAGATGTAGATACCCGAGCTTACTTCACTTCAGCAACTATAATTATT
GCGGTTCCAACTGGGATTAAAGTCTTTAGATGACTAGCCACCTTTCATGGAACT
CAAATTTTAAATAGACCAGTAACCCTGTGAGCTTTAGGTTTTGTATTTTTATTTAC
AGTAGGAGGATTAACAGGGGTAATTTTAGCTAATTCTTCAATTGATATTATTCTTC
ACGATACTTACTATGTAGTAGCTCATTTTCACTATGTTCTTTCTATAGGAGCTGTA
TTTGCAATTATAGCCGGAATTGTCCAATGATTCCCATTATTTACCGGACTAACCT
TAAATAATTTTTTTTTAAAAACCCAATTTATTACTATATTTTTAGGTGTAAATTTAAC
ATTTTTTCCCCAACACTTTTTAGGGCTAAGAGGAATGCCTCGGCGATATTCGGAT
TATCCAGATGCTTTCACTATCTGAAATGCCATTTCATCTATTGGTTCCATTATTTC
TCTCACAAGAATTATCTTTTTTTTATTTATCTTATGAGAAAGGCTTTCTTCCCAAC
GTAAATCTTTGAGAACTTTAAATGTAACAACTTCAATTGAATGGTTCAAGCCCTAC
CCCCTAGTG
GOV2
ATTATTAGACAAGAAAGGGGGAAAAAAGAAGCTTTTGGACCTTAGGAATAATTTA
TGCCATGATAGCTATCGGATTATTAGGATTCGTAGTTTGAGCTCACCATATATTT
ACAGTAGATAGATGTAGATACCCGAGCTTACTTCACTTCAGCAACTATAATTATT
GCGGTTCCAACTGGGATTAAAGTCTTTAGATGACTAGCCACCTTTCATGGAACT
CAAATTTTAAATAGACCAGTAACCCTGTGAGCTTTAGGTTTTGTATTTTTATTTAC
AGTAGGAGGATTAACAGGGGTAATTTTAGCTAATTCTTCAATTGATATTATTCTTC
ACGATACTTACTATGTAGTAGCTCATTTTCACTATGTTCTTTCTATAGGAGCTGTA
TTTGCAATTATAGCCGGAATTGTCCAATGATTCCCATTATTTACCGGACTAACCT
TAAATAATTTTTTTTTAAAAACCCAATTTATTACTATATTTTTAGGTGTAAATTTAAC
ATTTTTTCCCCAACACTTTTTAGGGCTAAGAGGAATGCCTCGGCGATATTCGGAT
TATCCAGATGCTTTCACTATCTGAAATGCCATTTCATCTATTGGTTCCATTATTTC
TCTCACAAGAATTATCTTTTTTTTATTTATCTTATGAGAAAGGCTTTCTTCCCAAC
GTAAATCTTTGAGAACTTTAAATGTAACAACTTCAATTGAATGGTTCAAGCCCTAC
CCCCTAGTG
HEM1
ATTATTAGACAAGAAAGGGGGAAAAAAGAAGCTTTTGGACCTTAGGAATAATTTA
TGCCATGATAGCTATCGGATTATTAGGATTCGTAGTTTGAGCTCACCATATATTT
ACAGTAGATAGATGTAGATACCCGAGCTTACTTCACTTCAGCAACTATAATTATT
GCGGTTCCAACTGGGATTAAAGTCTTTAGATGACTAGCCACCTTTCATGGAACT
CAAATTTTAAATAGACCAGTAACCCTGTGAGCTTTAGGTTTTGTATTTTTATTTAC
AGTAGGAGGATTAACAGGGGTAATTTTAGCTAATTCTTCAATTGATATTATTCTTC

ACGATACTTACTATGTAGTAGCTCATTTTCACTATGTTCTTTCTATAGGAGCTGTA
TTTGCAATTATAGCCGGAATTGTCCAATGATTCCCATTATTTACCGGACTAACCT
TAAATAATTTTTTTTTAAAAACCCAATTTATTACTATATTTTTAGGTGTAAATTTAAC
ATTTTTTCCCCAACACTTTTTAGGGCTAAGAGGAATGCCTCGGCGATATTCGGAT
TATCCAGATGCTTTCACTATCTGAAATGCCATTTCATCTATTGGTTCCATTATTTC
TCTCACAAGAATTATCTTTTTTTTATTTATCTTATGAGAAAGGCTTTCTTCCCAAC
GTAAATCTTTGAGAACTTTAAATGTAACAACTTCAATTGAATGGTTCAAGCCCTAC
CCCCTAGTG
HEM2
ATTATTAGACAAGAAAGGGGGAAAAAAGAAGCTTTTGGACCTTAGGAATAATTTA
TGCCATGATAGCTATCGGATTATTAGGATTCGTAGTTTGAGCTCACCATATATTT
ACAGTAGATAGATGTAGATACCCGAGCTTACTTCACTTCAGCAACTATAATTATT
GCGGTTCCAACTGGGATTAAAGTCTTTAGATGACTAGCCACCTTTCATGGAACT
CAAATTTTAAATAGACCAGTAACCCTGTGAGCTTTAGGTTTTGTATTTTTATTTAC
AGTAGGAGGATTAACAGGGGTAATTTTAGCTAATTCTTCAATTGATATTATTCTTC
ACGATACTTACTATGTAGTAGCTCATTTTCACTATGTTCTTTCTATAGGAGCTGTA
TTTGCAATTATAGCCGGAATTGTCCAATGATTCCCATTATTTACCGGACTAACCT
TAAATAATTTTTTTTTAAAAACCCAATTTATTACTATATTTTTAGGTGTAAATTTAAC
ATTTTTTCCCCAACACTTTTTAGGGCTAAGAGGAATGCCTCGGCGATATTCGGAT
TATCCAGATGCTTTCACTATCTGAAATGCCATTTCATCTATTGGTTCCATTATTTC
TCTCACAAGAATTATCTTTTTTTTATTTATCTTATGAGAAAGGCTTTCTTCCCAAC
GTAAATCTTTGAGAACTTTAAATGTAACAACTTCAATTGAATGGTTCAAGCCCTAC
CCCCTAGTG
HER1
ATTATTAGACAAGAAAGGGGGAAAAAAGAAGCTTTTGGACCTTAGGAATAATTTA
TGCCATGATAGCTATCGGATTATTAGGATTCGTAGTTTGAGCTCACCATATATTT
ACAGTAGATAGATGTAGATACCCGAGCTTACTTCACTTCAGCAACTATAATTATT
GCGGTTCCAACTGGGATTAAAGTCTTTAGATGACTAGCCACCTTTCATGGAACT
CAAATTTTAAATAGACCAGTAACCCTGTGAGCTTTAGGTTTTGTATTTTTATTTAC
AGTAGGAGGATTAACAGGGGTAATTTTAGCTAATTCTTCAATTGATATTATTCTTC
ACGATACTTACTATGTAGTAGCTCATTTTCACTATGTTCTTTCTATAGGAGCTGTA
TTTGCAATTATAGCCGGAATTGTCCAATGATTCCCATTATTTACCGGACTAACCT
TAAATAATTTTTTTTTAAAAACCCAATTTATTACTATATTTTTAGGTGTAAATTTAAC
ATTTTTTCCCCAACACTTTTTAGGGCTAAGAGGAATGCCTCGGCGATATTCGGAT
TATCCAGATGCTTTCACTATCTGAAATGCCATTTCATCTATTGGTTCCATTATTTC
TCTCACAAGAATTATCTTTTTTTTATTTATCTTATGAGAAAGGCTTTCTTCCCAAC
GTAAATCTTTGAGAACTTTAAATGTAACAACTTCAATTGAATGGTTCAAGCCCTAC
CCCCTAGTG
HER2
ATTATTAGACAAGAAAGGGGGAAAAAAGAAGCTTTTGGACCTTAGGAATAATTTA
TGCCATGATAGCTATCGGATTATTAGGATTCGTAGTTTGAGCTCACCATATATTT
ACAGTAGATAGATGTAGATACCCGAGCTTACTTCACTTCAGCAACTATAATTATT
GCGGTTCCAACTGGGATTAAAGTCTTTAGATGACTAGCCACCTTTCATGGAACT
CAAATTTTAAATAGACCAGTAACCCTGTGAGCTTTAGGTTTTGTATTTTTATTTAC
AGTAGGAGGATTAACAGGGGTAATTTTAGCTAATTCTTCAATTGATATTATTCTTC
ACGATACTTACTATGTAGTAGCTCATTTTCACTATGTTCTTTCTATAGGAGCTGTA
TTTGCAATTATAGCCGGAATTGTCCAATGATTCCCATTATTTACCGGACTAACCT
TAAATAATTTTTTTTTAAAAACCCAATTTATTACTATATTTTTAGGTGTAAATTTAAC
ATTTTTTCCCCAACACTTTTTAGGGCTAAGAGGAATGCCTCGGCGATATTCGGAT
TATCCAGATGCTTTCACTATCTGAAATGCCATTTCATCTATTGGTTCCATTATTTC

TCTCACAAGAATTATCTTTTTTTTATTTATCTTATGAGAAAGGCTTTCTTCCCAAC
GTAAATCTTTGAGAACTTTAAATGTAACAACTTCAATTGAATGGTTCAAGCCCTAC
CCCCTAGTG
HEV1
ATTATTAGACAAGAAAGGGGGAAAAAAGAAGCTTTTGGACCTTAGGAATAATTTA
TGCCATGATAGCTATCGGATTATTAGGATTCGTAGTTTGAGCTCACCATATATTT
ACAGTAGATAGATGTAGATACCCGAGCTTACTTCACTTCAGCAACTATAATTATT
GCGGTTCCAACTGGGATTAAAGTCTTTAGATGACTAGCCACCTTTCATGGAACT
CAAATTTTAAATAGACCAGTAACCCTGTGAGCTTTAGGTTTTGTATTTTTATTTAC
AGTAGGAGGATTAACAGGGGTAATTTTAGCTAATTCTTCAATTGATATTATTCTTC
ACGATACTTACTATGTAGTAGCTCATTTTCACTATGTTCTTTCTATAGGAGCTGTA
TTTGCAATTATAGCCGGAATTGTCCAATGATTCCCATTATTTACCGGACTAACCT
TAAATAATTTTTTTTTAAAAACCCAATTTATTACTATATTTTTAGGTGTAAATTTAAC
ATTTTTTCCCCAACACTTTTTAGGGCTAAGAGGAATGCCTCGGCGATATTCGGAT
TATCCAGATGCTTTCACTATCTGAAATGCCATTTCATCTATTGGTTCCATTATTTC
TCTCACAAGAATTATCTTTTTTTTATTTATCTTATGAGAAAGGCTTTCTTCCCAAC
GTAAATCTTTGAGAACTTTAAATGTAACAACTTCAATTGAATGGTTCAAGCCCTAC
CCCCTAGTG
HEV1_2
ATTATTAGACAAGAAAGGGGGAAAAAAGAAGCTTTTGGACCTTAGGAATAATTTA
TGCCATGATAGCTATCGGATTATTAGGATTCGTAGTTTGAGCTCACCATATATTT
ACAGTAGATAGATGTAGATACCCGAGCTTACTTCACTTCAGCAACTATAATTATT
GCGGTTCCAACTGGGATTAAAGTCTTTAGATGACTAGCCACCTTTCATGGAACT
CAAATTTTAAATAGACCAGTAACCCTGTGAGCTTTAGGTTTTGTATTTTTATTTAC
AGTAGGAGGATTAACAGGGGTAATTTTAGCTAATTCTTCAATTGATATTATTCTTC
ACGATACTTACTATGTAGTAGCTCATTTTCACTATGTTCTTTCTATAGGAGCTGTA
TTTGCAATTATAGCCGGAATTGTCCAATGATTCCCATTATTTACCGGACTAACCT
TAAATAATTTTTTTTTAAAAACCCAATTTATTACTATATTTTTAGGTGTAAATTTAAC
ATTTTTTCCCCAACACTTTTTAGGGCTAAGAGGAATGCCTCGGCGATATTCGGAT
TATCCAGATGCTTTCACTATCTGAAATGCCATTTCATCTATTGGTTCCATTATTTC
TCTCACAAGAATTATCTTTTTTTTATTTATCTTATGAGAAAGGCTTTCTTCCCAAC
GTAAATCTTTGAGAACTTTAAATGTAACAACTTCAATTGAATGGTTCAAGCCCTAC
CCCCTAGTG
HEV2
ATTATTAGACAAGAAAGGGGGAAAAAAGAAGCTTTTGGACCTTAGGAATAATTTA
TGCCATGATAGCTATCGGATTATTAGGATTCGTAGTTTGAGCTCACCATATATTT
ACAGTAGATAGATGTAGATACCCGAGCTTACTTCACTTCAGCAACTATAATTATC
GCGGTTCCAACTGGGATTAAAGTCTTTAGGTGACTAGCCACCTTTCATGGAACT
CAAATTTTAAATAGACCAGTAACCCTGTGAGCTTTAGGTTTTGTATTTTTATTTAC
AGTAGGAGGATTAACAGGGGTAATTTTAGCTAATTCTTCAATTGATATTATTCTTC
ACGATACTTACTATGTAGTAGCTCATTTTCACTATGTTCTTTCTATAGGAGCTGTA
TTTGCAATTATAGCCGGAATTGTCCAATGATTCCCATTATTTACTGGACTAACCTT
AAATAATTTTTTTTTAAAAACCCAATTTATTACTATATTTTTAGGTGTAAATTTAACA
TTTTTTCCTCAACACTTTTTAGGGCTAAGAGGAATGCCTCGGCGATATTCGGATT
ATCCAGATGCTTTCACTATCTGAAATGCCATTTCATCTATTGGTTCCATTATTTCT
CTCACAAGAATTATCTTTTTTTTATTTATCTTATGAGAAAGGCTTTCTTCCCAACG
TAAATCTTTGAGAACTTTAAATGTAACAACTTCAATTGAATGGTTCAAGCCCTACC
CCCTAGTG
HEV2_2
ATTATTAGACAAGAAAGGGGGAAAAAAGAAGCTTTTGGACCTTAGGAATAATTTA

TGCCATGATAGCTATCGGATTATTAGGATTCGTAGTTTGAGCTCACCATATATTT
ACAGTAGATAGATGTAGATACCCGAGCTTACTTCACTTCAGCAACTATAATTATC
GCGGTTCCAACTGGGATTAAAGTCTTTAGGTGACTAGCCACCTTTCATGGAACT
CAAATTTTAAATAGACCAGTAACCCTGTGAGCTTTAGGTTTTGTATTTTTATTTAC
AGTAGGAGGATTAACAGGGGTAATTTTAGCTAATTCTTCAATTGATATTATTCTTC
ACGATACTTACTATGTAGTAGCTCATTTTCACTATGTTCTTTCTATAGGAGCTGTA
TTTGCAATTATAGCCGGAATTGTCCAATGATTCCCATTATTTACTGGACTAACCTT
AAATAATTTTTTTTTAAAAACCCAATTTATTACTATATTTTTAGGTGTAAATTTAACA
TTTTTTCCTCAACACTTTTTAGGGCTAAGAGGAATGCCTCGGCGATATTCGGATT
ATCCAGATGCTTTCACTATCTGAAATGCCATTTCATCTATTGGTTCCATTATTTCT
CTCACAAGAATTATCTTTTTTTTATTTATCTTATGAGAAAGGCTTTCTTCCCAACG
TAAATCTTTGAGAACTTTAAATGTAACAACTTCAATTGAATGGTTCAAGCCCTACC
CCCTAGTG
JAB2
ATTATTAGACAAGAAAGGGGGAAAAAAGAAGCTTTTGGACCTTAGGAATAATTTA
TGCCATGATAGCTATCGGATTATTAGGATTCGTAGTTTGAGCTCACCATATATTT
ACAGTAGATAGATGTAGATACCCGAGCTTACTTCACTTCAGCAACTATAATTATT
GCGGTTCCAACTGGGATTAAAGTCTTTAGATGACTAGCCACCTTTCATGGAACT
CAAATTTTAAATAGACCAGTAACCCTGTGAGCTTTAGGTTTTGTATTTTTATTTAC
AGTAGGAGGATTAACAGGGGTAATTTTAGCTAATTCTTCAATTGATATTATTCTTC
ACGATACTTACTATGTAGTAGCTCATTTTCACTATGTTCTTTCTATAGGAGCTGTA
TTTGCAATTATAGCCGGAATTGTCCAATGATTCCCATTATTTACCGGACTAACCT
TAAATAATTTTTTTTTAAAAACCCAATTTATTACTATATTTTTAGGTGTAAATTTAAC
ATTTTTTCCCCAACACTTTTTAGGGCTAAGAGGAATGCCTCGGCGATATTCGGAT
TATCCAGATGCTTTCACTATCTGAAATGCCATTTCATCTATTGGTTCCATTATTTC
TCTCACAAGAATTATCTTTTTTTTATTTATCTTATGAGAAAGGCTTTCTTCCCAAC
GTAAATCTTTGAGAACTTTAAATGTAACAACTTCAATTGAATGGTTCAAGCCCTAC
CCCCTAGTG
JAB4
ATTATTAGACAAGAAAGGGGGAAAAAAGAAGCTTTTGGACCTTAGGAATAATTTA
TGCCATGATAGCTATCGGATTATTAGGATTCGTAGTTTGAGCTCACCATATATTT
ACAGTAGATAGATGTAGATACCCGAGCTTACTTCACTTCAGCAACTATAATTATT
GCGGTTCCAACTGGGATTAAAGTCTTTAGATGACTAGCCACCTTTCATGGAACT
CAAATTTTAAATAGACCAGTAACCCTGTGAGCTTTAGGTTTTGTATTTTTATTTAC
AGTAGGAGGATTAACAGGGGTAATTTTAGCTAATTCTTCAATTGATATTATTCTTC
ACGATACTTACTATGTAGTAGCTCATTTTCACTATGTTCTTTCTATAGGAGCTGTA
TTTGCAATTATAGCCGGAATTGTCCAATGATTCCCATTATTTACCGGACTAACCT
TAAATAATTTTTTTTTAAAAACCCAATTTATTACTATATTTTTAGGTGTAAATTTAAC
ATTTTTTCCCCAACACTTTTTAGGGCTAAGAGGAATGCCTCGGCGATATTCGGAT
TATCCAGATGCTTTCACTATCTGAAATGCCATTTCATCTATTGGTTCCATTATTTC
TCTCACAAGAATTATCTTTTTTTTATTTATCTTATGAGAAAGGCTTTCTTCCCAAC
GTAAATCTTTGAGAACTTTAAATGTAACAACTTCAATTGAATGGTTCAAGCCCTAC
CCCCTAGTG
LAV1
ATTATTAGACAAGAAAGGGGGAAAAAAGAAGCTTTTGGACCTTAGGAATAATTTA
TGCCATGATAGCTATCGGATTATTAGGATTCGTAGTTTGAGCTCACCATATATTT
ACAGTAGATAGATGTAGATACCCGAGCTTACTTCACTTCAGCAACTATAATTATT
GCGGTTCCAACTGGGATTAAAGTCTTTAGATGACTAGCCACCTTTCATGGAACT
CAAATTTTAAATAGACCAGTAACCCTGTGAGCTTTAGGTTTTGTATTTTTATTTAC
AGTAGGAGGATTAACAGGGGTAATTTTAGCTAATTCTTCAATTGATATTATTCTTC

ACGATACTTACTATGTAGTAGCTCATTTTCACTATGTTCTTTCTATAGGAGCTGTA
TTTGCAATTATAGCCGGAATTGTCCAATGATTCCCATTATTTACCGGACTAACCT
TAAATAATTTTTTTTTAAAAACCCAATTTATTACTATATTTTTAGGTGTAAATTTAAC
ATTTTTTCCCCAACACTTTTTAGGGCTAAGAGGAATGCCTCGGCGATATTCGGAT
TATCCAGATGCTTTCACTATCTGAAATGCCATTTCATCTATTGGTTCCATTATTTC
TCTCACAAGAATTATCTTTTTTTTTATTTATCTTATGAGAAAGGCTTTCTTCCCAAC
GTAAATCTTTGAGAACTTTAAATGTAACAACTTCAATTGAATGGTTCAAGCCCTAC
CCCCTAGTG
LAV2
ATTATTAGACAAGAAAGGGGGAAAAAAGAAGCTTTTGGACCTTAGGAATAATTTA
TGCCATGATAGCTATCGGATTATTAGGATTCGTAGTTTGAGCTCACCATATATTT
ACAGTAGATAGATGTAGATACCCGAGCTTACTTCACTTCAGCAACTATAATTATT
GCGGTTCCAACTGGGATTAAAGTCTTTAGATGACTAGCCACCTTTCATGGAACT
CAAATTTTAAATAGACCAGTAACCCTGTGAGCTTTAGGTTTTGTATTTTTATTTAC
AGTAGGAGGATTAACAGGGGTAATTTTAGCTAATTCTTCAATTGATATTATTCTTC
ACGATACTTACTATGTAGTAGCTCATTTTCACTATGTTCTTTCTATAGGAGCTGTA
TTTGCAATTATAGCCGGAATTGTTCAATGATTCCCATTATTTACCGGACTAACCTT
AAATAATTTTTTTTTAAAAACCCAATTTATTACTATATTTTTAGGTGTAAATTTAACA
TTTTTTCCCCAACACTTTTTAGGGCTAAGAGGAATGCCTCGGCGATATTCGGATT
ATCCAGATGCTTTCACTATCTGAAATGCCATTTCATCTATTGGTTCCATTATTTCT
CTCACAAGAATTATCTTTTTTTTTATTTATCTTATGAGAAAGGCTTTCTTCCCAACG
TAAATCTTTGAGAACTTTAAATGTAACAACTTCAATTGAATGGTTCAAGCCCTACC
CCCTAGTG
MAC2
ATTATTAGACAAGAAAGGGGGAAAAAAGAAGCTTTTGGACCTTAGGAATAATTTA
TGCCATGATAGCTATCGGATTATTAGGATTCGTAGTTTGAGCTCACCATATATTT
ACAGTAGATAGATGTAGATACCCGAGCTTACTTCACTTCAGCAACTATAATTATT
GCGGTTCCAACTGGGATTAAAGTCTTTAGATGACTAGCCACCTTTCATGGAACT
CAAATTTTAAATAGACCAGTAACCCTGTGAGCTTTAGGTTTTGTATTTTTATTTAC
AGTAGGAGGATTAACAGGGGTAATTTTAGCTAATTCTTCAATTGATATTATTCTTC
ACGATACTTACTATGTAGTAGCTCATTTTCACTATGTTCTTTCTATAGGAGCTGTA
TTTGCAATTATAGCCGGAATTGTCCAATGATTCCCATTATTTACCGGACTAACCT
TAAATAATTTTTTTTTAAAAACCCAATTTATTACTATATTTTTAGGTGTAAATTTAAC
ATTTTTTCCCCAACACTTTTTAGGGCTAAGAGGAATGCCTCGGCGATATTCGGAT
TATCCAGATGCTTTCACTATCTGAAATGCCATTTCATCTATTGGTTCCATTATTTC
TCTCACAAGAATTATCTTTTTTTTTATTTATCTTATGAGAAAGGCTTTCTTCCCAAC
GTAAATCTTTGAGAACTTTAAATGTAACAACTTCAATTGAATGGTTCAAGCCCTAC
CCCCTAGTG
MAC4
ATTATTAGACAAGAAAGGGGGAAAAAAGAAGCTTTTGGACCTTAGGAATAATTTA
TGCCATGATAGCTATCGGATTATTAGGATTCGTAGTTTGAGCTCACCATATATTT
ACAGTAGATAGATGTAGATACCCGAGCTTACTTCACTTCAGCAACTATAATTATT
GCGGTTCCAACTGGGATTAAAGTCTTTAGATGACTAGCCACCTTTCATGGAACT
CAAATTTTAAATAGACCAGTAACCCTGTGAGCTTTAGGTTTTGTATTTTTATTTAC
AGTAGGAGGATTAACAGGGGTAATTTTAGCTAATTCTTCAATTGATATTATTCTTC
ACGATACTTACTATGTAGTAGCTCATTTTCACTATGTTCTTTCTATAGGAGCTGTA
TTTGCAATTATAGCCGGAATTGTCCAATGATTCCCATTATTTACCGGACTAACCT
TAAATAATTTTTTTTTAAAAACCCAATTTATTACTATATTTTTAGGTGTAAATTTAAC
ATTTTTTCCCCAACACTTTTTAGGGCTAAGAGGAATGCCTCGGCGATATTCGGAT
TATCCAGATGCTTTCACTATCTGAAATGCCATTTCATCTATTGGTTCCATTATTTC

TCTCACAAGAATTATCTTTTTTTTATTTATCTTATGAGAAAGGCTTTCTTCCCAAC
GTAAATCTTTGAGAACTTTAAATGTAACAACTTCAATTGAATGGTTCAAGCCCTAC
CCCCTAGTG
MAC6
ATTATTAGACAAGAAAGGGGGAAAAAAGAAGCTTTTGGACCTTAGGAATAATTTA
TGCCATGATAGCTATCGGATTATTAGGATTCGTAGTTTGAGCTCACCATATATTT
ACAGTAGATAGATGTAGATACCCGAGCTTACTTCACTTCAGCAACTATAATTATT
GCGGTTCCAACTGGGATTAAAGTCTTTAGATGACTAGCCACCTTTCATGGAACT
CAAATTTTAAATAGACCAGTAACCCTGTGAGCTTTAGGTTTTGTATTTTTATTTAC
AGTAGGAGGATTAACAGGGGTAATTTTAGCTAATTCTTCAATTGATATTATTCTTC
ACGATACTTACTATGTAGTAGCTCATTTTCACTATGTTCTTTCTATAGGAGCTGTA
TTTGCAATTATAGCCGGAATTGTCCAATGATTCCCATTATTTACCGGACTAACCT
TAAATAATTTTTTTTTAAAAACCCAATTTATTACTATATTTTTAGGTGTAAATTTAAC
ATTTTTTCCCCAACACTTTTTAGGGCTAAGAGGAATGCCTCGGCGATATTCGGAT
TATCCAGATGCTTTCACTATCTGAAATGCCATTTCATCTATTGGTTCCATTATTTC
TCTCACAAGAATTATCTTTTTTTTATTTATCTTATGAGAAAGGCTTTCTTCCCAAC
GTAAATCTTTGAGAACTTTAAATGTAACAACTTCAATTGAATGGTTCAAGCCCTAC
CCCCTAGTG
MAR2_1
ATTATTAGACAAGAAAGGGGGAAAAAAGAAGCTTTTGGACCTTAGGAATAATTTA
TGCCATGATAGCTATCGGATTATTAGGATTCGTAGTTTGAGCTCACCATATATTT
ACAGTAGATAGATGTAGATACCCGAGCTTACTTCACTTCAGCAACTATAATTATT
GCGGTTCCAACTGGGATTAAAGTCTTTAGATGACTAGCCACCTTTCATGGAACT
CAAATTTTAAATAGACCAGTAACCCTGTGAGCTTTAGGTTTTGTATTTTTATTTAC
AGTAGGAGGATTAACAGGGGTAATTTTAGCTAATTCTTCAATTGATATTATTCTTC
ACGATACTTACTATGTAGTAGCTCATTTTCACTATGTTCTTTCTATAGGAGCTGTA
TTTGCAATTATAGCCGGAATTGTCCAATGATTCCCATTATTTACCGGACTAACCT
TAAATAATTTTTTTTTAAAAACCCAATTTATTACTATATTTTTAGGTGTAAATTTAAC
ATTTTTTCCCCAACACTTTTTAGGGCTAAGAGGAATGCCTCGGCGATATTCGGAT
TATCCAGATGCTTTCACTATCTGAAATGCCATTTCATCTATTGGTTCCATTATTTC
TCTCACAAGAATTATCTTTTTTTTATTTATCTTATGAGAAAGGCTTTCTTCCCAAC
GTAAATCTTTGAGAACTTTAAATGTAACAACTTCAATTGAATGGTTCAAGCCCTAC
CCCCTAGTG
MAR2_2
ATTATTAGACAAGAAAGGGGGAAAAAAGAAGCTTTTGGACCTTAGGAATAATTTA
TGCCATGATAGCTATCGGATTATTAGGATTCGTAGTTTGAGCTCACCATATATTT
ACAGTAGATAGATGTAGATACCCGAGCTTACTTCACTTCAGCAACTATAATTATT
GCGGTTCCAACTGGGATTAAAGTCTTTAGATGACTAGCCACCTTTCATGGAACT
CAAATTTTAAATAGACCAGTAACCCTGTGAGCTTTAGGTTTTGTATTTTTATTTAC
AGTAGGAGGATTAACAGGGGTAATTTTAGCTAATTCTTCAATTGATATTATTCTTC
ACGATACTTACTATGTAGTAGCTCATTTTCACTATGTTCTTTCTATAGGAGCTGTA
TTTGCAATTATAGCCGGAATTGTCCAATGATTCCCATTATTTACCGGACTAACCT
TAAATAATTTTTTTTTAAAAACCCAATTTATTACTATATTTTTAGGTGTAAATTTAAC
ATTTTTTCCCCAACACTTTTTAGGGCTAAGAGGAATGCCTCGGCGATATTCGGAT
TATCCAGATGCTTTCACTATCTGAAATGCCATTTCATCTATTGGTTCCATTATTTC
TCTCACAAGAATTATCTTTTTTTTATTTATCTTATGAGAAAGGCTTTCTTCCCAAC
GTAAATCTTTGAGAACTTTAAATGTAACAACTTCAATTGAATGGTTCAAGCCCTAC
CCCCTAGTG
MAR3_1
ATTATTAGACAAGAAAGGGGGAAAAAAGAAGCTTTTGGACCTTAGGAATAATTTA

TGCCATGATAGCTATCGGATTATTAGGATTCGTAGTTTGAGCTCACCATATATTT
ACAGTAGATAGATGTAGATACCCGAGCTTACTTCACTTCAGCAACTATAATTATT
GCGGTTCCAACTGGGATTAAAGTCTTTAGATGACTAGCCACCTTTCATGGAACT
CAAATTTTAAATAGACCAGTAACCCTGTGAGCTTTAGGTTTTGTATTTTTATTTAC
AGTAGGAGGATTAACAGGGGTAATTTTAGCTAATTCTTCAATTGATATTATTCTTC
ACGATACTTACTATGTAGTAGCTCATTTTCACTATGTTCTTTCTATAGGAGCTGTA
TTTGCAATTATAGCCGGAATTGTCCAATGATTCCCATTATTTACCGGACTAACCT
TAAATAATTTTTTTTTAAAAACCCAATTTATTACTATATTTTTAGGTGTAAATTTAAC
ATTTTTTCCCCAACACTTTTTAGGGCTAAGAGGAATGCCTCGGCGATATTCGGAT
TATCCAGATGCTTTCACTATCTGAAATGCCATTTCATCTATTGGTTCCATTATTTC
TCTCACAAGAATTATCTTTTTTTTATTTATCTTATGAGAAAGGCTTTCTTCCCAAC
GTAAATCTTTGAGAACTTTAAATGTAACAACTTCAATTGAATGGTTCAAGCCCTAC
CCCCTAGTG
MAR3_2
ATTATTAGACAAGAAAGGGGGAAAAAAGAAGCTTTTGGACCTTAGGAATAATTTA
TGCCATGATAGCTATCGGATTATTAGGATTCGTAGTTTGAGCTCACCATATATTT
ACAGTAGATAGATGTAGATACCCGAGCTTACTTCACTTCAGCAACTATAATTATT
GCGGTTCCAACTGGGATTAAAGTCTTTAGATGACTAGCCACCTTTCATGGAACT
CAAATTTTAAATAGACCAGTAACCCTGTGAGCTTTAGGTTTTGTATTTTTATTTAC
AGTAGGAGGATTAACAGGGGTAATTTTAGCTAATTCTTCAATTGATATTATTCTTC
ACGATACTTACTATGTAGTAGCTCATTTTCACTATGTTCTTTCTATAGGAGCTGTA
TTTGCAATTATAGCCGGAATTGTCCAATGATTCCCATTATTTACCGGACTAACCT
TAAATAATTTTTTTTTAAAAACCCAATTTATTACTATATTTTTAGGTGTAAATTTAAC
ATTTTTTCCCCAACACTTTTTAGGGCTAAGAGGAATGCCTCGGCGATATTCGGAT
TATCCAGATGCTTTCACTATCTGAAATGCCATTTCATCTATTGGTTCCATTATTTC
TCTCACAAGAATTATCTTTTTTTTATTTATCTTATGAGAAAGGCTTTCTTCCCAAC
GTAAATCTTTGAGAACTTTAAATGTAACAACTTCAATTGAATGGTTCAAGCCCTAC
CCCCTAGTG
MOC1
ATTATTAGACAAGAAAGGGGGAAAAAAGAAGCTTTTGGACCTTAGGAATAATTTA
TGCCATGATAGCTATCGGATTATTAGGATTCGTAGTTTGAGCTCACCATATATTT
ACAGTAGATAGATGTAGATACCCGAGCTTACTTCACTTCAGCAACTATAATTATT
GCGGTTCCAACTGGGATTAAAGTCTTTAGATGACTAGCCACCTTTCATGGAACT
CAAATTTTAAATAGACCAGTAACCCTGTGAGCTTTAGGTTTTGTATTTTTATTTAC
AGTAGGAGGATTAACAGGGGTAATTTTAGCTAATTCTTCAATTGATATTATTCTTC
ACGATACTTACTATGTAGTAGCTCATTTTCACTATGTTCTTTCTATAGGAGCTGTA
TTTGCAATTATAGCCGGAATTGTCCAATGATTCCCATTATTTACCGGACTAACCT
TAAATAATTTTTTTTTAAAAACCCAATTTATTACTATATTTTTAGGTGTAAATTTAAC
ATTTTTTCCCCAACACTTTTTAGGGCTAAGAGGAATGCCTCGGCGATATTCGGAT
TATCCAGATGCTTTCACTATCTGAAATGCCATTTCATCTATTGGTTCCATTATTTC
TCTCACAAGAATTATCTTTTTTTTATTTATCTTATGAGAAAGGCTTTCTTCCCAAC
GTAAATCTTTGAGAACTTTAAATGTAACAACTTCAATTGAATGGTTCAAGCCCTAC
CCCCTAGTG
MOC2
ATTATTAGACAAGAAAGGGGGAAAAAAGAAGCTTTTGGACCTTAGGAATAATTTA
TGCCATGATAGCTATCGGATTATTAGGATTCGTAGTTTGAGCTCACCATATATTT
ACAGTAGATAGATGTAGATACCCGAGCTTACTTCACTTCAGCAACTATAATTATT
GCGGTTCCAACTGGGATTAAAGTCTTTAGATGACTAGCCACCTTTCATGGAACT
CAAATTTTAAATAGACCAGTAACCCTGTGAGCTTTAGGTTTTGTATTTTTATTTAC
AGTAGGAGGATTAACAGGGGTAATTTTAGCTAATTCTTCAATTGATATTATTCTTC

ACGATACTTACTATGTAGTAGCTCATTTTCACTATGTTCTTTCTATAGGAGCTGTA
TTTGCAATTATAGCCGGAATTGTCCAATGATTCCCATTATTTACCGGACTAACCT
TAAATAATTTTTTTTTAAAAACCCAATTTATTACTATATTTTTAGGTGTAAATTTAAC
ATTTTTTCCCCAACACTTTTTAGGGCTAAGAGGAATGCCTCGGCGATATTCGGAT
TATCCAGATGCTTTCACTATCTGAAATGCCATTTCATCTATTGGTTCCATTATTTC
TCTCACAAGAATTATCTTTTTTTTATTTATCTTATGAGAAAGGCTTTCTTCCCAAC
GTAAATCTTTGAGAACTTTAAATGTAACAACTTCAATTGAATGGTTCAAGCCCTAC
CCCCTAGTG
MOC2_0
ATTATTAGACAAGAAAGGGGGAAAAAAGAAGCTTTTGGACCTTAGGAATAATTTA
TGCCATGATAGCTATCGGATTATTAGGATTCGTAGTTTGAGCTCACCATATATTT
ACAGTAGATAGATGTAGATACCCGAGCTTACTTCACTTCAGCAACTATAATTATT
GCGGTTCCAACTGGGATTAAAGTCTTTAGATGACTAGCCACCTTTCATGGAACT
CAAATTTTAAATAGACCAGTAACCCTGTGAGCTTTAGGTTTTGTATTTTTATTTAC
AGTAGGAGGATTAACAGGGGTAATTTTAGCTAATTCTTCAATTGATATTATTCTTC
ACGATACTTACTATGTAGTAGCTCATTTTCACTATGTTCTTTCTATAGGAGCTGTA
TTTGCAATTATAGCCGGAATTGTCCAATGATTCCCATTATTTACCGGACTAACCT
TAAATAATTTTTTTTTAAAAACCCAATTTATTACTATATTTTTAGGTGTAAATTTAAC
ATTTTTTCCCCAACACTTTTTAGGGCTAAGAGGAATGCCTCGGCGATATTCGGAT
TATCCAGATGCTTTCACTATCTGAAATGCCATTTCATCTATTGGTTCCATTATTTC
TCTCACAAGAATTATCTTTTTTTTATTTATCTTATGAGAAAGGCTTTCTTCCCAAC
GTAAATCTTTGAGAACTTTAAATGTAACAACTTCAATTGAATGGTTCAAGCCCTAC
CCCCTAGTG
MOC2_1
ATTATTAGACAAGAAAGGGGGAAAAAAGAAGCTTTTGGACCTTAGGAATAATTTA
TGCCATGATAGCTATCGGATTATTAGGATTCGTAGTTTGAGCTCACCATATATTT
ACAGTAGATAGATGTAGATACCCGAGCTTACTTCACTTCAGCAACTATAATTATT
GCGGTTCCAACTGGGATTAAAGTCTTTAGATGACTAGCCACCTTTCATGGAACT
CAAATTTTAAATAGACCAGTAACCCTGTGAGCTTTAGGTTTTGTATTTTTATTTAC
AGTAGGAGGATTAACAGGGGTAATTTTAGCTAATTCTTCAATTGATATTATTCTTC
ACGATACTTACTATGTAGTAGCTCATTTTCACTATGTTCTTTCTATAGGAGCTGTA
TTTGCAATTATAGCCGGAATTGTCCAATGATTCCCATTATTTACCGGACTAACCT
TAAATAATTTTTTTTTAAAAACCCAATTTATTACTATATTTTTAGGTGTAAATTTAAC
ATTTTTTCCCCAACACTTTTTAGGGCTAAGAGGAATGCCTCGGCGATATTCGGAT
TATCCAGATGCTTTCACTATCTGAAATGCCATTTCATCTATTGGTTCCATTATTTC
TCTCACAAGAATTATCTTTTTTTTATTTATCTTATGAGAAAGGCTTTCTTCCCAAC
GTAAATCTTTGAGAACTTTAAATGTAACAACTTCAATTGAATGGTTCAAGCCCTAC
CCCCTAGTG
MOC3
ATTATTAGACAAGAAAGGGGGAAAAAAGAAGCTTTTGGACCTTAGGAATAATTTA
TGCCATGATAGCTATCGGATTATTAGGATTCGTAGTTTGAGCTCACCATATATTT
ACAGTAGATAGATGTAGATACCCGAGCTTACTTCACTTCAGCAACTATAATTATT
GCGGTTCCAACTGGGATTAAAGTCTTTAGATGACTAGCCACCTTTCATGGAACT
CAAATTTTAAATAGACCAGTAACCCTGTGAGCTTTAGGTTTTGTATTTTTATTTAC
AGTAGGAGGATTAACAGGGGTAATTTTAGCTAATTCTTCAATTGATATTATTCTTC
ACGATACTTACTATGTAGTAGCTCATTTTCACTATGTTCTTTCTATAGGAGCTGTA
TTTGCAATTATAGCCGGAATTGTCCAATGATTCCCATTATTTACCGGACTAACCT
TAAATAATTTTTTTTTAAAAACCCAATTTATTACTATATTTTTAGGTGTAAATTTAAC
ATTTTTTCCCCAACACTTTTTAGGGCTAAGAGGAATGCCTCGGCGATATTCGGAT
TATCCAGATGCTTTCACTATCTGAAATGCCATTTCATCTATTGGTTCCATTATTTC

TCTCACAAGAATTATCTTTTTTTTATTTATCTTATGAGAAAGGCTTTCTTCCCAAC
GTAAATCTTTGAGAACTTTAAATGTAACAACTTCAATTGAATGGTTCAAGCCCTAC
CCCCTAGTG
MOC4
ATTATTAGACAAGAAAGGGGGAAAAAAGAAGCTTTTGGACCTTAGGAATAATTTA
TGCCATGATAGCTATCGGATTATTAGGATTCGTAGTTTGAGCTCACCATATATTT
ACAGTAGATAGATGTAGATACCCGAGCTTACTTCACTTCAGCAACTATAATTATT
GCGGTTCCAACTGGGATTAAAGTCTTTAGATGACTAGCCACCTTTCATGGAACT
CAAATTTTAAATAGACCAGTAACCCTGTGAGCTTTAGGTTTTGTATTTTTATTTAC
AGTAGGAGGATTAACAGGGGTAATTTTAGCTAATTCTTCAATTGATATTATTCTTC
ACGATACTTACTATGTAGTAGCTCATTTTCACTATGTTCTTTCTATAGGAGCTGTA
TTTGCAATTATAGCCGGAATTGTCCAATGATTCCCATTATTTACCGGACTAACCT
TAAATAATTTTTTTTTAAAAACCCAATTTATTACTATATTTTTAGGTGTAAATTTAAC
ATTTTTTCCCCAACACTTTTTAGGGCTAAGAGGAATGCCTCGGCGATATTCGGAT
TATCCAGATGCTTTCACTATCTGAAATGCCATTTCATCTATTGGTTCCATTATTTC
TCTCACAAGAATTATCTTTTTTTTATTTATCTTATGAGAAAGGCTTTCTTCCCAAC
GTAAATCTTTGAGAACTTTAAATGTAACAACTTCAATTGAATGGTTCAAGCCCTAC
CCCCTAGTG
moc5
ATTATTAGACAAGAAAGGGGGAAAAAAGAAGCTTTTGGACCTTAGGAATAATTTA
TGCCATGATAGCTATCGGATTATTAGGATTCGTAGTTTGAGCTCACCATATATTT
ACAGTAGATAGATGTAGATACCCGAGCTTACTTCACTTCAGCAACTATAATTATT
GCGGTTCCAACTGGGATTAAAGTCTTTAGATGACTAGCCACCTTTCATGGAACT
CAAATTTTAAATAGACCAGTAACCCTGTGAGCTTTAGGTTTTGTATTTTTATTTAC
AGTAGGAGGATTAACAGGGGTAATTTTAGCTAATTCTTCAATTGATATTATTCTTC
ACGATACTTACTATGTAGTAGCTCATTTTCACTATGTTCTTTCTATAGGAGCTGTA
TTTGCAATTATAGCCGGAATTGTCCAATGATTCCCATTATTTACCGGACTAACCT
TAAATAATTTTTTTTTAAAAACCCAATTTATTACTATATTTTTAGGTGTAAATTTAAC
ATTTTTTCCCCAACACTTTTTAGGGCTAAGAGGAATGCCTCGGCGATATTCGGAT
TATCCAGATGCTTTCACTATCTGAAATGCCATTTCATCTATTGGTTCCATTATTTC
TCTCACAAGAATTATCTTTTTTTTATTTATCTTATGAGAAAGGCTTTCTTCCCAAC
GTAAATCTTTGAGAACTTTAAATGTAACAACTTCAATTGAATGGTTCAAGCCCTAC
CCCCTAGTG
pit1
ATTATTAGACAAGAAAGGGGGAAAAAAGAAGCTTTTGGACCTTAGGAATAATTTA
TGCCATGATAGCTATCGGATTATTAGGATTCGTAGTTTGAGCTCACCATATATTT
ACAGTAGATAGATGTAGATACCCGAGCTTACTTCACTTCAGCAACTATAATTATC
GCGGTTCCAACTGGGATTAAAGTCTTTAGGTGACTAGCCACCTTTCATGGAACT
CAAATTTTAAATAGACCAGTAACCCTGTGAGCTTTAGGTTTTGTATTTTTATTTAC
AGTAGGAGGATTAACAGGGGTAATTTTAGCTAATTCTTCAATTGATATTATTCTTC
ACGATACTTACTATGTAGTAGCTCATTTTCACTATGTTCTTTCTATAGGAGCTGTA
TTTGCAATTATAGCCGGAATTGTCCAATGATTCCCATTATTTACTGGACTAACCTT
AAATAATTTTTTTTTAAAAACCCAATTTATTACTATATTTTTAGGTGTAAATTTAACA
TTTTTTCCTCAACACTTTTTAGGGCTAAGAGGAATGCCTCGGCGATATTCGGATT
ATCCAGATGCTTTCACTATCTGAAATGCCATTTCATCTATTGGTTCCATTATTTCT
CTCACAAGAATTATCTTTTTTTTATTTATCTTATGAGAAAGGCTTTCTTCCCAACG
TAAATCTTTGAGAACTTTAAATGTAACAACTTCAATTGAATGGTTCAAGCCCTACC
CCCTAGTG
PIT1_1
ATTATTAGACAAGAAAGGGGGAAAAAAGAAGCTTTTGGACCTTAGGAATAATTTA

TGCCATGATAGCTATCGGATTATTAGGATTCGTAGTTTGAGCTCACCATATATTT
ACAGTAGATAGATGTAGATACCCGAGCTTACTTCACTTCAGCAACTATAATTATC
GCGGTTCCAACTGGGATTAAAGTCTTTAGGTGACTAGCCACCTTTCATGGAACT
CAAATTTTAAATAGACCAGTAACCCTGTGAGCTTTAGGTTTTGTATTTTTATTTAC
AGTAGGAGGATTAACAGGGGTAATTTTAGCTAATTCTTCAATTGATATTATTCTTC
ACGATACTTACTATGTAGTAGCTCATTTTCACTATGTTCTTTCTATAGGAGCTGTA
TTTGCAATTATAGCCGGAATTGTCCAATGATTCCCATTATTTACTGGACTAACCTT
AAATAATTTTTTTTTAAAAACCCAATTTATTACTATATTTTTAGGTGTAAATTTAACA
TTTTTTCCTCAACACTTTTTAGGGCTAAGAGGAATGCCTCGGCGATATTCGGATT
ATCCAGATGCTTTCACTATCTGAAATGCCATTTCATCTATTGGTTCCATTATTTCT
CTCACAAGAATTATCTTTTTTTTTATTTATCTTATGAGAAAGGCTTTCTTCCCAACG
TAAATCTTTGAGAACTTTAAATGTAACAACTTCAATTGAATGGTTCAAGCCCTACC
CCCTAGTG
PIT1_2
ATTATTAGACAAGAAAGGGGGAAAAAAGAAGCTTTTGGACCTTAGGAATAATTTA
TGCCATGATAGCTATCGGATTATTAGGATTCGTAGTTTGAGCTCACCATATATTT
ACAGTAGATAGATGTAGATACCCGAGCTTACTTCACTTCAGCAACTATAATTATT
GCGGTTCCAACTGGGATTAAAGTCTTTAGATGACTAGCCACCTTTCATGGAACT
CAAATTTTAAATAGACCAGTAACCCTGTGAGCTTTAGGTTTTGTATTTTTATTTAC
AGTAGGAGGATTAACAGGGGTAATTTTAGCTAATTCTTCAATTGATATTATTCTTC
ACGATACTTACTATGTAGTAGCTCATTTTCACTATGTTCTTTCTATAGGAGCTGTA
TTTGCAATTATAGCCGGAATTGTCCAATGATTCCCATTATTTACTGGACTAACCTT
AAATAATTTTTTTTTAAAAACCCAATTTATTACTATATTTTTAGGTGTAAATTTAACA
TTTTTTCCTCAACACTTTTTAGGGCTAAGAGGAATGCCTCGGCGATATTCGGATT
ATCCAGATGCTTTCACTATCTGAAATGCCATTTCATCTATTGGTTCCATTATTTCT
CTCACAAGAATTATCTTTTTTTTTATTTATCTTATGAGAAAGGCTTTCTTCCCAACG
TAAATCTTTGAGAACTTTAAATGTAACAACTTCAATTGAATGGTTCAAGCCCTACC
CCCTAGTG
pit2
ATTATTAGACAAGAAAGGGGGAAAAAAGAAGCTTTTGGACCTTAGGAATAATTTA
TGCCATGATAGCTATCGGATTATTAGGATTCGTAGTTTGAGCTCACCATATATTT
ACAGTAGATAGATGTAGATACCCGAGCTTACTTCACTTCAGCAACTATAATTATC
GCGGTTCCAACTGGGATTAAAGTCTTTAGGTGACTAGCCACCTTTCATGGAACT
CAAATTTTAAATAGACCAGTAACCCTGTGAGCTTTAGGTTTTGTATTTTTATTTAC
AGTAGGAGGATTAACAGGGGTAATTTTAGCTAATTCTTCAATTGATATTATTCTTC
ACGATACTTACTATGTAGTAGCTCATTTTCACTATGTTCTTTCTATAGGAGCTGTA
TTTGCAATTATAGCCGGAATTGTCCAATGATTCCCATTATTTACTGGACTAACCTT
AAATAATTTTTTTTTAAAAACCCAATTTATTACTATATTTTTAGGTGTAAATTTAACA
TTTTTTCCTCAACACTTTTTAGGGCTAAGAGGAATGCCTCGGCGATATTCGGATT
ATCCAGATGCTTTCACTATCTGAAATGCCATTTCATCTATTGGTTCCATTATTTCT
CTCACAAGAATTATCTTTTTTTTTATTTATCTTATGAGAAAGGCTTTCTTCCCAACG
TAAATCTTTGAGAACTTTAAATGTAACAACTTCAATTGAATGGTTCAAGCCCTACC
CCCTAGTG
pit4
ATTATTAGACAAGAAAGGGGGAAAAAAGAAGCTTTTGGACCTTAGGAATAATTTA
TGCCATGATAGCTATCGGATTATTAGGATTCGTAGTTTGAGCTCACCATATATTT
ACAGTAGATAGATGTAGATACCCGAGCTTACTTCACTTCAGCAACTATAATTATT
GCGGTTCCAACTGGGATTAAAGTCTTTAGATGACTAGCCACCTTTCATGGAACT
CAAATTTTAAATAGACCAGTAACCCTGTGAGCTTTAGGTTTTGTATTTTTATTTAC
AGTAGGAGGATTAACAGGGGTAATTTTAGCTAATTCTTCAATTGATATTATTCTTC

ACGATACTTACTATGTAGTAGCTCATTTTCACTATGTTCTTTCTATAGGAGCTGTA
TTTGCAATTATAGCCGGAATTGTCCAATGATTCCCATTATTTACTGGACTAACCTT
AAATAATTTTTTTTTAAAAACCCAATTTATTACTATATTTTTAGGTGTAAATTTAACA
TTTTTTCCTCAACACTTTTTAGGGCTAAGAGGAATGCCTCGGCGATATTCGGATT
ATCCAGATGCTTTCACTATCTGAAATGCCATTTCATCTATTGGTTCCATTATTTCT
CTCACAAGAATTATCTTTTTTTTATTTATCTTATGAGAAAGGCTTTCTTCCCAACG
TAAATCTTTGAGAACTTTAAATGTAACAACTTCAATTGAATGGTTCAAGCCCTACC
CCCTAGTG
RON1
ATTATTAGACAAGAAAGGGGGAAAAAAGAAGCTTTTGGACCTTAGGAATAATTTA
TGCCATGATAGCTATCGGATTATTAGGATTCGTAGTTTGAGCTCACCATATATTT
ACAGTAGATAGATGTAGATACCCGAGCTTACTTCACTTCAGCAACTATAATTATT
GCGGTTCCAACTGGGATTAAAGTCTTTAGATGACTAGCCACCTTTCATGGAACT
CAAATTTTAAATAGACCAGTAACCCTGTGAGCTTTAGGTTTTGTATTTTTATTTAC
AGTAGGAGGATTAACAGGGGTAATTTTAGCTAATTCTTCAATTGATATTATTCTTC
ACGATACTTACTATGTAGTAGCTCATTTTCACTATGTTCTTTCTATAGGAGCTGTA
TTTGCAATTATAGCCGGAATTGTCCAATGATTCCCATTATTTACCGGACTAACCT
TAAATAATTTTTTTTTAAAAACCCAATTTATTACTATATTTTTAGGTGTAAATTTAAC
ATTTTTTCCCCAACACTTTTTAGGGCTAAGAGGAATGCCTCGGCGATATTCGGAT
TATCCAGATGCTTTCACTATCTGAAATGCCATTTCATCTATTGGTTCCATTATTTC
TCTCACAAGAATTATCTTTTTTTTATTTATCTTATGAGAAAGGCTTTCTTCCCAAC
GTAAATCTTTGAGAACTTTAAATGTAACAACTTCAATTGAATGGTTCAAGCCCTAC
CCCCTAGTG
RON2
ATTATTAGACAAGAAAGGGGGAAAAAAGAAGCTTTTGGACCTTAGGAATAATTTA
TGCCATGATAGCTATCGGATTATTAGGATTCGTAGTTTGAGCTCACCATATATTT
ACAGTAGATAGATGTAGATACCCGAGCTTACTTCACTTCAGCAACTATAATTATT
GCGGTTCCAACTGGGATTAAAGTCTTTAGATGACTAGCCACCTTTCATGGAACT
CAAATTTTAAATAGACCAGTAACCCTGTGAGCTTTAGGTTTTGTATTTTTATTTAC
AGTAGGAGGATTAACAGGGGTAATTTTAGCTAATTCTTCAATTGATATTATTCTTC
ACGATACTTACTATGTAGTAGCTCATTTTCACTATGTTCTTTCTATAGGAGCTGTA
TTTGCAATTATAGCCGGAATTGTCCAATGATTCCCATTATTTACCGGACTAACCT
TAAATAATTTTTTTTTAAAAACCCAATTTATTACTATATTTTTAGGTGTAAATTTAAC
ATTTTTTCCCCAACACTTTTTAGGGCTAAGAGGAATGCCTCGGCGATATTCGGAT
TATCCAGATGCTTTCACTATCTGAAATGCCATTTCATCTATTGGTTCCATTATTTC
TCTCACAAGAATTATCTTTTTTTTATTTATCTTATGAGAAAGGCTTTCTTCCCAAC
GTAAATCTTTGAGAACTTTAAATGTAACAACTTCAATTGAATGGTTCAAGCCCTAC
CCCCTAGTG
ron3
ATTATTAGACAAGAAAGGGGGAAAAAAGAAGCTTTTGGACCTTAGGAATAATTTA
TGCCATGATAGCTATCGGATTATTAGGATTCGTAGTTTGAGCTCACCATATATTT
ACAGTAGATAGATGTAGATACCCGAGCTTACTTCACTTCAGCAACTATAATTATT
GCGGTTCCAACTGGGATTAAAGTCTTTAGATGACTAGCCACCTTTCATGGAACT
CAAATTTTAAATAGACCAGTAACCCTGTGAGCTTTAGGTTTTGTATTTTTATTTAC
AGTAGGAGGATTAACAGGGGTAATTTTAGCTAATTCTTCAATTGATATTATTCTTC
ACGATACTTACTATGTAGTAGCTCATTTTCACTATGTTCTTTCTATAGGAGCTGTA
TTTGCAATTATAGCCGGAATTGTCCAATGATTCCCATTATTTACCGGACTAACCT
TAAATAATTTTTTTTTAAAAACCCAATTTATTACTATATTTTTAGGTGTAAATTTAAC
ATTTTTTCCCCAACACTTTTTAGGGCTAAGAGGAATGCCTCGGCGATATTCGGAT
TATCCAGATGCTTTCACTATCTGAAATGCCATTTCATCTATTGGTTCCATTATTTC

TCTCACAAGAATTATCTTTTTTTTATTTATCTTATGAGAAAGGCTTTCTTCCCAAC
GTAAATCTTTGAGAACTTTAAATGTAACAACTTCAATTGAATGGTTCAAGCCCTAC
CCCCTAGTG
RON4
ATTATTAGACAAGAAAGGGGGAAAAAAGAAGCTTTTGGACCTTAGGAATAATTTA
TGCCATGATAGCTATCGGATTATTAGGATTCGTAGTTTGAGCTCACCATATATTT
ACAGTAGATAGATGTAGATACCCGAGCTTACTTCACTTCAGCAACTATAATTATT
GCGGTTCCAACTGGGATTAAAGTCTTTAGATGACTAGCCACCTTTCATGGAACT
CAAATTTTAAATAGACCAGTAACCCTGTGAGCTTTAGGTTTTGTATTTTTATTTAC
AGTAGGAGGATTAACAGGGGTAATTTTAGCTAATTCTTCAATTGATATTATTCTTC
ACGATACTTACTATGTAGTAGCTCATTTTCACTATGTTCTTTCTATAGGAGCTGTA
TTTGCAATTATAGCCGGAATTGTCCAATGATTCCCATTATTTACCGGACTAACCT
TAAATAATTTTTTTTTAAAAACCCAATTTATTACTATATTTTTAGGTGTAAATTTAAC
ATTTTTTCCCCAACACTTTTTAGGGCTAAGAGGAATGCCTCGGCGATATTCGGAT
TATCCAGATGCTTTCACTATCTGAAATGCCATTTCATCTATTGGTTCCATTATTTC
TCTCACAAGAATTATCTTTTTTTTATTTATCTTATGAGAAAGGCTTTCTTCCCAAC
GTAAATCTTTGAGAACTTTAAATGTAACAACTTCAATTGAATGGTTCAAGCCCTAC
CCCCTAGTG
RON7
ATTATTAGACAAGAAAGGGGGAAAAAAGAAGCTTTTGGACCTTAGGAATAATTTA
TGCCATGATAGCTATCGGATTATTAGGATTCGTAGTTTGAGCTCACCATATATTT
ACAGTAGATAGATGTAGATACCCGAGCTTACTTCACTTCAGCAACTATAATTATT
GCGGTTCCAACTGGGATTAAAGTCTTTAGATGACTAGCCACCTTTCATGGAACT
CAAATTTTAAATAGACCAGTAACCCTGTGAGCTTTAGGTTTTGTATTTTTATTTAC
AGTAGGAGGATTAACAGGGGTAATTTTAGCTAATTCTTCAATTGATATTATTCTTC
ACGATACTTACTATGTAGTAGCTCATTTTCACTATGTTCTTTCTATAGGAGCTGTA
TTTGCAATTATAGCCGGAATTGTCCAATGATTCCCATTATTTACCGGACTAACCT
TAAATAATTTTTTTTTAAAAACCCAATTTATTACTATATTTTTAGGTGTAAATTTAAC
ATTTTTTCCCCAACACTTTTTAGGGCTAAGAGGAATGCCTCGGCGATATTCGGAT
TATCCAGATGCTTTCACTATCTGAAATGCCATTTCATCTATTGGTTCCATTATTTC
TCTCACAAGAATTATCTTTTTTTTATTTATCTTATGAGAAAGGCTTTCTTCCCAAC
GTAAATCTTTGAGAACTTTAAATGTAACAACTTCAATTGAATGGTTCAAGCCCTAC
CCCCTAGTG
SGO1
ATTATTAGACAAGAAAGGGGGAAAAAAGAAGCTTTTGGACCTTAGGAATAATTTA
TGCCATGATAGCTATCGGATTATTAGGATTCGTAGTTTGAGCTCACCATATATTT
ACAGTAGATAGATGTAGATACCCGAGCTTACTTCACTTCAGCAACTATAATTATT
GCGGTTCCAACTGGGATTAAAGTCTTTAGATGACTAGCCACCTTTCATGGAACT
CAAATTTTAAATAGACCAGTAACCCTGTGAGCTTTAGGTTTTGTATTTTTATTTAC
AGTAGGAGGATTAACAGGGGTAATTTTAGCTAATTCTTCAATTGATATTATTCTTC
ACGATACTTACTATGTAGTAGCTCATTTTCACTATGTTCTTTCTATAGGAGCTGTA
TTTGCAATTATAGCCGGAATTGTCCAATGATTCCCATTATTTACCGGACTAACCT
TAAATAATTTTTTTTTAAAAACCCAATTTATTACTATATTTTTAGGTGTAAATTTAAC
ATTTTTTCCCCAACACTTTTTAGGGCTAAGAGGAATGCCTCGGCGATATTCGGAT
TATCCAGATGCTTTCACTATCTGAAATGCCATTTCATCTATTGGTTCCATTATTTC
TCTCACAAGAATTATCTTTTTTTTATTTATCTTATGAGAAAGGCTTTCTTCCCAAC
GTAAATCTTTGAGAACTTTAAATGTAACAACTTCAATTGAATGGTTCAAGCCCTAC
CCCCTAGTG
SGO2
ATTATTAGACAAGAAAGGGGGAAAAAAGAAGCTTTTGGACCTTAGGAATAATTTA

TGCCATGATAGCTATCGGATTATTAGGATTCGTAGTTTGAGCTCACCATATATTT
ACAGTAGATAGATGTAGATACCCGAGCTTACTTCACTTCAGCAACTATAATTATT
GCGGTTCCAACTGGGATTAAAGTCTTTAGATGACTAGCCACCTTTCATGGAACT
CAAATTTTAAATAGACCAGTAACCCTGTGAGCTTTAGGTTTTGTATTTTTATTTAC
AGTAGGAGGATTAACAGGGGTAATTTTAGCTAATTCTTCAATTGATATTATTCTTC
ACGATACTTACTATGTAGTAGCTCATTTTCACTATGTTCTTTCTATAGGAGCTGTA
TTTGCAATTATAGCCGGAATTGTCCAATGATTCCCATTATTTACCGGACTAACCT
TAAATAATTTTTTTTTAAAAACCCAATTTATTACTATATTTTTAGGTGTAAATTTAAC
ATTTTTTCCCCAACACTTTTTAGGGCTAAGAGGAATGCCTCGGCGATATTCGGAT
TATCCAGATGCTTTCACTATCTGAAATGCCATTTCATCTATTGGTTCCATTATTTC
TCTCACAAGAATTATCTTTTTTTTATTTATCTTATGAGAAAGGCTTTCTTCCCAAC
GTAAATCTTTGAGAACTTTAAATGTAACAACTTCAATTGAATGGTTCAAGCCCTAC
CCCCTAGTG
SJA12
ATTATTAGACAAGAAAGGGGGAAAAAAGAAGCTTTTGGACCTTAGGAATAATTTA
TGCCATGATAGCTATCGGATTATTAGGATTCGTAGTTTGAGCTCACCATATATTT
ACAGTAGATAGATGTAGATACCCGAGCTTACTTCACTTCAGCAACTATAATTATT
GCGGTTCCAACTGGGATTAAAGTCTTTAGATGACTAGCCACCTTTCATGGAACT
CAAATTTTAAATAGACCAGTAACCCTGTGAGCTTTAGGTTTTGTATTTTTATTTAC
AGTAGGAGGATTAACAGGGGTAATTTTAGCTAATTCTTCAATTGATATTATTCTTC
ACGATACTTACTATGTAGTAGCTCATTTTCACTATGTTCTTTCTATAGGAGCTGTA
TTTGCAATTATAGCCGGAATTGTCCAATGATTCCCATTATTTACCGGACTAACCT
TAAATAATTTTTTTTTAAAAACCCAATTTATTACTATATTTTTAGGTGTAAATTTAAC
ATTTTTTCCCCAACACTTTTTAGGGCTAAGAGGAATGCCTCGGCGATATTCGGAT
TATCCAGATGCTTTCACTATCTGAAATGCCATTTCATCTATTGGTTCCATTATTTC
TCTCACAAGAATTATCTTTTTTTTATTTATCTTATGAGAAAGGCTTTCTTCCCAAC
GTAAATCTTTGAGAACTTTAAATGTAACAACTTCAATTGAATGGTTCAAGCCCTAC
CCCCTAGTG
SJA13
ATTATTAGACAAGAAAGGGGGAAAAAAGAAGCTTTTGGACCTTAGGAATAATTTA
TGCCATGATAGCTATCGGATTATTAGGATTCGTAGTTTGAGCTCACCATATATTT
ACAGTAGATAGATGTAGATACCCGAGCTTACTTCACTTCAGCAACTATAATTATT
GCGGTTCCAACTGGGATTAAAGTCTTTAGATGACTAGCCACCTTTCATGGAACT
CAAATTTTAAATAGACCAGTAACCCTGTGAGCTTTAGGTTTTGTATTTTTATTTAC
AGTAGGAGGATTAACAGGGGTAATTTTAGCTAATTCTTCAATTGATATTATTCTTC
ACGATACTTACTATGTAGTAGCTCATTTTCACTATGTTCTTTCTATAGGAGCTGTA
TTTGCAATTATAGCCGGAATTGTCCAATGATTCCCATTATTTACCGGACTAACCT
TAAATAATTTTTTTTTAAAAACCCAATTTATTACTATATTTTTAGGTGTAAATTTAAC
ATTTTTTCCCCAACACTTTTTAGGGCTAAGAGGAATGCCTCGGCGATATTCGGAT
TATCCAGATGCTTTCACTATCTGAAATGCCATTTCATCTATTGGTTCCATTATTTC
TCTCACAAGAATTATCTTTTTTTTATTTATCTTATGAGAAAGGCTTTCTTCCCAAC
GTAAATCTTTGAGAACTTTAAATGTAACAACTTCAATTGAATGGTTCAAGCCCTAC
CCCCTAGTG
SJA14
ATTATTAGACAAGAAAGGGGGAAAAAAGAAGCTTTTGGACCTTAGGAATAATTTA
TGCCATGATAGCTATCGGATTATTAGGATTCGTAGTTTGAGCTCACCATATATTT
ACAGTAGATAGATGTAGATACCCGAGCTTACTTCACTTCAGCAACTATAATTATT
GCGGTTCCAACTGGGATTAAAGTCTTTAGATGACTAGCCACCTTTCATGGAACT
CAAATTTTAAATAGACCAGTAACCCTGTGAGCTTTAGGTTTTGTATTTTTATTTAC
AGTAGGAGGATTAACAGGGGTAATTTTAGCTAATTCTTCAATTGATATTATTCTTC

ACGATACTTACTATGTAGTAGCTCATTTTCACTATGTTCTTTCTATAGGAGCTGTA
TTTGCAATTATAGCCGGAATTGTCCAATGATTCCCATTATTTACCGGACTAACCT
TAAATAATTTTTTTTTAAAAACCCAATTTATTACTATATTTTTAGGTGTAAATTTAAC
ATTTTTTCCCCAACACTTTTTAGGGCTAAGAGGAATGCCTCGGCGATATTCGGAT
TATCCAGATGCTTTCACTATCTGAAATGCCATTTCATCTATTGGTTCCATTATTTC
TCTCACAAGAATTATCTTTTTTTTATTTATCTTATGAGAAAGGCTTTCTTCCCAAC
GTAAATCTTTGAGAACTTTAAATGTAACAACTTCAATTGAATGGTTCAAGCCCTAC
CCCCTAGTG
SJA17
ATTATTAGACAAGAAAGGGGGAAAAAAGAAGCTTTTGGACCTTAGGAATAATTTA
TGCCATGATAGCTATCGGATTATTAGGATTCGTAGTTTGAGCTCACCATATATTT
ACAGTAGATAGATGTAGATACCCGAGCTTACTTCACTTCAGCAACTATAATTATT
GCGGTTCCAACTGGGATTAAAGTCTTTAGATGACTAGCCACCTTTCATGGAACT
CAAATTTTAAATAGACCAGTAACCCTGTGAGCTTTAGGTTTTGTATTTTTATTTAC
AGTAGGAGGATTAACAGGGGTAATTTTAGCTAATTCTTCAATTGATATTATTCTTC
ACGATACTTACTATGTAGTAGCTCATTTTCACTATGTTCTTTCTATAGGAGCTGTA
TTTGCAATTATAGCCGGAATTGTCCAATGATTCCCATTATTTACCGGACTAACCT
TAAATAATTTTTTTTTAAAAACCCAATTTATTACTATATTTTTAGGTGTAAATTTAAC
ATTTTTTCCCCAACACTTTTTAGGGCTAAGAGGAATGCCTCGGCGATATTCGGAT
TATCCAGATGCTTTCACTATCTGAAATGCCATTTCATCTATTGGTTCCATTATTTC
TCTCACAAGAATTATCTTTTTTTTATTTATCTTATGAGAAAGGCTTTCTTCCCAAC
GTAAATCTTTGAGAACTTTAAATGTAACAACTTCAATTGAATGGTTCAAGCCCTAC
CCCCTAGTG
SJA3
ATTATTAGACAAGAAAGGGGGAAAAAAGAAGCTTTTGGACCTTAGGAATAATTTA
TGCCATGATAGCTATCGGATTATTAGGATTCGTAGTTTGAGCTCACCATATATTT
ACAGTAGATAGATGTAGATACCCGAGCTTACTTCACTTCAGCAACTATAATTATT
GCGGTTCCAACTGGGATTAAAGTCTTTAGATGACTAGCCACCTTTCATGGAACT
CAAATTTTAAATAGACCAGTAACCCTGTGAGCTTTAGGTTTTGTATTTTTATTTAC
AGTAGGAGGATTAACAGGGGTAATTTTAGCTAATTCTTCAATTGATATTATTCTTC
ACGATACTTACTATGTAGTAGCTCATTTTCACTATGTTCTTTCTATAGGAGCTGTA
TTTGCAATTATAGCCGGAATTGTCCAATGATTCCCATTATTTACCGGACTAACCT
TAAATAATTTTTTTTTAAAAACCCAATTTATTACTATATTTTTAGGTGTAAATTTAAC
ATTTTTTCCCCAACACTTTTTAGGGCTAAGAGGAATGCCTCGGCGATATTCGGAT
TATCCAGATGCTTTCACTATCTGAAATGCCATTTCATCTATTGGTTCCATTATTTC
TCTCACAAGAATTATCTTTTTTTTATTTATCTTATGAGAAAGGCTTTCTTCCCAAC
GTAAATCTTTGAGAACTTTAAATGTAACAACTTCAATTGAATGGTTCAAGCCCTAC
CCCCTAGTG
SJA4
ATTATTAGACAAGAAAGGGGGAAAAAAGAAGCTTTTGGACCTTAGGAATAATTTA
TGCCATGATAGCTATCGGATTATTAGGATTCGTAGTTTGAGCTCACCATATATTT
ACAGTAGATAGATGTAGATACCCGAGCTTACTTCACTTCAGCAACTATAATTATT
GCGGTTCCAACTGGGATTAAAGTCTTTAGATGACTAGCCACCTTTCATGGAACT
CAAATTTTAAATAGACCAGTAACCCTGTGAGCTTTAGGTTTTGTATTTTTATTTAC
AGTAGGAGGATTAACAGGGGTAATTTTAGCTAATTCTTCAATTGATATTATTCTTC
ACGATACTTACTATGTAGTAGCTCATTTTCACTATGTTCTTTCTATAGGAGCTGTA
TTTGCAATTATAGCCGGAATTGTCCAATGATTCCCATTATTTACCGGACTAACCT
TAAATAATTTTTTTTTAAAAACCCAATTTATTACTATATTTTTAGGTGTAAATTTAAC
ATTTTTTCCCCAACACTTTTTAGGGCTAAGAGGAATGCCTCGGCGATATTCGGAT
TATCCAGATGCTTTCACTATCTGAAATGCCATTTCATCTATTGGTTCCATTATTTC

TCTCACAAGAATTATCTTTTTTTTATTTATCTTATGAGAAAGGCTTTCTTCCCAAC
GTAAATCTTTGAGAACTTTAAATGTAACAACTTCAATTGAATGGTTCAAGCCCTAC
CCCCTAGTG
SJA5
ATTATTAGACAAGAAAGGGGGAAAAAAGAAGCTTTTGGACCTTAGGAATAATTTA
TGCCATGATAGCTATCGGATTATTAGGATTCGTAGTTTGAGCTCACCATATATTT
ACAGTAGATAGATGTAGATACCCGAGCTTACTTCACTTCAGCAACTATAATTATT
GCGGTTCCAACTGGGATTAAAGTCTTTAGATGACTAGCCACCTTTCATGGAACT
CAAATTTTAAATAGACCAGTAACCCTGTGAGCTTTAGGTTTTGTATTTTTATTTAC
AGTAGGAGGATTAACAGGGGTAATTTTAGCTAATTCTTCAATTGATATTATTCTTC
ACGATACTTACTATGTAGTAGCTCATTTTCACTATGTTCTTTCTATAGGAGCTGTA
TTTGCAATTATAGCCGGAATTGTCCAATGATTCCCATTATTTACCGGACTAACCT
TAAATAATTTTTTTTTAAAAACCCAATTTATTACTATATTTTTAGGTGTAAATTTAAC
ATTTTTTCCCCAACACTTTTTAGGGCTAAGAGGAATGCCTCGGCGATATTCGGAT
TATCCAGATGCTTTCACTATCTGAAATGCCATTTCATCTATTGGTTCCATTATTTC
TCTCACAAGAATTATCTTTTTTTTATTTATCTTATGAGAAAGGCTTTCTTCCCAAC
GTAAATCTTTGAGAACTTTAAATGTAACAACTTCAATTGAATGGTTCAAGCCCTAC
CCCCTAGTG
SMA1
ATTATTAGACAAGAAAGGGGGAAAAAAGAAGCTTTTGGACCTTAGGAATAATTTA
TGCCATGATAGCTATCGGATTATTAGGATTCGTAGTTTGAGCTCACCATATATTT
ACAGTAGATAGATGTAGATACCCGAGCTTACTTCACTTCAGCAACTATAATTATT
GCGGTTCCAACTGGGATTAAAGTCTTTAGATGACTAGCCACCTTTCATGGAACT
CAAATTTTAAATAGACCAGTAACCCTGTGAGCTTTAGGTTTTGTATTTTTATTTAC
AGTAGGAGGATTAACAGGGGTAATTTTAGCTAATTCTTCAATTGATATTATTCTTC
ACGATACTTACTATGTAGTAGCTCATTTTCACTATGTTCTTTCTATAGGAGCTGTA
TTTGCAATTATAGCCGGAATTGTCCAATGATTCCCATTATTTACCGGACTAACCT
TAAATAATTTTTTTTTAAAAACCCAATTTATTACTATATTTTTAGGTGTAAATTTAAC
ATTTTTTCCCCAACACTTTTTAGGGCTAAGAGGAATGCCTCGGCGATATTCGGAT
TATCCAGATGCTTTCACTATCTGAAATGCCATTTCATCTATTGGTTCCATTATTTC
TCTCACAAGAATTATCTTTTTTTTATTTATCTTATGAGAAAGGCTTTCTTCCCAAC
GTAAATCTTTGAGAACTTTAAATGTAACAACTTCAATTGAATGGTTCAAGCCCTAC
CCCCTAGTG
SMA10
ATTATTAGACAAGAAAGGGGGAAAAAAGAAGCTTTTGGACCTTAGGAATAATTTA
TGCCATGATAGCTATCGGATTATTAGGATTCGTAGTTTGAGCTCACCATATATTT
ACAGTAGATAGATGTAGATACCCGAGCTTACTTCACTTCAGCAACTATAATTATT
GCGGTTCCAACTGGGATTAAAGTCTTTAGATGACTAGCCACCTTTCATGGAACT
CAAATTTTAAATAGACCAGTAACCCTGTGAGCTTTAGGTTTTGTATTTTTATTTAC
AGTAGGAGGATTAACAGGGGTAATTTTAGCTAATTCTTCAATTGATATTATTCTTC
ACGATACTTACTATGTAGTAGCTCATTTTCACTATGTTCTTTCTATAGGAGCTGTA
TTTGCAATTATAGCCGGAATTGTCCAATGATTCCCATTATTTACCGGACTAACCT
TAAATAATTTTTTTTTAAAAACCCAATTTATTACTATATTTTTAGGTGTAAATTTAAC
ATTTTTTCCCCAACACTTTTTAGGGCTAAGAGGAATGCCTCGGCGATATTCGGAT
TATCCAGATGCTTTCACTATCTGAAATGCCATTTCATCTATTGGTTCCATTATTTC
TCTCACAAGAATTATCTTTTTTTTATTTATCTTATGAGAAAGGCTTTCTTCCCAAC
GTAAATCTTTGAGAACTTTAAATGTAACAACTTCAATTGAATGGTTCAAGCCCTAC
CCCCTAGTG
SMA11
ATTATTAGACAAGAAAGGGGGAAAAAAGAAGCTTTTGGACCTTAGGAATAATTTA

TGCCATGATAGCTATCGGATTATTAGGATTCGTAGTTTGAGCTCACCATATATTT
ACAGTAGATAGATGTAGATACCCGAGCTTACTTCACTTCAGCAACTATAATTATT
GCGGTTCCAACTGGGATTAAAGTCTTTAGATGACTAGCCACCTTTCATGGAACT
CAAATTTTAAATAGACCAGTAACCCTGTGAGCTTTAGGTTTTGTATTTTTATTTAC
AGTAGGAGGATTAACAGGGGTAATTTTAGCTAATTCTTCAATTGATATTATTCTTC
ACGATACTTACTATGTAGTAGCTCATTTTCACTATGTTCTTTCTATAGGAGCTGTA
TTTGCAATTATAGCCGGAATTGTCCAATGATTCCCATTATTTACCGGACTAACCT
TAAATAATTTTTTTTTAAAAACCCAATTTATTACTATATTTTTAGGTGTAAATTTAAC
ATTTTTTCCCCAACACTTTTTAGGGCTAAGAGGAATGCCTCGGCGATATTCGGAT
TATCCAGATGCTTTCACTATCTGAAATGCCATTTCATCTATTGGTTCCATTATTTC
TCTCACAAGAATTATCTTTTTTTTATTTATCTTATGAGAAAGGCTTTCTTCCCAAC
GTAAATCTTTGAGAACTTTAAATGTAACAACTTCAATTGAATGGTTCAAGCCCTAC
CCCCTAGTG
SMA12
ATTATTAGACAAGAAAGGGGGAAAAAAGAAGCTTTTGGACCTTAGGAATAATTTA
TGCCATGATAGCTATCGGATTATTAGGATTCGTAGTTTGAGCTCACCATATATTT
ACAGTAGATAGATGTAGATACCCGAGCTTACTTCACTTCAGCAACTATAATTATT
GCGGTTCCAACTGGGATTAAAGTCTTTAGATGACTAGCCACCTTTCATGGAACT
CAAATTTTAAATAGACCAGTAACCCTGTGAGCTTTAGGTTTTGTATTTTTATTTAC
AGTAGGAGGATTAACAGGGGTAATTTTAGCTAATTCTTCAATTGATATTATTCTTC
ACGATACTTACTATGTAGTAGCTCATTTTCACTATGTTCTTTCTATAGGAGCTGTA
TTTGCAATTATAGCCGGAATTGTCCAATGATTCCCATTATTTACCGGACTAACCT
TAAATAATTTTTTTTTAAAAACCCAATTTATTACTATATTTTTAGGTGTAAATTTAAC
ATTTTTTCCCCAACACTTTTTAGGGCTAAGAGGAATGCCTCGGCGATATTCGGAT
TATCCAGATGCTTTCACTATCTGAAATGCCATTTCATCTATTGGTTCCATTATTTC
TCTCACAAGAATTATCTTTTTTTTATTTATCTTATGAGAAAGGCTTTCTTCCCAAC
GTAAATCTTTGAGAACTTTAAATGTAACAACTTCAATTGAATGGTTCAAGCCCTAC
CCCCTAGTG
SMA13
ATTATTAGACAAGAAAGGGGGAAAAAAGAAGCTTTTGGACCTTAGGAATAATTTA
TGCCATGATAGCTATCGGATTATTAGGATTCGTAGTTTGAGCTCACCATATATTT
ACAGTAGATAGATGTAGATACCCGAGCTTACTTCACTTCAGCAACTATAATTATT
GCGGTTCCAACTGGGATTAAAGTCTTTAGATGACTAGCCACCTTTCATGGAACT
CAAATTTTAAATAGACCAGTAACCCTGTGAGCTTTAGGTTTTGTATTTTTATTTAC
AGTAGGAGGATTAACAGGGGTAATTTTAGCTAATTCTTCAATTGATATTATTCTTC
ACGATACTTACTATGTAGTAGCTCATTTTCACTATGTTCTTTCTATAGGAGCTGTA
TTTGCAATTATAGCCGGAATTGTCCAATGATTCCCATTATTTACCGGACTAACCT
TAAATAATTTTTTTTTAAAAACCCAATTTATTACTATATTTTTAGGTGTAAATTTAAC
ATTTTTTCCCCAACACTTTTTAGGGCTAAGAGGAATGCCTCGGCGATATTCGGAT
TATCCAGATGCTTTCACTATCTGAAATGCCATTTCATCTATTGGTTCCATTATTTC
TCTCACAAGAATTATCTTTTTTTTATTTATCTTATGAGAAAGGCTTTCTTCCCAAC
GTAAATCTTTGAGAACTTTAAATGTAACAACTTCAATTGAATGGTTCAAGCCCTAC
CCCCTAGTG
SMA14
ATTATTAGACAAGAAAGGGGGAAAAAAGAAGCTTTTGGACCTTAGGAATAATTTA
TGCCATGATAGCTATCGGATTATTAGGATTCGTAGTTTGAGCTCACCATATATTT
ACAGTAGATAGATGTAGATACCCGAGCTTACTTCACTTCAGCAACTATAATTATT
GCGGTTCCAACTGGGATTAAAGTCTTTAGATGACTAGCCACCTTTCATGGAACT
CAAATTTTAAATAGACCAGTAACCCTGTGAGCTTTAGGTTTTGTATTTTTATTTAC
AGTAGGAGGATTAACAGGGGTAATTTTAGCTAATTCTTCAATTGATATTATTCTTC

ACGATACTTACTATGTAGTAGCTCATTTTCACTATGTTCCTTCTATAGGAGCTGTA
TTTGCAATTATAGCCGGAATTGTCCAATGATTCCCATTATTTACCGGACTAACCT
TAAATAATTTTTTTTTAAAAACCCAATTTATTACTATATTTTTAGGTGTAAATTTAAC
ATTTTTTCCCCAACACTTTTTAGGGCTAAGAGGAATGCCTCGGCGATATTCGGAT
TATCCAGATGCTTTCACTATCTGAAATGCCATTTCATCTATTGGTTCCATTATTTC
TCTCACAAGAATTATCTTTTTTTTTATTTATCTTATGAGAAAGGCTTTCTTCCCAAC
GTAAATCTTTGAGAACTTTAAATGTAACAACTTCAATTGAATGGTTCAAGCCCTAC
CCCCTAGTG
SMA6
ATTATTAGACAAGAAAGGGGGAAAAAAGAAGCTTTTGGACCTTAGGAATAATTTA
TGCCATGATAGCTATCGGATTATTAGGATTCGTAGTTTGAGCTCACCATATATTT
ACAGTAGATAGATGTAGATACCCGAGCTTACTTCACTTCAGCAACTATAATTATT
GCGGTTCCAACTGGGATTAAAGTCTTTAGATGACTAGCCACCTTTCATGGAACT
CAAATTTTAAATAGACCAGTAACCCTGTGAGCTTTAGGTTTTGTATTTTTATTTAC
AGTAGGAGGATTAACAGGGGTAATTTTAGCTAATTCTTCAATTGATATTATTCTTC
ACGATACTTACTATGTAGTAGCTCATTTTCACTATGTTCTTTCTATAGGAGCTGTA
TTTGCAATTATAGCCGGAATTGTCCAATGATTCCCATTATTTACCGGACTAACCT
TAAATAATTTTTTTTTAAAAACCCAATTTATTACTATATTTTTAGGTGTAAATTTAAC
ATTTTTTCCCCAACACTTTTTAGGGCTAAGAGGAATGCCTCGGCGATATTCGGAT
TATCCAGATGCTTTCACTATCTGAAATGCCATTTCATCTATTGGTTCCATTATTTC
TCTCACAAGAATTATCTTTTTTTTTATTTATCTTATGAGAAAGGCTTTCTTCCCAAC
GTAAATCTTTGAGAACTTTAAATGTAACAACTTCAATTGAATGGTTCAAGCCCTAC
CCCCTAGTG
SMI1
ATTATTAGACAAGAAAGGGGGAAAAAAGAAGCTTTTGGACCTTAGGAATAATTTA
TGCCATGATAGCTATCGGATTATTAGGATTCGTAGTTTGAGCTCACCATATATTT
ACAGTAGATAGATGTAGATACCCGAGCTTACTTCACTTCAGCAACTATAATTATT
GCGGTTCCAACTGGGATTAAAGTCTTTAGATGACTAGCCACCTTTCATGGAACT
CAAATTTTAAATAGACCAGTAACCCTGTGAGCTTTAGGTTTTGTATTTTTATTTAC
AGTAGGAGGATTAACAGGGGTAATTTTAGCTAATTCTTCAATTGATATTATTCTTC
ACGATACTTACTATGTAGTAGCTCATTTTCACTATGTTCTTTCTATAGGAGCTGTA
TTTGCAATTATAGCCGGAATTGTCCAATGATTCCCATTATTTACCGGACTAACCT
TAAATAATTTTTTTTTAAAAACCCAATTTATTACTATATTTTTAGGTGTAAATTTAAC
ATTTTTTCCCCAACACTTTTTAGGGCTAAGAGGAATGCCTCGGCGATATTCGGAT
TATCCAGATGCTTTCACTATCTGAAATGCCATTTCATCTATTGGTTCCATTATTTC
TCTCACAAGAATTATCTTTTTTTTTATTTATCTTATGAGAAAGGCTTTCTTCCCAAC
GTAAATCTTTGAGAACTTTAAATGTAACAACTTCAATTGAATGGTTCAAGCCCTAC
CCCCTAGTG
SMI2
ATTATTAGACAAGAAAGGGGGAAAAAAGAAGCTTTTGGACCTTAGGAATAATTTA
TGCCATGATAGCTATCGGATTATTAGGATTCGTAGTTTGAGCTCACCATATATTT
ACAGTAGATAGATGTAGATACCCGAGCTTACTTCACTTCAGCAACTATAATTATT
GCGGTTCCAACTGGGATTAAAGTCTTTAGATGACTAGCCACCTTTCATGGAACT
CAAATTTTAAATAGACCAGTAACCCTGTGAGCTTTAGGTTTTGTATTTTTATTTAC
AGTAGGAGGATTAACAGGGGTAATTTTAGCTAATTCTTCAATTGATATTATTCTTC
ACGATACTTACTATGTAGTAGCTCATTTTCACTATGTTCTTTCTATAGGAGCTGTA
TTTGCAATTATAGCCGGAATTGTCCAATGATTCCCATTATTTACCGGACTAACCT
TAAATAATTTTTTTTTAAAAACCCAATTTATTACTATATTTTTAGGTGTAAATTTAAC
ATTTTTTCCCCAACACTTTTTAGGGCTAAGAGGAATGCCTCGGCGATATTCGGAT
TATCCAGATGCTTTCACTATCTGAAATGCCATTTCATCTATTGGTTCCATTATTTC

TCTCACAAGAATTATCTTTTTTTTATTTATCTTATGAGAAAGGCTTTCTTCCCAAC
GTAAATCTTTGAGAACTTTAAATGTAACAACTTCAATTGAATGGTTCAAGCCCTAC
CCCCTAGTG
sob1
ATTATTAGACAAGAAAGGGGGAAAAAAGAAGCTTTTGGACCTTAGGAATAATTTA
TGCCATGATAGCTATCGGATTATTAGGATTCGTAGTTTGAGCTCACCATATATTT
ACAGTAGATAGATGTAGATACCCGAGCTTACTTCACTTCAGCAACTATAATTATT
GCGGTTCCAACTGGGATTAAAGTCTTTAGATGACTAGCCACCTTTCATGGAACT
CAAATTTTAAATAGACCAGTAACCCTGTGAGCTTTAGGTTTTGTATTTTTATTTAC
AGTAGGAGGATTAACGGGGGTAATTTTAGCTAATTCTTCAATTGATATTATTCTTC
ACGATACTTACTATGTAGTAGCTCATTTTCACTATGTTCTTTCTATAGGAGCTGTA
TTTGCAATTATAGCCGGAATTGTCCAATGATTCCCATTATTTACCGGACTAACCT
TAAATAATTTTTTTTTAAAAACCCAATTTATTACTATATTTTTAGGTGTAAATTTAAC
ATTTTTTCCCCAACACTTTTTAGGGCTAAGAGGAATGCCTCGGCGATATTCGGAT
TATCCAGATGCTTTCACTATCTGAAATGCCATTTCATCTATTGGTTCCATTATTTC
TCTCACAAGAATTATCTTTTTTTTATTTATCTTATGAGAAAGGCTTTCTTCCCAAC
GTAAATCTTTGAGAACTTTAAATGTAACAACTTCAATTGAATGGTTCAAGCCCTAC
CCCCTAGTG
SOB10
ATTATTAGACAAGAAAGGGGGAAAAAAGAAGCTTTTGGACCTTAGGAATAATTTA
TGCCATGATAGCTATCGGATTATTAGGATTCGTAGTTTGAGCTCACCATATATTT
ACAGTAGATAGATGTAGATACCCGAGCTTACTTCACTTCAGCAACTATAATTATC
GCGGTTCCAACTGGGATTAAAGTCTTTAGGTGACTAGCCACCTTTCATGGAACT
CAAATTTTAAATAGACCAGTAACCCTGTGAGCTTTAGGTTTTGTATTTTTATTTAC
AGTAGGAGGATTAACAGGGGTAATTTTAGCTAATTCTTCAATTGATATTATTCTTC
ACGATACTTACTATGTAGTAGCTCATTTTCACTATGTTCTTTCTATAGGAGCTGTA
TTTGCAATTATAGCCGGAATTGTCCAATGATTCCCATTATTTACTGGACTAACCTT
AAATAATTTTTTTTTAAAAACCCAATTTATTACTATATTTTTAGGTGTAAATTTAACA
TTTTTTCCTCAACACTTTTTAGGGCTAAGAGGGATGCCTCGGCGATATTCGGATT
ATCCAGATGCTTTCACTATCTGAAATGCCATTTCATCTATTGGTTCCATTATTTCT
CTCACAAGAATTATCTTTTTTTTATTTATCTTATGAGAAAGGCTTTCTTCCCAACG
TAAATCTTTGAGAACTTTAAATGTAACAACTTCAATTGAATGGTTCAAGCCCTACC
CCCTAGTG
SOB12
ATTATTAGACAAGAAAGGGGGAAAAAAGAAGCTTTTGGACCTTAGGAATAATTTA
TGCCATGATAGCTATCGGATTATTAGGATTCGTAGTTTGAGCTCACCATATATTT
ACAGTAGATAGATGTAGATACCCGAGCTTACTTCACTTCAGCAACTATAATTATC
GCGGTTCCAACTGGGATTAAAGTCTTTAGGTGACTAGCCACCTTTCATGGAACT
CAAATTTTAAATAGACCAGTAACCCTGTGAGCTTTAGGTTTTGTATTTTTATTTAC
AGTAGGAGGATTAACAGGGGTAATTTTAGCTAATTCTTCAATTGATATTATTCTTC
ACGATACTTACTATGTAGTAGCTCATTTTCACTATGTTCTTTCTATAGGAGCTGTA
TTTGCAATTATAGCCGGAATTGTCCAATGATTCCCATTATTTACTGGACTAACCTT
AAATAATTTTTTTTTAAAAACCCAATTTATTACTATATTTTTAGGTGTAAATTTAACA
TTTTTTCCTCAACACTTTTTAGGGCTAAGAGGAATGCCTCGGCGATATTCGGATT
ATCCAGATGCTTTCACTATCTGAAATGCCATTTCATCTATTGGTTCCATTATTTCT
CTCACAAGAATTATCTTTTTTTTATTTATCTTATGAGAAAGGCTTTCTTCCCAACG
TAAATCTTTGAGAACTTTAAATGTAACAACTTCAATTGAATGGTTCAAGCCCTACC
CCCTAGTG
SOB1_2
ATTATTAGACAAGAAAGGGGGAAAAAAGAAGCTTTTGGACCTTAGGAATAATTTA

TGCCATGATAGCTATCGGATTATTAGGATTCGTAGTTTGAGCTCACCATATATTT
ACAGTAGATAGATGTAGATACCCGAGCTTACTTCACTTCAGCAACTATAATTATC
GCGGTTCCAACTGGGATTAAAGTCTTTAGGTGACTAGCCACCTTTCATGGAACT
CAAATTTTAAATAGACCAGTAACCCTGTGAGCTTTAGGTTTTGTATTTTTATTTAC
AGTAGGAGGATTAACAGGGGTAATTTTAGCTAATTCTTCAATTGATATTATTCTTC
ACGATACTTACTATGTAGTAGCTCATTTTCACTATGTTCTTTCTATAGGAGCTGTA
TTTGCAATTATAGCCGGAATTGTCCAATGATTCCCATTATTTACTGGACTAACCTT
AAATAATTTTTTTTTAAAAACCCAATTTATTACTATATTTTTAGGTGTAAATTTAACA
TTTTTTCCTCAACACTTTTTAGGGCTAAGAGGAATGCCTCGGCGATATTCGGATT
ATCCAGATGCTTTCACTATCTGAAATGCCATTTCATCTATTGGTTCCATTATTTCT
CTCACAAGAATTATCTTTTTTTTTATTTATCTTATGAGAAAGGCTTTCTTCCCAACG
TAAATCTTTGAGAACTTTAAATGTAACAACTTCAATTGAATGGTTCAAGCCCTACC
CCCTAGTG
SOB2
ATTATTAGACAAGAAAGGGGGAAAAAAGAAGCTTTTGGACCTTAGGAATAATTTA
TGCCATGATAGCTATCGGATTATTAGGATTCGTAGTTTGAGCTCACCATATATTT
ACAGTAGATAGATGTAGATACCCGAGCTTACTTCACTTCAGCAACTATAATTATT
GCGGTTCCAACTGGGATTAAAGTCTTTAGATGACTAGCCACCTTTCATGGAACT
CAAATTTTAAATAGACCAGTAACCCTGTGAGCTTTAGGTTTTGTATTTTTATTTAC
AGTAGGAGGATTAACGGGGGTAATTTTAGCTAATTCTTCAATTGATATTATTCTTC
ACGATACTTACTATGTAGTAGCTCATTTTCACTATGTTCTTTCTATAGGAGCTGTA
TTTGCAATTATAGCCGGAATTGTCCAATGATTCCCATTATTTACCGGACTAACCT
TAAATAATTTTTTTTTAAAAACCCAATTTATTACTATATTTTTAGGTGTAAATTTAAC
ATTTTTTCCCCAACACTTTTTAGGGCTAAGAGGAATGCCTCGGCGATATTCGGAT
TATCCAGATGCTTTCACTATCTGAAATGCCATTTCATCTATTGGTTCCATTATTTC
TCTCACAAGAATTATCTTTTTTTTTATTTATCTTATGAGAAAGGCTTTCTTCCCAAC
GTAAATCTTTGAGAACTTTAAATGTAACAACTTCAATTGAATGGTTCAAGCCCTAC
CCCCTAGTG
SOB2_1
ATTATTAGACAAGAAAGGGGGAAAAAAGAAGCTTTTGGACCTTAGGAATAATTTA
TGCCATGATAGCTATCGGATTATTAGGATTCGTAGTTTGAGCTCACCATATATTT
ACAGTAGATAGATGTAGATACCCGAGCTTACTTCACTTCAGCAACTATAATTATC
GCGGTTCCAACTGGGATTAAAGTCTTTAGGTGACTAGCCACCTTTCATGGAACT
CAAATTTTAAATAGACCAGTAACCCTGTGAGCTTTAGGTTTTGTATTTTTATTTAC
AGTAGGAGGATTAACAGGGGTAATTTTAGCTAATTCTTCAATTGATATTATTCTTC
ACGATACTTACTATGTAGTAGCTCATTTTCACTATGTTCTTTCTATAGGAGCTGTA
TTTGCAATTATAGCCGGAATTGTCCAATGATTCCCATTATTTACTGGACTAACCTT
AAATAATTTTTTTTTAAAAACCCAATTTATTACTATATTTTTAGGTGTAAATTTAACA
TTTTTTCCTCAACACTTTTTAGGGCTAAGAGGAATGCCTCGGCGATATTCGGATT
ATCCAGATGCTTTCACTATCTGAAATGCCATTTCATCTATTGGTTCCATTATTTCT
CTCACAAGAATTATCTTTTTTTTTATTTATCTTATGAGAAAGGCTTTCTTCCCAACG
TAAATCTTTGAGAACTTTAAATGTAACAACTTCAATTGAATGGTTCAAGCCCTACC
CCCTAGTG
TAP1
ATTATTAGACAAGAAAGGGGGAAAAAAGAAGCTTTTGGACCTTAGGAATAATTTA
TGCCATGATAGCTATCGGATTATTAGGATTCGTAGTTTGAGCTCACCATATATTT
ACAGTAGATAGATGTAGATACCCGAGCTTACTTCACTTCAGCAACTATAATTATT
GCGGTTCCAACTGGGATTAAAGTCTTTAGATGACTAGCCACCTTTCATGGAACT
CAAATTTTAAATAGACCAGTAACCCTGTGAGCTTTAGGTTTTGTATTTTTATTTAC
AGTAGGAGGATTAACAGGGGTAATTTTAGCTAATTCTTCAATTGATATTATTCTTC

ACGATACTTACTATGTAGTAGCTCATTTTCACTATGTTCTTTCTATAGGAGCTGTA
TTTGCAATTATAGCCGGAATTGTCCAATGATTCCCATTATTTACCGGACTAACCT
TAAATAATTTTTTTTTAAAAACCCAATTTATTACTATATTTTTAGGTGTAAATTTAAC
ATTTTTTCCCCAACACTTTTTAGGGCTAAGAGGAATGCCTCGGCGATATTCGGAT
TATCCAGATGCTTTCACTATCTGAAATGCCATTTCATCTATTGGTTCCATTATTTC
TCTCACAAGAATTATCTTTTTTTTATTTATCTTATGAGAAAGGCTTTCTTCCCAAC
GTAAATCTTTGAGAACTTTAAATGTAACAACTTCAATTGAATGGTTCAAGCCCTAC
CCCCTAGTG
TAP2
ATTATTAGACAAGAAAGGGGGAAAAAAGAAGCTTTTGGACCTTAGGAATAATTTA
TGCCATGATAGCTATCGGATTATTAGGATTCGTAGTTTGAGCTCACCATATATTT
ACAGTAGATAGATGTAGATACCCGAGCTTACTTCACTTCAGCAACTATAATTATT
GCGGTTCCAACTGGGATTAAAGTCTTTAGATGACTAGCCACCTTTCATGGAACT
CAAATTTTAAATAGACCAGTAACCCTGTGAGCTTTAGGTTTTGTATTTTTATTTAC
AGTAGGAGGATTAACAGGGGTAATTTTAGCTAATTCTTCAATTGATATTATTCTTC
ACGATACTTACTATGTAGTAGCTCATTTTCACTATGTTCTTTCTATAGGAGCTGTA
TTTGCAATTATAGCCGGAATTGTCCAATGATTCCCATTATTTACCGGACTAACCT
TAAATAATTTTTTTTTAAAAACCCAATTTATTACTATATTTTTAGGTGTAAATTTAAC
ATTTTTTCCCCAACACTTTTTAGGGCTAAGAGGAATGCCTCGGCGATATTCGGAT
TATCCAGATGCTTTCACTATCTGAAATGCCATTTCATCTATTGGTTCCATTATTTC
TCTCACAAGAATTATCTTTTTTTTATTTATCTTATGAGAAAGGCTTTCTTCCCAAC
GTAAATCTTTGAGAACTTTAAATGTAACAACTTCAATTGAATGGTTCAAGCCCTAC
CCCCTAGTG
tol1
ATTATTAGACAAGAAAGGGGGAAAAAAGAAGCTTTTGGACCTTAGGAATAATTTA
TGCCATGATAGCTATCGGATTATTAGGATTCGTAGTTTGAGCTCACCATATATTT
ACAGTAGATAGATGTAGATACCCGAGCTTACTTCACTTCAGCAACTATAATTATT
GCGGTTCCAACTGGGATTAAAGTCTTTAGATGACTAGCCACCTTTCATGGAACT
CAAATTTTAAATAGACCAGTAACCCTGTGAGCTTTAGGTTTTGTATTTTTATTTAC
AGTAGGAGGATTAACAGGGGTAATTTTAGCTAATTCTTCAATTGATATTATTCTTC
ACGATACTTACTATGTAGTAGCTCATTTTCACTATGTTCTTTCTATAGGAGCTGTA
TTTGCAATTATAGCCGGAATTGTCCAATGATTCCCATTATTTACCGGACTAACCT
TAAATAATTTTTTTTTAAAAACCCAATTTATTACTATATTTTTAGGTGTAAATTTAAC
ATTTTTTCCCCAACACTTTTTAGGGCTAAGAGGAATGCCTCGGCGATATTCGGAT
TATCCAGATGCTTTCACTATCTGAAATGCCATTTCATCTATTGGTTCCATTATTTC
TCTCACAAGAATTATCTTTTTTTTATTTATCTTATGAGAAAGGCTTTCTTCCCAAC
GTAAATCTTTGAGAACTTTAAATGTAACAACTTCAATTGAATGGTTCAAGCCCTAC
CCCCTAGTG
TOL1_1
ATTATTAGACAAGAAAGGGGGAAAAAAGAAGCTTTTGGACCTTAGGAATAATTTA
TGCCATGATAGCTATCGGATTATTAGGATTCGTAGTTTGAGCTCACCATATATTT
ACAGTAGATAGATGTAGATACCCGAGCTTACTTCACTTCAGCAACTATAATTATT
GCGGTTCCAACTGGGATTAAAGTCTTTAGATGACTAGCCACCTTTCATGGAACT
CAAATTTTAAATAGACCAGTAACCCTGTGAGCTTTAGGTTTTGTATTTTTATTTAC
AGTAGGAGGATTAACAGGGGTAATTTTAGCTAATTCTTCAATTGATATTATTCTTC
ACGATACTTACTATGTAGTAGCTCATTTTCACTATGTTCTTTCTATAGGAGCTGTA
TTTGCAATTATAGCCGGAATTGTCCAATGATTCCCATTATTTACCGGACTAACCT
TAAATAATTTTTTTTTAAAAACCCAATTTATTACTATATTTTTAGGTGTAAATTTAAC
ATTTTTTCCCCAACACTTTTTAGGGCTAAGAGGAATGCCTCGGCGATATTCGGAT
TATCCAGATGCTTTCACTATCTGAAATGCCATTTCATCTATTGGTTCCATTATTTC

TCTCACAAGAATTATCTTTTTTTTATTTATCTTATGAGAAAGGCTTTCTTCCCAAC
GTAAATCTTTGAGAACTTTAAATGTAACAACTTCAATTGAATGGTTCAAGCCCTAC
CCCCTAGTG
TOL5
ATTATTAGACAAGAAAGGGGGAAAAAAGAAGCTTTTGGACCTTAGGAATAATTTA
TGCCATGATAGCTATCGGATTATTAGGATTCGTAGTTTGAGCTCACCATATATTT
ACAGTAGATAGATGTAGATACCCGAGCTTACTTCACTTCAGCAACTATAATTATT
GCGGTTCCAACTGGGATTAAAGTCTTTAGATGACTAGCCACCTTTCATGGAACT
CAAATTTTAAATAGACCAGTAACCCTGTGAGCTTTAGGTTTTGTATTTTTATTTAC
AGTAGGAGGATTAACAGGGGTAATTTTAGCTAATTCTTCAATTGATATTATTCTTC
ACGATACTTACTATGTAGTAGCTCATTTTCACTATGTTCTTTCTATAGGAGCTGTA
TTTGCAATTATAGCCGGAATTGTCCAATGATTCCCATTATTTACCGGACTAACCT
TAAATAATTTTTTTTTAAAAACCCAATTTATTACTATATTTTTAGGTGTAAATTTAAC
ATTTTTTCCCCAACACTTTTTAGGGCTAAGAGGAATGCCTCGGCGATATTCGGAT
TATCCAGATGCTTTCACTATCTGAAATGCCATTTCATCTATTGGTTCCATTATTTC
TCTCACAAGAATTATCTTTTTTTTATTTATCTTATGAGAAAGGCTTTCTTCCCAAC
GTAAATCTTTGAGAACTTTAAATGTAACAACTTCAATTGAATGGTTCAAGCCCTAC
CCCCTAGTG
VIC10
ATTATTAGACAAGAAAGGGGGAAAAAAGAAGCTTTTGGACCTTAGGAATAATTTA
TGCCATGATAGCTATCGGATTATTAGGATTCGTAGTTTGAGCTCACCATATATTT
ACAGTAGATAGATGTAGATACCCGAGCTTACTTCACTTCAGCAACTATAATTATT
GCGGTTCCAACTGGGATTAAAGTCTTTAGATGACTAGCCACCTTTCATGGAACT
CAAATTTTAAATAGACCAGTAACCCTGTGAGCTTTAGGTTTTGTATTTTTATTTAC
AGTAGGAGGATTAACAGGGGTAATTTTAGCTAATTCTTCAATTGATATTATTCTTC
ACGATACTTACTATGTAGTAGCTCATTTTCACTATGTTCTTTCTATAGGAGCTGTA
TTTGCAATTATAGCCGGAATTGTCCAATGATTCCCATTATTTACCGGACTAACCT
TAAATAATTTTTTTTTAAAAACCCAATTTATTACTATATTTTTAGGTGTAAATTTAAC
ATTTTTTCCCCAACACTTTTTAGGGCTAAGAGGAATGCCTCGGCGATATTCGGAT
TATCCAGATGCTTTCACTATCTGAAATGCCATTTCATCTATTGGTTCCATTATTTC
TCTCACAAGAATTATCTTTTTTTTATTTATCTTATGAGAAAGGCTTTCTTCCCAAC
GTAAATCTTTGAGAACTTTAAATGTAACAACTTCAATTGAATGGTTCAAGCCCTAC
CCCCTAGTG
XAN4
ATTATTAGACAAGAAAGGGGGAAAAAAGAAGCTTTTGGACCTTAGGAATAATTTA
TGCCATGATAGCTATCGGATTATTAGGATTCGTAGTTTGAGCTCACCATATATTT
ACAGTAGATAGATGTAGATACCCGAGCTTACTTCACTTCAGCAACTATAATTATC
GCGGTTCCAACTGGGATTAAAGTCTTTAGGTGACTAGCCACCTTTCATGGAACT
CAAATTTTAAATAGACCAGTAACCCTGTGAGCTTTAGGTTTTGTATTTTTATTTAC
AGTAGGAGGATTAACAGGGGTAATTTTAGCTAATTCTTCAATTGATATTATTCTTC
ACGATACTTACTATGTAGTAGCTCATTTTCACTATGTTCTTTCTATAGGAGCTGTA
TTTGCAATTATAGCCGGAATTGTCCAATGATTCCCATTATTTACTGGACTAACCTT
AAATAATTTTTTTTTAAAAACCCAATTTATTACTATATTTTTAGGTGTAAATTTAACA
TTTTTTCCTCAACACTTTTTAGGGCTAAGAGGAATGCCTCGGCGATATTCGGATT
ATCCAGATGCTTTCGCTATCTGAAATGCCATTTCATCTATTGGTTCCATTATTTCT
CTCACAAGAATTATCTTTTTTTTATTTATCTTATGAGAAAGGCTTTCTTCCCAACG
TAAATCTTTGAGAACTTTAAATGTAACAACTTCAATTGAATGGTTCAAGCCCTACC
CCCTAGTG
XAX2
ATTATTAGACAAGAAAGGGGGAAAAAAGAAGCTTTTGGACCTTAGGAATAATTTA

TGCCATGATAGCTATCGGATTATTAGGATTCGTAGTTTGAGCTCACCATATATTT
ACAGTAGATAGATGTAGATACCCGAGCTTACTTCACTTCAGCAACTATAATTATT
GCGGTTCCAACTGGGATTAAAGTCTTTAGATGACTAGCCACCTTTCATGGAACT
CAAATTTTAAATAGACCAGTAACCCTGTGAGCTTTAGGTTTTGTATTTTTATTTAC
AGTAGGAGGATTAACAGGGGTAATTTTAGCTAATTCTTCAATTGATATTATTCTTC
ACGATACTTACTATGTAGTAGCTCATTTTCACTATGTTCTTTCTATAGGAGCTGTA
TTTGCAATTATAGCCGGAATTGTCCAATGATTCCCATTATTTACCGGACTAACCT
TAAATAATTTTTTTTTAAAAACCCAATTTATTACTATATTTTTAGGTGTAAATTTAAC
ATTTTTTCCCCAACACTTTTTAGGGCTAAGAGGAATGCCTCGGCGATATTCGGAT
TATCCAGATGCTTTCACTATCTGAAATGCCATTTCATCTATTGGTTCCATTATTTC
TCTCACAAGAATTATCTTTTTTTTATTTATCTTATGAGAAAGGCTTTCTTCCCAAC
GTAAATCTTTGAGAACTTTAAATGTAACAACTTCAATTGAATGGTTCAAGCCCTAC
CCCCTAGTG
MEX_SFP
ATTATTAGACAAGAAAGGGGGAAAAAAGAAGCTTTTGGACCTTAGGAATAATTTA
TGCCATAATAGCTATCGGATTATTAGGATTCGTAGTTTGAGCTCACCATATATTTA
CAGTAGATAGATGTAGATACCCGAGCTTACTTCACTTCAGCAACTATAATTATTG
CGGTTCCAACTGGGATTAAAGTCTTTAGATGACTAGCCACCTTTCATGGAACTCA
AATTTTAAATAGACCAGTAACCCTGTGAGCTTTAGGTTTTGTATTTTTATTTACAG
TAGGAGGATTAACAGGGGTAATTTTAGCTAATTCTTCAATTGATATTATTCTTCAC
GATACTTACTATGTAGTAGCTCATTTTCACTATGTTCTTTCTATAGGAGCTGTATT
TGCAATTATAGCCGGAATTGTCCAATGATTCCCATTATTTACTGGACTAACCTTA
AATAATTTTTTTTTAAAAACCCAATTTATTACTATATTTTTAGGTGTAAATTTAACAT
TTTTTCCTCAACACTTTTTAGGGCTAAGAGGAATGCCTCGGCGATATTCGGATTA
TCCAGATGCTTTCACTATCTGAAATGCCATTTCATCTATTGGTTCCATTATTTCTC
TCACAAGAATTATCTTTTTTTTATTTATCTTATGAGAAAGGCTTTCTTCCCAACGT
AAATCTTTGAGAACTTTAAATGTAACAACTTCAATTGAATGGTTCAAGCCCTACC
CCCTAGTG
MEX_COE_2
ATTATTAGACAAGAAAGGGGGAAAAAAGAACCTTTTGGACCTTAGGAATAATTTA
TGCCATAATAGCTATCGGATTATTAGGATTCGTAGTTTGAGCTCACCATATATTTA
CAGTAGATAGATGTAGATACCCGAGCTTACTTCACTTCAGCAACTATAATTATTG
CGGTTCCAACTGGGATTAAAGTCTTTAGATGACTAGCCACCTTTCATGGAACTCA
AATTTTAAATAGACCAGTAACCCTGTGAGCTTTAGGTTTTGTATTTTTATTTACAG
TAGGAGGATTAACAGGGGTAATTTTAGCTAATTCTTCAATTGATATTATTCTTCAC
GATACTTACTATGTAGTAGCTCATTTTCACTATGTTCTTTCTATAGGAGCTGTATT
TGCAATTATAGCCGGAATTGTCCAATGATTCCCATTATTTACTGGACTAACCTTA
AATAATTTTTTTTTAAAAACCCAATTTATTACTATATTTTTAGGTGTAAATTTAACAT
TTTTTCCTCAACACTTTTTAGGGCTAAGAGGAATGCCTCGGCGATATTCGGATTA
TCCAGATGCTTTCACTATCTGAAATGCCATTTCATCTATTGGTTCCATTATTTCTC
TCACAAGAATTATCTTTTTTTTATTTATCTTATGAGAAAGGCTTTCTTCCCAACGT
AAATCTTTGAGAACTTTAAATGTAACAACTTCAATTGAATGGTTCAAGCCCTACC
CCCTAGTG
MEX_SAG_5
ATTATTAGACAAGAAAGGGGGAAAAAAGAAGCTTTTGGACCTTAGGAATAATTTA
TGCCATAATAGCTATCGGATTATTAGGATTCGTAGTTTGAGCTCACCATATATTTA
CAGTAGATAGATGTAGATACCCGAGCTTACTTCACTTCAGCAACTATAATTATTG
CGGTTCCAACTGGGATTAAAGTCTTTAGATGACTAGCCACCTTTCATGGAACTCA
AATTTTAAATAGACCAGTAACCCTGTGAGCTTTAGGTTTTGTATTTTTATTTACAG
TAGGAGGATTAACAGGGGTAATTTTAGCTAATTCTTCAATTGATATTATTCTTCAC

GATACTTACTATGTAGTAGCTCATTTTCACTATGTTCTTTCTATAGGAGCTGTATT
TGCAATTATAGCCGGAATTGTCCAATGATTCCCATTATTTACTGGGCTAACCTTA
AATAATTTTTTTTTAAAAACCCAATTTATTACTATATTTTTAGGTGTAAATTTAACAT
TTTTTCCTCAACACTTTTTAGGGCTAAGAGGAATGCCTCGGCGATATTCGGATTA
TCCAGATGCTTTCACTATCTGAAATGCCATTTCATCTATTGGTTCCATTATTTCTC
TCACAAGAATTATCTTTTTTTTATTTATCTTATGAGAAAGGCTTTCTTCCCAACGT
AAATCTTTGAGAACTTTAAATGTAACAACTTCAATTGAATGGTTCAAGCCCTACC
CCCTAGTG
MEX_SCA
ATTATTAGACAAGAAAGGGGGAAAAAAGAAGCTTTTGGACCTTAGGAATAATTTA
TGCCATAATAGCTATCGGATTATTAGGATTCGTAGTTTGAGCTCACCATATATTTA
CAGTAGATAGATGTAGATACCCGAGCTTACTTCACTTCAGCACCTATGATTATTG
CGGTTCCAACTGGGATTAAAGTCTTTAGATGACTAGCCACCTTTCATGGAACTCA
AATTTTAAATAGACCAGTAACCCTGTGAGCTTTAGGTTTTGTATTTTTATTTACAG
TAGGAGGATTAACAGGGGTAATTTTAGCTAATTCTTCAATTGATATTATTCTTCAC
GATACTTACTATGTAGTAGCTCATTTTCACTATGTTCTTTCTATAGGAGCTGTATT
TGCAATTATAGCCGGAATTGTCCAATGATTCCCATTATTTACTGGACTAACCTTA
AATAATTTTTTTTTAAAAACCCAATTTATTACTATATTTTTAGGTGTAAATTTAACAT
TTTTTCCTCAACACTTTTTAGGGCTAAGAGGAATGCCTCGGCGATATTCGGATTA
TCCAGATGCTTTCACTATCTGAAATGCCATTTCATCTATTGGTTCCATTATTTCTC
TCACAAGAATTATCTTTTTTTTATTTATCTTATGAGAAAGGCTTTCTTCCCAACGT
AAATCTTTGAGAACTTTAAATGTAACAACTTCAATTGAATGGTTCAAGCCCTACC
CCCTAGTG
MEX_SJS_2
ATTATTAGACAAGAAAGGGGGAAAAAAGAACCTTTTGGACCTTAGGAATAATTTA
TGCCATAATAGCTATCGGATTATTAGGATTCGTAGTTTGAGCTCACCATATATTTA
CAGTAGATAGATGTAGATACCCGAGCTTACTTCACTTCAGCAACTATAATTATTG
CGGTTCCAACTGGAATTAAAGTCTTTAGATGACTAGCCACCTTTCATGGAACTCA
AATTTTAAATAGACCAGTAACCCTGTGAGCTTTAGGTTTTGTATTTTTATTTACAG
TAGGAGGATTAACAGGGGTAATTTTAGCTAATTCTTCAATTGATATTATTCTTCAC
GATACTTACTATGTAGTAGCTCATTTTCACTATGTTCTTTCTATAGGAGCTGTATT
TGCAATTATAGCCGGAATTGTCCAATGATTCCCATTATTTACTGGACTAACCTTA
AATAATTTTTTTTTAAAAACCCAATTTATTACTATATTTTTAGGTGTAAATTTAACAT
TTTTTCCTCAACACTTTTTAGGGCTAAGAGGAATGCCTCGGCGATATTCGGATTA
TCCAGATGCTTTCACTATCTGAAATGCCATTTCATCTATTGGTTCCATTATTTCTC
TCACAAGAATTATCTTTTTTTTATTTATCTTATGAGAAAGGCTTTCTTCCCAACGT
AAATCTTTGAGAACTTTAAATGTAACAACTTCAATTGAATGGTTCAAGCCCTACC
CCCTAGTG
MEX_STO
ATTATTAGACAAGAAAGGGGGAAAAAAGAACCTTTTGGACCTTAGGAATAATTTA
TGCCATAATAGCTATCGGATTATTAGGATTCGTAGTTTGAGCTCACCATATATTTA
CAGTAGATAGATGTAGATACCCGAGCTTACTTCACTTCAGCAACTATAATTATTG
CGGTTCCAACTGGGATTAAAGTCTTTAGATGACTAGCCACCTTTCATGGAACTCA
AATTTTAAATAGACCAGTAACCCTGTGAGCTTTAGGTTTTGTATTTTTATTTACAG
TAGGAGGATTAACAGGGGTAATTTTAGCTAATTCTTCAATTGATATTATTCTTCAC
GATACTTACTATGTAGTAGCTCATTTTCACTATGTTCTTTCTATAGGAGCTGTATT
TGCAATTATAGCCGGAATTGTCCAATGATTCCCATTATTTACTGGACTAACCTTA
AATAATTTTTTTTTAAAAACCCAATTTATTACTATATTTTTAGGTGTAAATTTAACAT
TTTTTCCTCAACACTTTTTAGGGCTAAGAGGAATGCCTCGGCGATATTCGGATTA
TCCAGATGCTTTCACTATCTGAAATGCCATTTCATCTATTGGTTCCATTATTTCTC

TCACAAGAATTATCTTTTTTTTATTTATCTTATGAGAAAGGCTTTCTTCCCAACGT
AAATCTTTGAGAACTTTAAATGTAACAACTTCAATTGAATGGTTCAAGCCCTACC
CCCTAGTG
MEX_TLA_1
ATTATTAGACAAGAAAGGGGGAAAAAAGAAGCTTTTGGACCTTAGGAATAATTTA
TGCCATAATAGCTATCGGATTATTAGGATTCGTAGTTTGAGCTCACCATATATTTA
CAGTAGATAGATGTAGATACCCGAGCTTACTTCACTTCAGCACCTATGATTATTG
CGGTTCCAACTGGGATTAAAGTCTTTAGATGACTAGCCACCTTTCATGGAACTCA
AATTTTAAATAGACCAGTAACCCTGTGAGCTTTAGGTTTTGTATTTTTATTTACAG
TAGGAGGATTAACAGGGGTAATTTTAGCTAATTCTTCAATTGATATTATTCTTCAC
GATACTTACTATGTAGTAGCTCATTTTCACTATGTTCTTTCTATAGGAGCTGTATT
TGCAATTATAGCCGGAATTGTCCAATGATTCCCATTATTTACTGGACTAACCTTA
AATAATTTTTTTTTAAAAACCCAATTTATTACTATATTTTTAGGTGTAAATTTAACAT
TTTTTCCTCAACACTTTTTAGGGCTAAGAGGAATGCCTCGGCGATATTCGGATTA
TCCAGATGCTTTCACTATCTGAAATGCCATTTCATCTATTGGTTCCATTATTTCTC
TCACAAGAATTATCTTTTTTTTATTTATCTTATGAGAAAGGCTTTCTTCCCAACGT
AAATCTTTGAGAACTTTAAATGTAACAACTTCAATTGAATGGTTCAAGCCCTACC
CCCTAGTG
MEX_MAL_1
ATTATTAGACAAGAAAGGGGGAAAAAAGAAGCTTTTGGACCTTAGGAATAATTTA
TGCCATAATAGCTATCGGATTATTAGGATTCGTAGTTTGAGCTCACCATATATTTA
CAGTAGATAGATGTAGATACCCGAGCTTACTTCACTTCAGCAACTATAATTATTG
CGGTTCCAACTGGGATTAAAGTCTTTAGATGACTAGCCACCTTTCATGGAACTCA
AATTTTAAATAGACCAGTAACCCTGTGAGCTTTAGGTTTTGTATTTTTATTTACAG
TAGGAGGATTAACAGGGGTAATTTTAGCTAATTCTTCAATTGATATTATTCTTCAC
GATACTTACTATGTAGTAGCTCATTTTCACTATGTTCTTTCTATAGGAGCTGTATT
TGCAATTATAGCCGGAATTGTCCAATGATTCCCATTATTTACTGGACTAACCTTA
AATAATTTTTTTTTAAAAACCCAATTTATTACTATATTTTTAGGTGTAAATTTAACAT
TTTTTCCTCAACACTTTTTAGGGCTAAGAGGAATGCCTCGGCGATATTCGGATTA
TCCAGATGCTTTCACTATCTGAAATGCCATTTCATCTATTGGTTCCATTATTTCTC
TCACAAGAATTATCTTTTTTTTATTTATCTTATGAGAAAGGCTTTCTTCCCAACGT
AAATCTTTGAGAACTTTAAATGTAACAACTTCAATTGAATGGTTCAAGCCCTACC
CCCTAGTG
MEX_MAL_2
ATTATTAGACAAGAAAGGGGGAAAAAAGAAGCTTTTGGACCTTAGGAATAATTTA
TGCCATAATAGCTATGGGATTATTAGGATTCGTCGTTTGAGCTCACCATATATTT
ACAGTAGATAGATGTAGATACCCGAGCTTACTTCACTTCAGCAACTATAATTATT
GCGGTTCCAACTGGGATTAAAGTCTTTAGATGACTAGCCACCTTTCATGGAACT
CAAATTTTAAATAGACCAGTAACCCTGTGAGCTTTAGGTTTTGTATTTTTATTTAC
AGTAGGAGGATTAACAGGGGTAATTTTAGCTAATTCTTCAATTGATATTATTCTTC
ACGATACTTACTATGTAGTAGCTCATTTTCACTATGTTCTTTCTATAGGAGCTGTA
TTTGCAATTATAGCCGGAATTGTCCAATGATTCCCATTATTTACTGGACTAACCTT
AAATAATTTTTTTTTAAAAACCCAATTTATTACTATATTTTTAGGTGTAAATTTAACA
TTTTTTCCTCAACACTTTTTAGGGCTAAGAGGAATGCCTCGGCGATATTCGGATT
ATCCAGATGCTTTCACTATCTGAAATGCCATTTCATCCATTGGTTCCATTATTTCT
CTCACAAGAATTATCTTTTTTTTATTTATCCTATGAGAAAGGCTTTCTTCCCAACG
TAAATCTTTGAGAACTTTAAATGTAACAACTTCAATTGAATGGTTCAAGCCCTACC
CCCTAGTG
MEX_OCU_1
ATTATTAGACAAGAAAGGGGGAAAAAAGAAGCTTTTGGACCTTAGGAATAATTTA

TGCCATAATAGCTATCGGATTATTAGGATTCGTAGTTTGAGCTCACCATATATTTA
CAGTAGATAGATGTAGATACCCGAGCTTACTTCACTTCAGCAACTATAATTATTG
CGGTTCCAACTGGGATTAAAGTCTTTAGATGACTAGCCACCTTTCATGGAACTCA
AATTTTAAATAGACCAGTAACCCTGTGAGCTTTAGGTTTTGTATTTTTATTTACAG
TAGGAGGATTAACAGGGGTAATTTTAGCTAATTCTTCAATTGATATTATTCTTCAC
GATACTTACTATGTAGTAGCTCATTTTCACTATGTTCTTTCTATAGGAGCTGTATT
TGCAATTATAGCCGGAATTGTCCAATGATTCCCATTATTTACTGGACTAACCTTA
AATAATTTTTTTTTAAAAACCCAATTTATTACTATATTTTTAGGTGTAAATTTAACAT
TTTTTCCTCAACACTTTTTAGGGCTAAGAGGAATGCCTCGGCGATATTCGGATTA
TCCAGATGCTTTCACTATCTGAAATGCCATTTCATCTATTGGTTCCATTATTTCTC
TCACAAGAATTATCTTTTTTTTTATTTATCTTATGAGAAAGGCTTTCTTCCCAACGT
AAATCTTTGAGAACTTTAAATGTAACAACTTCAATTGAATGGTTCAAGCCCTACC
CCCTAGTG
MEX_OCU_2
ATTATTAGACAAGAAAGGGGGAAAAAAGAAGCTTTTGGACCTTAGGAATAATTTA
TGCCATAATAGCTATCGGATTATTAGGATTCGTAGTTTGAGCTCACCATATATTTA
CAGTAGATAGATGTAGATACCCGAGCTTACTTCACTTCAGCAACTATAATTATTG
CGGTTCCAACTGGGATTAAAGTCTTTAGATGACTAGCCACCTTTCATGGAACTCA
AATTTTAAATAGACCAGTAACCCTGTGAGCTTTAGGTTTTGTATTTTTATTTACAG
TAGGAGGATTAACAGGGGTAATTTTAGCTAATTCTTCAATTGATATTATTCTTCAC
GATACTTACTATGTAGTAGCTCATTTTCACTATGTTCTTTCTATAGGAGCTGTATT
TGCAATTATAGCCGGAATTGTCCAATGATTCCCATTATTTACTGGACTAACCTTA
AATAATTTTTTTTTAAAAACCCAATTTATTACTATATTTTTAGGTGTAAATTTAACAT
TTTTTCCTCAACACTTTTTAGGGCTAAGAGGAATGCCTCGGCGATATTCGGATTA
TCCAGATGCTTTCACTATCTGAAATGCCATTTCATCTATTGGTTCCATTATTTCTC
TCACAAGAATTATCTTTTTTTTTATTTATCTTATGAGAAAGGCTTTCTTCCCAACGT
AAATCTTTGAGAACTTTAAATGTAACAACTTCAATTGAATGGTTCAAGCCCTACC
CCCTAGTG
MEX_SAG_1
ATTATTAGACAAGAAAGGGGGAAAAAAGAAGCTTTTGGACCTTAGGAATAATTTA
TGCCATAATAGCTATCGGATTATTAGGATTCGTAGTTTGAGCTCACCATATATTTA
CAGTAGATAGATGTAGATACCCGAGCTTACTTCACTTCAGCAACTATAATTATTG
CGGTTCCAACTGGGATTAAAGTCTTTAGATGACTAGCCACCTTTCATGGAACTCA
AATTTTAAATAGACCAGTAACCCTGTGAGCTTTAGGTTTTGTATTTTTATTTACAG
TAGGAGGATTAACAGGGGTAATTTTAGCTAATTCTTCAATTGATATTATTCTTCAC
GATACTTACTATGTAGTAGCTCATTTTCACTATGTTCTTTCTATAGGAGCTGTATT
TGCAATTATAGCCGGAATTGTCCAATGATTCCCATTATTTACTGGGCTAACCTTA
AATAATTTTTTTTTAAAAACCCAATTTATTACTATATTTTTAGGTGTAAATTTAACAT
TTTTTCCTCAACACTTTTTAGGGCTAAGAGGAATGCCTCGGCGATATTCGGATTA
TCCAGATGCTTTCACTATCTGAAATGCCATTTCATCTATTGGTTCCATTATTTCTC
TCACAAGAATTATCTTTTTTTTTATTTATCTTATGAGAAAGGCTTTCTTCCCAACGT
AAATCTTTGAGAACTTTAAATGTAACAACTTCAATTGAATGGTTCAAGCCCTACC
CCCTAGTG
MEX_SAG_2
ATTATTAGACAAGAAAGGGGGAAAAAAGAAGCTTTTGGACCTTAGGAATAATTTA
TGCCATAATAGCTATCGGATTATTAGGATTCGTAGTTTGAGCTCACCATATATTTA
CAGTAGATAGATGTAGATACCCGAGCTTACTTCACTTCAGCAACTATAATTATTG
CGGTTCCAACTGGGATTAAAGTCTTTAGATGACTAGCCACCTTTCATGGAACTCA
AATTTTAAATAGACCAGTAACCCTGTGAGCTTTAGGTTTTGTATTTTTATTTACAG
TAGGAGGATTAACAGGGGTAATTTTAGCTAATTCTTCAATTGATATTATTCTTCAC

GATACTTACTATGTAGTAGCTCATTTTCACTATGTTCTTTCTATAGGAGCTGTATT
TGCAATTATAGCCGGAATTGTCCAATGATTCCCATTATTTACTGGGCTAACCTTA
AATAATTTTTTTTTTAAAAACCCAATTTATTACTATATTTTTAGGTGTAAATTTAACAT
TTTTTCCTCAACACTTTTTAGGGCTAAGAGGAATGCCTCGGCGATATTCGGATTA
TCCAGATGCTTTCACTATCTGAAATGCCATTTCATCTATTGGTTCCATTATTTCTC
TCACAAGAATTATCTTTTTTTTATTTATCTTATGAGAAAGGCTTTCTTCCCAACGT
AAATCTTTGAGAACTTTAAATGTAACAACTTCAATTGAATGGTTCAAGCCCTACC
CCCTAGTG
MEX_SJC_1
ATTATTAGACAAGAAAGGGGGAAAAAAGAAGCTTTTGGACCTTAGGAATAATTTA
TGCCATAATAGCTATCGGATTATTAGGATTCGTAGTTTGAGCTCACCATATATTTA
CAGTAGATAGATGTAGATACCCGAGCTTACTTCACTTCAGCAACTATAATTATTG
CGGTTCCAACTGGGATTAAAGTCTTTAGATGACTAGCCACCTTTCATGGAACTCA
AATTTTAAATAGACCAGTAACCCTGTGAGCTTTAGGTTTTGTATTTTTATTTACAG
TAGGAGGATTAACAGGGGTAATTTTAGCTAATTCTTCAATTGATATTATTCTTCAC
GATACTTACTATGTAGTAGCTCATTTTCACTATGTTCTTTCTATAGGAGCTGTATT
TGCAATTATAGCCGGAATTGTCCAATGATTCCCATTATTTACTGGACTAACCTTA
AATAATTTTTTTTTTAAAAACCCAATTTATTACTATATTTTTAGGTGTAAATTTAACAT
TTTTTCCTCAACACTTTTTAGGGCTAAGAGGAATGCCTCGGCGATATTCGGATTA
TCCAGATGCTTTCACTATCTGAAATGCCATTTCATCTATTGGTTCCATTATTTCTC
TCACAAGAATTATCTTTTTTTTATTTATCTTATGAGAAAGGCTTTCTTCCCAACGT
AAATCTTTGAGAACTTTAAATGTAACAACTTCAATTGAATGATTCAAGCCCTACCC
CCTAGTG
MEX_SJC_2
ATTATTAGACAAGAAAGGGGGAAAAAAGAAGCTTTTGGACCTTAGGAATAATTTA
TGCCATAATAGCTATCGGATTATTAGGATTCGTAGTTTGAGCTCACCATATATTTA
CAGTAGATAGATGTAGATACCCGAGCTTACTTCACTTCAGCAACTATAATTATTG
CGGTTCCAACTGGGATTAAAGTCTTTAGATGACTAGCCACCTTTCATGGAACTCA
AATTTTAAATAGACCAGTAACCCTGTGAGCTTTAGGTTTTGTATTTTTATTTACAG
TAGGAGGATTAACAGGGGTAATTTTAGCTAATTCTTCAATTGATATTATTCTTCAC
GATACTTACTATGTAGTAGCTCATTTTCACTATGTTCTTTCTATAGGAGCTGTATT
TGCAATTATAGCCGGAATTGTCCAATGATTCCCATTATTTACTGGACTAACCTTA
AATAATTTTTTTTTTAAAAACCCAATTTATTACTATATTTTTAGGTGTAAATTTAACAT
TTTTTCCTCAACACTTTTTAGGGCTAAGAGGAATGCCTCGGCGATATTCGGATTA
TCCAGATGCTTTCACTATCTGAAATGCCATTTCATCTATTGGTTCCATTATTTCTC
TCACAAGAATTATCTTTTTTTTATTTATCTTATGAGAAAGGCTTTCTTCCCAACGT
AAATCTTTGAGAACTTTAAATGTAACAACTTCAATTGAATGGTTCAAGCCCTACC
CCCTAGTG
MEX_SJC_3
ATTATTAGACAAGAAAGGGGGAAAAAAGAAGCTTTTGGACCTTAGGAATAATTTA
TGCCATAATAGCTATCGGATTATTAGGATTCGTAGTTTGAGCTCACCATATATTTA
CAGTAGATAGATGTAGATACCCGAGCTTACTTCACTTCAGCAACTATAATTATTG
CGGTTCCAACTGGGATTAAAGTCTTTAGATGACTAGCCACCTTTCATGGAACTCA
AATTTTAAATAGACCAGTAACCCTGTGAGCTTTAGGTTTTGTATTTTTATTTACAG
TAGGAGGATTAACAGGGGTAATTTTAGCTAATTCTTCAATTGATATTATTCTTCAC
GATACTTACTATGTAGTAGCTCATTTTCACTATGTTCTTTCTATAGGAGCTGTATT
TGCAATTATAGCCGGAATTGTCCAATGATTCCCATTATTTACTGGACTAACCTTA
AATAATTTTTTTTTTAAAAACCCAATTTATTACTATATTTTTAGGTGTAAATTTAACAT
TTTTTCCTCAACACTTTTTAGGGCTAAGAGGAATGCCTCGGCGATATTCGGATTA
TCCAGATGCTTTCACTATCTGAAATGCCATTTCATCTATTGGTTCCATTATTTCTC

TCACAAGAATTATCTTTTTTTTATTTATCTTATGAGAAAGGCTTTCTTCCCAACGT
AAATCTTTGAGAACTTTAAATGTAACAACTTCAATTGAATGGTTCAAGCCCTACC
CCCTAGTG
MEX_SJS
ATTATTAGACAAGAAAGGGGGAAAAAAGAACCTTTTGGACCTTAGGAATAATTTA
TGCCATAATAGCTATCGGATTATTAGGATTCGTAGTTTGAGCTCACCATATATTTA
CAGTAGATAGATGTAGATACCCGAGCTTACTTCACTTCAGCAACTATAATTATTG
CGGTTCCAACTGGAATTAAAGTCTTTAGATGACTAGCCACCTTTCATGGAACTCA
AATTTTAAATAGACCAGTAACCCTGTGAGCTTTAGGTTTTGTATTTTTATTTACAG
TAGGAGGATTAACAGGGGTAATTTTAGCTAATTCTTCAATTGATATTATTCTTCAC
GATACTTACTATGTAGTAGCTCATTTTCACTATGTTCTTTCTATAGGAGCTGTATT
TGCAATTATAGCCGGAATTGTCCAATGATTCCCATTATTTACTGGACTAACCTTA
AATAATTTTTTTTTAAAAACCCAATTTATTACTATATTTTTAGGTGTAAATTTAACAT
TTTTTCCTCAACACTTTTTAGGGCTAAGAGGAATGCCTCGGCGATATTCGGATTA
TCCAGATGCTTTCACTATCTGAAATGCCATTTCATCTATTGGTTCCATTATTTCTC
TCACAAGAATTATCTTTTTTTTATTTATCTTATGAGAAAGGCTTTCTTCCCAACGT
AAATCTTTGAGAACTTTAAATGTAACAACTTCAATTGAATGGTTCAAGCCCTACC
CCCTAGTG
MEX_SJS_1
ATTATTAGACAAGAAAGGGGGAAAAAAGAAGCTTTTGGACCTTAGGAATAATTTA
TGCCATAATACCTATCGGATTATTAGGATTCGTCGTTTGAGCTCACCATATATTTA
CAGTAGATAGATGTAGATACCCGAGCTTACTTCACTTCAGCAACTATAATTATTG
CGGTTCCAACTGGGATTAAAGTCTTTAGATGACTAGCCACCTTTCATGGAACTCA
AATTTTAAATAGACCAGTAACCCTGTGAGCTTTAGGTTTTGTATTTTTATTTACAG
TAGGAGGATTAACAGGGGTAATTTTAGCTAATTCTTCAATTGATATTATTCTTCAC
GATACTTACTATGTAGTAGCTCATTTTCACTATGTTCTTTCTATAGGAGCTGTATT
TGCAATTATAGCCGGAATTATCCAATGATTCCCATTATTTACTGGACTAACCTTAA
ATAATTTTTTTTTAAAAACCCAATTTATTACTATATTTTTAGGTGTAAATTTAACATT
TTTTCCTCAACACTTTTTAGGGCTAAGAGGAATGCCTCGGCGATATTCGGATTAT
CCAGATGCTTTCACTATCTGAAATGCCATTTCATCCATTGGTTCCATTATTTCTCT
CACAAGAATTATCTTTTTTTTATTTATCTTATGAGAAAGGCTTTCTTCCCAACGTA
AATCTTTGAGAACTTCAAATGTAACAACTTCAATTGAATGGTGCAAGCCCTACCC
CCTAGTG
MEX_SJS_3
ATTATTAGACAAGAAAGGGGGAAAAAAGAAGCTTTTGGACCTTAGGAATAATTTA
TGCCATAATACCTATCGGATTATTAGGATTCGTCGTTTGAGCTCACCATATATTTA
CAGTAGATAGATGTAGATACCCGAGCTTACTTCACTTCAGCAACTATAATTATTG
CGGTTCCAACTGGGATTAAAGTCTTTAGATGACTAGCCACCTTTCATGGAACTCA
AATTTTAAATAGACCAGTAACCCTGTGAGCTTTAGGTTTTGTATTTTTATTTACAG
TAGGAGGATTAACAGGGGTAATTTTAGCTAATTCTTCAATTGATATTATTCTTCAC
GATACTTACTATGTAGTAGCTCATTTTCACTATGTTCTTTCTATAGGAGCTGTATT
TGCAATTATAGCCGGAATTATCCAATGATTCCCATTATTTACTGGACTAACCTTAA
ATAATTTTTTTTTAAAAACCCAATTTATTACTATATTTTTAGGTGTAAATTTAACATT
TTTTCCTCAACACTTTTTAGGGCTAAGAGGAATGCCTCGGCGATATTCGGATTAT
CCAGATGCTTTCACTATCTGAAATGCCATTTCATCCATTGGTTCCATTATTTCTCT
CACAAGAATTATCTTTTTTTTATTTATCTTATGAGAAAGGCTTTCTTCCCAACGTA
AATCTTTGAGAACTTCAAATGTAACAACTTCAATTGAATGGTGCAAGCCCTACCC
CCTAGTG
MEX_SPT_1
ATTATTAGACAAGAAAGGGGGAAAAAAGAAGCTTTTGGACCTTAGGAATAATTTA

TGCCATAATAGCTATCGGATTATTAGGATTCGTAGTTTGAGCTCACCATATATTTA
CAGTAGATAGATGTAGATACCCGAGCTTACTTCACTTCAGCAACTATAATTATTG
CGGTTCCAACTGGGATTAAAGTCTTTAGATGACTAGCCACCTTTCATGGAACTCA
AATTTTAAATAGACCAGTAACCCTGTGAGCTCTAGGTTTTGTATTTTTATTTACAG
TAGGAGGATTAACAGGGGTAATTTTAGCTAATTCTTCAATTGATATTATTCTTCAC
GATACTTACTATGTAGTAGCTCATTTTCACTATGTTCTTTCTATAGGAGCTGTATT
TGCAATTATAGCCGGAATTGTCCAATGATTCCCATTATTTACTGGACTAACCTTA
AATAATTTTTTTTTAAAAACCCAATTTATTACTATATTTTTAGGTGTAAATTTAACAT
TTTTTCCTCAACACTTTTTAGGGCTAAGAGGAATGCCTCGGCGATATTCGGATTA
TCCAGATGCTTTCACTATCTGAAATGCCATTTCATCTATTGGTTCCATTATTTCTC
TCACAAGAATTATCTTTTTTTTTATTTATCTTATGAGAAAGGCTTTCTTCCCAACGT
AAATCTTTGAGAACTTTAAATGTAACAACTTCAATTGAATGGTTCAAGCCCTACC
CCCTAGTG
MEX_SPT_2
ATTATTAGACAAGAAAGGGGGAAAAAAGAAGCTTTTGGACCTTAGGAATAATTTA
TGCCATAATAGCTATCGGATTATTAGGATTCGTAGTTTGAGCTCACCATATATTTA
CAGTAGATAGATGTAGATACCCGAGCTTACTTCACTTCAGCAACTATAATTATTG
CGGTTCCAACTGGGATTAAAGTCTTTAGATGACTAGCCACCTTTCATGGAACTCT
AATTTTAAATAGACCAGTAACCCTATGAGCTTTAGGTTTTGTATTTTTATTTACAG
TAGGAGGTTTAACAGGAGTAATTTTAGCTAATTCTTCAATTGATATTATTCTTCAC
GATACTTACTATGTAGTAGCTCATTTTCACTATGTTCTTTCTATAGGAGCTGTATT
TGCAATTATAGCCGGAATTGTCCAATGATTCCCATTATTTACTGGACTAACCTTA
AATAATTTTTTTTTAAAAACCCAATTTATTACTATATTTTTAGGTGTAAATTTAACAT
TTTTTCCTCAACACTTTTTAGGGCTAAGAGGAATGCCTCGGCGATATTCGGATTA
TCCAGATGCTTTCACTATCTGAAATGCCATTTCATCTATTGGTTCTATTATTTCTC
TCACAAGAATTATCTTTTTTTTTATTTATCTTATGAGAAAGGCTTTCTTCCCAACGT
AAATCTTTGAGAACTTTAAATGTAACAACTTCAATTGAATGGTTCAAGCCCTACC
CCCTAGTG
MEX_SPT_3
ATTATTAGACAAGAAAGGGGGAAAAAAGAAGCTTTTGGACCTTAGGAATAATTTA
TGCCATAATAGCTATCGGATTATTAGGATTCGTAGTTTGAGCTCACCATATATTTA
CAGTAGATAGATGTAGATACCCGAGCTTACTTCACTTCAGCAACTATAATTATTG
CGGTTCCAACTGGGATTAAAGTCTTTAGATGACTAGCCACCTTTCATGGAACTCT
AATTTTAAATAGACCAGTAACCCTATGAGCTTTAGGTTTTGTATTTTTATTTACAG
TAGGAGGTTTAACAGGAGTAATTTTAGCTAATTCTTCAATTGATATTATTCTTCAC
GATACTTACTATGTAGTAGCTCATTTTCACTATGTTCTTTCTATAGGAGCTGTATT
TGCAATTATAGCCGGAATTGTCCAATGATTCCCATTATTTACTGGACTAACCTTA
AATAATTTTTTTTTAAAAACCCAATTTATTACTATATTTTTAGGTGTAAATTTAACAT
TTTTTCCTCAACACTTTTTAGGGCTAAGAGGAATGCCTCGGCGATATTCGGATTA
TCCAGATGCTTTCACTATCTGAAATGCCATTTCATCTATTGGTTCTATTATTTCTC
TCACAAGAATTATCTTTTTTTTTATTTATCTTATGAGAAAGGCTTTCTTCCCAACGT
AAATCTTTGAGAACTTTAAATGTAACAACTTCAATTGAATGGTTCAAGCCCTACC
CCCTAGTG
MEX_TEP
ATTATTAGACAAGAAAGGGGGAAAAAAGAAGCTTTTGGACCTTAGGAATAATTTA
TGCCATAATAGCTATCGGATTATTAGGATTCGTAGTTTGAGCTCACCATATATTTA
CAGTAGATAGATGTAGATACCCGAGCTTACTTCACTTCAGCAACTATAATTATTG
CGGTTCCAACTGGGATTAAAGTCTTTAGATGACTAGCCACCTTTCATGGAACTCA
AATTTTAAATAGACCAGTAACCCTGTGAGCTTTAGGTTTTGTATTTTTATTTACAG
TAGGAGGATTAACAGGGGTAATTTTAGCTAATTCTTCAATTGATATTATTCTTCAC

GATACTTACTATGTAGTAGCTCATTTTCACTATGTTCTTTCTATAGGAGCTGTATT
TGCAATTATAGCCGGAATTGTCCAATGATTCCCATTATTTACTGGACTAACCTTA
AATAATTTTTTTTTAAAAACCCAATTTATTACTATATTTTTAGGTGTAAATTTAACAT
TTTTTCCTCAACACTTTTTAGGGCTAAGAGGAATGCCTCGGCGATATTCGGATTA
TCCAGATGCTTTCACTATCTGAAATGCCATTTCATCTATTGGTTCCATTATTTCTC
TCACAAGAATTATCTTTTTTTTTATTTATCTTATGAGAAAGGCTTTCTTCCCAACGT
AAATCTTTGAGAACTTTAAATGTAACAACTTCAATTGAATGGTTCAAGCCCTACC
CCCTAGTG
MEX_TEP_1
ATTATTAGACAAGAAAGGGGGAAAAAAGAAGCTTTTGGACCTTAGGAATAATTTA
TGCCATAATAGCTATCGGATTATTAGGATTCGTAGTTTGAGCTCACCATATATTTA
CAGTAGATAGATGTAGATACCCGAGCTTACTTCACTTCAGCAACTATAATTATTG
CGGTTCCAACTGGGATTAAAGTCTTTAGATGACTAGCCACCTTTCATGGAACTCA
AATTTTAAATAGACCAGTAACCCTGTGAGCTTTAGGTTTTGTATTTTTATTTACAG
TAGGAGGATTAACAGGGGTAATTTTAGCTAATTCTTCAATTGATATTATTCTTCAC
GATACTTACTATGTAGTAGCTCATTTTCACTATGTTCTTTCTATAGGAGCTGTATT
TGCAATTATAGCCGGAATTGTCCAATGATTCCCATTATTTACTGGACTAACCTTA
AATAATTTTTTTTTAAAAACCCAATTTATTACTATATTTTTAGGTGTAAATTTAACAT
TTTTTCCTCAACACTTTTTAGGGCTAAGAGGAATGCCTCGGCGATATTCGGATTA
TCCAGATGCTTTCACTATCTGAAATGCCATTTCATCTATTGGTTCCATTATTTCTC
TCACAAGAATTATCTTTTTTTTTATTTATCTTATGAGAAAGGCTTTCTTCCCAACGT
AAATCTTTGAGAACTTTAAATGTAACAACTTCAATTGAATGGTTCAAGCCCTACC
CCCTAGTG
MEX_TEP_2
ATTATTAGACAAGAAAGGGGGAAAAAAGAAGCTTTTGGACCTTAGGAATAATTTA
TGCCATAATAGCTATCGGATTATTAGGATTCGTAGTTTGAGCTCACCATATATTTA
CAGTAGATAGATGTAGATACCCGAGCTTACTTCACTTCAGCAACTATAATTATTG
CGGTTCCAACTGGGATTAAAGTCTTTAGATGACTAGCCACCTTTCATGGAACTCA
AATTTTAAATAGACCAGTAACCCTGTGAGCTTTAGGTTTTGTATTTTTATTTACAG
TAGGAGGATTAACAGGGGTAATTTTAGCTAATTCTTCAATTGATATTATTCTTCAC
GATACTTACTATGTAGTAGCTCATTTTCACTATGTTCTTTCTATAGGAGCTGTATT
TGCAATTATAGCCGGAATTGTCCAATGATTTCCATTATTTACTGGACTAACCTTAA
ATAATTTTTTTTTAAAAACCCAATTTATTACTATATTTTTAGGTGTAAATTTAACATT
TTTTCCTCAACACTTTTTAGGGCTAAGAGGAATGCCTCGGCGATATTCGGATTAT
CCAGATGCTTTCACTATCTGAAATGCCATTTCATCTATTGGTTCCATTATTTCTCT
CACAAGAATTATCTTTTTTTTTATTTATCTTATGAGAAAGGCTTTCTTCCCAACGTA
AATCTTTGAGAACTTTAAATGTAACAACTTCAATTGAATGGTTCAAGCCCTACCC
CCTAGTG
MEX_TLA
ATTATTAGACAAGAAAGGGGGAAAAAAGAAGCTTTTGGACCTTAGGAATAATTTA
TGCCATAATAGCTATCGGATTATTAGGATTCGTAGTTTGAGCTCACCATATATTTA
CAGTAGATAGATGTAGATACCCGAGCTTACTTCACTTCAGCACCTATGATTATTG
CGGTTCCAACTGGGATTAAAGTCTTTAGATGACTAGCCACCTTTCATGGAACTCA
AATTTTAAATAGACCAGTAACCCTGTGAGCTTTAGGTTTTGTATTTTTATTTACAG
TAGGAGGATTAACAGGGGTAATTTTAGCTAATTCTTCAATTGATATTATTCTTCAC
GATACTTACTATGTAGTAGCTCATTTTCACTATGTTCTTTCTATAGGAGCTGTATT
TGCAATTATAGCCGGAATTGTCCAATGATTCCCATTATTTACTGGACTAACCTTA
AATAATTTTTTTTTAAAAACCCAATTTATTACTATATTTTTAGGTGTAAATTTAACAT
TTTTTCCTCAACACTTTTTAGGGCTAAGAGGAATGCCTCGGCGATATTCGGATTA
TCCAGATGCTTTCACTATCTGAAATGCCATTTCATCTATTGGTTCCATTATTTCTC

TCACAAGAATTATCTTTTTTTTATTTATCTTATGAGAAAGGCTTTCTTCCCAACGT
AAATCTTTGAGAACTTTAAATGTAACAACTTCAATTGAATGGTTCAAGCCCTACC
CCCTAGTG
MEX_TLA_2
ATTATTAGACAAGAAAGGGGGAAAAAAGAACCTTTTGGACCTTAGGAATAATTTA
TCCCTTATTACCTATCGGATTATTAGGATTCGTCGTTTGAGCTCACCATATCTTTA
CAGTAGATAGCTGTAGATACCCGAGCTTACTTCACTTCAGCAACTATAATTATTG
CGGTTCCAACTGGGATTAAAGTCTTTAGATGACTAGCCACCTTTCATGGAACTCA
AATTTTAAATAGACCAGTAACCCTGTGAGCTTTAGGTTTTGTATTTTTATTTACAG
TAGGAGGATTAACAGGGGTAATTTTAGCTAATTCTTCAATTGATATTATTCTTCAC
GATACTTACTATGTAGTAGCTCATTTTCACTATGTTCTTTCTATAGGAGCTGTATT
TGCAATTATAGCCGGAATTAGCCAATGATTCCCATTATTTACTGGACTAACCTTA
AATAATTTTTTTTTAAAAACCCAATTTATTACTATATTTTTAGGTGTAAATTTAACAT
TTTTTCCTCAACACTTTTTAGGGCTAAGAGGAATGCCTCGGCGATATTCGGATTA
TCCAGATGCTTTCACTACCTGAAATGCCATTTCATCCATTGGTTCCATTATTTCTC
TCACAAGAATTATCTTTTTTTTATTTATCTTATGAGAAAGGCTTTCTTCCCAACGT
AAATCTTTGAGAACTTCAAATGTAACAACTTCAATTGAATGGTGCAAGCCCTACC
CCCTAGTG
MEX_TLA_3
ATTATTAGACAAGAAAGGGGGAAAAAAGAAGCTTTTGGACCTTAGGAATAATTTA
TGCCATAATAGCTATCGGATTATTAGGATTCGTAGTTTGAGCTCACCATATATTTA
CAGTAGATAGATGTAGATACCCGAGCTTACTTCACTTCAGCAACTATAATTATTG
CGGTTCCAACTGGGATTAAAGTCTTTAGATGACTAGCCACCTTTCATGGAACTCA
AATTTTAAATAGACCAGTAACCCTGTGAGCTTTAGGTTTTGTATTTTTATTTACAG
TAGGAGGATTAACAGGGGTAATTTTAGCTAATTCTTCAATTGATATTATTCTTCAC
GATACTTACTATGTAGTAGCTCATTTTCACTATGTTCTTTCTATAGGAGCTGTATT
TGCAATTATAGCCGGAATTGTCCAATGATTCCCATTATTTACTGGACTAACCTTA
AATAATTTTTTTTTAAAAACCCAATTTATTACTATATTTTTAGGTGTAAATTTAACAT
TTTTTCCTCAACACTTTTTAGGGCTAAGAGGAATGCCTCGGCGATATTCGGATTA
TCCAGATGCTTTCACTATCTGAAATGCCATTTCATCTATTGGTTCCATTATTTCTC
TCACAAGAATTATCTTTTTTTTATTTATCTTATGAGAAAGGCTTTCTTCCCAACGT
AAATCTTTGAGAACTTTAAATGTAACAACTTCAATTGAATGGTTCAAGCCCTACC
CCCTAGTG
MEX_YAU_1
ATTATTAGACAAGAAAGGGGGAAAAAAGAAGCTTTTGGACCTTAGGAATAATTTA
TGCCATGATAGCTATCGGATTATTAGGATTCGTAGTTTGAGCTCACCATATATTT
ACAGTAGATAGATGTAGATACCCGAGCTTACTTCACTTCAGCAACTATAATTATT
GCGGTTCCAACTGGGATTAAAGTCTTTAGATGGCTAGCCACCTTTCATGGAACT
CAAATTTTAAATAGACCAGTAACCCTGTGAGCTTTAGGTTTTGTATTTTTATTTAC
AGTAGGAGGATTAACAGGGGTAATTTTAGCTAATTCTTCAATTGATATTATTCTTC
ACGATACTTACTATGTAGTAGCTCATTTTCACTATGTTCTTTCTATAGGAGCTGTA
TTTGCAATTATAGCCGGAATTAGCCAATGATTCCCATTATTTACTGGACTAACCTT
AAATAATTTTTTTTTAAAAACCCAATTTATTACTATATTTTTAGGTGTAAATTTAACA
TTTTTTCCTCAACACTTTTTAGGGCTAAGAGGAATGCCTCGGCGATATTCGGATT
ATCCAGATGCTTTCACTATCTGAAATGCCATTTCATCTATTGGTTCCATTATTTCT
CTCACAAGAATTATCTTTTTTTTATTTATCTTATGAGAAAGGCTTTCTTCCCAACG
TAAATCTTTGAGAACTTTAAATGTAACAACTTCAATTGAATGGTTCAAGCCCTACC
CCCTAGTG
MEX_YAU_2
ATTATTAGACAAGAAAGGGGGAAAAAAGAAGCTTTTGGACCTTAGGAATAATTTA

TGCCATAATAGCTATCGGATTATTAGGATTCGTAGTTTGAGCTCACCATATATTTA
CAGTAGATAGATGTAGATACCCGAGCTTACTTCACTTCAGCAACTATAATTATTG
CGGTTCCAACTGGGATTAAAGTCTTTAGATGACTAGCCACCTTTCATGGAACTCA
AATTTTAAATAGACCAGTAACCCTGTGAGCTTTAGGTTTTGTATTTTTATTTACAG
TAGGAGGATTAACAGGGGTAATTTTAGCTAATTCTTCAATTGATATTATTCTTCAC
GATACTTACTATGTAGTAGCTCATTTTCACTATGTTCTTTCTATAGGAGCTGTATT
TGCAATTATAGCCGGAATTGTCCAATGATTCCCATTATTTACTGGACTAACCTTA
AATAATTTTTTTTTAAAAACCCAATTTATTACTATATTTTTAGGTGTAAATTTAACAT
TTTTTCCTCAACACTTTTTAGGGCTAAGAGGAATGCCTCGGCGATATTCGGATTA
TCCAGATGCTTTCACTATCTGAAATGCCATTTCATCTATTGGCTCCATTATTTCTC
TCACAAGAATTATCTTTTTTTTTATTTATCTTATGAGAAAGGCTTTCTTCCCAACGT
AAATCTTTGAGAACTTTAAATGTAACAACTTCAATTGAATGGTTCAAGCCCTACC
CCCTAGTG
CAM_YAH
ATTATTAGACAAGAAAGGGGGAAAAAAGAAGCTTTTGGACCTTAGGAATAATTTA
TGCCATGATAGCTATCGGATTATTAGGATTCGTAGTTTGAGCTCTCCATATATTT
ACAGTAGATAGATGTAGATACCCGAGCTTACTTCACTTCAGCAACTATGATTATC
GCGGTTCCAACTGGGATTAAAGTCTTTAGGTGACTAGCCACCTTTCATGGAACT
CAAATTTTAAATAGACCAGTAACCCTGTGAGCTTTAGGTTTTGTATTTTTATTTAC
AGTAGGAGGATTAACAGGGGTAATTTTAGCTAATTCTTCAATTGATATTATTCTTC
ACGATACTTACTATGTAGTAGCTCATTTTCACTATGTTCTTTCTATAGGAGCTGTA
TTTGCAATTATAGCCGGAATTGTCCAATGATTCCCATTATTTACTGGACTAACCTT
AAATAATTTTTTTTTAAAAACCCAATTTATTACTATATTTTTAGGTGTAAATTTAACA
TTTTTTCCTCAACACTTTTTAGGGCTAAGAGGAATGCCTCGGCGATATTCGGATT
ATCCAGATGCTTTCACTATCTGAAATGCCATTTCATCTATTGGTTCCATTATTTCT
CTCACAAGAATTATCTTTTTTTTTATTTATCTTATGAGAAAGGCTTTCTTCCCAACG
TAAATCTTTGAGAACTTTAAATGTAACAACTTCAATTGAATGGTTCAAGCCCTACC
CCCTAGTG
CAM_YAH01
ATTATTAGACAAGAAAGGGGGAAAAAAGAAGCTTTTGGACCTTAGGAATAATTTA
TGCCATGATAGCTATCGGATTATTAGGATTCGTAGTTTGAGCTCTCCATATATTT
ACAGTAGATAGATGTAGATACCCGAGCTTACTTCACTTCAGCAACTATGATTATC
GCGGTTCCAACTGGGATTAAAGTCTTTAGGTGACTAGCCACCTTTCATGGAACT
CAAATTTTAAATAGACCAGTAACCCTGTGAGCTTTAGGTTTTGTATTTTTATTTAC
AGTAGGAGGATTAACAGGGGTAATTTTAGCTAATTCTTCAATTGATATTATTCTTC
ACGATACTTACTATGTAGTAGCTCATTTTCACTATGTTCTTTCTATAGGAGCTGTA
TTTGCAATTATAGCCGGAATTGTCCAATGATTCCCATTATTTACTGGACTAACCTT
AAATAATTTTTTTTTAAAAACCCAATTTATTACTATATTTTTAGGTGTAAATTTAACA
TTTTTTCCTCAACACTTTTTAGGGCTAAGAGGAATGCCTCGGCGATATTCGGATT
ATCCAGATGCTTTCACTATCTGAAATGCCATTTCATCTATTGGTTCCATTATTTCT
CTCACAAGAATTATCTTTTTTTTTATTTATCTTATGAGAAAGGCTTTCTTCCCAACG
TAAATCTTTGAGAACTTTAAATGTAACAACTTCAATTGAATGGTTCAAGCCCTACC
CCCTAGTG
CAM_YAH02
ATTATTAGACAAGAAAGGGGGAAAAAAGAAGCTTTTGGACCTTAGGAATAATTTA
TGCCATGATAGCTATCGGATTATTAGGATTCGTAGTTTGAGCTCTCCATATATTT
ACAGTAGATAGATGTAGATACCCGAGCTTACTTCACTTCAGCAACTATGATTATC
GCGGTTCCAACTGGGATTAAAGTCTTTAGGTGACTAGCCACCTTTCATGGAACT
CAAATTTTAAATAGACCAGTAACCCTGTGAGCTTTAGGTTTTGTATTTTTATTTAC
AGTAGGAGGATTAACAGGGGTAATTTTAGCTAATTCTTCAATTGATATTATTCTTC

ACGATACTTACTATGTAGTAGCTCATTTTCACTATGTTCTTTCTATAGGAGCTGTA
TTTGCAATTATAGCCGGAATTGTCCAATGATTCCCATTATTTACTGGACTAACCTT
AAATAATTTTTTTTTAAAAACCCAATTTATTACTATATTTTTAGGTGTAAATTTAACA
TTTTTTCCTCAACACTTTTTAGGGCTAAGAGGAATGCCTCGGCGATATTCGGATT
ATCCAGATGCTTTCACTATCTGAAATGCCATTTCATCTATTGGTTCCATTATTTCT
CTCACAAGAATTATCTTTTTTTTATTTATCTTATGAGAAAGGCTTTCTTCCCAACG
TAAATCTTTGAGAACTTTAAATGTAACAACTTCAATTGAATGGTTCAAGCCCTACC
CCCTAGTG
China
ATTATTAGACAAGAAAGGGGGAAAAAAGAAGCTTTTGGACCTTAGGAATAATTTA
TGCCATGATAGCTATCGGATTATTAGGATTCGTAGTTTGAGCTCACCATATATTT
ACAGTAGATAGATGTAGATACCCGAGCTTACTTCACTTCAGCAACTATAATTATT
GCGGTTCCAACTGGGATTAAAGTCTTTAGATGACTAGCCACCTTTCATGGAACT
CAAATTTTAAATAGACCAGTAACCCTGTGAGCTTTAGGTTTTGTATTTTTATTTAC
AGTAGGAGGATTAACAGGGGTAATTTTAGCTAATTCTTCAATTGATATTATTCTTC
ACGATACTTACTATGTAGTAGCTCATTTTCACTATGTTCTTTCTATAGGAGCTGTA
TTTGCAATTATAGCCGGAATTGTCCAATGATTCCCATTATTTACCGGACTAACCT
TAAATAATTTTTTTTTAAAAACCCAATTTATTACTATATTTTTAGGTGTAAATTTAAC
ATTTTTTCCCCAACACTTTTTAGGGCTAAGAGGAATGCCTCGGCGATATTCGGAT
TATCCAGATGCTTTCACTATCTGAAATGCCATTTCATCTATTGGTTCCATTATTTC
TCTCACAAGAATTATCTTTTTTTTATTTATCTTATGAGAAAGGCTTTCTTCCCAAC
GTAAATCTTTGAGAACTTTAAATGTAACAACTTCAATTGAATGGTTCAAGCCCTAC
CCCCTAGTG
FRA_CHA_5
ATTATTAGACAAGAAAGGGGGAAAAAAGAAGCTTTTGGACCTTAGGAATAATTTA
TGCCATGATAGCTATCGGATTATTAGGATTCGTAGTTTGAGCTCACCATATATTT
ACAGTAGATAGATGTAGATACCCGAGCTTACTTCACTTCAGCAACTATAATTATT
GCGGTTCCAACTGGGATTAAAGTCTTTAGATGACTAGCCACCTTTCATGGAACT
CAAATTTTAAATAGACCAGTAACCCTGTGAGCTTTAGGCTTTGTATTTTTATTTAC
AGTAGGAGGATTAACAGGGGTAATTTTAGCTAATTCTTCAATTGATATTATTCTTC
ACGATACTTACTATGTAGTAGCTCATTTTCACTATGTTCTTTCTATAGGAGCTGTA
TTTGCAATTATAGCCGGAATTGTCCAATGATTTCCATTATTTACTGGACTAACCTT
AAATAATTTTTTTTTAAAAACCCAATTTATTACTATATTTTTAGGTGTAAATTTAACA
TTTTTTCCTCAACACTTTTTAGGGCTAAGAGGAATGCCTCGGCGATATTCGGATT
ATCCAGATGCTTTCACTATCTGAAATGCCATTTCATCTATTGGTTCCATTATTTCT
CTCACAAGAATTATCTTTTTTTTATTTATCTTATGAGAAAGGCTTTCTTCCCAACG
TAAATCTTTGAGAACTTTAAATGTAACAACTTCAATTGAATGGTTCAAGCCCTACC
CCCTAGTG
SPA_PRA_1
ATTATTAGACAAGAAAGGGGGAAAAAAGAAGCTTTTGGACCTTAGGAATAATTTA
TGCCATGATAGCTATCGGATTATTAGGATTCGTAGTTTGAGCTCACCATATATTT
ACAGTAGATAGATGTAGATACCCGAGCTTACTTCACTTCAGCAACTATAATTATT
GCGGTTCCAACTGGGATTAAAGTCTTTAGATGACTAGCCACCTTTCATGGAACT
CAAATTTTAAATAGACCAGTAACCCTGTGAGCTTTAGGTTTTGTATTTTTATTTAC
AGTAGGAGGATTAACAGGGGTAATTTTAGCTAATTCTTCAATTGATATTATTCTTC
ACGATACTTACTATGTAGTAGCTCATTTTCACTATGTTCTTTCTATAGGAGCTGTA
TTTGCAATTATAGCCGGAATTGTCCAATGATTCCCATTATTTACCGGACTAACCT
TAAATAATTTTTTTTTAAAAACCCAATTTATTACTATATTTTTAGGTGTAAATTTAAC
ATTTTTTCCCCAACACTTTTTAGGGCTAAGAGGAATGCCTCGGCGATATTCGGAT
TATCCAGATGCTTTCACTATCTGAAATGCCATTTCATCTATTGGTTCCATTATTTC

TCTCACAAGAATTATCTTTTTTTTATTTATCTTATGAGAAAGGCTTTCTTCCCAAC
GTAAATCTTTGAGAACTTTAAATGTAACAACTTCAATTGAATGGTTCAAGCCCTAC
CCCCTAGTG
SWI_NEU_1
ATTATTAGACAAGAAAGGGGGAAAAAAGAAGCTTTTGGACCTTAGGAATAATTTA
TGCCATGATAGCTATCGGATTATTAGGATTCGTAGTTTGAGCTCACCATATATTT
ACAGTAGATAGATGTAGATACCCGAGCTTACTTCACTTCAGCAACTATGATTATC
GCGGTTCCAACTGGGATTAAAGTCTTTAGGTGGCTAGCCACCTTTCATGGAACT
CAAATTTTAAATAGACCAGTAACCCTGTGAGCTTTAGGTTTTGTATTTTTATTTAC
AGTAGGAGGATTAACAGGGGTAATTTTAGCTAATTCTTCAATTGATATTATTCTTC
ACGATACTTACTATGTAGTAGCTCATTTTCACTATGTTCTTTCTATAGGAGCTGTA
TTTGCAATTATAGCCGGAATTGTCCAATGATTCCCATTATTTACTGGACTAACCTT
AAATAATTTTTTTTTAAAAACCCAATTTATTACTATATTTTTAGGTGTAAATTTAACA
TTTTTTCCTCAACACTTTTTAGGGCTAAGAGGAATGCCTCGGCGATATTCGGATT
ATCCAGATGCTTTCACTATCTGAAATGCCATTTCATCTATTGGTTCCATTATTTCT
CTCACAAGAATTATCTTTTTTTTATTTATCTTATGAGAAAGGCTTTCTTCCCAACG
TAAATCTTTGAGAACTTTAAATGTAACAACTTCAATTGAATGGTTCAAGCCCTACC
CCCTAGTG
SWT_NEU
ATTATTAGACAAGAAAGGGGGAAAAAAGAAGCTTTTGGACCTTAGGAATAATTTA
TGCCATGATAGCTATCGGATTATTAGGATTCGTAGTTTGAGCTCACCATATATTT
ACAGTAGATAGATGTAGATACCCGAGCTTACTTCACTTCAGCAACTATGATTATC
GCGGTTCCAACTGGGATTAAAGTCTTTAGGTGGCTAGCCACCTTTCATGGAACT
CAAATTTTAAATAGACCAGTAACCCTGTGAGCTTTAGGTTTTGTATTTTTATTTAC
AGTAGGAGGATTAACAGGGGTAATTTTAGCTAATTCTTCAATTGATATTATTCTTC
ACGATACTTACTATGTAGTAGCTCATTTTCACTATGTTCTTTCTATAGGAGCTGTA
TTTGCAATTATAGCCGGAATTGTCCAATGATTCCCATTATTTACTGGACTAACCTT
AAATAATTTTTTTTTAAAAACCCAATTTATTACTATATTTTTAGGTGTAAATTTAACA
TTTTTTCCTCAACACTTTTTAGGGCTAAGAGGAATGCCTCGGCGATATTCGGATT
ATCCAGATGCTTTCACTATCTGAAATGCCATTTCATCTATTGGTTCCATTATTTCT
CTCACAAGAATTATCTTTTTTTTATTTATCTTATGAGAAAGGCTTTCTTCCCAACG
TAAATCTTTGAGAACTTTAAATGTAACAACTTCAATTGAATGGTTCAAGCCCTACC
CCCTAGTG
SWT_NEU2
ATTATTAGACAAGAAAGGGGGAAAAAAGAAGCTTTTGGACCTTAGGAATAATTTA
TGCCATGATAGCTATCGGATTATTAGGATTCGTAGTTTGAGCTCACCATATATTT
ACAGTAGATAGATGTAGATACCCGAGCTTACTTCACTTCAGCAACTATGATTATC
GCGGTTCCAACTGGGATTAAAGTCTTTAGGTGGCTAGCCACCTTTCATGGAACT
CAAATTTTAAATAGACCAGTAACCCTGTGAGCTTTAGGTTTTGTATTTTTATTTAC
AGTAGGAGGATTAACAGGGGTAATTTTAGCTAATTCTTCAATTGATATTATTCTTC
ACGATACTTACTATGTAGTAGCTCATTTTCACTATGTTCTTTCTATAGGAGCTGTA
TTTGCAATTATAGCCGGAATTGTCCAATGATTCCCATTATTTACTGGACTAACCTT
AAATAATTTTTTTTTAAAAACCCAATTTATTACTATATTTTTAGGTGTAAATTTAACA
TTTTTTCCTCAACACTTTTTAGGGCTAAGAGGAATGCCTCGGCGATATTCGGATT
ATCCAGATGCTTTCACTATCTGAAATGCCATTTCATCTATTGGTTCCATTATTTCT
CTCACAAGAATTATCTTTTTTTTATTTATCTTATGAGAAAGGCTTTCTTCCCAACG
TAAATCTTTGAGAACTTTAAATGTAACAACTTCAATTGAATGGTTCAAGCCCTACC
CCCTAGTG
AOPE_15
ATTATTAGACAAGAAAGGGGGAAAAAAGAAGCTTTTGGACCTTAGGAATAATTTA

TGCTATAATAGCTATCGGATTATTAGGATTCGTAGTTTGAGCTCACCATATATTTA
CAGTAGATAGATGTAGATACCCGAGCTTACTTCACTTCAGCAACTATAATTATTG
CGGTTCCAACTGGGATTAAAGTCTTTAGATGACTAGCCACCTTTCATGGAACTCA
AATTTTAAATAGACCAGTAACCCTGTGAGCTTTAGGTTTTGTATTTTTATTTACAG
TAGGAGGATTAACAGGGGTAATTTTAGCTAATTCTTCAATTGATATTATTCTCCAC
GATACTTACTATGTAGTAGCTCATTTTCACTATGTTCTTTCTATAGGAGCTGTATT
TGCAATTATAGCCGGAATTGTCCAATGATTCCCATTATTTACCGGACTAACCTTA
AATAATTTTTTTTTAAAAACCCAATTTATTACTATATTTTTAGGTGTAAATTTAACAT
TTTTTCCTCAACACTTTTTAGGGCTAAGAGGAATGCCTCGGCGATATTCGGATTA
TCCAGATGCTTTCACTATCTGAAATGCCATTTCATCTATTGGTTCCATTATTTCTC
TCACAAGAATTATCTTTTTTTTTATTTATCTTATGAGAAAGGCTTTCTTCCCAACGT
AAATCTTTGAGAACTTTAAATGTAACAACTTCAATTGAATGGTTCAAGCCCTACC
CCCTAGTG
AOPE_2
ATTATTAGACAAGAAAGGGGGAAAAAAGAAGCTTTTGGACCTTAGGAATAATTTA
TGCTATAATAGCTATCGGATTATTAGGATTCGTAGTTTGAGCTCACCATATATTTA
CAGTAGATAGATGTAGATACCCGAGCTTACTTCACTTCAGCAACTATAATTATTG
CGGTTCCAACTGGGATTAAAGTCTTTAGATGACTAGCCACCTTTCATGGAACTCA
AATTTTAAATAGACCAGTAACCCTGTGAGCTTTAGGTTTTGTATTTTTATTTACAG
TAGGAGGATTAACAGGGGTAATTTTAGCTAATTCTTCAATTGATATTATTCTCCAC
GATACTTACTATGTAGTAGCTCATTTTCACTATGTTCTTTCTATAGGAGCTGTATT
TGCAATTATAGCCGGAATTGTCCAATGATTCCCATTATTTACCGGACTAACCTTA
AATAATTTTTTTTTAAAAACCCAATTTATTACTATATTTTTAGGTGTAAATTTAACAT
TTTTTCCTCAACACTTTTTAGGGCTAAGAGGAATGCCTCGGCGATATTCGGATTA
TCCAGATGCTTTCACTATCTGAAATGCCATTTCATCTATTGGTTCCATTATTTCTC
TCACAAGAATTATCTTTTTTTTTATTTATCTTATGAGAAAGGCTTTCTTCCCAACGT
AAATCTTTGAGAACTTTAAATGTAACAACTTCAATTGAATGGTTCAAGCCCTACC
CCCTAGTG
AOPE_3
ATTATTAGACAAGAAAGGGGGAAAAAAGAAGCTTTTGGACCTTAGGAATAATTTA
TGCTATAATAGCTATCGGATTATTAGGATTCGTAGTTTGAGCTCACCATATATTTA
CAGTAGATAGATGTAGATACCCGAGCTTACTTCACTTCAGCAACTATAATTATTG
CGGTTCCAACTGGGATTAAAGTCTTTAGATGACTAGCCACCTTTCATGGAACTCA
AATTTTAAATAGACCAGTAACCCTGTGAGCTTTAGGTTTTGTATTTTTATTTACAG
TAGGAGGATTAACAGGGGTAATTTTAGCTAATTCTTCAATTGATATTATTCTCCAC
GATACTTACTATGTAGTAGCTCATTTTCACTATGTTCTTTCTATAGGAGCTGTATT
TGCAATTATAGCCGGAATTGTCCAATGATTCCCATTATTTACCGGACTAACCTTA
AATAATTTTTTTTTAAAAACCCAATTTATTACTATATTTTTAGGTGTAAATTTAACAT
TTTTTCCTCAACACTTTTTAGGGCTAAGAGGAATGCCTCGGCGATATTCGGATTA
TCCAGATGCTTTCACTATCTGAAATGCCATTTCATCTATTGGTTCCATTATTTCTC
TCACAAGAATTATCTTTTTTTTTATTTATCTTATGAGAAAGGCTTTCTTCCCAACGT
AAATCTTTGAGAACTTTAAATGTAACAACTTCAATTGAATGGTTCAAGCCCTACC
CCCTAGTG
AOPE_12
ATTATTAGACAAGAAAGGGGGAAAAAAGAAGCTTTTGGACCTTAGGAATAATTTA
TGCTATAATAGCTATCGGATTATTAGGATTCGTAGTTTGAGCTCACCATATATTTA
CAGTAGATAGATGTAGATACCCGAGCTTACTTCACTTCAGCAACTATAATTATTG
CGGTTCCAACTGGGATTAAAGTCTTTAGATGACTAGCCACCTTTCATGGAACTCA
AATTTTAAATAGACCAGTAACCCTGTGAGCTTTAGGTTTTGTATTTTTATTTACAG
TAGGAGGATTAACAGGGGTAATTTTAGCTAATTCTTCAATTGATATTATTCTCCAC

GATACTTACTATGTAGTAGCTCATTTTCACTATGTTCTTTCTATAGGAGCTGTATT
TGCAATTATAGCCGGAATTGTCCAATGATTCCCATTATTTACCGGACTAACCTTA
AATAATTTTTTTTTAAAAACCCAATTTATTACTATATTTTTAGGTGTAAATTTAACAT
TTTTTCCTCAACACTTTTTAGGGCTAAGAGGAATGCCTCGGCGATATTCGGATTA
TCCAGATGCTTTCACTATCTGAAATGCCATTTCATCTATTGGTTCCATTATTTCTC
TCACAAGAATTATCTTTTTTTTTATTTATCTTATGAGAAAGGCTTTCTTCCCAACGT
AAATCTTTGAGAACTTTAAATGTAACAACTTCAATTGAATGGTTCAAGCCCTACC
CCCTAGTG
AOPE_13
ATTATTAGACAAGAAAGGGGGAAAAAAGAAGCTTTTGGACCTTAGGAATAATTTA
TGCTATAATAGCTATCGGATTATTAGGATTCGTAGTTTGAGCTCACCATATATTTA
CAGTAGATAGATGTAGATACCCGAGCTTACTTCACTTCAGCAACTATAATTATTG
CGGTTCCAACTGGGATTAAAGTCTTTAGATGACTAGCCACCTTTCATGGAACTCA
AATTTTAAATAGACCAGTAACCCTGTGAGCTTTAGGTTTTGTATTTTTATTTACAG
TAGGAGGATTAACAGGGGTAATTTTAGCTAATTCTTCAATTGATATTATTCTCCAC
GATACTTACTATGTAGTAGCTCATTTTCACTATGTTCTTTCTATAGGAGCTGTATT
TGCAATTATAGCCGGAATTGTCCAATGATTCCCATTATTTACCGGACTAACCTTA
AATAATTTTTTTTTAAAAACCCAATTTATTACTATATTTTTAGGTGTAAATTTAACAT
TTTTTCCTCAACACTTTTTAGGGCTAAGAGGAATGCCTCGGCGATATTCGGATTA
TCCAGATGCTTTCACTATCTGAAATGCCATTTCATCTATTGGTTCCATTATTTCTC
TCACAAGAATTATCTTTTTTTTTATTTATCTTATGAGAAAGGCTTTCTTCCCAACGT
AAATCTTTGAGAACTTTAAATGTAACAACTTCAATTGAATGGTTCAAGCCCTACC
CCCTAGTG
AOPE_14
ATTATTAGACAAGAAAGGGGGAAAAAAGAAGCTTTTGGACCTTAGGAATAATTTA
TGCTATAATAGCTATCGGATTATTAGGATTCGTAGTTTGAGCTCACCATATATTTA
CAGTAGATAGATGTAGATACCCGAGCTTACTTCACTTCAGCAACTATAATTATTG
CGGTTCCAACTGGGATTAAAGTCTTTAGATGACTAGCCACCTTTCATGGAACTCA
AATTTTAAATAGACCAGTAACCCTGTGAGCTTTAGGTTTTGTATTTTTATTTACAG
TAGGAGGATTAACAGGGGTAATTTTAGCTAATTCTTCAATTGATATTATTCTCCAC
GATACTTACTATGTAGTAGCTCATTTTCACTATGTTCTTTCTATAGGAGCTGTATT
TGCAATTATAGCCGGAATTGTCCAATGATTCCCATTATTTACCGGACTAACCTTA
AATAATTTTTTTTTAAAAACCCAATTTATTACTATATTTTTAGGTGTAAATTTAACAT
TTTTTCCTCAACACTTTTTAGGGCTAAGAGGAATGCCTCGGCGATATTCGGATTA
TCCAGATGCTTTCACTATCTGAAATGCCATTTCATCTATTGGTTCCATTATTTCTC
TCACAAGAATTATCTTTTTTTTTATTTATCTTATGAGAAAGGCTTTCTTCCCAACGT
AAATCTTTGAGAACTTTAAATGTAACAACTTCAATTGAATGGTTCAAGCCCTACC
CCCTAGTG
AOPE_4
ATTATTAGACAAGAAAGGGGGAAAAAAGAAGCTTTTGGACCTTAGGAATAATTTA
TGCTATAATAGCTATCGGATTATTAGGATTCGTAGTTTGAGCTCACCATATATTTA
CAGTAGATAGATGTAGATACCCGAGCTTACTTCACTTCAGCAACTATAATTATTG
CGGTTCCAACTGGGATTAAAGTCTTTAGATGACTAGCCACCTTTCATGGAACTCA
AATTTTAAATAGACCAGTAACCCTGTGAGCTTTAGGTTTTGTATTTTTATTTACAG
TAGGAGGATTAACAGGGGTAATTTTAGCTAATTCTTCAATTGATATTATTCTCCAC
GATACTTACTATGTAGTAGCTCATTTTCACTATGTTCTTTCTATAGGAGCTGTATT
TGCAATTATAGCCGGAATTGTCCAATGATTCCCATTATTTACCGGACTAACCTTA
AATAATTTTTTTTTAAAAACCCAATTTATTACTATATTTTTAGGTGTAAATTTAACAT
TTTTTCCTCAACACTTTTTAGGGCTAAGAGGAATGCCTCGGCGATATTCGGATTA
TCCAGATGCTTTCACTATCTGAAATGCCATTTCATCTATTGGTTCCATTATTTCTC

TCACAAGAATTATCTTTTTTTTATTTATCTTATGAGAAAGGCTTTCTTCCCAACGT
AAATCTTTGAGAACTTTAAATGTAACAACTTCAATTGAATGGTTCAAGCCCTACC
CCCTAGTG
PE2_1
ATTATTAGACAAGAAAGGGGGAAAAAAGAAGCTTTTGGACCTTAGGAATAATTTA
TGCTATAATAGCTATCGGATTATTAGGATTCGTAGTTTGAGCTCACCATATATTTA
CAGTAGATAGATGTAGATACCCGAGCTTACTTCACTTCAGCAACTATAATTATTG
CGGTTCCAACTGGGATTAAAGTCTTTAGATGACTAGCCACCTTTCATGGAACTCA
AATTTTAAATAGACCAGTAACCCTGTGAGCTTTAGGTTTTGTATTTTTATTTACAG
TAGGAGGATTAACAGGGGTAATTTTAGCTAATTCTTCAATTGATATTATTCTCCAC
GATACTTACTATGTAGTAGCTCATTTTCACTATGTTCTTTCTATAGGAGCTGTATT
TGCAATTATAGCCGGAATTGTCCAATGATTCCCATTATTTACCGGACTAACCTTA
AATAATTTTTTTTTAAAAACCCAATTTATTACTATATTTTTAGGTGTAAATTTAACAT
TTTTTCCTCAACACTTTTTAGGGCTAAGAGGAATGCCTCGGCGATATTCGGATTA
TCCAGATGCTTTCACTATCTGAAATGCCATTTCATCTATTGGTTCCATTATTTCTC
TCACAAGAATTATCTTTTTTTTATTTATCTTATGAGAAAGGCTTTCTTCCCAACGT
AAATCTTTGAGAACTTTAAATGTAACAACTTCAATTGAATGGTTCAAGCCCTACC
CCCTAGTG
PE2_2
ATTATTAGACAAGAAAGGGGGAAAAAAGAAGCTTTTGGACCTTAGGAATAATTTA
TGCTATAATAGCTATCGGATTATTAGGATTCGTAGTTTGAGCTCACCATATATTTA
CAGTAGATAGATGTAGATACCCGAGCTTACTTCACTTCAGCAACTATAATTATTG
CGGTTCCAACTGGGATTAAAGTCTTTAGATGACTAGCCACCTTTCATGGAACTCA
AATTTTAAATAGACCAGTAACCCTGTGAGCTTTAGGTTTTGTATTTTTATTTACAG
TAGGAGGATTAACAGGGGTAATTTTAGCTAATTCTTCAATTGATATTATTCTCCAC
GATACTTACTATGTAGTAGCTCATTTTCACTATGTTCTTTCTATAGGAGCTGTATT
TGCAATTATAGCCGGAATTGTCCAATGATTCCCATTATTTACCGGACTAACCTTA
AATAATTTTTTTTTAAAAACCCAATTTATTACTATATTTTTAGGTGTAAATTTAACAT
TTTTTCCTCAACACTTTTTAGGGCTAAGAGGAATGCCTCGGCGATATTCGGATTA
TCCAGATGCTTTCACTATCTGAAATGCCATTTCATCTATTGGTTCCATTATTTCTC
TCACAAGAATTATCTTTTTTTTATTTATCTTATGAGAAAGGCTTTCTTCCCAACGT
AAATCTTTGAGAACTTTAAATGTAACAACTTCAATTGAATGGTTCAAGCCCTACC
CCCTAGTG
PE2_3
ATTATTAGACAAGAAAGGGGGAAAAAAGAAGCTTTTGGACCTTAGGAATAATTTA
TGCTATAATAGCTATCGGATTATTAGGATTCGTAGTTTGAGCTCACCATATATTTA
CAGTAGATAGATGTAGATACCCGAGCTTACTTCACTTCAGCAACTATAATTATTG
CGGTTCCAACTGGGATTAAAGTCTTTAGATGACTAGCCACCTTTCATGGAACTCA
AATTTTAAATAGACCAGTAACCCTGTGAGCTTTAGGTTTTGTATTTTTATTTACAG
TAGGAGGATTAACAGGGGTAATTTTAGCTAATTCTTCAATTGATATTATTCTCCAC
GATACTTACTATGTAGTAGCTCATTTTCACTATGTTCTTTCTATAGGAGCTGTATT
TGCAATTATAGCCGGAATTGTCCAATGATTCCCATTATTTACCGGACTAACCTTA
AATAATTTTTTTTTAAAAACCCAATTTATTACTATATTTTTAGGTGTAAATTTAACAT
TTTTTCCTCAACACTTTTTAGGGCTAAGAGGAATGCCTCGGCGATATTCGGATTA
TCCAGATGCTTTCACTATCTGAAATGCCATTTCATCTATTGGTTCCATTATTTCTC
TCACAAGAATTATCTTTTTTTTATTTATCTTATGAGAAAGGCTTTCTTCCCAACGT
AAATCTTTGAGAACTTTAAATGTAACAACTTCAATTGAATGGTTCAAGCCCTACC
CCCTAGTG
PE2_4
ATTATTAGACAAGAAAGGGGGAAAAAAGAAGCTTTTGGACCTTAGGAATAATTTA

TGCTATAATAGCTATCGGATTATTAGGATTCGTAGTTTGAGCTCACCATATATTTA
CAGTAGATAGATGTAGATACCCGAGCTTACTTCACTTCAGCAACTATAATTATTG
CGGTTCCAACTGGGATTAAAGTCTTTAGATGACTAGCCACCTTTCATGGAACTCA
AATTTTAAATAGACCAGTAACCCTGTGAGCTTTAGGTTTTGTATTTTTATTTACAG
TAGGAGGATTAACAGGGGTAATTTTAGCTAATTCTTCAATTGATATTATTCTCCAC
GATACTTACTATGTAGTAGCTCATTTTCACTATGTTCTTTCTATAGGAGCTGTATT
TGCAATTATAGCCGGAATTGTCCAATGATTCCCATTATTTACCGGACTAACCTTA
AATAATTTTTTTTTAAAAACCCAATTTATTACTATATTTTTAGGTGTAAATTTAACAT
TTTTTCCTCAACACTTTTTAGGGCTAAGAGGAATGCCTCGGCGATATTCGGATTA
TCCAGATGCTTTCACTATCTGAAATGCCATTTCATCTATTGGTTCCATTATTTCTC
TCACAAGAATTATCTTTTTTTTATTTATCTTATGAGAAAGGCTTTCTTCCCAACGT
AAATCTTTGAGAACTTTAAATGTAACAACTTCAATTGAATGGTTCAAGCCCTACC
CCCTAGTG
PE2_5
ATTATTAGACAAGAAAGGGGGAAAAAAGAAGCTTTTGGACCTTAGGAATAATTTA
TGCTATAATAGCTATCGGATTATTAGGATTCGTAGTTTGAGCTCACCATATATTTA
CAGTAGATAGATGTAGATACCCGAGCTTACTTCACTTCAGCAACTATAATTATTG
CGGTTCCAACTGGGATTAAAGTCTTTAGATGACTAGCCACCTTTCATGGAACTCA
AATTTTAAATAGACCAGTAACCCTGTGAGCTTTAGGTTTTGTATTTTTATTTACAG
TAGGAGGATTAACAGGGGTAATTTTAGCTAATTCTTCAATTGATATTATTCTCCAC
GATACTTACTATGTAGTAGCTCATTTTCACTATGTTCTTTCTATAGGAGCTGTATT
TGCAATTATAGCCGGAATTGTCCAATGATTCCCATTATTTACCGGACTAACCTTA
AATAATTTTTTTTTAAAAACCCAATTTATTACTATATTTTTAGGTGTAAATTTAACAT
TTTTTCCTCAACACTTTTTAGGGCTAAGAGGAATGCCTCGGCGATATTCGGATTA
TCCAGATGCTTTCACTATCTGAAATGCCATTTCATCTATTGGTTCCATTATTTCTC
TCACAAGAATTATCTTTTTTTTATTTATCTTATGAGAAAGGCTTTCTTCCCAACGT
AAATCTTTGAGAACTTTAAATGTAACAACTTCAATTGAATGGTTCAAGCCCTACC
CCCTAGTG
PE2_6
ATTATTAGACAAGAAAGGGGGAAAAAAGAAGCTTTTGGACCTTAGGAATAATTTA
TGCTATAATAGCTATCGGATTATTAGGATTCGTAGTTTGAGCTCACCATATATTTA
CAGTAGATAGATGTAGATACCCGAGCTTACTTCACTTCAGCAACTATAATTATTG
CGGTTCCAACTGGGATTAAAGTCTTTAGATGACTAGCCACCTTTCATGGAACTCA
AATTTTAAATAGACCAGTAACCCTGTGAGCTTTAGGTTTTGTATTTTTATTTACAG
TAGGAGGATTAACAGGGGTAATTTTAGCTAATTCTTCAATTGATATTATTCTCCAC
GATACTTACTATGTAGTAGCTCATTTTCACTATGTTCTTTCTATAGGAGCTGTATT
TGCAATTATAGCCGGAATTGTCCAATGATTCCCATTATTTACCGGACTAACCTTA
AATAATTTTTTTTTAAAAACCCAATTTATTACTATATTTTTAGGTGTAAATTTAACAT
TTTTTCCTCAACACTTTTTAGGGCTAAGAGGAATGCCTCGGCGATATTCGGATTA
TCCAGATGCTTTCACTATCTGAAATGCCATTTCATCTATTGGTTCCATTATTTCTC
TCACAAGAATTATCTTTTTTTTATTTATCTTATGAGAAAGGCTTTCTTCCCAACGT
AAATCTTTGAGAACTTTAAATGTAACAACTTCAATTGAATGGTTCAAGCCCTACC
CCCTAGTG
PE2_7
ATTATTAGACAAGAAAGGGGGAAAAAAGAAGCTTTTGGACCTTAGGAATAATTTA
TGCTATAATAGCTATCGGATTATTAGGATTCGTAGTTTGAGCTCACCATATATTTA
CAGTAGATAGATGTAGATACCCGAGCTTACTTCACTTCAGCAACTATAATTATTG
CGGTTCCAACTGGGATTAAAGTCTTTAGATGACTAGCCACCTTTCATGGAACTCA
AATTTTAAATAGACCAGTAACCCTGTGAGCTTTAGGTTTTGTATTTTTATTTACAG
TAGGAGGATTAACAGGGGTAATTTTAGCTAATTCTTCAATTGATATTATTCTCCAC

GATACTTACTATGTAGTAGCTCATTTTCACTATGTTCTTTCTATAGGAGCTGTATT
TGCAATTATAGCCGGAATTGTCCAATGATTCCCATTATTTACCGGACTAACCTTA
AATAATTTTTTTTTAAAAACCCAATTTATTACTATATTTTTAGGTGTAAATTTAACAT
TTTTTCCTCAACACTTTTTAGGGCTAAGAGGAATGCCTCGGCGATATTCGGATTA
TCCAGATGCTTTCACTATCTGAAATGCCATTTCATCTATTGGTTCCATTATTTCTC
TCACAAGAATTATCTTTTTTTTTATTTATCTTATGAGAAAGGCTTTCTTCCCAACGT
AAATCTTTGAGAACTTTAAATGTAACAACTTCAATTGAATGGTTCAAGCCCTACC
CCCTAGTG
PE3_1
ATTATTAGACAAGAAAGGGGGAAAAAAGAAGCTTTTGGACCTTAGGAATAATTTA
TGCTATAATAGCTATCGGATTATTAGGATTCGTAGTTTGAGCTCACCATATATTTA
CAGTAGATAGATGTAGATACCCGAGCTTACTTCACTTCAGCAACTATAATTATTG
CGGTTCCAACTGGGATTAAAGTCTTTAGATGACTAGCCACCTTTCATGGAACTCA
AATTTTAAATAGACCAGTAACCCTGTGAGCTTTAGGTTTTGTATTTTTATTTACAG
TAGGAGGATTAACAGGGGTAATTTTAGCTAATTCTTCAATTGATATTATTCTCCAC
GATACTTACTATGTAGTAGCTCATTTTCACTATGTTCTTTCTATAGGAGCTGTATT
TGCAATTATAGCCGGAATTGTCCAATGATTCCCATTATTTACCGGACTAACCTTA
AATAATTTTTTTTTAAAAACCCAATTTATTACTATATTTTTAGGTGTAAATTTAACAT
TTTTTCCTCAACACTTTTTAGGGCTAAGAGGAATGCCTCGGCGATATTCGGATTA
TCCAGATGCTTTCACTATCTGAAATGCCATTTCATCTATTGGTTCCATTATTTCTC
TCACAAGAATTATCTTTTTTTTTATTTATCTTATGAGAAAGGCTTTCTTCCCAACGT
AAATCTTTGAGAACTTTAAATGTAACAACTTCAATTGAATGGTTCAAGCCCTACC
CCCTAGTG
PE4_1
ATTATTAGACAAGAAAGGGGGAAAAAAGAAGCTTTTGGACCTTAGGAATAATTTA
TGCTATAATAGCTATCGGATTATTAGGATTCGTAGTTTGAGCTCACCATATATTTA
CAGTAGATAGATGTAGATACCCGAGCTTACTTCACTTCAGCAACTATAATTATTG
CGGTTCCAACTGGGATTAAAGTCTTTAGATGACTAGCCACCTTTCATGGAACTCA
AATTTTAAATAGACCAGTAACCCTGTGAGCTTTAGGTTTTGTATTTTTATTTACAG
TAGGAGGATTAACAGGGGTAATTTTAGCTAATTCTTCAATTGATATTATTCTCCAC
GATACTTACTATGTAGTAGCTCATTTTCACTATGTTCTTTCTATAGGAGCTGTATT
TGCAATTATAGCCGGAATTGTCCAATGATTCCCATTATTTACCGGACTAACCTTA
AATAATTTTTTTTTAAAAACCCAATTTATTACTATATTTTTAGGTGTAAATTTAACAT
TTTTTCCTCAACACTTTTTAGGGCTAAGAGGAATGCCTCGGCGATATTCGGATTA
TCCAGATGCTTTCACTATCTGAAATGCCATTTCATCTATTGGTTCCATTATTTCTC
TCACAAGAATTATCTTTTTTTTTATTTATCTTATGAGAAAGGCTTTCTTCCCAACGT
AAATCTTTGAGAACTTTAAATGTAACAACTTCAATTGAATGGTTCAAGCCCTACC
CCCTAGTG
PE4_2
ATTATTAGACAAGAAAGGGGGAAAAAAGAAGCTTTTGGACCTTAGGAATAATTTA
TGCTATAATAGCTATCGGATTATTAGGATTCGTAGTTTGAGCTCACCATATATTTA
CAGTAGATAGATGTAGATACCCGAGCTTACTTCACTTCAGCAACTATAATTATTG
CGGTTCCAACTGGGATTAAAGTCTTTAGATGACTAGCCACCTTTCATGGAACTCA
AATTTTAAATAGACCAGTAACCCTGTGAGCTTTAGGTTTTGTATTTTTATTTACAG
TAGGAGGATTAACAGGGGTAATTTTAGCTAATTCTTCAATTGATATTATTCTCCAC
GATACTTACTATGTAGTAGCTCATTTTCACTATGTTCTTTCTATAGGAGCTGTATT
TGCAATTATAGCCGGAATTGTCCAATGATTCCCATTATTTACCGGACTAACCTTA
AATAATTTTTTTTTAAAAACCCAATTTATTACTATATTTTTAGGTGTAAATTTAACAT
TTTTTCCTCAACACTTTTTAGGGCTAAGAGGAATGCCTCGGCGATATTCGGATTA
TCCAGATGCTTTCACTATCTGAAATGCCATTTCATCTATTGGTTCCATTATTTCTC

TCACAAGAATTATCTTTTTTTTTATTTATCTTATGAGAAAGGCTTTCTTCCCAACGT
AAATCTTTGAGAACTTTAAATGTAACAACTTCAATTGAATGGTTCAAGCCCTACC
CCCTAGTG
PE4_3
ATTATTAGACAAGAAAGGGGGAAAAAAGAAGCTTTTGGACCTTAGGAATAATTTA
TGCTATAATAGCTATCGGATTATTAGGATTCGTAGTTTGAGCTCACCATATATTTA
CAGTAGATAGATGTAGATACCCGAGCTTACTTCACTTCAGCAACTATAATTATTG
CGGTTCCAACTGGGATTAAAGTCTTTAGATGACTAGCCACCTTTCATGGAACTCA
AATTTTAAATAGACCAGTAACCCTGTGAGCTTTAGGTTTTGTATTTTTATTTACAG
TAGGAGGATTAACAGGGGTAATTTTAGCTAATTCTTCAATTGATATTATTCTCCAC
GATACTTACTATGTAGTAGCTCATTTTCACTATGTTCTTTCTATAGGAGCTGTATT
TGCAATTATAGCCGGAATTGTCCAATGATTCCCATTATTTACCGGACTAACCTTA
AATAATTTTTTTTTAAAAACCCAATTTATTACTATATTTTTAGGTGTAAATTTAACAT
TTTTTCCTCAACACTTTTTAGGGCTAAGAGGAATGCCTCGGCGATATTCGGATTA
TCCAGATGCTTTCACTATCTGAAATGCCATTTCATCTATTGGTTCCATTATTTCTC
TCACAAGAATTATCTTTTTTTTTATTTATCTTATGAGAAAGGCTTTCTTCCCAACGT
AAATCTTTGAGAACTTTAAATGTAACAACTTCAATTGAATGGTTCAAGCCCTACC
CCCTAGTG
PE4_5
ATTATTAGACAAGAAAGGGGGAAAAAAGAAGCTTTTGGACCTTAGGAATAATTTA
TGCTATAATAGCTATCGGATTATTAGGATTCGTAGTTTGAGCTCACCATATATTTA
CAGTAGATAGATGTAGATACCCGAGCTTACTTCACTTCAGCAACTATAATTATTG
CGGTTCCAACTGGGATTAAAGTCTTTAGATGACTAGCCACCTTTCATGGAACTCA
AATTTTAAATAGACCAGTAACCCTGTGAGCTTTAGGTTTTGTATTTTTATTTACAG
TAGGAGGATTAACAGGGGTAATTTTAGCTAATTCTTCAATTGATATTATTCTCCAC
GATACTTACTATGTAGTAGCTCATTTTCACTATGTTCTTTCTATAGGAGCTGTATT
TGCAATTATAGCCGGAATTGTCCAATGATTCCCATTATTTACCGGACTAACCTTA
AATAATTTTTTTTTAAAAACCCAATTTATTACTATATTTTTAGGTGTAAATTTAACAT
TTTTTCCTCAACACTTTTTAGGGCTAAGAGGAATGCCTCGGCGATATTCGGATTA
TCCAGATGCTTTCACTATCTGAAATGCCATTTCATCTATTGGTTCCATTATTTCTC
TCACAAGAATTATCTTTTTTTTTATTTATCTTATGAGAAAGGCTTTCTTCCCAACGT
AAATCTTTGAGAACTTTAAATGTAACAACTTCAATTGAATGGTTCAAGCCCTACC
CCCTAGTG
PE_12
ATTATTAGTCAAGAAAGGGGGAAAAAAGAACCTTTTGGACCTTAGGAATAATTTA
TGCTATAATAGCTATCGGATTATTAGGATTCGTAGTTTGAGCTCACCATATATTTA
CAGTAGATAGATGTAGATACCCGAGCTTACTTCACTTCAGCAACTATAATTATTG
CGGTTCCAACTGGGATTAAAGTCTTTAGATGACTAGCCACCTTTCATGGAACTCA
AATTTTAAATAGACCAGTAACCCTGTGAGCTTTAGGTTTTGTATTTTTATTTACAG
TAGGAGGATTAACAGGGGTAATTTTAGCTAATTCTTCAATTGATATTATTCTCCAC
GATACTTACTATGTAGTAGCTCATTTTCACTATGTTCTTTCTATAGGAGCTGTATT
TGCAATTATAGCCGGAATTGTCCAATGATTCCCATTATTTACCGGACTAACCTTA
AATAATTTTTTTTTAAAAACCCAATTTATTACTATATTTTTAGGTGTAAATTTAACAT
TTTTTCCTCAACACTTTTTAGGGCTAAGAGGAATGCCTCGGCGATATTCGGATTA
TCCAGATGCTTTCACTATCTGAAATGCCATTTCATCTATTGGTTCCATTATTTCTC
TCACAAGAATTATCTTTTTTTTTATTTATCTTATGAGAAAGGCTTTCTTCCCAACGT
AAATCTTTGAGAACTTTAAATGTAACAACTTCAATTGAATGGTTCAAGCCCTACC
CCCTAGTG
PE_13
ATTATTAGACAAGAAAGGGGGAAAAAAGAACCTTTTGGACCTTAGGAATAATTTA

TGCTATAATAGCTATCGGATTATTAGGATTCGTAGTTTGAGCTCACCATATATTTA
CAGTAGATAGATGTAGATACCCGAGCTTACTTCACTTCAGCAACTATAATTATTG
CGGTTCCAACTGGGATTAAAGTCTTTAGATGACTAGCCACCTTTCATGGAACTCA
AATTTTAAATAGACCAGTAACCCTGTGAGCTTTAGGTTTTGTATTTTTATTTACAG
TAGGAGGATTAACAGGGGTAATTTTAGCTAATTCTTCAATTGATATTATTCTCCAC
GATACTTACTATGTAGTAGCTCATTTTCACTATGTTCTTTCTATAGGAGCTGTATT
TGCAATTATAGCCGGAATTGTCCAATGATTCCCATTATTTACCGGACTAACCTTA
AATAATTTTTTTTTAAAAACCCAATTTATTACTATATTTTTAGGTGTAAATTTAACAT
TTTTTCCTCAACACTTTTTAGGGCTAAGAGGAATGCCTCGGCGATATTCGGATTA
TCCAGATGCTTTCACTATCTGAAATGCCATTTCATCTATTGGTTCCATTATTTCTC
TCACAAGAATTATCTTTTTTTTTATTTATCTTATGAGAAAGGCTTTCTTCCCAACGT
AAATCTTTGAGAACTTTAAATGTAACAACTTCAATTGAATGGTTCAAGCCCTACC
CCCTAGTG
PE_15
ATTATTAGACAAGAAAGGGGGAAAAAAGAACCTTTTGGACCTTAGGAATAATTTA
TGCTATAATACCTATCGGATTATTAGGATTCGTAGTTTGAGCTCACCATATATTTA
CAGTAGATAGATGTAGATACCCGAGCTTACTTCACTTCAGCAACTATAATTATTG
CGGTTCCAACTGGGATTAAAGTCTTTAGATGACTAGCCACCTTTCATGGAACTCA
AATTTTAAATAGACCAGTAACCCTGTGAGCTTTAGGTTTTGTATTTTTATTTACAG
TAGGAGGATTAACAGGGGTAATTTTAGCTAATTCTTCAATTGATATTATTCTCCAC
GATACTTACTATGTAGTAGCTCATTTTCACTATGTTCTTTCTATAGGAGCTGTATT
TGCAATTATAGCCGGAATTGTCCAATGATTCCCATTATTTACCGGACTAACCTTA
AATAATTTTTTTTTAAAAACCCAATTTATTACTATATTTTTAGGTGTAAATTTAACAT
TTTTTCCTCAACACTTTTTAGGGCTAAGAGGAATGCCTCGGCGATATTCGGATTA
TCCAGATGCTTTCACTATCTGAAATGCCATTTCATCCATTGGTTCCATTATTTCTC
TCACAAGAATTATCTTTTTTTTTATTTATCTTATGAGAAAGGCTTTCTTCCCAACGT
AAATCTGTGAGAACTTTAAATGTAACAACTTCAATTGAATGGTTCAAGCCCTACC
CCCTAGTG
PE_16
ATTATTAGACAAGAAAGGGGGAAAAAAGAAGCTTTTGGACCTTAGGAATAATTTA
TGCTATAATAGCTATCGGATTATTAGGATTCGTAGTTTGAGCTCACCATATATTTA
CAGTAGATAGATGTAGATACCCGAGCTTACTTCACTTCAGCAACTATAATTATTG
CGGTTCCAACTGGGATTAAAGTCTTTAGATGACTAGCCACCTTTCATGGAACTCA
AATTTTAAATAGACCAGTCACCCTGTGAGCTTTAGGTTTTGTATTTTTATTTACAG
TAGGAGGATTAACAGGGGTAATTTTAGCTAATTCTTCAATTGATATTATTCTCCAC
GATACTTACTATGTAGTAGCTCATTTTCACTATGTTCTTTCTATAGGAGCTGTATT
TGCAATTATAGCCGGAATTGTCCAATGATTCCCATTATTTACCGGACTAACCTTA
AATAATTTTTTTTTAAAAACCCAATTTATTACTATATTTTTAGGTGTAAATTTAACAT
TTTTTCCTCAACACTTTTTAGGGCTAAGAGGAATGCCTCGGCGATATTCGGATTA
TCCAGATGCTTTCACTATCTGAAATGCCATTTCATCTATTGGTTCCATTATTTCTC
TCACAAGAATTATCTTTTTTTTTATTTATCTTATGAGAAAGGCTTTCTTCCCAACGT
AAATCTTTGAGAACTTTAAATGTAACAACTTCAATTGAATGGTTCAAGCCCTACC
CCCTAGTG
PE_19
ATTATTAGACAAGAAAGGGGGAAAAAAGAAGCTTTTGGACCTTAGGAATAATTTA
TGCTATAATAGCTATCGGATTATTAGGATTCGTAGTTTGAGCTCACCATATATTTA
CAGTAGATAGATGTAGATACCCGAGCTTACTTCACTTCAGCAACTATAATTATTG
CGGTTCCAACTGGGATTAAAGTCTTTAGATGACTAGCCACCTTTCATGGAACTCA
AATTTTAAATAGACCAGTCACCCTGTGAGCTTTAGGTTTTGTATTTTTATTTACAG
TAGGAGGATTAACAGGGGTAATTTTAGCTAATTCTTCAATTGATATTATTCTCCAC

GATACTTACTATGTAGTAGCTCATTTTCACTATGTTCTTTCTATAGGAGCTGTATT
TGCAATTATAGCCGGAATTGTCCAATGATTCCCATTATTTACCGGACTAACCTTA
AATAATTTTTTTTTAAAAACCCAATTTATTACTATATTTTTAGGTGTAAATTTAACAT
TTTTTCCTCAACACTTTTTAGGGCTAAGAGGAATGCCTCGGCGATATTCGGATTA
TCCAGATGCTTTCACTATCTGAAATGCCATTTCATCCATTGGTTCCATTATTTCTC
TCACAAGAATTATCTTTTTTTTTATTTATCTTATGAGAAAGGCTTTCTTCCCAACGT
AAATCTTTGAGAACTTTAAATGTAACAACTTCAATTGAATGGTTCAAGCCCTACC
CCCTAGTG
PE_2
ATTATTAGACAAGAAAGGGGGAAAAAAGAAGCTTTTGGACCTTAGGAATAATTTA
TGCTATAATAGCTATCGGATTATTAGGATTCGTAGTTTGAGCTCACCATATATTTA
CAGTAGATAGATGTAGATACCCGAGCTTACTTCACTTCAGCAACTATAATTATTG
CGGTTCCAACTGGGATTAAAGTCTTTAGATGACTAGCCACCTTTCATGGAACTCA
AATTTTAAATAGACCAGTCACCCTGTGAGCTTTAGGTTTTGTATTTTTATTTACAG
TAGGAGGATTAACAGGGGTAATTTTAGCTAATTCTTCAATTGATATTATTCTCCAC
GATACTTACTATGTAGTAGCTCATTTTCACTATGTTCTTTCTATAGGAGCTGTATT
TGCAATTATAGCCGGAATTGTCCAATGATTCCCATTATTTACCGGACTAACCTTA
AATAATTTTTTTTTAAAAACCCAATTTATTACTATATTTTTAGGTGTAAATTTAACAT
TTTTTCCTCAACACTTTTTAGGGCTAAGAGGAATGCCTCGGCGATATTCGGATTA
TCCAGATGCTTTCACTATCTGAAATGCCATTTCATCTATTGGTTCCATTATTTCTC
TCACAAGAATTATCTTTTTTTTTATTTATCTTATGAGAAAGGCTTTCTTCCCAACGT
AAATCTTTGAGAACTTTAAATGTAACAACTTCAATTGAATGGTTCAAGCCCTACC
CCCTAGTG
PE_23
ATTATTAGACAAGAAAGGGGGAAAAAAGAAGCTTTTGGACCTTAGGAATAATTTA
TGCTATAATAGCTATCGGATTATTAGGATTCGTAGTTTGAGCTCACCATATATTTA
CAGTAGATAGATGTAGATACCCGAGCTTACTTCACTTCAGCAACTATAATTATTG
CGGTTCCAACTGGGATTAAAGTCTTTAGATGACTAGCCACCTTTCATGGAACTCA
AATTTTAAATAGACCAGTAACCCTGTGAGCTTTAGGTTTTGTATTTTTATTTACAG
TAGGAGGATTAACAGGGGTAATTTTAGCTAATTCTTCAATTGATATTATTCTCCAC
GATACTTACTATGTAGTAGCTCATTTTCACTATGTTCTTTCTATAGGAGCTGTATT
TGCAATTATAGCCGGAATTGTCCAATGATTCCCATTATTTACCGGACTAACCTTA
AATAATTTTTTTTTAAAAACCCAATTTATTACTATATTTTTAGGTGTAAATTTAACAT
TTTTTCCTCAACACTTTTTAGGGCTAAGAGGAATGCCTCGGCGATATTCGGATTA
TCCAGATGCTTTCACTATCTGAAATGCCATTTCATCTATTGGTTCCATTATTTCTC
TCACAAGAATTATCTTTTTTTTTATTTATCTTATGAGAAAGGCTTTCTTCCCAACGT
AAATCTTTGAGAACTTTAAATGTAACAACTTCAATTGAATGGTTCAAGCCCTACC
CCCTAGTG
PE_29
ATTATTAGACAAGAAAGGGGGAAAAAAGAAGCTTTTGGACCTTAGGAATAATTTA
TGCTATAATAGCTATCGGATTATTAGGATTCGTAGTTTGAGCTCACCATATATTTA
CAGTAGATAGATGTAGATACCCGAGCTTACTTCACTTCAGCAACTATAATTATTG
CGGTTCCAACTGGGATTAAAGTCTTTAGATGACTAGCCACCTTTCATGGAACTCA
AATTTTAAATAGACCAGTCACCCTGTGAGCTTTAGGTTTTGTATTTTTATTTACAG
TAGGAGGATTAACAGGGGTAATTTTAGCTAATTCTTCAATTGATATTATTCTCCAC
GATACTTACTATGTAGTAGCTCATTTTCACTATGTTCTTTCTATAGGAGCTGTATT
TGCAATTATAGCCGGAATTGTCCAATGATTCCCATTATTTACCGGACTAACCTTA
AATAATTTTTTTTTAAAAACCCAATTTATTACTATATTTTTAGGTGTAAATTTAACAT
TTTTTCCTCAACACTTTTTAGGGCTAAGAGGAATGCCTCGGCGATATTCGGATTA
TCCAGATGCTTTCACTATCTGAAATGCCATTTCATCTATTGGTTCCATTATTTCTC

TCACAAGAATTATCTTTTTTTTATTTATCTTATGAGAAAGGCTTTCTTCCCAACGT
AAATCTTTGAGAACTTTAAATGTAACAACTTCAATTGAATGGTTCAAGCCCTACC
CCCTAGTG
PE_6
ATTATTAGACAAGAAAGGGGGAAAAAAGAAGCTTTTGGACCTTAGGAATAATTTA
TGCTATAATAGCTATCGGATTATTAGGATTCGTAGTTTGAGCTCACCATATATTTA
CAGTAGATAGATGTAGATACCCGAGCTTACTTCACTTCAGCAACTATAATTATTG
CGGTTCCAACTGGGATTAAAGTCTTTAGATGACTAGCCACCTTTCATGGAACTCA
AATTTTAAATAGACCAGTAACCCTGTGAGCTTTAGGTTTTGTATTTTTATTTACAG
TAGGAGGATTAACAGGGGTAATTTTAGCTAATTCTTCAATTGATATTATTCTCCAC
GATACTTACTATGTAGTAGCTCATTTTCACTATGTTCTTTCTATAGGAGCTGTATT
TGCAATTATAGCCGGAATTGTCCAATGATTCCCATTATTTACCGGACTAACCTTA
AATAATTTTTTTTTAAAAACCCAATTTATTACTATATTTTTAGGTGTAAATTTAACAT
TTTTTCCTCAACACTTTTTAGGGCTAAGAGGAATGCCTCGGCGATATTCGGATTA
TCCAGATGCTTTCACTATCTGAAATGCCATTTCATCTATTGGTTCCATTATTTCTC
TCACAAGAATTATCTTTTTTTTATTTATCTTATGAGAAAGGCTTTCTTCCCAACGT
AAATCTTTGAGAACTTTAAATGTAACAACTTCAATTGAATGGTTCAAGCCCTACC
CCCTAGTG
PE_8
ATTATTAGACAAGAAAGGGGGAAAAAAGAAGCTTTTGGACCTTAGGAATAATTTA
TGCTATAATAGCTATCGGATTATTAGGATTCGTAGTTTGAGCTCACCATATATTTA
CAGTAGATAGATGTAGATACCCGAGCTTACTTCACTTCAGCAACTATAATTATTG
CGGTTCCAACTGGGATTAAAGTCTTTAGATGACTAGCCACCTTTCATGGAACTCA
AATTTTAAATAGACCAGTAACCCTGTGAGCTTTAGGTTTTGTATTTTTATTTACAG
TAGGAGGATTAACAGGGGTAATTTTAGCTAATTCTTCAATTGATATTATTCTCCAC
GATACTTACTATGTAGTAGCTCATTTTCACTATGTTCTTTCTATAGGAGCTGTATT
TGCAATTATAGCCGGAATTGTCCAATGATTCCCATTATTTACCGGACTAACCTTA
AATAATTTTTTTTTAAAAACCCAATTTATTACTATATTTTTAGGTGTAAATTTAACAT
TTTTTCCTCAACACTTTTTAGGGCTAAGAGGAATGCCTCGGCGATATTCGGATTA
TCCAGATGCTTTCACTATCTGAAATGCCATTTCATCTATTGGTTCCATTATTTCTC
TCACAAGAATTATCTTTTTTTTATTTATCTTATGAGAAAGGCTTTCTTCCCAACGT
AAATCTTTGAGAACTTTAAATGTAACAACTTCAATTGAATGGTTCAAGCCCTACC
CCCTAGTG
CL1_1
ATTATTAGACAAGAAAGGGGGAAAAAAGAAGCTTTTGGACCTTAGGAATAATTTA
TGCTATAATAGCTATCGGATTATTAGGATTCGTAGTTTGAGCTCACCATATATTTA
CAGTAGATAGATGTAGATACCCGAGCTTACTTCACTTCAGCAACTATAATTATTG
CGGTTCCAACTGGGATTAAAGTCTTTAGATGACTAGCCACCTTTCATGGAACTCA
AATTTTAAATAGACCAGTAACCCTGTGAGCTTTAGGTTTTGTATTTTTATTTACAG
TAGGAGGATTAACAGGGGTAATTTTAGCTAATTCTTCAATTGATATTATTCTCCAC
GATACTTACTATGTAGTAGCTCATTTTCACTATGTTCTTTCTATAGGAGCTGTATT
TGCAATTATAGCCGGAATTGTCCAATGATTCCCATTATTTACCGGACTAACCTTA
AATAATTTTTTTTTAAAAACCCAATTTATTACTATATTTTTAGGTGTAAATTTAACAT
TTTTTCCTCAACACTTTTTAGGGCTAAGAGGAATGCCTCGGCGATATTCGGATTA
TCCAGATGCTTTCACTATCTGAAATGCCATTTCATCTATTGGTTCCATTATTTCTC
TCACAAGAATTATCTTTTTTTTATTTATCTTATGAGAAAGGCTTTCTTCCCAACGT
AAATCTTTGAGAACTTTAAATGTAACAACTTCAATTGAATGGTTCAAGCCCTACC
CCCTAGTG
CL1_2
ATTATTAGACAAGAAAGGGGGAAAAAAGAAGCTTTTGGACCTTAGGAATAATTTA

TGCTATAATAGCTATCGGATTATTAGGATTCGTAGTTTGAGCTCACCATATATTTA
CAGTAGATAGATGTAGATACCCGAGCTTACTTCACTTCAGCAACTATAATTATTG
CGGTTCCAACTGGGATTAAAGTCTTTAGATGACTAGCCACCTTTCATGGAACTCA
AATTTTAAATAGACCAGTAACCCTGTGAGCTTTAGGTTTTGTATTTTTATTTACAG
TAGGAGGATTAACAGGGGTAATTTTAGCTAATTCTTCAATTGATATTATTCTCCAC
GATACTTACTATGTAGTAGCTCATTTTCACTATGTTCTTTCTATAGGAGCTGTATT
TGCAATTATAGCCGGAATTGTCCAATGATTCCCATTATTTACCGGACTAACCTTA
AATAATTTTTTTTTAAAAACCCAATTTATTACCATATTTTTAGGTGTAAATTTAACA
TTTTTTCCTCAACACTTTTTAGGGCTAAGAGGAATGCCTCGGCGATATTCGGATT
ATCCAGATGCTTTCACTATCTGAAATGCCATTTCATCTATTGGTTCCATTATTTCT
CTCACAAGAATTATCTTTTTTTTATTTATCTTATGAGAAAGGCTTTCTTCCCAACG
TAAATCTTTGAGAACTTTAAATGTAACAACTTCAATTGAATGGTTCAAGCCCTACC
CCCTAGTG
CL1_3
ATTATTAGACAAGAAAGGGGGAAAAAAGAAGCTTTTGGACCTTAGGAATAATTTA
TGCTATAATAGCTATCGGATTATTAGGATTCGTAGTTTGAGCTCACCATATATTTA
CAGTAGATAGATGTAGATACCCGAGCTTACTTCACTTCAGCAACTATAATTATTG
CGGTTCCAACTGGGATTAAAGTCTTTAGATGACTAGCCACCTTTCATGGAACTCA
AATTTTAAATAGACCAGTAACCCTGTGAGCTTTAGGTTTTGTATTTTTATTTACAG
TAGGAGGATTAACAGGGGTAATTTTAGCTAATTCTTCAATTGATATTATTCTCCAC
GATACTTACTATGTAGTAGCTCATTTTCACTATGTTCTTTCTATAGGAGCTGTATT
TGCAATTATAGCCGGAATTGTCCAATGATTCCCATTATTTACCGGACTAACCTTA
AATAATTTTTTTTTAAAAACCCAATTTATTACTATATTTTTAGGTGTAAATTTAACAT
TTTTTCCTCAACACTTTTTAGGGCTAAGAGGAATGCCTCGGCGATATTCGGATTA
TCCAGATGCTTTCACTATCTGAAATGCCATTTCATCTATTGGTTCCATTATTTCTC
TCACAAGAATTATCTTTTTTTTATTTATCTTATGAGAAAGGCTTTCTTCCCAACGT
AAATCTTTGAGAACTTTAAATGTAACAACTTCAATTGAATGGTTCAAGCCCTACC
CCCTAGTG
CL1_4
ATTATTAGACAAGAAAGGGGGAAAAAAGAAGCTTTTGGACCTTAGGAATAATTTA
TGCTATAATAGCTATCGGATTATTAGGATTCGTAGTTTGAGCTCACCATATATTTA
CAGTAGATAGATGTAGATACCCGAGCTTACTTCACTTCAGCAACTATAATTATTG
CGGTTCCAACTGGGATTAAAGTCTTTAGATGACTAGCCACCTTTCATGGAACTCA
AATTTTAAATAGACCAGTAACCCTGTGAGCTTTAGGTTTTGTATTTTTATTTACAG
TAGGAGGATTAACAGGGGTAATTTTAGCTAATTCTTCAATTGATATTATTCTCCAC
GATACTTACTATGTAGTAGCTCATTTTCACTATGTTCTTTCTATAGGAGCTGTATT
TGCAATTATAGCCGGAATTGTCCAATGATTCCCATTATTTACCGGACTAACCTTA
AATAATTTTTTTTTAAAAACCCAATTTATTACTATATTTTTAGGTGTAAATTTAACAT
TTTTTCCTCAACACTTTTTAGGGCTAAGAGGAATGCCTCGGCGATATTCGGATTA
TCCAGATGCTTTCACTATCTGAAATGCCATTTCATCTATTGGTTCCATTATTTCTC
TCACAAGAATTATCTTTTTTTTATTTATCTTATGAGAAAGGCTTTCTTCCCAACGT
AAATCTTTGAGAACTTTAAATGTAACAACTTCAATTGAATGGTTCAAGCCCTACC
CCCTAGTG
CL1_5
ATTATTAGACAAGAAAGGGGGAAAAAAGAAGCTTTTGGACCTTAGGAATAATTTA
TGCTATAATAGCTATCGGATTATTAGGATTCGTAGTTTGAGCTCACCATATATTTA
CAGTAGATAGATGTAGATACCCGAGCTTACTTCACTTCAGCAACTATAATTATTG
CGGTTCCAACTGGGATTAAAGTCTTTAGATGACTAGCCACCTTTCATGGAACTCA
AATTTTAAATAGACCAGTAACCCTGTGAGCTTTAGGTTTTGTATTTTTATTTACAG
TAGGAGGATTAACAGGGGTAATTTTAGCTAATTCTTCAATTGATATTATTCTCCAC

GATACTTACTATGTAGTAGCTCATTTTCACTATGTTCTTTCTATAGGAGCTGTATT
TGCAATTATAGCCGGAATTGTCCAATGATTCCCATTATTTACCGGACTAACCTTA
AATAATTTTTTTTTAAAAACCCAATTTATTACCATATTTTTAGGTGTAAATTTAACA
TTTTTTCCTCAACACTTTTTAGGGCTAAGAGGAATGCCTCGGCGATATTCGGATT
ATCCAGATGCTTTCACTATCTGAAATGCCATTTCATCTATTGGTTCCATTATTTCT
CTCACAAGAATTATCTTTTTTTTTATTTATCTTATGAGAAAGGCTTTCTTCCCAACG
TAAATCTTTGAGAACTTTAAATGTAACAACTTCAATTGAATGGTTCAAGCCCTACC
CCCTAGTG
CL1_6
ATTATTAGACAAGAAAGGGGGAAAAAAGAAGCTTTTGGACCTTAGGAATAATTTA
TGCTATAATAGCTATCGGATTATTAGGATTCGTAGTTTGAGCTCACCATATATTTA
CAGTAGATAGATGTAGATACCCGAGCTTACTTCACTTCAGCAACTATAATTATTG
CGGTTCCAACTGGGATTAAAGTCTTTAGATGACTAGCCACCTTTCATGGAACTCA
AATTTTAAATAGACCAGTAACCCTGTGAGCTTTAGGTTTTGTATTTTTATTTACAG
TAGGAGGATTAACAGGGGTAATTTTAGCTAATTCTTCAATTGATATTATTCTCCAC
GATACTTACTATGTAGTAGCTCATTTTCACTATGTTCTTTCTATAGGAGCTGTATT
TGCAATTATAGCCGGAATTGTCCAATGATTCCCATTATTTACCGGACTAACCTTA
AATAATTTTTTTTTAAAAACCCAATTTATTACCATATTTTTAGGTGTAAATTTAACA
TTTTTTCCTCAACACTTTTTAGGGCTAAGAGGAATGCCTCGGCGATATTCGGATT
ATCCAGATGCTTTCACTATCTGAAATGCCATTTCATCTATTGGTTCCATTATTTCT
CTCACAAGAATTATCTTTTTTTTTATTTATCTTATGAGAAAGGCTTTCTTCCCAACG
TAAATCTTTGAGAACTTTAAATGTAACAACTTCAATTGAATGGTTCAAGCCCTACC
CCCTAGTG
CL1_7
ATTATTAGACAAGAAAGGGGGAAAAAAGAAGCTTTTGGACCTTAGGAATAATTTA
TGCTATAATAGCTATCGGATTATTAGGATTCGTAGTTTGAGCTCACCATATATTTA
CAGTAGATAGATGTAGATACCCGAGCTTACTTCACTTCAGCAACTATAATTATTG
CGGTTCCAACTGGGATTAAAGTCTTTAGATGACTAGCCACCTTTCATGGAACTCA
AATTTTAAATAGACCAGTAACCCTGTGAGCTTTAGGTTTTGTATTTTTATTTACAG
TAGGAGGATTAACAGGGGTAATTTTAGCTAATTCTTCAATTGATATTATTCTCCAC
GATACTTACTATGTAGTAGCTCATTTTCACTATGTTCTTTCTATAGGAGCTGTATT
TGCAATTATAGCCGGAATTGTCCAATGATTCCCATTATTTACCGGACTAACCTTA
AATAATTTTTTTTTAAAAACCCAATTTATTACTATATTTTTAGGTGTAAATTTAACAT
TTTTTCCTCAACACTTTTTAGGGCTAAGAGGAATGCCTCGGCGATATTCGGATTA
TCCAGATGCTTTCACTATCTGAAATGCCATTTCATCTATTGGTTCCATTATTTCTC
TCACAAGAATTATCTTTTTTTTTATTTATCTTATGAGAAAGGCTTTCTTCCCAACGT
AAATCTTTGAGAACTTTAAATGTAACAACTTCAATTGAATGGTTCAAGCCCTACC
CCCTAGTG
CL1_8
ATTATTAGACAAGAAAGGGGGAAAAAAGAAGCTTTTGGACCTTAGGAATAATTTA
TGCTATAATAGCTATCGGATTATTAGGATTCGTAGTTTGAGCTCACCATATATTTA
CAGTAGATAGATGTAGATACCCGAGCTTACTTCACTTCAGCAACTATAATTATTG
CGGTTCCAACTGGGATTAAAGTCTTTAGATGACTAGCCACCTTTCATGGAACTCA
AATTTTAAATAGACCAGTAACCCTGTGAGCTTTAGGTTTTGTATTTTTATTTACAG
TAGGAGGATTAACAGGGGTAATTTTAGCTAATTCTTCAATTGATATTATTCTCCAC
GATACTTACTATGTAGTAGCTCATTTTCACTATGTTCTTTCTATAGGAGCTGTATT
TGCAATTATAGCCGGAATTGTCCAATGATTCCCATTATTTACCGGACTAACCTTA
AATAATTTTTTTTTAAAAACCCAATTTATTACCATATTTTTAGGTGTAAATTTAACA
TTTTTTCCTCAACACTTTTTAGGGCTAAGAGGAATGCCTCGGCGATATTCGGATT
ATCCAGATGCTTTCACTATCTGAAATGCCATTTCATCTATTGGTTCCATTATTTCT

CTCACAAGAATTATCTTTTTTTTATTTATCTTATGAGAAAGGCTTTCTTCCCAACG
TAAATCTTTGAGAACTTTAAATGTAACAACTTCAATTGAATGGTTCAAGCCCTACC
CCCTAGTG
CL1_9
ATTATTAGACAAGAAAGGGGGAAAAAAGAAGCTTTTGGACCTTAGGAATAATTTA
TGCTATAATAGCTATCGGATTATTAGGATTCGTAGTTTGAGCTCACCATATATTTA
CAGTAGATAGATGTAGATACCCGAGCTTACTTCACTTCAGCAACTATAATTATTG
CGGTTCCAACTGGGATTAAAGTCTTTAGATGACTAGCCACCTTTCATGGAACTCA
AATTTTAAATAGACCAGTAACCCTGTGAGCTTTAGGTTTTGTATTTTTATTTACAG
TAGGAGGATTAACAGGGGTAATTTTAGCTAATTCTTCAATTGATATTATTCTCCAC
GATACTTACTATGTAGTAGCTCATTTTCACTATGTTCTTTCTATAGGAGCTGTATT
TGCAATTATAGCCGGAATTGTCCAATGATTCCCATTATTTACCGGACTAACCTTA
AATAATTTTTTTTTAAAAACCCAATTTATTACTATATTTTTAGGTGTAAATTTAACAT
TTTTTCCTCAACACTTTTTAGGGCTAAGAGGAATGCCTCGGCGATATTCGGATTA
TCCAGATGCTTTCACTATCTGAAATGCCATTTCATCTATTGGTTCCATTATTTCTC
TCACAAGAATTATCTTTTTTTTATTTATCTTATGAGAAAGGCTTTCTTCCCAACGT
AAATCTTTGAGAACTTTAAATGTAACAACTTCAATTGAATGGTTCAAGCCCTACC
CCCTAGTG
CL2_3
ATTATTAGACAAGAAAGGGGGAAAAAAGAAGCTTTTGGACCTTAGGAATAATTTA
TGCTATAATAGCTATCGGATTATTAGGATTCGTAGTTTGAGCTCACCATATATTTA
CAGTAGATAGATGTAGATACCCGAGCTTACTTCACTTCAGCAACTATAATTATTG
CGGTTCCAACTGGGATTAAAGTCTTTAGATGACTAGCCACCTTTCATGGAACTCA
AATTTTAAATAGACCAGTAACCCTGTGAGCTTTAGGTTTTGTATTTTTATTTACAG
TAGGAGGATTAACAGGGGTAATTTTAGCTAATTCTTCAATTGATATTATTCTCCAC
GATACTTACTATGTAGTAGCTCATTTTCACTATGTTCTTTCTATAGGAGCTGTATT
TGCAATTATAGCCGGAATTGTCCAATGATTCCCATTATTTACCGGACTAACCTTA
AATAATTTTTTTTTAAAAACCCAATTTATTACTATATTTTTAGGTGTAAATTTAACAT
TTTTTCCTCAACACTTTTTAGGGCTAAGAGGAATGCCTCGGCGATATTCGGATTA
TCCAGATGCTTTCACTATCTGAAATGCCATTTCATCTATTGGTTCCATTATTTCTC
TCACAAGAATTATCTTTTTTTTATTTATCTTATGAGAAAGGCTTTCTTCCCAACGT
AAATCTTTGAGAACTTTAAATGTAACAACTTCAATTGAATGGTTCAAGCCCTACC
CCCTAGTG
CL2_4
ATTATTAGACAAGAAAGGGGGAAAAAAGAAGCTTTTGGACCTTAGGAATAATTTA
TGCTATAATAGCTATCGGATTATTAGGATTCGTAGTTTGAGCTCACCATATATTTA
CAGTAGATAGATGTAGATACCCGAGCTTACTTCACTTCAGCAACTATAATTATTG
CGGTTCCAACTGGGATTAAAGTCTTTAGATGACTAGCCACCTTTCATGGAACTCA
AATTTTAAATAGACCAGTAACCCTGTGAGCTTTAGGTTTTGTATTTTTATTTACAG
TAGGAGGATTAACAGGGGTAATTTTAGCTAATTCTTCAATTGATATTATTCTCCAC
GATACTTACTATGTAGTAGCTCATTTTCACTATGTTCTTTCTATAGGAGCTGTATT
TGCAATTATAGCCGGAATTGTCCAATGATTCCCATTATTTACCGGACTAACCTTA
AATAATTTTTTTTTAAAAACCCAATTTATTACTATATTTTTAGGTGTAAATTTAACAT
TTTTTCCTCAACACTTTTTAGGGCTAAGAGGAATGCCTCGGCGATATTCGGATTA
TCCAGATGCTTTCACTATCTGAAATGCCATTTCATCTATTGGTTCCATTATTTCTC
TCACAAGAATTATCTTTTTTTTATTTATCTTATGAGAAAGGCTTTCTTCCCAACGT
AAATCTTTGAGAACTTTAAATGTAACAACTTCAATTGAATGGTTCAAGCCCTACC
CCCTAGTG
CL2_5
ATTATTAGACAAGAAAGGGGGAAAAAAGAAGCTTTTGGACCTTAGGAATAATTTA

TGCTATAATAGCTATCGGATTATTAGGATTCGTAGTTTGAGCTCACCATATATTTA
CAGTAGATAGATGTAGATACCCGAGCTTACTTCACTTCAGCAACTATAATTATTG
CGGTTCCAACTGGGATTAAAGTCTTTAGATGACTAGCCACCTTTCATGGAACTCA
AATTTTAAATAGACCAGTAACCCTGTGAGCTTTAGGTTTTGTATTTTTATTTACAG
TAGGAGGATTAACAGGGGTAATTTTAGCTAATTCTTCAATTGATATTATTCTCCAC
GATACTTACTATGTAGTAGCTCATTTTCACTATGTTCTTTCTATAGGAGCTGTATT
TGCAATTATAGCCGGAATTGTCCAATGATTCCCATTATTTACCGGACTAACCTTA
AATAATTTTTTTTTAAAAACCCAATTTATTACTATATTTTTAGGTGTAAATTTAACAT
TTTTTCCTCAACACTTTTTAGGGCTAAGAGGAATGCCTCGGCGATATTCGGATTA
TCCAGATGCTTTCACTATCTGAAATGCCATTTCATCTATTGGTTCCATTATTTCTC
TCACAAGAATTATCTTTTTTTTTATTTATCTTATGAGAAAGGCTTTCTTCCCAACGT
AAATCTTTGAGAACTTTAAATGTAACAACTTCAATTGAATGGTTCAAGCCCTACC
CCCTAGTG
CL2_6
ATTATTAGACAAGAAAGGGGGAAAAAAGAAGCTTTTGGACCTTAGGAATAATTTA
TGCTATAATAGCTATCGGATTATTAGGATTCGTAGTTTGAGCTCACCATATATTTA
CAGTAGATAGATGTAGATACCCGAGCTTACTTCACTTCAGCAACTATAATTATTG
CGGTTCCAACTGGGATTAAAGTCTTTAGATGACTAGCCACCTTTCATGGAACTCA
AATTTTAAATAGACCAGTAACCCTGTGAGCTTTAGGTTTTGTATTTTTATTTACAG
TAGGAGGATTAACAGGGGTAATTTTAGCTAATTCTTCAATTGATATTATTCTCCAC
GATACTTACTATGTAGTAGCTCATTTTCACTATGTTCTTTCTATAGGAGCTGTATT
TGCAATTATAGCCGGAATTGTCCAATGATTCCCATTATTTACCGGACTAACCTTA
AATAATTTTTTTTTAAAAACCCAATTTATTACTATATTTTTAGGTGTAAATTTAACAT
TTTTTCCTCAACACTTTTTAGGGCTAAGAGGAATGCCTCGGCGATATTCGGATTA
TCCAGATGCTTTCACTATCTGAAATGCCATTTCATCTATTGGTTCCATTATTTCTC
TCACAAGAATTATCTTTTTTTTTATTTATCTTATGAGAAAGGCTTTCTTCCCAACGT
AAATCTTTGAGAACTTTAAATGTAACAACTTCAATTGAATGGTTCAAGCCCTACC
CCCTAGTG
CL2_7
ATTATTAGACAAGAAAGGGGGAAAAAAGAAGCTTTTGGACCTTAGGAATAATTTA
TGCTATAATAGCTATCGGATTATTAGGATTCGTAGTTTGAGCTCACCATATATTTA
CAGTAGATAGATGTAGATACCCGAGCTTACTTCACTTCAGCAACTATAATTATTG
CGGTTCCAACTGGGATTAAAGTCTTTAGATGACTAGCCACCTTTCATGGAACTCA
AATTTTAAATAGACCAGTAACCCTGTGAGCTTTAGGTTTTGTATTTTTATTTACAG
TAGGAGGATTAACAGGGGTAATTTTAGCTAATTCTTCAATTGATATTATTCTCCAC
GATACTTACTATGTAGTAGCTCATTTTCACTATGTTCTTTCTATAGGAGCTGTATT
TGCAATTATAGCCGGAATTGTCCAATGATTCCCATTATTTACCGGACTAACCTTA
AATAATTTTTTTTTAAAAACCCAATTTATTACTATATTTTTAGGTGTAAATTTAACAT
TTTTTCCTCAACACTTTTTAGGGCTAAGAGGAATGCCTCGGCGATATTCGGATTA
TCCAGATGCTTTCACTATCTGAAATGCCATTTCATCTATTGGTTCCATTATTTCTC
TCACAAGAATTATCTTTTTTTTTATTTATCTTATGAGAAAGGCTTTCTTCCCAACGT
AAATCTTTGAGAACTTTAAATGTAACAACTTCAATTGAATGGTTCAAGCCCTACC
CCCTAGTG
CL2_8
ATTATTAGACAAGAAAGGGGGAAAAAAGAAGCTTTTGGACCTTAGGAATAATTTA
TGCTATAATAGCTATCGGATTATTAGGATTCGTAGTTTGAGCTCACCATATATTTA
CAGTAGATAGATGTAGATACCCGAGCTTACTTCACTTCAGCAACTATAATTATTG
CGGTTCCAACTGGGATTAAAGTCTTTAGATGACTAGCCACCTTTCATGGAACTCA
AATTTTAAATAGACCAGTAACCCTGTGAGCTTTAGGTTTTGTATTTTTATTTACAG
TAGGAGGATTAACAGGGGTAATTTTAGCTAATTCTTCAATTGATATTATTCTCCAC

GATACTTACTATGTAGTAGCTCATTTTCACTATGTTCTTTCTATAGGAGCTGTATT
TGCAATTATAGCCGGAATTGTCCAATGATTCCCATTATTTACCGGACTAACCTTA
AATAATTTTTTTTTAAAAACCCAATTTATTACTATATTTTTAGGTGTAAATTTAACAT
TTTTTCCTCAACACTTTTTAGGGCTAAGAGGAATGCCTCGGCGATATTCGGATTA
TCCAGATGCTTTCACTATCTGAAATGCCATTTCATCTATTGGTTCCATTATTTCTC
TCACAAGAATTATCTTTTTTTTTATTTATCTTATGAGAAAGGCTTTCTTCCCAACGT
AAATCTTTGAGAACTTTAAATGTAACAACTTCAATTGAATGGTTCAAGCCCTACC
CCCTAGTG
CL2_1
ATTATTAGACAAGAAAGGGGGAAAAAAGAAGCTTTTGGACCTTAGGAATAATTTA
TGCTATAATAGCTATCGGATTATTAGGATTCGTAGTTTGAGCTCACCATATATTTA
CAGTAGATAGATGTAGATACCCGAGCTTACTTCACTTCAGCAACTATAATTATTG
CGGTTCCAACTGGGATTAAAGTCTTTAGATGACTAGCCACCTTTCATGGAACTCA
AATTTTAAATAGACCAGTAACCCTGTGAGCTTTAGGTTTTGTATTTTTATTTACAG
TAGGAGGATTAACAGGGGTAATTTTAGCTAATTCTTCAATTGATATTATTCTCCAC
GATACTTACTATGTAGTAGCTCATTTTCACTATGTTCTTTCTATAGGAGCTGTATT
TGCAATTATAGCCGGAATTGTCCAATGATTCCCATTATTTACCGGACTAACCTTA
AATAATTTTTTTTTAAAAACCCAATTTATTACTATATTTTTAGGTGTAAATTTAACAT
TTTTTCCTCAACACTTTTTAGGGCTAAGAGGAATGCCTCGGCGATATTCGGATTA
TCCAGATGCTTTCACTATCTGAAATGCCATTTCATCTATTGGTTCCATTATTTCTC
TCACAAGAATTATCTTTTTTTTTATTTATCTTATGAGAAAGGCTTTCTTCCCAACGT
AAATCTTTGAGAACTTTAAATGTAACAACTTCAATTGAATGGTTCAAGCCCTACC
CCCTAGTG
CL2_2
ATTATTAGACAAGAAAGGGGGAAAAAAGAAGCTTTTGGACCTTAGGAATAATTTA
TGCTATAATAGCTATCGGATTATTAGGATTCGTAGTTTGAGCTCACCATATATTTA
CAGTAGATAGATGTAGATACCCGAGCTTACTTCACTTCAGCAACTATAATTATTG
CGGTTCCAACTGGGATTAAAGTCTTTAGATGACTAGCCACCTTTCATGGAACTCA
AATTTTAAATAGACCAGTAACCCTGTGAGCTTTAGGTTTTGTATTTTTATTTACAG
TAGGAGGATTAACAGGGGTAATTTTAGCTAATTCTTCAATTGATATTATTCTCCAC
GATACTTACTATGTAGTAGCTCATTTTCACTATGTTCTTTCTATAGGAGCTGTATT
TGCAATTATAGCCGGAATTGTCCAATGATTCCCATTATTTACCGGACTAACCTTA
AATAATTTTTTTTTAAAAACCCAATTTATTACTATATTTTTAGGTGTAAATTTAACAT
TTTTTCCTCAACACTTTTTAGGGCTAAGAGGAATGCCTCGGCGATATTCGGATTA
TCCAGATGCTTTCACTATCTGAAATGCCATTTCATCTATTGGTTCCATTATTTCTC
TCACAAGAATTATCTTTTTTTTTATTTATCTTATGAGAAAGGCTTTCTTCCCAACGT
AAATCTTTGAGAACTTTAAATGTAACAACTTCAATTGAATGGTTCAAGCCCTACC
CCCTAGTG
EQ1_3
ATTATTAGACAAGAAAGGGGGAAAAAAGAAGCTTTTGGACCTTAGGAATAATTTA
TGCTATAATAGCTATCGGATTATTAGGATTCGTAGTTTGAGCTCACCATATATTTA
CAGTAGATAGATGTAGATACCCGAGCTTACTTCACTTCAGCAACTATAATTATTG
CGGTTCCAACTGGGATTAAAGTCTTTAGATGACTAGCCACCTTTCATGGAACTCA
AATTTTAAATAGACCAGTAACCCTGTGAGCTTTAGGTTTTGTATTTTTATTTACAG
TAGGAGGATTAACAGGGGTAATTTTAGCTAATTCTTCAATTGATATTATTCTCCAC
GATACTTACTATGTAGTAGCTCATTTTCACTATGTTCTTTCTATAGGAGCTGTATT
TGCAATTATAGCCGGAATTGTCCAATGATTCCCATTATTTACCGGACTAACCTTA
AATAATTTTTTTTTAAAAACCCAATTTATTACTATATTTTTAGGTGTAAATTTAACAT
TTTTTCCTCAACACTTTTTAGGGCTAAGAGGAATGCCTCGGCGATATTCGGATTA
TCCAGATGCTTTCACTATCTGAAATGCCATTTCATCTATTGGTTCCATTATTTCTC

TCACAAGAATTATCTTTTTTTTTATTTATCTTATGAGAAAGGCTTTCTTCCCAACGT
AAATCTTTGAGAACTTTAAATGTAACAACTTCAATTGAATGGTTCAAGCCCTACC
CCCTAGTG
EQ1_6
ATTATTAGACAAGAAAGGGGGAAAAAAGAAGCTTTTGGACCTTAGGAATAATTTA
TGCTATAATAGCTATCGGATTATTAGGATTCGTAGTTTGAGCTCACCATATATTTA
CAGTAGATAGATGTAGATACCCGAGCTTACTTCACTTCAGCAACTATAATTATTG
CGGTTCCAACTGGGATTAAAGTCTTTAGATGACTAGCCACCTTTCATGGAACTCA
AATTTTAAATAGACCAGTAACCCTGTGAGCTTTAGGTTTTGTATTTTTATTTACAG
TAGGAGGATTAACAGGGGTAATTTTAGCTAATTCTTCAATTGATATTATTCTCCAC
GATACTTACTATGTAGTAGCTCATTTTCACTATGTTCTTTCTATAGGAGCTGTATT
TGCAATTATAGCCGGAATTGTCCAATGATTCCCATTATTTACCGGACTAACCTTA
AATAATTTTTTTTTAAAAACCCAATTTATTACTATATTTTTAGGTGTAAATTTAACAT
TTTTTCCTCAACACTTTTTAGGGCTAAGAGGAATGCCTCGGCGATATTCGGATTA
TCCAGATGCTTTCACTATCTGAAATGCCATTTCATCTATTGGTTCCATTATTTCTC
TCACAAGAATTATCTTTTTTTTTATTTATCTTATGAGAAAGGCTTTCTTCCCAACGT
AAATCTTTGAGAACTTTAAATGTAACAACTTCAATTGAATGGTTCAAGCCCTACC
CCCTAGTG
EQ1_7
ATTATTAGACAAGAAAGGGGGAAAAAAGAAGCTTTTGGACCTTAGGAATAATTTA
TGCTATAATAGCTATCGGATTATTAGGATTCGTAGTTTGAGCTCACCATATATTTA
CAGTAGATAGATGTAGATACCCGAGCTTACTTCACTTCAGCAACTATAATTATTG
CGGTTCCAACTGGGATTAAAGTCTTTAGATGACTAGCCACCTTTCATGGAACTCA
AATTTTAAATAGACCAGTAACCCTGTGAGCTTTAGGTTTTGTATTTTTATTTACAG
TAGGAGGATTAACAGGGGTAATTTTAGCTAATTCTTCAATTGATATTATTCTCCAC
GATACTTACTATGTAGTAGCTCATTTTCACTATGTTCTTTCTATAGGAGCTGTATT
TGCAATTATAGCCGGAATTGTCCAATGATTCCCATTATTTACCGGACTAACCTTA
AATAATTTTTTTTTAAAAACCCAATTTATTACTATATTTTTAGGTGTAAATTTAACAT
TTTTTCCTCAACACTTTTTAGGGCTAAGAGGAATGCCTCGGCGATATTCGGATTA
TCCAGATGCTTTCACTATCTGAAATGCCATTTCATCTATTGGTTCCATTATTTCTC
TCACAAGAATTATCTTTTTTTTTATTTATCTTATGAGAAAGGCTTTCTTCCCAACGT
AAATCTTTGAGAACTTTAAATGTAACAACTTCAATTGAATGGTTCAAGCCCTACC
CCCTAGTG
EQ1_8
ATTATTAGACAAGAAAGGGGGAAAAAAGAAGCTTTTGGACCTTAGGAATAATTTA
TGCTATAATAGCTATCGGATTATTAGGATTCGTAGTTTGAGCTCACCATATATTTA
CAGTAGATAGATGTAGATACCCGAGCTTACTTCACTTCAGCAACTATAATTATTG
CGGTTCCAACTGGGATTAAAGTCTTTAGATGACTAGCCACCTTTCATGGAACTCA
AATTTTAAATAGACCAGTAACCCTGTGAGCTTTAGGTTTTGTATTTTTATTTACAG
TAGGAGGATTAACAGGGGTAATTTTAGCTAATTCTTCAATTGATATTATTCTCCAC
GATACTTACTATGTAGTAGCTCATTTTCACTATGTTCTTTCTATAGGAGCTGTATT
TGCAATTATAGCCGGAATTGTCCAATGATTCCCATTATTTACCGGACTAACCTTA
AATAATTTTTTTTTAAAAACCCAATTTATTACTATATTTTTAGGTGTAAATTTAACAT
TTTTTCCTCAACACTTTTTAGGGCTAAGAGGAATGCCTCGGCGATATTCGGATTA
TCCAGATGCTTTCACTATCTGAAATGCCATTTCATCTATTGGTTCCATTATTTCTC
TCACAAGAATTATCTTTTTTTTTATTTATCTTATGAGAAAGGCTTTCTTCCCAACGT
AAATCTTTGAGAACTTTAAATGTAACAACTTCAATTGAATGGTTCAAGCCCTACC
CCCTAGTG
EQU_5
ATTATTAGACAAGAAAGGGGGAAAAAAGAAGCTTTTGGACCTTAGGAATAATTTA

TGCTATAATAGCTATCGGATTATTAGGATTCGTAGTTTGAGCTCACCATATATTTA
CAGTAGATAGATGTAGATACCCGAGCTTACTTCACTTCAGCAACTATAATTATTG
CGGTTCCAACTGGGATTAAAGTCTTTAGATGACTAGCCACCTTTCATGGAACTCA
AATTTTAAATAGACCAGTAACCCTGTGAGCTTTAGGTTTTGTATTTTTATTTACAG
TAGGAGGATTAACAGGGGTAATTTTAGCTAATTCTTCAATTGATATTATTCTCCAC
GATACTTACTATGTAGTAGCTCATTTTCACTATGTTCTTTCTATAGGAGCTGTATT
TGCAATTATAGCCGGAATTGTCCAATGATTCCCATTATTTACCGGACTAACCTTA
AATAATTTTTTTTTAAAAACCCAATTTATTACTATATTTTTAGGTGTAAATTTAACAT
TTTTTCCTCAACACTTTTTAGGGCTAAGAGGAATGCCTCGGCGATATTCGGATTA
TCCAGATGCTTTCACTATCTGAAATGCCATTTCATCTATTGGTTCCATTATTTCTC
TCACAAGAATTATCTTTTTTTTTATTTATCTTATGAGAAAGGCTTTCTTCCCAACGT
AAATCTTTGAGAACTTTAAATGTAACAACTTCAATTGAATGGTTCAAGCCCTACC
CCCTAGTG
;

endblock;
